# Nickel-Catalyzed Enantio- and Diastereoselective Synthesis of Fluorine-Containing Vicinal Stereogenic Centers

**DOI:** 10.1021/acscentsci.4c00819

**Published:** 2024-08-17

**Authors:** Uttam Dhawa, Lara Lavrencic, Xile Hu

**Affiliations:** Laboratory of Inorganic Synthesis and Catalysis, Institute of Chemical Sciences and Engineering, École Polytechnique Fédérale de Lausanne (EPFL), ISIC-LSCI, Lausanne 1015, Switzerland

## Abstract

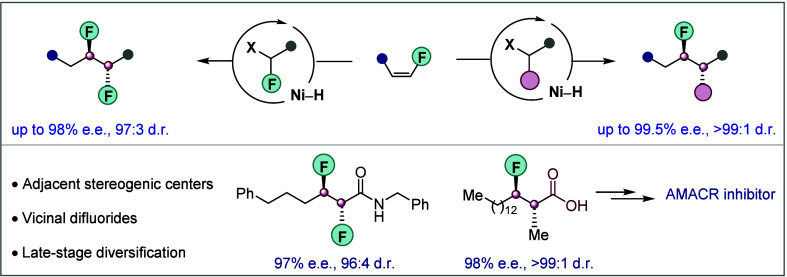

The construction of fluorinated architectures has been a topic of interest to medicinal chemists due to their unique ability to improve the pharmacokinetic properties of bioactive compounds. However, the stereoselective synthesis of fluoro-organic compounds with vicinal stereogenic centers is a challenge. Herein, we present a directing-groupfree nickel-hydride catalyzed hydroalkylation of fluoroalkenes to afford fluorinated motifs with two adjacent chiral centers in excellent yields and stereoselectivities. Our method provides expedient access to biologically relevant, highly enantioenriched organofluorine compounds. Furthermore, the strategy can be used for the diastereo- and enantioselective synthesis of vicinal difluorides, which have recently gained attention in the fields of organocatalysis and peptide mimics.

## Abstract 2

Nickel-catalyzed enantio- and diastereoselective hydroalkylation of fluoroalkenes is developed to give enantioenriched organofluorine compounds bearing adjacent stereocenters.

## Introduction

**Figure 1:**
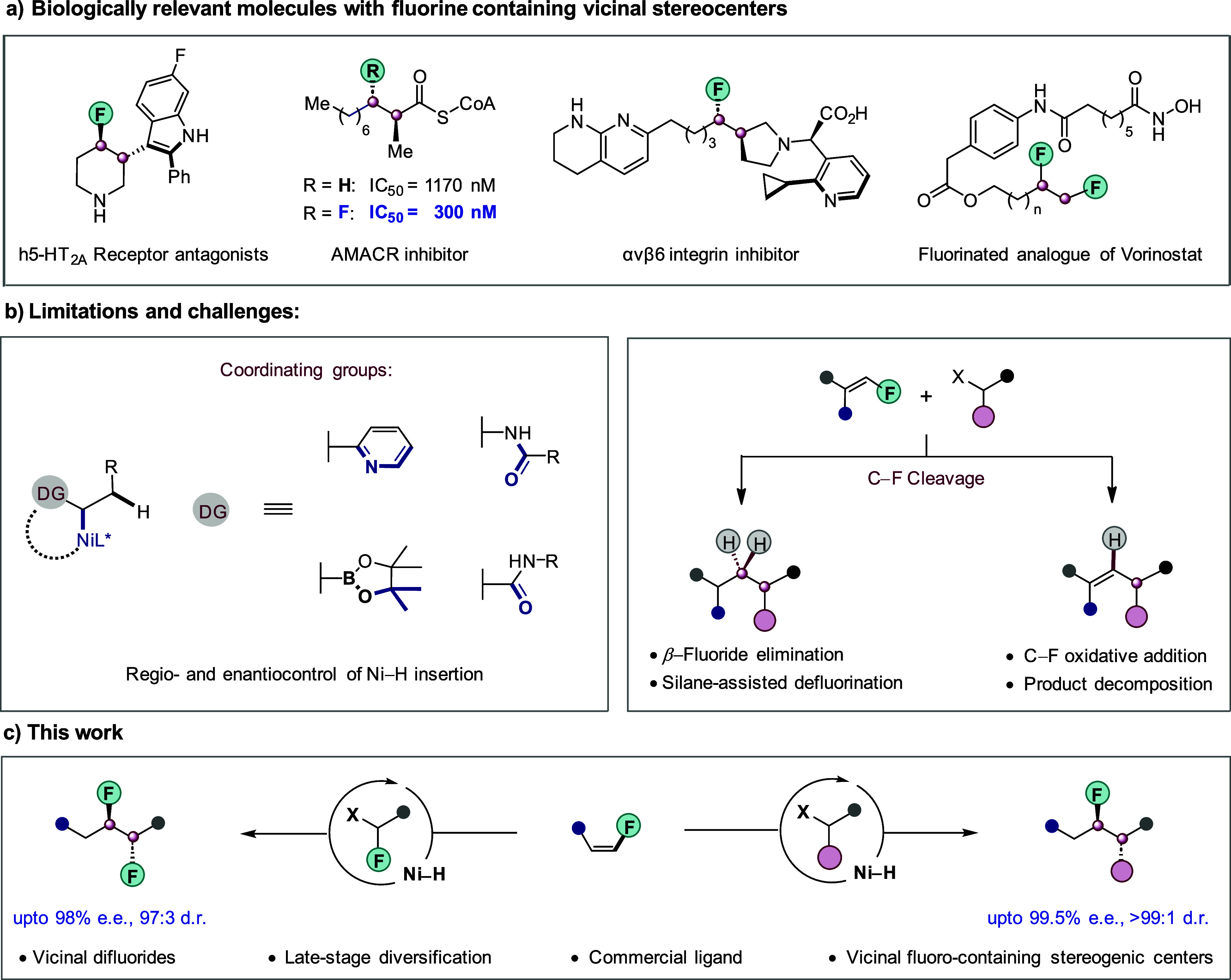
Context of enantio- and diastereoselective synthesis of fluorine-containing vicinal stereogenic centers. (a) Representative examples of biologically relevant molecules. h5-HT_2A_, serotonin 2A receptor, AMACR, α-methylacyl CoA racemase. (b) Limitations and challenges. Left panel: Necessity of a directing group or proximal *p/π* orbital to control reaction regioselectivity. Right panel: unproductive C–F cleavage. (c) This work: Enantio- and diastereoselective coupling of fluoroalkenes with secondary alkyl halides to access compounds with fluorine-containing vicinal stereogenic centers and vicinal difluorides. DG, directing group.

Fluorinated skeletal units are privileged scaffolds in materials,^1^ agrochemicals,^2^ and drug molecules (Figure 1a).^3,4^ The incorporation of fluorine into therapeutic candidates often tailors their potency, lipophilicity, and metabolic stability.^5^ As a consequence, there has been a significant increase in the presence of fluorine in FDA-approved top 100 best-selling small-molecule drugs.^6^ To date, various approaches have been developed to directly access C(sp^3^)–F stereocenters.^7−11^ Nonetheless, these approaches primarily suffer from multistep starting material synthesis and the use of expensive or hazardous fluorinating reagents.^12,13^ There exist few literature precedents where a single catalyst can generate a fluorine-containing chiral center with excellent enantioselectivity, while at the same time also control stereoinduction at the adjacent carbon to selectively form one out of four possible stereoisomers.^14^ Consequently, highly enantio- and diastereoselective synthesis of fluoro-organic compounds remains an outstanding synthetic challenge.^15,16^

Recently, nickel-hydride-catalyzed hydroalkylation of alkenes has emerged as an efficient method for stereoselective C(sp^3^)–C(sp^3^) cross-couplings.^17−19^ However, most reports achieve the introduction of one single enantioenriched C(sp^3^) center,^20−23^ so the generation of contiguous C(sp^3^) stereocenters remains scarce.^24−26^ Moreover, a strongly coordinating auxiliary group,^27^ such as a boryl,^21,26,28^ carbonyl,^24,25,29,30^ or pyridinyl,^31^ is needed to control the regioselectivity of nickel-hydride insertion (Figure 1b, left panel). Stereoselective hydroalkylation of alkenes without a preinstalled directing group is unknown for nickel-hydride catalysis. In particular, a single fluoro substituent could so far not be employed to control reaction regio- and enantioselectivity in nickel catalysis, while a single example of a cobalt-based system was recently reported.^32^ No method to simultaneously construct two adjacent fluorine-containing stereocenters has been developed to date.

We recently became interested in the development of an enantio- and diastereoselective hydroalkylation of fluoroalkenes to access fluoro-organic compounds with vicinal stereocenters. In addition to the lack of a directing group, the main challenges associated with this approach include (a) nickel-catalyzed unproductive C–F oxidative addition (Figure 1b, right panel),^33^ (b) hydrodefluorination, commonly observed in the presence of a Lewis acid and silane,^34−36^ and (c) β-fluoride elimination from the alkyl nickel intermediate.^37^ We also anticipated that stereoinduction at the C–F carbon would be difficult due to the similar size of fluorine and hydrogen atoms.^38^

Herein, we present our successful efforts to overcome these challenges by the judicious choice of a commercially available ligand and reaction conditions. Our method converts readily accessible fluoroalkenes into compounds with vicinal fluorine-containing chiral centers with excellent levels of regio-, enantio-, and diastereocontrol (Figure 1c). Due to its high functional group tolerance, the reported protocol could be applied to the synthesis of numerous organofluorine compounds and late-stage functionalization of biologically relevant molecules. Moreover, it could also be further extended to the synthesis of challenging vicinal difluorides, which are of interest in the fields of organocatalysis^39^ and peptide mimics.^40^

## Results and Discussion

### Ligand Optimization

**Figure 2:**
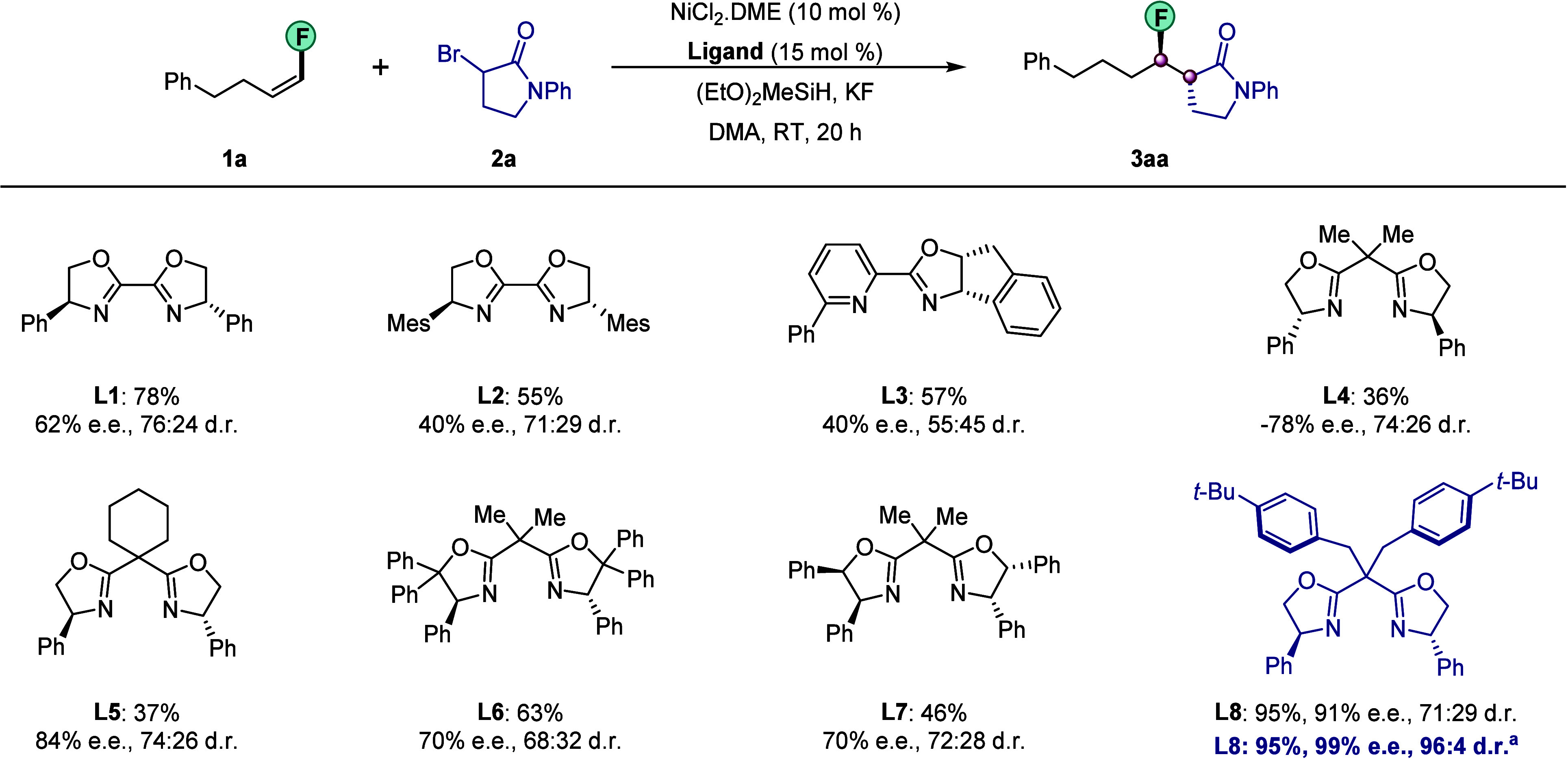
Optimization of ligands. Conditions: NiCl_2_·DME (10 mol %), ligand (15 mol %), **1a** (0.20 mmol), **2a** (0.10 mmol), (EtO)_2_MeSiH (0.25 mmol), KF (0.25 mmol), and DMA (1.0 mL) at RT for 20 h; yields and dr were measured by ^19^F NMR of the crude reaction mixture. The ee was determined using chiral HPLC analysis of the product after purification. ^a^**1a** (0.13 mmol), DMA/*t*-BuOH (0.5:0.5 mL) as solvent, 0 °C, 40 h. Mes, 2,4,6-trimethylphenyl; DME, dimethoxyethane; DMA, *N*,*N*-dimethylacetamide; RT, room temperature.

We initiated our studies by testing various chiral ligands for the envisioned hydroalkylation of fluoroalkene **1a** with lactam **2a** (Figure 2). Initial experimentation with a bioxazoline (BiOX) ligand **L1** provided the desired product **3aa** in good yield and with moderate enantio- and diastereoselectivity of 62% ee and 76:24 dr. A noticeable amount of unproductive C–F cleavage product was also detected in the crude reaction mixture. A sterically bulky mesityl-substituted BiOX **L2** ligand was then tested, but it led to a worse result. A pyridine-oxazoline ligand **L3** similarly resulted in a diminished reaction yield and stereocontrol. Next, a series of bisoxazoline (BOX) ligands **L4**–**L8** was tested. Ligand **L4** provided the desired product **3aa** with improved enantioselectivity but with a poor yield and diastereoselectivity. A structural change on the bridge position from **L4** to **L5** further improved the enantioselectivity to 84% ee, but no change in diastereoselectivity was observed. More sterically bulky ligands **L6** and **L7** failed to provide satisfactory improvements. Nevertheless, a considerable improvement in enantioselectivity was observed when **L8** bearing a *tert*-butyl substituent on its backbone was employed. The desired hydroalkylation product **3aa** was obtained in almost quantitative yield and with 91% ee, albeit with poor diastereoselectivity. We identified temperature as another crucial factor in controlling the stereochemical outcome of the reaction. By lowering the temperature to 0 °C, **3aa** was obtained in excellent yield and with 96% ee and 85:15 dr (Table S1). Then, a systemic screening of various solvent mixtures was performed. We were delighted to find that mixing DMA and *t*-BuOH in a 1:1 ratio provided a great improvement in diastereoselectivity, likely due to noncovalent bonding interactions between the alcoholic solvent and activated complexes. Under the optimized reaction conditions, the desired product **3aa** was now formed in 88% isolated yield and with 99% ee and 96:4 dr. All attempts to perform the same reaction with a cobalt catalyst failed, highlighting the unique ability of our nickel catalysis to form two vicinal carbon stereocenters *via* hydroalkylation (Table S2).

### Substrate Scope

**Figure 3:**
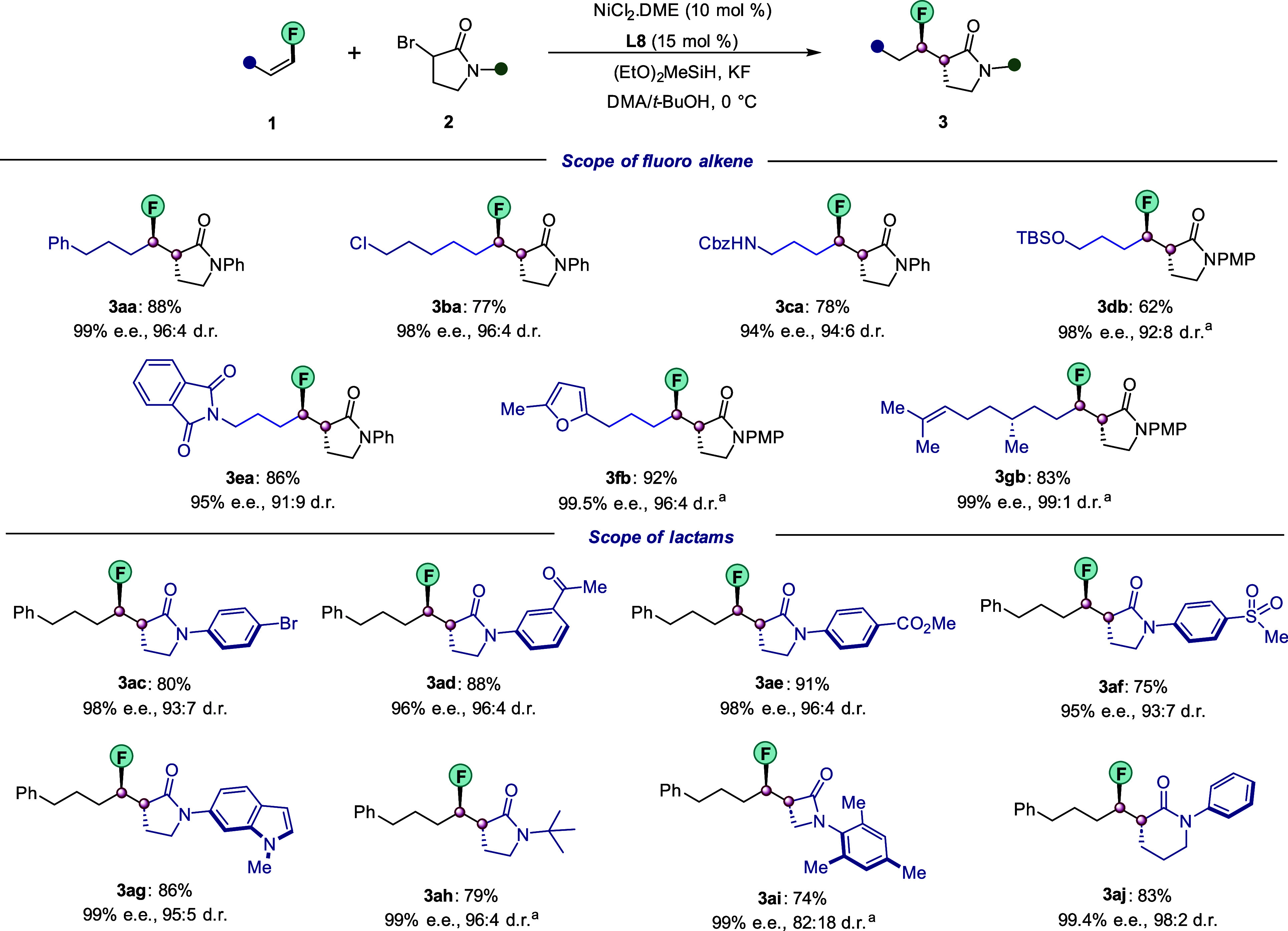
Scope of nickel-catalyzed enantio- and diastereoselective hydroalkylation, part I. Conditions: NiCl_2_·DME (10 mol %), **L8** (15 mol %), **1a** (0.13 mmol), **2a** (0.10 mmol), (EtO)_2_MeSiH (0.25 mmol), KF (0.25 mmol), and DMA/*t*-BuOH (0.5:0.5 mL) at 0 °C for 40 h; dr was measured by ^19^F NMR of the crude reaction mixture. The ee was determined using chiral HPLC analysis of the product after purification. ^a^**1a** (0.15 mmol). PMP, 4-methoxyphenyl. Yields refer to isolated yields of the major diastereomer (>99:1) except for **3ea** (isolated dr 91:9), **3ac** (92:8), **3ad** (98:2), **3ae** (96:4), **3af** (95:5), and **3ai** (82:18).

With the optimized conditions in hand, we probed the scope of the reaction by reacting a wide variety of fluoroalkenes **1** with lactams **2** (Figure 3). All of the fluoroalkenes used could be prepared from their corresponding aldehydes in a simple, one-step procedure. We were pleased to find that the coupling was, in all cases, highly regioselective at the carbon bearing the electronegative fluorine atom. Overall, the products **3** were formed in uniformly high enantio- and diastereomeric purities and high yields. Various functional groups including alkyl chloride (**1b**), protected amine (**1c**), and alcohol (**1d**) on the fluoroalkenes were well tolerated. Furthermore, fluoroalkenes containing medicinally relevant heterocycles such as phthalimide (**1e**) and furan (**1f**) were viable substrates. When substrate **1g** containing multiple unsaturated bonds was tested, a high degree of chemoselectivity was observed. This fluoroalkene **1g,** derived from (*S*)-citronellol, provided **3gb** in 83% yield with 99% ee and 99:1 dr.

Likewise, a series of synthetically useful functional groups could also be incorporated into lactam partners **2**. For example, bromo (**2c**)-, ketone (**2d**)-, ester (**2e**)-, sulfone (**2f**)-, and indole (**2g**)-containing substrates underwent hydroalkylation to yield highly enantioenriched products **3ac**–**3ag**. The reaction of an alkyl-substituted lactam **2h** was also highly stereoselective, affording the desired product **3ah** with 99% ee and 96:4 dr. Moreover, lactams of different ring sizes could be successfully engaged in this transformation. Six-membered lactam **2j** provided the desired product **3aj** in 99.4% ee with 98:2 dr whereas the reaction of four-membered lactam **2i** showed a slight drop in diastereoselectivity (82:18 dr). The structure of **3ac** was confirmed by single-crystal X-ray diffraction analysis.

**Figure 4:**
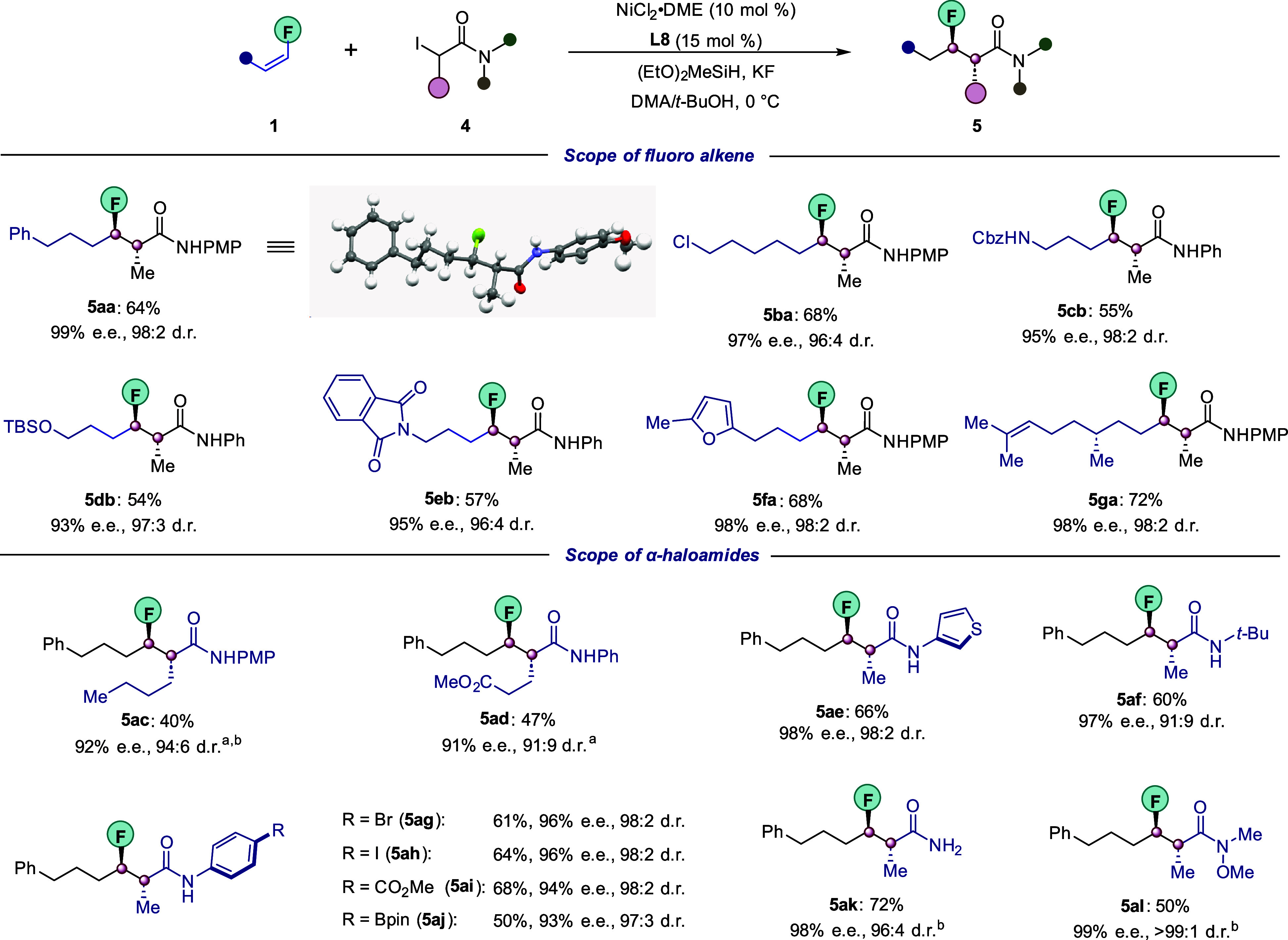
Scope of nickel-catalyzed enantio- and diastereoselective hydroalkylation, part II. Conditions: NiCl_2_·DME (10 mol %), **L8** (15 mol %), **1** (0.20 mmol), **4** (0.10 mmol), (EtO)_2_MeSiH (0.25 mmol), KF (0.25 mmol), and DMA/*t*-BuOH (0.5:0.5 mL) at 0 °C for 60 h; dr was measured by ^19^F NMR of the crude reaction mixture. The ee was determined using chiral HPLC analysis of the product after purification. Yields refer to isolated yields of the major diastereomer (>98:2). ^a^NiCl_2_·DME (15 mol %), **L8** (22.5 mol %). ^b^Alkyl bromide with 1 equiv of KI was used.

We next investigated whether our protocol could be extended to the coupling of linear secondary alkyl amides **4**. In contrast to the previous protocol from our group which remained restricted to cyclic alkyl amides,^26^ our current methodology could be applied to a plethora of acyclic α-halo substrates without any detriment to the enantio- and diastereopurity of the desired products **5** (Figure 4). Fluoroalkenes bearing functional groups such as chloro (**1b**), protected amine (**1c**), and alcohol (**1d**) were all viable coupling partners, as exemplified by corresponding products **5ba**–**5db**. Excellent enantio- and diastereocontrol was also observed for heterocycles, including phthalimide **1e** and furan **1f**. Importantly, the fluoroalkene derived from (*S*)-citronellol provided the desired **5ga** with 98% ee and 98:2 dr.

Next, a wide range of racemic α-alkyl-α-iodoamides **4** were also explored under the standard conditions. Secondary electrophiles with longer side chains, which are either fully aliphatic (**4c**) or bear an ester functionality (**4d**), could be efficiently coupled with fluoroalkene **1a** with excellent enantio- and diasteroselectivites. Moreover, an electron-rich *N*-heteroaryl amide **(4e)** as well as aliphatic (**4f**) were both suitable coupling partners. Gratifyingly, our protocol was well applicable to substrates containing various functionalities on the arene ring, such as bromo (**4g**), iodo (**4h**), ester (**4i**), and Bpin (**4j**) groups, which provides reactive moieties for late-stage modification. The catalytic system was also not limited to the coupling of secondary aryl amides. Indeed, more challenging primary (**4k**) and tertiary amides (**4l**) were also viable substrates. It is noteworthy to mention that the latter is a synthetically versatile Weinreb amide which provided the corresponding product **5al** as a single diastereomer with 99% ee.

Recently, the invention of novel bioisosteres has gained increasing interest in medicinal chemistry. In this context, vicinal difluoroethane motifs are of particular interest, as they align with the biological fingerprint of ethyl and trifluoromethyl groups.^41,42^ Furthermore, the stereoelectronic gauche effect of a vicinal difluoro motif has been found to modulate the bioactivity of drug molecules while also finding significant application in organocatalysis^39^ and the field of peptide mimics.^40^

**Figure 5:**
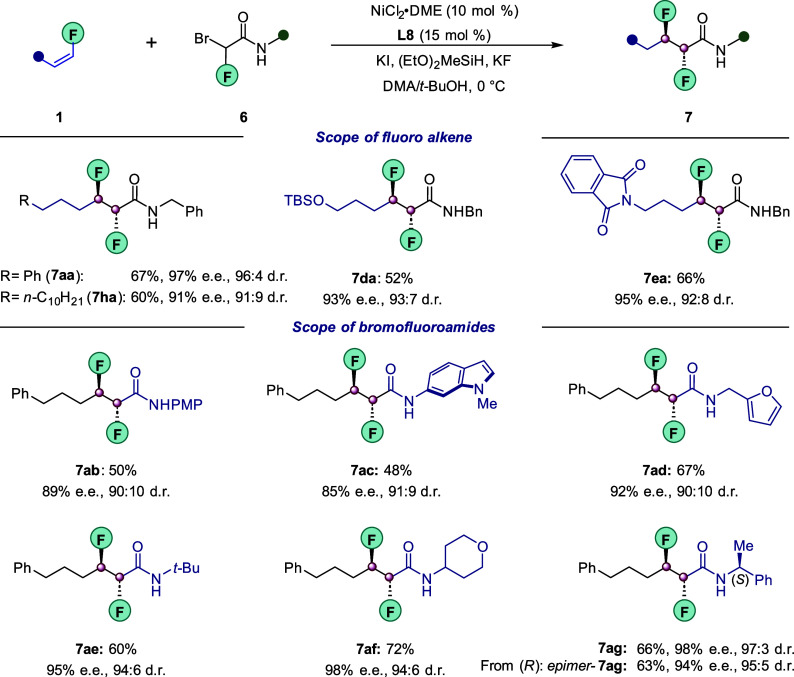
Synthesis of vicinal difluorides. Conditions: NiCl_2_·DME (10 mol %), **L8** (15 mol %), **1a** (0.20 mmol), **2a** (0.10 mmol), (EtO)_2_MeSiH (0.25 mmol), KF (0.25 mmol), KI (0.10 mmol) and DMA/*t*-BuOH (0.5:0.5 mL) at 0 °C for 60 h. Yields refer to isolated yields of the major diastereomer (>99:1). The dr was measured by ^19^F NMR of the crude reaction mixture. The ee was determined using chiral HPLC analysis of the product after purification.

Despite some early contributions,^43−46^ synthetic routes that generate two contiguous fluorinated carbons in a highly enantio- and diastereocontrolled fashion continue to be scarce and are often synthetically laborious.^47^ With this in mind, we were particularly pleased to demonstrate that our chemistry can be successfully applied to the coupling of challenging bromo-fluoroamides **6**. As illustrated in Figure 5, under nearly identical reaction conditions, the couplings between fluoroalkenes **1** and bromo-fluoroamides **6** proceeded efficiently to furnish the desired vicinal difluorides **7** in moderate to good yields. Excellent enantio- and diasteroselectivities were observed regardless of the fluoroalkene chain lengths (**1a** and **1h)**. Likewise, the coupling showed good functional group tolerance, as both the alcohol-derived **1d** and phtalimide-bearing **1e** were tolerated. We also found that the substitution pattern on the nitrogen of the substrate’s amide functionality did not affect the reaction performance: substrates bearing aryl (**6b**), heteroaryl (**6c**), furfuryl (**6d**), alkyl (**6e**), tetrahydropyran (**6f**), and chiral benzyl (**6g**) amides all reacted well.

### Synthetic Application

**Figure 6:**
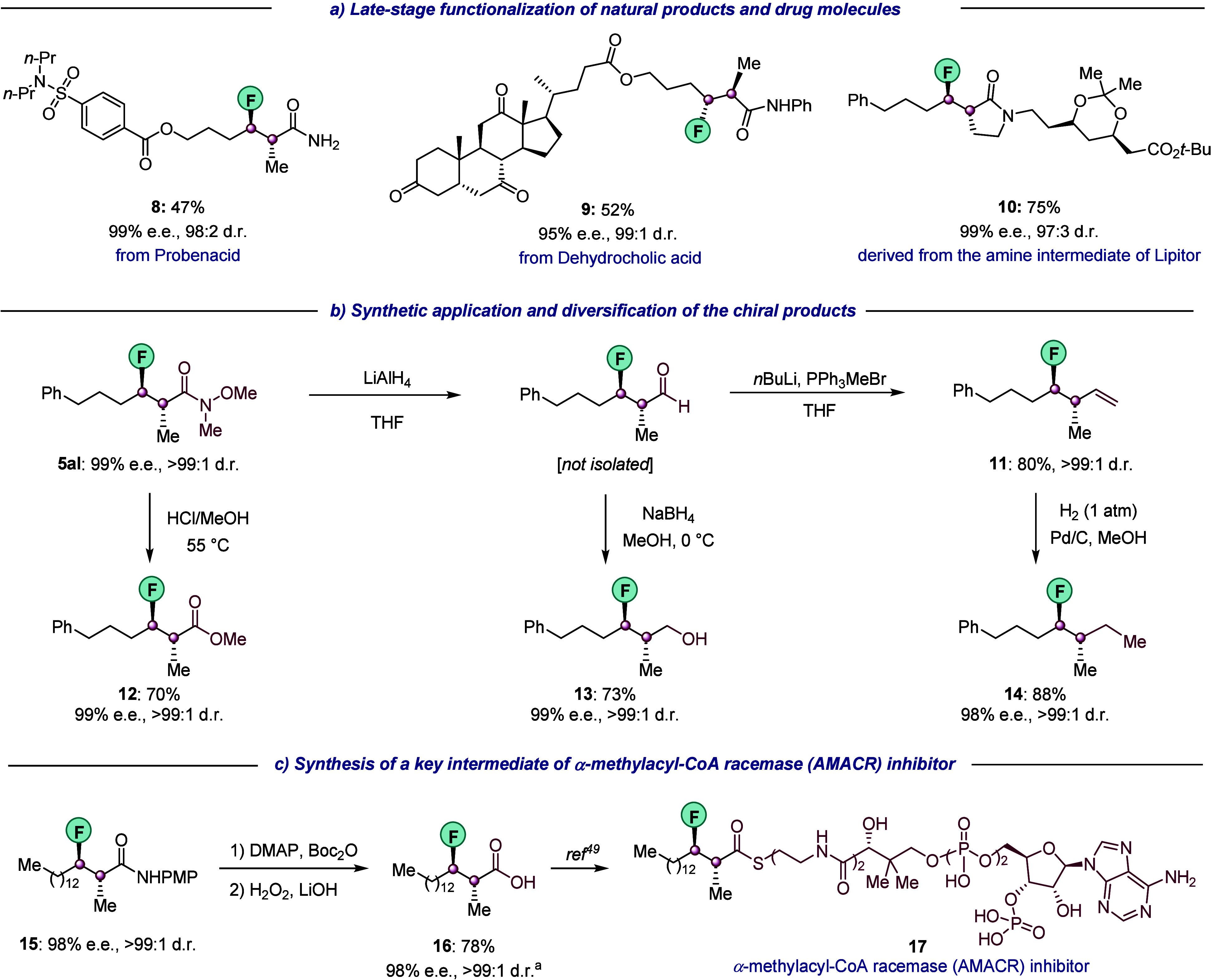
Synthetic application. (a) Functionalization of drug and natural product derivatives. (b) Synthetic elaboration of hydroalkylation products to enantioenriched organofluorine compounds of value to asymmetric synthesis. (c) Synthesis of a key intermediate in the preparation of an α-methylacyl-CoA racemase (AMACR) inhibitor. See Supporting Information for full details. ^a^The ee was determined after performing a subsequent transformation. THF, tetrahydrofuran; DMAP, 4-dimethylaminopyridine.

We next applied our methodology to the late-stage modification of biologically relevant and structurally complex drug molecules (Figure 6a). Fluoroalkenes derived from probenecid (a drug used for treating gout and hyperuricemia) and from dehydrocholic acid (a synthetic bile acid) underwent hydroalkylation in moderate yields but with excellent enantio- and diastereoselectivities (compounds **8** and **9**, respecitively). In addition, the coupling of a lactam derived from an amine intermediate used in the synthesis of Lipitor proceeded unimpeded to furnish the desired product **10** in 75% yield with excellent stereopurity (99% ee, 97:3 dr).

Compounds containing a fluorine atom attached to one of the two vicinal enantiopure carbon centers are often recognized as key intermediates in the asymmetric synthesis. We further demonstrated the utility of our method by constructing such valuable synthons from our products (Figure 6b). The Weinreb amide motif in **5al** was successfully applied in Fisher esterification to afford the methyl ester **12**. **5al** could also be engaged in two sequential reduction steps to afford the alcohol derivative **13**, or converted into alkene **11***via* a reduction and Wittig olefination sequence. The latter was then reduced in the presence of Pd/C to its alkane analogue **14**. It is noteworthy to mention that **14** formally bears a methyl substituent on its main aliphatic chain and that selective methylation is a challenging transformation in the field of medicinal chemistry.^48^ With our approach, we can therefore incorporate fluorine next to a chiral center that bears the two smallest alkyl substituents. In general, the enantio- and diastereopurities of the starting compounds were well preserved in all of these downstream transformations.

The appealing utility of our chemistry was further exhibited by the preparation of a key intermediate in the synthesis of α-methylacyl-CoA racemase (AMACR) inhibitor **17**, which has been investigated as an anticancer agent (Figure 6c).^49^ First, our hydroalkylation method provided the compound **15** in a high yield and with excellent ee and dr (see Supporting Information). A subsequent mild hydrolysis provided the free acid **16** which can be converted into **17**, by following an established procedure.^49^ By comparison, previous methods to prepare intermediate **16** relied on the use of Evan’s auxiliary for chiral induction which required additional steps for installation and removal.^50^

### Mechanistic Investigation

**Figure 7:**
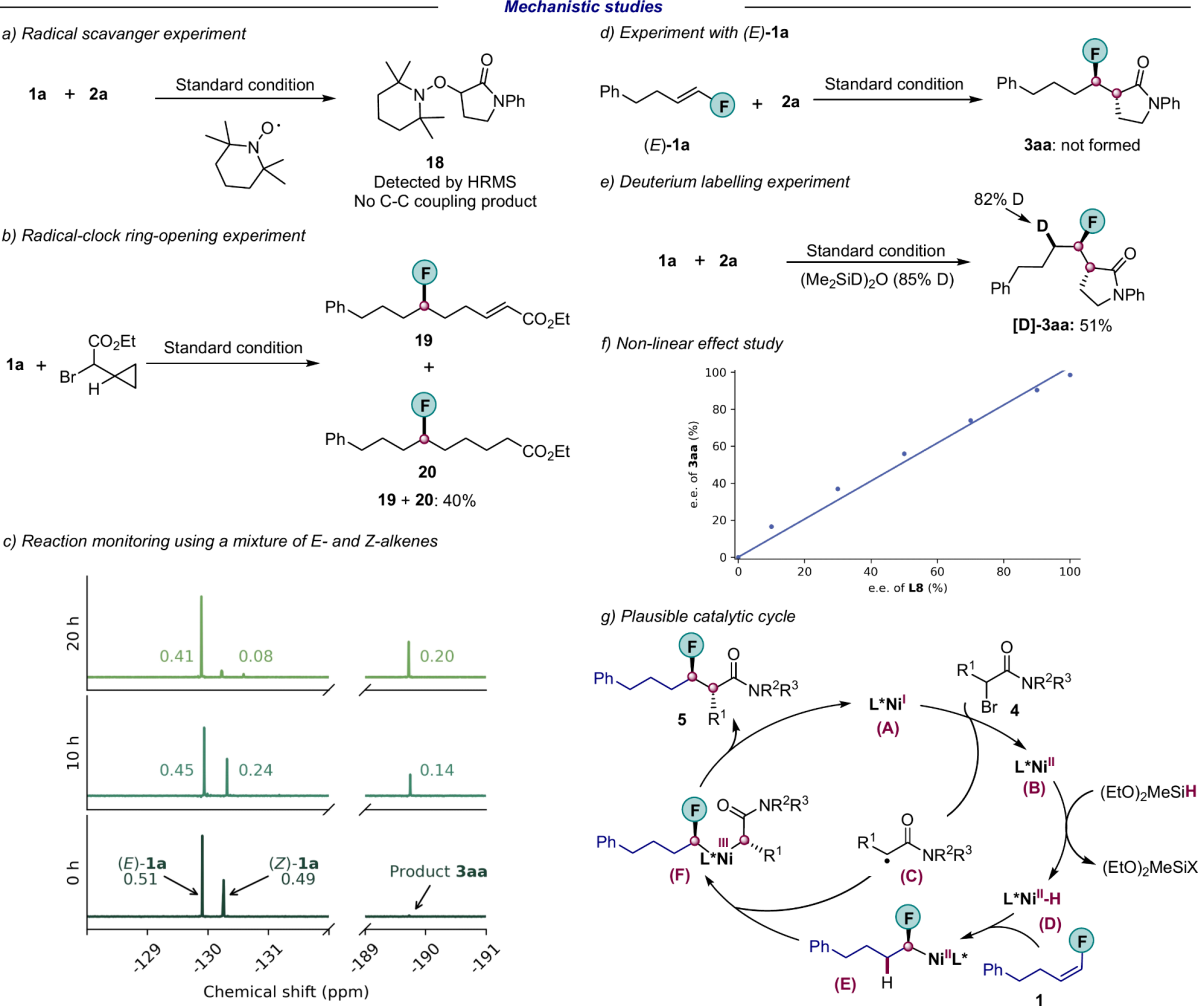
Mechanistic study. (a) An experiment using a radical scavenger TEMPO performed under the standard reaction conditions. (b) A radical clock experiment. (c) ^19^F NMR spectra for *Z*/*E* isomerization tracking experiments using PhCF_3_ as internal standard. Amounts of the product and alkene isomers are given as equivalents per starting lactam (0.4 mmol, 1 equiv). (d) Experiment with pure (*E*)-**1a**. (e) A deuterium labeling experiment performed under the standard reaction conditions. (f) Nonlinear effect study. (g) Outline of a plausible mechanism. See Supporting Information for full details of a–f. HRMS, high-resolution mass spectrometry.

Key experiments were performed to shed light onto the reaction mechanism. When a radical scavenger, TEMPO (2,2,6,6-tetramethylpiperidin-1-oxyl), was added to the reaction mixture, the C(sp^3^)–C(sp^3^) coupled product was not formed, while an alkyl-TEMPO adduct **18** was detected (Figure 7a). The coupling of ethyl 2-bromo-2-cyclopropyl ester, a classical radical clock substrate, provided ring-opening products **19** and **20** (Figure 7b). These results indicate the presence of alkyl radical species formed *via* the activation of secondary alkyl halide electrophiles. When the reaction progress was monitored by ^19^F NMR, we did not observe any isomerization of (*Z*)-**1a** into its (*E*)-counterpart (Figures S8 and S10). A 1:1 mixture of both stereoisomers was also separately prepared and used in the same experiment (Figure 7c and Figures S9 and S10). (*E*)-**1a** was left largely unreacted, and we linked the observed product formation only to the consumption of (*Z*)-**1a**. Carrying out our reaction with pure (*E*)-**1a** gave no **3aa** (Figure 7d and Figures S11 and S12). According to previous studies, metal-hydride insertions across double bonds are often highly sensitive to alkene stereochemistry.^51,52^ We think that such strong stereopreference of our developed catalytic protocol is likely of electronic origin. Next, a deuterium labeling experiment was conducted to gain insight into the selectivity of Ni–H insertion step using (Me_2_SiD)_2_O (85% D) as hydride source (Figure 7e). An almost quantitative deuterium incorporation was observed exclusively at the *β*-position to the fluorine atom. The product was isolated as a single diastereoisomer without any deuterium scrambling, implying *syn*-hydronicklellation as an enantiodetermining event. Additionally, we did not observe a nonlinear effect, suggesting that the enantiodetermining step involves a species with a nickel to ligand ratio of 1:1 (Figure 7f).

On the basis of these studies and literature precedents,^26^ a plausible catalytic cycle is proposed in Figure 7g. The catalytic cycle starts with the generation of a Ni(I) species (**A**) which activates the secondary alkyl halide **4** through single-electron transfer to provide a Ni(II) species (**B**) and an alkyl radical (**C**). Next, Ni(II) species (**B**) reacts with silane to form Ni(II)–H (**D**). Subsequent enantioselective hydrometalation into fluoroalkene **1** forms a Ni(II)−α-fluoroalkyl complex (**E**) which is trapped by the secondary alkyl radical to form a Ni(III) species (**F**). Finally, reductive elimination from (**F**) provides coupling product **5** and regenerates Ni(I) species (**A**) to complete the cycle. At this point, we cannot exclude the possibility that the order of substrate engagement is reversed, that is, that the Ni(I) species (**A**) reacts first with silane and then with fluoroalkene **1** to give a Ni(I)–alkyl species (**H**) (Figure S15, path b). The latter then activates **4** to generate an alkyl radical while being transformed into a Ni(II)–alkyl species (**I**). The details of the mechanism are subject to future studies.

## Conclusion

In summary, we have developed a method for regio-, enantio-, and diastereoselective hydroalkylation of fluoroalkenes with secondary alkyl halides bearing both cyclic and linear amide moieties. Our method provides expedient access to a large number of valuable fluorinated compounds bearing vicinal chiral carbon centers. This protocol is general and is tolerant of a broad range of functional groups. Its utility was demonstrated by the synthesis of elusive vicinal difluorides and the late-stage functionalization of substrates derived from natural products and drug molecules. The current work provides new avenues to nickel-hydride chemistry, as alkenes without a directing group can now be used. We anticipate that our chemistry will add to the toolbox of valuable asymmetric methods for the synthesis of bioactive and pharmacoactive fluorinated molecules.

## Methods

### General Procedure: Enantio- and Diastereoselective Hydroalkyaltion of Fluoroalkene

To an oven-dried 10 mL Teflon-lined screw capped vial equipped with a stirring bar was added **L8** (15 mol %, 0.015 mmol). The vial was introduced in a nitrogen-filled glovebox. Nickel(II) chloride ethylene glycol dimethyl ether complex (2.2 mg, 10 mol %, 0.01 mmol) and anhydrous DMA/*t*-BuOH (0.50/0.50 mL) were added, and the mixture was stirred for 1.5 h at room temperature until it became a clear pink solution. Then the racemic electrophile (0.10 mmol, 1.0 equiv), anhydrous KF (14.5 mg, 0.25 mmol, 2.5 equiv), (*Z*)-fluoroalkene (0.13–0.20 mmol, 1.3–2.0 equiv), and methyldiethoxysilane (40.5 μL, 0.25 mmol, 2.5 equiv) were added to the reaction mixture in this sequence. The vial was then wrapped with airtight electrical tapes, removed from the glovebox, and stirred for 40–60 h at 0 °C, maintaining 520 rpm. After that, the reaction was diluted with EtOAc and evaporated under vacuum to remove the residual solvents. The crude mixture was purified by automated flash column chromatography to yield the products. No unexpected or unusually high safety hazards were encountered.

## Data Availability

The authors declare that the data supporting the findings of this study are available within the paper and its Supporting Information files. Raw data files are available at Zenodo online repository: doi: 10.5281/zenodo.13284985. All other requests for materials and information should be addressed to the corresponding authors.

## Supporting Information Available

Experimental details, crystallography details, and NMR spectra (PDF)
X-ray crystal structure data for **3ac** (CIF)
X-ray crystal structure data for **5aa** (CIF)

## Supplementary Material

oc4c00819_si_001.pdf

oc4c00819_si_002.cif

oc4c00819_si_003.cif
