# Supplementary material for: Nickel-Catalyzed Enantio- and Diastereoselective Synthesis of Fluorine-Containing Vicinal Stereogenic Centers
Source: ACS Cent Sci. 2024 Aug 17;10(8):1657–66. doi: 10.1021/acscentsci.4c00819 (PMC11363326; doi:10.1021/acscentsci.4c00819)
Supplement: Supplementary file 1 — oc4c00819_si_001.pdf [file oc4c00819_si_001.pdf]

Supporting Information

Nickel-Catalyzed Enantio- and Diastereoselective Synthesis of Fluorine-Containing Vicinal Stereogenic Centers

Uttam Dhawa,[†] Lara Lavrencic[†] and Xile Hu*

Laboratory of Inorganic Synthesis and Catalysis, Institute of Chemical Sciences and Engineering, École Polytechnique Fédérale de Lausanne (EPFL), ISIC-LSCI, Lausanne, Switzerland

Correspondence to: xile.hu@epfl.ch; [†]These authors contributed equally.

Table of Contents

1. Instrumentation and Chemicals S3

2. General Procedure for Enantio- and Diastereoselective Hydroalkylation S5

3. Synthesis of Starting Materials S6

4. Reaction Optimization S15

5. Product Characterization Data S22

6. Synthetic Application S89

7. Mechanistic Experiments S106

8. Crystallography Details S120

9. NMR Spectra S127

10. References S262

1. Instrumentation and Chemicals:

All reactions for the nickel-catalyzed hydroalkylation were set up in 10 mL Teflon-screw capped vials (unless otherwise noted) under an inert nitrogen (N_2) atmosphere using glove-box techniques. Solvents were bought from the commercial sources and transferred to the glovebox without exposure to air.

NMR: ^1H, ^{13}C and ^{19}F NMR spectra were recorded on a Bruker Advance 400 Spectrometer. ^1H and $^{13}C\{1H\}$ chemical shifts were referenced internally to residual solvent peaks relative to TMS (δ = 0 ppm) at 299 K.

TLC: Merck silica gel 60 F 254 plates; detection with UV light or by dipping into a solution of $KMnO_4$ (1.5 g in 400 mL H_2O, 5.0 g $NaHCO_3$), followed by heating.

Flash column chromatography (FC): Flash column chromatography was performed using silica gel (Silicycle, ultra-pure grade). Preparative Thin Layer Chromatography (PTLC) was performed using glass plates from Merck KGaA, Darmstadt, Germany. The eluents for column chromatography and PTLC were presented as ratios of solvent volumes.

Automated flash column chromatography : Automated flash column chromatography was performed using Biotage® Isolera™ One Flash Chromatography Instrument on Biotage® Sfär Silica D Duo 60 µm columns (10 or 25 g with flowrates of 40 and 80 mL/min, respectively) with detection at 245 and 280 nm.

Preparative Thin Layer Chromatography (pTLC): Preparative thin layer chromatography was performed using silica-coated glass plates from Merck KGaA, Darmstadt, Germany. The eluents are presented as ratios of solvent volumes.

GC and GC-MS: All GC analyses were performed on a Perkin-Elmer Clarus 400 GC system with an FID detector. All GC-MS analyses were performed on an Agilent Technologies 7890A GC system equipped with a 5975C MS detector.

HPLC: High-performance liquid chromatography was performed on an Agilent HPLC instrument. The utilized column (with a chiral stationary phase), eluent mixture, and retention times used for the determination of enantiomeric excess (e.e.) are given below in the details of relevant experiments.

Optical rotations were measured on a Polartronic M polarimeter using a 0.5 cm cell with a Na 589 nm filter. The specific solvents, temperatures, concentrations (in g / 100 mL) are indicated.

High-resolution mass spectra (HRMS): by electrospray ionization (ESI), atmospheric pressure chemical ionization (APCI) and atmospheric pressure photoionization (APPI) method were performed at the EPFL ISIC Mass Spectroscopy Service.

All reagents were either prepared according to reported methods or purchased from Sigma Aldrich, TCI, Acros Organics, Alfa Aesar, Fluorochem, Enamine and ABCR. Anhydrous $NiCl_2$·DME from Sigma Aldrich, anhydrous *t*-BuOH and KF from Alfa Aesar, KI from ABCR, $(EtO)_2MeSiH$ and anhydrous DMA from Acros Organics were used.
Racemic compounds were prepared using a racemic ligand.

2. General Procedure for Enantio- and Diastereoselective Hydroalkylation

2.1 General Procedure 1: Enantio- and Diastereoselective Hydroalkylation of Fluoroalkenes with Lactams

To an oven-dried 10 mL Teflon-screw capped vial equipped with a stirring bar was added ligand **L8** (15 mol%, 0.015 mmol). The vial was introduced in a nitrogen-filled glovebox. Nickel(II) chloride ethylene glycol dimethyl ether complex (2.2 mg, 10 mol%, 0.01 mmol) and anhydrous DMA/*t*-BuOH (0.5/0.5 mL) were added and the mixture was stirred for 1.5 hour at room temperature until it became a clear pink solution. Then, anhydrous KF (14.5 mg, 0.25 mmol, 2.5 equiv.), lactam **2** (0.10 mmol, 1.0 equiv.), (*Z*)-fluoroalkene **1** (0.13 mmol, 1.3 equiv.) and $(EtO)_2MeSiH$ (40.5 µL, 0.25 mmol, 2.5 equiv.) were added to it in sequence. The vial was then wrapped with airtight electrical tapes, removed from the glove box and stirred in an ice-water bath at 0 °C for 40 hours, maintaining 520 rpm. After that, the reaction was diluted with EtOAc, transferred into a one-necked flask, and the solvents were removed under vacuum. Then, NMR analysis was performed to determine the crude reaction yield and product d.r. (trifluorotoluene was used as internal standard). The crude mixture was purified by automated flash column chromatography to yield the products **3**.

2.2 General Procedure 2: Enantio- and Diastereoselective Hydroalkylation of Fluoroalkenes with Acyclic Amides and Bromofluoroamides

To an oven-dried 10 mL Teflon-screw capped vial equipped with a stirring bar was added ligand **L8** (15 mol%, 0.015 mmol). The vial was introduced in a nitrogen-filled glovebox. Nickel(II) chloride ethylene glycol dimethyl ether complex (2.2 mg, 10 mol%, 0.01 mmol) and anhydrous DMA/*t*-BuOH (0.5/0.5 mL) were added and the mixture was stirred for 1.5 hour at room temperature until it became a clear pink solution. Then anhydrous KF (14.5 mg, 0.25 mmol, 2.5 equiv.), a racemic alkyl halide **4** or **6** (0.10 mmol, 1.0 equiv.), and (*Z*)-fluoroalkene **1** (0.20 mmol, 2.0 equiv.) were added to it. If KI (16.6 mg, 0.1 mmol, 1 equiv.) was used, it was added at this point. Then, $(EtO)_2MeSiH$ (40.5 µL, 0.25 mmol, 2.5 equiv.) was added to the reaction mixture and the vial was wrapped with airtight electrical tapes, removed from the glove-box, and stirred for 60 h at 0 °C, maintaining 520 rpm. Afterward, the general procedure (**GP1**) for analysis of the crude reaction mixture and product isolation was followed.

3. Synthesis of Starting Materials

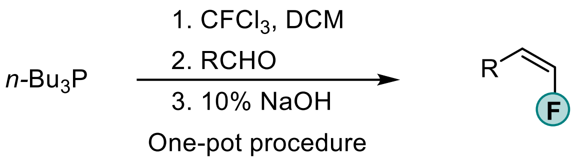

3.1 General procedure for the Synthesis of Fluoroalkenes

A modified literature procedure[1] was followed: A 250 mL two-necked flask, equipped with magnetic stir bar was charged with tri-*n*-butylphosphine (3.75 equiv.) and DCM (0.5 M). The solution was cooled in an ice bath and trichlorofluoromethane (1.25 equiv.) was added in one portion. The resultant mixture was stirred at 0°C for 1h and then at room temperature overnight. Then, the corresponding aldehyde (1 equiv.) was added in one portion and the reaction was stirred for another 8 h at room temperature. After that, 10% NaOH was added to the reaction mixture followed by stirring overnight. Then, the reaction mixture was washed with 40% sodium bisulfite (3 x 25 mL) and water (3 x 25 mL), and the organic portion dried over anhydrous sodium sulfate. After solvent removal, flash column chromatography afforded the desired products **1**.

Compounds **1a**, **1c**, **1e** were prepared according to previous literature procedure on gram-scale.[2]

(*Z*)-6-Chloro-1-fluorohex-1-ene (1b)

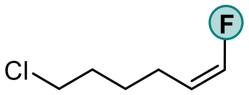

Z/E = 93:7

^{1}H NMR (400 MHz, $CDCl_3$) δ 6.47 (ddt, *J* = 85.7, 4.7, 1.5 Hz, 1H), 4.72 (dtd, *J* = 43.0, 7.7, 4.7 Hz, 1H), 3.55 (t, *J* = 6.6 Hz, 2H), 2.16 (qt, *J* = 7.4, 1.6 Hz, 2H), 1.87 – 1.74 (m, 2H), 1.62 – 1.48 (m, 2H). *Please note: Compound is volatile. Unassigned peaks in the spectrum correspond to residual n-hexane solvent.*

^{13}C NMR (101 MHz, $CDCl_3$) δ 148.21 (d, $^1J_{C–F}$ = 256.6 Hz), 110.38 (d, $^2J_{C–F}$ = 5.5 Hz), 44.91, 29.84 (d, $^3J_{C–F}$ = 4.5 Hz), 26.46 (d, $^5J_{C–F}$ = 2.1 Hz), 22.02 (d, $^4J_{C–F}$ = 5.2 Hz). *Please note: Compound is volatile. Unassigned peaks in the spectrum correspond to residual n-hexane solvent.*

^{19}F NMR (377 MHz, $CDCl_3$) δ -129.87 (*E*-isomer), -130.51 (*Z*-isomer).

EI-MS (m/z, relative intensity): 136 (M^+, 5), 116 (20), 90 (40), 85 (100), 59 (90), 41 (50).

(*Z*)-*tert*-Butyl((4-fluorobut-3-en-1-yl)oxy)dimethylsilane (1d)

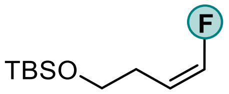

Z/E = 98:2

^{1}H NMR (400 MHz, $CDCl_3$) δ 6.48 (ddt, *J* = 85.8, 4.8, 1.5 Hz, 1H), 4.82 (dtd, *J* = 43.6, 7.5, 4.8 Hz, 1H), 3.63 (t, *J* = 6.6 Hz, 2H), 2.38 – 2.28 (m, 2H), 0.89 (s, 9H), 0.06 (s, 6H).

^{13}C NMR (101 MHz, $CDCl_3$) δ 148.60 (d, $^1J_{C–F}$ = 257.0 Hz), 107.72 (d, $^2J_{C–F}$ = 5.1 Hz), 62.44 (d, $^4J_{C–F}$ = 2.2 Hz), 26.72 (d, $^3J_{C–F}$ = 4.5 Hz), 26.05, 18.47, -5.16.

^{19}F NMR (377 MHz, $CDCl_3$) δ -128.15 (*E*-isomer), -129.51 (*Z*-isomer).

HRMS (Sicrit plasma/LTQ-Orbitrap) m/z: $[M + H]^+$ Calcd for $C_{10}H_{22}FOSi^+$ 205.1418; Found 205.1418.

(*Z*)-2-(4-Fluorobut-3-en-1-yl)-5-methylfuran (1f)

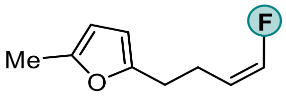

Z/E = 96:2

^{1}H NMR (400 MHz, $CDCl_3$) δ 6.45 (ddt, *J* = 85.5, 4.8, 1.6 Hz, 1H), 5.86 (dd, *J* = 15.6, 3.0 Hz, 2H), 4.77 (dtd, *J* = 43.1, 7.4, 4.6 Hz, 1H), 2.65 (t, *J* = 7.4 Hz, 2H), 2.44 (tdd, *J* = 7.3, 5.8, 1.9 Hz, 2H), 2.25 (s, 3H).

^{13}C NMR (101 MHz, $CDCl_3$) δ 153.33, 150.47, 148.01 (d, $^1J_{C–F}$ = 257.2 Hz), 109.88 (d, $^2J_{C–F}$ = 5.1 Hz), 105.80, 105.70, 27.66 (d, $^4J_{C–F}$ = 1.9 Hz), 21.42 (d, $^3J_{C–F}$ = 5.1 Hz), 13.50.

^{19}F NMR (377 MHz, $CDCl_3$) δ -128.75 (*E*-isomer), -129.90 (*Z*-isomer).

The analytical data correspond with those reported in the literature.[3]

(*R*,*Z*)-1-Fluoro-4,8-dimethylnona-1,7-diene (1g)

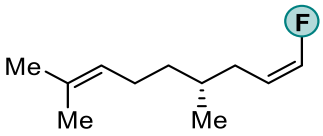

Z/E = 90:10

^{1}H NMR (400 MHz, $CDCl_3$) δ 6.63 – 6.31 (m, 1H), 5.19 – 5.01 (m, 1H), 4.72 (dtd, *J* = 43.5, 7.8, 4.8 Hz, 1H), 2.16 – 2.05 (m, 1H), 2.05 – 1.91 (m, 3H), 1.69 (s, 3H), 1.61 (s, 3H), 1.55 – 1.44 (m, 1H), 1.41 – 1.28 (m, 1H), 1.24 – 1.10 (m, 1H), 0.90 (d, *J* = 6.7 Hz, 3H).

^{13}C NMR (101 MHz, $CDCl_3$) δ 148.22 (d, $^{1}J_{C–F}$ = 255.8 Hz), 131.38, 124.88, 109.55 (d, $^{2}J_{C–F}$ = 5.3 Hz), 36.65, 32.63 (d, $^{4}J_{C–F}$ = 1.9 Hz), 29.81 (d, $^{3}J_{C–F}$ = 4.1 Hz), 25.85, 25.73, 19.44, 17.75.

^{19}F NMR (377 MHz, $CDCl_3$) δ -129.21 (*E*-isomer), -130.72 (*Z*-isomer).

EI-MS (m/z, relative intensity): 170 (M^+, 10), 135 (10), 109 (30), 86 (50), 69 (100), 55 (30), 41 (30).

(*Z*)-4-Fluorobut-3-en-1-yl 4-(*N*,*N*-dipropylsulfamoyl)benzoate (S-8)

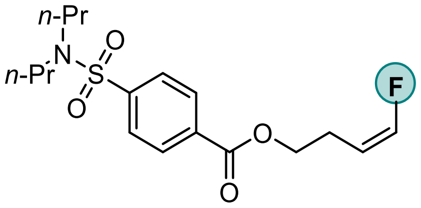

Z/E = 97:3

^{1}H NMR (400 MHz, $CDCl_3$) δ 8.15 (d, *J* = 8.3 Hz, 2H), 7.87 (d, *J* = 8.3 Hz, 2H), 6.56 (ddt, *J* = 84.7, 4.9, 1.7 Hz, 1H), 4.86 (dtd, *J* = 42.0, 7.6, 4.9 Hz, 1H), 4.37 (t, *J* = 6.6 Hz, 2H), 3.09 (br t, *J* = 7.5 Hz, 4H), 2.65 – 2.57 (m, 2H), 1.54 (td, *J* = 7.5, 7.4 Hz, 4H), 0.86 (t, *J* = 7.4 Hz, 6H).

^{13}C NMR (101 MHz, $CDCl_3$) δ 165.30, 149.72 (d, $^{1}J_{C–F}$ = 259.5 Hz), 144.44, 133.61, 130.34, 127.15, 106.32 (d, $^{2}J_{C–F}$ = 5.0 Hz), 64.40 (d, $^{4}J_{C–F}$ = 2.5 Hz), 50.07, 22.78 (d, $^{3}J_{C–F}$ = 5.5 Hz), 22.07, 11.28.

^{19}F NMR (377 MHz, $CDCl_3$) δ -126.06 (*E*-isomer), -127.81 (*Z*-isomer).

HRMS (ESI/QTOF) m/z: $[M + Na]^+$ Calcd for $C_{17}H_{24}FNNaO_4S^+$ 380.1302; Found 380.1310.

(*Z*)-4-fluorobut-3-en-1-yl (*R*)-4-((5*S*,8*R*,9*S*,10*S*,13*R*,14*S*,17*R*)-10,13-dimethyl-3,7,12-trioxohexadecahydro-1*H*-cyclopenta[a]phenanthren-17-yl)pentanoate (S-9)

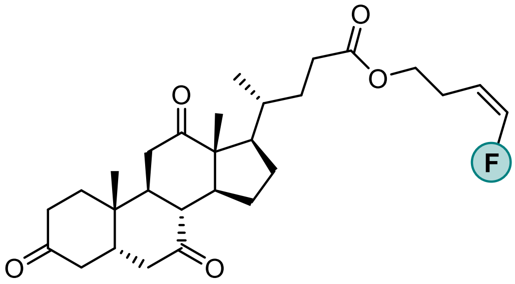

Z/E = 95:5

^{1}H NMR (400 MHz, $CDCl_3$) δ 6.50 (dd, *J* = 85.0, 4.8 Hz, 1H), 4.86 – 4.68 (m, 1H), 4.12 – 4.05 (m, 2H), 2.95 – 2.84 (m, 2H), 2.48 – 1.17 (m, 34H*), 1.39 (s, 3H), 1.06 (s, 3H), 0.84 (d, *J* =

6.7 Hz, 3H). *Please note: * = should be 24H. Further purification was not attempted as the present impurities did not seem to hinder the subsequent catalytic reaction.*

^{13}C NMR (101 MHz, $CDCl_3$) δ 212.05, 209.16, 208.82, 174.13, 149.33 (d, $^1J_{C–F}$ = 258.9 Hz), 106.61 (d, $^2J_{C–F}$ = 4.9 Hz), 63.13 (d, $^4J_{C–F}$ = 2.3 Hz), 57.02, 51.88, 49.12, 46.97, 45.78, 45.68, 45.11, 42.92, 38.76, 36.61, 36.14, 35.62, 35.41, 31.57, 30.55, 27.73, 25.26, 22.78 (d, $^3J_{C–F}$ = 5.4 Hz), 22.04, 18.75, 11.97.

^{19}F NMR (377 MHz, $CDCl_3$) δ -126.68 (*E*-isomer), -128.25 (*Z*-isomer).

HRMS (ESI/QTOF) m/z: [M + Na]$^+$ Calcd for $C_{28}H_{39}FNaO_5^+$ 497.2674; Found 497.2682.

3.2 Procedure for the Synthesis of Alkyl Bromides

Lactams **2**[4] and acyclic amides **4**[5] were prepared according to previous literature procedures on gram-scale.

3.3 General Procedure for the Synthesis of Bromofluoroamides

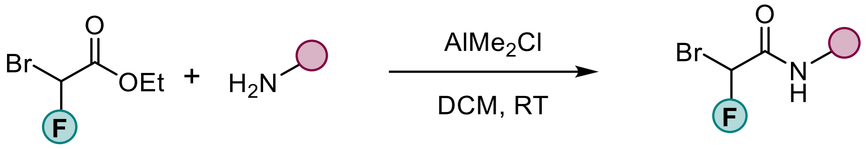

Bromofluoroamides **6** were prepared according to previous literature procedure on gram-scale.[6]

***N*-Benzyl-2-bromo-2-fluoroacetamide (6a)**

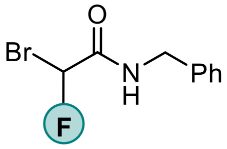

^{1}H NMR (400 MHz, $CDCl_3$) δ 7.41 – 7.28 (m, 5H), 6.64 (d, *J* = 50.9 Hz, 1H), 6.64 (br s, 1H), 4.60 – 4.46 (m, 2H).

^{13}C NMR (101 MHz, $CDCl_3$) δ 164.77 (d, $^2J_{C–F}$ = 20.9 Hz), 136.82, 129.06, 128.17, 127.97, 84.73 (d, $^1J_{C–F}$ = 266.8 Hz), 43.76.

^{19}F NMR (377 MHz, $CDCl_3$) δ -148.14.

HRMS (ESI/QTOF) m/z: [M + Na]$^+$ Calcd for $C_9H_9BrFNNaO^+$ 267.9744; Found 267.9749.

2-Bromo-2-fluoro-*N*-(4-methoxyphenyl)acetamide (6b)

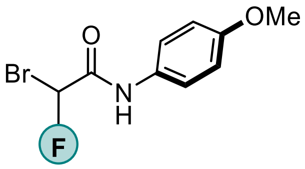

^{1}H NMR (400 MHz, $CDCl_3$) δ 7.88 (br s, 1H), 7.48 (d, *J* = 9.0 Hz, 2H), 6.90 (d, *J* = 9.0 Hz, 2H), 6.71 (d, *J* = 51.0 Hz, 1H), 3.81 (s, 3H).

^{13}C NMR (101 MHz, $CDCl_3$) δ 162.34 (d, $^{2}J_{C–F}$ =19.9 Hz), 157.52, 129.07, 122.17, 114.51, 84.97 (d, $^{1}J_{C–F}$ = 268.1 Hz), 55.64.

^{19}F NMR (377 MHz, $CDCl_3$) δ -146.53.

HRMS (ESI/QTOF) m/z: $[M + Na]^+$ Calcd for $C_9H_9BrFNNaO_2^+$ 283.9693; Found 283.9691.

2-Bromo-2-fluoro-*N*-(1-methyl-1*H*-indol-6-yl)acetamide (6c)

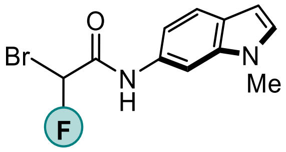

^{1}H NMR (400 MHz, $CDCl_3$) δ 7.95 (s, 1H), 7.92 – 7.89 (m, 1H), 7.33 – 7.27 (m, 2H), 7.08 (d, *J* = 3.1 Hz, 1H), 6.75 (d, *J* = 51.0 Hz, 1H), 6.48 (d, *J* = 3.1 Hz, 1H), 3.80 (s, 3H).

^{13}C NMR (101 MHz, $CDCl_3$) δ 162.34 (d, $^{2}J_{C–F}$ = 19.7 Hz), 134.91, 130.27, 128.67, 128.28, 115.64, 113.25, 109.72, 101.42, 85.27 (d, $^{1}J_{C–F}$ = 268.4 Hz), 33.14.

^{19}F NMR (377 MHz, $CDCl_3$) δ -146.08.

HRMS (ESI/QTOF) m/z: $[M + H]^+$ Calcd for $C_{11}H_{11}BrFN_2O^+$ 285.0033; Found 285.0040.

2-bromo-2-fluoro-*N*-(furan-2-ylmethyl)acetamide (6d)

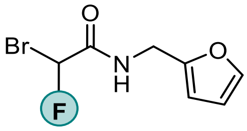

^{1}H NMR (400 MHz, $CDCl_3$) δ 7.39 (dd, *J* = 1.8, 0.8 Hz, 1H), 6.63 (d, *J* = 50.9 Hz, 1H), 6.62 (br s, 1H), 6.35 (dd, *J* = 3.3, 1.8 Hz, 1H), 6.30 (dd, *J* = 3.3, 0.8 Hz, 1H), 4.66 – 4.43 (m, 2H).

^{13}C NMR (101 MHz, $CDCl_3$) 164.61 (d, $^{2}J_{C–F}$ = 21.2 Hz), 149.72, 142.88, 110.73, 108.47, 84.58 (d, $^{1}J_{C–F}$ = 266.7 Hz), 36.76.

^{19}F NMR (377 MHz, $CDCl_3$) δ -148.56.

HRMS (nanochip-ESI/LTQ-Orbitrap) m/z: $[M + H]^+$ Calcd. for $C_7H_8BrFNO_2^+$ 235.9717; Found 235.9716.

2-Bromo-*N*-(*tert*-butyl)-2-fluoroacetamide (6e)

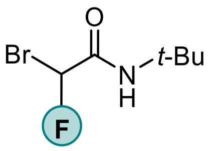

^{1}H NMR (400 MHz, $CDCl_3$) δ 6.48 (d, J = 51.3 Hz, 1H), 6.04 (br s, 1H), 1.41 (s, 9H).

^{13}C NMR (101 MHz, $CDCl_3$) δ 163.82 (d, $^2J_{C–F}$ = 19.2 Hz), 85.36 (d, $^1J_{C–F}$ = 269.1 Hz), 52.27, 28.57.

^{19}F NMR (377 MHz, $CDCl_3$) δ -144.95.

HRMS (ESI/QTOF) m/z: $[M + Na]^+$ Calcd for $C_6H_{11}BrFNNaO^+$ 233.9900; Found 233.9902.

2-Bromo-*N*-(*tert*-butyl)-2-fluoroacetamide (6f)

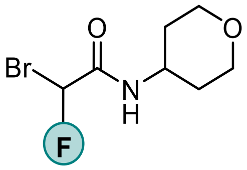

^{1}H NMR (400 MHz, $CDCl_3$) δ 6.58 (d, J = 51.0 Hz, 1H), 6.24 (bs, 1H), 4.01 (ddq, J = 22.6, 11.3, 3.8 Hz, 3H), 3.49 (tt, J = 11.7, 1.9 Hz, 2H), 1.94 (tdq, J = 15.1, 4.5, 2.3 Hz, 2H), 1.56 (dtt, J = 12.7, 11.4, 4.6 Hz, 2H).

^{13}C NMR (101 MHz, CDCl3) δ 164.09 (d, $^2J_{C–F}$ = 20.6 Hz), 84.59 (d, $^1J_{C–F}$ = 267.2 Hz), 66.53, 66.54, 46.29, 32.70, 32.44.

^{19}F NMR (376 MHz, $CDCl_3$) δ -147.87.

HRMS (ESI/QTOF) m/z: $[M + H]^+$ Calcd for $C_7H_{12}BrFNO_2^+$ 240.0030; Found 240.0033.

2-Bromo-2-fluoro-*N*-((*S*)-1-phenylethyl)acetamide (6g)

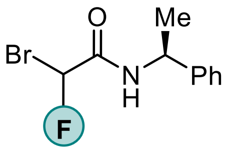

d.r. = 1:1

^{1}H NMR (400 MHz, $CDCl_3$) δ 7.45 – 7.27 (m, 5H), 6.59 (dd, J = 51.0, 12.6 Hz, 1H), 6.50 (bs, 1H), 5.16 (tt, J = 9.5, 6.9 Hz, 1H), 1.58 (dd, J = 6.9, 4.1 Hz, 3H).

^{13}C NMR (101 MHz, $CDCl_3$) (1:1 mixture of two diastereomer) δ 163.82 (d, $^2J_{C–F}$ = 20.3 Hz), 163.77 (d, $^2J_{C–F}$ = 20.7 Hz), 141.71, 141.64, 128.91, 127.94, 127.86, 126.22, 126.07, 84.75 (d, $^1J_{C–F}$ = 267.0 Hz), 84.70 (d, $^1J_{C–F}$ = 267.0 Hz), 49.36, 49.14, 21.49, 21.17.
^{19}F NMR (377 MHz, $CDCl_3$) δ –147.71 (s, 1F), –147.83 (s, 1F).
HRMS (nanochip-ESI/LTQ-Orbitrap) m/z: $[M + H]^+$ Calcd for $C_{10}H_{12}{}^{79}BrFNO^+$ 260.0081; Found 260.0081.

(*Z*)-1-Bromooct-1-ene (S-10)

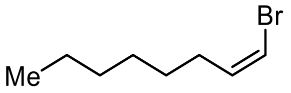

A reported literature procedure[7] was followed using 1-octyne (1.1 g, 10 mmol, 1.0 equiv.). Automated flash column chromatography (10 g SiO_2, pentane) afforded the mixture of the target compound (0.45 g, 23%) and dibrominated byproduct. To remove the latter, the isolated material was further purified by Kugelrohr distillation under high vacuum, at 55 °C. The most upper bulb contained pure **S-10** (80 mg, 4%, *Z/E* = 87:13).

^{1}H NMR (400 MHz, $CDCl_3$) δ 6.16 – 6.06 (m, 2H), 2.23 – 2.14 (m, 2H), 1.47 – 1.37 (m, 2H), 1.37 – 1.25 (m, 6H), 0.93 – 0.84 (m, 3H).
^{13}C NMR (101 MHz, $CDCl_3$) δ 135.24, 107.66, 31.78, 29.86, 28.97, 28.27, 22.74, 14.22.
HRMS (Sicrit plasma/LTQ-Orbitrap) m/z: $[M + H]^+$ Calcd for $C_8H_{16}Br^+$ 191.0430; Found 191.0429.

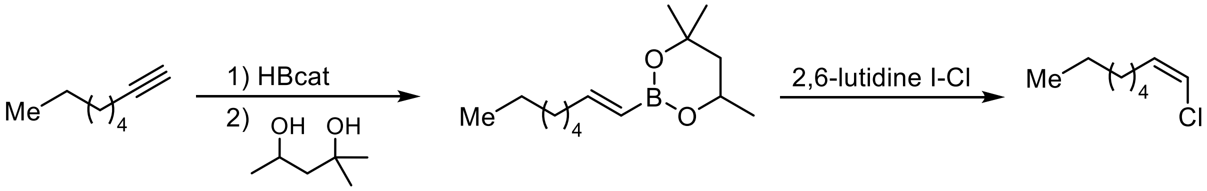

(*E*)-4,4,6-Trimethyl-2-(oct-1-en-1-yl)-1,3,2-dioxaborinane (S-11a)

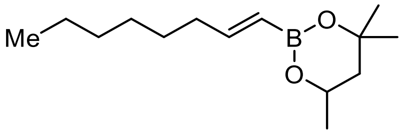

A reported literature procedure[8] was followed using 1-octyne (1.1 g, 10 mmol, 1.0 equiv.). Automated flash column chromatography (10 g SiO_2, gradient elution: hexane to 10% EtOAc in hexane) afforded the desired product **S-11a** as a slightly yellow oil (1.3 g, 55%).

^{1}H NMR (400 MHz, $CDCl_3$) δ 6.52 (dt, *J* = 17.7, 6.4 Hz, 1H), 5.33 (dt, *J* = 17.7, 1.6 Hz, 1H), 4.26 – 4.14 (m, 1H), 2.10 (dtd, *J* = 8.0, 6.5, 1.6 Hz, 2H), 1.76 (dd, *J* = 13.9, 2.9 Hz, 1H), 1.49

(dd, J = 13.9, 11.6 Hz, 1H), 1.43 – 1.35 (m, 2H), 1.31 – 1.23 (m, 9H), 1.29 (s, 6H), 0.94 – 0.82 (m, 3H).

^{13}C NMR (101 MHz, $CDCl_3$) δ 151.36, 70.67, 64.70, 46.13, 35.66, 31.91, 31.41, 29.14, 28.54, 28.25, 23.33, 22.75, 14.24.

HRMS (Sicrit plasma/LTQ-Orbitrap) m/z: $[M + H]^+$ Calcd for $C_{14}H_{28}BO_2^+$ 239.2177; Found 239.2177.

(*Z*)-1-Chlorooct-1-ene (S-11)

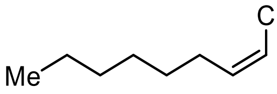

A reported literature procedure[8] was followed using (*E*)-4,4,6-trimethyl-2-(oct-1-en-1-yl)-1,3,2-dioxaborinane (**S-11a**) (0.41 g, 1.7 mmol, 1.0 equiv.) and 2,6-lutidine–iodine chloride complex[8] (0.93 g, 3.5 mmol, 2.0 mmol). Automated flash column chromatography (10 g SiO_2, pure pentane) afforded the desired product **S-11** as a colourless liquid (0.15 g, 58%).

^{1}H NMR (400 MHz, $CDCl_3$) δ ^{1}H NMR (400 MHz, $CDCl_3$) δ 6.00 (dt, J = 7.1, 1.6 Hz, 1H), 5.75 (dd, J = 7.2, 7.1 Hz, 1H), 2.22 (tdd, J = 7.2, 7.1, 1.6 Hz, 2H), 1.44 – 1.37 (m, 2H), 1.35 – 1.25 (m, 6H), 0.93 – 0.82 (m, 3H).

^{13}C NMR (101 MHz, $CDCl_3$) δ 132.09, 117.94, 31.77, 28.98, 28.50, 27.15, 22.74, 14.22.

EI-MS m/z (relative intensity): 148.1 (6), 146.1 (21, M^+), 104.0 (15), 84.1 (21), 81.1 (25), 77.1 (15), 75.0 (33), 69.1 (57), 67.1 (26), 56.1 (100), 55.1 (69), 53.1 (35), 51.0 (15).

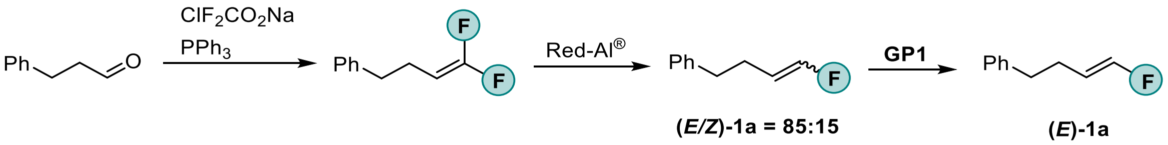

Wittig *gem*-difluorination of 3-phenylpropanal (2.7 g, 20 mmol, 1.0 equiv.) was performed according to a known literature procedure.[2] The isolated product was subsequently reduced[9] to yield **1a** as a *E/Z* mixture 85:15. To obtain pure **(*E*)-1a**, the former mixture (35 mg, 0.23 mmol, 2.3 equiv.) was engaged according to **GP1** together with 3-bromo-1-phenylpyrrolidin-2-one (**2a**) (24 mg, 0.10 mmol, 1.0 equiv.). Reaction time was 72 h. The reaction was performed in three replicates which were combined before isolation. Flash column chromatography (pure hexane) afforded the unreacted pure **(*E*)-1a** as a colourless oil (70 mg, 80%).

(*E*)-(4-fluorobut-3-en-1-yl)benzene ((*E*)-1a)

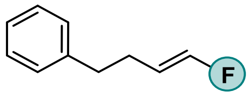

^{1}H NMR (400 MHz, $CDCl_3$) δ 7.32 – 7.27 (m, 2H), 7.23 – 7.15 (m, 3H), 6.49 (ddt, J = 85.6, 11.1, 1.5 Hz, 1H), 5.38 (ddt, J = 18.9, 11.1, 7.7 Hz, 1H), 2.73 – 2.64 (m, 2H), 2.28 – 2.18 (m, 2H).

^{13}C NMR (101 MHz, $CDCl_3$) δ 149.09 (d, $^{1}J_{C–F}$ = 254.1 Hz), 141.29, 128.60, 128.54, 126.19, 110.90 (d, $^{2}J_{C–F}$ = 9.6 Hz), 36.23 (d, $^{4}J_{C–F}$ = 2.8 Hz), 27.13 (d, $^{3}J_{C–F}$ = 9.4 Hz).

^{19}F NMR (377 MHz, $CDCl_3$) δ -129.85.

EI-MS m/z (relative intensity): 151.1 (4), 150.1 (39, M^+), 130.1 (10), 115.1 (6), 92.1 (26), 91.1 (100), 77.0 (9), 65.1 (39), 59.1 (14), 51.0 (17).

4. Reaction Optimization

Figure S1. Screening of Ligands

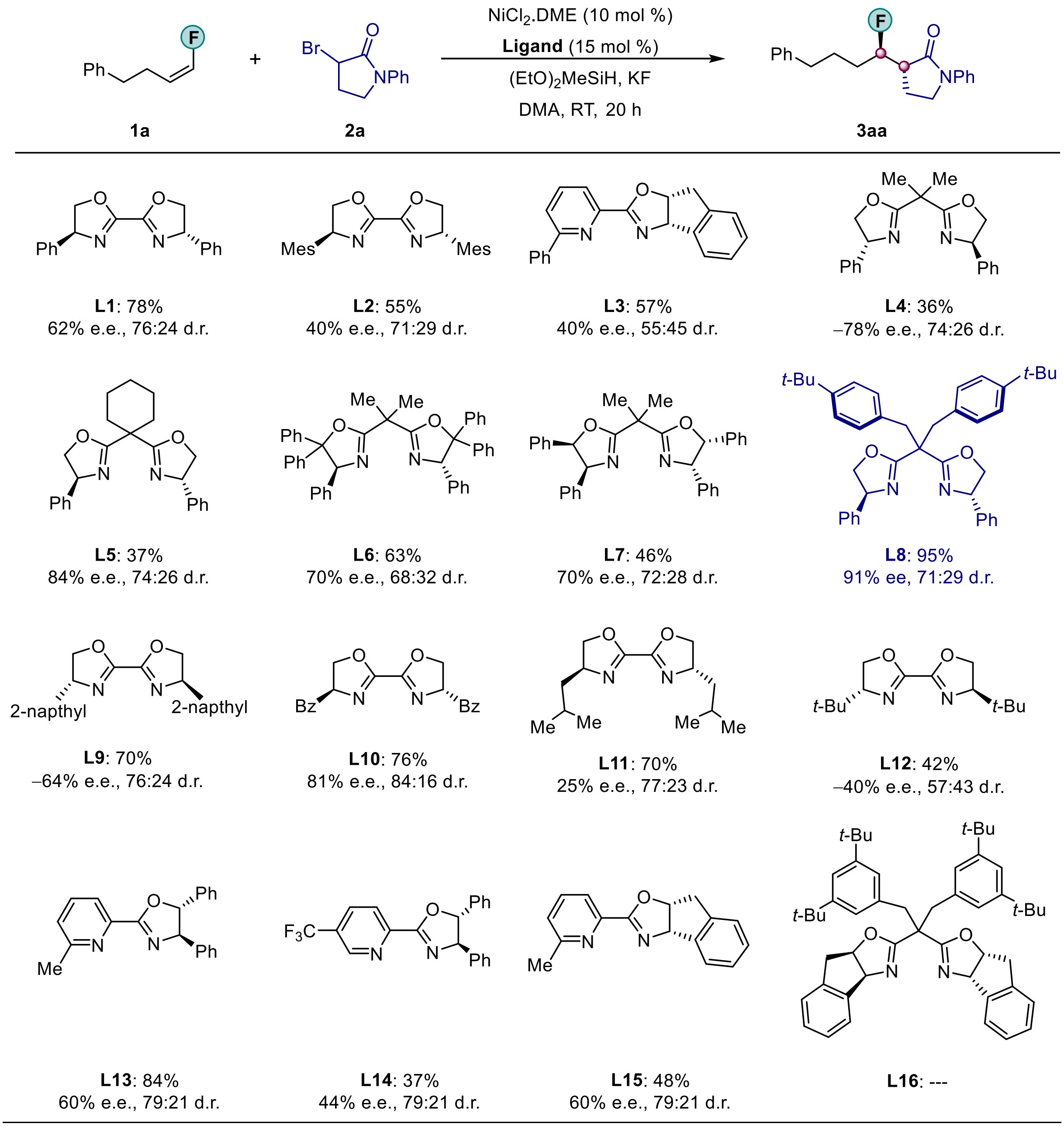

General reaction conditions: $NiCl_2$·DME (10 mol %), ligand (15 mol %), **1a** (0.20 mmol), **2a** (0.10 mmol), $(EtO)_2MeSiH$ (0.25 mmol), KF (0.25 mmol) and DMA (1.0 ml) at RT for 20 h; yields and d.r. were measured by ^{19}F-NMR of the crude reaction mixture. The e.e. was determined using chiral HPLC analysis of the product after purification. Mes = Mesityl, DME = Ethylene glycol dimethyl ether, Bn = Benzyl, DMA = *N*,*N*-Dimethylacetamide, RT = Room temperature.

Table S1. Screening of Different Reaction Conditions

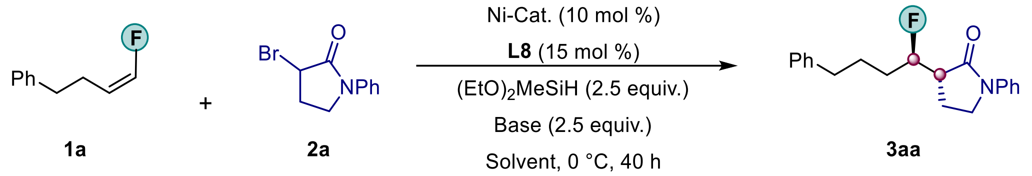

Entry	Ni-Catalyst	Base	Solvent	Yield	e.e.	d.r.
1[a]	$NiCl_2$·DME	KF	DMA	95	91	71:29
2	$NiCl_2$·DME	KF	DMA	80	96	85:15
3	$NiCl_2$·DME	KF	DMA/MeOH	nd	---	---
4	$NiCl_2$·DME	KF	DMA/*i*-PrOH	90	98	96:4
5	$NiCl_2$·DME	KF	DMA/*t*-BuOH	95	99	96:4
6	$NiCl_2$·DME	RbF	DMA/*t*-BuOH	85	99	97:3
7	$NiCl_2$·DME	K_2CO_3	DMA/*t*-BuOH	40	98	93:7
8	$NiCl_2$·DME	K_3PO_4	DMA/*t*-BuOH	86	98	96:4
10	$NiBr_2$·DME	KF	DMA/*t*-BuOH	93	97	96:4
11	$NiBr_2$·diglyme	KF	DMA/*t*-BuOH	94	98	96:4
12	NiI_2	KF	DMA/*t*-BuOH	90	98	96:4
13	$Ni(NO_3)_2$·$6H_2O$	KF	DMA/*t*-BuOH	88	99	96:4
**14[b]**	**$NiCl_2$·DME**	**KF**	**DMA/*t*-BuOH**	**95 (88)**	**99**	**96:4**
15[b]	---	KF	DMA/*t*-BuOH	nd	---	---
16[b]	$NiCl_2$·DME	---	DMA/*t*-BuOH	nd	---	---
17[b,c]	$NiCl_2$·DME	KF	DMA/*t*-BuOH	nd	---	---
18[b,d]	$NiCl_2$·DME	KF	DMA/*t*-BuOH	nd	---	---

General reaction conditions: Ni-catalyst (10 mol %), **L8** (15 mol %), **1a** (0.20 mmol), **2a** (0.10 mmol), $(EtO)_2MeSiH$ (0.25 mmol), base (0.25 mmol) and solvent (0.5/0.5 ml) at 0 °C for 40 h; yields and d.r. were measured by ^{19}F-NMR of the crude reaction mixture. The e.e. was determined using chiral HPLC analysis of the product after purification. [a]At RT, 20 h, [b]**1a**

(0.13 mmol), [c]Without **L8**, [d]Without $(EtO)_2MeSiH$. Isolated yield is given in parenthesis. nd = Not detected.

Table S2. Screening of Different Cobalt Conditions

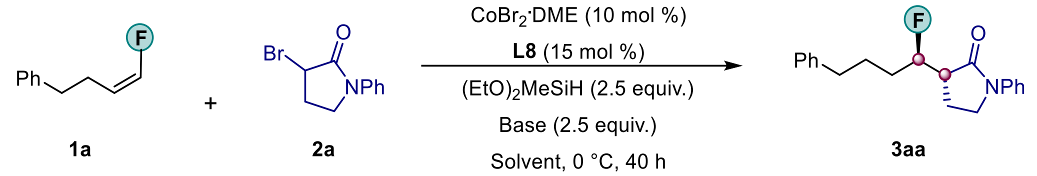

Entry	Base	Solvent	Yield	e.e.	d.r.
1	KF	DMA	nd	---	---
2	KF	DMA/*t*-BuOH	nd	---	---
3	KF	DME	nd	---	---
4	CsF	DME	nd	---	---

General reaction conditions: $CoBr_2$·DME (10 mol %), **L8** (15 mol %), **1a** (0.20 mmol), **2a** (0.10 mmol), $(EtO)_2MeSiH$ (0.25 mmol), base (0.25 mmol) and solvent (0.5/0.5 mL) or (1.0 mL) at 0 °C for 40 h; yields were measured by ^{19}F-NMR of the crude reaction mixture. nd = Not detected.

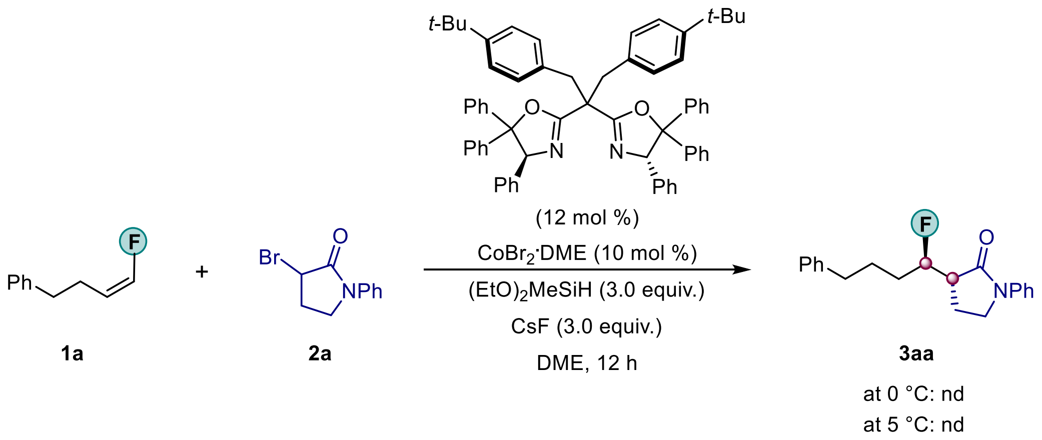

Table S3. Screening of Difluorinated Alkenes

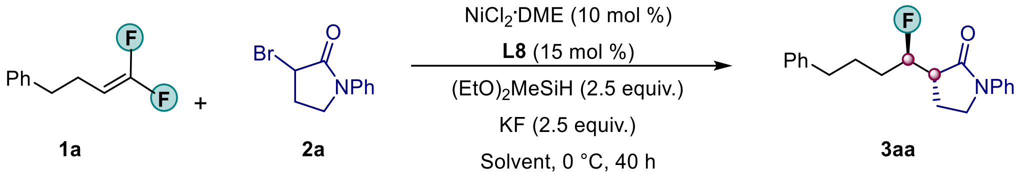

Entry	Solvent	Yield	e.e.	d.r.
1	DMA/*t*-BuOH	21%	80%	70:30
2	DMA	10%	79%	79:21

General reaction conditions: $NiCl_2$·DME (10 mol %), **L8** (15 mol %), **1a** (0.20 mmol), **2a** (0.10 mmol), $(EtO)_2MeSiH$ (0.25 mmol), base (0.25 mmol) and solvent (0.5/0.5 mL) or (1.0 mL) at 0 °C for 60 h; yields were measured by ^{19}F-NMR of the crude reaction mixture. nd = Not detected.

Figure S2. Unsuccessful Substrates

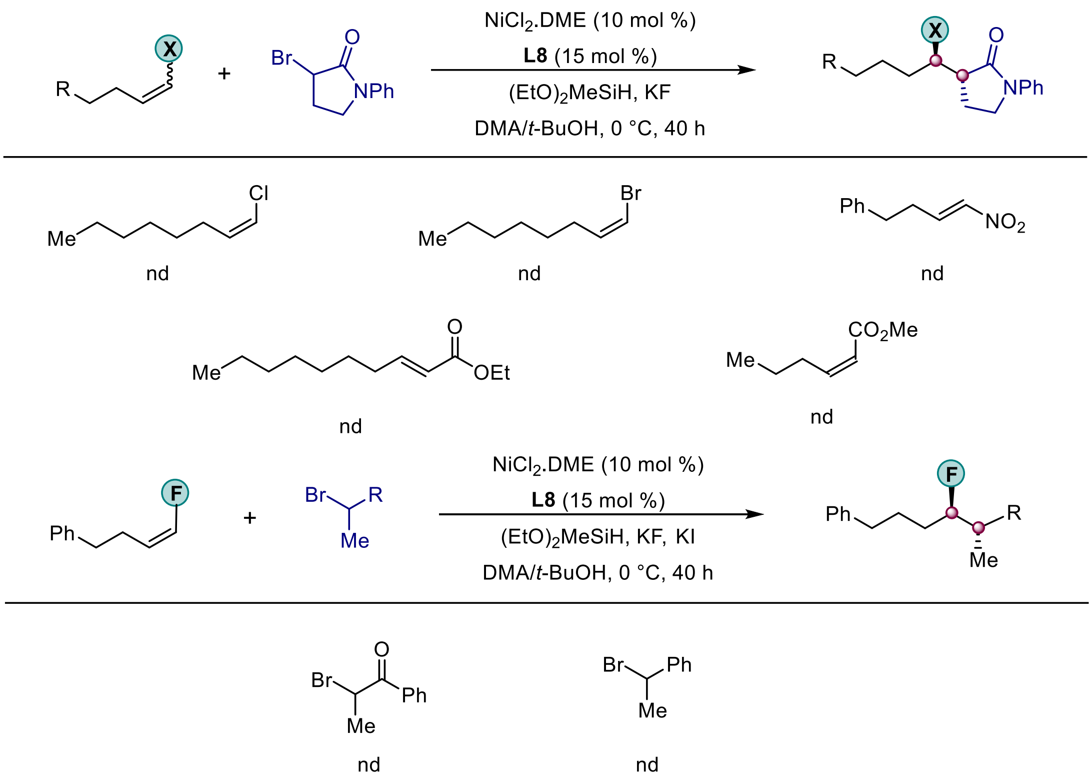

Reactions with compounds S-10 and S-11:

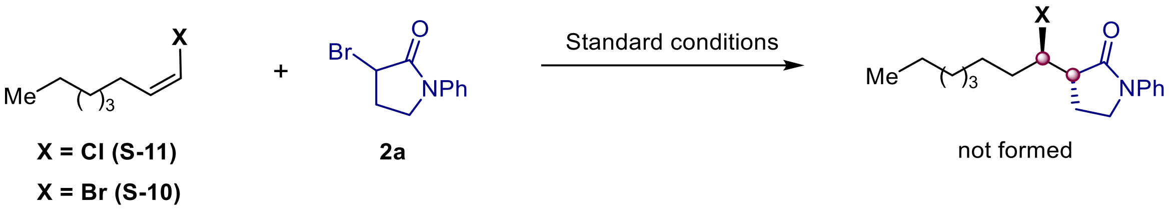

The reactions were conducted following **GP1**. Crude reaction mixtures were analyzed by GC-MS, which showed no formation of the desired product for either of the reactions. The figures below show products that most likely formed based on the observed mass.

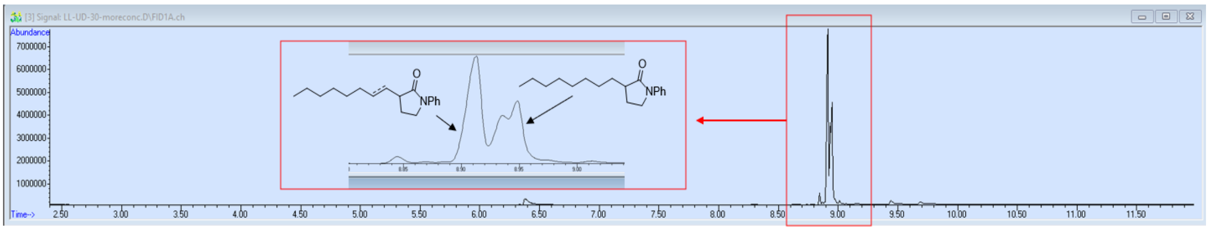

Figure S3: GC-MS analysis of the crude reaction mixture when (*Z*)-**S-11** was engaged.

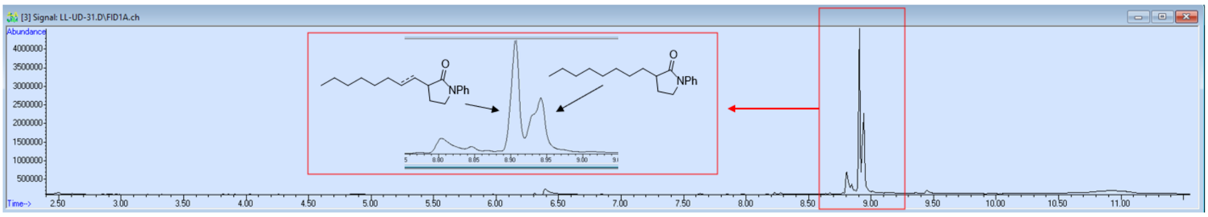

Figure S4: GC-MS analysis of the crude reaction mixture when (*Z*)-**S-10** was engaged.

The reaction performed with ***(Z)*-S-10** was purified by automated flash column chromatography (10 g SiO_2, gradient elution: hexane to 20% EtOAc in hexane). Two fractions were collected (7.5 mg and 10 mg, respectively). The first one predominately included one single product, while the second was a complex mixture. Characterization of the former one is given below.

(*Z*)-3-(Oct-1-en-1-yl)-1-phenylpyrrolidin-2-one (S-12)

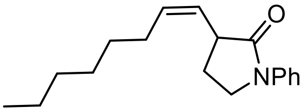

^{1}H NMR (400 MHz, $CDCl_3$) δ 7.67 – 7.61 (m, 2H), 7.41 – 7.32 (m, 2H), 7.16 – 7.08 (m, 1H), 5.70 (dtd, *J* = 10.4, 7.4, 1.2 Hz, 1H), 5.44 (ddt, *J* = 10.4, 8.7, 1.6 Hz, 1H), 3.90 – 3.73 (m, 2H), 3.59 (dddd, *J* = 9.9, 8.7, 8.7, 1.2 Hz, 1H), 2.40 (dddd, *J* = 12.3, 8.7, 6.8, 3.2 Hz, 1H), 2.24 – 2.08 (m, 2H), 2.00 – 1.88 (m, 1H), 1.48 – 1.19 (m, 8H), 0.92 – 0.86 (m, 3H). *Please note: unassigned peaks in the spectrum and unmatching integrations are due to the presence of a co-eluted reaction product (likely the fully saturated **S-12**).*

^{13}C NMR (101 MHz, $CDCl_3$) δ 174.67, 139.76, 134.81, 128.94, 126.34, 124.51, 119.77, 46.88, 42.74, 31.88, 29.74, 29.19, 28.09, 26.54, 22.78, 14.24. *Please note: unassigned peaks in the spectrum correspond to a co-eluted reaction product (likely the fully saturated **S-12**).*

HRMS (nanochip-ESI/LTQ-Orbitrap) m/z: $[M + H]^+$ Calcd. for $C_{18}H_{26}NO^+$ 272.2009; Found 272.2011.

5. Product Characterization Data

(*S*)-3-{(*R*)-1-Fluoro-4-phenylbutyl}-1-phenylpyrrolidin-2-one (3aa)

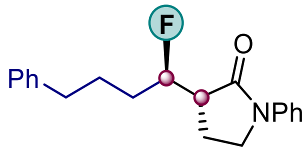

Prepared according to **GP1** with 3-bromo-1-phenylpyrrolidin-2-one (**2a**) (24.0 mg, 0.10 mmol, 1.0 equiv.) and (*Z*)-(4-fluorobut-3-en-1-yl)benzene (**1a**) (19.5 mg, 0.13 mmol, 1.3 equiv.). Automated flash column chromatography (10 g SiO_2, gradient elution: hexane to 10% EtOAc in hexane) afforded the desired product (+) **3aa** as a colourless oil (27.5 mg, 88%) in >99:1 diastereomeric ratio.

^{1}H NMR (400 MHz, $CDCl_3$) δ 7.69 – 7.51 (m, 2H), 7.43 – 7.33 (m, 2H), 7.31 – 7.24 (m, 2H), 7.22 – 7.09 (m, 4H), 4.89 (ddt, *J* = 47.8, 9.7, 3.3 Hz, 1H), 3.92 – 3.74 (m, 2H), 3.04 (dddd, *J* = 22.9, 9.4, 7.4, 3.5 Hz, 1H), 2.75 – 2.60 (m, 2H), 2.32 (dddd, *J* = 13.1, 9.5, 8.1, 5.1 Hz, 1H), 2.17 (ddt, *J* = 13.4, 8.4, 6.7 Hz, 1H), 2.04 (ddq, *J* = 19.2, 9.8, 4.8 Hz, 1H), 1.90 (dddd, *J* = 17.6, 8.6, 6.3, 4.8 Hz, 1H), 1.83 – 1.61 (m, 2H).
^{13}C NMR (101 MHz, $CDCl_3$) δ 171.90 (d, $^3J_{C–F}$ = 10.3 Hz), 142.03, 139.18, 128.87, 128.44, 128.36, 125.84, 124.82, 120.09, 94.59 (d, $^1J_{C–F}$ = 172.0 Hz), 47.20 (d, $^2J_{C–F}$ = 22.9 Hz), 47.20, 35.54, 30.97 (d, $^2J_{C–F}$ = 21.3 Hz), 27.57 (d, $^3J_{C–F}$ = 3.6 Hz), 20.79 (d, $^3J_{C–F}$ = 2.0 Hz).
^{19}F{1H} (376 MHz, $CDCl_3$) δ -189.70.

HRMS (APCI/QTOF) *m/z*: $[M + Na]^+$ Calcd for $C_{20}H_{22}FNNaO^+$ 334.1578; Found 334.1596.
$[\alpha]_D^{23}$ = +5.9 (c = 0.50 in $CHCl_3$).
HPLC: The enantiomeric excess (99%) was determined *via* HPLC analysis using a CHIRALCEL® IA column, with hexane:isopropanol = 95:5 at a flow rate 1.0 mL/min detected at 254 nm wavelength. Retention time: t_{major} = 16.4 min and t_{minor} = 23.9 min. Diastereomeric ratio (96:4) was determined by ^{19}F-NMR of the crude reaction mixture.

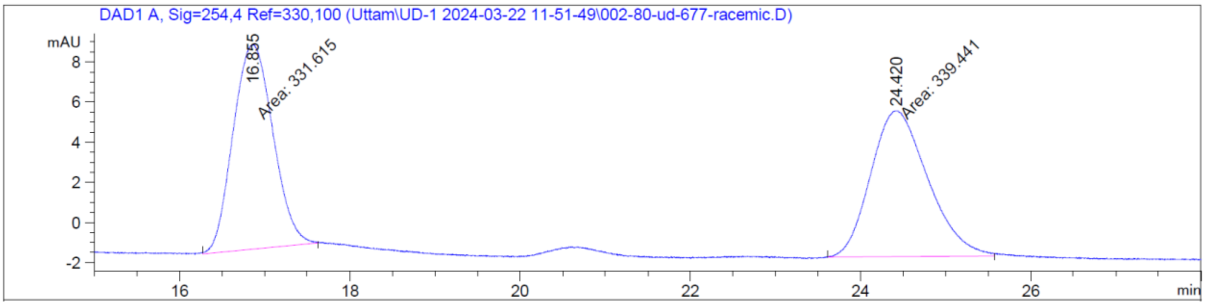

Peak #	RetTime [min]	Type	Width [min]	Area [mAU*s]	Height [mAU]	Area %
1	16.855	MM	0.5410	331.61472	10.21542	49.4169
2	24.420	MM	0.7781	339.44122	7.27105	50.5831

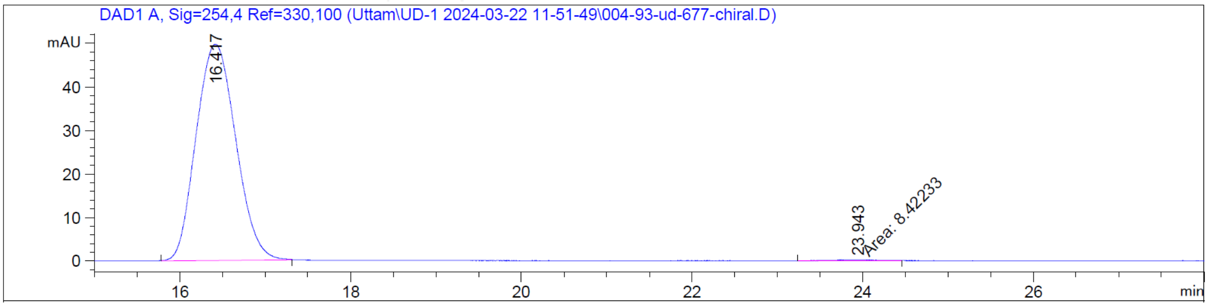

Peak #	RetTime [min]	Type	Width [min]	Area [mAU*s]	Height [mAU]	Area %
1	16.417	BB	0.3775	1598.48499	49.60429	99.4759
2	23.943	MM	0.6078	8.42233	2.30955e-1	0.5241

(*S*)-3-{(*R*)-6-Chloro-1-fluorohexyl}-1-phenylpyrrolidin-2-one (3ba)

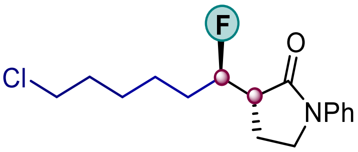

Prepared according to **GP1** with 3-bromo-1-phenylpyrrolidin-2-one (**2a**) (24.0 mg, 0.10 mmol, 1.0 equiv.) and (*Z*)-6-chloro-1-fluorohex-1-ene (**1b**) (17.8 mg, 0.13 mmol, 1.3 equiv.). Automated flash column chromatography (10 g SiO_2, gradient elution: hexane to 10% EtOAc in hexane) afforded the desired product (+) **3ba** as a colourless oil (23.0 mg, 77%) in >99:1 diastereomeric ratio.

^{1}H NMR (400 MHz, $CDCl_3$) δ 7.62 – 7.47 (m, 2H), 7.30 (t, *J* = 8.0 Hz, 2H), 7.09 (t, *J* = 7.4 Hz, 1H), 4.79 (ddt, *J* = 47.6, 10.1, 3.3 Hz, 1H), 3.77 (dtd, *J* = 27.4, 9.2, 5.8 Hz, 2H), 3.46 (t, *J* = 6.6 Hz, 2H), 2.97 (dddd, *J* = 23.1, 9.4, 7.4, 3.5 Hz, 1H), 2.37 – 2.19 (m, 1H), 2.19 – 2.05 (m, 1H), 1.95 (dqd, *J* = 13.6, 9.4, 5.3 Hz, 1H), 1.72 (p, *J* = 6.9 Hz, 2H), 1.59 – 1.38 (m, 5H).

^{13}C NMR (101 MHz, $CDCl_3$) δ 171.89 (d, $^{3}J_{C–F}$ = 10.3 Hz), 139.16, 128.87, 124.82, 120.06, 94.60 (d, $^{1}J_{C–F}$ = 171.6 Hz), 47.20, 47.15 (d, $^{2}J_{C–F}$ = 22.5 Hz), 44.95, 32.42, 31.17 (d, $^{2}J_{C–F}$ = 21.0 Hz), 26.59, 25.03 (d, $^{3}J_{C–F}$ = 4.0 Hz), 20.81 (d, $^{3}J_{C–F}$ = 2.0 Hz).

^{19}F{1H} NMR (376 MHz, $CDCl_3$) δ -190.04.

HRMS (ESI/QTOF) m/z: $[M + H]^+$ Calcd for $C_{16}H_{22}{}^{35}ClFNO+$ 298.1369; Found 298.1374.

$[\alpha]_D^{23}$ = +10.5 (c = 1.00 in $CHCl_3$).

HPLC: The enantiomeric excess (98%) was determined *via* HPLC analysis using a CHIRALCEL® IA column, with hexane:isopropanol = 80:20 at a flow rate 1.0 mL/min detected at 254 nm wavelength. Retention time: t_{major} = 8.3 min and t_{minor} = 11.5 min. Diastereomeric ratio (96:4) was determined by ^{19}F-NMR of the crude reaction mixture.

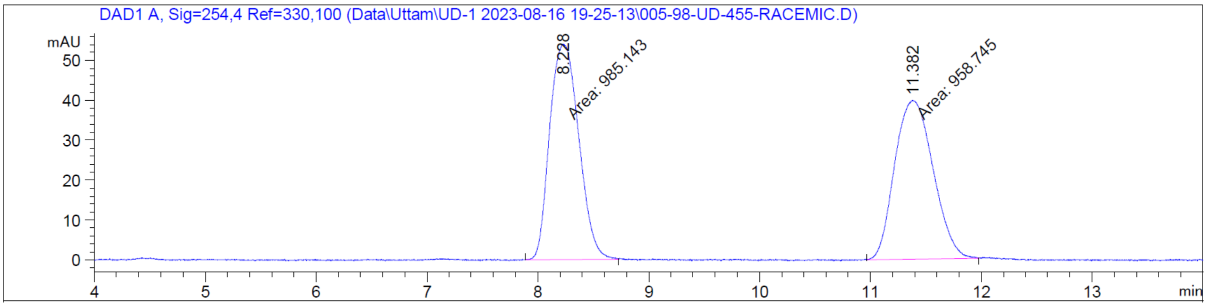

Peak #	RetTime [min]	Type	Width [min]	Area [mAU*s]	Height [mAU]	Area %
1	8.228	MM	0.3042	985.14337	53.98177	50.6790
2	11.382	MM	0.4013	958.74542	39.81898	49.3210

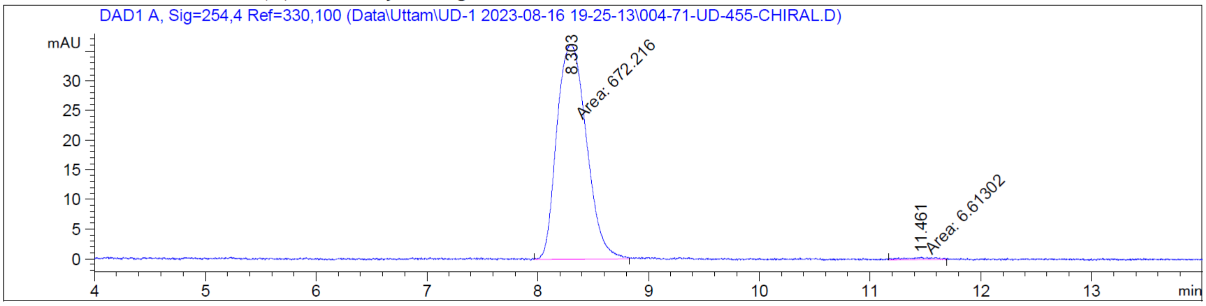

Peak #	RetTime [min]	Type	Width [min]	Area [mAU*s]	Height [mAU]	Area %
1	8.303	MM	0.3097	672.21637	36.18040	99.0258
2	11.461	MM	0.2781	6.61302	3.96285e-1	0.9742

_N_-[(_R_)-4-fluoro-4-{(_S_)-2-oxo-1-phenylpyrrolidin-3-yl}butyl]-2-phenylacetamide (3ca)

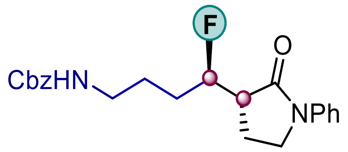

Prepared according to **GP1** with 3-bromo-1-phenylpyrrolidin-2-one (**2a**) (24.0 mg, 0.10 mmol, 1.0 equiv.) and (_Z_)-_N_-(4-fluorobut-3-en-1-yl)-2-phenylacetamide (**1c**) (26.9 mg, 0.13 mmol, 1.3 equiv.). Automated flash column chromatography (10 g SiO_2, gradient elution: hexane to 50% EtOAc in hexane) afforded the desired product (+) **3ca** as a yellow oil (30.0 mg, 78%) in 99:1 diastereomeric ratio.

^{1}H NMR (400 MHz, $CDCl_3$) δ 7.69 – 7.51 (m, 2H), 7.41 – 7.29 (m, 7H), 7.16 (t, _J_ = 7.4 Hz, 1H), 5.08 (s, 2H), 4.99 – 4.43 (m, 2H), 3.82 (dtd, _J_ = 24.0, 9.2, 5.4 Hz, 2H), 3.25 (h, _J_ = 6.4 Hz, 2H), 3.15 – 2.80 (m, 1H), 2.32 (dtd, _J_ = 13.5, 8.6, 4.9 Hz, 1H), 2.25 – 2.09 (m, 1H), 2.03 (dq, _J_ = 11.0, 6.9 Hz, 1H), 1.81 – 1.59 (m, 3H).

^{13}C NMR (101 MHz, $CDCl_3$) δ 171.82 (d, $^{3}J_{C–F}$ = 9.5 Hz), 156.47, 139.12, 136.64, 128.88, 128.52, 128.10, 128.08, 124.87, 120.11, 94.31 (d, $^{1}J_{C–F}$ = 172.0 Hz), 66.62, 47.21, 47.09 (d, $^{2}J_{C–F}$ = 20.9 Hz), 40.56, 28.56 (d, $^{2}J_{C–F}$ = 21.3 Hz), 26.23 (d, $^{3}J_{C–F}$ = 3.5 Hz), 20.89 (d, $^{3}J_{C–F}$ = 1.5 Hz).

$^{19}F\{1H\}$ NMR (376 MHz, $CDCl_3$) δ -190.00.

HRMS (ESI/QTOF) m/z: $[M + Na]^+$ Calcd for $C_{22}H_{25}FN_2NaO_3^+$ 407.1741; Found 407.1757.

$[\alpha]_D^{23}$ = +8.7 (c = 1.00 in $CHCl_3$).

HPLC: The enantiomeric excess (94%) was determined _via_ HPLC analysis using a CHIRALCEL® IA column, with hexane:isopropanol = 80:20 at a flow rate 1.0 mL/min detected at 254 nm wavelength. Retention time: t_{major} = 20.2 min and t_{minor} = 18.1 min. Diastereomeric ratio (94:6) was determined by ^{19}F-NMR of the crude reaction mixture.

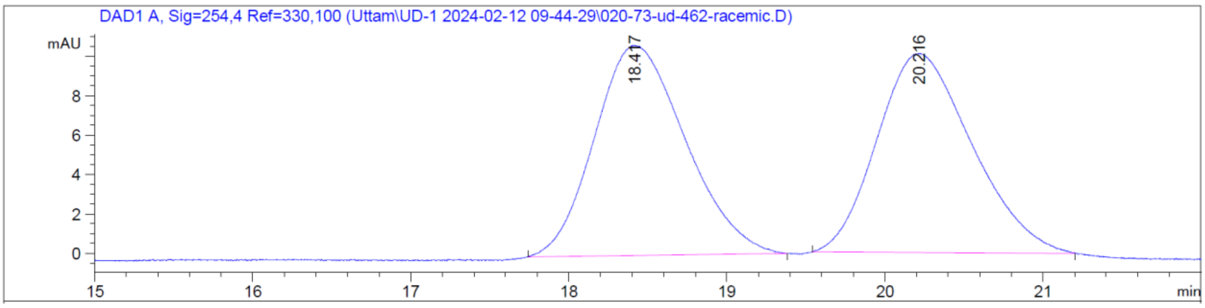

Peak #	RetTime [min]	Type	Width [min]	Area [mAU*s]	Height [mAU]	Area %
1	18.417	BB	0.4683	426.81046	10.67867	50.3248
2	20.216	BB	0.4875	421.30081	10.11380	49.6752

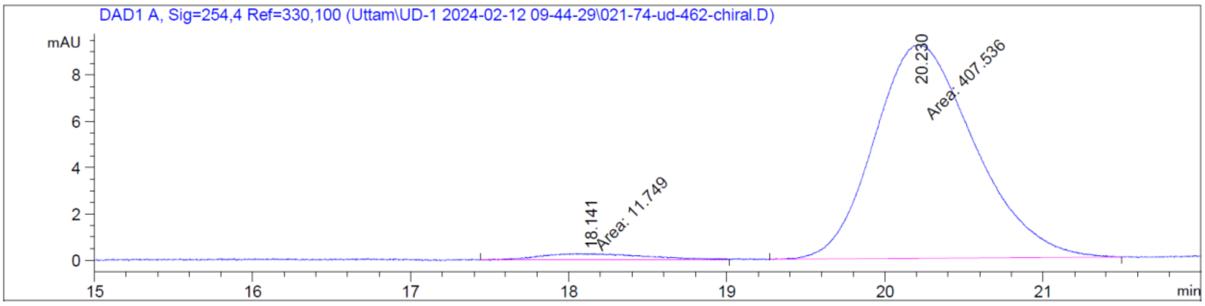

Peak #	RetTime [min]	Type	Width [min]	Area [mAU*s]	Height [mAU]	Area %
1	18.141	MM	0.7188	11.74899	2.72415e-1	2.8021
2	20.230	MM	0.7346	407.53604	9.24589	97.1979

(*S*)-3-[(*R*)-4-{(*tert*-Butyldimethylsilyl)oxy}-1-fluorobutyl]-1-(4-methoxyphenyl)-pyrrolidin-2-one (3db)

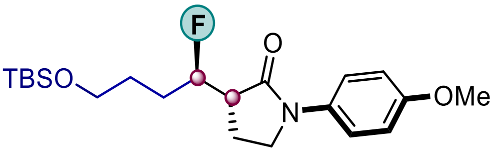

Prepared according to **GP1** with 3-bromo-1-(4-methoxyphenyl)pyrrolidin-2-one (**2b**) (27 mg, 0.10 mmol, 1.0 equiv.) and (*Z*)-*tert*-butyl((4-fluorobut-3-en-1-yl)oxy)dimethylsilane (**1a**) (31 mg, 0.15 mmol, 1.5 equiv.). Automated flash column chromatography (10 g SiO_2, gradient elution: hexane to 10% EtOAc in hexane) afforded the desired product (+) **3db** as a white solid (24.5 mg, 62%) >99:1 diastereomeric ratio.

^{1}H NMR (400 MHz, $CDCl_3$) δ 7.51 – 7.46 (m, 2H), 6.92 – 6.85 (m, 2H), 4.88 (ddt, *J* = 47.5, 9.6, 3.3 Hz, 1H), 3.80 (s, 3H), 3.88 – 3.73 (m, 2H), 3.71 – 3.59 (m, 2H), 3.03 (dddd, *J* = 23.0, 9.6, 7.1, 3.3 Hz, 1H), 2.32 (dddd, *J* = 13.3, 9.5, 8.2, 5.3 Hz, 1H), 2.18 (ddt, *J* = 13.3, 8.2, 6.5 Hz, 1H), 2.10 – 1.93 (m, 1H), 1.89 – 1.59 (m, 3H), 0.88 (s, 9H), 0.04 (s, 6H).

^{13}C NMR (101 MHz, $CDCl_3$) δ 171.73 (d, $^3J_{C–F}$ = 10.0 Hz), 156.88, 132.55, 122.07, 114.18, 94.91 (d, $^1J_{C–F}$ = 171.7 Hz), 62.83, 55.63, 47.77, 47.07 (d, $^2J_{C–F}$ = 22.8 Hz), 29.09 (d, $^3J_{C–F}$ = 3.6 Hz), 28.07 (d, $^2J_{C–F}$ = 21.3 Hz), 26.09, 20.95 (d, $^3J_{C–F}$ = 1.8 Hz), 18.46, -5.16.

^{19}F{1H} NMR (377 MHz, $CDCl_3$) δ -189.42.

HRMS (ESI/QTOF) m/z: [M + Na]$^+$ Calcd. for $C_{21}H_{34}FNNaO_3Si^+$ 418.2184; Found 418.2187.

$[\alpha]_D^{25}$ = +7.8 (c = 0.50 in $CHCl_3$).

HPLC: The enantiomeric excess (98%) was determined *via* HPLC analysis using a CHIRALCEL® IA column, with hexane:isopropanol = 95:5 at a flow rate 1.0 mL/min detected at 254 nm wavelength. Retention time: t_{major} = 16.1 min and t_{minor} = 19.9 min. Diastereomeric ratio (92:8) was determined by ^{19}F-NMR analysis of the crude reaction mixture.

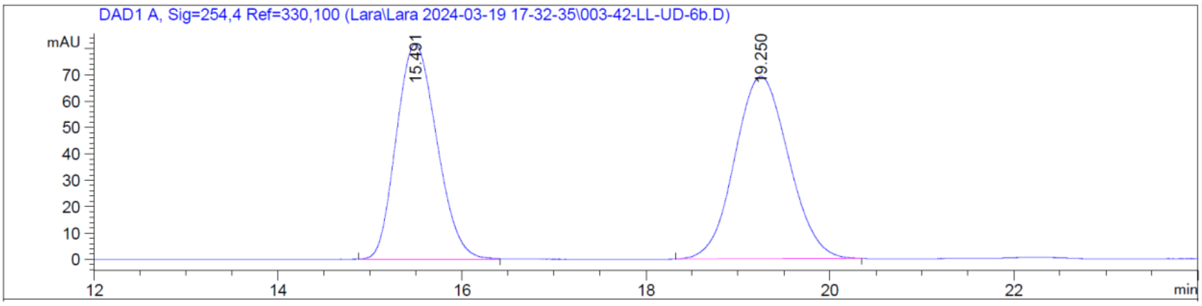

Peak #	RetTime [min]	Type	Width [min]	Area [mAU*s]	Height [mAU]	Area %
1	15.491	BB	0.3595	2483.56250	81.49372	46.8849
2	19.250	BB	0.4762	2813.59082	69.22398	53.1151

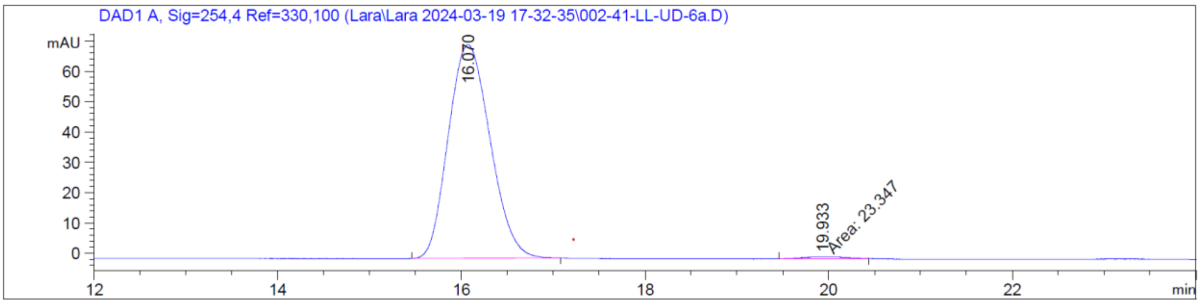

Peak #	RetTime [min]	Type	Width [min]	Area [mAU*s]	Height [mAU]	Area %
1	16.070	BB	0.3777	2222.72559	70.32481	98.9605
2	19.933	MM	0.5477	23.34699	7.10470e-1	1.0395

2-[(*R*)-4-fluoro-4-{(*S*)-2-oxo-1-phenylpyrrolidin-3-yl}butyl]isoindoline-1,3-dione (3ea)

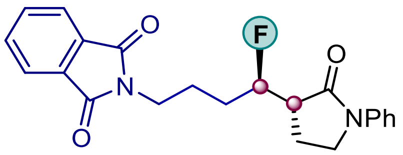

Prepared according to **GP1** with 3-bromo-1-phenylpyrrolidin-2-one (**2a**) (24.0 mg, 0.10 mmol, 1.0 equiv.) and (*Z*)-2-(4-fluorobut-3-en-1-yl)isoindoline-1,3-dione (**1e**) (28.5 mg, 0.13 mmol, 1.3 equiv.). Automated flash column chromatography (10 g SiO_2, gradient elution: hexane to 40% EtOAc in hexane) afforded the desired product (+) **3ea** as a yellow oil (32.5 mg, 86%) in 91:9 diastereomeric ratio.

^{1}H NMR (400 MHz, $CDCl_3$) δ 7.83 (dd, *J* = 5.5, 3.1 Hz, 2H), 7.70 (dd, *J* = 5.5, 3.0 Hz, 2H), 7.60 – 7.55 (m, 2H), 7.37 – 7.32 (m, 2H), 7.17 – 7.11 (m, 1H), 4.90 (ddt, *J* = 47.9, 10.0, 3.3 Hz, 1H), 3.89 – 3.71 (m, 4H), 3.02 (dddd, *J* = 23.4, 9.4, 7.4, 3.5 Hz, 1H), 2.33 (dddd, *J* = 13.1, 9.5, 8.2, 5.0 Hz, 1H), 2.26 – 2.06 (m, 2H), 1.95 (dddt, *J* = 12.2, 7.4, 4.8, 2.4 Hz, 1H), 1.88 – 1.66 (m, 2H).

^{13}C NMR (101 MHz, $CDCl_3$): δ 171.71 (d, $^{3}J_{C–F}$ = 9.5 Hz), 168.40, 139.13, 133.93, 132.12, 128.84, 124.82, 123.24, 120.12, 94.02 (d, $^{1}J_{C–F}$ = 172.4 Hz), 47.19, 47.12 (d, $^{2}J_{C–F}$ = 22.7 Hz), 37.48, 28.76 (d, $^{2}J_{C–F}$ = 21.3 Hz), 25.00 (d, $^{3}J_{C–F}$ = 3.7 Hz), 20.88 (d, $^{3}J_{C–F}$ = 1.8 Hz).

^{19}F{1H} NMR (376 MHz, $CDCl_3$) δ -190.44 (major diastereomer), -191.98 (minor diastereomer).

HRMS (APCI/QTOF) m/z: $[M + Na]^+$ Calcd for $C_{22}H_{21}FN_2NaO_3^+$ 403.1428; Found 403.1452.

$[\alpha]_D^{23}$ = +14.9 (c = 1.00 in $CHCl_3$).

HPLC: The enantiomeric excess (95%) was determined *via* HPLC analysis using a CHIRALCEL® IA column, with hexane:isopropanol = 75:25 at a flow rate 1.0 mL/min detected at 214 nm wavelength. Retention time: t_{major} = 33.7 min and t_{minor} = 25.1 min. Diastereomeric ratio (91:9) was determined by ^{19}F-NMR of the crude reaction mixture.

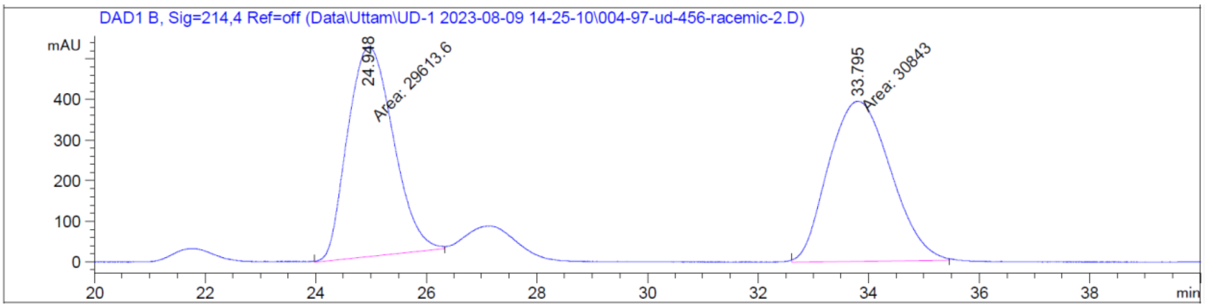

Peak #	RetTime [min]	Type	Width [min]	Area [mAU*s]	Height [mAU]	Area %
1	24.948	MM	0.9558	2.96136e4	516.36346	48.9832
2	33.795	MM	1.3044	3.08430e4	394.08432	51.0168

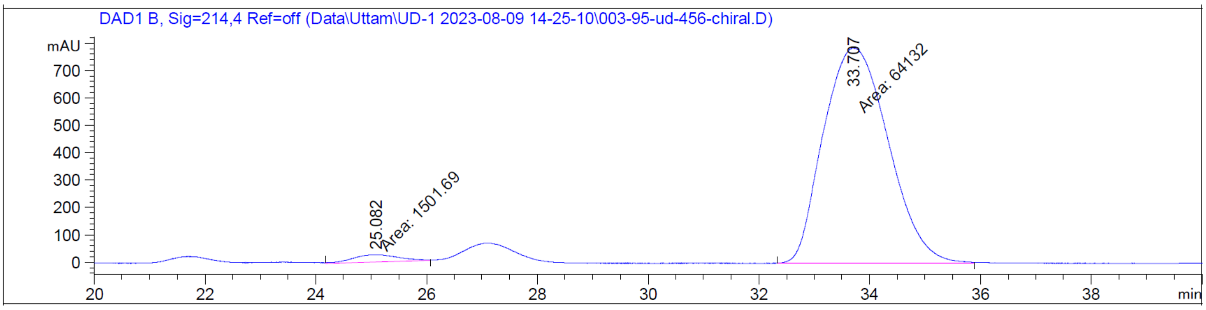

Peak #	RetTime [min]	Type	Width [min]	Area [mAU*s]	Height [mAU]	Area %
1	25.082	MM	0.9370	1501.69397	26.71095	2.2880
2	33.707	MM	1.3563	6.41320e4	788.06232	97.7120

(*S*)-3-{(*R*)-1-Fluoro-4-(5-methylfuran-2-yl)butyl}-1-(4-methoxyphenyl)pyrrolidin-2-one (3fb)

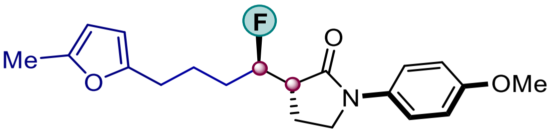

Prepared according to **GP1** with 3-bromo-1-(4-methoxyphenyl)pyrrolidin-2-one (**2b**) (27 mg, 0.10 mmol, 1.0 equiv.) and (*Z*)-2-(4-fluorobut-3-en-1-yl)-5-methylfuran (**1f**) (23 mg, 0.15 mmol, 1.5 equiv.). Automated flash column chromatography (10 g SiO_2, gradient elution: hexane to 15% EtOAc in hexane) afforded the desired product **3fb** as a white solid (31.5 mg, 92%) in 99:1 diastereomeric ratio.

^{1}H NMR (400 MHz, $CDCl_3$) δ 7.52 – 7.45 (m, 2H), 6.93 – 6.86 (m, 2H), 5.87 – 5.81 (m, 2H), 4.87 (ddt, *J* = 47.7, 9.7, 3.2 Hz, 1H), 3.86 – 3.72 (m, 2H), 3.80 (s, 3H), 3.02 (dddd, *J* = 23.0, 9.5, 7.2, 3.2 Hz, 1H), 2.63 (t, *J* = 7.4 Hz, 2H), 2.31 (dddd, *J* = 13.2, 9.5, 8.2, 5.2 Hz, 1H), 2.24 (s, 3H), 2.21 – 2.12 (m, 1H), 2.04 (dtd, *J* = 19.3, 9.3, 4.8 Hz, 1H), 1.94 – 1.82 (m, 1H), 1.81 – 1.61 (m, 2H).

^{13}C NMR (101 MHz, $CDCl_3$) δ 171.67 (d, $^{3}J_{C\text{–}F}$ = 10.1 Hz), 156.90, 153.95, 150.41, 132.52, 122.07, 114.19, 105.93, 105.74, 94.72 (d, $^{1}J_{C\text{–}F}$ = 171.8 Hz), 55.62, 47.74, 47.06 (d, $^{2}J_{C\text{–}F}$ = 22.7 Hz), 30.95 (d, $^{2}J_{C\text{–}F}$ = 21.1 Hz), 27.82, 24.53 (d, $^{3}J_{C\text{–}F}$ = 3.7 Hz), 20.96 (d, $^{3}J_{C\text{–}F}$ = 1.8 Hz), 13.65.

$^{19}F\{1H\}$ NMR (377 MHz, $CDCl_3$) δ -189.87.

HRMS (ESI/QTOF) m/z: $[M + Na]^+$ Calcd for $C_{20}H_{24}FNNaO_3^+$ 368.1632; Found 368.1630.

$[\alpha]_D^{25}$ = +17.8 (c = 0.50 in $CHCl_3$).

HPLC: The enantiomeric excess (99.5%) was determined *via* HPLC analysis using a CHIRALCEL® IA column, with hexane:isopropanol = 90:10 at a flow rate 1.0 mL/min detected at 254 nm wavelength. Retention time: t_{major} = 19.5 min and t_{minor} = 25.7 min. Diastereomeric ratio (96:4) was determined by ^{19}F-NMR analysis of the crude reaction mixture.

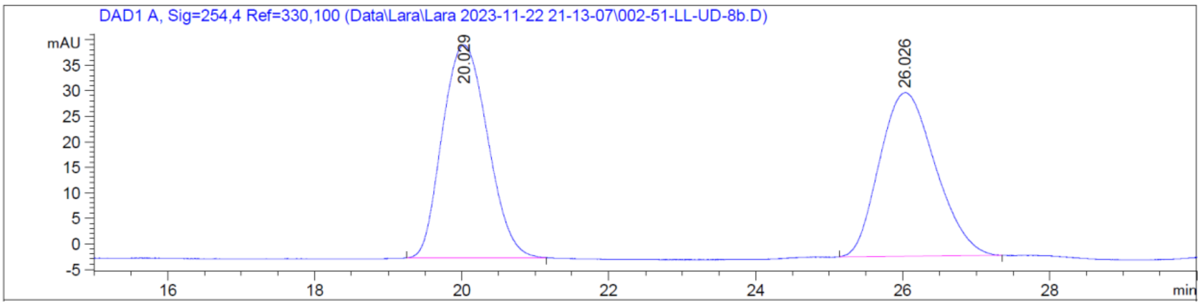

Peak #	RetTime [min]	Type	Width [min]	Area [mAU*s]	Height [mAU]	Area %
1	20.029	BB	0.5035	1787.15967	41.78131	51.3290
2	26.026	BB	0.6188	1694.61560	32.05423	48.6710

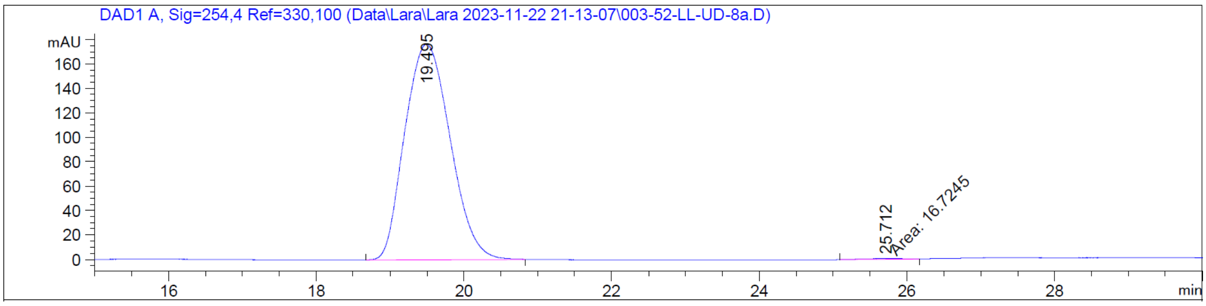

Peak #	RetTime [min]	Type	Width [min]	Area [mAU*s]	Height [mAU]	Area %
1	19.495	BB	0.5114	7530.71045	176.45419	99.7784
2	25.712	MM	0.6200	16.72448	4.49613e-1	0.2216

(*S*)-3-{(1*R*,5*S*)-1-fluoro-5,8-dimethylnon-7-en-1-yl}-1-(4-methoxyphenyl)pyrrolidin-2-one (3gb)

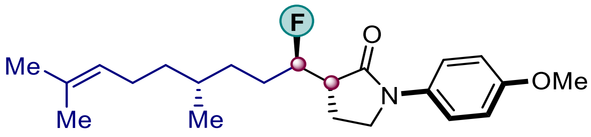

Prepared according to **GP1** with 3-bromo-1-(4-methoxyphenyl)pyrrolidin-2-one (**2b**) (27 mg, 0.10 mmol, 1.0 equiv.) and (*S*,*Z*)-1-fluoro-4,8-dimethylnona-1,7-diene (**1g**) (26 mg, 0.15 mmol, 1.5 equiv.). Automated flash column chromatography (10 g SiO_2, gradient elution: hexane to 10% EtOAc in hexane) afforded the desired product **3gb** as a white solid (30 mg, 83%) in 99:1 diastereomeric ratio.

^{1}H NMR (400 MHz, $CDCl_3$) δ 7.52 – 7.47 (m, 2H), 6.92 – 6.86 (m, 2H), 5.12 – 5.05 (m, 1H), 4.83 (ddt, *J* = 47.6, 9.6, 3.4 Hz, 1H), 3.80 (s, 3H), 3.87 – 3.72 (m, 2H), 3.03 (dddd, *J* = 22.8, 9.5, 7.3, 3.4 Hz, 1H), 2.32 (dddd, *J* = 13.1, 9.4, 8.2, 5.1 Hz, 1H), 2.26 – 2.11 (m, 1H), 2.07 – 1.88 (m, 3H), 1.73– 1.60 (m, 1H), 1.67 (br s, 3H), 1.59 (br s, 3H), 1.51 – 1.27 (m, 4H), 1.16 (dddd, *J* = 13.5, 9.4, 7.5, 6.0 Hz, 1H), 0.89 (d, *J* = 6.4 Hz, 3H).

^{13}C NMR (101 MHz, $CDCl_3$) δ 171.78 (d, $^{3}J_{C–F}$ = 10.4 Hz), 156.87, 132.57, 131.30, 124.96, 122.03, 114.19, 95.23 (d, $^{1}J_{C–F}$ = 171.5 Hz), 55.63, 47.73, 47.11 (d, $^{2}J_{C–F}$ = 22.9 Hz), 37.18, 32.90 (d, $^{3}J_{C–F}$ = 3.5 Hz), 32.31, 28.96 (d, $^{2}J_{C–F}$ = 21.1 Hz), 25.86, 25.67, 20.99 (d, $^{3}J_{C–F}$ = 1.7 Hz), 19.52, 17.78.

^{19}F{1H} NMR (377 MHz, $CDCl_3$) δ -189.52.

HRMS (ESI/QTOF) m/z: $[M + Na]^+$ Calcd. for $C_{22}H_{32}FNNaO_2^+$ 384.2309; Found 384.2315.

$[\alpha]_D^{25}$ = +8.4 (c = 0.50 in $CHCl_3$).

HPLC: The enantiomeric excess (99%) was determined *via* HPLC analysis using a CHIRALCEL® IA column, with hexane:isopropanol = 90:10 at a flow rate 1.0 mL/min detected at 254 nm wavelength. Retention time: t_{major} = 11.9 min and t_{minor} = 14.9 min. Diastereomeric ratio (99:1) was determined by ^{19}F-NMR analysis of the crude reaction mixture.

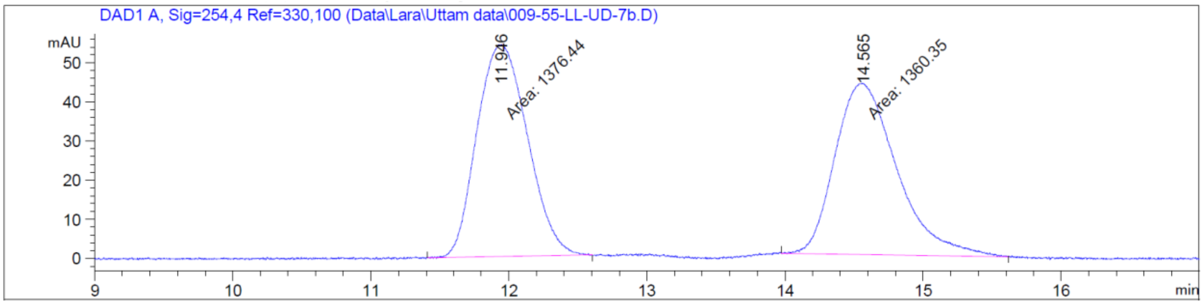

Peak #	RetTime [min]	Type	Width [min]	Area [mAU*s]	Height [mAU]	Area %
1	11.946	MM	0.4241	1376.44250	54.09638	50.2941
2	14.565	MM	0.5189	1360.34631	43.69277	49.7059

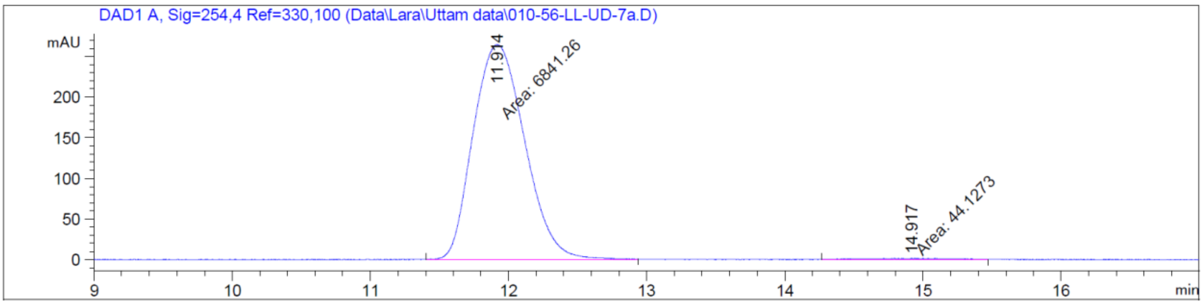

Peak #	RetTime [min]	Type	Width [min]	Area [mAU*s]	Height [mAU]	Area %
1	11.914	MM	0.4310	6841.25635	264.57019	99.3591
2	14.917	MM	0.6369	44.12729	1.15469	0.6409

(*S*)-1-(4-Bromophenyl)-3-((*R*)-1-fluoro-4-phenylbutyl)pyrrolidin-2-one (3ac)

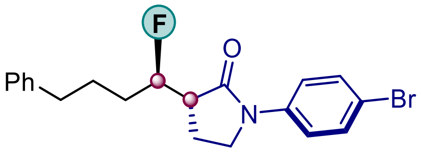

Prepared according to **GP1** with 3-bromo-1-(4-bromophenyl)pyrrolidin-2-one (**2c**) (32 mg, 0.10 mmol, 1.0 equiv.) and (*Z*)-(4-fluorobut-3-en-1-yl)benzene (**1a**) (19.5 mg, 0.13 mmol, 1.3 equiv.). Automated flash column chromatography (10 g SiO_2, gradient elution: hexane to 10% EtOAc in hexane) afforded the desired product (+) **3ac** as light brown crystals (31.0 mg, 80%) in 92:8 diastereomeric ratio.

^{1}H NMR (400 MHz, $CDCl_3$) δ 7.54 – 7.45 (m, 4H), 7.30 – 7.24 (m, 2H), 7.21 – 7.15 (m, 3H), 4.86 (ddt, *J* = 47.8, 9.7, 3.3 Hz, 1H), 3.90 – 3.68 (m, 2H), 3.01 (dddd, *J* = 23.7, 9.4, 7.4, 3.3 Hz, 1H), 2.68 (dd, *J* = 8.6, 6.9 Hz, 2H), 2.32 (dddd, *J* = 13.2, 9.5, 8.2, 5.1 Hz, 1H), 2.26 – 2.12 (m, 1H), 2.11 – 1.98 (m, 1H), 1.95 – 1.82 (m, 1H), 1.81 – 1.59 (m, 2H).

^{13}C NMR (101 MHz, $CDCl_3$) δ 172.10 (d, $^{3}J_{C–F}$ = 9.6 Hz), 142.08, 138.36, 131.94, 128.55, 128.50, 125.99, 121.53, 117.72, 94.66 (d, $^{1}J_{C–F}$ = 172.3 Hz), 47.22 (d, $^{2}J_{C–F}$ = 22.8 Hz), 47.14, 35.62, 31.16 (d, $^{2}J_{C–F}$ = 21.2 Hz), 27.65 (d, $^{3}J_{C–F}$ = 3.6 Hz), 20.95 (d, $^{3}J_{C–F}$ = 1.8 Hz).

$^{19}F\{1H\}$ NMR (377 MHz, $CDCl_3$) δ -189.84 (major diastereomer), -192.15 (minor diastereomer).

HRMS (ESI/QTOF) m/z: $[M + Na]^+$ Calcd. for $C_{20}H_{21}BrFNNaO^+$ 412.0683; Found 412.0687.

$[\alpha]_D^{25}$ = +15.2 (c = 0.50 in $CHCl_3$).

HPLC: The enantiomeric excess (98%) was determined *via* HPLC analysis using a CHIRALCEL® IA column, with hexane:isopropanol = 95:5 at a flow rate 1.0 mL/min detected at 254 nm wavelength. Retention time: t_{major} = 20.5 min and t_{minor} = 39.4 min. Diastereomeric ratio (93:7) was determined by ^{19}F-NMR analysis of the crude reaction mixture.

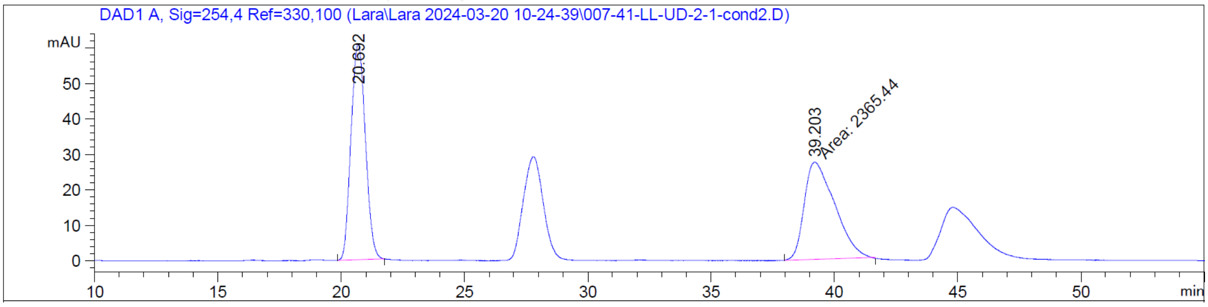

Peak #	RetTime [min]	Type	Width [min]	Area [mAU*s]	Height [mAU]	Area %
1	20.692	BB	0.4811	2466.13721	60.75276	51.0421
2	39.203	MM	1.4330	2365.43970	27.51120	48.9579

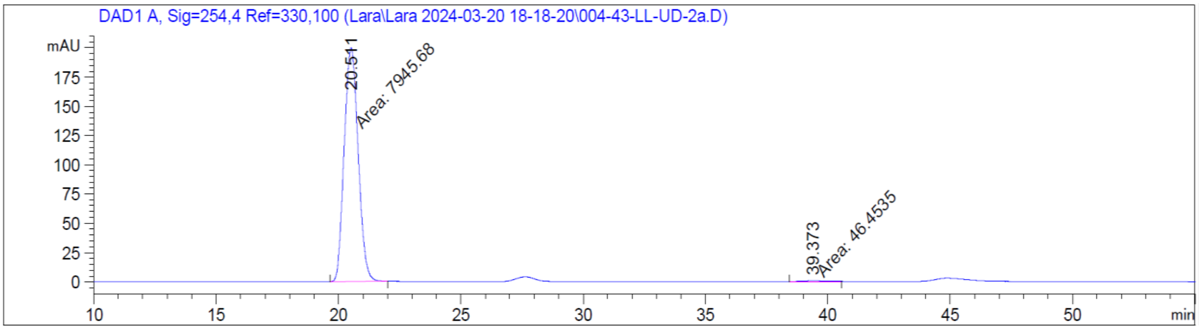

Peak #	RetTime [min]	Type	Width [min]	Area [mAU*s]	Height [mAU]	Area %
1	20.511	MM	0.6622	7945.68164	199.99278	99.4188
2	39.373	MM	1.0837	46.45350	7.14424e-1	0.5812

(*S*)-1-(3-Acetylphenyl)-3-{(*R*)-1-fluoro-4-phenylbutyl}pyrrolidin-2-one (3ad)

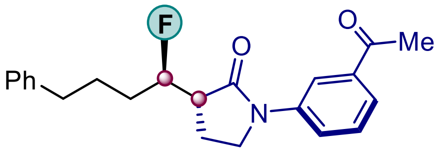

Prepared according to **GP1** with 3-bromo-1-(4-methoxyphenyl)pyrrolidin-2-one (**2d**) (27.0 mg, 0.10 mmol, 1.0 equiv.) and (*Z*)-(4-fluorobut-3-en-1-yl)benzene (**1a**) (19.5 mg, 0.13 mmol, 1.3 equiv.). Automated flash column chromatography (10 g SiO_2, gradient elution: hexane to 30% EtOAc in hexane) afforded the desired product (+) **3ad** as a yellow oil (31.0 mg, 88%) in 98:2 diastereomeric ratio.

^{1}H NMR (400 MHz, $CDCl_3$) δ 8.11 (t, *J* = 2.0 Hz, 1H), 7.97 (ddd, *J* = 8.2, 2.4, 1.0 Hz, 1H), 7.74 (dt, *J* = 7.8, 1.2 Hz, 1H), 7.47 (t, *J* = 8.0 Hz, 1H), 7.31 – 7.23 (m, 2H), 7.21 – 7.15 (m, 3H), 4.87 (ddt, *J* = 47.8, 9.6, 3.3 Hz, 1H), 3.95 – 3.81 (m, 2H), 3.04 (dddd, *J* = 23.9, 9.5, 7.4, 3.4 Hz, 1H), 2.68 (t, *J* = 7.6 Hz, 2H), 2.62 (s, 3H), 2.35 (dddd, *J* = 13.1, 9.4, 8.2, 5.0 Hz, 1H), 2.25 – 2.14 (m, 1H), 2.06 (dtd, *J* = 19.3, 9.3, 4.7 Hz, 1H), 1.89 (dddd, *J* = 11.6, 10.2, 6.0, 2.6 Hz, 1H), 1.80 – 1.62 (m, 2H).
^{13}C NMR (101 MHz, $CDCl_3$) (one resonance is missing due to overlap): δ 197.86, 172.25 (d, $^3J_{C–F}$ = 9.2 Hz), 141.96, 139.66, 137.68, 129.14, 128.43, 128.37, 125.87, 124.61, 119.02, 94.54 (d, $^1J_{C–F}$ = 172.4 Hz), 47.16 (d, $^2J_{C–F}$ = 22.7 Hz), 47.11, 35.51, 31.10 (d, $^2J_{C–F}$ = 20.9 Hz), 27.54 (d, $^3J_{C–F}$ = 3.7 Hz), 26.74, 20.94 (d, $^3J_{C–F}$ = 2.2 Hz).
^{19}F{1H} NMR (376 MHz, $CDCl_3$) δ -189.81 (major diastereomer), -192.18 (minor diastereomer).
HRMS (ESI/QTOF) m/z: $[M + Na]^+$ Calcd for $C_{22}H_{24}FNNaO_2^+$ 376.1683; Found 376.1693.
$[\alpha]_D^{23}$ = +5.3 (c = 0.90 in $CHCl_3$).
HPLC: The enantiomeric excess (96%) was determined *via* HPLC analysis using a CHIRALCEL® IA column, with hexane:isopropanol = 90:10 at a flow rate 1.0 mL/min detected at 254 nm wavelength. Retention time: t_{major} = 21.3 min and t_{minor} = 31.3 min. Diastereomeric ratio (96:4) was determined by ^{19}F-NMR of the crude reaction mixture.

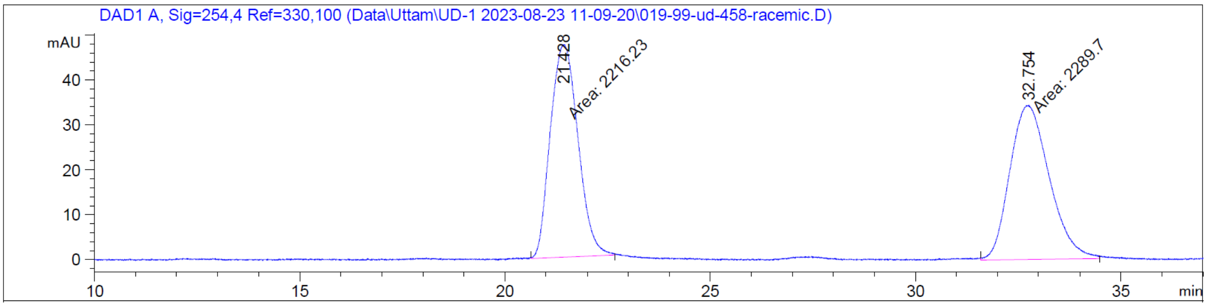

Peak #	RetTime [min]	Type	Width [min]	Area [mAU*s]	Height [mAU]	Area %
1	21.428	MM	0.7802	2216.23413	47.34591	49.1848
2	32.754	MM	1.1133	2289.69824	34.27668	50.8152

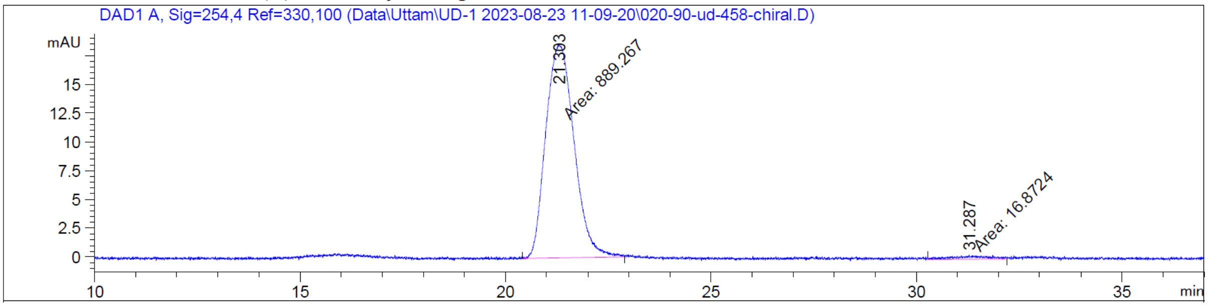

Peak #	RetTime [min]	Type	Width [min]	Area [mAU*s]	Height [mAU]	Area %
1	21.303	MM	0.7989	889.26691	18.55163	98.1380
2	31.287	MM	0.7569	16.87244	3.71538e-1	1.8620

Methyl 4-((*S*)-3-((*R*)-1-fluoro-4-phenylbutyl)-2-oxopyrrolidin-1-yl)benzoate (3ae)

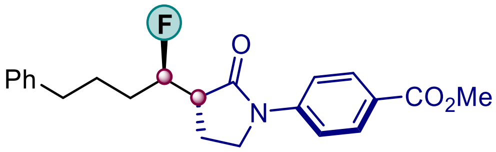

Prepared according to **GP1** with methyl 4-(3-bromo-2-oxopyrrolidin-1-yl)benzoate (**2e**) (29.8 mg, 0.10 mmol, 1.0 equiv.) and (*Z*)-(4-fluorobut-3-en-1-yl)benzene (**1a**) (19.5 mg, 0.13 mmol, 1.3 equiv.). Automated flash column chromatography (10 g SiO_2, gradient elution: hexane to 40% EtOAc in hexane) afforded the desired product (+) **3ae** as a yellow oil (33.5 mg, 91%) in 96:4 diastereomeric ratio.

^{1}H NMR (400 MHz, $CDCl_3$) δ 8.05 (d, J = 8.7 Hz, 2H), 7.72 (d, J = 8.7 Hz, 2H), 7.31 – 7.24 (m, 2H), 7.22 – 7.13 (m, 3H), 4.86 (ddt, J = 47.7, 9.7, 3.3 Hz, 1H), 3.91 (s, 3H), 3.84 (dtd, J = 17.5, 9.0, 5.9 Hz, 2H), 3.04 (dddd, J = 23.9, 10.5, 7.7, 3.4 Hz, 1H), 2.68 (t, J = 7.7 Hz, 2H), 2.34 (dtd, J = 13.6, 8.7, 4.8 Hz, 1H), 2.26 – 2.14 (m, 1H), 2.06 (dtt, J = 14.6, 9.6, 4.8 Hz, 1H), 1.88 (q, J = 9.1 Hz, 1H), 1.81 – 1.64 (m, 2H).

^{13}C NMR (101 MHz, $CDCl_3$) δ 172.38 (d, $^3J_{C–F}$ = 9.5 Hz), 166.59, 143.13, 141.94, 130.51, 128.43, 128.38, 125.88, 125.85, 118.83, 94.48 (d, $^1J_{C–F}$ = 172.4 Hz), 52.08, 47.27 (d, $^2J_{C–F}$ = 22.7 Hz), 46.92, 35.49, 31.08 (d, $^2J_{C–F}$ = 21.3 Hz), 27.52 (d, $^3J_{C–F}$ = 3.7 Hz), 20.86 (d, $^3J_{C–F}$ = 1.8 Hz).

^{19}F{1H} NMR (376 MHz, $CDCl_3$) δ -189.82 (major diastereomer), -192.08 (minor diastereomer).

HRMS (ESI/QTOF) m/z: $[M + Na]^+$ Calcd for $C_{22}H_{24}FNNaO_3^+$ 392.1632; Found 392.1643.

$[\alpha]_D^{23}$ = +104.7 (c = 0.50 in $CHCl_3$).

HPLC: The enantiomeric excess (98%) was determined *via* HPLC analysis using a CHIRALCEL® IA column, with hexane:isopropanol = 80:20 at a flow rate 1.0 mL/min detected at 254 nm wavelength. Retention time: t_{major} = 16.4 min and t_{minor} = 20.3 min. Diastereomeric ratio (96:4) was determined by ^{19}F-NMR of the crude reaction mixture.

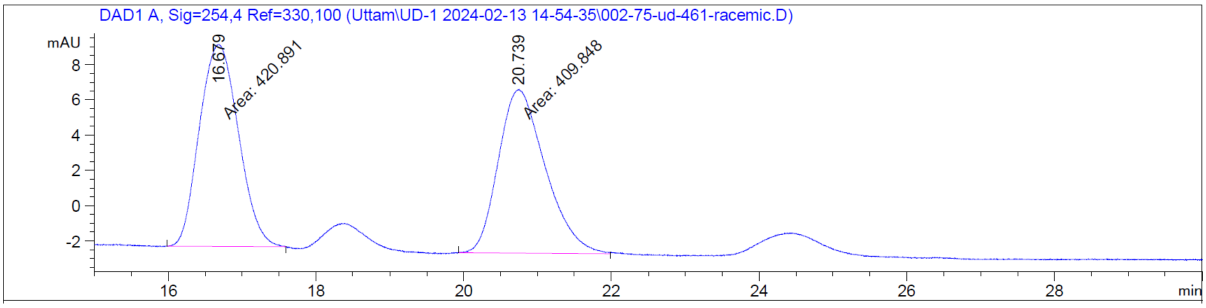

Peak #	RetTime [min]	Type	Width [min]	Area [mAU*s]	Height [mAU]	Area %
1	16.679	MM	0.6122	420.89105	11.45927	50.6647
2	20.739	MM	0.7363	409.84756	9.27699	49.3353

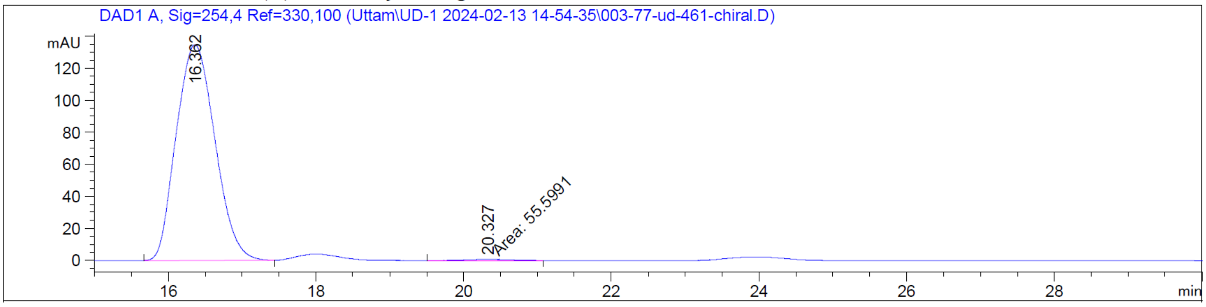

Peak #	RetTime [min]	Type	Width [min]	Area [mAU*s]	Height [mAU]	Area %
1	16.362	BB	0.4345	4899.51563	134.64937	98.8779
2	20.327	MM	0.8645	55.59914	1.07194	1.1221

(*S*)-3-{(*R*)-1-Fluoro-4-phenylbutyl}-1-{4-(methylsulfonyl)phenyl}pyrrolidin-2-one (3af)

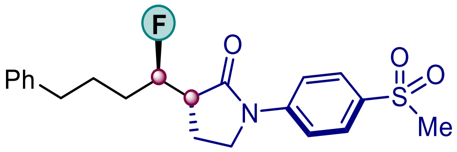

Prepared according to **GP1** with 3-bromo-1-(4-(methylsulfonyl)phenyl)pyrrolidin-2-one (**2f**) (32 mg, 0.10 mmol, 1.0 equiv.) and (*Z*)-(4-fluorobut-3-en-1-yl)benzene (**1a**) (20 mg, 0.13 mmol, 1.3 equiv.). Automated flash column chromatography (10 g SiO_2, gradient elution: hexane to 50% EtOAc in hexane) afforded the desired product **3af** as a white solid (29 mg, 75%) in 95:5 diastereomeric ratio.

^{1}H NMR (400 MHz, $CDCl_3$) δ 7.96 – 7.91 (m, 2H), 7.89 – 7.83 (m, 2H), 7.31 – 7.25 (m, 2H), 7.22 – 7.16 (m, 3H), 4.85 (ddt, *J* = 47.7, 9.6, 3.3 Hz, 1H), 3.86 (dtd, *J* = 29.2, 9.1, 5.8 Hz, 2H), 3.11 – 2.96 (m, 1H), 3.04 (s, 3H), 2.68 (t, *J* = 7.7 Hz, 2H), 2.38 (dddd, *J* = 13.1, 9.5, 8.3, 5.1 Hz, 1H), 2.28 – 2.15 (m, 1H), 2.07 (dtd, *J* = 19.3, 9.2, 4.6 Hz, 1H), 1.88 (dddd, *J* = 16.4, 11.3, 6.2, 2.8 Hz, 1H), 1.82 – 1.62 (m, 2H).

^{13}C NMR (101 MHz, $CDCl_3$) (one resonance is missing due to overlap): δ 172.77 (d, $^3J_{C–F}$ = 8.7 Hz), 143.88, 141.96, 135.67, 128.51, 128.48, 126.00, 119.61, 94.62 (d, $^1J_{C–F}$ = 172.6 Hz), 47.24 (d, $^2J_{C–F}$ = 22.7 Hz), 47.01, 44.79, 35.55, 31.28 (d, $^2J_{C–F}$ = 21.0 Hz), 27.55 (d, $^3J_{C–F}$ = 3.8 Hz), 21.13 (d, $^3J_{C–F}$ = 1.8 Hz).

^{19}F{1H} NMR (377 MHz, $CDCl_3$) δ -189.92 (major diastereomer), -192.19 (minor diastereomer).

HRMS (ESI/QTOF) m/z: $[M + Na]^+$ Calcd for $C_{21}H_{24}FNNaO_3S^+$ 412.1353; Found 412.1355.

$[\alpha]_D^{25}$ = +13.2 (c = 0.50 in $CHCl_3$).

HPLC: The enantiomeric excess (95%) was determined *via* HPLC analysis using a CHIRALCEL® IA column, with hexane:isopropanol = 90:10 at a flow rate 1.0 mL/min detected at 254 nm wavelength. Retention time: t_{major} = 37.9 min and t_{minor} = 32.7 min. Diastereomeric ratio (93:7) was determined by ^{19}F-NMR analysis of the crude reaction mixture.

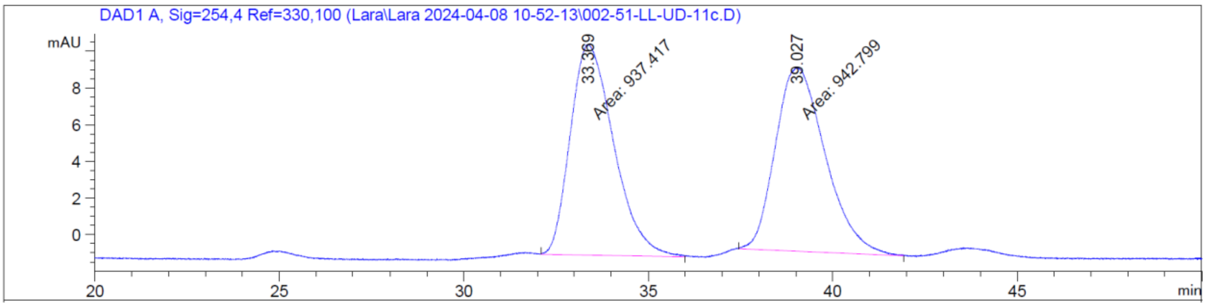

Peak #	RetTime [min]	Type	Width [min]	Area [mAU*s]	Height [mAU]	Area %
1	33.369	MM	1.3613	937.41748	11.47728	49.8569
2	39.027	MM	1.5710	942.79932	10.00212	50.1431

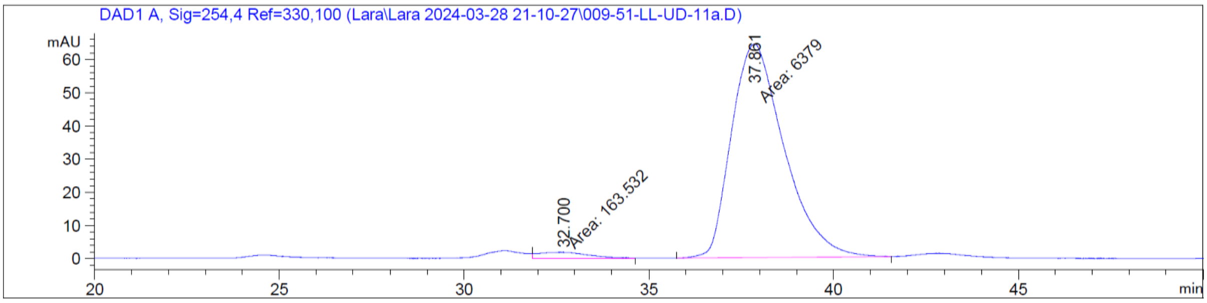

Peak #	RetTime [min]	Type	Width [min]	Area [mAU*s]	Height [mAU]	Area %
1	32.700	MM	1.5323	163.53218	1.77876	2.4995
2	37.861	MM	1.6522	6378.99805	64.34884	97.5005

(*S*)-3-{(*R*)-1-Fluoro-4-phenylbutyl}-1-(1-methyl-1*H*-indol-6-yl)pyrrolidin-2-one (3ag)

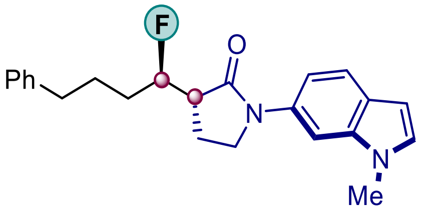

Prepared according to **GP1** with 3-bromo-1-(1-methyl-1*H*-indol-6-yl)pyrrolidin-2-one (**2g**) (29 mg, 0.10 mmol, 1.0 equiv.) and (*Z*)-(4-fluorobut-3-en-1-yl)benzene (**1a**) (19.5 mg, 0.13

mmol, 1.3 equiv.). Automated flash column chromatography (10 g SiO_2, gradient elution: hexane to 30% EtOAc in hexane) afforded the desired product **3ag** as a brown solid (31.5 mg, 86%) in >99:1 diastereomeric ratio.

^{1}H NMR (400 MHz, $CDCl_3$) δ 7.66 (d, J = 2.1 Hz, 1H), 7.46 (dd, J = 8.8, 2.1 Hz, 1H), 7.32 – 7.26 (m, 3H), 7.23 – 7.15 (m, 3H), 7.06 (d, J = 3.1 Hz, 1H), 6.47 (dd, J = 3.1, 0.8 Hz, 1H), 4.94 (ddt, J = 47.7, 9.7, 3.3 Hz, 1H), 3.90 – 3.84 (m, 2H), 3.79 (s, 3H), 3.09 (dddd, J = 21.9, 9.5, 7.3, 3.3 Hz, 1H), 2.73 – 2.65 (m, 2H), 2.32 (dddd, J = 13.1, 9.5, 7.2, 5.9 Hz, 1H), 2.24 – 2.14 (m, 1H), 2.12 – 2.01 (m, 1H), 1.98 – 1.86 (m, 1H), 1.84 – 1.64 (m, 2H).

^{13}C NMR (101 MHz, $CDCl_3$) δ 171.79 (d, $^{3}J_{C–F}$ = 10.8 Hz), 142.23, 134.70, 131.65, 129.89, 128.58, 128.49, 128.46, 125.91, 116.47, 113.76, 109.40, 101.27, 94.82 (d, $^{1}J_{C–F}$ = 171.5 Hz), 48.70, 47.17 (d, $^{2}J_{C–F}$ = 22.9 Hz), 35.69, 33.09, 30.98 (d, $^{2}J_{C–F}$ = 21.1 Hz), 27.74 (d, $^{3}J_{C–F}$ = 3.5 Hz), 20.90 (d, $^{3}J_{C–F}$ = 1.7 Hz).

^{19}F{1H} NMR (377 MHz, $CDCl_3$) δ -189.48.

HRMS (ESI/QTOF) m/z: $[M + Na]^+$ Calcd. for $C_{23}H_{25}FN_2NaO^+$ 387.1843; Found 387.1852.

$[\alpha]_D^{25}$ = +32.1 (c = 0.50 in $CHCl_3$).

HPLC: The enantiomeric excess (99%) was determined *via* HPLC analysis using a CHIRALCEL® IA column, with hexane:isopropanol = 80:20 at a flow rate 1.0 mL/min detected at 254 nm wavelength. Retention time: t_{major} = 18.6 min and t_{minor} = 32.9 min. Diastereomeric ratio (95:5) was determined by ^{19}F-NMR analysis of the crude reaction mixture.

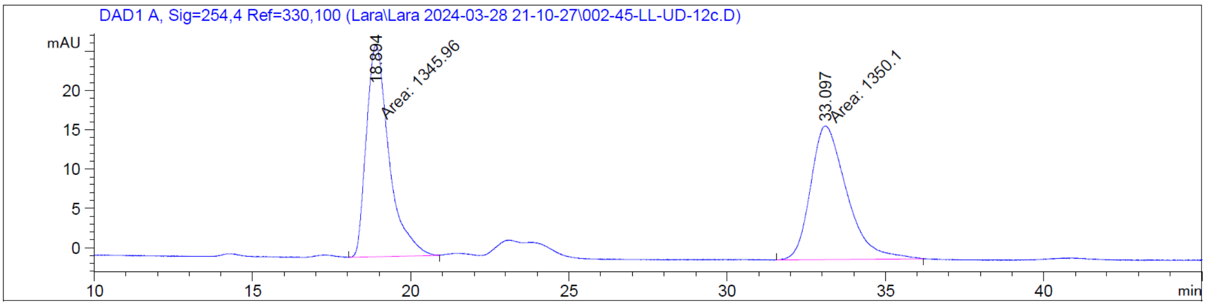

Peak #	RetTime [min]	Type	Width [min]	Area [mAU*s]	Height [mAU]	Area %
1	18.894	MM	0.8309	1345.96057	26.99917	49.9232
2	33.097	MM	1.3236	1350.09998	16.99991	50.0768

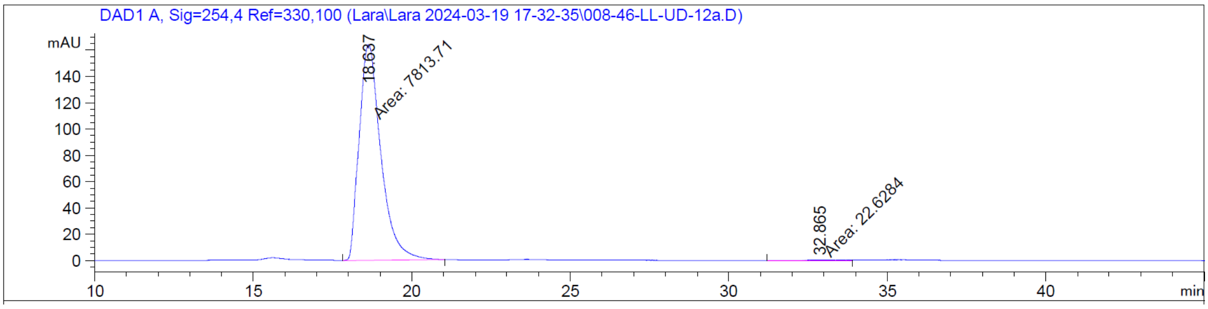

Peak #	RetTime [min]	Type	Width [min]	Area [mAU*s]	Height [mAU]	Area %
1	18.637	MM	0.7928	7813.71143	164.26657	99.7112
2	32.865	MM	1.4629	22.62836	2.57800e-1	0.2888

(*S*)-1-(*tert*-Butyl)-3-{(*R*)-1-fluoro-4-phenylbutyl}pyrrolidin-2-one (3ah)

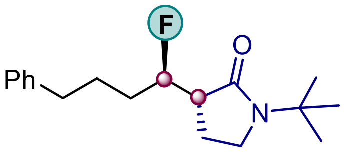

Prepared according to **GP1** with 3-bromo-1-(*tert*-butyl)pyrrolidin-2-one (**2h**) (22 mg, 0.10 mmol, 1.0 equiv.) and (*Z*)-(4-fluorobut-3-en-1-yl)benzene (**1a**) (23 mg, 0.15 mmol, 1.5 equiv.). Automated flash column chromatography (10 g SiO_2, gradient elution: hexane to 10% EtOAc in hexane) afforded the desired product (+) **3ah** as a transparent liquid (23 mg, 79%) in 99:1 diastereomeric ratio.

^{1}H NMR (400 MHz, $CDCl_3$) δ 7.30 – 7.24 (m, 2H), 7.20 – 7.15 (m, 3H), 4.78 (ddt, J = 47.8, 9.7, 3.3 Hz, 1H), 3.48 – 3.31 (m, 2H), 2.87 – 2.74 (m, 1H), 2.66 (t, J = 7.6 Hz, 2H), 2.06 (dddd, J = 13.2, 9.7, 8.2, 5.1 Hz, 1H), 2.00 – 1.79 (m, 3H), 1.78 – 1.67 (m, 1H), 1.66 – 1.49 (m, 1H), 1.37 (s, 9H).

^{13}C NMR (101 MHz, $CDCl_3$) δ 173.16 (d, $^3J_{C\text{–}F}$ = 11.1 Hz), 142.25, 128.58, 128.44, 125.90, 94.94 (d, $^1J_{C\text{–}F}$ = 170.8 Hz), 54.31, 47.47 (d, $^2J_{C\text{–}F}$ = 22.4 Hz), 44.39, 35.67, 30.76 (d, $^2J_{C\text{–}F}$ = 21.2 Hz), 27.72, 27.68, 20.46 (d, $^3J_{C\text{–}F}$ = 1.5 Hz).

^{19}F{1H} NMR (377 MHz, $CDCl_3$) δ -190.03.

HRMS (ESI/QTOF) m/z: [M + Na]$^+$ Calcd. for $C_{18}H_{26}FNNaO^+$ 314.1891; Found 314.1897.

$[\alpha]_D^{25}$ = +12.4 (c = 0.50 in $CHCl_3$).

HPLC: The enantiomeric excess (99%) was determined *via* HPLC analysis using a CHIRALCEL® IA column, with hexane:isopropanol = 95:5 at a flow rate 0.5 mL/min detected

at 254 nm wavelength. Retention time: t_{major} = 12.3 min and t_{minor} = 14.0 min. Diastereomeric ratio (96:4) was determined by ^{19}F-NMR analysis of the crude reaction mixture.

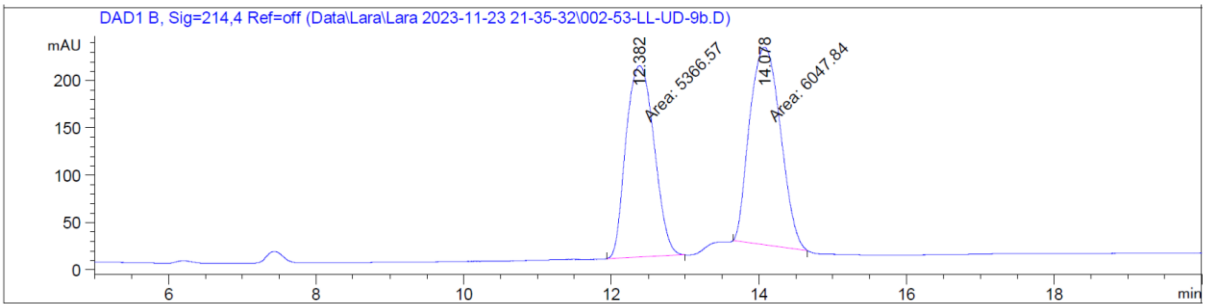

Peak #	RetTime [min]	Type	Width [min]	Area [mAU*s]	Height [mAU]	Area %
1	12.382	MM	0.4423	5366.56982	202.23442	47.0158
2	14.078	MM	0.4831	6047.83594	208.63364	52.9842

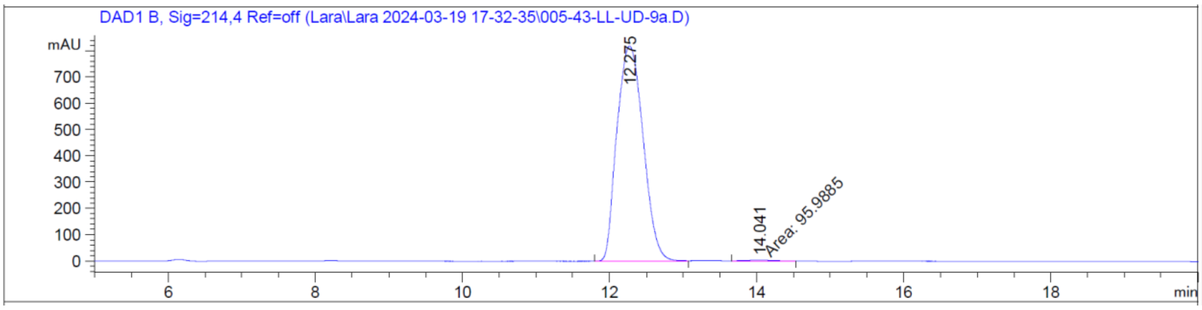

Peak #	RetTime [min]	Type	Width [min]	Area [mAU*s]	Height [mAU]	Area %
1	12.275	BB	0.2937	2.02456e4	818.06293	99.5281
2	14.041	MM	0.4516	95.98846	3.54226	0.4719

(*S*)-3-{(*R*)-1-Fluoro-4-phenylbutyl}-1-mesitylazetidin-2-one (3ai)

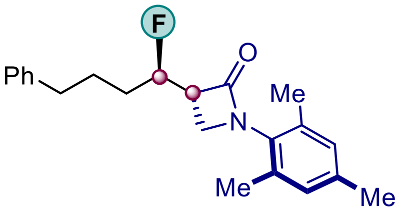

Prepared according to **GP1** with 3-bromo-1-mesitylazetidin-2-one (**2i**) (27 mg, 0.10 mmol, 1.0 equiv.) and (*Z*)-(4-fluorobut-3-en-1-yl)benzene (**1a**) (23 mg, 0.15 mmol, 1.5 equiv.). Automated flash column chromatography (10 g SiO_2, gradient elution: hexane to 10% EtOAc

in hexane) afforded the desired product (-) **3ai** as a white solid (25 mg, 74%) in 82:18 diastereomeric ratio.

The given data is for the major diastereomer.

^{1}H NMR (400 MHz, $CDCl_3$) δ 7.32 – 7.27 (m, 2H), 7.24 – 7.16 (m, 3H), 6.88 (s, 2H), 4.83 (ddt, J = 49.7, 9.4, 3.3 Hz, 1H), 3.71 – 3.53 (m, 3H), 2.77 – 2.64 (m, 2H), 2.27 (s, 3H), 2.24 (s, 6H), 2.23 – 2.15 (m, 1H), 1.99 – 1.71 (m, 3H).

^{13}C NMR (101 MHz, $CDCl_3$) δ 164.99 (d, $^{3}J_{C–F}$ = 6.0 Hz), 142.03, 138.29, 136.12, 131.55, 129.10, 128.58, 128.52, 126.03, 90.56 (d, $^{1}J_{C–F}$ = 171.9 Hz), 53.42 (d, $^{2}J_{C–F}$ = 23.5 Hz), 44.09 (d, $^{3}J_{C–F}$ = 3.8 Hz), 35.56, 32.30 (d, $^{2}J_{C–F}$ = 20.8 Hz), 27.55 (d, $^{3}J_{C–F}$ = 4.0 Hz), 21.09, 18.52.

^{19}F{1H} NMR (377 MHz, $CDCl_3$) δ -191.41 (major diastereomer), -187.93 (minor diastereomer).

HRMS (ESI/QTOF) m/z: $[M + Na]^+$ Calcd. for $C_{22}H_{26}FNNaO^+$ 362.1891; Found 362.1897.

$[\alpha]_D^{25}$ = -12.5 (c = 0.50 in $CHCl_3$).

HPLC: The enantiomeric excess (99%) of major diastereomer was determined *via* HPLC analysis using a CHIRALCEL® AD-H column, with hexane:isopropanol = 95:5 at a flow rate 1.0 mL/min detected at 210 nm wavelength. Retention time: t_{major} = 20.2 min and t_{minor} = 24.7 min.

Diastereomeric ratio (82:18) was determined by ^{19}F-NMR analysis of the crude reaction mixture.

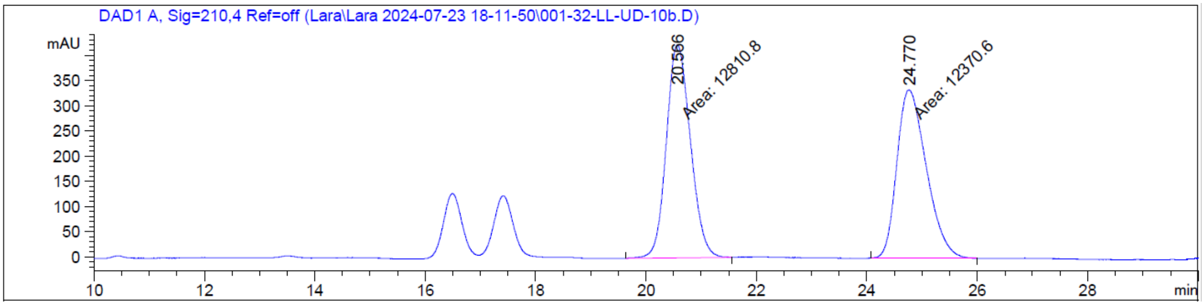

Peak #	RetTime [min]	Type	Width [min]	Area [mAU*s]	Height [mAU]	Area %
1	20.566	MM	0.5054	1.28108e4	422.43903	50.8741
2	24.770	MM	0.6177	1.23706e4	333.76398	49.1259

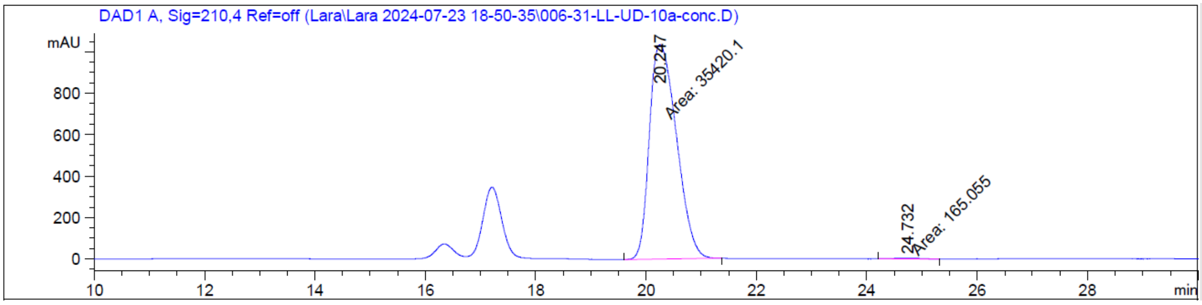

Peak #	RetTime [min]	Type	Width [min]	Area [mAU*s]	Height [mAU]	Area %
1	20.247	MM	0.5695	3.54201e4	1036.56848	99.5362
2	24.732	MM	0.5474	165.05467	5.02511	0.4638

HPLC: The enantiomeric excess (71%) of minor diastereomer was determined *via* HPLC analysis using a CHIRALCEL® AD-H column, with hexane:isopropanol = 95:5 at a flow rate 1.0 mL/min detected at 210 nm wavelength. Retention time: t_{major} = 17.2 min and t_{minor} = 16.4 min.

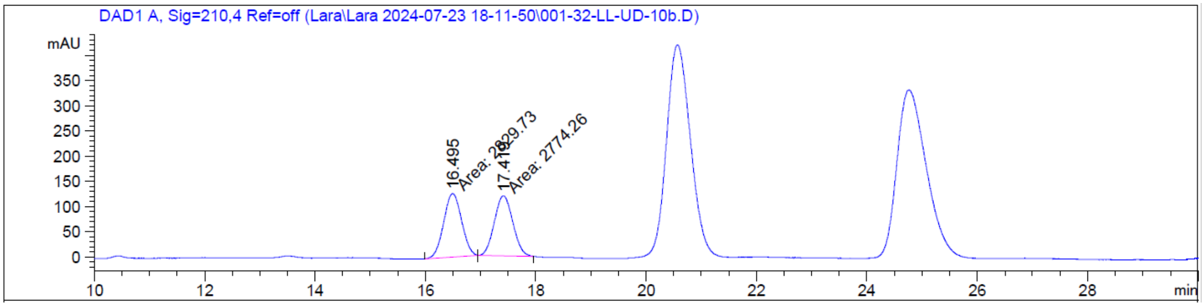

Peak #	RetTime [min]	Type	Width [min]	Area [mAU*s]	Height [mAU]	Area %
1	16.495	MM	0.3750	2829.72949	125.77112	50.4949
2	17.419	MM	0.3880	2774.25781	119.15903	49.5051

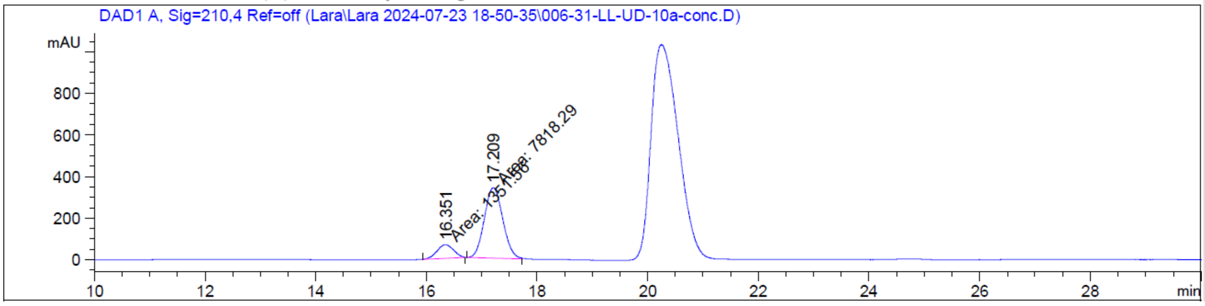

Peak #	RetTime [min]	Type	Width [min]	Area [mAU*s]	Height [mAU]	Area %
1	16.351	MM	0.3451	1351.55750	65.27006	14.7392
2	17.209	MM	0.3846	7818.28516	338.77704	85.2608

(*S*)-3-{(*R*)-1-Fluoro-4-phenylbutyl}-1-phenylpiperidin-2-one (3aj)

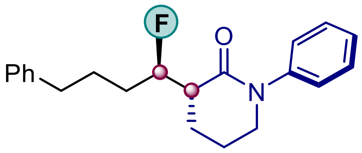

Prepared according to **GP1** with 3-bromo-1-phenylpiperidin-2-one (**2j)** (25.4 mg, 0.10 mmol, 1.0 equiv.) and (*Z*)-(4-fluorobut-3-en-1-yl)benzene (**1a**) (19.5 mg, 0.13 mmol, 1.3 equiv.). Automated flash column chromatography (10 g SiO_2, gradient elution: hexane to 10% EtOAc in hexane) afforded the desired product (+) **3aj** as a yellow oil (27.0 mg, 83%) in >99:1 diastereomeric ratio.

^{1}H NMR (400 MHz, $CDCl_3$) δ 7.41 (dd, *J* = 8.3, 7.2 Hz, 2H), 7.34 – 7.26 (m, 3H), 7.25 – 7.14 (m, 5H), 5.32 – 5.07 (m, 1H), 3.74 – 3.59 (m, 2H), 3.04 (dddd, *J* = 14.5, 10.0, 6.0, 3.9 Hz, 1H), 2.76 – 2.61 (m, 2H), 2.13 (dtt, *J* = 13.9, 7.4, 4.0 Hz, 2H), 2.02 – 1.83 (m, 4H), 1.82 – 1.62 (m, 2H).

^{13}C NMR (101 MHz, $CDCl_3$) δ 168.91 (d, $^{3}J_{C–F}$ = 13.2 Hz), 143.05, 142.22, 129.21, 128.47, 128.32, 126.90, 126.23, 125.75, 94.78 (d, $^{1}J_{C–F}$ = 169.1 Hz), 51.55, 46.14 (d, $^{2}J_{C–F}$ = 23.8 Hz), 35.57, 31.00 (d, $^{2}J_{C–F}$ = 21.3 Hz), 27.93 (d, $^{3}J_{C–F}$ = 2.9 Hz), 22.54, 22.03 (d, $^{3}J_{C–F}$ = 1.8 Hz).

^{19}F{1H} NMR (376 MHz, $CDCl_3$) δ -189.99.

HRMS (ESI/QTOF) m/z: $[M + Na]^+$ Calcd for $C_{21}H_{24}FNNaO^+$ 348.1734; Found 348.1745.

$[\alpha]_D^{23}$ = +99.8 (c = 0.50 in $CHCl_3$).

HPLC: The enantiomeric excess (99.4%) was determined *via* HPLC analysis using a CHIRALCEL® IA column, with hexane:isopropanol = 90:10 at a flow rate 1.0 mL/min detected at 254 nm wavelength. Retention time: t_{major} = 12.9 min and t_{minor} = 10.0 min. Diastereomeric ratio (98:2) was determined by ^{19}F-NMR of the crude reaction mixture.

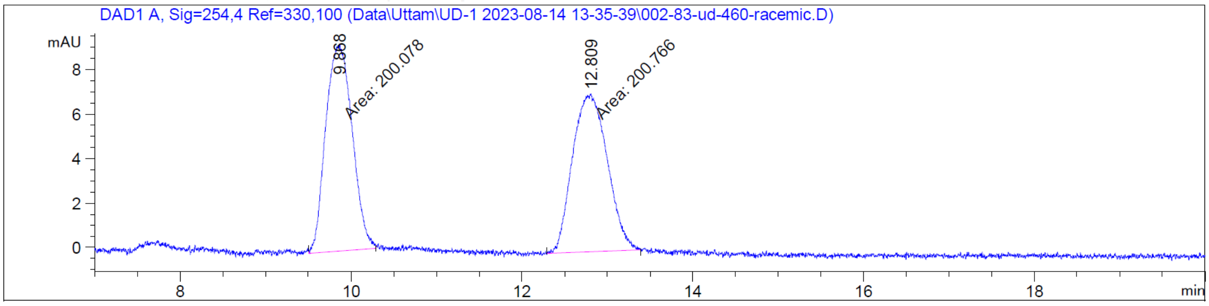

Peak #	RetTime [min]	Type	Width [min]	Area [mAU*s]	Height [mAU]	Area %
1	9.868	MM	0.3585	200.07799	9.30150	49.9142
2	12.809	MM	0.4723	200.76620	7.08486	50.0858

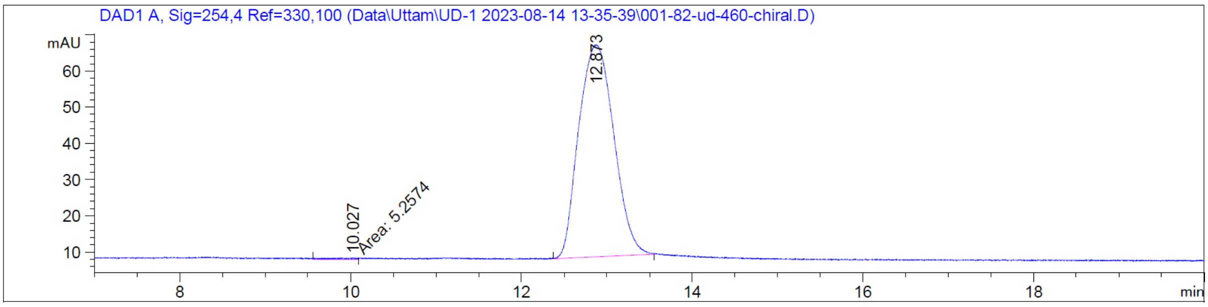

Peak #	RetTime [min]	Type	Width [min]	Area [mAU*s]	Height [mAU]	Area %
1	10.027	MM	0.2678	5.25740	3.27247e-1	0.3121
2	12.873	VV	0.3353	1679.11938	58.68866	99.6879

(2*S*,3*R*)-3-Fluoro-*N*-(4-methoxyphenyl)-2-methyl-6-phenylhexanamide (5aa)

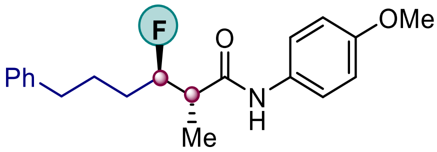

Prepared according to **GP2** with 2-iodo-*N*-(4-methoxyphenyl)propenamide (**4a**) (30.5 mg, 0.10 mmol, 1.0 equiv.) and (*Z*)-(4-fluorobut-3-en-1-yl)benzene (**1a**) (30.0 mg, 0.20 mmol, 2.0 equiv.). Automated flash column chromatography (10 g SiO_2, gradient elution: hexane to 10% EtOAc in hexane) afforded the desired product (-) **5aa** as a yellow oil (21.0 mg, 64%) in >99:1 diastereomeric ratio.

^{1}H NMR (400 MHz, $CDCl_3$) δ 7.44 – 7.38 (m, 2H), 7.32 – 7.26 (m, 3H), 7.22 – 7.15 (m, 3H), 6.89 – 6.82 (m, 2H), 4.80 – 4.57 (m, 1H), 3.79 (s, 3H), 2.80 – 2.45 (m, 3H), 1.90 – 1.64 (m, 4H), 1.24 (d, J = 7.1 Hz, 3H).

^{13}C NMR (100 MHz, $CDCl_3$) (one resonance is missing due to overlap): δ 171.27 (d, $^3J_{C–F}$ = 1.7 Hz), 156.51, 141.78, 130.83, 128.40, 125.93, 121.89, 114.14, 95.5 (d, $^1J_{C–F}$ = 170.1 Hz), 55.51, 47.07 (d, $^2J_{C–F}$ = 19.3 Hz), 35.43, 32.01 (d, $^2J_{C–F}$ = 21.1 Hz), 26.57 (d, $^3J_{C–F}$ = 3.65 Hz), 13.87 (d, $^3J_{C–F}$ = 7.34 Hz).

^{19}F{1H} NMR (376 MHz, $CDCl_3$) δ -182.09.

HRMS (APCI/QTOF) m/z: $[M + Na]^+$ Calcd for $C_{20}H_{24}FNNaO_2^+$ 352.1683; Found 352.1683.

$[\alpha]_D^{23}$ = –5.3 (c = 0.50 in $CHCl_3$).

HPLC: The enantiomeric excess (99%) was determined *via* HPLC analysis using a CHIRALCEL® OJ-H column, with hexane:isopropanol = 75:25 at a flow rate 1.0 mL/min detected at 254 nm wavelength. Retention time: t_{major} = 22.5 min and t_{minor} = 30.3 min. Diastereomeric ratio (98:2) was determined by ^{19}F-NMR of the crude reaction mixture.

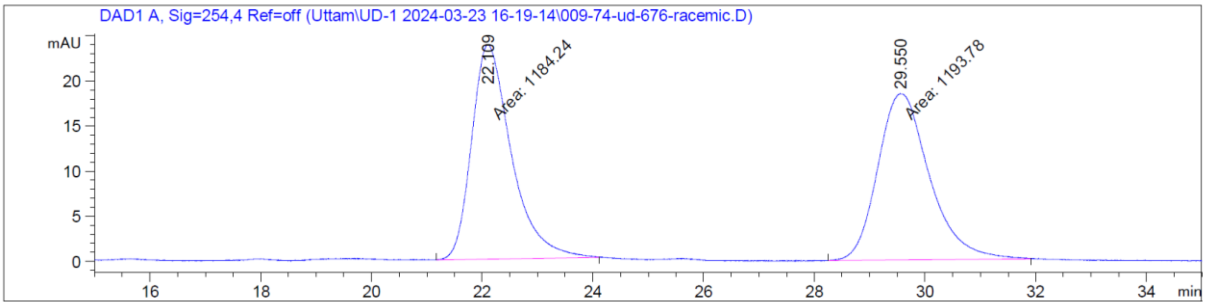

Peak #	RetTime [min]	Type	Width [min]	Area [mAU*s]	Height [mAU]	Area %
1	22.109	MM	0.8314	1184.24121	23.73850	49.7995
2	29.550	MM	1.0806	1193.77942	18.41285	50.2005

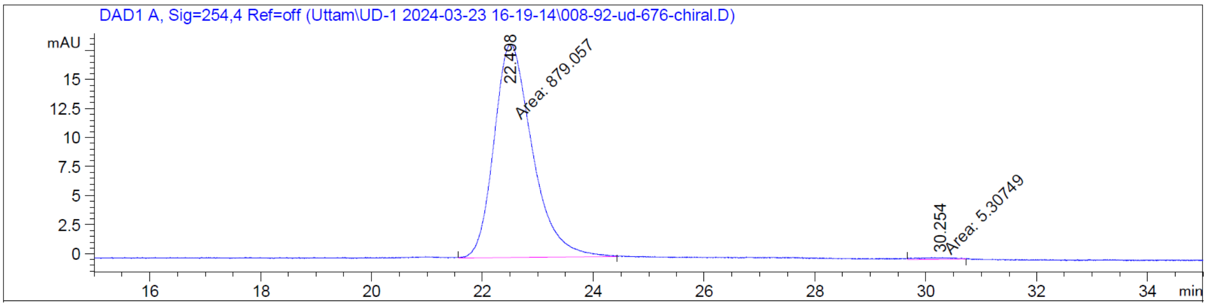

Peak #	RetTime [min]	Type	Width [min]	Area [mAU*s]	Height [mAU]	Area %
1	22.498	MM	0.7992	879.05750	18.33305	99.3999
2	30.254	MM	0.6754	5.30749	1.30965e-1	0.6001

(2*S*,3*R*)-8-Chloro-3-fluoro-*N*-(4-methoxyphenyl)-2-methyloctanamide (5ba)

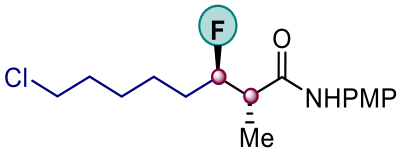

Prepared according to **GP2** with 2-iodo-*N*-(4-methoxyphenyl)propenamide (**4a**) (30.5 mg, 0.10 mmol, 1.0 equiv.) and (*Z*)-6-chloro-1-fluorohex-1-ene (**1b**) (24.5 mg, 0.20 mmol, 2.0 equiv.). Automated flash column chromatography (10 g SiO_2, gradient elution: hexane to 10% EtOAc in hexane) afforded the desired product (-) **5ba** as a yellow oil (21.5 mg, 68%) in >99:1 diastereomeric ratio.

^{1}H NMR (400 MHz, $CDCl_3$) δ 7.41 – 7.32 (m, 2H), 7.33 – 7.25 (m, 1H), 6.87 – 6.65 (m, 2H), 4.74 – 4.45 (m, 1H), 3.71 (d, *J* = 1.4 Hz, 3H), 3.46 (t, *J* = 6.6 Hz, 2H), 2.51 (dt, *J* = 17.1, 7.1 Hz, 1H), 1.72 (p, *J* = 6.6 Hz, 3H), 1.59 (d, *J* = 6.2 Hz, 1H), 1.43 (dp, *J* = 23.7, 7.8 Hz, 4H), 1.18 (d, *J* = 7.0 Hz, 3H).
^{13}C NMR (101 MHz, $CDCl_3$) δ 171.30 (d, $^{3}J_{C–F}$ = 1.8 Hz), 156.51, 130.86, 121.88, 114.14, 95.39 (d, $^{1}J_{C–F}$ = 170.6 Hz), 55.51, 47.06 (d, $^{2}J_{C–F}$ = 20.2 Hz), 44.90, 32.37 (d, $^{2}J_{C–F}$ = 20.3 Hz), 32.41, 26.61, 24.26 (d, $^{3}J_{C–F}$ = 3.3 Hz), 13.87 (d, $^{3}J_{C–F}$ = 7.3 Hz).
^{19}F{1H} NMR (376 MHz, $CDCl_3$) δ -182.06.
HRMS (ESI/QTOF) m/z: $[M + Na]^+$ Calcd for $C_{16}H_{23}ClFNNaO_2^+$ 338.1294; Found 338.1303.
$[\alpha]_D^{23}$ = –4.4 (c = 0.50 in $CHCl_3$).
HPLC: The enantiomeric excess (97%) was determined *via* HPLC analysis using a CHIRALCEL® OD-H column, with hexane:isopropanol = 90:10 at a flow rate 1.0 mL/min detected at 254 nm wavelength. Retention time: t_{major} = 30.5 min and t_{minor} = 34.7 min. Diastereomeric ratio (96:4) was determined by ^{19}F-NMR of the crude reaction mixture.

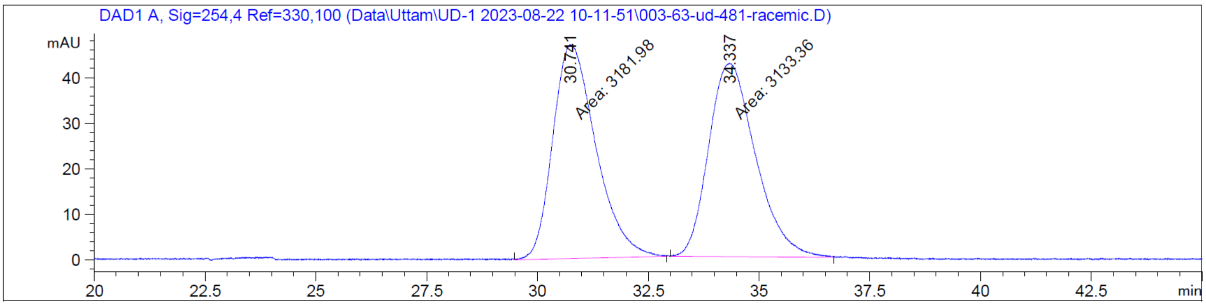

Peak #	RetTime [min]	Type	Width [min]	Area [mAU*s]	Height [mAU]	Area %
1	30.741	MM	1.1272	3181.98145	47.04675	50.3850
2	34.337	MM	1.2273	3133.35742	42.55229	49.6150

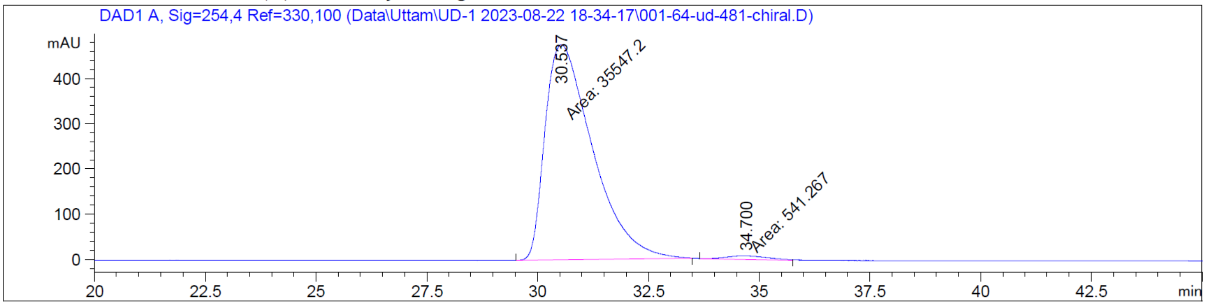

Peak #	RetTime [min]	Type	Width [min]	Area [mAU*s]	Height [mAU]	Area %
1	30.537	MM	1.2461	3.55472e4	475.43979	98.5002
2	34.700	MM	1.0658	541.26746	8.46426	1.4998

(2*S*,3*R*)-3-Fluoro-2-methyl-*N*-phenyl-6-(2-phenylacetamido)hexanamide (5cb)

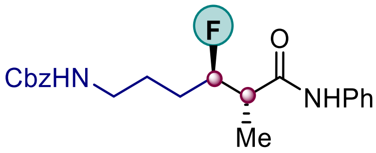

Prepared according to **GP2** with 2-iodo-*N*-phenylpropanamide (**4b**) (27.5 mg, 0.10 mmol, 1.0 equiv.) and benzyl (*Z*)-(4-fluorobut-3-en-1-yl)carbamate (**1c**) (44.6 mg, 0.20 mmol, 2.0 equiv.). Automated flash column chromatography (10 g SiO_2, gradient elution: hexane to 50% EtOAc in hexane) afforded the desired product (-) **5cb** as a yellow oil (20.5 mg, 55%) in >99:1 diastereomeric ratio.

^{1}H NMR (400 MHz, $CDCl_3$) δ 7.63 – 7.44 (m, 3H), 7.38 – 7.28 (m, 7H), 7.14 – 7.07 (m, 1H), 5.09 (s, 2H), 4.87 (bs, 1H), 4.68 (dd, *J* = 47.4, 7.3 Hz, 1H), 3.24 (q, *J* = 6.2 Hz, 2H), 2.83 – 2.43 (m, 1H), 1.86 – 1.64 (m, 4H), 1.24 (d, *J* = 7.0 Hz, 3H).

^{13}C NMR (101 MHz, $CDCl_3$) δ 171.36 (d, $^{3}J_{C–F}$ = 1.74 Hz), 156.50, 137.76, 136.53, 129.00, 128.56, 128.17, 128.12, 124.44, 119.99, 95.11 (d, $^{1}J_{C–F}$ = 171.5 Hz), 66.72, 47.05 (d, $^{2}J_{C–F}$ = 21.0 Hz), 40.60, 29.50 (d, $^{2}J_{C–F}$ = 21.2 Hz), 25.59 (d, $^{3}J_{C–F}$ = 2.8 Hz), 13.75 (d, $^{3}J_{C–F}$ = 7.1 Hz).

^{19}F{1H} NMR (376 MHz, $CDCl_3$) δ −181.00 - -181.50 (m, 1F)

HRMS (ESI/QTOF) m/z: $[M + Na]^+$ Calcd for $C_{21}H_{25}FN_2NaO_3^+$ 395.1741; Found 395.1760.

$[\alpha]_D^{23}$ = –10.7 (c = 1.00 in $CHCl_3$).

HPLC: The enantiomeric excess (95%) was determined *via* HPLC analysis using a CHIRALCEL® OD-H column, with hexane:isopropanol = 80:20 at a flow rate 1.0 mL/min detected at 254 nm wavelength. Retention time: t_{major} = 15.2 min and t_{minor} = 13.0 min. Diastereomeric ratio (98:2) was determined by ^{19}F-NMR of the crude reaction mixture.

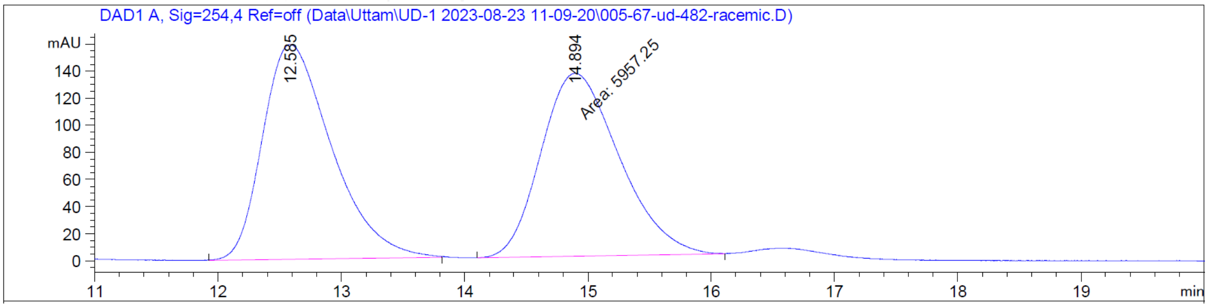

Peak #	RetTime [min]	Type	Width [min]	Area [mAU*s]	Height [mAU]	Area %
1	12.585	VV	0.4564	6158.24902	158.28972	50.8295
2	14.894	MM	0.7358	5957.25098	134.93597	49.1705

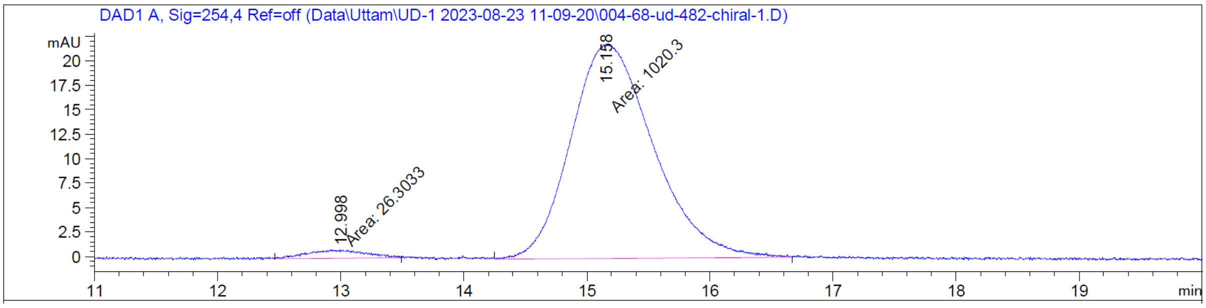

Peak #	RetTime [min]	Type	Width [min]	Area [mAU*s]	Height [mAU]	Area %
1	12.998	MM	0.5118	26.30334	8.56564e-1	2.5132
2	15.158	MM	0.7775	1020.29816	21.87148	97.4868

(2*S*,3*R*)-6-{(*tert*-Butyldimethylsilyl)oxy}-3-fluoro-2-methyl-*N*-phenylhexanamide (5db)

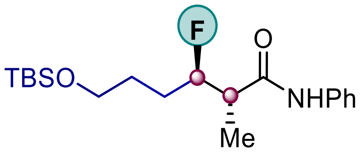

Prepared according to **GP2** with 2-iodo-*N*-phenylpropanamide (**4b**) (27.5 mg, 0.10 mmol, 1.0 equiv.) and (*Z*)-*tert*-butyl((4-fluorobut-3-en-1-yl)oxy)dimethylsilane (**1d**) (40.8 mg, 0.20 mmol, 2.0 equiv.). Automated flash column chromatography (10 g SiO_2, gradient elution: hexane to 10% EtOAc in hexane) afforded the desired product (-) **5db** as a yellow oil (19.0 mg, 54%) in >99:1 diastereomeric ratio.

^{1}H NMR (400 MHz, $CDCl_3$) δ 7.58 – 7.46 (m, 2H), 7.37 (bs, 1H), 7.35 – 7.29 (m, 2H), 7.15 – 7.07 (m, 1H), 4.71 (dtd, J = 47.8, 7.4, 3.0 Hz, 1H), 3.70 – 3.60 (m, 2H), 2.61 (dt, J = 17.3, 7.1 Hz, 1H), 1.92 – 1.61 (m, 4H), 1.27 (d, J = 7.2 Hz, 3H), 0.88 (s, 9H), 0.04 (s, 6H).
^{13}C NMR (101 MHz, $CDCl_3$) δ 171.49 (d, $^3J_{C–F}$ = 1.74 Hz), 137.77, 128.99, 124.38, 119.96, 95.54 (d, $^1J_{C–F}$ = 170.4 Hz), 62.41, 47.33 (d, $^2J_{C–F}$ = 20.2 Hz), 29.12 (d, $^2J_{C–F}$ = 21.0 Hz), 28.02 (d, $^3J_{C–F}$ = 3.2 Hz), 25.92, 18.30, 13.91 (d, $^3J_{C–F}$ = 7.1 Hz), -5.32.
^{19}F{1H} NMR (376 MHz, $CDCl_3$) δ -181.79.
HRMS (ESI/QTOF) m/z: $[M + Na]^+$ Calcd for $C_{19}H_{32}FNNaO_2Si^+$ 376.2079; Found 376.2090.
$[\alpha]_D^{23}$ = –7.7 (c = 0.50 in $CHCl_3$).
HPLC: The enantiomeric excess (93%) was determined *via* HPLC analysis using a CHIRALCEL® OD-H column, with hexane:isopropanol = 98:2 at a flow rate 1.0 mL/min detected at 254 nm wavelength. Retention time: t_{major} = 30.8 min and t_{minor} = 38.3 min. Diastereomeric ratio (97:3) was determined by ^{19}F-NMR of the crude reaction mixture.

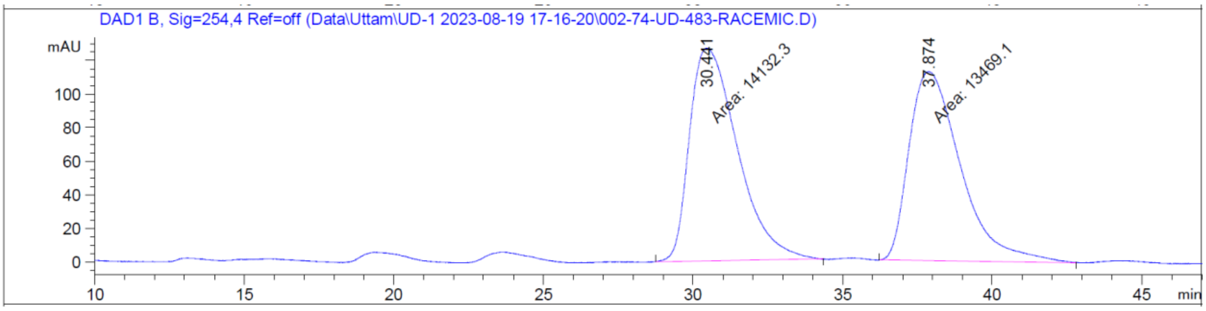

Peak #	RetTime [min]	Type	Width [min]	Area [mAU*s]	Height [mAU]	Area %
1	30.441	MM	1.8598	1.41323e4	126.64668	51.2014
2	37.874	MM	1.9954	1.34691e4	112.50073	48.7986

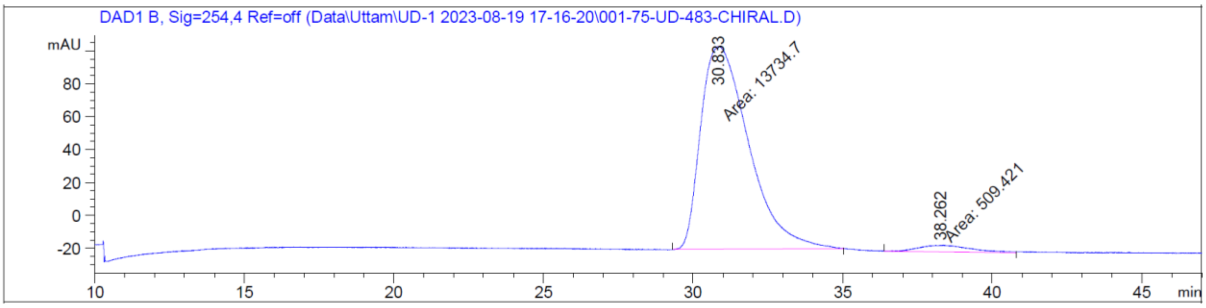

Peak #	RetTime [min]	Type	Width [min]	Area [mAU*s]	Height [mAU]	Area %
1	30.833	MM	1.8560	1.37347e4	123.33327	96.4236
2	38.262	MM	2.0592	509.42142	4.12306	3.5764

(2*S*,3*R*)-6-(1,3-Dioxoisoindolin-2-yl)-3-fluoro-2-methyl-*N*-phenylhexanamide (5eb)

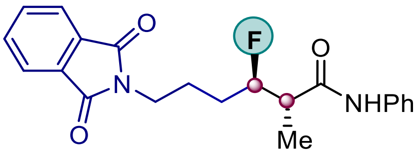

Prepared according to **GP2** with 2-iodo-*N*-phenylpropanamide (**4b**) (27.5 mg, 0.10 mmol, 1.0 equiv.) and (*Z*)-2-(4-fluorobut-3-en-1-yl)isoindoline-1,3-dione (**1e**) (43.8 mg, 0.20 mmol, 2.0 equiv.). Automated flash column chromatography (10 g SiO_2, gradient elution: hexane to 25% EtOAc in hexane) afforded the desired product (-) **5eb** as a yellow oil (21.0 mg, 57%) in >99:1 diastereomeric ratio.

^{1}H NMR (400 MHz, $CDCl_3$) δ 7.83 (dd, *J* = 5.5, 3.0 Hz, 2H), 7.71 (dd, *J* = 5.4, 3.0 Hz, 2H), 7.52 (d, *J* = 8.0 Hz, 2H), 7.42 (s, 1H), 7.31 (t, *J* = 7.8 Hz, 2H), 7.10 (t, *J* = 7.4 Hz, 1H), 4.74 (dtd, *J* = 48.6, 7.8, 3.4 Hz, 1H), 3.74 (t, *J* = 6.9 Hz, 2H), 2.61 (dt, *J* = 16.1, 7.2 Hz, 1H), 1.99 – 1.65 (m, 4H), 1.25 (d, *J* = 7.0 Hz, 3H).

^{13}C NMR (101 MHz, $CDCl_3$): δ 171.20 (d, $^{3}J_{C–F}$ = 1.72 Hz), 168.41, 137.73, 134.02, 132.05, 128.98, 124.41, 123.29, 119.98, 94.80 (d, $^{1}J_{C–F}$ = 171.6 Hz), 47.05 (d, $^{2}J_{C–F}$ = 20.2 Hz), 37.42, 29.66 (d, $^{2}J_{C–F}$ = 21.0 Hz), 24.17 (d, $^{3}J_{C–F}$ = 3.2 Hz), 13.74 (d, $^{3}J_{C–F}$ = 7.1 Hz).

^{19}F{1H} NMR (376 MHz, $CDCl_3$) δ -181.69.

HRMS (ESI/QTOF) m/z: $[M + Na]^+$ Calcd for $C_{21}H_{21}FN_2NaO_3^+$ 391.1428; Found 391.1441.

$[\alpha]_D^{23}$ = –8.7 (c = 0.50 in $CHCl_3$).

HPLC: The enantiomeric excess (95%) was determined *via* HPLC analysis using a CHIRALCEL® OJ-H column, with hexane:isopropanol = 70:30 at a flow rate 1.0 mL/min

detected at 254 nm wavelength. Retention time: t_{major} = 29.0 min and t_{minor} = 19.7 min. Diastereomeric ratio (96:4) was determined by ^{19}F-NMR of the crude reaction mixture.

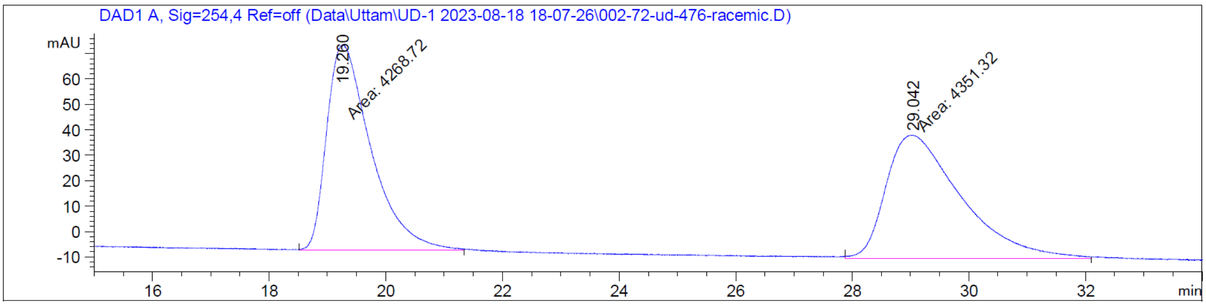

Peak #	RetTime [min]	Type	Width [min]	Area [mAU*s]	Height [mAU]	Area %
1	19.260	MM	0.8786	4268.72217	80.97790	49.5209
2	29.042	MM	1.4923	4351.31592	48.59622	50.4791

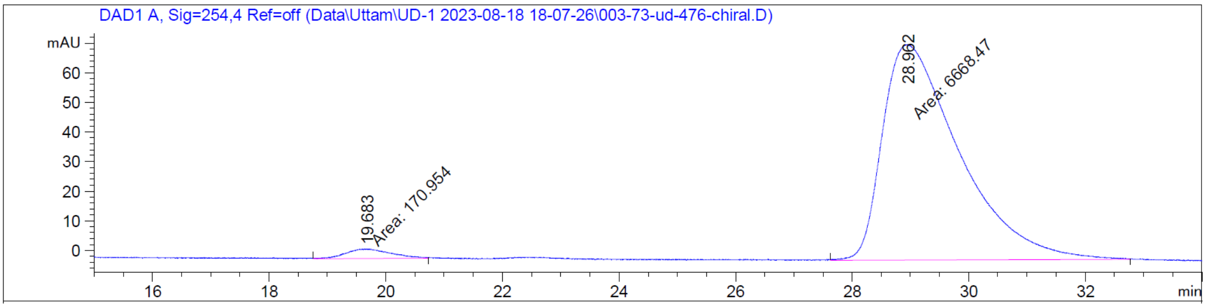

Peak #	RetTime [min]	Type	Width [min]	Area [mAU*s]	Height [mAU]	Area %
1	19.683	MM	0.8934	170.95445	3.18903	2.4995
2	28.962	MM	1.5246	6668.46973	72.89698	97.5005

(2*S*,3*R*)-3-Fluoro-*N*-(4-methoxyphenyl)-2-methyl-6-(5-methylfuran-2-yl)hexanamide (5fa)

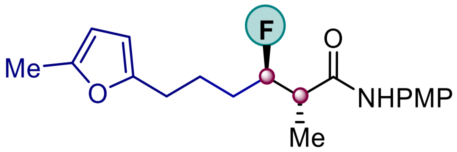

Prepared according to **GP2** with 2-iodo-*N*-(4-methoxyphenyl)propanamide (**4a**) (30.5 mg, 0.10 mmol, 1.0 equiv.) and (*Z*)-2-(4-fluorobut-3-en-1-yl)-5-methylfuran (**1f**) (30.8 mg, 0.20 mmol,

2.0 equiv.). Automated flash column chromatography (10 g SiO_2, gradient elution: hexane to 10% EtOAc in hexane) afforded the desired product (-) **5fa** as a yellow oil (22.5 mg, 68%) in >99:1 diastereomeric ratio.

^{1}H NMR (400 MHz, $CDCl_3$) δ 7.47 – 7.40 (m, 2H), 7.29 (d, J = 8.1 Hz, 1H), 6.91 – 6.80 (m, 2H), 5.90 – 5.82 (m, 2H), 4.71 (dtd, J = 48.3, 7.4, 3.4 Hz, 1H), 3.81 (s, 3H), 2.67 – 2.53 (m, 3H), 2.27 (s, 3H), 1.93 – 1.67 (m, 4H), 1.27 (d, J = 7.1 Hz, 3H).
^{13}C NMR (101 MHz, $CDCl_3$) δ 171.23 (d, $^3J_{C–F}$ = 1.72 Hz), 156.54, 153.57, 150.39, 130.87, 121.90, 114.15, 105.81, 105.73, 95.34 (d, $^1J_{C–F}$ = 170.9 Hz), 55.50, 47.03 (d, $^2J_{C–F}$ = 20.2 Hz), 31.90 (d, $^2J_{C–F}$ = 20.9 Hz), 27.60, 23.51 (d, $^3J_{C–F}$ = 3.8 Hz), 13.84 (d, $^3J_{C–F}$ = 7.0 Hz), 13.48.
^{19}F{1H} NMR (376 MHz, $CDCl_3$) δ -182.12.
HRMS (ESI/QTOF) m/z: $[M + Na]^+$ Calcd for $C_{19}H_{24}FNNaO_3^+$ 356.1632; Found 356.1639.
$[\alpha]_D^{23}$ = –12.9 (c = 1.00 in $CHCl_3$).
HPLC: The enantiomeric excess (98%) was determined *via* HPLC analysis using a CHIRALCEL® IA column, with hexane:isopropanol = 90:10 at a flow rate 1.0 mL/min detected at 254 nm wavelength. Retention time: t_{major} = 18.0 min and t_{minor} = 14.5 min. Diastereomeric ratio (98:2) was determined by ^{19}F-NMR of the crude reaction mixture.

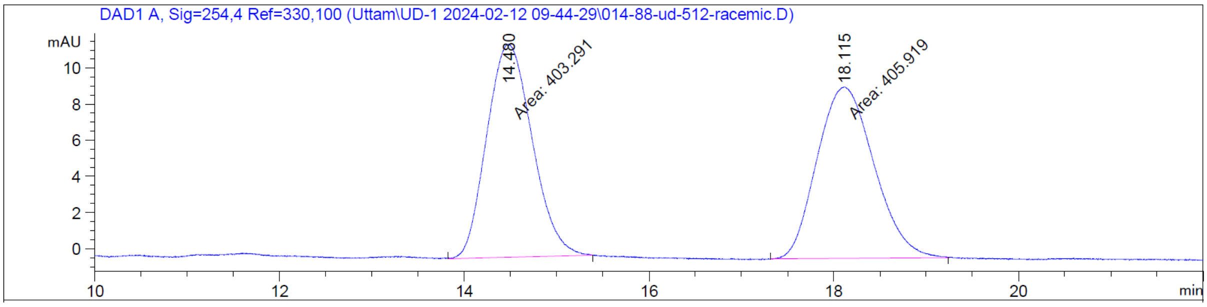

Peak #	RetTime [min]	Type	Width [min]	Area [mAU*s]	Height [mAU]	Area %
1	14.480	MM	0.5694	403.29117	11.80534	49.8376
2	18.115	MM	0.7134	405.91943	9.48298	50.1624

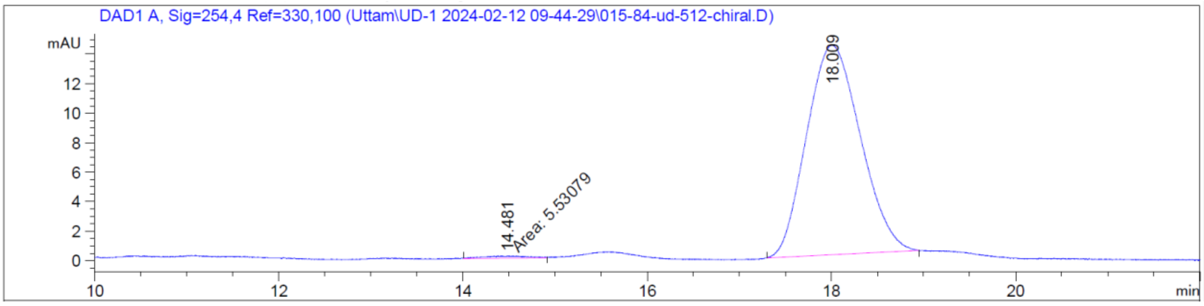

Peak #	RetTime [min]	Type	Width [min]	Area [mAU*s]	Height [mAU]	Area %
1	14.481	MM	0.5081	5.53079	1.81416e-1	0.9462
2	18.009	BB	0.4754	579.01593	14.25369	99.0538

(2*S*,3*R*,6*R*)-3-Fluoro-*N*-(4-methoxyphenyl)-2,6,10-trimethylundec-9-enamide (5ga)

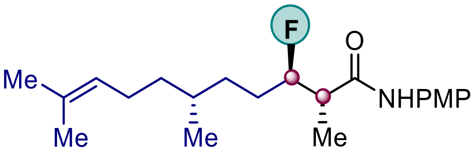

Prepared according to **GP2** with 2-iodo-*N*-(4-methoxyphenyl)propanamide (**4a**) (30.5 mg, 0.10 mmol, 1.0 equiv.) and (*S*,*Z*)-1-fluoro-4,8-dimethylnona-1,7-diene (**1g**) (34.1 mg, 0.20 mmol, 2.0 equiv.). Automated flash column chromatography (10 g SiO_2, gradient elution: hexane to 10% EtOAc in hexane) afforded the desired product (–) **5ga** as a yellow oil (25.1 mg, 72%) in 98:2 diastereomeric ratio.

^{1}H NMR (400 MHz, $CDCl_3$) δ 7.47 – 7.39 (m, 2H), 7.37 (bs, 1H), 6.90 – 6.79 (m, 2H), 5.08 (tdt, *J* = 5.9, 2.9, 1.5 Hz, 1H), 4.64 (dtd, *J* = 48.1, 7.1, 4.6 Hz, 1H), 3.78 (s, 3H), 2.59 (dt, *J* = 17.1, 7.1 Hz, 1H), 1.96 (tt, *J* = 14.8, 7.2 Hz, 2H), 1.74 – 1.64 (m, 5H), 1.60 (s, 3H), 1.50 – 1.38 (m, 2H), 1.37 – 1.30 (m, 2H), 1.25 (d, *J* = 7.1 Hz, 3H), 1.16 (dddd, *J* = 13.3, 9.3, 7.3, 6.1 Hz, 1H), 0.88 (d, *J* = 6.3 Hz, 3H).

^{13}C NMR (101 MHz, $CDCl_3$) δ 171.46 (d, $^3J_{C–F}$ = 1.8 Hz), 156.48, 131.27, 130.92, 124.72, 121.90, 114.13, 95.82 (d, $^1J_{C–F}$ = 170.6 Hz), 55.50, 47.02 (d, $^2J_{C–F}$ = 20.2 Hz), 37.05, 32.11, 31.75 (d, $^3J_{C–F}$ = 2.6 Hz), 30.01 (d, $^2J_{C–F}$ = 20.9 Hz), 25.73, 25.51, 19.31, 17.66, 13.91 (d, $^3J_{C-F}$ = 7.3 Hz).

^{19}F{1H} NMR (376 MHz, $CDCl_3$) δ -181.83.

HRMS (ESI/QTOF) *m/z*: $[M + Na]^+$ Calcd for $C_{21}H_{32}FNNaO_2^+$ 372.2309; Found 372.2315.

$[\alpha]_D^{23}$ = –8.0 (c = 0.67 in $CHCl_3$).

HPLC: The enantiomeric excess (98%) was determined *via* HPLC analysis using a CHIRALCEL® OJ-H column, with hexane:isopropanol = 75:25 at a flow rate 1.0 mL/min detected at 254 nm wavelength. Retention time: t_{major} = 5.3 min and t_{minor} = 4.8 min. Diastereomeric ratio (98:2) was determined by ^{19}F-NMR of the crude reaction mixture.

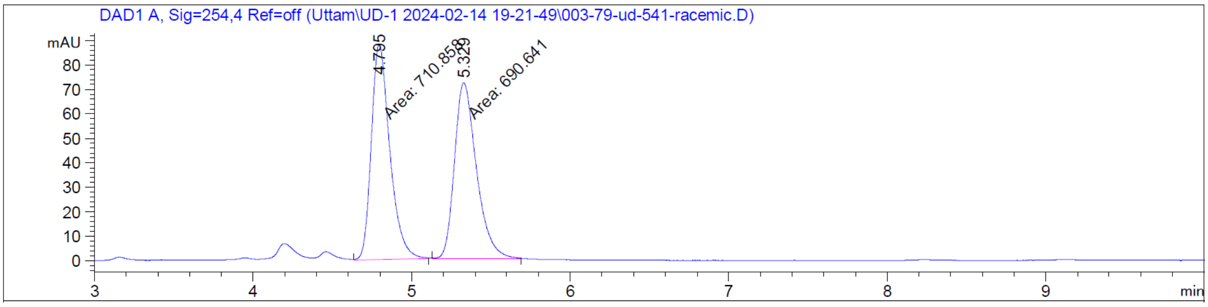

Peak #	RetTime [min]	Type	Width [min]	Area [mAU*s]	Height [mAU]	Area %
1	4.795	MM	0.1348	710.85822	87.86604	50.7213
2	5.329	MM	0.1603	690.64050	71.80668	49.2787

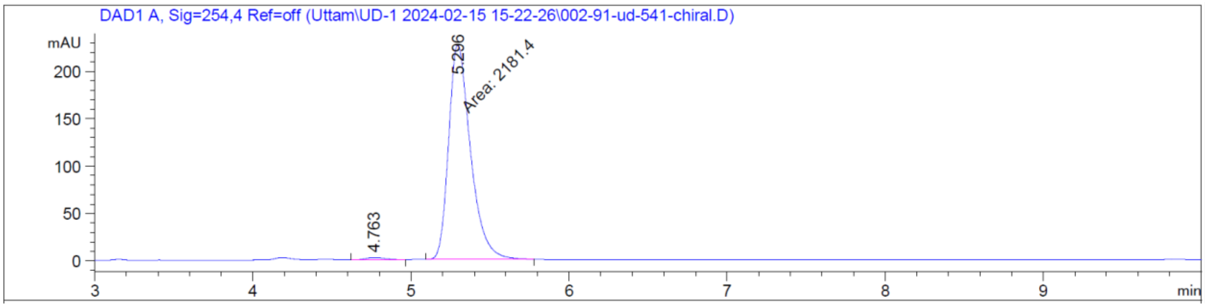

Peak #	RetTime [min]	Type	Width [min]	Area [mAU*s]	Height [mAU]	Area %
1	4.763	BB	0.0970	19.81351	2.42160	0.9001
2	5.296	MM	0.1602	2181.40454	226.89662	99.0999

(*S*)-2-{(*R*)-1-Fluoro-4-phenylbutyl}-*N*-(4-methoxyphenyl)heptanamide (5ac)

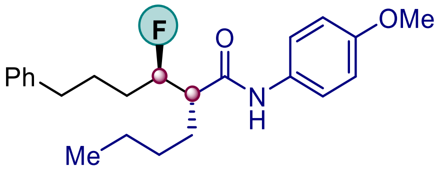

A modified procedure **GP2** was followed with 2-bromo-*N*-(4-methoxyphenyl)hexanamide (**4c**) (30.0 mg, 0.10 mmol, 1.0 equiv.), (*Z*)-(4-fluorobut-3-en-1-yl)benzene (**1a**) (30.0 mg, 0.20 mmol, 2.0 equiv.), $NiCl_2$·DME (3.3 mg, 0.015 mmol, 0.15 equiv.), ligand **L8** (0.023 mmol, 0.23 equiv.) and KI (16.6 mg, 0.1 mmol, 1 equiv.). Automated flash column chromatography (10 g SiO_2, gradient elution: hexane to 10% EtOAc in hexane) afforded the desired product (+) **5ac** as a yellow oil (15.0 mg, 40%) in >99:1 diastereomeric ratio.

^{1}H NMR (400 MHz, CDCl3) δ 7.44 – 7.37 (m, 2H), 7.31 – 7.24 (m, 3H), 7.20 – 7.14 (m, 3H), 6.89 – 6.83 (m, 2H), 4.83 – 4.55 (m, 1H), 3.79 (s, 3H), 2.65 (dq, *J* = 13.8, 6.5 Hz, 2H), 2.41 (ddt, *J* = 21.0, 10.5, 5.4 Hz, 1H), 1.77 (dtt, *J* = 24.2, 8.9, 5.3 Hz, 5H), 1.55 – 1.45 (m, 1H), 1.39 – 1.30 (m, 4H), 0.93 – 0.84 (m, 3H).

^{13}C NMR (101 MHz, $CDCl_3$) δ 170.69 (d, $^3J_{C–F}$ = 1.6 Hz), 156.53, 141.79, 130.82, 128.40, 125.92, 121.95, 114.15, 94.74 (d, $^1J_{C–F}$ = 170.9 Hz), 55.53, 53.60 (d, $^2J_{C–F}$ = 19.4 Hz), 35.43, 32.35 (d, $^2J_{C–F}$ = 20.9 Hz), 31.47, 29.47, 28.81 (d, $^3J_{C–F}$ = 5.5 Hz), 26.79 (d, $^3J_{C–F}$ = 3.1 Hz), 22.65, 13.89.

^{19}F{1H} NMR (376 MHz, $CDCl_3$) δ -183.98.

HRMS (APCI/QTOF) m/z: $[M + Na]^+$ Calcd for $C_{23}H_{30}FNNaO_2^+$ 394.2153; Found 394.2149.

$[\alpha]_D^{23}$ = +1.8 (c = 0.45 in $CHCl_3$).

HPLC: The enantiomeric excess (92%) was determined *via* HPLC analysis using a CHIRALCEL® IA column, with hexane:isopropanol = 95:15 at a flow rate 1.0 mL/min detected at 254 nm wavelength. Retention time: t_{major} = 53.6 min and t_{minor} = 35.6 min. Diastereomeric ratio (94:6) was determined by GC-MS analysis of the crude reaction mixture. Minor amount of other regio-isomer was observed in the crude reaction mixture.

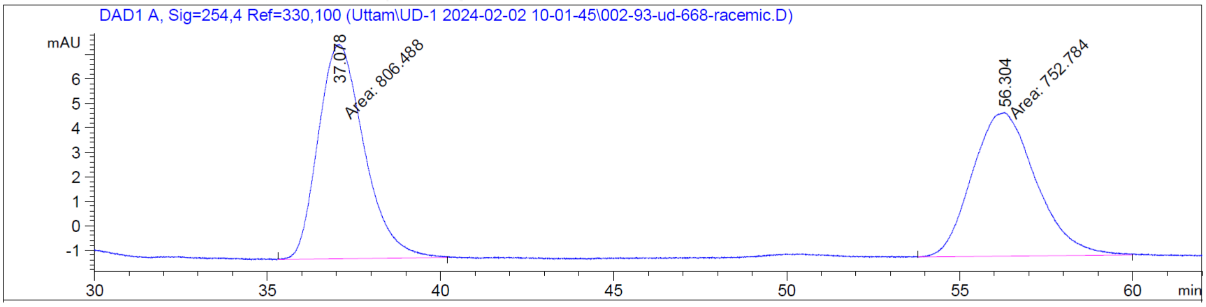

Peak #	RetTime [min]	Type	Width [min]	Area [mAU*s]	Height [mAU]	Area %
1	37.078	MM	1.5333	806.48792	8.76654	51.7221
2	56.304	MM	2.1329	752.78400	5.88230	48.2779

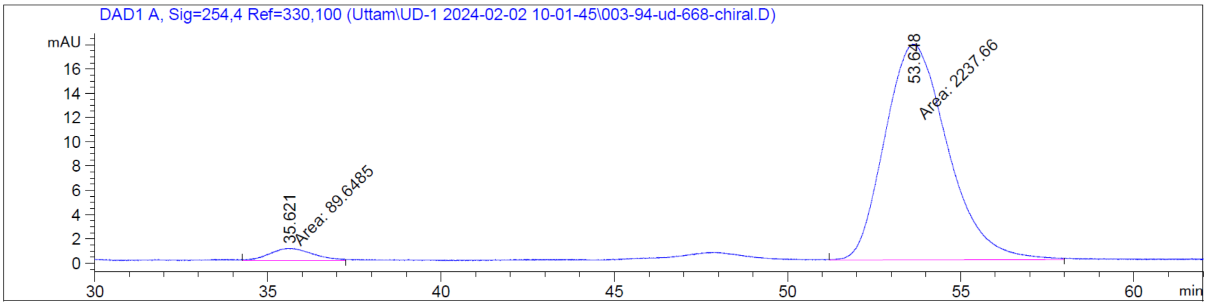

Peak #	RetTime [min]	Type	Width [min]	Area [mAU*s]	Height [mAU]	Area %
1	35.621	MM	1.4461	89.64845	1.03325	3.8520
2	53.648	MM	2.0972	2237.65967	17.78298	96.1480

(4*S*,5*R*)-Methyl -5-fluoro-8-phenyl-4-(phenylcarbamoyl)octanoate (5ad)

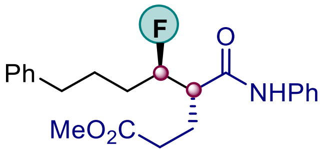

A modified procedure **GP2** was followed with methyl 4-iodo-5-oxo-5-(phenylamino)pentanoate (**4d**) (34.7 mg, 0.10 mmol, 1.0 equiv.) and (*Z*)-(4-fluorobut-3-en-1-yl)benzene (**1a**) (30.0 mg, 0.20 mmol, 2.0 equiv.), $NiCl_2$·DME (3.3 mg, 0.015 mmol, 0.15 equiv.) and ligand **L8** (0.023 mmol, 0.23 equiv.). Automated flash column chromatography (10

g SiO_2, gradient elution: hexane to 50% EtOAc in hexane) afforded the desired product (+) **5ad** as a yellow oil (17.5 mg, 47%) in >99:1 diastereomeric ratio.

^{1}H NMR (400 MHz, $CDCl_3$) δ 7.52 (d, J = 7.8 Hz, 3H), 7.31 (dd, J = 17.9, 10.1 Hz, 4H), 7.15 (dt, J = 24.6, 7.3 Hz, 4H), 4.88 – 4.58 (m, 1H), 3.68 (s, 3H), 2.72 – 2.32 (m, 5H), 2.11 – 1.95 (m, 1H), 1.94 – 1.70 (m, 5H).

^{13}C NMR (101 MHz, $CDCl_3$) δ 173.46, 170.04 (d, $^{3}J_{C–F}$ = 1.7 Hz), 141.70, 137.55, 129.03, 128.47, 128.41, 125.95, 124.57, 120.02, 94.52 (d, $^{1}J_{C–F}$ = 171.3 Hz), 52.15 (d, $^{2}J_{C–F}$ = 20.2 Hz), 51.84, 35.40, 32.13 (d, $^{2}J_{C–F}$ = 20.9 Hz), 31.25, 26.61 (d, $^{3}J_{C–F}$ = 3.7 Hz), 24.04 (d, $^{3}J_{C–F}$ = 6.6 Hz).

^{19}F{1H} NMR (376 MHz, $CDCl_3$) δ -183.00.

HRMS (APCI/QTOF) m/z: [M + Na]$^+$ Calcd for $C_{22}H_{26}FNNaO_3^+$ 394.1789; Found 394.1784.

$[\alpha]_D^{23}$ = +12.0 (c = 0.50 in $CHCl_3$).

HPLC: The enantiomeric excess (91%) was determined *via* HPLC analysis using a CHIRALCEL® OJ-H column, with hexane:isopropanol = 70:30 at a flow rate 1.0 mL/min detected at 254 nm wavelength. Retention time: t_{major} = 21.3 min and t_{minor} = 16.2 min. Diastereomeric ratio (91:9) was determined by ^{19}F-NMR of the crude reaction mixture.

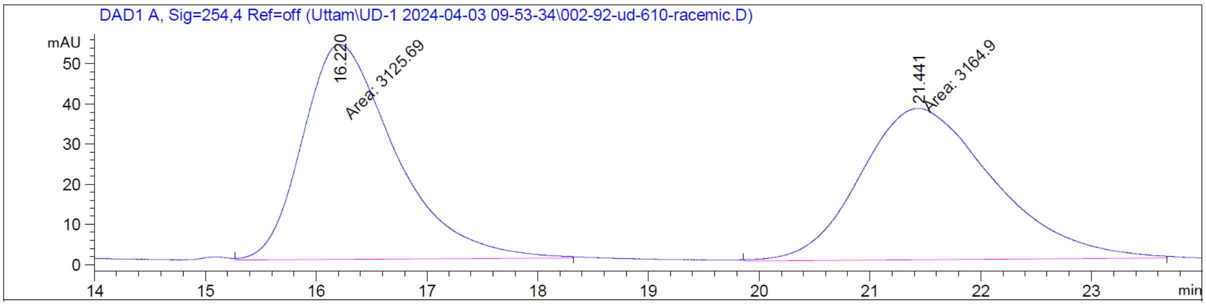

Peak #	RetTime [min]	Type	Width [min]	Area [mAU*s]	Height [mAU]	Area %
1	16.220	MM	0.9727	3125.69019	53.55649	49.6883
2	21.441	MM	1.3982	3164.90161	37.72536	50.3117

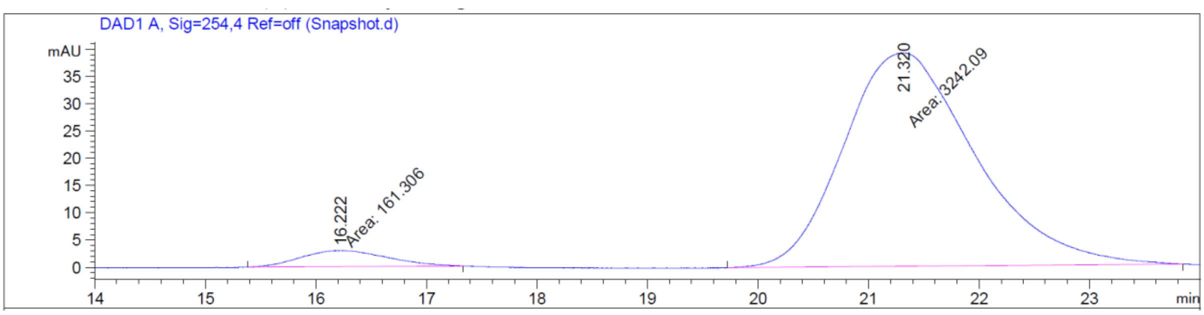

Peak #	RetTime [min]	Type	Width [min]	Area [mAU*s]	Height [mAU]	Area %
1	16.222	MM	0.9018	161.30641	2.98127	4.7396
2	21.320	MM	1.3831	3242.08960	39.06833	95.2604

(2*S*,3*R*)-3-fluoro-2-methyl-6-phenyl-*N*-(thiophen-3-yl)hexanamide (5ae)

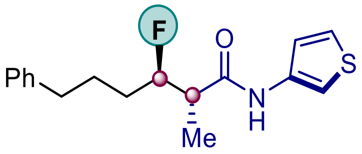

Prepared according to **GP2** with 2-iodo-*N*-(thiophen-3-yl)propanamide (**4e**) (28.1 mg, 0.10 mmol, 1.0 equiv.) and (*Z*)-(4-fluorobut-3-en-1-yl)benzene (**1a**) (30.0 mg, 0.20 mmol, 2.0 equiv.). Automated flash column chromatography (10 g SiO_2, gradient elution: hexane to 10% EtOAc in hexane) afforded the desired product (-) **5ae** as a yellow oil (20.0 mg, 66%) in >99:1 diastereomeric ratio.

^{1}H NMR (400 MHz, $CDCl_3$) δ 7.83 – 7.65 (m, 1H), 7.61 (dd, *J* = 3.2, 1.4 Hz, 1H), 7.33 – 7.28 (m, 2H), 7.27 – 7.17 (m, 4H), 7.01 (dd, *J* = 5.2, 1.4 Hz, 1H), 4.70 (dtd, *J* = 48.5, 7.7, 3.7 Hz, 1H), 2.74 – 2.50 (m, 3H), 1.92 – 1.66 (m, 4H), 1.25 (d, *J* = 7.1 Hz, 3H).

^{13}C NMR (101 MHz, $CDCl_3$) (one resonance is missing due to overlap): δ 170.74 (d, $^3J_{C–F}$ = 1.7 Hz), 141.74, 135.34, 128.41, 125.95, 124.50, 121.07, 110.49, 95.36 (d, $^1J_{C–F}$ = 170.8 Hz), 46.69 (d, $^2J_{C–F}$ = 20.2 Hz), 35.41, 32.02 (d, $^2J_{C–F}$ = 21.0 Hz), 26.54 (d, $^3J_{C–F}$ = 3.6 Hz), 13.86 (d, $^3J_{C–F}$ = 7.1 Hz).

^{19}F{1H} NMR (376 MHz, $CDCl_3$) δ -181.91.

HRMS (ESI/QTOF) m/z: [M + Na]$^+$ Calcd for $C_{17}H_{20}FNOSNa^+$ 328.1142; Found 328.1155.

$[\alpha]_D^{23}$ = –8.4 (c = 0.50 in $CHCl_3$).

HPLC: The enantiomeric excess (98%) was determined *via* HPLC analysis using a CHIRALCEL® OD-H column, with hexane:isopropanol = 90:10 at a flow rate 1.0 mL/min

detected at 214 nm wavelength. Retention time: t_{major} = 24.5 min and t_{minor} = 17.9 min. Diastereomeric ratio (98:2) was determined by ^{19}F-NMR of the crude reaction mixture.

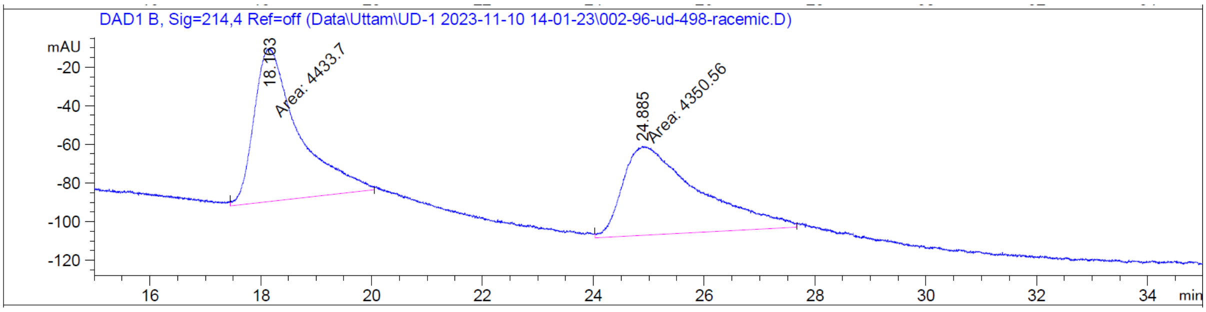

Peak #	RetTime [min]	Type	Width [min]	Area [mAU*s]	Height [mAU]	Area %
1	18.163	MM	0.9312	4433.69629	79.35439	50.4732
2	24.885	MM	1.5700	4350.56152	46.18338	49.5268

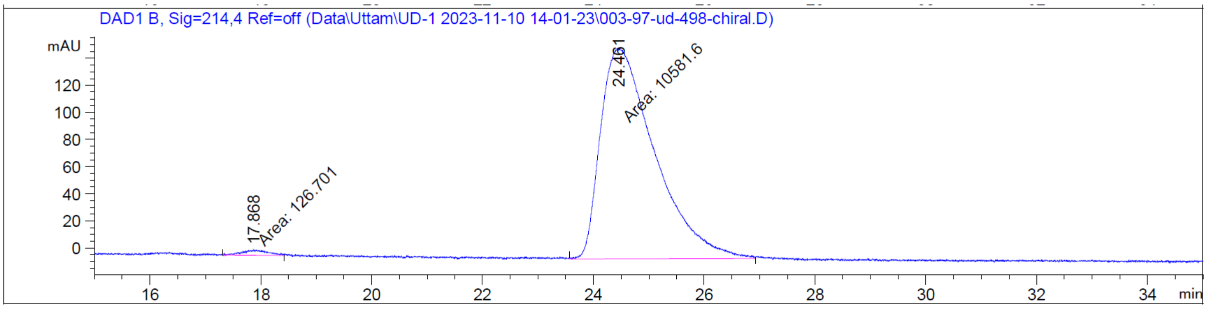

Peak #	RetTime [min]	Type	Width [min]	Area [mAU*s]	Height [mAU]	Area %
1	17.868	MM	0.4557	126.70068	4.63412	1.1832
2	24.461	MM	1.1330	1.05816e4	155.66141	98.8168

(2*S*,3*R*)-*N*-(*tert*-Butyl)-3-fluoro-2-methyl-6-phenylhexanamide (5af)

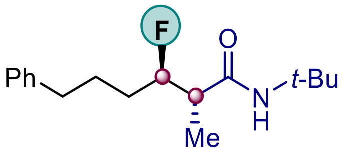

Prepared according to **GP2** with *N*-(*tert*-butyl)-2-iodopropanamide (**4f**) (25.5 mg, 0.10 mmol, 1.0 equiv.) and (*Z*)-(4-fluorobut-3-en-1-yl)benzene (**1a**) (30.0 mg, 0.20 mmol, 2.0 equiv.). Automated flash column chromatography (10 g SiO_2, gradient elution: hexane to 10% EtOAc

in hexane) afforded the desired product (-) **5af** as a yellow oil (16.7 mg, 60%) in >99:1 diastereomeric ratio.

^{1}H NMR (400 MHz, $CDCl_3$) δ 7.33 – 7.26 (m, 2H), 7.18 (td, J = 5.9, 1.7 Hz, 3H), 5.40 (s, 1H), 4.59 (dtd, J = 48.4, 8.2, 3.4 Hz, 1H), 2.66 (dtt, J = 21.4, 14.1, 7.1 Hz, 2H), 2.43 – 2.20 (m, 1H), 1.91 – 1.59 (m, 4H), 1.34 (s, 9H), 1.09 (d, J = 7.0 Hz, 3H).

^{13}C NMR (101 MHz, $CDCl_3$) δ 172.55 (d, $^{3}J_{C\text{–}F}$ = 2.6 Hz), 141.88, 128.41, 128.37, 125.87, 95.33 (d, $^{1}J_{C\text{–}F}$ = 170.2 Hz), 51.23, 47.02 (d, $^{2}J_{C\text{–}F}$ = 20.5 Hz), 35.45, 31.84 (d, $^{2}J_{C\text{–}F}$ = 20.9 Hz), 28.76, 26.56 (d, $^{3}J_{C\text{–}F}$ = 3.3 Hz), 13.76 (d, $^{3}J_{C\text{–}F}$ = 7.7 Hz).

^{19}F{1H} NMR (376 MHz, $CDCl_3$) δ -182.06.

HRMS (ESI/QTOF) m/z: $[M + Na]^+$ Calcd for $C_{17}H_{26}FNNaO^+$ 302.1891; Found 302.1895.

$[\alpha]_D^{23}$ = –0.8 (c = 0.70 in $CHCl_3$).

HPLC: The enantiomeric excess (97%) was determined *via* HPLC analysis using a CHIRALCEL® OD-H column, with hexane:isopropanol = 98:2 at a flow rate 1.0 mL/min detected at 210 nm wavelength. Retention time: t_{major} = 16.6 min and t_{minor} = 12.8 min. Diastereomeric ratio (91:9) was determined by ^{19}F-NMR of the crude reaction mixture.

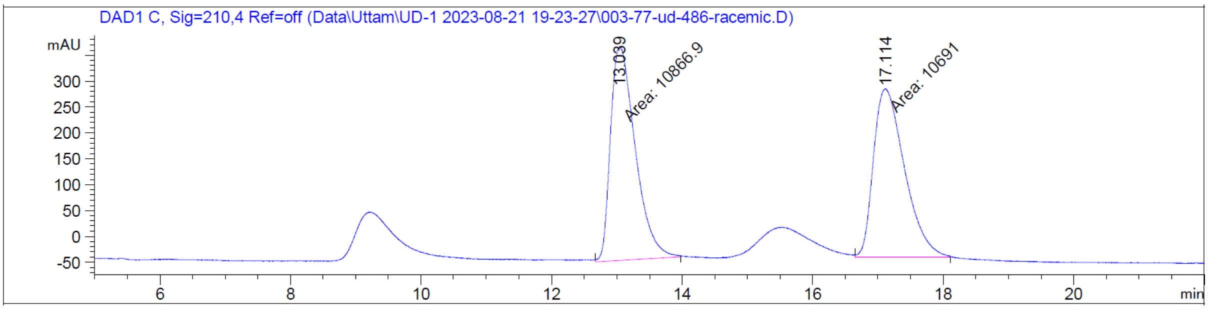

Peak #	RetTime [min]	Type	Width [min]	Area [mAU*s]	Height [mAU]	Area %
1	13.039	MM	0.4387	1.08669e4	412.86804	50.4079
2	17.114	MM	0.5484	1.06910e4	324.90625	49.5921

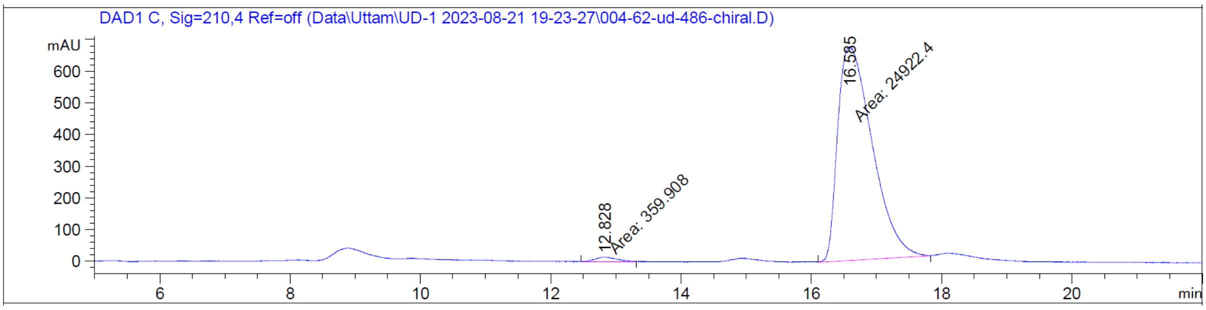

Peak #	RetTime [min]	Type	Width [min]	Area [mAU*s]	Height [mAU]	Area %
1	12.828	MM	0.3916	359.90823	15.31708	1.4236
2	16.585	MM	0.6152	2.49224e4	675.20197	98.5764

(2*S*,3*R*)-*N*-(4-Bromophenyl)-3-fluoro-2-methyl-6-phenylhexanamide (5ag)

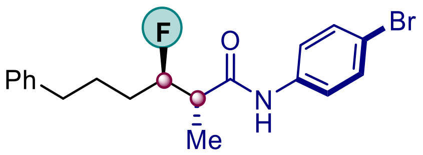

Prepared according to **GP2** with *N*-(4-bromophenyl)-2-iodopropanamide (**4g**) (35.4 mg, 0.10 mmol, 1.0 equiv.) and (*Z*)-(4-fluorobut-3-en-1-yl)benzene (**1a**) (30.0 mg, 0.20 mmol, 2.0 equiv.). Automated flash column chromatography (10 g SiO_2, gradient elution: hexane to 10% EtOAc in hexane) afforded the desired product (-) **5ag** as a yellow oil (23 mg, 61%) in >99:1 diastereomeric ratio.

^{1}H NMR (400 MHz, $CDCl_3$) δ 7.42 (bs, 5H), 7.31 – 7.23 (m, 2H), 7.23 – 7.07 (m, 3H), 4.67 (dtd, *J* = 48.5, 7.7, 3.5 Hz, 1H), 2.86 – 2.42 (m, 3H), 1.96 – 1.64 (m, 4H), 1.22 (d, *J* = 7.1 Hz, 3H).

^{13}C NMR (101 MHz, $CDCl_3$) δ 171.57 (d, $^{3}J_{C–F}$ = 1.2 Hz), 141.69, 136.80, 131.95, 128.43, 128.40, 125.98, 121.57, 116.99, 95.40 (d, $^{1}J_{C–F}$ = 170.6 Hz), 47.23 (d, $^{2}J_{C–F}$ = 19.8 Hz), 35.40, 32.03 (d, $^{2}J_{C–F}$ = 20.9 Hz), 26.49 (d, $^{3}J_{C–F}$ = 3.3 Hz), 13.81 (d, $^{3}J_{C–F}$ = 7.3 Hz).

^{19}F{1H} NMR (376 MHz, $CDCl_3$) δ -181.69.

HRMS (ESI/QTOF) m/z: $[M + Na]^+$ Calcd for $C_{19}H_{21}{}^{79}BrFNNaO^+$ 400.0683; Found 400.0698.

$[\alpha]_D^{23}$ = –11.6 (c = 0.45 in $CHCl_3$).

HPLC: The enantiomeric excess (96%) was determined *via* HPLC analysis using a CHIRALCEL® OJ-H column, with hexane:isopropanol = 75:25 at a flow rate 1.0 mL/min detected at 254 nm wavelength. Retention time: t_{major} = 10.3 min and t_{minor} = 12.0 min. Diastereomeric ratio (98:2) was determined by ^{19}F-NMR of the crude reaction mixture.

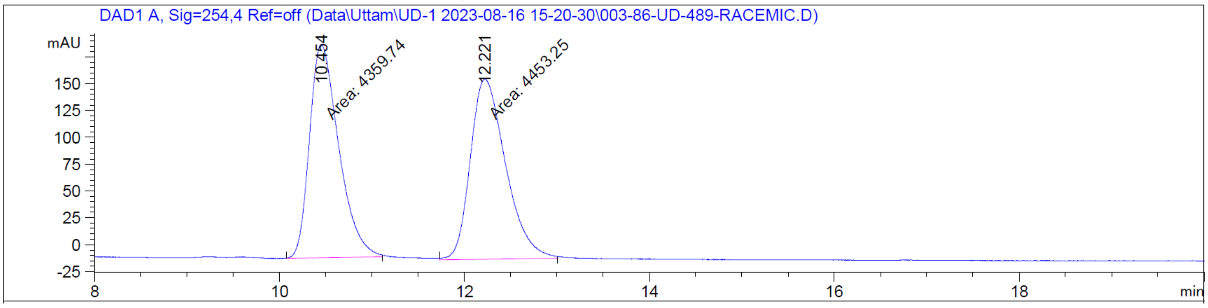

Peak #	RetTime [min]	Type	Width [min]	Area [mAU*s]	Height [mAU]	Area %
1	10.454	MM	0.3657	4359.74268	198.68976	49.4695
2	12.221	MM	0.4419	4453.24707	167.95399	50.5305

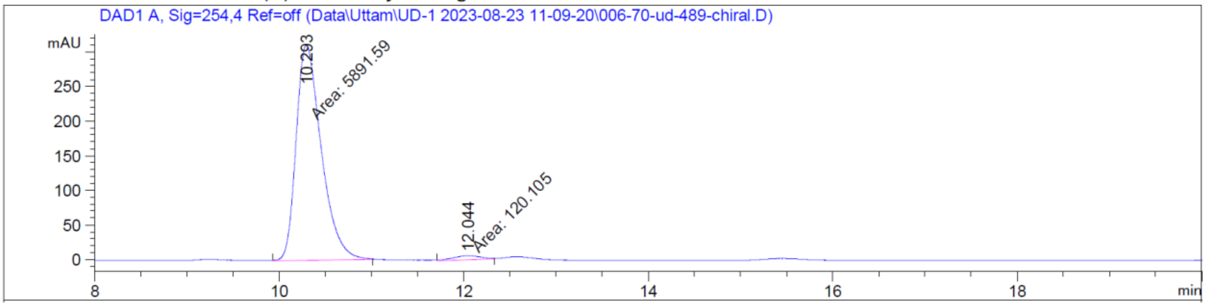

Peak #	RetTime [min]	Type	Width [min]	Area [mAU*s]	Height [mAU]	Area %
1	10.293	MM	0.3156	5891.59033	311.09207	98.0022
2	12.044	MM	0.3442	120.10451	5.81597	1.9978

(2*S*,3*R*)-*N*-(4-iodophenyl)-3-fluoro-2-methyl-6-phenylhexanamide (5ah)

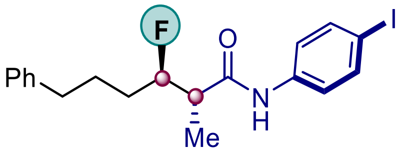

Prepared according to **GP2** with *N*-(4-iodophenyl)-2-iodopropanamide (**4h**) (40.1 mg, 0.10 mmol, 1.0 equiv.) and (*Z*)-(4-fluorobut-3-en-1-yl)benzene (**1a**) (30.0 mg, 0.20 mmol, 2.0 equiv.). Automated flash column chromatography (10 g SiO_2, gradient elution: hexane to 10% EtOAc in hexane) afforded the desired product (-) **5ah** as a yellow oil (27 mg, 64%) in >99:1 diastereomeric ratio.

^{1}H NMR (400 MHz, $CDCl_3$) δ 7.72 – 7.52 (m, 2H), 7.38 (bs, 1H), 7.35 – 7.26 (m, 4H), 7.22 – 7.10 (m, 3H), 4.67 (dtd, J = 48.5, 7.6, 3.6 Hz, 1H), 2.62 (ddt, J = 39.8, 17.1, 7.5 Hz, 3H), 1.91 – 1.63 (m, 4H), 1.22 (d, J = 7.1 Hz, 3H).

^{13}C NMR (101 MHz, $CDCl_3$) δ 171.56 (d, $^{3}J_{C–F}$ = 1.2 Hz), 141.68, 137.91, 137.51, 128.42, 128.39, 125.97, 121.83, 95.40 (d, $^{1}J_{C–F}$ = 170.6 Hz), 87.56, 47.26 (d, $^{2}J_{C–F}$ = 20.2 Hz), 35.39, 32.03 (d, $^{2}J_{C–F}$ = 20.9 Hz), 26.48 (d, $^{3}J_{C–F}$ = 3.3 Hz), 13.80 (d, $^{3}J_{C–F}$ = 7.3 Hz).

^{19}F{1H} NMR (376 MHz, $CDCl_3$) δ -181.73.

HRMS (ESI/QTOF) *m/z*: $[M + Na]^+$ Calcd for $C_{19}H_{21}F^{127}INNaO^+$ 448.0544; Found 448.0559.

$[\alpha]_D^{23}$ = –10.1 (c = 0.83 in $CHCl_3$).

HPLC: The enantiomeric excess (96%) was determined *via* HPLC analysis using a CHIRALCEL® OD-H column, with hexane:isopropanol = 90:10 at a flow rate 1.0 mL/min detected at 254 nm wavelength. Retention time: t_{major} = 17.4 min and t_{minor} = 13.6 min. Diastereomeric ratio (98:2) was determined by ^{19}F-NMR of the crude reaction mixture.

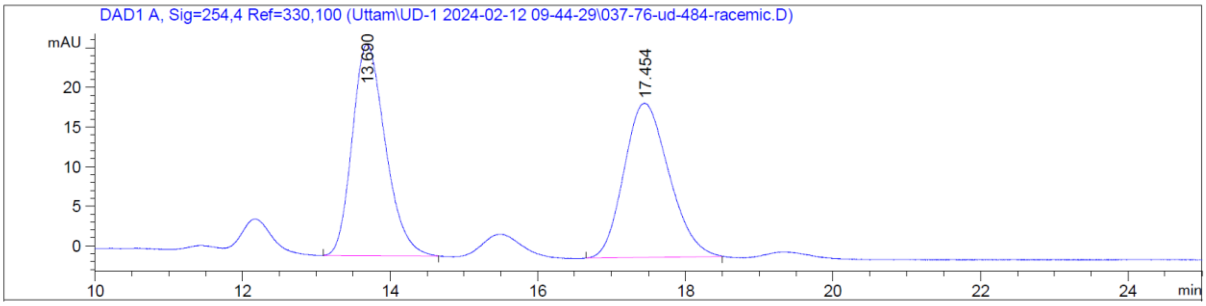

Peak #	RetTime [min]	Type	Width [min]	Area [mAU*s]	Height [mAU]	Area %
1	13.690	BB	0.3662	833.09576	26.68977	50.2591
2	17.454	BB	0.4965	824.50739	19.47155	49.7409

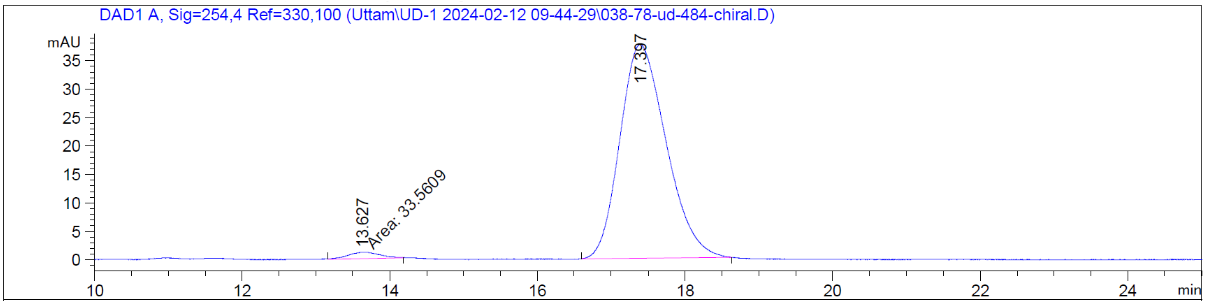

Peak #	RetTime [min]	Type	Width [min]	Area [mAU*s]	Height [mAU]	Area %
1	13.627	MM	0.4901	33.56089	1.14138	2.0132
2	17.397	BB	0.5092	1633.44275	37.57296	97.9868

Methyl 4-((2*S*,3*R*)-3-fluoro-2-methyl-6-phenylhexanamido)benzoate (5ai)

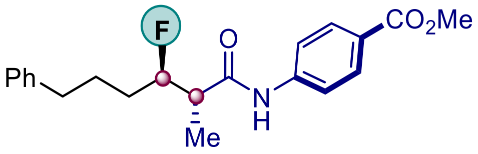

Prepared according to **GP2** with methyl 4-(2-iodopropanamido)benzoate (**4i**) (33.3 mg, 0.10 mmol, 1.0 equiv.) and (*Z*)-(4-fluorobut-3-en-1-yl)benzene (**1a**) (30.0 mg, 0.20 mmol, 2.0 equiv.). Automated flash column chromatography (10 g SiO_2, gradient elution: hexane to 40% EtOAc in hexane) afforded the desired product (-) **5ai** as a colourless oil (24.3 mg, 68%) in >99:1 diastereomeric ratio.

^{1}H NMR (400 MHz, $CDCl_3$) δ 7.74 – 7.59 (m, 2H), 7.37 (bs, 1H), 7.27 (d, *J* = 8.8 Hz, 2H), 6.94 (dd, *J* = 8.0, 6.7 Hz, 2H), 6.84 (ddd, *J* = 8.5, 7.0, 1.6 Hz, 3H), 4.35 (dtd, *J* = 48.5, 7.8, 3.4 Hz, 1H), 3.56 (s, 3H), 2.30 (ddt, *J* = 21.3, 16.6, 7.5 Hz, 3H), 1.62 – 1.30 (m, 4H), 0.90 (d, *J* = 7.1 Hz, 3H).

^{13}C NMR (101 MHz, $CDCl_3$) δ 171.85 (d, $^{3}J_{C–F}$ = 1.2 Hz), 166.62, 141.94, 141.67, 130.83, 128.42, 128.39, 125.97, 125.72, 119.04, 95.37 (d, $^{1}J_{C–F}$ = 170.8 Hz), 52.05, 47.34 (d, $^{2}J_{C–F}$ = 19.8 Hz), 35.38, 32.02 (d, $^{2}J_{C–F}$ = 21.0 Hz), 26.46 (d, $^{3}J_{C–F}$ = 3.2 Hz), 13.79 (d, $^{3}J_{C–F}$ = 7.1 Hz).

^{19}F{1H} NMR (376 MHz, CDCl3) δ -181.47.

HRMS (ESI/QTOF) m/z: $[M + Na]^+$ Calcd for $C_{21}H_{24}FNNaO_3^+$ 380.1632; Found 380.1640.

$[\alpha]_D^{23}$ = –12.8 (c = 0.73 in $CHCl_3$).

HPLC: The enantiomeric excess (94%) was determined *via* HPLC analysis using a CHIRALCEL® OJ-H column, with hexane:isopropanol = 70:30 at a flow rate 1.0 mL/min detected at 254 nm wavelength. Retention time: t_{major} = 12.1 min and t_{minor} = 24.8 min. Diastereomeric ratio (98:2) was determined by ^{19}F-NMR of the crude reaction mixture.

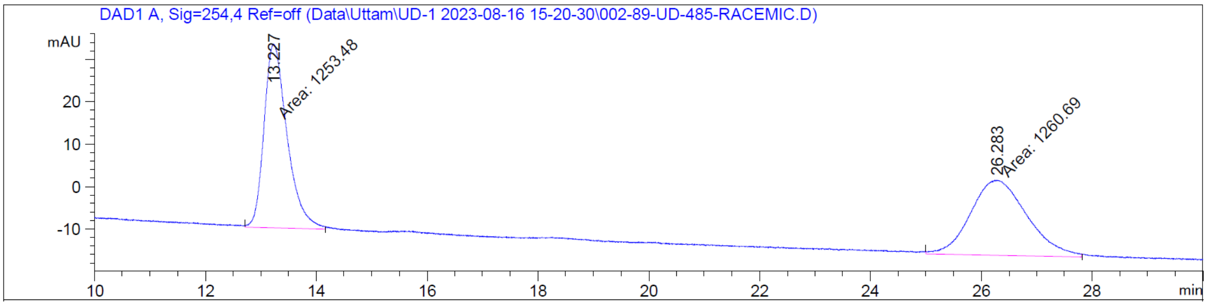

Peak #	RetTime [min]	Type	Width [min]	Area [mAU*s]	Height [mAU]	Area %
1	13.227	MM	0.4815	1253.48340	43.38865	49.8567
2	26.283	MM	1.1815	1260.68665	17.78431	50.1433

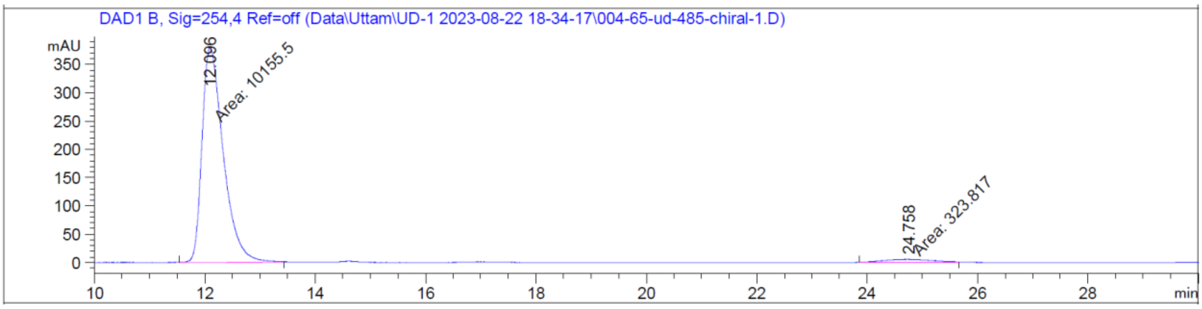

Peak #	RetTime [min]	Type	Width [min]	Area [mAU*s]	Height [mAU]	Area %
1	12.096	MM	0.4457	1.01555e4	379.72580	96.9099
2	24.758	MM	0.9847	323.81693	5.48083	3.0901

(2*S*,3*R*)-3-Fluoro-2-methyl-6-phenyl-*N*-(4-(4,4,5,5-tetramethyl-1,3,2-dioxaborolan-2-yl)phenyl)hexanamide (5aj)

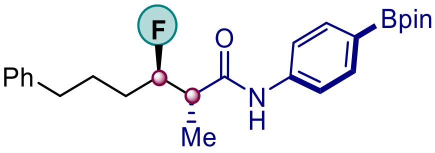

Prepared according to **GP2** with 2-bromo-*N*-(4-(4,4,5,5-tetramethyl-1,3,2-dioxaborolan-2-yl)phenyl)propanamide (**4j**) (35.4 mg, 0.10 mmol, 1.0 equiv.) and (*Z*)-(4-fluorobut-3-en-1-yl)benzene (**1a**) (30.0 mg, 0.20 mmol, 2.0 equiv.). Automated flash column chromatography (10 g SiO_2, gradient elution: hexane to 15% EtOAc in hexane) afforded the desired product (-) **5aj** as a colourless oil (21.2 mg, 50%) in >99:1 diastereomeric ratio.

^{1}H NMR (400 MHz, $CDCl_3$) δ 7.81 – 7.72 (m, 2H), 7.58 – 7.47 (m, 2H), 7.43 (bs, 1H), 7.32 – 7.26 (m, 2H), 7.18 (td, J = 6.9, 1.4 Hz, 3H), 4.68 (dtd, J = 48.3, 7.5, 3.8 Hz, 1H), 2.70 – 2.53 (m, 3H), 1.88 – 1.66 (m, 4H), 1.34 (s, 12H), 1.24 (d, J = 7.0 Hz, 3H).

^{13}C NMR (101 MHz, $CDCl_3$) (two resonances are missing due to overlap) δ 171.51 (d, $^3J_{C–F}$ = 1.2 Hz), 141.74, 140.40, 135.81, 128.41, 125.94, 118.76, 95.39 (d, $^1J_{C–F}$ = 170.6 Hz), 83.76, 47.36 (d, $^2J_{C–F}$ = 20.2 Hz), 35.41, 32.02 (d, $^2J_{C–F}$ = 20.9 Hz), 26.54 (d, $^3J_{C–F}$ = 3.7 Hz), 24.88, 13.81 (d, $^3J_{C–F}$ = 7.0 Hz).

^{19}F{1H} NMR (376 MHz, $CDCl_3$) δ -181.95.

HRMS (ESI/QTOF) m/z: $[M + Na]^+$ Calcd for $C_{25}H_{33}BFNNaO_3^+$ 448.2434; Found 448.2447.

$[\alpha]_D^{23}$ = –7.2 (c = 0.50 in $CHCl_3$).

HPLC: The enantiomeric excess (93%) was determined *via* HPLC analysis using a CHIRALCEL® IA column, with hexane:isopropanol = 95:5 at a flow rate 1.0 mL/min detected at 254 nm wavelength. Retention time: t_{major} = 16.6 min and t_{minor} = 13.9 min. Diastereomeric ratio (97:3) was determined by ^{19}F-NMR of the crude reaction mixture.

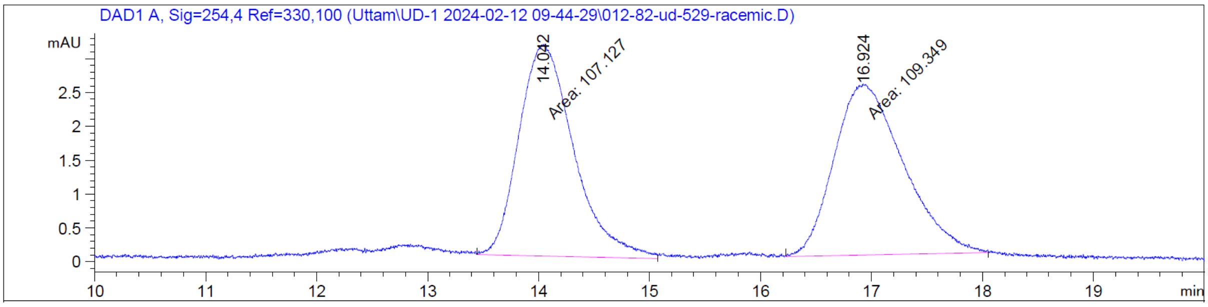

Peak #	RetTime [min]	Type	Width [min]	Area [mAU*s]	Height [mAU]	Area %
1	14.042	MM	0.5716	107.12715	3.12370	49.4868
2	16.924	MM	0.7217	109.34914	2.52541	50.5132

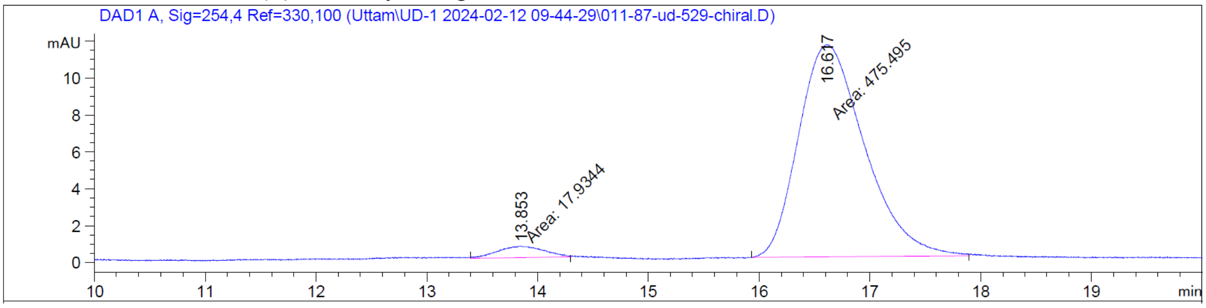

Peak #	RetTime [min]	Type	Width [min]	Area [mAU*s]	Height [mAU]	Area %
1	13.853	MM	0.4757	17.93443	6.28395e-1	3.6347
2	16.617	MM	0.6897	475.49475	11.48999	96.3653

(2*S*,3*R*)-3-Fluoro-2-methyl-6-phenylhexanamide (5ak)

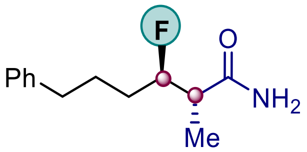

A modified procedure **GP2** was followed with 2-bromopropanamide (**4k**) (15.2 mg, 0.10 mmol, 1.0 equiv.), (*Z*)-(4-fluorobut-3-en-1-yl)benzene (**1a**) (30.0 mg, 0.20 mmol, 2.0 equiv.) and KI (16.6 mg, 0.1 mmol, 1 equiv.). Automated flash column chromatography (10 g SiO_2, gradient elution: hexane to 60% EtOAc in hexane) afforded the desired product (+) **5ak** as a yellow oil (16.0 mg, 72%) in >99:1 diastereomeric ratio.

^{1}H NMR (400 MHz, $CDCl_3$) δ 7.33 – 7.23 (m, 2H), 7.22 – 7.12 (m, 3H), 5.99 – 5.23 (m, 2H), 4.61 (dtd, *J* = 48.3, 7.1, 4.5 Hz, 1H), 2.68 – 2.40 (m, 3H), 1.88 – 1.60 (m, 4H), 1.17 (d, *J* = 7.2 Hz, 3H).

^{13}C NMR (101 MHz, $CDCl_3$) δ 175.66 (d, $^3J_{C–F}$ = 2.6 Hz), 175.65, 141.80, 128.40, 125.92, 95.08 (d, $^1J_{C–F}$ = 171.3 Hz), 45.61 (d, $^2J_{C–F}$ = 20.5 Hz), 35.45, 31.93 (d, $^2J_{C–F}$ = 20.9 Hz), 26.62 (d, $^3J_{C–F}$ = 3.3 Hz), 13.83 (d, $^3J_{C–F}$ = 7.3 Hz).

^{19}F{1H} NMR (376 MHz, $CDCl_3$) δ -183.06.

HRMS (ESI/QTOF) m/z: $[M + Na]^+$ Calcd for $C_{13}H_{18}FNNaO^+$ 246.1265; Found 246.1274.

$[\alpha]_D^{23}$ = +7.6 (c = 0.50 in $CHCl_3$).

HPLC: The enantiomeric excess (98%) was determined *via* HPLC analysis using a CHIRALCEL® OD-H column, with hexane:isopropanol = 90:10 at a flow rate 1.0 mL/min detected at 214 nm wavelength. Retention time: t_{major} = 16.5 min and t_{minor} = 15.4 min. Diastereomeric ratio (96:4) was determined by ^{19}F-NMR of the crude reaction mixture.

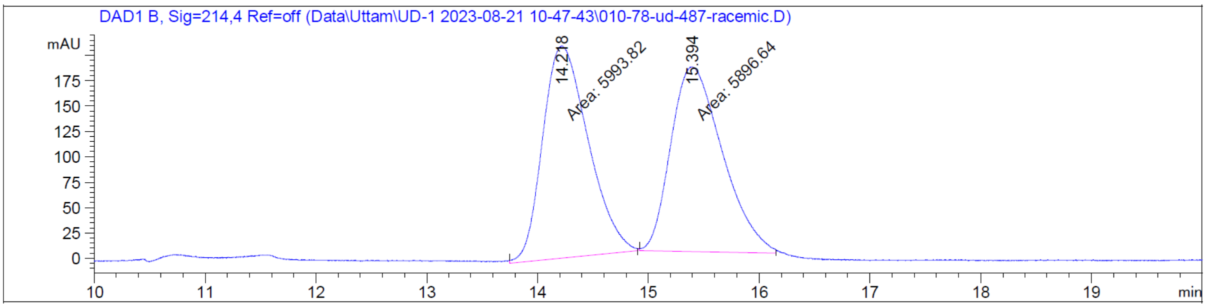

Peak #	RetTime [min]	Type	Width [min]	Area [mAU*s]	Height [mAU]	Area %
1	14.218	MM	0.4762	5993.82129	209.78477	50.4086
2	15.394	MM	0.5387	5896.64209	182.44157	49.5914

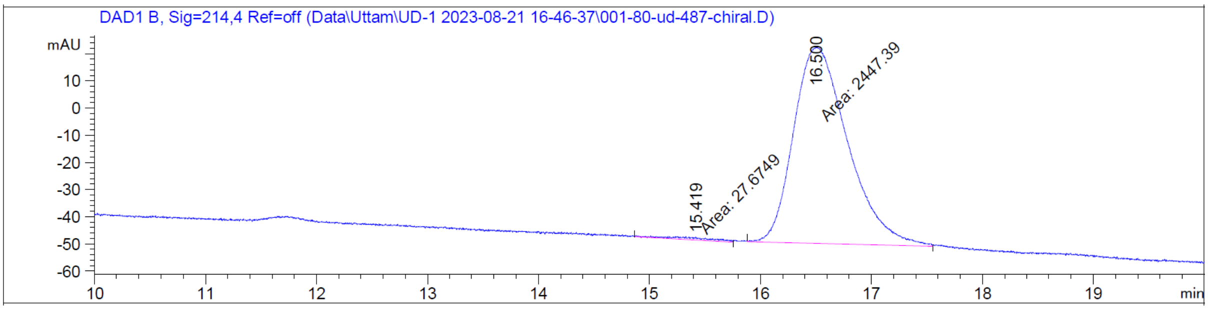

Peak #	RetTime [min]	Type	Width [min]	Area [mAU*s]	Height [mAU]	Area %
1	15.419	MM	0.4448	27.67489	1.03691	1.1181
2	16.500	MM	0.5643	2447.39478	72.28289	98.8819

(2*S*,3*R*)-3-Fluoro-*N*-methoxy-*N*,2-dimethyl-6-phenylhexanamide (5al)

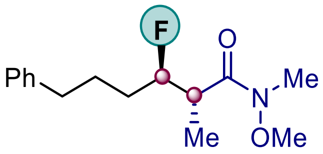

A modified procedure **GP2** was followed with 2-bromo-*N*-methoxy-*N*-methylpropanamide (**4l**) (19.6 mg, 0.10 mmol, 1.0 equiv.), (*Z*)-(4-fluorobut-3-en-1-yl)benzene (**1a**) (30.0 mg, 0.20 mmol, 2.0 equiv.) and KI (16.6 mg, 0.1 mmol, 1 equiv.). Automated flash column chromatography (10 g SiO_2, gradient elution: hexane to 20% EtOAc in hexane) afforded the desired product (-) **5al** as a yellow oil (13.3 mg, 50%) in >99:1 diastereomeric ratio.

^{1}H NMR (400 MHz, $CDCl_3$) δ 7.32 – 7.25 (m, 2H), 7.22 – 7.14 (m, 3H), 4.76 (dtd, J = 48.0, 8.9, 2.4 Hz, 1H), 3.70 (s, 3H), 3.25 – 3.12 (m, 4H), 2.67 (qdd, J = 14.1, 8.7, 6.2 Hz, 2H), 1.91 – 1.60 (m, 4H), 1.05 (d, J = 7.0 Hz, 3H).

^{13}C NMR (101 MHz, $CDCl_3$) δ 174.66, 141.99, 128.41, 128.35, 125.84, 94.69 (d, $^{1}J_{C–F}$ = 170.2 Hz), 61.46, 39.77 (d, $^{2}J_{C–F}$ = 22.0 Hz), 35.57, 32.10, 31.52 (d, $^{2}J_{C–F}$ = 20.9 Hz), 26.40 (d, $^{3}J_{C–F}$ = 2.9 Hz), 13.14 (d, $^{3}J_{C–F}$ = 8.8 Hz).

^{19}F{1H} NMR (376 MHz, $CDCl_3$) δ -178.72.

HRMS (ESI/QTOF) m/z: [M + Na]$^+$ Calcd for $C_{15}H_{22}FNNaO_2^+$ 290.1527; Found 290.1534.

$[\alpha]_D^{23}$ = –0.5 (c = 0.91 in $CHCl_3$).

HPLC: The enantiomeric excess (99%) was determined *via* HPLC analysis using a CHIRALCEL® IC column, with hexane:isopropanol = 70:30 at a flow rate 1.0 mL/min detected at 210 nm wavelength. Retention time: t_{major} = 11.6 min and t_{minor} = 16.5 min. Diastereomeric ratio (>99:1) was determined by ^{19}F-NMR of the crude reaction mixture.

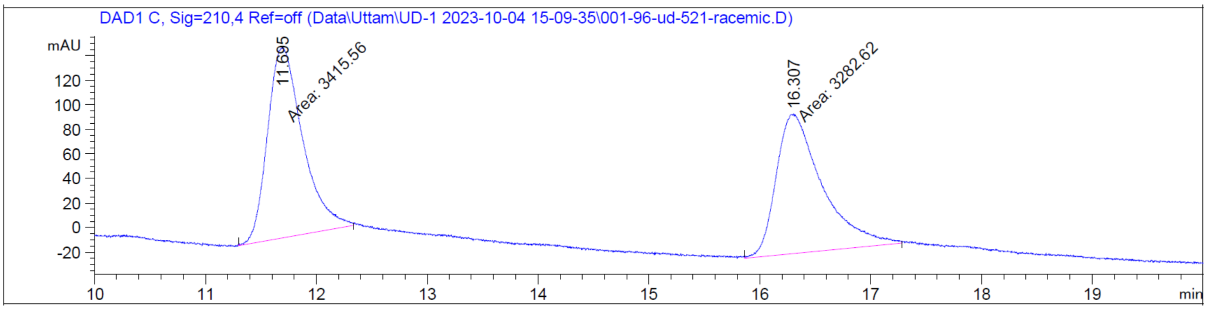

Peak #	RetTime [min]	Type	Width [min]	Area [mAU*s]	Height [mAU]	Area %
1	11.695	MM	0.3658	3415.55957	155.59998	50.9923
2	16.307	MM	0.4800	3282.62085	113.97581	49.0077

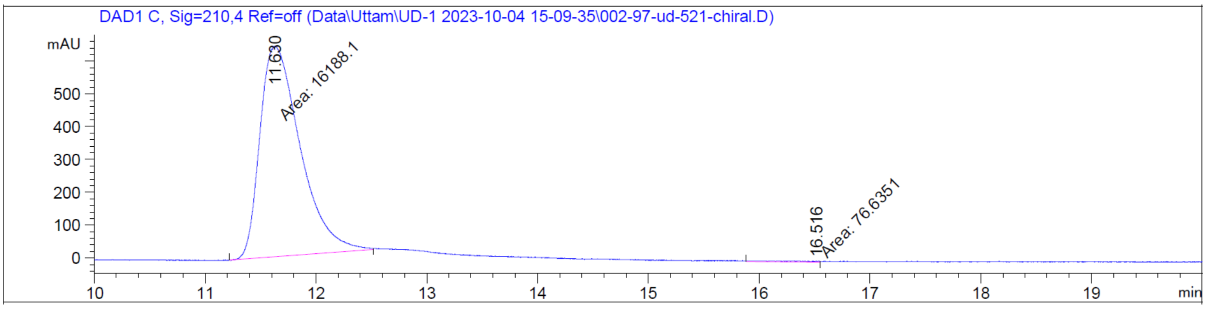

Peak #	RetTime [min]	Type	Width [min]	Area [mAU*s]	Height [mAU]	Area %
1	11.630	MM	0.4207	1.61881e4	641.28473	99.5288
2	16.516	MM	0.4428	76.63509	2.88476	0.4712

(2*S*,3*R*)-*N*-Benzyl-3-fluoro-2-methyl-6-phenylhexanamide (7aa)

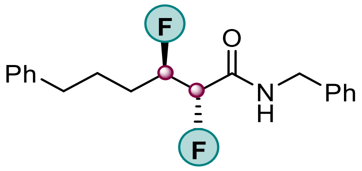

A modified procedure **GP2** was followed with *N*-benzyl-2-bromo-2-fluoroacetamide (**6a**) (25 mg, 0.10 mmol, 1.0 equiv.), (*Z*)-(4-fluorobut-3-en-1-yl)benzene (**1a**) (30 mg, 0.20 mmol, 2.0 equiv.) and KI (16.6 mg, 0.1 mmol, 1 equiv.). Automated flash column chromatography (10 g SiO_2, gradient elution: hexane to 20% EtOAc in hexane) afforded the desired product (-) **7aa** as a white solid (21.3 mg, 67%).

^{1}H NMR (400 MHz, $CDCl_3$) δ 7.37 – 7.22 (m, 7H), 7.21 – 7.13 (m, 3H), 6.62 (brs, 1H), 5.12 (ddd, *J* = 49.5, 19.2, 1.8 Hz, 1H), 4.91 (ddddd, *J* = 47.4, 25.2, 9.9, 3.3, 1.8 Hz, 1H), 4.56 – 4.41 (m, 2H), 2.64 (t, *J* = 7.5 Hz, 2H), 2.00 – 1.82 (m, 2H), 1.79 – 1.66 (m, 1H), 1.66 – 1.45 (m, 1H).

^{13}C NMR (101 MHz, $CDCl_3$) (one resonance is missing due to overlap): δ 165.96 (dd, $^{2}J_{C–F}$ = 19.3, $^{3}J_{C–F}$ = 9.8 Hz), 141.72, 137.27, 129.01, 128.54, 128.01, 127.93, 126.09, 92.74 (dd, $^{1}J_{C–F}$ = 177.7, $^{2}J_{C–F}$ = 19.9 Hz), 92.03 (dd, $^{1}J_{C–F}$ = 194.3, $^{2}J_{C–F}$ = 22.8 Hz), 43.25, 35.46, 28.32 (dd, $^{2}J_{C–F}$ = 21.6, $^{3}J_{C–F}$ = 6.0 Hz), 26.98 (d, $^{3}J_{C–F}$ = 3.3 Hz).

^{19}F{1H} NMR (377 MHz, $CDCl_3$) δ -192.85 (d, *J* = 12.6 Hz), -200.34 (d, *J* = 13.0 Hz).

HRMS (ESI/QTOF) m/z: $[M + Na]^+$ Calcd. for $C_{19}H_{21}F_2NNaO^+$ 340.1483; Found 340.1490.

$[\alpha]_D^{25}$ = -32.4 (c = 0.50 in $CHCl_3$).

HPLC: The enantiomeric excess (97%) was determined *via* HPLC analysis using a CHIRALCEL® IA column, with hexane:isopropanol = 95:5 at a flow rate 1.0 mL/min detected at 230 nm wavelength. Retention time: t_{major} = 7.2 min and t_{minor} = 18.6 min. Diastereomeric ratio (96:4) was determined by ^{19}F-NMR analysis of the crude reaction mixture.

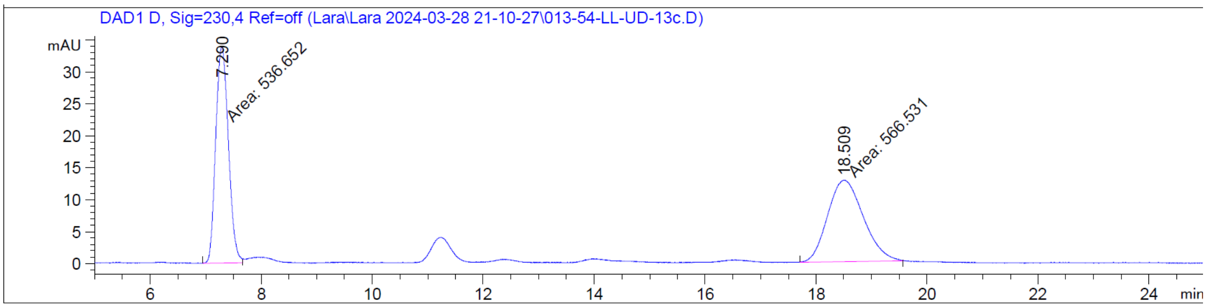

Peak #	RetTime [min]	Type	Width [min]	Area [mAU*s]	Height [mAU]	Area %
1	7.290	MM	0.2649	536.65161	33.76836	48.6458
2	18.509	MM	0.7415	566.53125	12.73415	51.3542

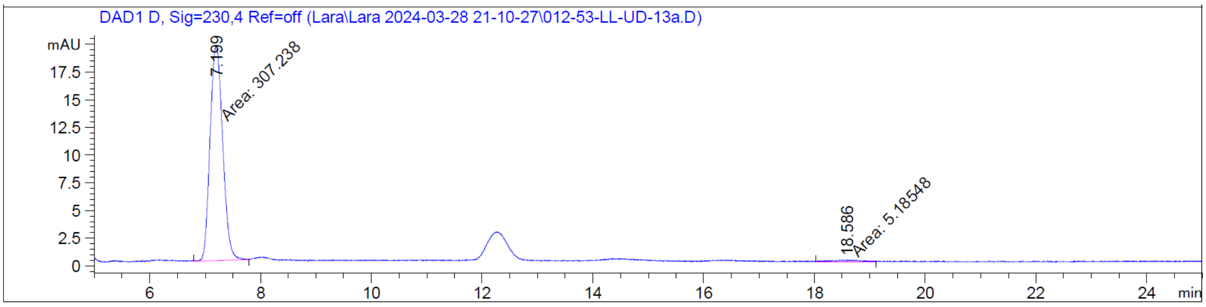

Peak #	RetTime [min]	Type	Width [min]	Area [mAU*s]	Height [mAU]	Area %
1	7.199	MM	0.2643	307.23825	19.37284	98.3402
2	18.586	MM	0.5629	5.18548	1.53544e-1	1.6598

(2*S*,3*R*)-*N*-Benzyl-2,3-difluorohexadecanamide (7ha)

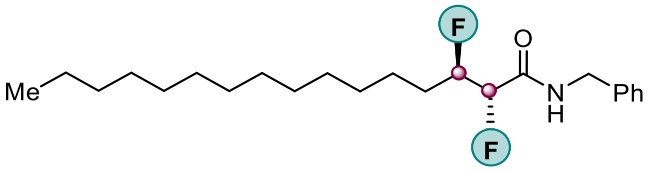

A modified procedure **GP2** was followed with *N*-benzyl-2-bromo-2-fluoroacetamide (**6a**) (25 mg, 0.10 mmol, 1.0 equiv.), (*Z*)-1-fluorotetradec-1-ene (**1h**) (43 mg, 0.20 mmol, 2.0 equiv.) and KI (16.6 mg, 0.1 mmol, 1 equiv.). Automated flash column chromatography (10 g SiO_2, gradient elution: hexane to 10% EtOAc in hexane) afforded the desired product (+) **7ha** as a white solid (23 mg, 60%).

^1H NMR (400 MHz, $CDCl_3$) δ 7.40 – 7.22 (m, 5H), 6.66 (bs, 1H), 5.35 – 4.80 (m, 2H), 4.59 – 4.41 (m, 2H), 1.87 (dqd, J = 13.4, 9.7, 5.0 Hz, 1H), 1.56 – 1.47 (m, 1H), 1.26 (s, 22H), 0.88 (t, J = 6.7 Hz, 3H).

^{13}C NMR (101 MHz, $CDCl_3$) δ 165.95 (dd, $^2J_{C–F}$ = 19.6, $^3J_{C–F}$ = 9.8 Hz), 137.22, 128.86, 127.85, 127.79, 92.75 (dd, $^1J_{C–F}$ = 176.9, $^2J_{C–F}$ = 19.6 Hz), 92.09 (dd, $^1J_{C–F}$ = 193.8, $^2J_{C–F}$ = 22.7 Hz), 43.10, 31.94, 29.70, 29.67, 29.65, 29.63, 29.54, 29.39, 29.37, 29.23, 28.68 (dd, $^2J_{C–F}$ = 21.4, $^3J_{C–F}$ = 5.8 Hz), 25.11 (d, $^3J_{C–F}$ = 3.6 Hz), 22.70, 14.13.

^{19}F NMR (377 MHz, $CDCl_3$) δ -192.36 – -193.00 (m), -200.24 (dddd, J = 49.4, 25.1, 12.6, 4.1 Hz).

HRMS (nanochip-ESI/LTQ-Orbitrap) m/z: $[M + H]^+$ Calcd for $C_{23}H_{38}F_2NO^+$ 382.2916; Found 382.2916.

$[\alpha]_D^{25}$ = +0.4 (c = 1.0 in $CHCl_3$)

HPLC: The enantiomeric excess (91%) was determined *via* HPLC analysis using a CHIRALCEL® IA column, with hexane:isopropanol = 95:5 at a flow rate 1.0 mL/min detected at 214 nm wavelength. Retention time: t_{major} = 6.4 min and t_{minor} = 7.9 min. Diastereomeric ratio (91:9) was determined by ^{19}F-NMR analysis of the crude reaction mixture.

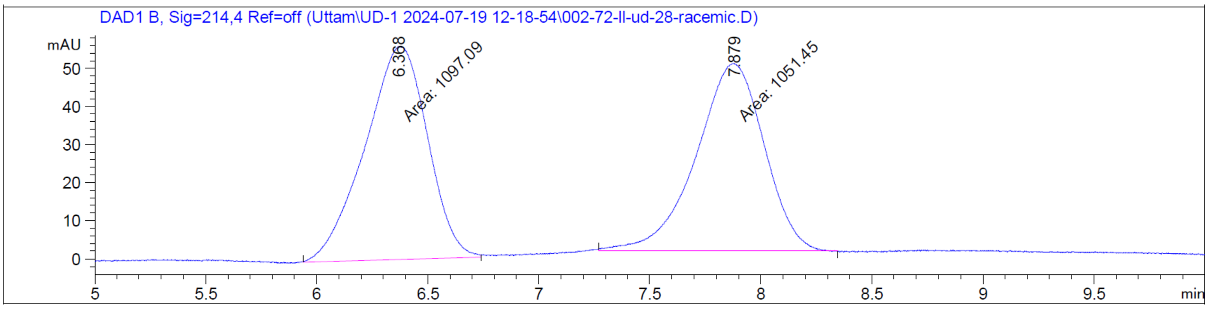

Peak #	RetTime [min]	Type	Width [min]	Area [mAU*s]	Height [mAU]	Area %
1	6.368	MM	0.3271	1097.08630	55.89845	51.0620
2	7.879	MM	0.3558	1051.45325	49.24908	48.9380

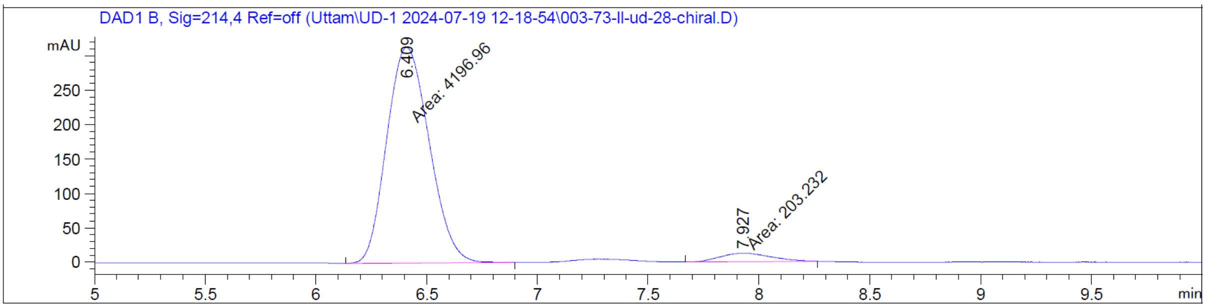

Peak #	RetTime [min]	Type	Width [min]	Area [mAU*s]	Height [mAU]	Area %
1	6.409	MM	0.2228	4196.95703	313.94708	95.3813
2	7.927	MM	0.2701	203.23203	12.53837	4.6187

(2*S*,3*R*)-*N*-benzyl-6-((*tert*-butyldimethylsilyl)oxy)-2,3-difluorohexanamide (7da)

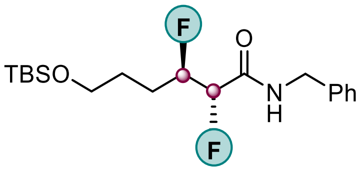

Prepared according to **GP2** with *N*-benzyl-2-bromo-2-fluoroacetamide (**6a**) (25 mg, 0.10 mmol, 1.0 equiv.) and (*Z*)-*tert*-butyl((4-fluorobut-3-en-1-yl)oxy)dimethylsilane (**1d**) (41 mg, 0.20 mmol, 2.0 equiv.). Automated flash column chromatography (10 g SiO_2, gradient elution: hexane to 15% EtOAc in hexane) afforded the desired product (+) **7da** as a colourless oil (19.3 mg, 52%).

^{1}H NMR (400 MHz, $CDCl_3$) δ 7.40 – 7.26 (m, 5H), 6.65 (br s, 1H), 5.14 (ddd, *J* = 49.4, 19.6, 1.8 Hz, 1H), 5.11 – 4.86 (m, 1H), 4.49 (d, *J* = 5.8 Hz, 2H), 3.71 – 3.59 (m, 2H), 2.04 – 1.86 (m, 1H), 1.81 – 1.69 (m, 1H), 1.69 – 1.57 (m, 2H), 0.89 (s, 9H), 0.05 (s, 6H).

^{13}C NMR (101 MHz, $CDCl_3$) δ 165.99 (dd, $^{2}J_{C–F}$ = 19.4, $^{3}J_{C–F}$ = 9.6 Hz), 137.29, 128.99, 127.99, 127.93, 92.80 (dd, $^{1}J_{C–F}$ = 177.0, $^{2}J_{C–F}$ = 19.8 Hz), 92.16 (dd, $^{1}J_{C–F}$ = 193.9, $^{2}J_{C–F}$ = 22.7 Hz) 62.50, 43.26, 28.42 (d, $^{3}J_{C–F}$ = 3.3 Hz), 26.07, 25.55 (dd, $^{2}J_{C–F}$ = 21.5, $^{3}J_{C–F}$ = 6.3 Hz), 18.44, -5.20, -5.21.

^{19}F NMR (377 MHz, $CDCl_3$) δ -192.73 (dddt, *J* = 48.8, 35.9, 19.8, 13.1 Hz), -200.01 (dddd, *J* = 49.3, 24.8, 12.7, 4.1 Hz).

HRMS (nanochip-ESI/LTQ-Orbitrap) m/z: $[M + H]^+$ Calcd. for $C_{19}H_{32}F_2NO_2Si^+$ 372.2165; Found 372.2161.

$[\alpha]_D^{25}$ = +1.7 (c = 1.0 in $CHCl_3$).

HPLC: The enantiomeric excess (93%) was determined *via* HPLC analysis using a CHIRALCEL® OD-H column, with hexane:isopropanol = 98:2 at a flow rate 1.0 mL/min detected at 210 nm wavelength. Retention time: t_{major} = 14.9 min and t_{minor} = 22.1 min. Diastereomeric ratio (93:7) was determined by ^{19}F-NMR analysis of the crude reaction mixture.

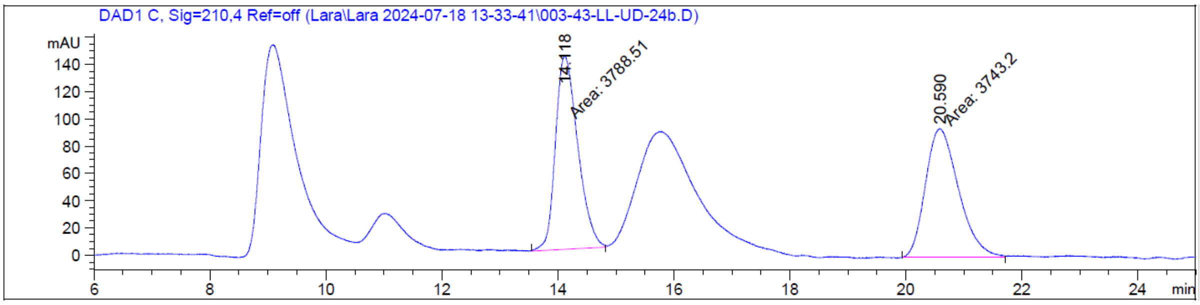

Peak #	RetTime [min]	Type	Width [min]	Area [mAU*s]	Height [mAU]	Area %
1	14.118	MM	0.4455	3788.50659	141.74707	50.3008
2	20.590	MM	0.6615	3743.19946	94.30700	49.6992

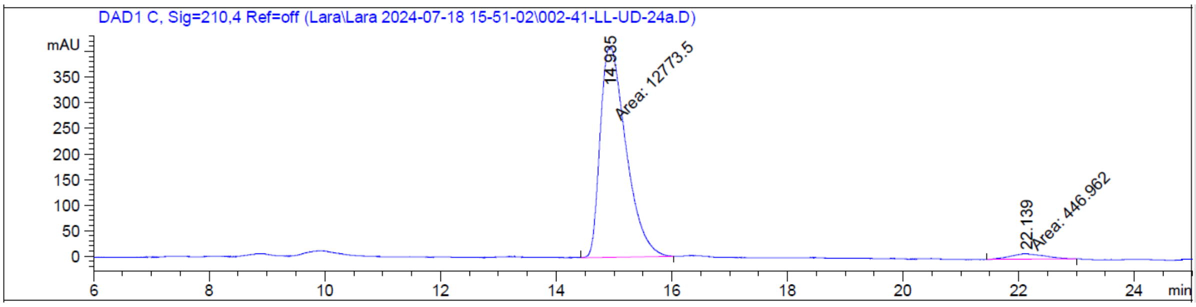

Peak #	RetTime [min]	Type	Width [min]	Area [mAU*s]	Height [mAU]	Area %
1	14.935	MM	0.5161	1.27735e4	412.47766	96.6192
2	22.139	MM	0.6950	446.96234	10.71780	3.3808

(2*S*,3*R*)-*N*-benzyl-6-(1,3-dioxoisoindolin-2-yl)-2,3-difluorohexanamide (7ea)

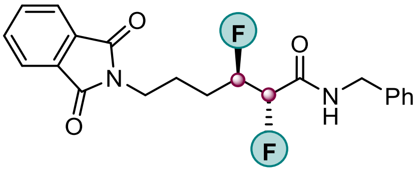

Prepared according to **GP2** with *N*-benzyl-2-bromo-2-fluoroacetamide (**6a**) (25 mg, 0.10 mmol, 1.0 equiv.) and (*Z*)-2-(4-fluorobut-3-en-1-yl)isoindoline-1,3-dione (**1e**) (44 mg, 0.20 mmol, 2.0 equiv.). Automated flash column chromatography (10 g SiO_2, gradient elution: 10% EtOAc in hexane to 40% EtOAc in hexane) afforded the desired product (+) **7ea** as a white solid (25.4 mg, 66%).

^{1}H NMR (400 MHz, $CDCl_3$) δ 7.88 – 7.80 (m, 2H), 7.76 – 7.67 (m, 2H), 7.41 – 7.25 (m, 5H), 6.67 (br s, 1H), 5.12 (ddd, J = 49.3, 19.1, 1.8 Hz, 1H), 5.13 – 4.87 (m, 1H), 4.48 (d, J = 5.9 Hz, 2H), 3.79 – 3.63 (m, 2H), 2.08 – 1.87 (m, 2H), 1.87 – 1.74 (m, 1H), 1.70 – 1.51 (m, 1H).

^{13}C NMR (101 MHz, $CDCl_3$) δ 168.45, 165.80 (dd, $^{2}J_{C–F}$ = 19.3, $^{3}J_{C–F}$ = 9.6 Hz), 137.24, 134.12, 132.20, 128.99, 127.98, 127.91, 123.42, 92.18 (dd, $^{1}J_{C–F}$ = 177.9, $^{2}J_{C–F}$ = 19.8 Hz), 91.91 (dd, $^{1}J_{C–F}$ = 194.6, $^{2}J_{C–F}$ = 22.6 Hz), 43.26, 37.48, 26.27 (dd, $^{2}J_{C–F}$ = 21.8, $^{3}J_{C–F}$ = 6.3 Hz), 24.59 (d, $^{3}J_{C–F}$ = 3.4 Hz).

^{19}F NMR (377 MHz, $CDCl_3$) δ -193.21 (dddt, J = 48.5, 36.5, 19.2, 12.9 Hz), -200.27 (dddd, J = 49.2, 24.8, 12.9, 4.0 Hz).

HRMS (nanochip-ESI/LTQ-Orbitrap) m/z: $[M + H]^+$ Calcd for $C_{21}H_{21}F_2N_2O_3^+$ 387.1515; Found 387.1511.

$[\alpha]_D^{25}$ = +3.4 (c = 1.0 in $CHCl_3$).

HPLC: The enantiomeric excess (95%) was determined *via* HPLC analysis using a CHIRALCEL® IA column, with hexane:isopropanol = 80:20 at a flow rate 1.0 mL/min detected at 230 nm wavelength. Retention time: t_{major} = 16.1 min and t_{minor} = 17.7 min. Diastereomeric ratio (92:8) was determined by ^{19}F-NMR analysis of the crude reaction mixture.

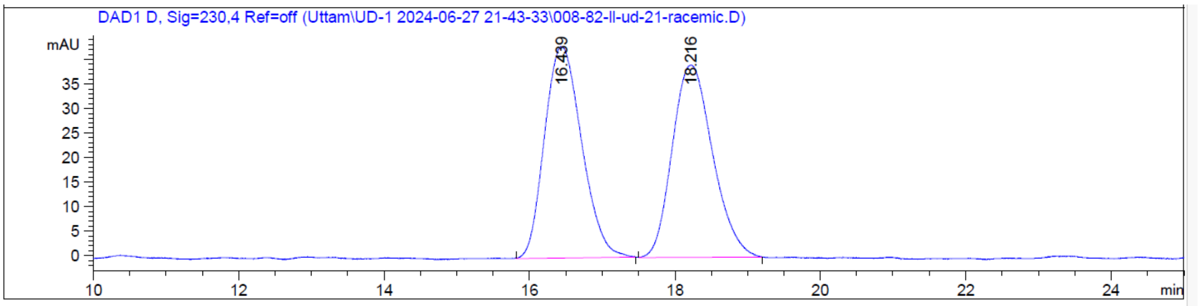

Peak #	RetTime [min]	Type	Width [min]	Area [mAU*s]	Height [mAU]	Area %
1	16.439	VB	0.4163	1528.58789	43.10122	50.3098
2	18.216	BB	0.4510	1509.76404	39.18946	49.6902

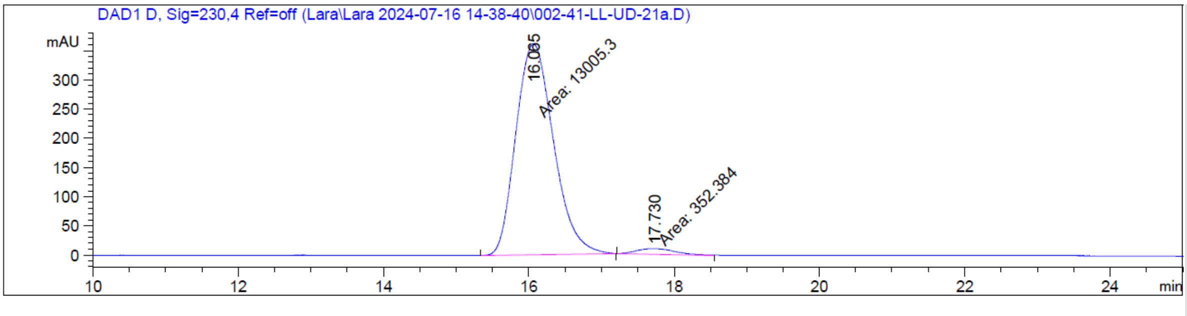

Peak #	RetTime [min]	Type	Width [min]	Area [mAU*s]	Height [mAU]	Area %
1	16.065	MM	0.5994	1.30053e4	361.63382	97.3619
2	17.730	MM	0.5916	352.38428	9.92744	2.6381

(2*S*,3*R*)-2,3-Difluoro-*N*-(4-methoxyphenyl)-6-phenylhexanamide (7ab)

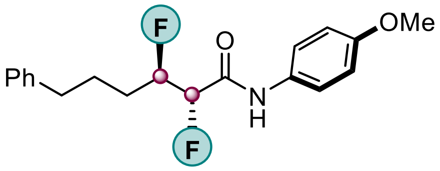

A modified procedure **GP2** was followed with 2-bromo-2-fluoro-*N*-(4-methoxyphenyl)acetamide (**6b**) (26.2 mg, 0.10 mmol, 1.0 equiv.), (*Z*)-(4-fluorobut-3-en-1-yl)benzene (**1a**) (30.0 mg, 0.20 mmol, 2.0 equiv.) and KI (16.6 mg, 0.1 mmol, 1 equiv.). Automated flash column chromatography (10 g SiO_2, gradient elution: hexane to 10% EtOAc in hexane) afforded the desired product (+) **7ab** as a yellow oil (16.5 mg, 50%) in >99:1 diastereomeric ratio.

^{1}H NMR (400 MHz, $CDCl_3$) δ 7.90 (bs, 1H), 7.50 – 7.39 (m, 2H), 7.34 – 7.24 (m, 2H), 7.24 – 7.13 (m, 3H), 6.92 (dt, *J* = 9.0, 1.7 Hz, 2H), 5.36 – 4.88 (m, 2H), 3.84 (s, 3H), 2.84 – 2.63 (m, 2H), 2.14 – 1.61 (m, 4H).

^{13}C NMR (101 MHz, $CDCl_3$) (one resonance is missing due to overlap): δ 163.73 (dd, $^3J_{C–F}$ = 9.8, $^2J_{C–F}$ = 18.8 Hz), 157.16, 141.57, 129.25, 128.41, 125.96, 122.02, 114.32, 92.56 (dd, $^1J_{C-F}$ = 177.1, $^2J_{C–F}$ =19.4 Hz), 92.02 (dd, $^1J_{C–F}$ = 194.4, $^2J_{C–F}$ = 22.7 Hz), 55.53, 35.29, 28.22 (dd, $^2J_{C–F}$ = 21.5, $^3J_{C–F}$ = 6.1 Hz), 26.91 (d, $^3J_{C–F}$ = 3.3 Hz).

^{19}F NMR (376 MHz, $CDCl_3$) δ -192.18 – -193.52 (m), -198.20 (dddd, *J* = 49.7, 25.7, 12.7, 5.8 Hz).

HRMS (APCI/QTOF) m/z: $[M + Na]^+$ Calcd for $C_{19}H_{21}F_2NNaO_2^+$ 356.1433; Found 356.1431.

$[\alpha]_D^{23}$ = +29.8 (c = 0.50 in $CHCl_3$).

HPLC: The enantiomeric excess (89%) was determined *via* HPLC analysis using a CHIRALCEL® OD-H column, with hexane:isopropanol = 90:10 at a flow rate 1.0 mL/min detected at 254 nm wavelength. Retention time: t_{major} = 28.8 min and t_{minor} = 41.5 min. Diastereomeric ratio (90:10) was determined by ^{19}F-NMR of the crude reaction mixture.

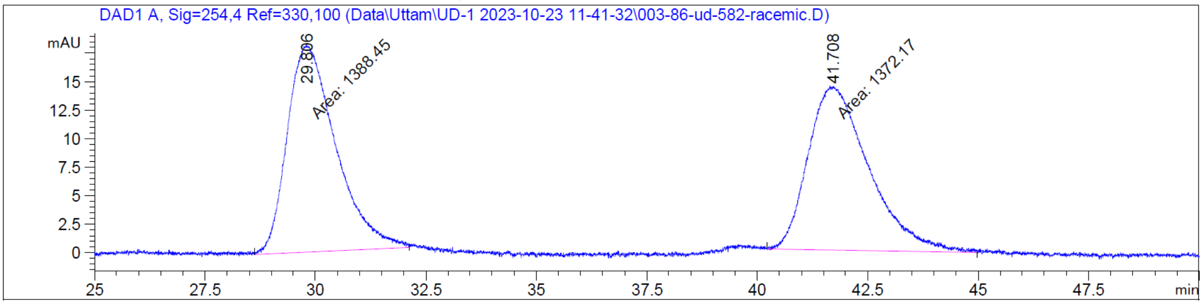

Peak #	RetTime [min]	Type	Width [min]	Area [mAU*s]	Height [mAU]	Area %
1	29.806	MM	1.2695	1388.44861	18.22792	50.2948
2	41.708	MM	1.5887	1372.17432	14.39483	49.7052

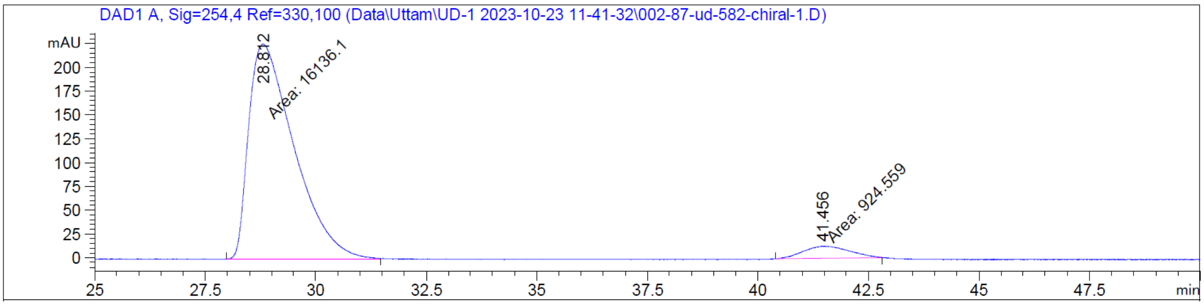

Peak #	RetTime [min]	Type	Width [min]	Area [mAU*s]	Height [mAU]	Area %
1	28.812	MM	1.1895	1.61361e4	226.08961	94.5808
2	41.456	MM	1.2131	924.55927	12.70212	5.4192

(2*S*,3*R*)-2,3-Difluoro-*N*-(1-methyl-1*H*-indol-6-yl)-6-phenylhexanamide (7ac)

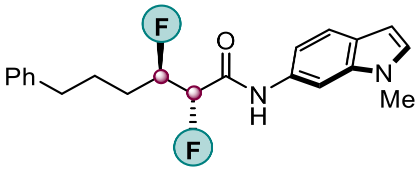

A modified procedure **GP2** was followed with 2-bromo-2-fluoro-*N*-(1-methyl-1*H*-indol-6-yl)acetamide (**6c**) (28.5 mg, 0.10 mmol, 1.0 equiv.), (*Z*)-(4-fluorobut-3-en-1-yl)benzene (**1a**) (30.0 mg, 0.20 mmol, 2.0 equiv.) and KI (16.6 mg, 0.1 mmol, 1 equiv.). Automated flash

column chromatography (10 g SiO_2, gradient elution: hexane to 30% EtOAc in hexane) afforded the desired product (+) **7ac** as a yellow oil (17.0 mg, 48%) in >99:1 diastereomeric ratio.

^{1}H NMR (400 MHz, $CDCl_3$) δ 8.02 (bs, 1H), 7.86 (d, J = 1.8 Hz, 1H), 7.27 (td, J = 6.7, 3.8 Hz, 4H), 7.22 – 7.14 (m, 3H), 7.08 (d, J = 3.1 Hz, 1H), 6.47 (d, J = 3.1 Hz, 1H), 5.38 – 4.92 (m, 2H), 3.79 (s, 3H), 2.67 (td, J = 9.4, 4.8 Hz, 2H), 2.06 – 1.62 (m, 4H).

^{13}C NMR (101 MHz, $CDCl_3$) (one resonance is missing due to overlap): δ 163.77 (dd, $^2J_{C–F}$ = 18.0, $^3J_{C–F}$ = 10.3 Hz), 141.65, 134.66, 130.03, 128.55, 128.44, 128.41, 125.94, 115.70, 113.12, 109.50, 101.21, 92.66 (dd, $^1J_{C–F}$ = 177.3, $^2J_{C–F}$ = 19.6 Hz), 92.14 (dd, $^1J_{C–F}$ = 195.9, $^2J_{C–F}$ = 22.7 Hz), 35.34, 32.99, 28.19 (dd, $^2J_{C–F}$ = 21.6, $^3J_{C–F}$ = 5.9 Hz), 26.97 (d, $^3J_{C–F}$ = 3.3 Hz).

^{19}F NMR (376 MHz, $CDCl_3$) δ -192.63 (dddt, J = 49.5, 36.7, 18.6, 13.2 Hz), -198.77 (dddd, J = 49.8, 25.7, 12.8, 5.9 Hz).

HRMS (APCI/QTOF) m/z: $[M + Na]^+$ Calcd for $C_{21}H_{22}F_2N_2NaO^+$ 379.1592; Found 379.1591.

$[\alpha]_D^{23}$ = +35.7 (c = 0.5 in $CHCl_3$).

HPLC: The enantiomeric excess (85%) was determined *via* HPLC analysis using a CHIRALCEL® IA column, with hexane:isopropanol = 80:20 at a flow rate 1.0 mL/min detected at 254 nm wavelength. Retention time: t_{major} = 12.3 min and t_{minor} = 26.7 min. Diastereomeric ratio (91:9) was determined by ^{19}F-NMR of the crude reaction mixture.

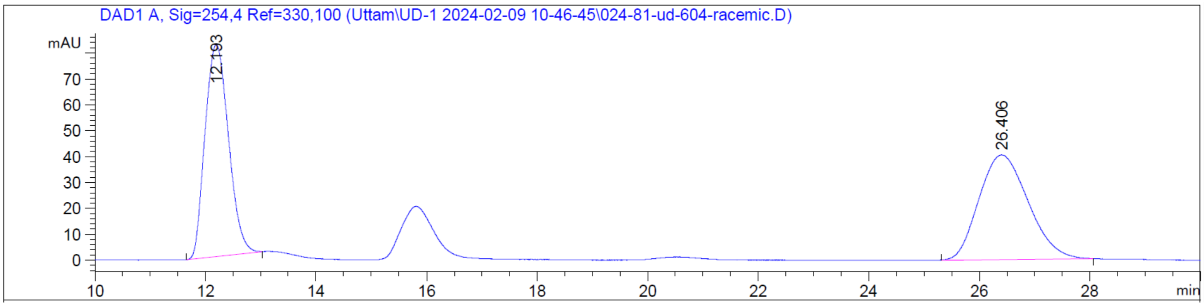

Peak #	RetTime [min]	Type	Width [min]	Area [mAU*s]	Height [mAU]	Area %
1	12.193	BB	0.3515	2407.72656	81.76753	49.1499
2	26.406	BB	0.7231	2491.01270	40.36012	50.8501

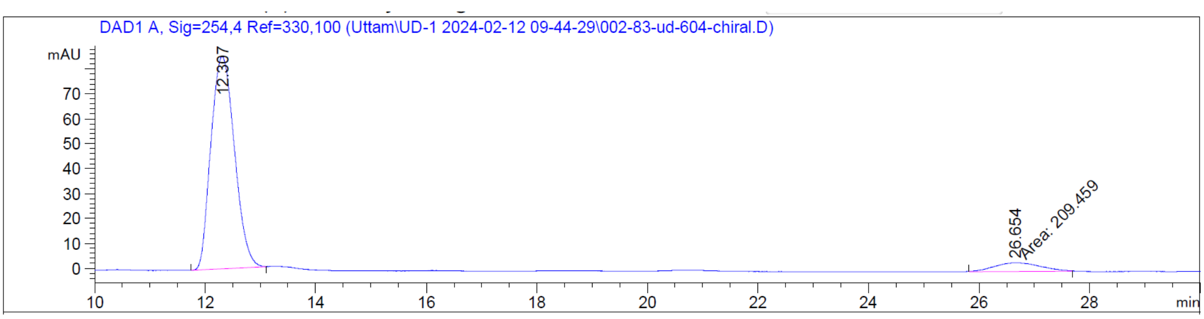

Peak #	RetTime [min]	Type	Width [min]	Area [mAU*s]	Height [mAU]	Area %
1	12.307	BB	0.3587	2525.43628	85.34976	92.3412
2	26.654	MM	0.9668	209.45897	3.61093	7.6588

(2*S*,3*R*)-2,3-Difluoro-*N*-(furan-2-ylmethyl)-6-phenylhexanamide (7ad)

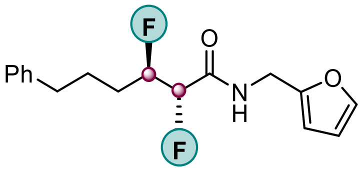

Prepared according to **GP2** with 2-bromo-2-fluoro-*N*-(furan-2-ylmethyl)acetamide (**6d**) (24 mg, 0.10 mmol, 1.0 equiv.) and (*Z*)-(4-fluorobut-3-en-1-yl)benzene (**1a**) (30 mg, 0.20 mmol, 2.0 equiv.). Automated flash column chromatography (10 g SiO_2, gradient elution: hexane to 20% EtOAc in hexane) afforded the desired product (-) **7ad** as a white solid (20.7 mg, 67%).

^{1}H NMR (400 MHz, $CDCl_3$) δ 7.37 – 7.32 (m, 1H), 7.31 – 7.23 (m, 2H), 7.22 – 7.12 (m, 3H), 6.63 (br s, 1H), 6.34 – 6.30 (m, 1H), 6.25 – 6.21 (m, 1H), 5.11 (ddd, *J* = 49.6, 18.7, 1.8 Hz, 1H), 5.06 – 4.83 (m, 1H), 4.47 (d, *J* = 5.7 Hz, 2H), 2.63 (br t, *J* = 7.2 Hz, 2H), 2.01 – 1.80 (m, 2H), 1.76 – 1.66 (m, 1H), 1.65 – 1.44 (m, 1H).

^{13}C NMR (101 MHz, $CDCl_3$) (one resonance is missing due to overlap) δ 165.94 (dd, $^2J_{C–F}$ = 19.5, $^3J_{C–F}$ = 10.0 Hz), 150.27, 142.69, 141.73, 128.53, 126.07, 110.66, 108.08, 92.58 (dd, $^1J_{C–F}$ = 177.4, $^2J_{C–F}$ = 19.6 Hz), 92.07 (dd, $^1J_{C–F}$ = 194.3, $^2J_{C–F}$ = 22.7 Hz), 36.13, 35.48, 28.23 (dd, $^2J_{C–F}$ = 21.5, $^3J_{C–F}$ = 6.0 Hz), 26.95 (d, $^3J_{C–F}$ = 3.3 Hz).

^{19}F NMR (377 MHz, $CDCl_3$) δ -192.54 – -192.91 (m), -201.15 (dddd, *J* = 49.5, 25.4, 12.6, 4.0 Hz).

HRMS (nanochip-ESI/LTQ-Orbitrap) m/z: [M + H]+ Calcd. for or $C_{17}H_{20}F_2NO_2^+$ 308.1457; Found 308.1453.

$[\alpha]_D^{23}$ = -8.1 (c = 1.0 in $CHCl_3$).

HPLC: The enantiomeric excess (92%) was determined *via* HPLC analysis using a CHIRALCEL® OD-H column, with hexane:isopropanol = 95:5 at a flow rate 1.0 mL/min detected at 210 nm wavelength. Retention time: t_{major} = 17.6 min and t_{minor} = 25.9 min. Diastereomeric ratio (90:10) was determined by ^{19}F-NMR analysis of the crude reaction mixture.

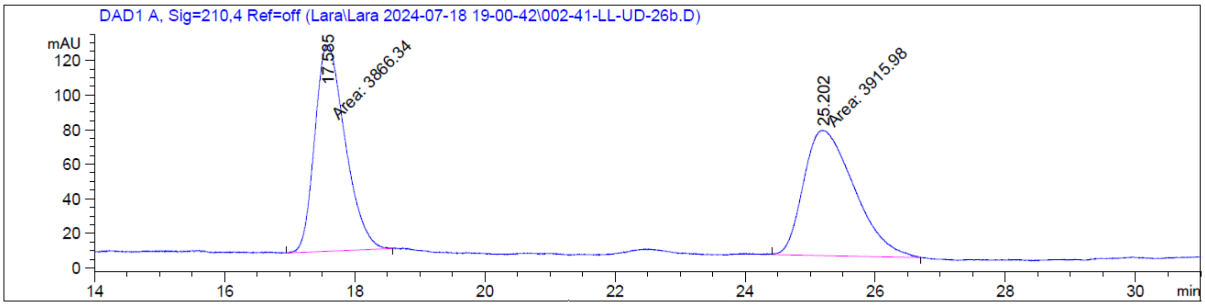

Peak #	RetTime [min]	Type	Width [min]	Area [mAU*s]	Height [mAU]	Area %
1	17.585	MM	0.5401	3866.33594	119.30859	49.6811
2	25.202	MM	0.8994	3915.97876	72.56256	50.3189

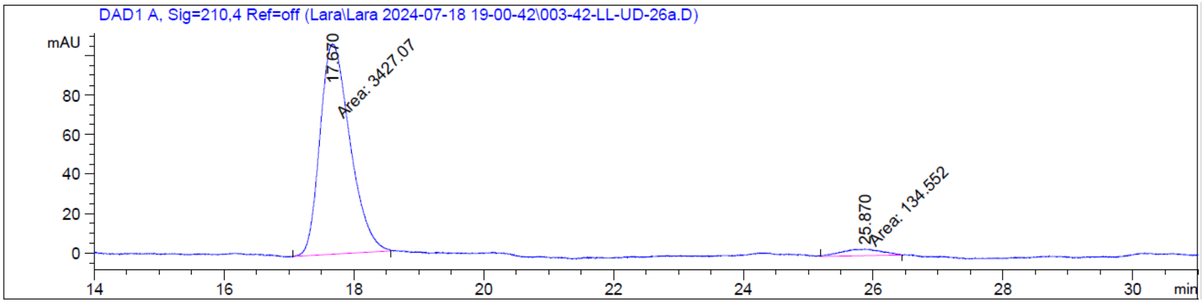

Peak #	RetTime [min]	Type	Width [min]	Area [mAU*s]	Height [mAU]	Area %
1	17.670	MM	0.5359	3427.06934	106.58334	96.2222
2	25.870	MM	0.6814	134.55194	3.29112	3.7778

(2*S*,3*R*)-*N*-(*tert*-butyl)-2,3-difluoro-6-phenylhexanamide (7ae)

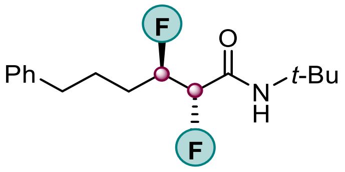

A modified procedure **GP2** was followed with 2-bromo-*N*-(*tert*-butyl)-2-fluoroacetamide (**6e**) (21.2 mg, 0.10 mmol, 1.0 equiv.), (*Z*)-(4-fluorobut-3-en-1-yl)benzene (**1a**) (30.0 mg, 0.20 mmol, 2.0 equiv.) and KI (16.6 mg, 0.10 mmol, 1 equiv.). Automated flash column chromatography (10 g SiO_2, gradient elution: hexane to 5% EtOAc in hexane) afforded the desired product (+) **7ae** as a yellow oil (17.0 mg, 60%) in >99:1 diastereomeric ratio.

^{1}H NMR (400 MHz, $CDCl_3$) δ 7.37 – 7.25 (m, 2H), 7.20 (d, *J* = 7.4 Hz, 3H), 6.14 (bs, 1H), 5.19 – 4.62 (m, 2H), 2.69 (t, *J* = 7.4 Hz, 2H), 1.91 (dddd, *J* = 14.2, 11.6, 7.2, 4.5 Hz, 2H), 1.83 – 1.70 (m, 1H), 1.67 – 1.50 (m, 1H), 1.44 – 1.32 (m, 9H).

^{13}C NMR (101 MHz, $CDCl_3$) δ 165.07 (dd, $^2J_{C–F}$ = 17.4, $^3J_{C–F}$ = 10.1 Hz), 141.61, 128.44, 128.39, 125.92, 92.57 (dd, $^1J_{C–F}$ = 177.0, $^2J_{C–F}$ = 19.6 Hz), 91.83 (dd, $^1J_{C–F}$ = 195.5, $^2J_{C–F}$ = 22.4 Hz), 51.67, 35.29, 28.60, 27.89 (dd, $^2J_{C–F}$ = 21.6, $^3J_{C–F}$ = 5.9 Hz), 26.79 (d, $^3J_{C–F}$ = 3.3 Hz).

^{19}F NMR (376 MHz, $CDCl_3$) δ -192.26 – -194.11 (m), -198.34 (dddd, *J* = 50.6, 26.1, 12.7, 5.1 Hz).

HRMS (APCI/QTOF) m/z: $[M + Na]^+$ Calcd for $C_{16}H_{23}F_2NNaO^+$ 306.1640; Found 306.1633.

$[\alpha]_D^{23}$ = +0.7 (c = 0.27 in $CHCl_3$).

HPLC: The enantiomeric excess (95%) was determined *via* HPLC analysis using a CHIRALCEL® OJ-H column, with hexane:isopropanol = 80:20 at a flow rate 1.0 mL/min detected at 210 nm wavelength. Retention time: t_{major} = 5.6 min and t_{minor} = 6.5 min. Diastereomeric ratio (94:6) was determined by ^{19}F-NMR of the crude reaction mixture.

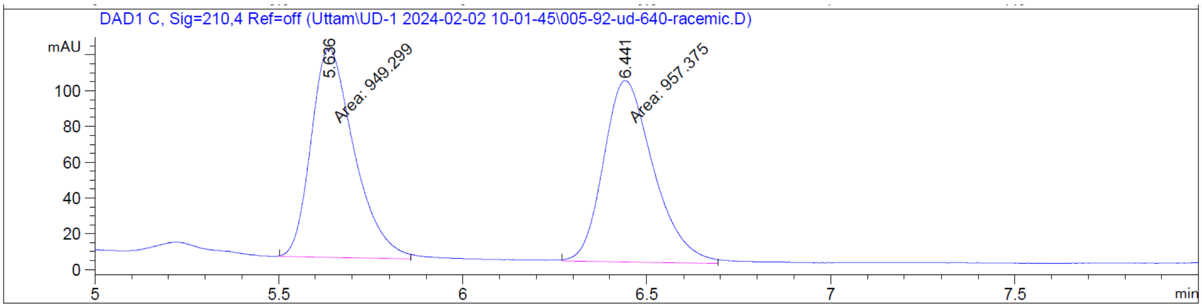

Peak #	RetTime [min]	Type	Width [min]	Area [mAU*s]	Height [mAU]	Area %
1	5.636	MM	0.1354	949.29932	116.89144	49.7882
2	6.441	MM	0.1573	957.37537	101.46133	50.2118

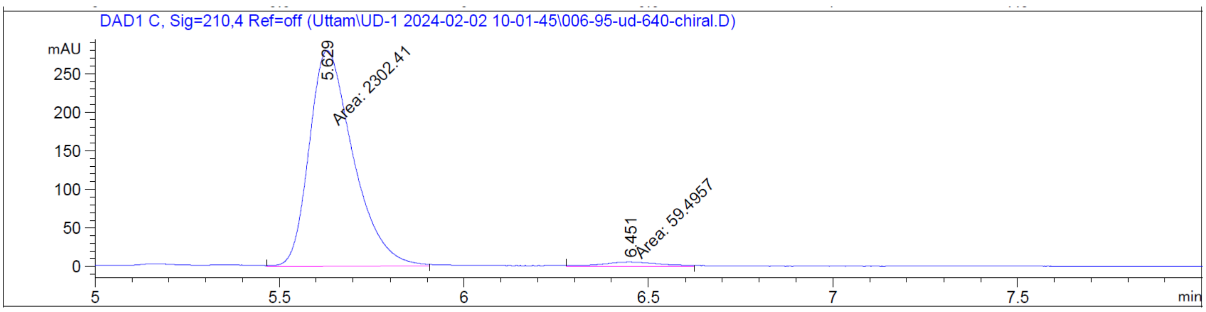

Peak #	RetTime [min]	Type	Width [min]	Area [mAU*s]	Height [mAU]	Area %
1	5.629	MM	0.1375	2302.40527	279.16226	97.4810
2	6.451	MM	0.1798	59.49567	5.51644	2.5190

(2*S*,3*R*)-2,3-Difluoro-6-phenyl-*N*-(tetrahydro-2*H*-pyran-4-yl)hexanamide (7af)

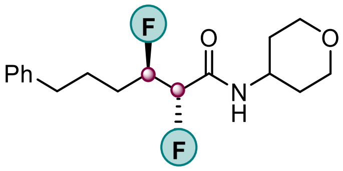

Prepared according to **GP2** with 2-bromo-2-fluoro-*N*-(tetrahydro-2*H*-pyran-4-yl)acetamide (**6f**) (24 mg, 0.10 mmol, 1.0 equiv.) and (*Z*)-(4-fluorobut-3-en-1-yl)benzene (**1a**) (30 mg, 0.20 mmol, 2.0 equiv.). Automated flash column chromatography (10 g SiO_2, gradient elution: hexane to 20% EtOAc in hexane) afforded the desired product (+) **7af** as a white solid (22.5 mg, 72%).

^{1}H NMR (400 MHz, $CDCl_3$) δ 7.32 – 7.23 (m, 2H), 7.21 – 7.13 (m, 3H), 6.23 (br s, 1H), 5.06 (ddd, *J* = 49.8, 18.7, 1.8 Hz, 1H), 5.02 – 4.81 (m, 1H), 4.07 – 3.87 (m, 3H), 3.46 (tdd, *J* = 11.5, 8.2, 2.4 Hz, 2H), 2.72 – 2.59 (m, 2H), 1.98 – 1.83 (m, 3H), 1.81 – 1.67 (m, 2H), 1.62 – 1.37 (m, 3H).

^{13}C NMR (101 MHz, $CDCl_3$) δ 165.37 (dd, $^{2}J_{C–F}$ = 19.1, $^{3}J_{C–F}$ = 9.9 Hz), 141.63, 128.54, 128.51, 126.06, 92.54 (dd, $^{1}J_{C–F}$ = 177.5, $^{2}J_{C–F}$ = 19.7 Hz), 91.98 (dd, $^{1}J_{C–F}$ = 194.3, $^{2}J_{C–F}$ = 22.6 Hz), 66.73, 66.69, 45.68, 35.37, 32.98, 32.82, 28.12 (dd, $^{2}J_{C–F}$ = 21.5, $^{3}J_{C–F}$ = 6.0 Hz), 26.86 (d, $^{3}J_{C–F}$ = 3.1 Hz).

^{19}F NMR (377 MHz, $CDCl_3$) δ -192.71 – -193.08 (m), -200.39 (dddd, *J* = 49.9, 25.6, 12.7, 4.2 Hz).

HRMS (nanochip-ESI/LTQ-Orbitrap) m/z: $[M + H]^+$ Calcd. for $C_{17}H_{24}F_2NO_2^+$ 312.1770; Found 312.1766.

$[\alpha]_D^{23}$ = 8.6 (c = 1.0 in $CHCl_3$).

HPLC: The enantiomeric excess (98%) was determined *via* HPLC analysis using a CHIRALCEL® OD-H column, with hexane:isopropanol = 90:10 at a flow rate 1.0 mL/min detected at 214 nm wavelength. Retention time: t_{major} = 10.2 min and t_{minor} = 11.1 min. Diastereomeric ratio (94:6) was determined by ^{19}F-NMR analysis of the crude reaction mixture.

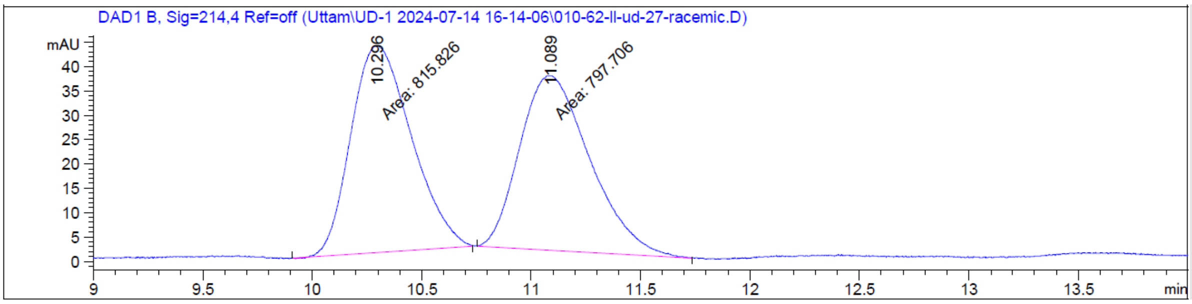

Peak #	RetTime [min]	Type	Width [min]	Area [mAU*s]	Height [mAU]	Area %
1	10.296	MM	0.3207	815.82623	42.39281	50.5615
2	11.089	MM	0.3716	797.70551	35.78220	49.4385

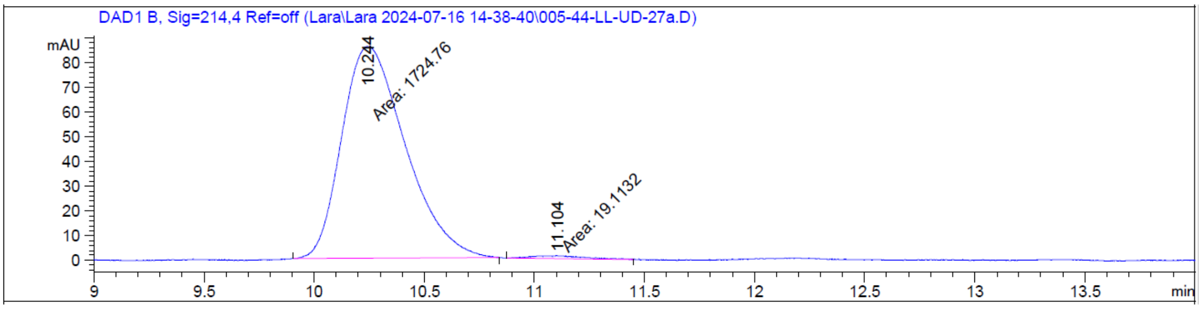

Peak #	RetTime [min]	Type	Width [min]	Area [mAU*s]	Height [mAU]	Area %
1	10.244	MM	0.3349	1724.75854	85.84147	98.9040
2	11.104	MM	0.2717	19.11324	1.17237	1.0960

(2*S*,3*R*)-2,3-difluoro-6-phenyl-*N*-((*S*)-1-phenylethyl)hexanamide (7ag)

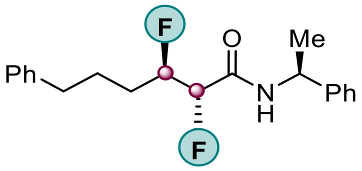

A modified procedure **GP2** was followed with 2-bromo-2-fluoro-*N*-((*S*)-1-phenylethyl)acetamide (**6g**) (26 mg, 0.10 mmol, 1.0 equiv.), (*Z*)-(4-fluorobut-3-en-1-

yl)benzene (30 mg, 0.20 mmol, 2.0 equiv.) and KI (16.6 mg, 0.1 mmol, 1 equiv.). Automated flash column chromatography (10 g SiO_2, gradient elution: hexane to 10% EtOAc in hexane) afforded the desired product (-) **7ag** as a white solid (22 mg, 66%).

^{1}H NMR (400 MHz, $CDCl_3$) δ 7.39 – 7.07 (m, 10H), 6.69 – 6.43 (m, 1H), 5.24 – 4.76 (m, 3H), 2.57 (t, J = 7.6 Hz, 2H), 1.93 – 1.72 (m, 2H), 1.70 – 1.60 (m, 1H), 1.53 (d, J = 6.9 Hz, 3H), 1.48 – 1.32 (m, 1H).

^{13}C NMR (101 MHz, $CDCl_3$) δ 165.07 (dd, $^2J_{C–F}$ = 19.1, $^3J_{C–F}$ = 10.0 Hz), 142.29, 141.59, 128.81, 128.38, 128.40, 127.66, 126.02, 125.93, 92.52 (dd, $^1J_{C–F}$ = 177.3, $^2J_{C–F}$ = 19.6 Hz), 91.94 (dd, $^1J_{C–F}$ = 194.0, $^2J_{C–F}$ = 22.5 Hz), 48.63, 35.35, 28.06 (dd, $^2J_{C–F}$ = 21.4, $^3J_{C–F}$ = 5.8 Hz), 26.77 (d, $^3J_{C–F}$ = 3.3 Hz), 21.66.

^{19}F NMR (377 MHz, $CDCl_3$) δ -192.54 (dddt, J = 49.0, 36.6, 18.3, 13.1 Hz), -200.75 (dddd, J = 49.4, 25.2, 12.7, 4.0 Hz).

HRMS (nanochip-ESI/LTQ-Orbitrap) m/z: $[M + H]^+$ Calcd for $C_{20}H_{24}F_2NO^+$ 332.1820; Found 332.1820.

$[\alpha]_D^{23}$ = -50.0 (c = 1.0 in $CHCl_3$).

The enantiomeric excess (98%) and diastereomeric ratio (97:3) was determined by ^{19}F-NMR analysis of the crude reaction mixture.

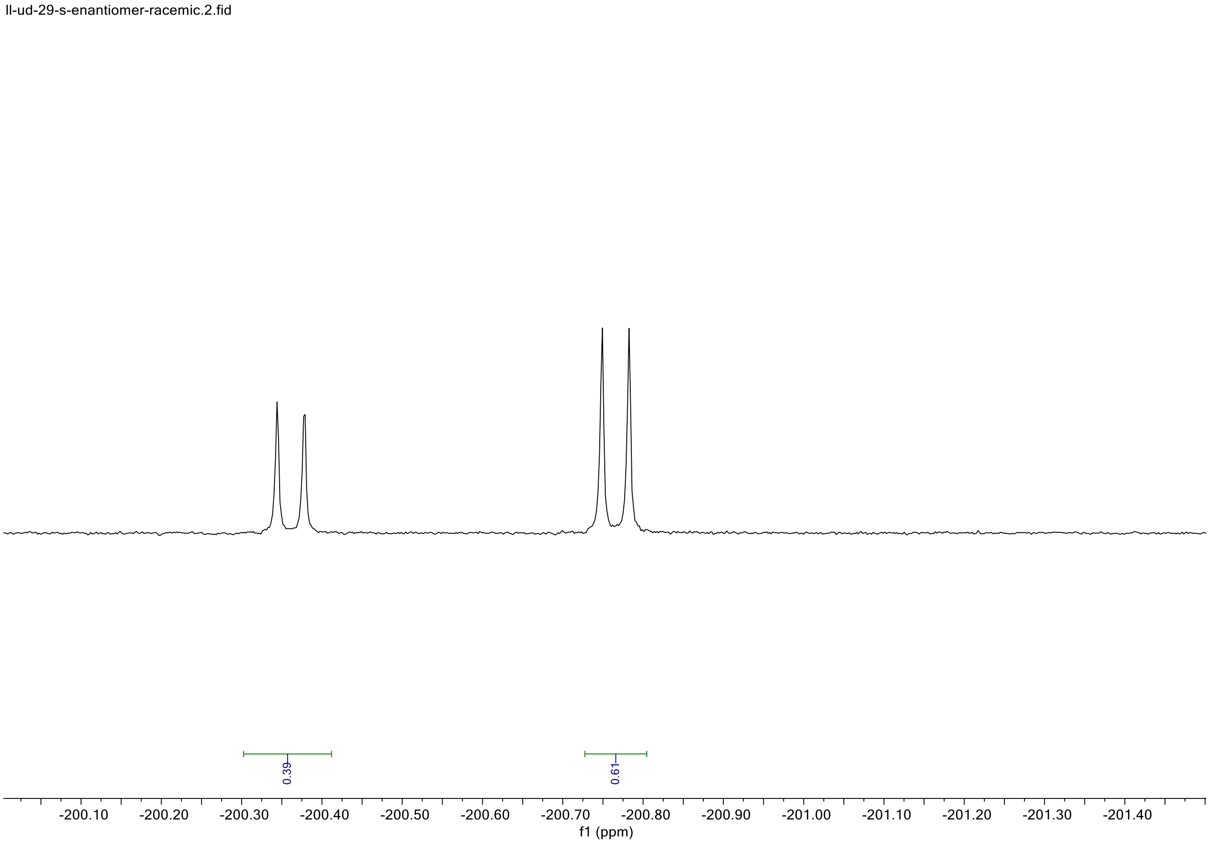

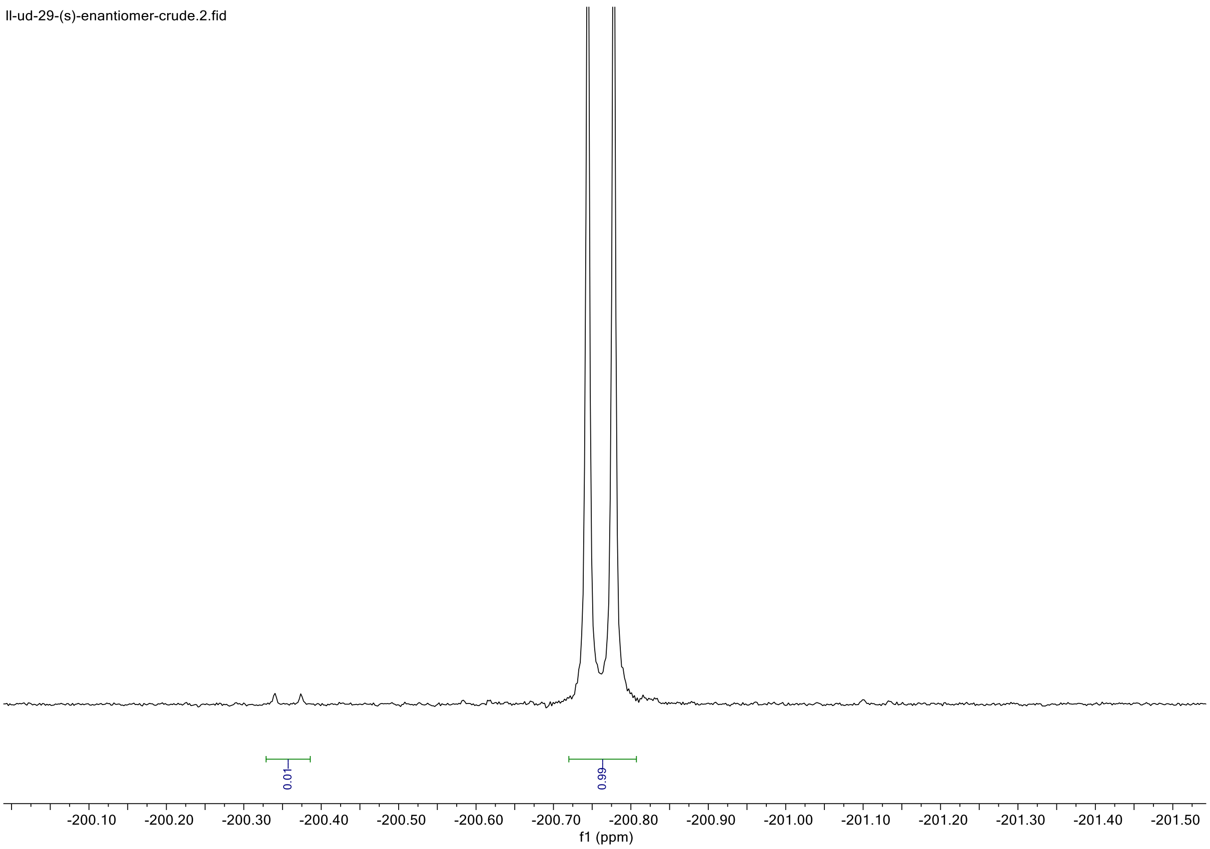

(2*S*,3*R*)-2,3-Difluoro-6-phenyl-*N*-((*R*)-1-phenylethyl)hexanamide (*epimer*-7ag)

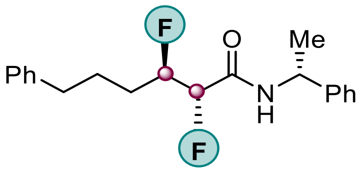

A modified procedure **GP2** was followed with 2-bromo-2-fluoro-*N*-((*R*)-1-phenylethyl)acetamide (*ent*-**6g**) (26 mg, 0.10 mmol, 1.0 equiv.), (*Z*)-(4-fluorobut-3-en-1-yl)benzene (30 mg, 0.20 mmol, 2.0 equiv.) and KI (16.6 mg, 0.1 mmol, 1 equiv.). Automated flash column chromatography (10 g SiO_2, gradient elution: hexane to 10% EtOAc in hexane) afforded the desired product (+)-*epimer*-**7ag** as a white solid (20.8 mg, 63%).

^{1}H NMR (400 MHz, $CDCl_3$) δ 7.43 – 7.13 (m, 10H), 6.55 (d, *J* = 8.2 Hz, 1H), 5.20 – 4.82 (m, 3H), 2.68 (t, *J* = 7.5 Hz, 2H), 1.93 (ddddd, *J* = 17.8, 15.0, 12.4, 8.1, 4.8 Hz, 2H), 1.81 – 1.68 (m, 1H), 1.64 – 1.55 (m, 1H), 1.48 (d, *J* = 6.9 Hz, 3H).

^{13}C NMR (101 MHz, $CDCl_3$) δ 165.01 (dd, $^{2}J_{C–F}$ = 19.5, $^{3}J_{C–F}$ = 9.9 Hz), 142.07, 141.59, 128.85, 128.44, 128.42, 127.78, 126.19, 125.97, 92.52 (dd, $^{1}J_{C–F}$ = 177.4, $^{2}J_{C–F}$ = 19.8 Hz), 91.91 (dd, $^{1}J_{C–F}$ = 194.2, $^{2}J_{C–F}$ = 22.9 Hz), 48.65, 35.32, 28.10 (dd, $^{2}J_{C–F}$ = 21.7, $^{3}J_{C–F}$ = 6.1 Hz) 26.84 (d, $^{3}J_{C–F}$ = 3.1 Hz) 21.55.

^{19}F NMR (376 MHz, $CDCl_3$) δ -192.40 – -193.47 (m), -200.35 (dddd, *J* = 49.8, 25.5, 12.8, 4.1 Hz).

HRMS (nanochip-ESI/LTQ-Orbitrap) m/z: [M + H]$^+$ Calcd for $C_{20}H_{24}F_2NO^+$ 332.1820; Found 332.1824.

$[\alpha]_D^{23}$ = 39.1 (c = 0.5 in $CHCl_3$).

The enantiomeric excess (94%) and diastereomeric ratio (95:5) was determined by ^{19}F-NMR analysis of the crude reaction mixture.

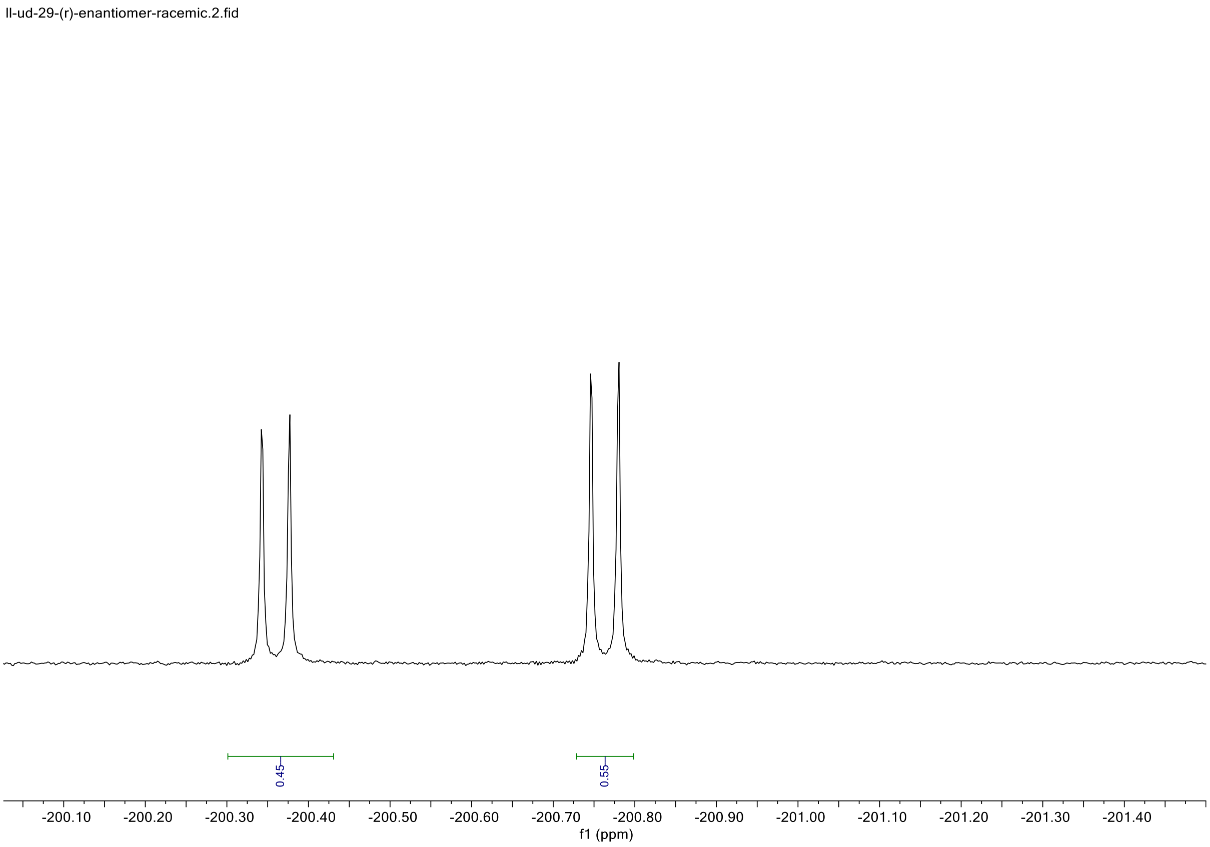

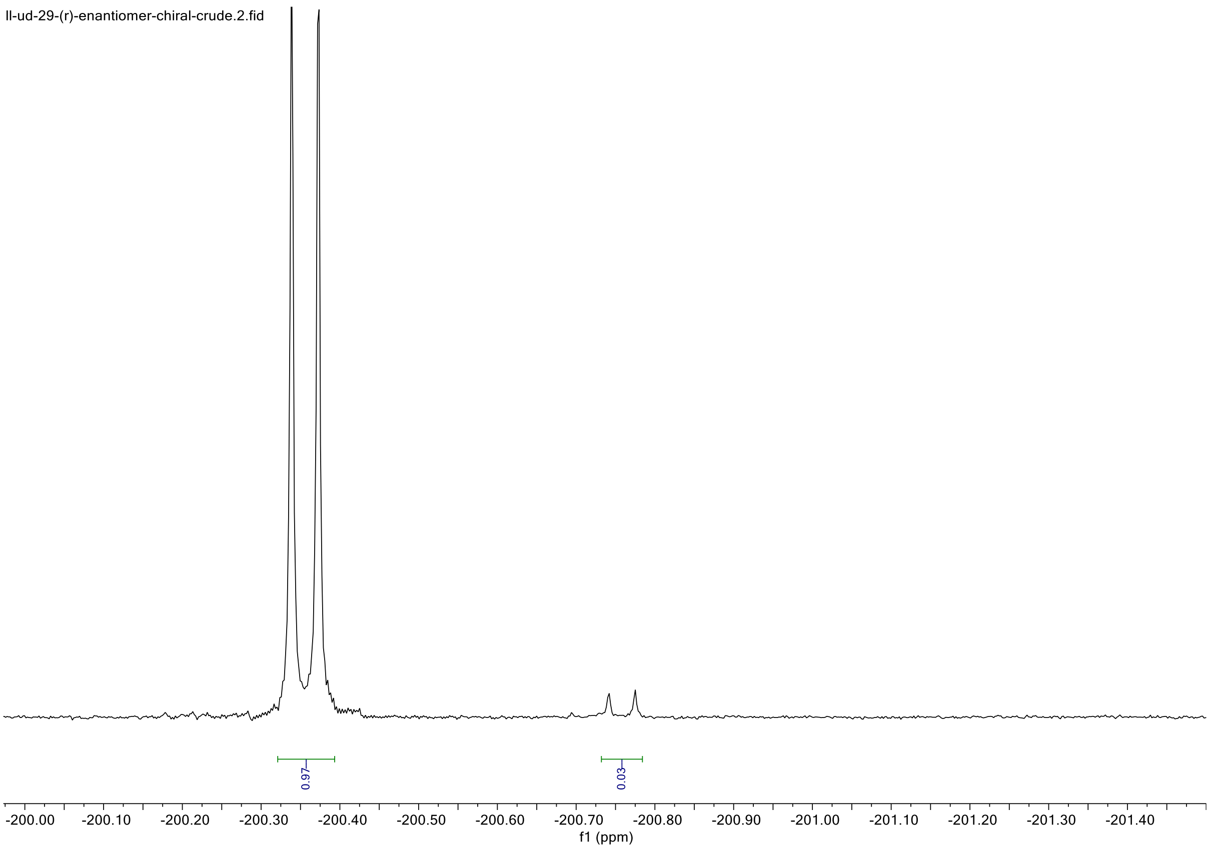
ll-ud-29-(r)-enantiomer-chiral-crude.2.fid
0.97
0.03
-200.00 -200.10 -200.20 -200.30 -200.40 -200.50 -200.60 -200.70 -200.80 -200.90 -201.00 -201.10 -201.20 -201.30 -201.40
f1 (ppm)

6. Synthetic Applications

(4*R*,5*S*)-6-Amino-4-fluoro-5-methyl-6-oxohexyl 4-(N,N-dipropylsulfamoyl)benzoate (8)

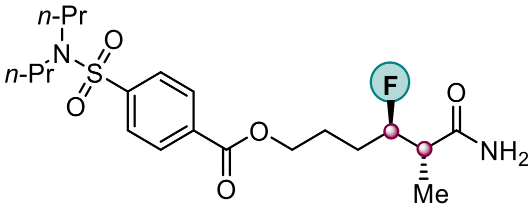

A modified procedure **GP2** was followed with 2-bromopropanamide (**4k**) (15.2 mg, 0.10 mmol, 1.0 equiv.), (*Z*)-4-fluorobut-3-en-1-yl 4-(*N*,*N*-dipropylsulfamoyl)benzoate (**S8**) (71.5 mg, 0.20 mmol, 2.0 equiv.), $NiCl_2{\cdot}DME$ (3.3 mg, 0.015 mmol, 0.15 equiv.), **L8** (0.023 mmol, 0.23 equiv.) and KI (16.6 mg, 0.1 mmol, 1 equiv.). Automated flash column chromatography (10 g SiO_2, gradient elution: hexane to 60% EtOAc in hexane) afforded the desired product (+) **8** as a yellow oil (20.2 mg, 47%) in 97:3 diastereomeric ratio.

^{1}H NMR (400 MHz, $CDCl_3$) δ 8.19 – 8.06 (m, 2H), 7.92 – 7.80 (m, 2H), 5.68 (bs, 1H), 5.54 (bs, 1H), 4.83 – 4.53 (m, 1H), 4.39 (td, *J* = 6.3, 3.3 Hz, 2H), 3.14 – 3.04 (m, 4H), 2.64 – 2.49 (m, 1H), 2.09 – 1.73 (m, 4H), 1.56 (dd, *J* = 15.1, 7.5 Hz, 4H), 1.22 (d, *J* = 7.1 Hz, 3H), 0.86 (t, *J* = 7.4 Hz, 6H).

^{13}C NMR (101 MHz, $CDCl_3$) δ 175.22 (d, $^{3}J_{C–F}$ = 2.6 Hz), 165.21, 144.33, 133.48, 130.20, 127.05, 94.62 (d, $^{1}J_{C–F}$ = 172.0 Hz), 65.01, 49.95, 45.61 (d, $^{2}J_{C–F}$ = 20.2 Hz), 29.00 (d, $^{2}J_{C–F}$ = 21.3 Hz), 24.46 (d, $^{3}J_{C–F}$ = 3.7 Hz), 21.95, 13.76 (d, $^{3}J_{C–F}$ = 6.6 Hz), 11.17.

^{19}F{1H} NMR (376 MHz, $CDCl_3$) δ -183.58.

HRMS (APCI/QTOF) m/z: $[M + Na]^+$ Calcd for $C_{20}H_{31}FN_2NaO_5S^+$ 453.1830; Found 453.1824.

$[\alpha]_D^{23}$ = +2.4 (c = 0.5 in $CHCl_3$).

HPLC: The enantiomeric excess (99%) was determined *via* HPLC analysis using a CHIRALCEL® OD-H column, with hexane:isopropanol = 60:40 at a flow rate 1.0 mL/min detected at 254 nm wavelength. Retention time: t_{major} = 9.8 min and t_{minor} = 6.7 min. Diastereomeric ratio (98:2) was determined by ^{19}F-NMR of the crude reaction mixture.

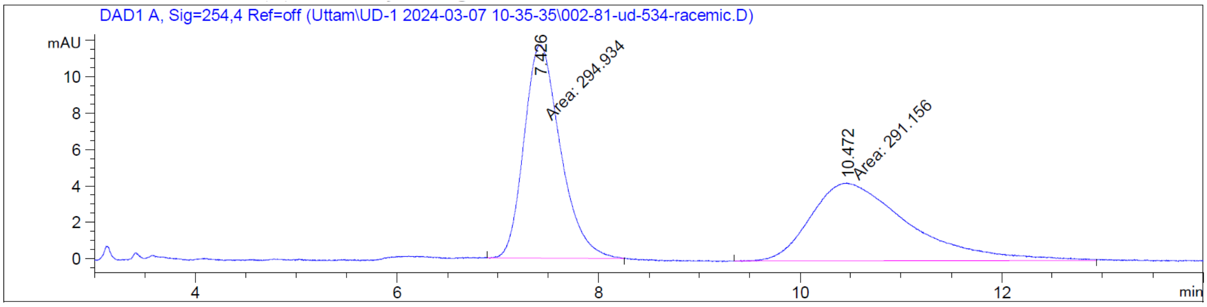

Peak #	RetTime [min]	Type	Width [min]	Area [mAU*s]	Height [mAU]	Area %
1	7.426	MM	0.4184	294.93396	11.74978	50.3223
2	10.472	MM	1.1314	291.15619	4.28905	49.6777

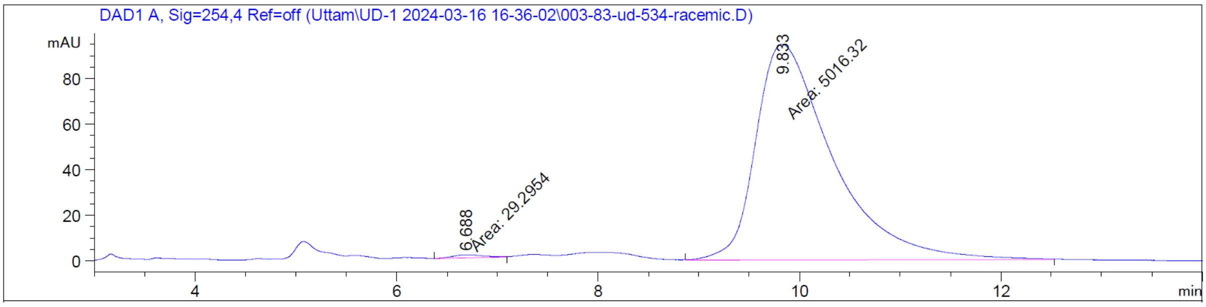

Peak #	RetTime [min]	Type	Width [min]	Area [mAU*s]	Height [mAU]	Area %
1	6.688	MM	0.3572	29.29542	1.36685	0.5806
2	9.833	MM	0.8837	5016.31689	94.60312	99.4194

(4*R*,5*S*)-4-Fluoro-5-methyl-6-oxo-6-(phenylamino)hexyl-(*R*)-4-((5*S*,8*R*,9*S*,10*S*,13*R*,14*S*,17*R*)-10,13-dimethyl-3,7,12-trioxohexadecahydro-1*H*-cyclopenta[*a*]phenanthren-17-yl)pentanoate (9)

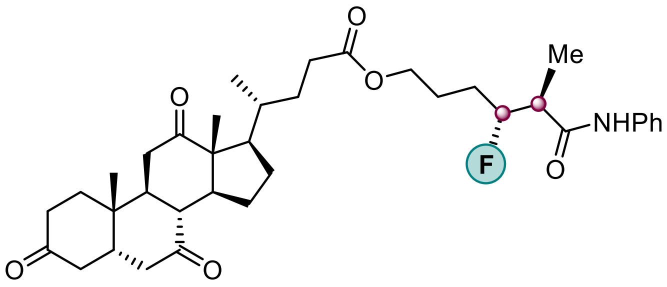

A modified procedure **GP2** was followed with 2-iodo-*N*-phenylpropanamide (**4b**) (13.8 mg, 0.05 mmol, 1.0 equiv.), (*Z*)-pent-3-en-1-yl (*R*)-4-((5*S*,8*R*,9*S*,10*S*,13*R*,14*S*,17*R*)-10,13-dimethyl-3,7,12-trioxohexadecahydro-1*H*-cyclopenta[*a*]phenanthren-17-yl)pentanoate (**S9**) (35.8 mg, 0.10 mmol, 2.0 equiv.), $NiCl_2$·DME (0.0075 mmol, 0.15 equiv.), ligand **L8** (0.0112 mmol, 0.112 equiv.). Automated flash column chromatography (10 g SiO_2, gradient elution: hexane to 80% EtOAc in hexane) afforded the desired product (+) **9** as a yellow oil (16.3 mg, 52%) in >99:1 diastereomeric ratio.

^{1}H NMR (400 MHz, $CDCl_3$) δ ^{1}H NMR (400 MHz, $CDCl_3$) δ 7.57 – 7.52 (m, 2H), 7.51 – 7.41 (m, 1H), 7.37 – 7.31 (m, 2H), 7.16 – 7.10 (m, 1H), 5.01 – 4.45 (m, 1H), 4.18 – 4.06 (m, 2H), 2.91 (ddd, *J* = 18.6, 12.9, 6.2 Hz, 3H), 2.70 – 2.54 (m, 1H), 2.48 – 2.19 (m, 10H), 2.16 – 2.12 (m, 1H), 2.07 – 2.01 (m, 3H), 2.00 – 1.96 (m, 1H), 1.91 – 1.75 (m, 6H), 1.62 (t, *J* = 7.2 Hz, 1H), 1.47 – 1.30 (m, 4H), 1.29 (dd, *J* = 7.1, 2.6 Hz, 5H), 1.09 (d, *J* = 1.7 Hz, 3H), 0.87 (d, *J* = 6.6 Hz, 3H).

^{13}C NMR (101 MHz, $CDCl_3$) (one resonance is missing due to overlap): δ 212.02, 209.06, 208.73, 174.09, 171.25 (d, $^{3}J_{C–F}$ = 1.8 Hz), 137.73, 129.02, 124.47, 119.98, 95.01 (d, $^{1}J_{C–F}$ = 171.3 Hz), 63.65, 56.91, 51.76, 49.00, 47.21 (d, $^{2}J_{C–F}$ = 20.5 Hz), 46.85, 45.59 (d, $^{4}J_{C–F}$ = 4.8 Hz), 44.99, 42.80, 38.65, 36.49, 36.02, 35.48, 35.28, 31.42, 30.41, 29.10 (d, $^{2}J_{C–F}$ = 21.3 Hz), 27.60, 25.14, 24.32 (d, $^{3}J_{C–F}$ = 3.7 Hz), 21.92, 18.65, 13.79 (d, $^{3}J_{C–F}$ = 7.0 Hz), 11.86.

$^{19}F\{1H\}$ NMR (376 MHz, $CDCl_3$) δ -182.07.

HRMS (ESI/QTOF) m/z: $[M + H]^+$ Calcd for $C_{37}H_{51}FNO_6^+$ 624.3695; Found 624.3705.

$[\alpha]_D^{23}$ = +11.4 (c = 0.40 in $CHCl_3$).

HPLC: The enantiomeric excess (95%) was determined *via* HPLC analysis using a CHIRALCEL® IA column, with hexane:isopropanol = 60:40 at a flow rate 1.2 mL/min detected at 254 nm wavelength. Retention time: t_{major} = 11.2 min and t_{minor} = 9.4 min. Diastereomeric ratio (99:1) was determined by ^{19}F-NMR of the crude reaction mixture.

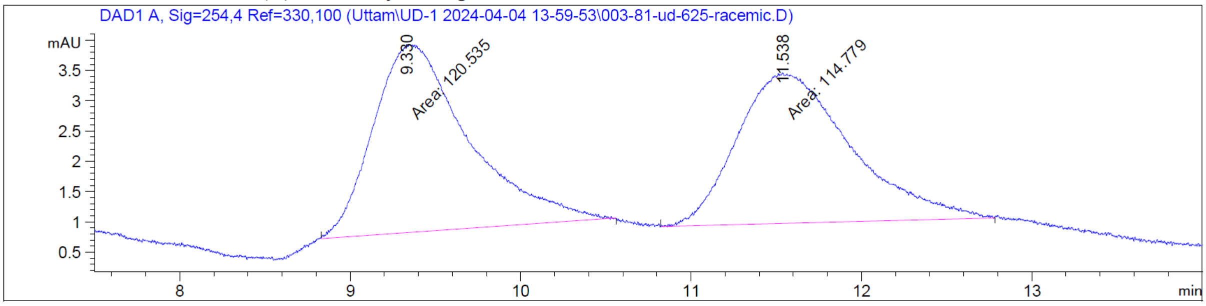

Peak #	RetTime [min]	Type	Width [min]	Area [mAU*s]	Height [mAU]	Area %
1	9.330	MM	0.6478	120.53460	3.10106	51.2229
2	11.538	MM	0.7713	114.77924	2.48035	48.7771

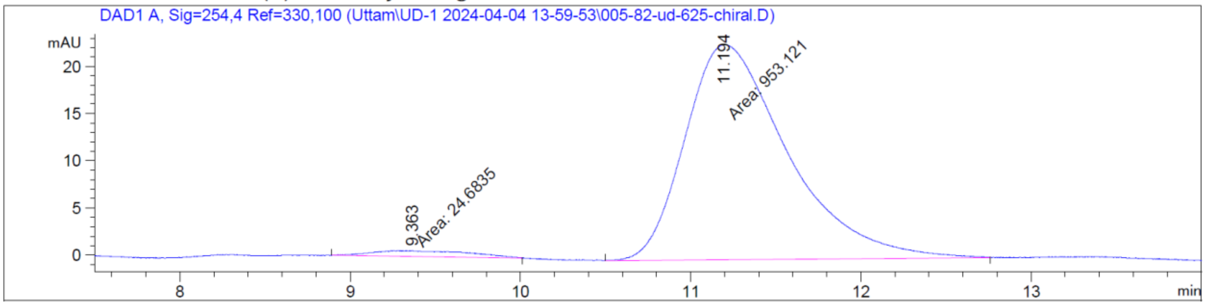

Peak #	RetTime [min]	Type	Width [min]	Area [mAU*s]	Height [mAU]	Area %
1	9.363	MM	0.6784	24.68353	6.06407e-1	2.5244
2	11.194	MM	0.6975	953.12128	22.77501	97.4756

***tert*-Butyl 2-((4*R*,6*S*)-6-(2-((*S*)-3-((*R*)-1-fluoro-4-phenylbutyl)-2-oxopyrrolidin-1-yl)ethyl)-2,2-dimethyl-1,3-dioxan-4-yl)acetate (10)**

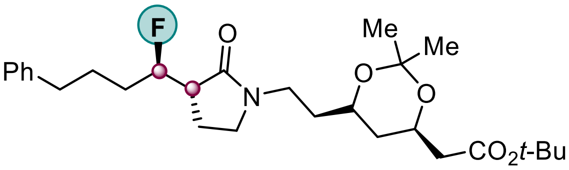

Prepared according to **GP1** with *tert*-butyl 2-((4*R*,6*S*)-6-(2-(3-bromo-2-oxopyrrolidin-1-yl)ethyl)-2,2-dimethyl-1,3-dioxan-4-yl)acetate (**S10**) (42.0 mg, 0.10 mmol, 1.0 equiv.), (*Z*)-(4-fluorobut-3-en-1-yl)benzene (30.0 mg, 0.20 mmol, 2.0 equiv.). Automated flash column chromatography (10 g SiO_2, gradient elution: hexane to 50% EtOAc in hexane) afforded the desired product (+) **10** as a yellow oil (37.0 mg, 75%) in 97:3 diastereomeric ratio.

^{1}H NMR (400 MHz, $CDCl_3$) δ 7.31 – 7.23 (m, 2H), 7.19 (dd, *J* = 7.6, 2.3 Hz, 3H), 4.78 (ddt, *J* = 48.1, 9.8, 3.3 Hz, 1H), 4.36 – 4.15 (m, 1H), 3.87 (td, *J* = 9.1, 4.8 Hz, 1H), 3.45 – 3.24 (m, 4H), 2.80 (dddd, *J* = 24.1, 9.8, 6.6, 3.4 Hz, 1H), 2.66 (t, *J* = 7.6 Hz, 2H), 2.46 – 2.26 (m, 2H),

2.23 – 2.11 (m, 1H), 2.02 (ddd, J = 13.7, 10.6, 6.0 Hz, 2H), 1.85 (ddd, J = 12.9, 9.0, 5.9 Hz, 1H), 1.75 – 1.58 (m, 5H), 1.45 (s, 9H), 1.43 (s, 3H), 1.36 (s, 3H), 1.24 – 1.14 (m, 1H).

^{13}C NMR (101 MHz, $CDCl_3$) δ 172.46 (d, $^3J_{C–F}$ = 9.9 Hz), 170.22, 142.08, 128.42, 128.34, 125.81, 98.76, 94.79 (d, $^1J_{C–F}$ = 171.3 Hz), 80.63, 66.91, 66.14, 45.86, 45.85 (d, $^2J_{C–F}$ = 20.1 Hz), 42.70, 39.26, 36.44, 35.56, 33.79, 30.94 (d, $^2J_{C–F}$ = 20.9 Hz), 30.11, 28.12, 27.56 (d, $^3J_{C-F}$ = 3.7 Hz), 21.11 (d, $^3J_{C–F}$ = 1.8 Hz), 19.69.

^{19}F{1H} NMR (376 MHz, $CDCl_3$) δ -190.02.

HRMS (APCI/QTOF) m/z: $[M + Na]^+$ Calcd for $C_{28}H_{42}FNNaO_5^+$ 514.2939; Found 514.2952.

$[\alpha]_D^{23}$ = +10.8 (c = 1.00 in $CHCl_3$).

HPLC: The enantiomeric excess (99%) was determined *via* HPLC analysis using a CHIRALCEL® IF column, with hexane:isopropanol = 90:10 at a flow rate 1.0 mL/min detected at 214 nm wavelength. Retention time: t_{major} = 17.9 min and t_{minor} = 21.7 min. Diastereomeric ratio (97:3) was determined by ^{19}F-NMR of the crude reaction mixture.

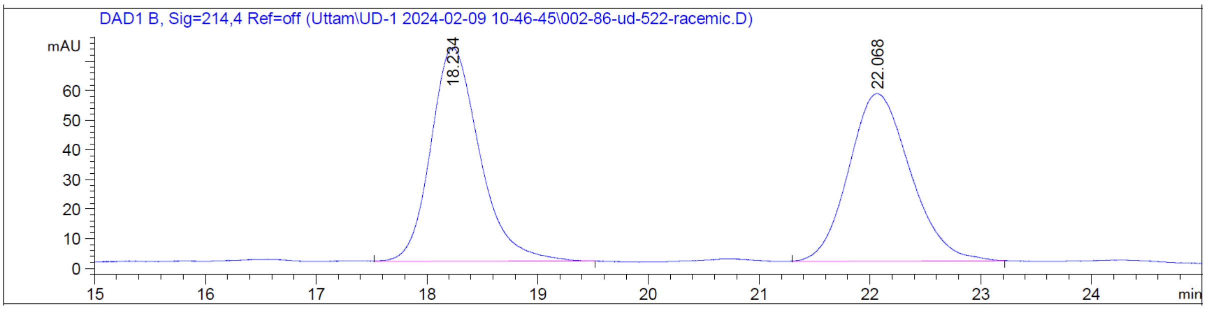

Peak #	RetTime [min]	Type	Width [min]	Area [mAU*s]	Height [mAU]	Area %
1	18.234	BB	0.3820	2208.10278	72.16629	50.2814
2	22.068	BB	0.4534	2183.38989	56.55733	49.7186

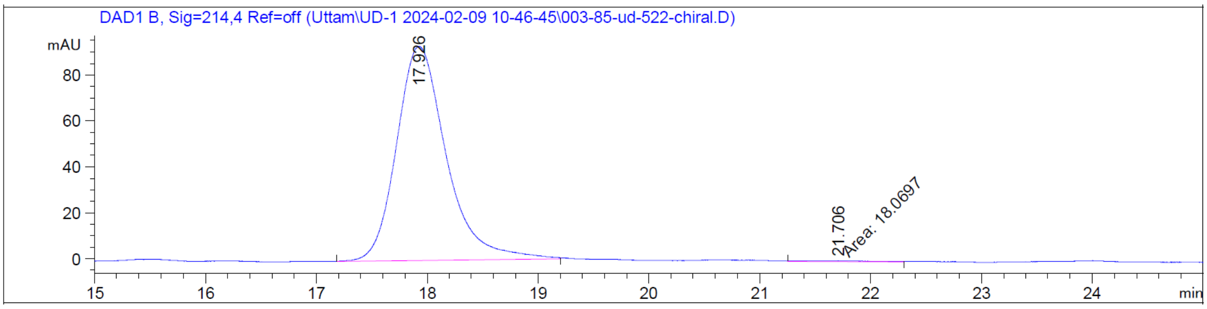

Peak #	RetTime [min]	Type	Width [min]	Area [mAU*s]	Height [mAU]	Area %
1	17.926	BV	0.3661	2908.83984	93.20066	99.3826
2	21.706	MM	0.6708	18.06967	4.48926e-1	0.6174

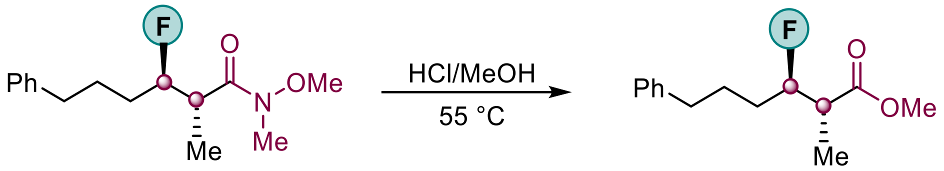

Methyl (2*S*,3*R*)-3-fluoro-2-methyl-6-phenylhexanoate (12)

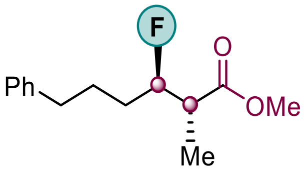

A solution of (2*S*,3*R*)-3-fluoro-*N*-methoxy-*N*,2-dimethyl-6-phenylhexanamide **5al** (12 mg, 44.9 µmol, 1 equiv., 99% e.e., >99:1 d.r.) in MeOH (1.0 mL) was added to a flame-dried 8 mL vial equipped with a magnetic stir bar under a nitrogen atmosphere. The flask was cooled to 0 °C for 10 min. Two drops of hydrochloric acid (36.5~38% w/w, aqueous solution) was added to the mixture *via* 1 mL of syringe, and the mixture was allowed to stir for 36 hours at 55 °C. The reaction mixture was quenched with brine solution (2 mL) and the resulting mixture was separated into two phases. The aqueous layer was extracted with EtOAc (3 x 5 mL), and the combined organic layer was dried over anhydrous Na_2SO_4, filtered, and concentrated under reduced pressure. The obtained crude product was purified by preparative TLC to afford the desired product methyl (2*S*,3*R*)-3-fluoro-2-methyl-6-phenylhexanoate (+) **12** as yellow liquid (7.5 mg, 70% yield, 99% ee).

^{1}H NMR (400 MHz, $CDCl_3$) δ 7.34 – 7.23 (m, 2H), 7.23 – 7.10 (m, 3H), 4.69 (dtd, J = 47.8, 7.6, 4.6 Hz, 1H), 3.70 (s, 3H), 2.84 – 2.56 (m, 3H), 1.93 – 1.50 (m, 4H), 1.13 (d, J = 7.1 Hz, 3H).

^{13}C NMR (101 MHz, $CDCl_3$) δ 174.03 (d, $^3J_{C\text{–}F}$ = 5.9 Hz), 141.85, 128.40, 128.37, 125.89, 94.35 (d, $^1J_{C\text{–}F}$ = 172.4 Hz), 51.89, 44.38 (d, $^2J_{C\text{–}F}$ = 21.7 Hz), 35.45, 31.29 (d, $^2J_{C\text{–}F}$ = 21.3 Hz), 26.53 (d, $^3J_{C\text{–}F}$ = 3.2 Hz), 12.70 (d, $^3J_{C\text{–}F}$ = 6.7 Hz).

^{19}F{1H} NMR (376 MHz, $CDCl_3$) δ -181.77.

HRMS (APCI/QTOF) m/z: $[M + Na]^+$ Calcd for $C_{14}H_{19}FNaO_2^+$ 261.1261; Found 261.1259.

$[\alpha]_D^{23}$ = +2.5 (c = 0.67 in $CHCl_3$).

HPLC: The enantiomeric excess (99%) was determined *via* HPLC analysis using a CHIRALCEL® IF column, with hexane:isopropanol = 98:2 at a flow rate 1.0 mL/min detected at 210 nm wavelength. Retention time: t_{major} = 6.2 min and t_{minor} = 7.6 min. Diastereomeric ratio (>99:1) was determined by GC-MS of the crude reaction mixture.

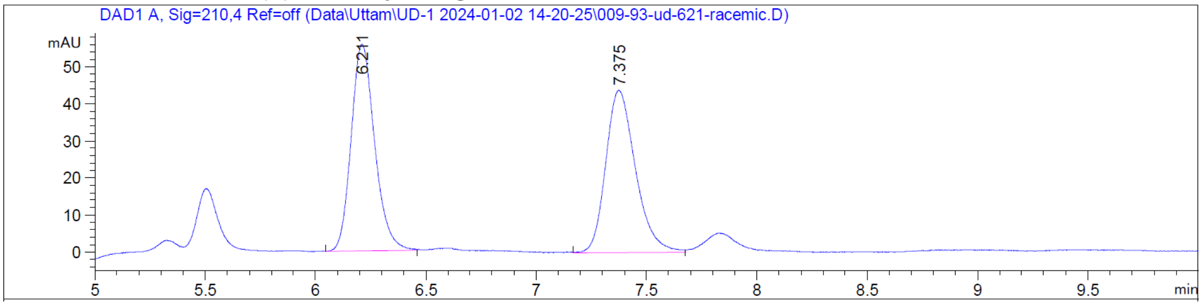

Peak #	RetTime [min]	Type	Width [min]	Area [mAU*s]	Height [mAU]	Area %
1	6.211	VV	0.1125	413.74588	55.82203	50.2095
2	7.375	VV	0.1388	410.29361	43.81087	49.7905

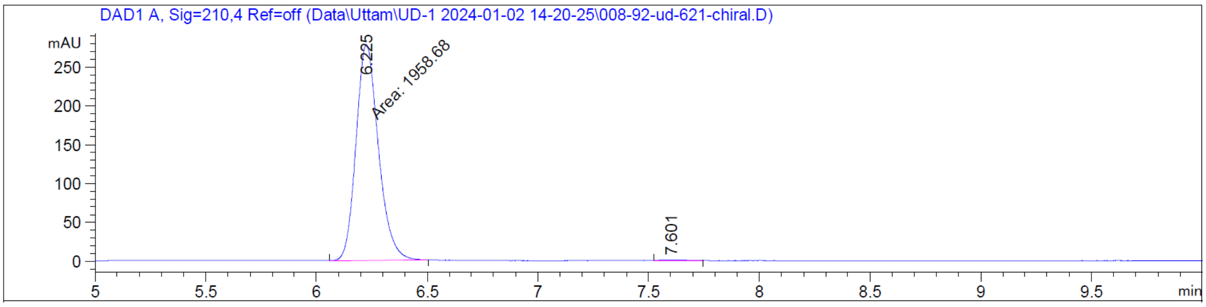

Peak #	RetTime [min]	Type	Width [min]	Area [mAU*s]	Height [mAU]	Area %
1	6.225	MM	0.1174	1958.67542	278.15308	99.4782
2	7.601	VV	0.0956	10.27361	1.28058	0.5218

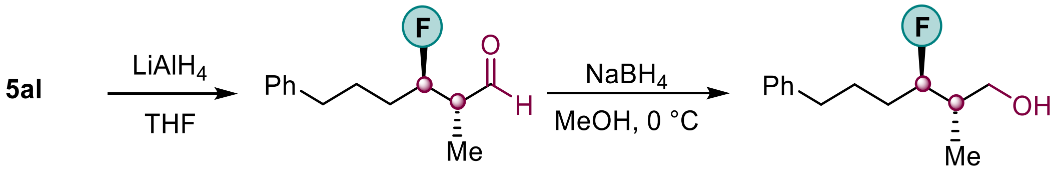

(2*S*,3*R*)-3-Fluoro-2-methyl-6-phenylhexan-1-ol (13)

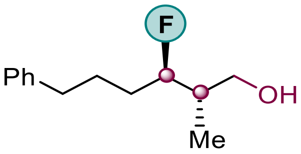

The solution of (2*S*,3*R*)-3-fluoro-*N*-methoxy-*N*,2-dimethyl-6-phenylhexanamide (**5al**) (20 mg, 74.8 μmol, 1 equiv., 99% e.e., >99:1 d.r.) in anhydrous THF (1 mL) was added to a flame-dried 8 mL vial equipped with a magnetic stir bar under a nitrogen atmosphere. The flask was cooled to -78 °C for 10 min. Lithium aluminium hydride (2.4 M, 34.3 μL, 82.3 μmol, 1.1 eq.) was

slowly added to the mixture *via* a syringe, and the mixture was allowed to stir for 1 h at room temperature. The reaction mixture was quenched with aqueous saturated NH_4Cl solution (1 mL) at -78 °C and the flask was allowed to slowly warm to room temperature. The resulting mixture was separated into two phases. The aqueous layer was extracted with EtOAc (3 x 5 mL), and the combined organic layer was dried over anhydrous Na_2SO_4, filtered, and concentrated under reduced pressure. The obtained crude product was found to be unstable on silica. It was directly used in the next step. The obtained crude was dissolved in MeOH (0.5 mL) and sodium borohydride (3.11 mg, 82.3 μmol, 1.1 eq.) was added at 0 °C. Then the reaction was stirred at 0 °C and was monitored by TLC. After full consumption of the starting material, the reaction mixture was concentrated under reduced pressure using rotary evaporation. The resulting mixture was separated into two phases. The aqueous layer was extracted with EtOAc (3 x 5 mL) and the combined organic layers were dried over anhydrous Na_2SO_4, filtered, and concentrated under reduced pressure. The residue was purified by automated flash column chromatography (10 g SiO_2, gradient elution: hexane to 15% EtOAc in hexane) which afforded the desired product (+) **13** as a yellow oil (11.5 mg, 73%) in >99:1 diastereomeric ratio.

^{1}H NMR (400 MHz, $CDCl_3$) δ 7.35 – 7.24 (m, 2H), 7.23 – 7.06 (m, 3H), 4.63 – 4.22 (m, 1H), 3.66 (dt, *J* = 11.0, 5.6 Hz, 2H), 2.66 (tt, *J* = 11.4, 6.0 Hz, 2H), 2.02 – 1.60 (m, 5H), 0.92 (d, *J* = 7.0 Hz, 3H).

^{13}C NMR (101 MHz, $CDCl_3$) δ 142.05, 128.42, 128.37, 125.86, 97.06 (d, $^1J_{C–F}$ = 168.4 Hz), 65.06 (d, $^3J_{C–F}$ = 5.9 Hz), 39.91 (d, $^2J_{C–F}$ = 18.0 Hz), 35.62, 32.18 (d, $^2J_{C–F}$ = 21.3 Hz), 26.79 (d, $^3J_{C–F}$ = 3.3 Hz), 13.04 (d, $^3J_{C–F}$ = 7.3 Hz).

^{19}F{1H} NMR (376 MHz, $CDCl_3$) δ -185.41.

HRMS (ESI/QTOF) m/z: $[M + Na]^+$ Calcd for $C_{13}H_{19}FNaO^+$ 233.1312; Found 233.1311.

$[\alpha]_D^{23}$ = +12.8 (c = 0.40 in $CHCl_3$).

HPLC: The enantiomeric excess (99%) was determined *via* HPLC analysis using a CHIRALCEL® OD-H column, with hexane:isopropanol = 90:10 at a flow rate 1.0 mL/min detected at 214 nm wavelength. Retention time: t_{major} = 7.3 min and t_{minor} = 6.6 min. Diastereomeric ratio (>99:1) was determined by GC-MS of the crude reaction mixture.

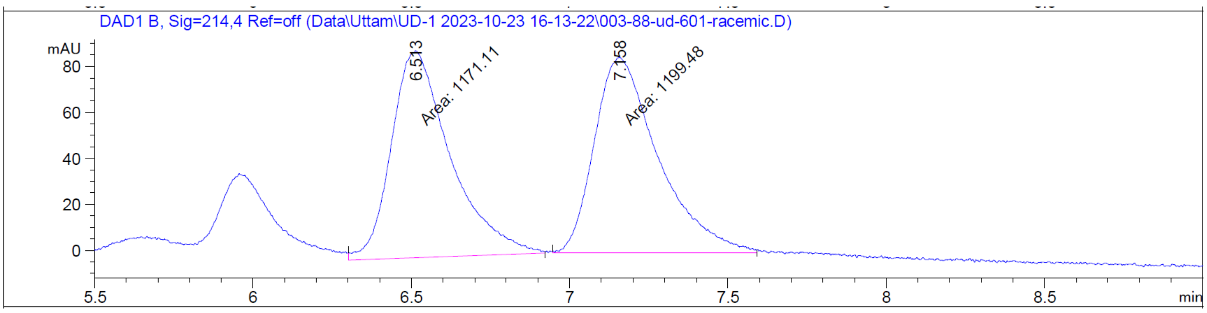

Peak #	RetTime [min]	Type	Width [min]	Area [mAU*s]	Height [mAU]	Area %
1	6.513	MM	0.2164	1171.11450	90.17833	49.4017
2	7.158	MM	0.2347	1199.48242	85.16801	50.5983

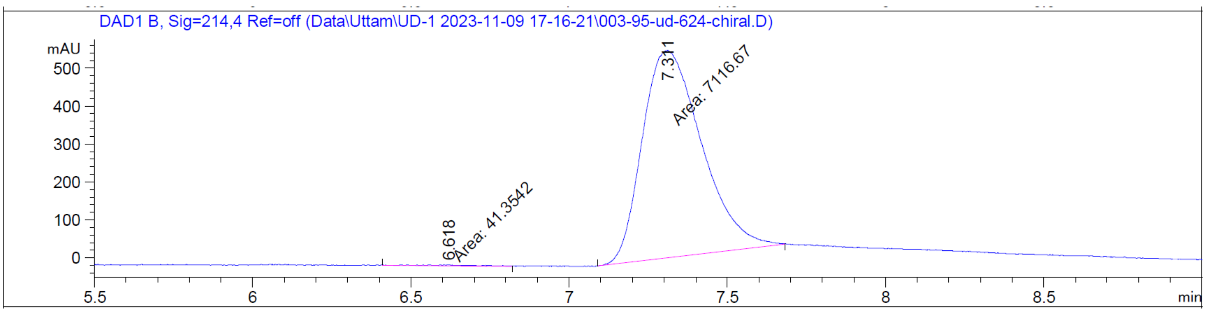

Peak #	RetTime [min]	Type	Width [min]	Area [mAU*s]	Height [mAU]	Area %
1	6.618	MM	0.2119	41.35419	3.25207	0.5777
2	7.311	MM	0.2170	7116.66553	546.47852	99.4223

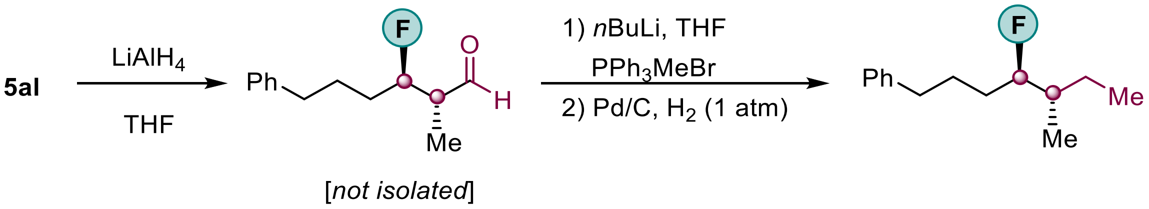

The solution of (2*S*,3*R*)-3-fluoro-*N*-methoxy-*N*,2-dimethyl-6-phenylhexanamide **5al** (30 mg, 112.2 µmol, 1 equiv., 99% e.e., >99:1 d.r.) in anhydrous THF (1 mL) was added to a flame-dried 8 mL vial equipped with a magnetic stir bar under a nitrogen atmosphere. The vial was cooled to –78 °C for 10 min. Lithium aluminium hydride (2.4 M, 51.4 µL, 123.4 µmol, 1.1 eq.) was slowly added to the mixture via syringe, and the mixture was allowed to warm up and was stirred for 1 h at room temperature. The reaction mixture was then quenched with aqueous

saturated NH_4Cl solution (1 mL) at 0 °C, and the vial was allowed to slowly warm to room temperature. The resulting mixture was separated into two phases. The aqueous layer was extracted with EtOAc (3 x 5 mL), and the combined organic layers were dried over anhydrous Na_2SO_4, filtered, and concentrated under reduced pressure. The obtained crude product was found to be unstable on silica. It was therefore directly used in the next step. An oven-dried flask was charged with methyl(triphenyl)phosphonium bromide (120 mg, 336 µmol, 3 eq.), and THF (0.50 mL) was added under N_2 atmosphere. The resulting suspension was cooled to 0 °C, and *n*-butyllithium (2.5 M, 112 µL, 280 µmol, 2.5 eq.) was added dropwise. After the resulting orange solution was stirred for 30 min, the crude product from the previous step was dissolved in THF (0.25 mL) and added to the reaction mixture. The reaction was stirred at 0 °C until the reaction was completed (as monitored by TLC). It was then quenched with saturated aqueous NH_4Cl solution. The aqueous layer was extracted with ethyl acetate (3 × 2.0 mL) and the combined organic layers were washed with brine, dried over anhydrous Na_2SO_4 and concentrated under reduced pressure. The resulting residue was purified by automated flash column chromatography (10 g SiO_2, gradient elution: hexane to 5% EtOAc in hexane) to afford the desired product **11** as a yellow oil (18.5 mg, 80%).

{(4*R*,5*S*)-4-fluoro-5-methylhept-6-en-1-yl}benzene (11)

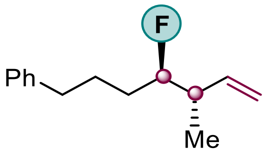

^{1}H NMR (400 MHz, $CDCl_3$) δ 7.34 – 7.25 (m, 2H), 7.19 (td, J = 5.7, 3.3 Hz, 3H), 5.78 (ddd, J = 16.8, 10.7, 7.8 Hz, 1H), 5.15 – 5.00 (m, 2H), 4.50 – 4.21 (m, 1H), 2.64 (t, J = 7.4 Hz, 2H), 2.49 – 2.29 (m, 1H), 1.84 (dq, J = 11.9, 8.1 Hz, 3H), 1.76 – 1.56 (m, 1H), 1.06 (d, J = 7.0 Hz, 3H).

^{13}C NMR (101 MHz, $CDCl_3$) δ 142.15, 138.75 (d, $^3J_{C–F}$ = 5.9 Hz), 128.40, 128.34, 125.81, 115.71, 96.82 (d, $^1J_{C–F}$ = 172.8 Hz), 42.23 (d, $^2J_{C–F}$ = 20.2 Hz), 35.66, 31.91 (d, $^2J_{C–F}$ = 21.3 Hz), 27.12 (d, $^3J_{C–F}$ = 4.0 Hz), 15.92 (d, $^3J_{C–F}$ = 4.8 Hz).

^{19}F{1H} NMR (376 MHz, $CDCl_3$) δ -187.59.

HRMS (Sicrit plasma/LTQ-Orbitrap) m/z: [M + H]+ Calcd for $C_{14}H_{20}F^+$ 207.1544; Found 207.1538.

Diastereomeric ratio (>99:1) was determined by GC-MS analysis of the crude reaction mixture.

{(4*R*,5*S*)-4-fluoro-5-methylheptyl}benzene (14)

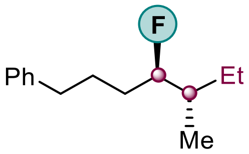

An oven-dried 8 mL vial equipped with a stirring bar was subjected to three vacuum/H_2 cycles to replace air inside with hydrogen gas. {(4*R*,5*S*)-4-fluoro-5-methylhept-6-en-1-yl}benzene **11** (12 mg, 58.2 μmol, 1 eq.), 10% Pd/C (10 wt %), and MeOH (2.0 mL) were then added to the vial and the resulting solution was vigorously stirred at room temperature with hydrogen balloon for 2 hour. After full conversion (as determined by GC-MS analysis), the reaction mixture was concentrated under reduced pressure. The residue was then purified by automated flash column chromatography (10 g SiO_2, gradient elution: hexane to 5% EtOAc in hexane) to afford the desired product (–) **14** as a yellow oil (10.7 mg, 88%).

^{1}H NMR (400 MHz, $CDCl_3$) δ 7.33 – 7.25 (m, 2H), 7.24 – 7.11 (m, 3H), 4.45 – 4.15 (m, 1H), 2.75 – 2.57 (m, 2H), 1.90 – 1.48 (m, 6H), 1.15 (ddt, *J* = 13.7, 8.6, 7.2 Hz, 1H), 0.94 – 0.85 (m, 6H).

^{13}C NMR (101 MHz, $CDCl_3$) δ 142.24, 128.41, 128.32, 125.77, 97.71 (d, $^1J_{C–F}$ = 169.8 Hz), 38.83 (d, $^2J_{C–F}$ = 19.1 Hz), 35.73, 31.32 (d, $^2J_{C–F}$ = 21.6 Hz), 27.17 (d, $^3J_{C–F}$ = 2.9 Hz), 24.44 (d, $^3J_{C–F}$ = 6.2 Hz), 14.47 (d, $^3J_{C–F}$ = 5.9 Hz), 11.37.

^{19}F{1H} NMR (376 MHz, $CDCl_3$) δ -184.93.

HRMS (Sicrit plasma/LTQ-Orbitrap) m/z: $[M + H]^+$ Calcd for $C_{14}H_{22}F^+$ 209.1700; Found 209.1695.

$[\alpha]_D^{23}$ = –1.6 (c = 0.45 in $CHCl_3$).

HPLC: The enantiomeric excess (98%) was determined *via* HPLC analysis using a CHIRALCEL® OJ-H column, with hexane:isopropanol = 99:1 at a flow rate 1.0 mL/min detected at 214 nm wavelength. Retention time: t_{major} = 4.4 min and t_{minor} = 5.1 min. Diastereomeric ratio (>99:1) was determined by GC-MS of the crude reaction mixture.

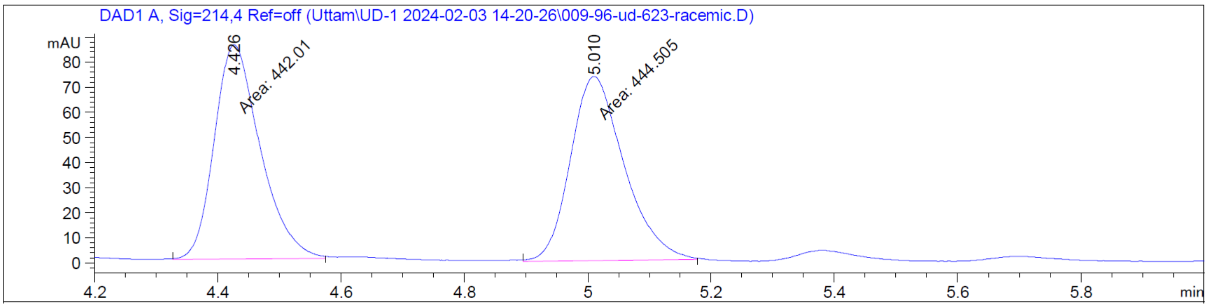

Peak #	RetTime [min]	Type	Width [min]	Area [mAU*s]	Height [mAU]	Area %
1	4.426	MM	0.0863	442.01013	85.31847	49.8593
2	5.010	MM	0.1010	444.50455	73.35660	50.1407

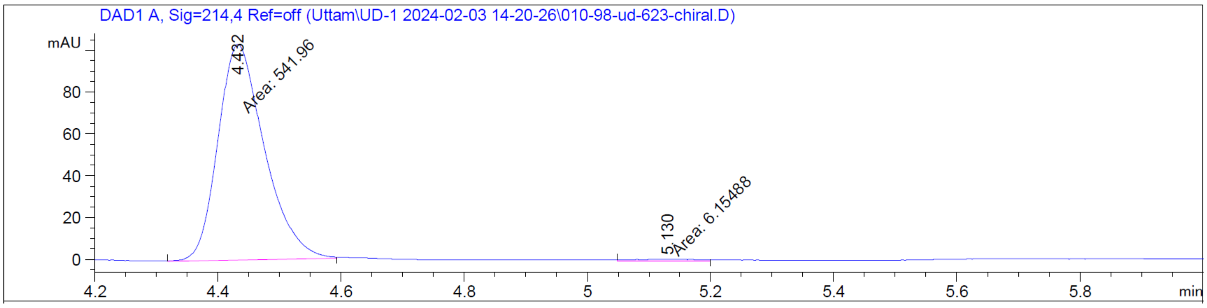

Peak #	RetTime [min]	Type	Width [min]	Area [mAU*s]	Height [mAU]	Area %
1	4.432	MM	0.0875	541.96008	103.28378	98.8771
2	5.130	MM	0.1306	6.15488	7.85504e-1	1.1229

(2*S*,3*R*)-3-fluoro-*N*-(4-methoxyphenyl)-2-methylhexadecanamide (15)

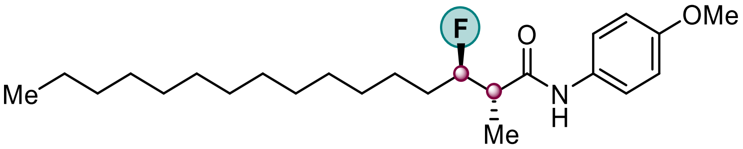

Prepared according to **GP2** with 2-iodo-*N*-(4-methoxyphenyl)propanamide (**4a**) (30.5 mg, 0.10 mmol, 1.0 equiv.), (*Z*)-1-fluorotetradec-1-ene (**S15**) (42.9 mg, 0.20 mmol, 2.0 equiv.). Automated flash column chromatography (10 g SiO_2, gradient elution: hexane to 7% EtOAc in hexane) afforded the desired product (–) **15** as a yellow oil (25 mg, 64%) in >99:1 diastereomeric ratio.

^{1}H NMR (400 MHz, $CDCl_3$) δ 7.50 – 7.37 (m, 2H), 7.35 – 7.28 (m, 1H), 6.90 – 6.79 (m, 2H), 4.77 – 4.48 (m, 1H), 3.79 (s, 3H), 2.57 (dq, *J* = 17.8, 7.0 Hz, 1H), 1.81 – 1.58 (m, 3H), 1.53 – 1.38 (m, 2H), 1.26 (d, *J* = 5.0 Hz, 22H), 0.88 (t, *J* = 6.8 Hz, 3H).

^{13}C NMR (101 MHz, $CDCl_3$) δ 171.41 (d, $^3J_{C–F}$ = 1.5 Hz), 156.47, 130.91, 121.86, 114.13, 95.68 (d, $^1J_{C–F}$ = 170.2 Hz), 55.50, 47.09 (d, $^2J_{C–F}$ = 20.5 Hz), 32.59 (d, $^2J_{C–F}$ = 20.9 Hz), 31.93, 29.69, 29.67, 29.66, 29.64, 29.56, 29.49, 29.37, 29.36, 24.85 (d, $^3J_{C–F}$ = 3.7 Hz), 22.70, 14.13, 13.95 (d, $^3J_{C–F}$ = 7.3 Hz).

^{19}F{1H} NMR (376 MHz, $CDCl_3$) δ -182.17.

HRMS (ESI/QTOF) m/z: $[M + Na]^+$ Calcd for $C_{24}H_{40}FNNaO_2^+$ 416.2935; Found 416.2950. $[\alpha]_D^{23}$ = –7.1 (c = 1.00 in $CHCl_3$).

HPLC: The enantiomeric excess (98%) was determined *via* HPLC analysis using a CHIRALCEL® OJ-H column, with hexane:isopropanol = 85:15 at a flow rate 1.0 mL/min detected at 254 nm wavelength. Retention time: t_{major} = 6.2 min and t_{minor} = 5.4 min. Diastereomeric ratio (>99:1) was determined by ^{19}F-NMR of the crude reaction mixture.

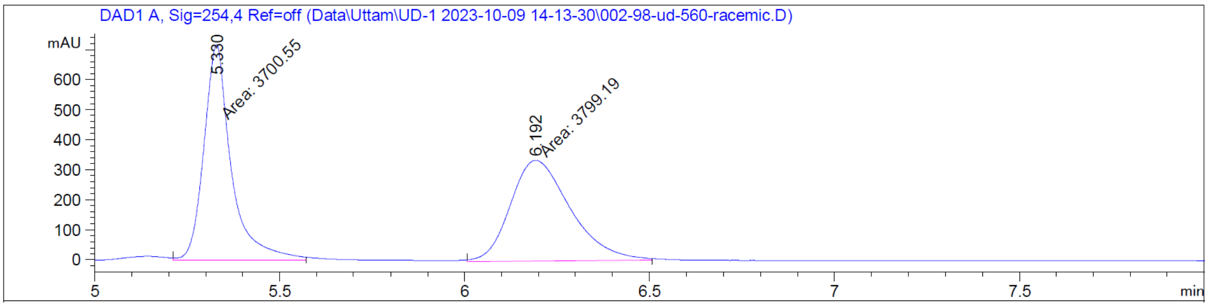

Peak #	RetTime [min]	Type	Width [min]	Area [mAU*s]	Height [mAU]	Area %
1	5.330	MM	0.0859	3700.55273	718.33484	49.3424
2	6.192	MM	0.1886	3799.19385	335.65631	50.6576

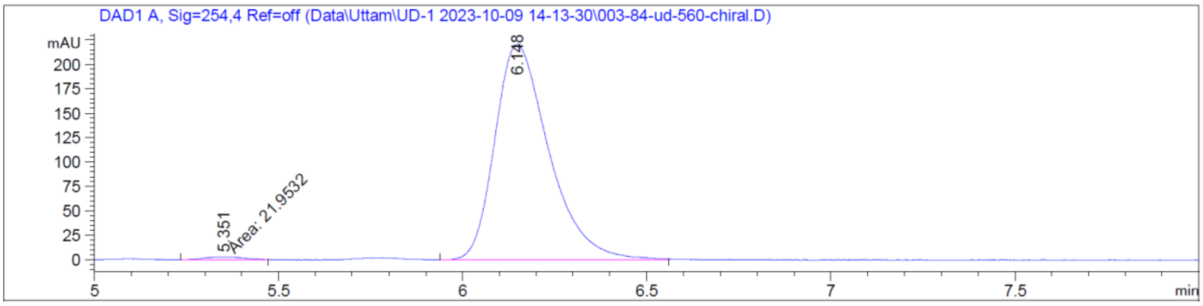

Peak #	RetTime [min]	Type	Width [min]	Area [mAU*s]	Height [mAU]	Area %
1	5.351	MM	0.1239	21.95317	2.95272	0.9683
2	6.148	BV	0.1505	2245.22607	220.05775	99.0317

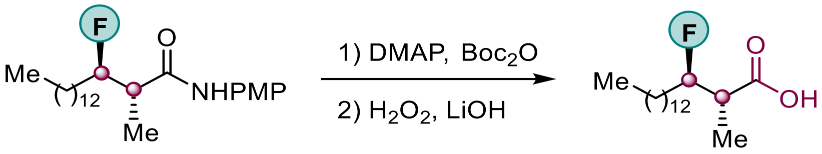

(2*S*,3*R*)-3-Fluoro-2-methylhexadecanoic acid (16)

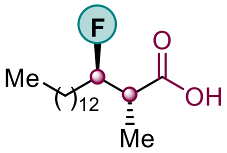

To a solution of (2*S*,3*R*)-3-fluoro-*N*-(4-methoxyphenyl)-2-methylhexadecanamide **(15)** (14 mg, 35.6 μmol, 1 equiv., 98% e.e., >99:1 d.r.) in CH_3CN (0.8 mL) was added di-*tert*-butyl dicarbonate (22.8 μL, 107 μmol, 3 eq.) at room temperature, and the reaction mixture was stirred at 50 ºC for 15 min, Then, 4-dimethylaminopyridine (435 μg, 3.56 μmol, 0.1 eq.) was added. After stirring for another 30 min at 50 ºC, the reaction mixture was cooled to room temperature. The solvent was removed under reduced pressure and the residue was dissolved in THF:H_2O (3:1, 1.0 mL). After cooling the biphasic mixture to 0 ºC, 30 wt% aq. hydrogen peroxide (30%, 36.3 μL) was added followed by lithium hydroxide monohydrate (8.96 mg, 213 μmol, 6 eq.). The mixture was allowed to warm up and was stirred at room temperature for 6 h. It was then again cooled to 0 ºC and treated with 1.5 N aqueous solution of Na_2SO_3 (1.1 equiv). After stirring the resulting mixture at room temperature for 5 min, it was then diluted with water. The mixture was washed with dichloromethane to remove organic impurities and the remaining aqueous layer was acidified to pH = 4 and extracted with EtOAc. The organic layer was concentrated under reduced pressure and the residue was purified by automated flash column chromatography (10 g SiO_2, gradient elution: hexane to 100% EtOAc in hexane) to afford the desired product (+)-**16** as a yellow oil (22.5 mg, 78%).

^{1}H NMR (400 MHz, $CDCl_3$) δ 4.89 – 4.45 (m, 1H), 2.93 – 2.63 (m, 1H), 1.74 – 1.43 (m, 6H), 1.34 – 1.26 (m, 18H), 1.23 – 1.15 (m, 3H), 0.91 (t, *J* = 6.6 Hz, 3H).
^{13}C NMR (101 MHz, $CDCl_3$) (two resonances are missing due to overlap): δ 178.86, 94.34 (d, $^1J_{C\text{–}F}$ = 172.0 Hz), 44.26 (d, $^2J_{C\text{–}F}$ = 22.4 Hz), 31.94, 31.78 (d, $^2J_{C\text{–}F}$ = 20.9 Hz), 29.70, 29.67, 29.64, 29.57, 29.50, 29.38, 24.85 (d, $^3J_{C\text{–}F}$ = 3.3 Hz), 22.71, 14.13, 12.61 (d, $^3J_{C\text{–}F}$ = 6.6 Hz).
^{19}F{1H} NMR (376 MHz, $CDCl_3$) δ -181.95.
HRMS (ESI/QTOF) m/z: $[M + H_{-1}]^-$ Calcd for $C_{17}H_{32}FO_2^-$ 287.2392; Found 287.2395.
$[\alpha]_D^{23}$ = +3.1 (c = 0.45 in $CHCl_3$).

For HPLC analysis, **16** was converted back into **15**: A solution of oxalyl chloride (2.62 µL, 30.5 µmol, 1.1 eq.) in dry DCM (0.5 M) was added slowly to a stirred solution of 16 in DCM (0.5 M) at 0 °C, then 2 drops of DMF were added. Gas evolution was observed and the reaction was allowed to stir at room temperature for 2 additional hours. The reaction mixture was concentrated under reduced pressuree and re-dissolved in THF. Then, *p*-anisidine (3.76 mg, 30.5 µmol, 1.1 eq.) and triethylamine (4.63 µL, 33.3 µmol, 1.2 eq.) were added. After reaction completion(as determined by TLC), the reaction was quenched with saturated NH_4Cl solution and extracted with EtOAc three times. Organic layers were combined and concentrated under reduced pressure. The resulting residue was purified by preparative TLC to yield compound (2*S*,3*R*)-3-fluoro-*N*-(4-methoxyphenyl)-2-methylhexadecanamide (**15**). HPLC analysis was performed of **15**.

HPLC: The enantiomeric excess (98%) was determined *via* HPLC analysis using a CHIRALCEL® OJ-H column, with hexane:isopropanol = 85:15 at a flow rate 1.0 mL/min detected at 254 nm wavelength. Retention time: t_{major} = 6.2 min and t_{minor} = 5.3 min. Diastereomeric ratio (>99:1) was determined by ^{19}F-NMR of the crude reaction mixture.

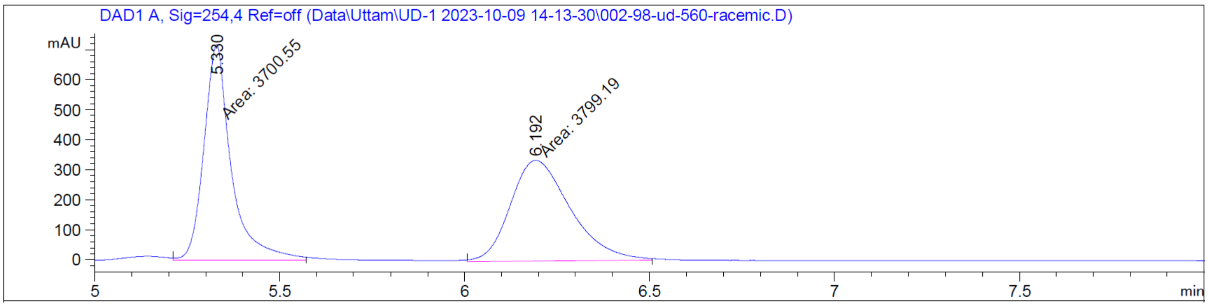

Peak #	RetTime [min]	Type	Width [min]	Area [mAU*s]	Height [mAU]	Area %
1	5.330	MM	0.0859	3700.55273	718.33484	49.3424
2	6.192	MM	0.1886	3799.19385	335.65631	50.6576

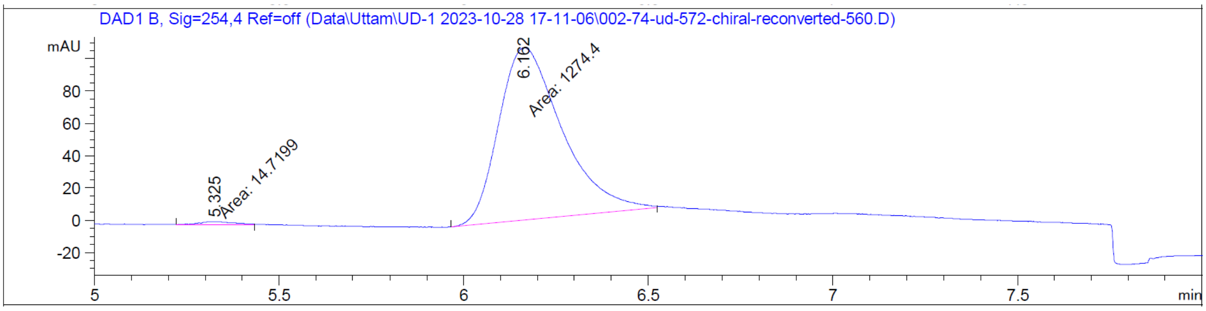

Peak #	RetTime [min]	Type	Width [min]	Area [mAU*s]	Height [mAU]	Area %
1	5.325	MM	0.1179	14.71993	2.08123	1.1419
2	6.162	MM	0.1991	1274.39807	106.67178	98.8581

7. Mechanistic experiments

a) Radical scavenger experiment

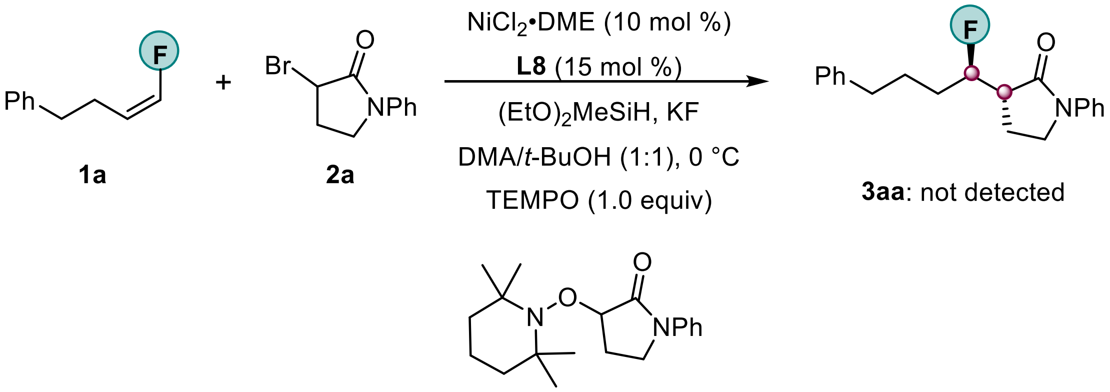

18: detected by HRMS

The reaction was conducted following **GP1**. TEMPO (15.6 mg, 0.10 mmol, 1.0 equiv.) was added after the addition of all other reagents. The hydroalkylation product **3aa** was not detected. TEMPO-adduct **18** was detected by HRMS.

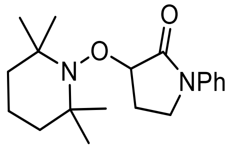

HRMS (ESI/QTOF) m/z: $[M + H]^+$ Calcd for $C_{19}H_{29}N_2O_2^+$ 317.2224; Found 317.2234.

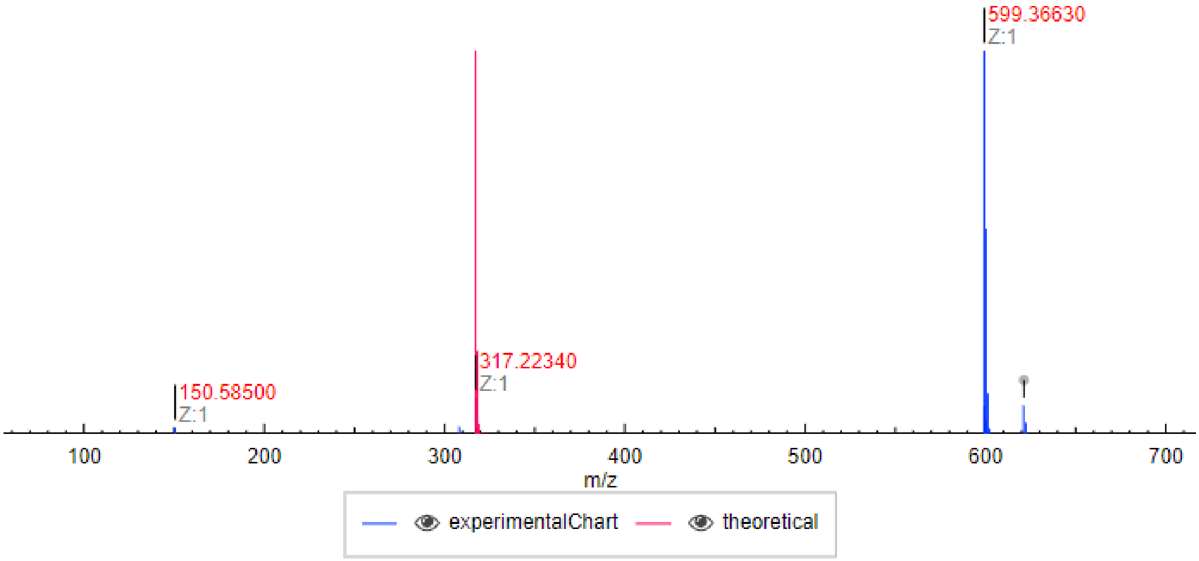

Figure S5. HRMS spectra of **18**.

b) Radical-clock ring-opening experiment

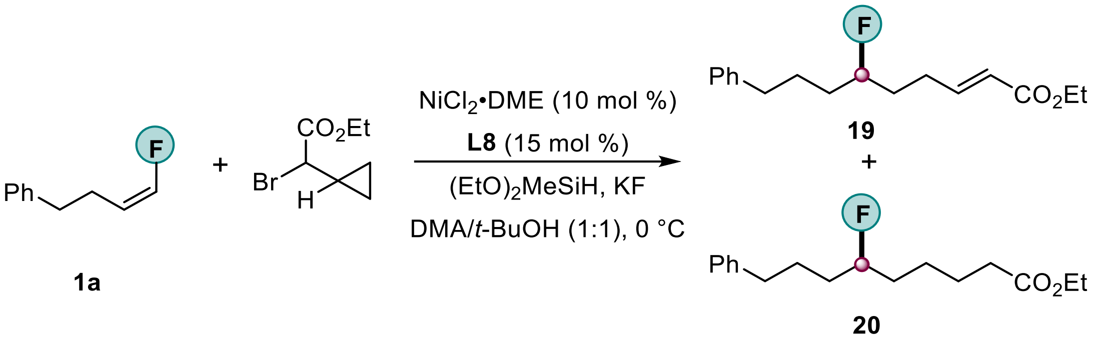

The reaction was conducted following **GP1**, with **1a** (30.0 mg, 0.10 mmol, 1.0 equiv.) and ethyl 2-bromo-2-cyclopropyl ester (41.4 mg, 200 µmol, 1 eq.) as coupling partners. Automated flash column chromatography (10 g SiO_2, gradient elution: hexane to 20% EtOAc in hexane) afforded the mixture of ring-opening products **19** and **20** as a colourless oil (11.0 mg, ~40%).

These two products cannot be purified separately. The supporting spectra (NMR and HRMS) are shown below. For **20**, HRMS (ESI/QTOF) m/z: $[M + Na]^+$ Calcd for $C_{17}H_{25}FNaO_2^+$ 303.1731; Found 303.1735. For **19**, HRMS (ESI/QTOF) m/z: $[M + Na]^+$ Calcd for $C_{17}H_{23}FNaO_2^+$ 301.1574; Found 301.1582.

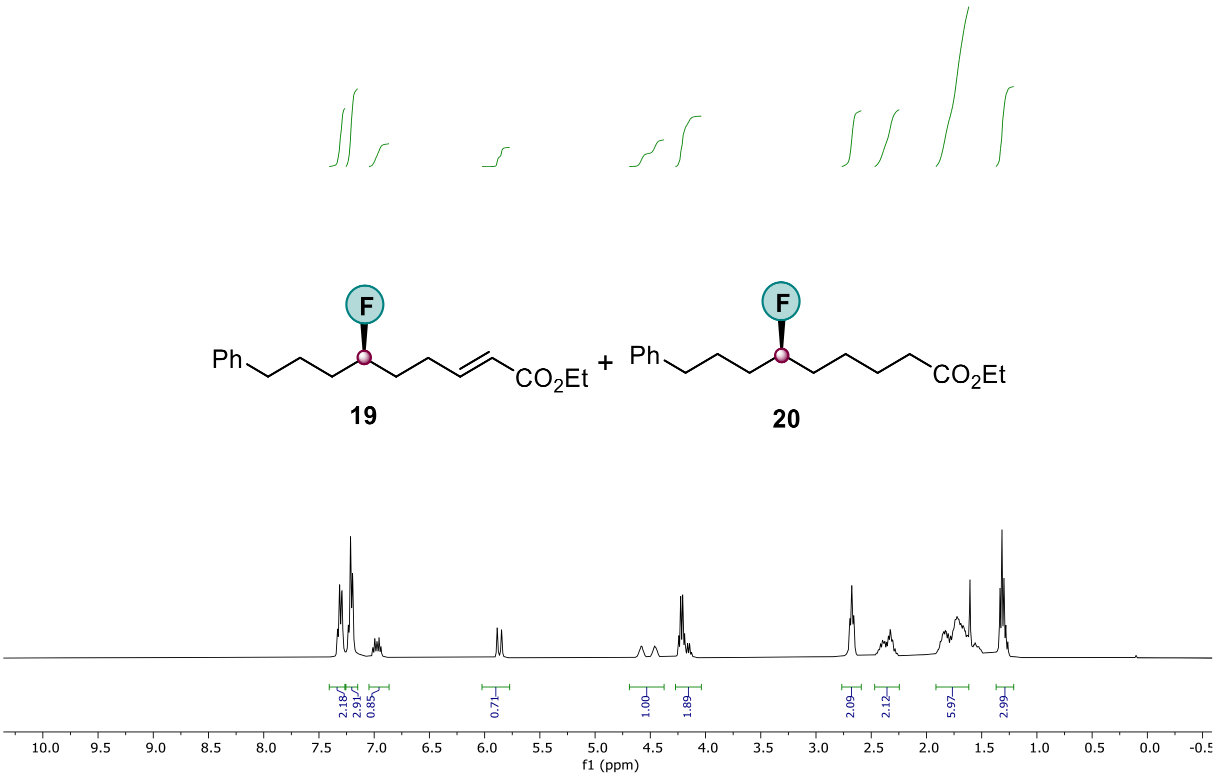

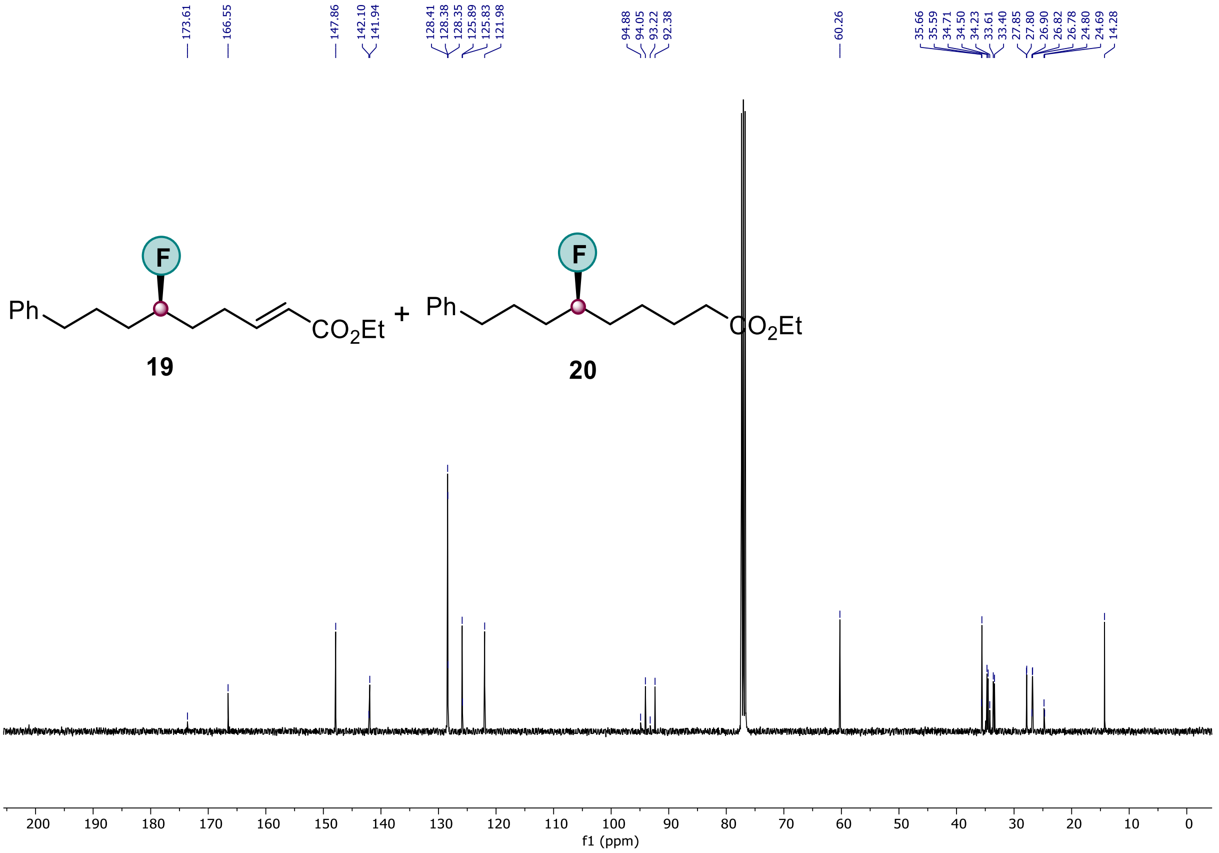

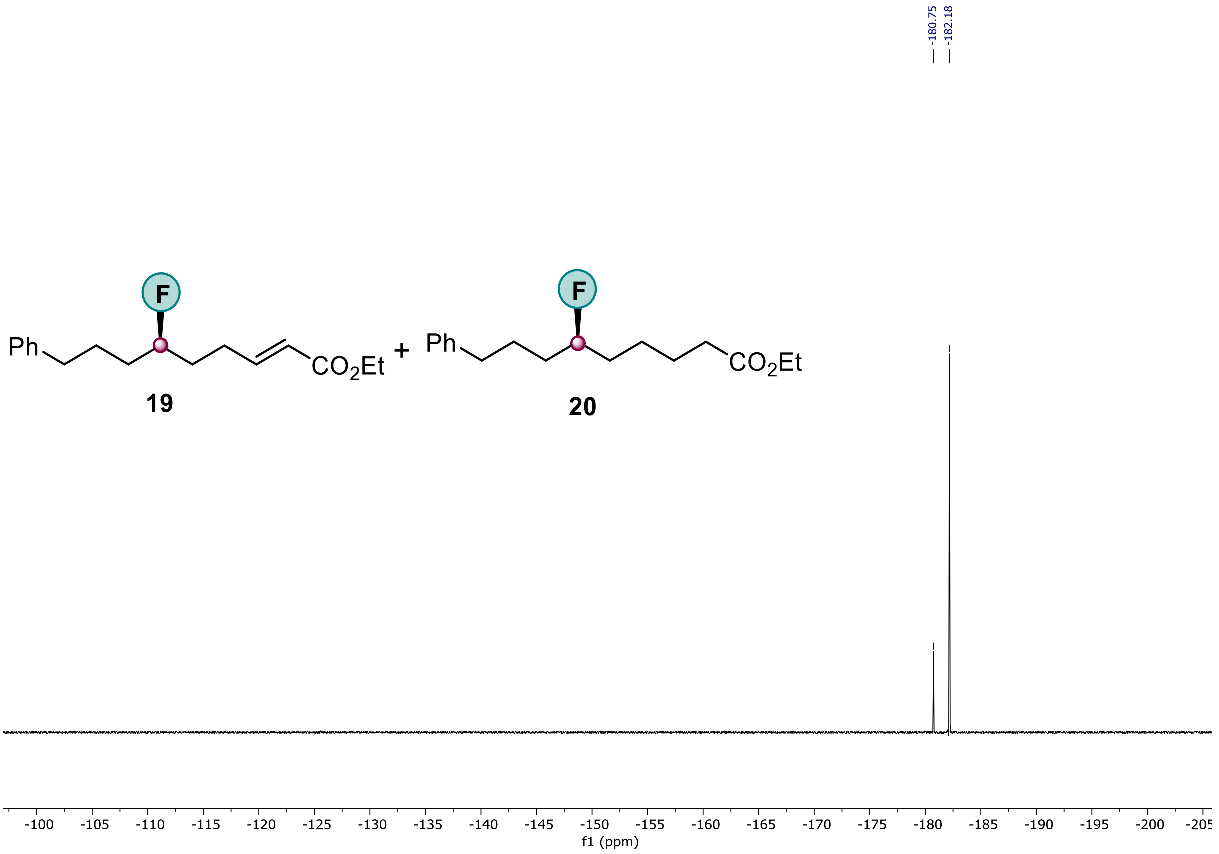

Figure S6. NMR Spectra of **19** and **20**.

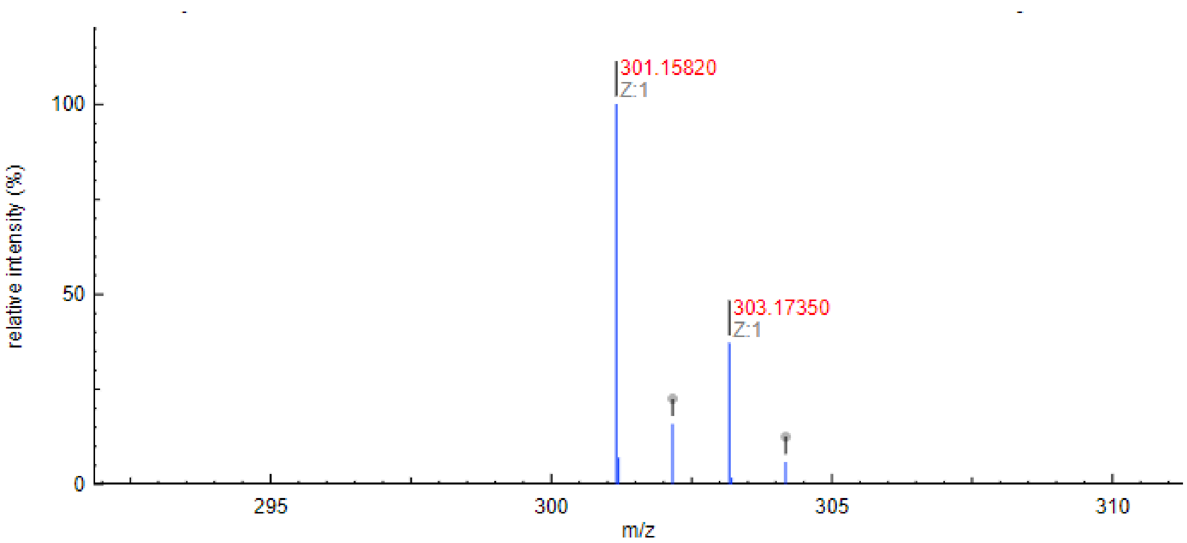

Figure S7. HRMS of **19** and **20**.

c) Reaction monitoring

Procedure (P1) for reaction-monitoring experiments

To an oven-dried 10 mL Schlenk flask equipped with a magnetic stir bar (6 x 15 mm) were added $NiCl_2$·DME (8.8 mg, 0.04 mmol, 0.10 equiv.) and ligand **L8** (36 mg, 0.06 mmol, 0.15 equiv.) under an inert nitrogen (N_2) atmosphere using glove-box techniques. Then anhydrous DMA/*t*-BuOH (2:2 mL) were added. The mixture was stirred for ~1.5 hours at room temperature. Then, anhydrous KF (58 mg, 1.0 mmol, 2.5 equiv.) and lactam **2a** (96 mg, 0.40 mmol, 1.0 equiv.), followed by fluoroalkene **1a** (~93% purity, 64 mg, 0.40 mmol, 1.0 equiv.) and trifluorotoluene as internal standard (15 µL, 0.12 mmol, 0.30 equiv.) were added to it. At this point, $(EtO)_2MeSiH$ (162 µL, 1.0 mmol, 2.5 equiv.) was added. The Schlenk flask was removed from the glove-box, fitted with a nitrogen balloon and stirred in an ice-water bath at 0 °C, maintaining 520 rpm. Aliquots of ~0.2 mL reaction mixture were taken *via* syringe at the following times after silane addition: 3 min, 6 h, 8 h, 10 h, 12 h, 14 h, 16 h, 18 h, 20 h. The withdrawn reaction mixture was quenched by addition of sat. aq. NH_4Cl solution (cca. 0.2 mL) and extracted into *d*-chloroform. The organic phase was dried over anhydrous Na_2SO_4 and syringed through a PFTE membrane filter into an NMR tube. ^{19}F NMR experiment was performed to determine the amounts of remaining fluoroalkene and product **3aa**.

Experiment A

Procedure **P1** was followed using (*Z*)-**1a**. The recorded ^{19}F-NMR spectra were processed in MestreNova software by applying auto phase and baseline corrections and using Sine Square 90.00 ° apodization to reduce ringing. The monitored species and their chemical shifts are: trifluorotoluene, -62.80 ppm; (*E*)-**1a**, -129.87 ppm; (*Z*)-**1a**, -130.24 ppm; product **3aa**, -189.70 ppm.

Experiment B

Procedure **P1** was followed using a (*Z*)-**1a / (***E*)-**1a** mixture in a 1.01: 1.00 ratio. The recorded NMR spectra were processed in the same way as for Experiment A.

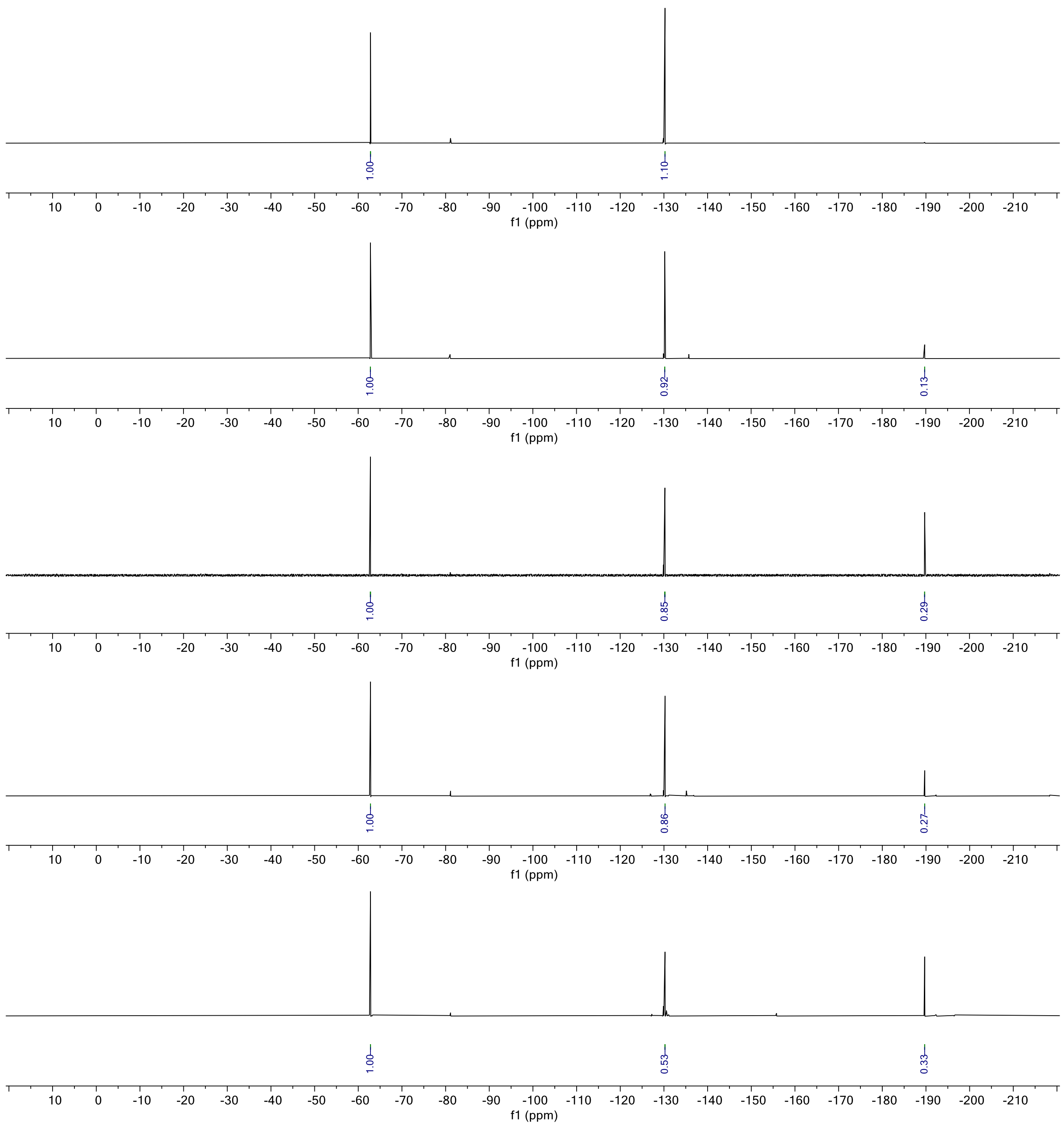

Figure S8: Selected spectra recorded as part of Experiment A and ordered in increasing time progression from top to bottom (3 min, 6 h, 8 h, 10 h, 20 h).

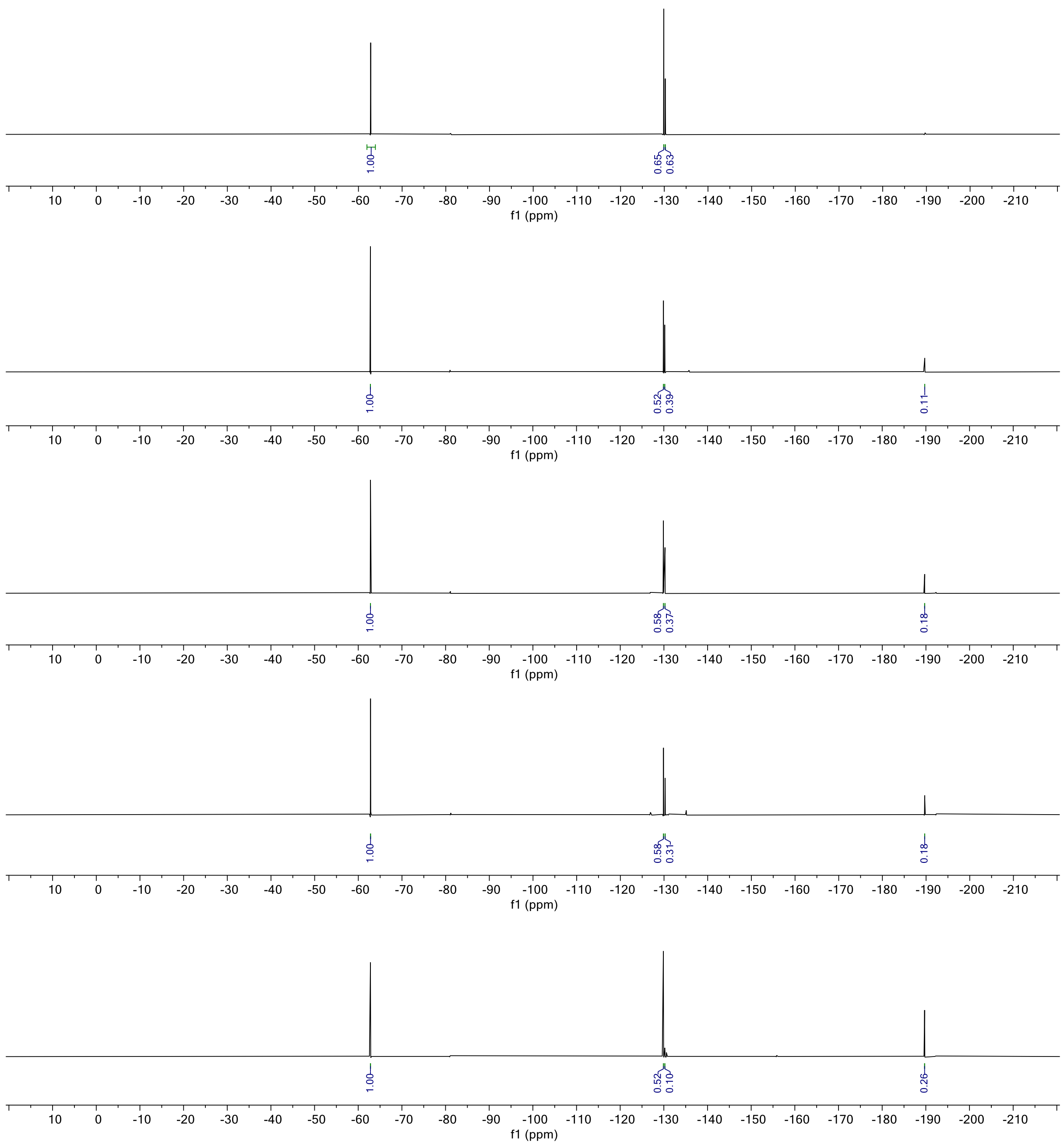

Figure S9: Selected spectra recorded as part of Experiment B and ordered in increasing time progression from top to bottom (3 min, 6 h, 8 h, 10 h, 20 h).

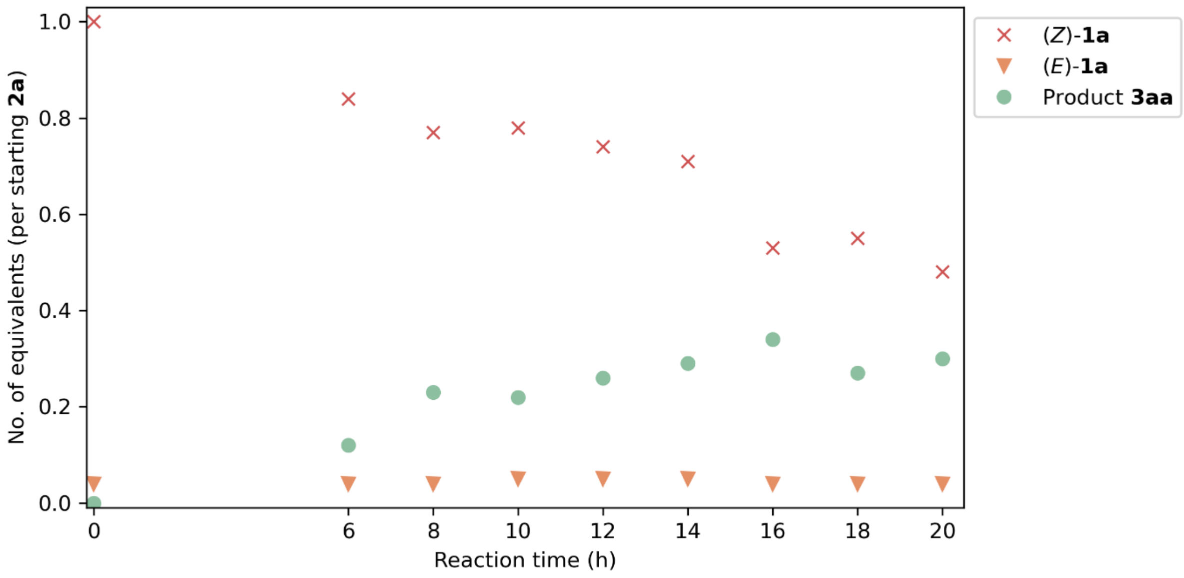

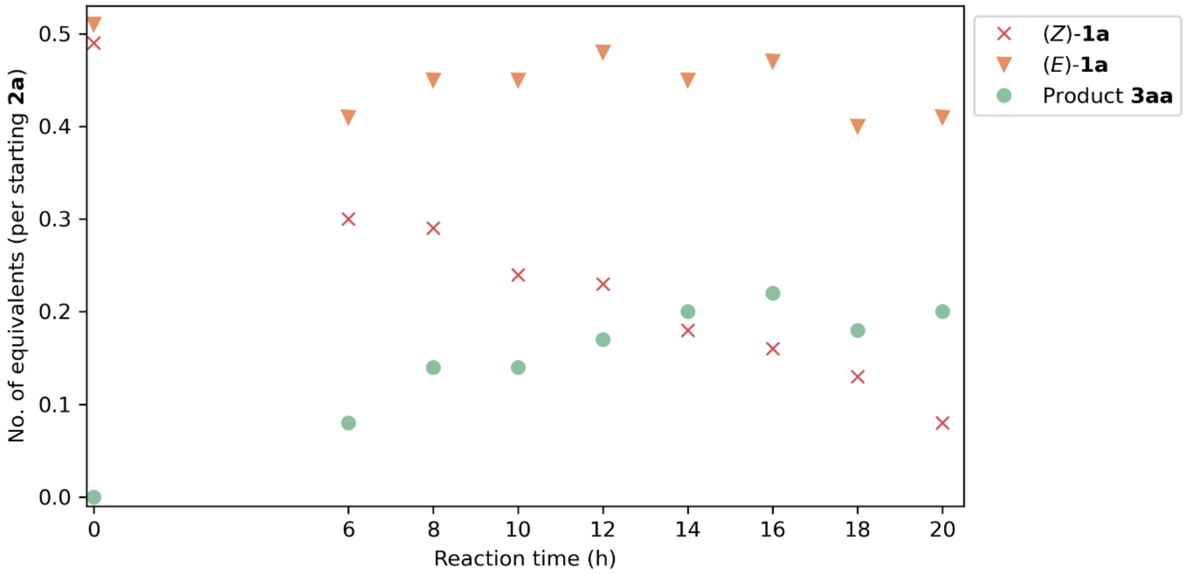

Figure S10: Data from Experiments **A** (top) and **B** (bottom) plotted as reaction time in hours *versus* the amount of monitored species in equivalents per starting lactam **2a** (0.4 mmol, 1.0 equiv.).

d) Experiment using pure (*E*)-1a

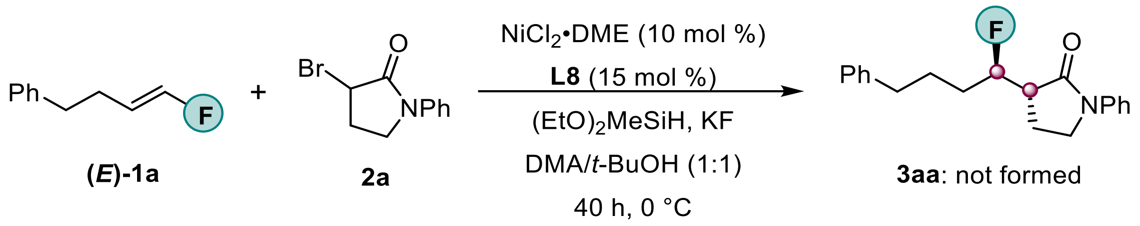

The reaction was conducted following **GP1**. Crude reaction mixture was analyzed by GC-MS and ^{19}F NMR (100 μL of 0.33 M trifluorotoluene solution in $CDCl_3$ were added as internal standard). No **3aa** formation was observed and 93% of the starting (*E*)-**1a** remained unreacted.

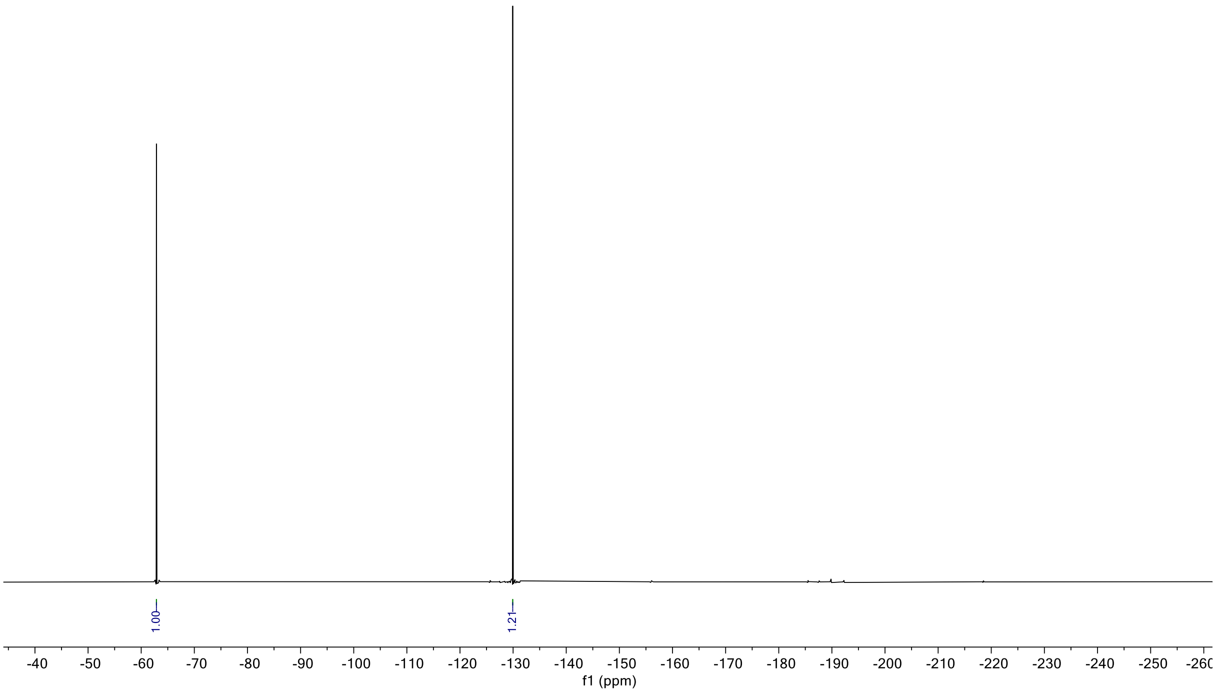

Figure S11: ^{19}F NMR analysis of the crude reaction mixture. The observed species and their chemical shifts are: trifluorotoluene, -62.80 ppm; (*E*)-**1a**, -129.87 ppm.

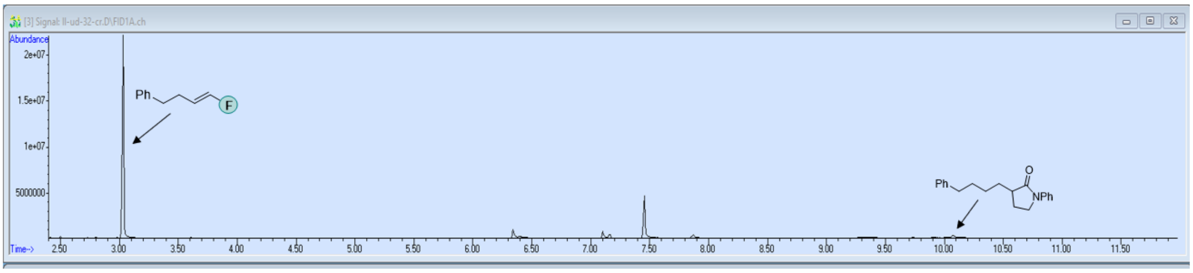

Figure S12: GC-MS analysis of the crude reaction mixture.

e) Deuterium labelling experiment

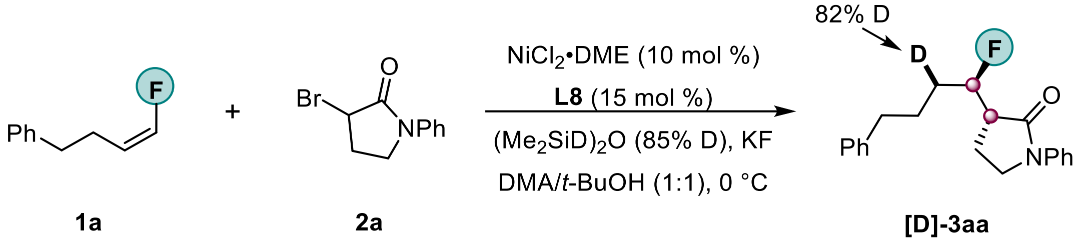

To an oven-dried 10 mL Teflon-screw capped vial was added $NiCl_2 \cdot DME$ (2.2 mg, 10 μmol, 0.1 eq.) and **L8** (8.98 mg, 15 μmol, 0.15 eq.). The vial was introduced in a nitrogen filled glovebox. A magnetic stir bar (6x15 mm) and anhydrous DMA/*t*-BuOH (0.5/0.5 mL) were added and the mixture was stirred for 1.5 hour at room temperature until it became a clear pink solution. Then, the racemic electrophile **2a** (24.0 mg, 0.1 mmol, 1.0 equiv.) and anhydrous KF (14.5 mg, 0.25 mmol, 2.5 equiv.), followed by (*Z*)-**1a** (30 mg, 0.20 mmol, 2.0 equiv.) were added to it. At this point, ddeuterated tetramethyldisiloxane (23.2 μL, 150 μmol, 1.5 equiv., 85% D) was added. The vial was then sealed with airtight electrical tapes, removed from the glove box immediately, and stirred for 40 h at 0 °C, maintaining 520 rpm. After that, the reaction was diluted with EtOAc and concentrated under reduced pressure. Automated flash column chromatography (10 g SiO_2, gradient elution: hexane to 20% EtOAc in hexane) afforded the deuterated product [**D]-3aa** as a yellow oil (16.0 mg, 51%).

^{1}H NMR (400 MHz, $CDCl_3$) δ 7.69 – 7.56 (m, 2H), 7.40 (t, *J* = 7.8 Hz, 2H), 7.34 – 7.26 (m, 2H), 7.25 – 7.14 (m, 4H), 4.91 (d, *J* = 47.8 Hz, 1H), 3.97 – 3.73 (m, 2H), 3.17 – 2.98 (m, 1H), 2.78 – 2.60 (m, 2H), 2.34 (dtd, *J* = 13.5, 8.8, 5.1 Hz, 1H), 2.19 (dq, *J* = 14.3, 7.5 Hz, 1H), 2.12 – 2.01 (m, 0.18H), 1.99 – 1.84 (m, 1H), 1.83 – 1.63 (m, 2H).

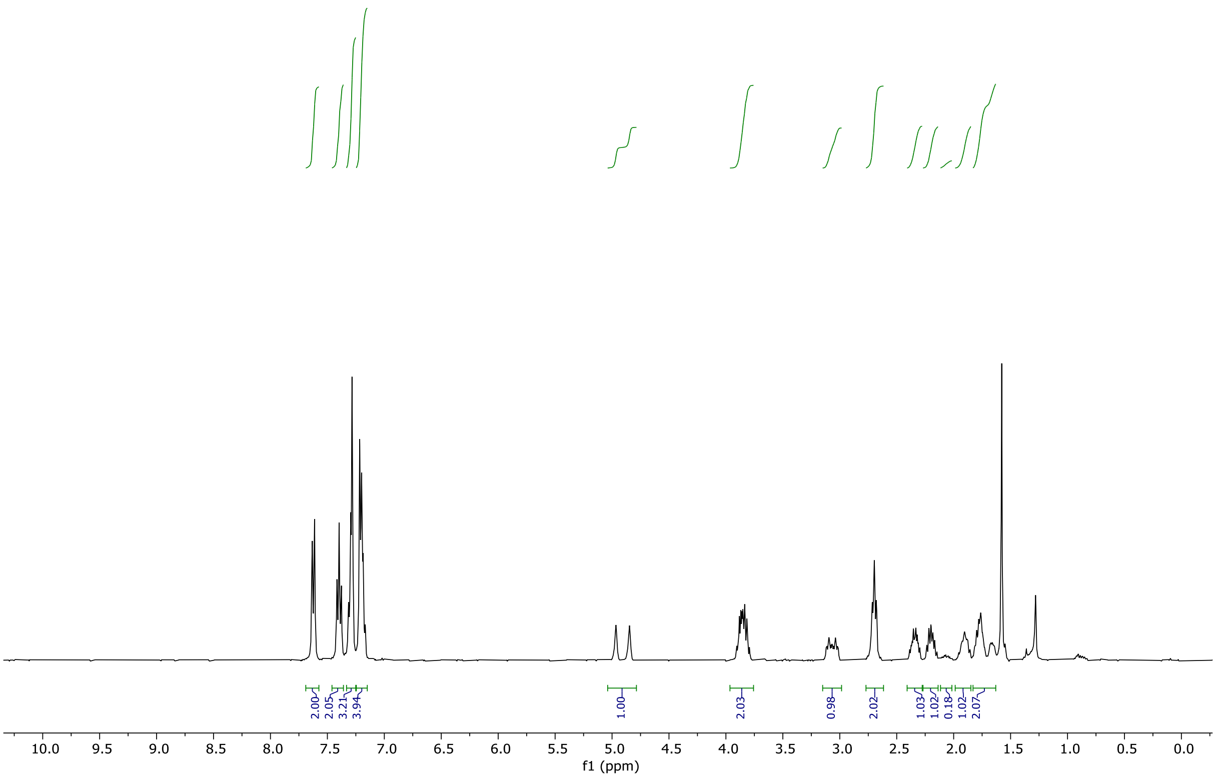

Figure S13. ^{1}H-NMR spectra of **[D]-3aa**.

f) Non-linear effect study

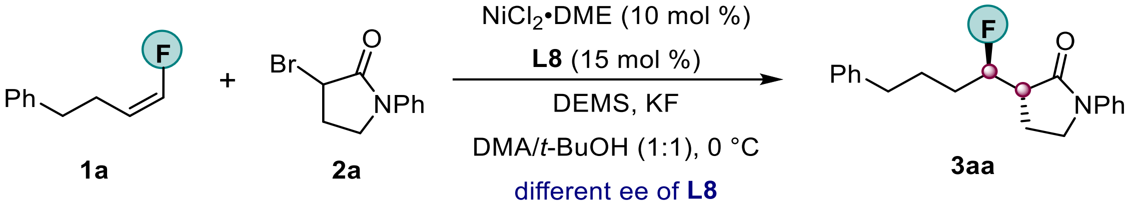

Six parallel independent reactions were conducted following **GP1**, with **1a** and **2a** as coupling partners. The crude mixture was purified by automated flash column chromatography (10 g SiO_2, gradient elution: hexane to 20% EtOAc in hexane). The enantiomeric excess was determined by HPLC with chiral stationary phase.

Table S4. Non-linear effect study.

entry	e.e. of the chiral ligand **L8**	e.e. of the product **3aa**
1	0	0
2	10	16.5
3	30	37
4	50	56
5	70	74
6	90	90.4
7	100	98.5

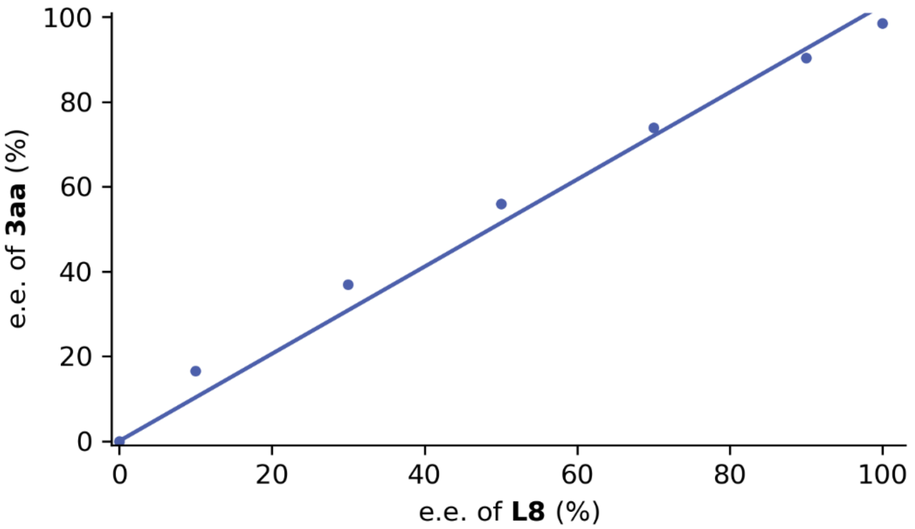

Figure S14. Absence of a non-linear effect.

g) Plausible Catalytic Cycles

Path a

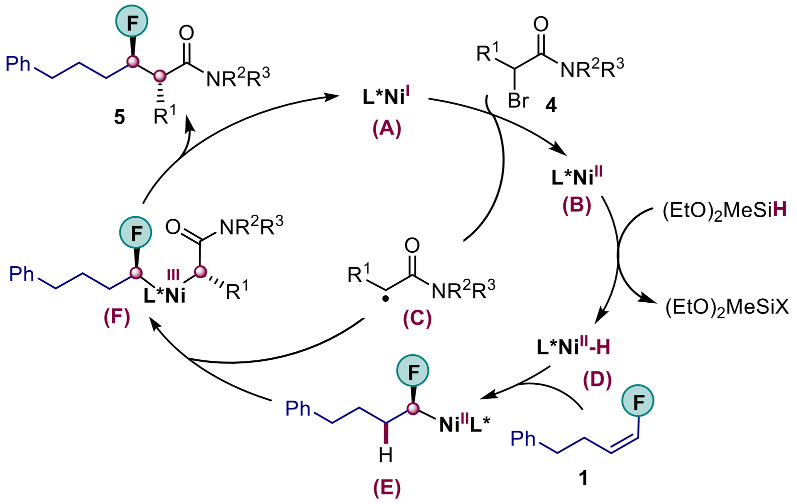

Path b

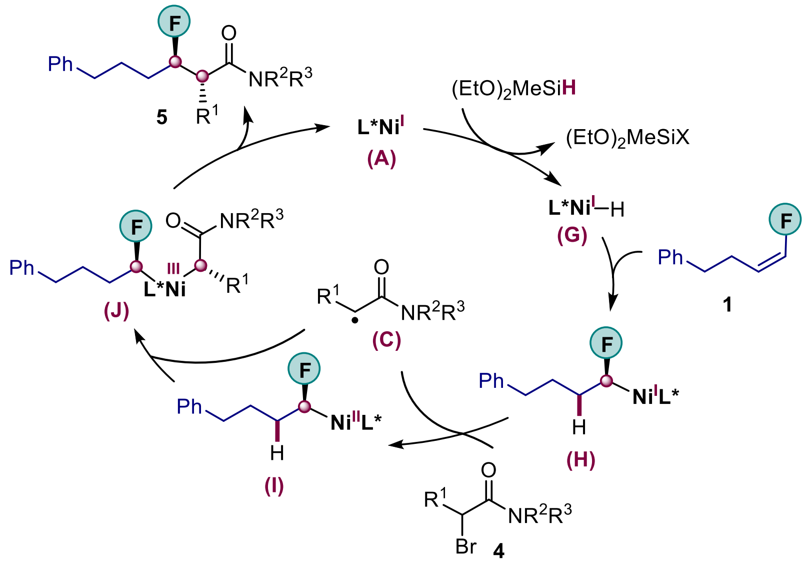

Figure S15. Two potential reaction pathways for the enantio- and diastereoselective coupling.

8. Crystallography Details

Compound (+) 3ac:

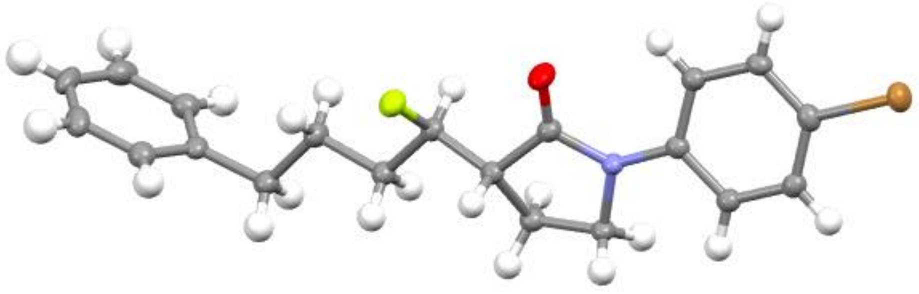

Experimental. Single colourless plate-shaped crystals of **3ac** were used as supplied. A suitable crystal with dimensions 0.55 × 0.15 × 0.03 mm^3 was selected and mounted on an XtaLAB Synergy R, DW system, HyPix-Arc 150 diffractometer. The crystal was kept at a steady T = 139.99(10) K during data collection. The structure was solved with the ShelXT 2018/2 (Sheldrick, 2015) solution program using dual methods and by using Olex2 1.5 (Dolomanov et al., 2009) as the graphical interface. The model was refined with ShelXL 2019/3 (Sheldrick, 2015) using full matrix least squares minimisation on $\boldsymbol{F^2}$.

Crystal Data. $C_{20}H_{21}NOFBr$, M_r = 390.29, orthorhombic, $P2_12_12_1$ (No. 19), a = 7.64781(10) Å, b = 7.84769(9) Å, c = 29.5254(4) Å, $a = b = g = 90^{\circ}$, V = 1772.05(4) $Å^3$, T = 139.99(10) K, Z = 4, Z' = 1, m(Cu K_a) = 3.293, 16302 reflections measured, 3621 unique (R_{int} = 0.0272) which were used in all calculations. The final wR_2 was 0.0664 (all data) and R_1 was 0.0248 (I≥2s(I)).

Compound	3ac
Formula	$C_{20}H_{21}NOFBr$
$D_{calc.}$/ g cm^{-3}	1.463
m/mm^{-1}	3.293
Formula Weight	390.29
Colour	colourless
Shape	plate-shaped
Size/mm^3	0.55×0.15×0.03
T/K	139.99(10)
Crystal System	orthorhombic
Flack Parameter	-0.009(9)
Space Group	$P2_12_12_1$
a/Å	7.64781(10)
b/Å	7.84769(9)
c/Å	29.5254(4)
a/°	90
b/°	90
g/°	90
V/Å^3	1772.05(4)
Z	4
Z'	1
Wavelength/Å	1.54184
Radiation type	Cu$K\alpha$
Q_{min}/°	2.993
Q_{max}/°	75.233
Measured Refl's.	16302
Indep't Refl's	3621
Refl's I≥2*s*(I)	3529
R_{int}	0.0272
Parameters	218
Restraints	0
Largest Peak/e Å^{-3}	0.323

Deepest Hole/e Å^{-3}	-0.431
GooF	1.042
wR_2 (all data)	0.0664
wR_2	0.0659
R_1 (all data)	0.0256
R_1	0.0248
CCDC number	2340473

Structure Quality Indicators

**Reflections:**	d min (CuKα) 2Θ=150.5° 0.80	I/σ(I) 44.6	Rint m=4.52 2.72%	Full 135.4° 99% to 150.5° 100	
**Refinement:**	Shift 0.001	Max Peak 0.3	Min Peak -0.4	GooF 1.042	Hooft -.009(9)

A colourless plate-shaped crystal with dimensions 0.55 × 0.15 × 0.03 mm^3 was mounted. Data were collected using an XtaLAB Synergy R, DW system, HyPix-Arc 150 diffractometer operating at T = 139.99(10) K.

Data were measured using ω scans with CuK$_\alpha$ radiation. The diffraction pattern was indexed and the total number of runs and images was based on the strategy calculation from the program CrysAlisPro 1.171.42.100a (Rigaku OD, 2023). The maximum resolution achieved was Θ = 75.233° (0.80 Å).

The unit cell was refined using CrysAlisPro 1.171.42.100a (Rigaku OD, 2023) on 11867 reflections, 73% of the observed reflections.

Data reduction, scaling and absorption corrections were performed using CrysAlisPro 1.171.42.100a (Rigaku OD, 2023). The final completeness is 100.00 % out to 75.233° in Θ. A Gaussian absorption correction was performed using CrysAlisPro 1.171.42.100a (Rigaku Oxford Diffraction, 2023) Numerical absorption correction based on Gaussian integration over a multifaceted crystal model. Empirical absorption correction using spherical harmonics as implemented in SCALE3 ABSPACK scaling algorithm. The absorption coefficient μ of this material is 3.293 mm^{-1} at this wavelength (λ = 1.54184Å) and the minimum and maximum transmissions are 0.407 and 1.000.

The structure was solved in the space group $P2_12_12_1$ (# 19) by the ShelXT 2018/2 (Sheldrick, 2015) structure solution program using dual methods and refined by full matrix least squares minimisation on $\boldsymbol{F^2}$ using version 2019/3 of ShelXL 2019/3 (Sheldrick, 2015). All non-hydrogen atoms were refined anisotropically. Hydrogen atom positions were calculated geometrically and

refined using the riding model.

There is a single formula unit in the asymmetric unit, which is represented by the reported sum formula. In other words: Z is 4 and Z' is 1. The moiety formula is C20 H21 Br F N O.

The Flack parameter was refined to -0.009(9). Determination of absolute structure using Bayesian statistics on Bijvoet differences using the Olex2 results in None. The chiral atoms in this structure are: C8(S), C11(R). Note: The Flack parameter is used to determine chirality of the crystal studied, the value should be near 0, a value of 1 means that the stereochemistry is wrong and the model should be inverted. A value of 0.5 means that the crystal consists of a racemic mixture of the two enantiomers.

CCDC- 2340473 contains the supplementary crystallographic data for **3ac**. These data can be obtained free of charge from The Cambridge Crystallographic Data Centre via www.ccdc.cam.ac.uk/data_request/cif.

Compound (-) 5aa:

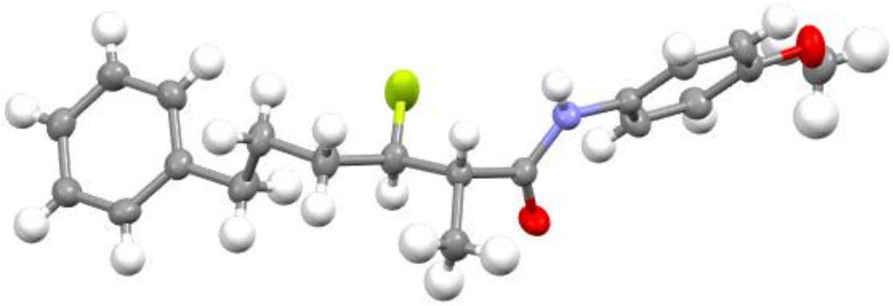

Experimental. Single colourless needle-shaped crystals of **5aa** were used as supplied. A suitable crystal with dimensions 0.56 × 0.08 × 0.04 mm^3 was selected and mounted on an XtaLAB Synergy R, DW system, HyPix-Arc 150 diffractometer. The crystal was kept at a steady T = 229.99(10) K during data collection. The structure was solved with the ShelXT 2018/2 (Sheldrick, 2015) solution program using dual methods and by using Olex2 1.5 (Dolomanov et al., 2009) as the graphical interface. The model was refined with ShelXL 2019/3 (Sheldrick, 2015) using full matrix least squares minimisation on $\boldsymbol{F}^2$.

Crystal Data. $C_{20}H_{24}FNO_2$, M_r = 329.40, orthorhombic, $P2_12_12_1$ (No. 19), a = 5.29898(5) Å, b = 16.84623(18) Å, c = 19.32257(18) Å, a = b = g = 90°, V = 1724.88(3) Å^3, T = 229.99(10) K, Z = 4, Z' = 1, m(Cu K_a) = 0.717, 20742 reflections measured, 3541 unique (R_{int} = 0.0168) which were used in all calculations. The final wR_2 was 0.0740 (all data) and R_1 was 0.0277 (I≥2s(I)).

Compound	**5aa**
Formula	$C_{20}H_{24}FNO_2$
$D_{calc.}$/ g cm^{-3}	1.268
m/mm^{-1}	0.717
Formula Weight	329.40
Colour	colourless
Shape	needle-shaped
Size/mm^3	0.56×0.08×0.04
T/K	229.99(10)
Crystal System	orthorhombic
Flack Parameter	0.07(4)
Space Group	$P2_12_12_1$
a/Å	5.29898(5)
b/Å	16.84623(18)
c/Å	19.32257(18)
a/°	90
b/°	90
g/°	90
V/Å^3	1724.88(3)
Z	4
Z'	1
Wavelength/Å	1.54184
Radiation type	Cu$K\alpha$
Q_{min}/°	3.481
Q_{max}/°	75.655
Measured Refl's.	20742
Indep't Refl's	3541
Refl's I≥2*s*(I)	3355
R_{int}	0.0168
Parameters	224
Restraints	0
Largest Peak/e Å^{-3}	0.150

Deepest Hole/e Å^{-3}	-0.111
GooF	1.072
wR_2 (all data)	0.0740
wR_2	0.0732
R_1 (all data)	0.0293
R_1	0.0277
CCDC number	2310546

Structure Quality Indicators

**Reflections:**	d min (CuKα) 2Θ=151.3° 0.80	I/σ(I) 80.0	Rint m=5.89 1.68%	Full 135.4° 99% to 151.3° 100	
**Refinement:**	Shift 0.000	Max Peak 0.1	Min Peak -0.1	GooF 1.072	Hooft [illegible]07(4)

A colourless needle-shaped crystal with dimensions 0.56 × 0.08 × 0.04 mm³ was mounted. Data were collected using an XtaLAB Synergy R, DW system, HyPix-Arc 150 diffractometer operating at T = 229.99(10) K.

Data were measured using w scans with CuK$_a$ radiation. The diffraction pattern was indexed and the total number of runs and images was based on the strategy calculation from the program CrysAlisPro 1.171.42.90a (Rigaku OD, 2023). The maximum resolution achieved was Q = 75.655° (0.80 Å).

The unit cell was refined using CrysAlisPro 1.171.42.90a (Rigaku OD, 2023) on 13922 reflections, 67% of the observed reflections.

Data reduction, scaling and absorption corrections were performed using CrysAlisPro 1.171.42.90a (Rigaku OD, 2023). The final completeness is 100.00 % out to 75.655° in Q. A Gaussian absorption correction was performed using CrysAlisPro 1.171.42.90a (Rigaku Oxford Diffraction, 2023) Numerical absorption correction based on Gaussian integration over a multifaceted crystal model. Empirical absorption correction using spherical harmonics as implemented in SCALE3 ABSPACK scaling algorithm. The absorption coefficient m of this material is 0.717 mm^{-1} at this wavelength (l = 1.54184Å) and the minimum and maximum transmissions are 0.859 and 1.000.

The structure was solved in the space group $P2_12_12_1$ (# 19) by the ShelXT 2018/2 (Sheldrick, 2015) structure solution program using dual methods and refined by full matrix least squares minimisation on $\boldsymbol{F}^2$ using version 2019/3 of ShelXL 2019/3 (Sheldrick, 2015). All non-hydrogen

atoms were refined anisotropically. Hydrogen atom positions were calculated geometrically and refined using the riding model, but the hydrogen atom bound to N1 was found in a difference map and refined freely.

There is a single formula unit in the asymmetric unit, which is represented by the reported sum formula. In other words: Z is 4 and Z' is 1. The moiety formula is C20 H24 F N O2.

The Flack parameter was refined to 0.07(4). Determination of absolute structure using Bayesian statistics on Bijvoet differences using the Olex2 results in None. The chiral atoms in this structure are: C2(S), C3(R). Note: The Flack parameter is used to determine chirality of the crystal studied, the value should be near 0, a value of 1 means that the stereochemistry is wrong and the model should be inverted. A value of 0.5 means that the crystal consists of a racemic mixture of the two enantiomers.

CCDC- 2310546 contains the supplementary crystallographic data for **5aa**. These data can be obtained free of charge from The Cambridge Crystallographic Data Centre via www.ccdc.cam.ac.uk/data_request/cif

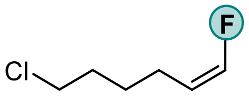
F
Cl

1b

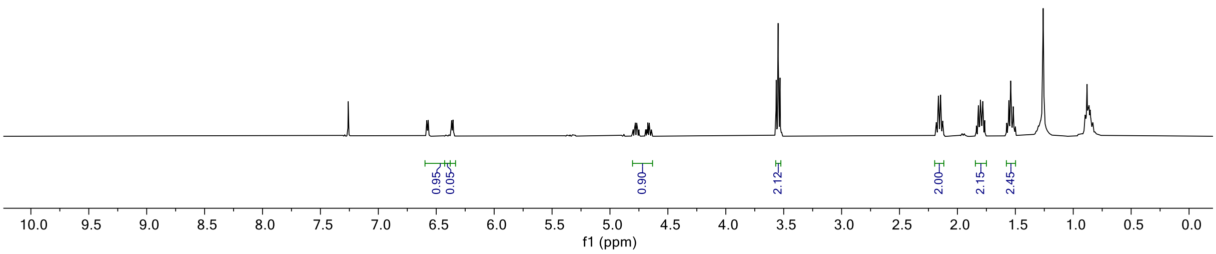
0.95
0.05
0.90
2.12
2.00
2.15
2.45
10.0 9.5 9.0 8.5 8.0 7.5 7.0 6.5 6.0 5.5 5.0 4.5 4.0 3.5 3.0 2.5 2.0 1.5 1.0 0.5 0.0
f1 (ppm)

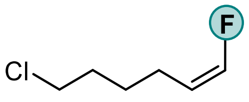
F
Cl

1b

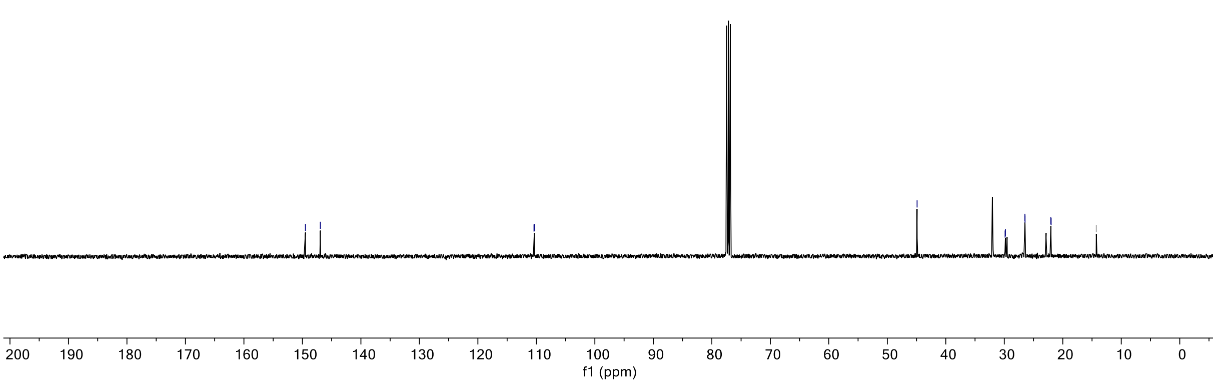
149.49
146.94
110.40
110.35
44.91
29.86
29.81
26.47
26.45
22.04
21.99
14.27 Hexane
200 190 180 170 160 150 140 130 120 110 100 90 80 70 60 50 40 30 20 10 0
f1 (ppm)

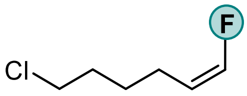

1b

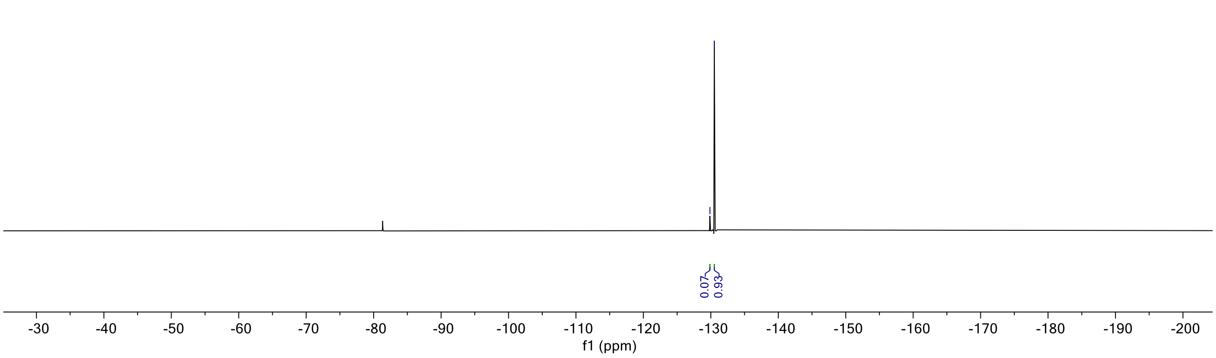

-129.87
-130.51
0.07
0.93
-30
-40
-50
-60
-70
-80
-90
-100
-110
-120
-130
-140
-150
-160
-170
-180
-190
-200
f1 (ppm)

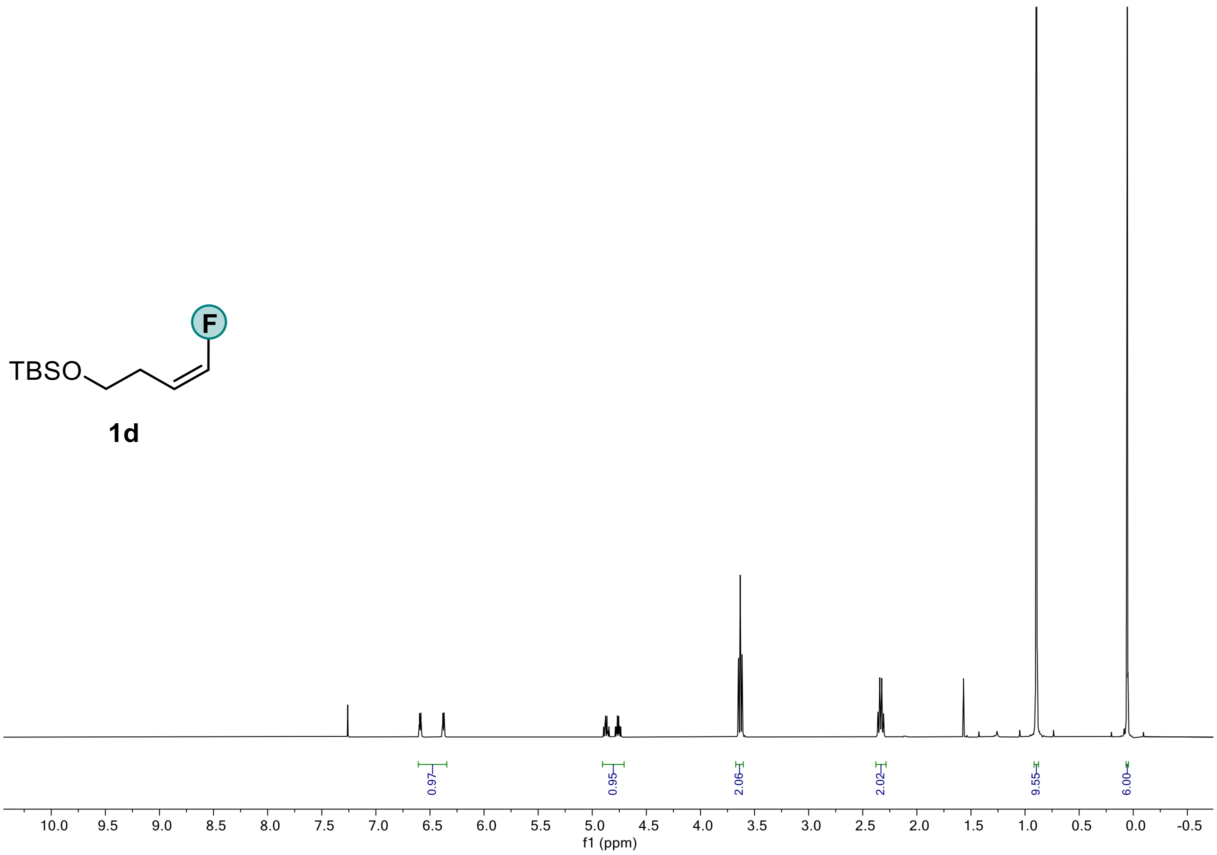
TBSO
F
1d
0.97
0.95
2.06
2.02
9.55
6.00
10.0 9.5 9.0 8.5 8.0 7.5 7.0 6.5 6.0 5.5 5.0 4.5 4.0 3.5 3.0 2.5 2.0 1.5 1.0 0.5 0.0 -0.5
f1 (ppm)

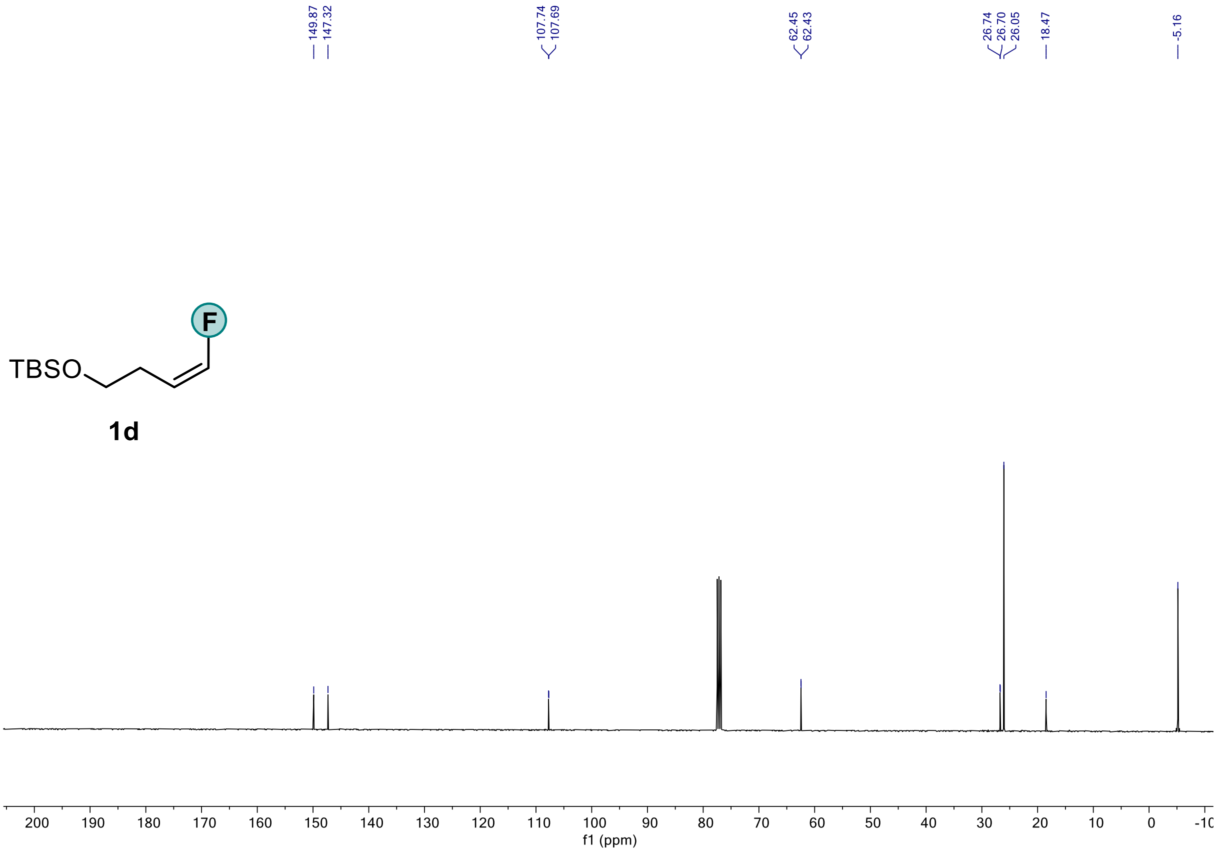
149.87
147.32
107.74
107.69
62.45
62.43
26.74
26.70
26.05
18.47
-5.16
TBSO
F
1d
200 190 180 170 160 150 140 130 120 110 100 90 80 70 60 50 40 30 20 10 0
f1 (ppm)

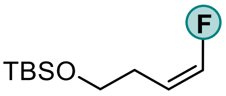

TBSO
F

1d

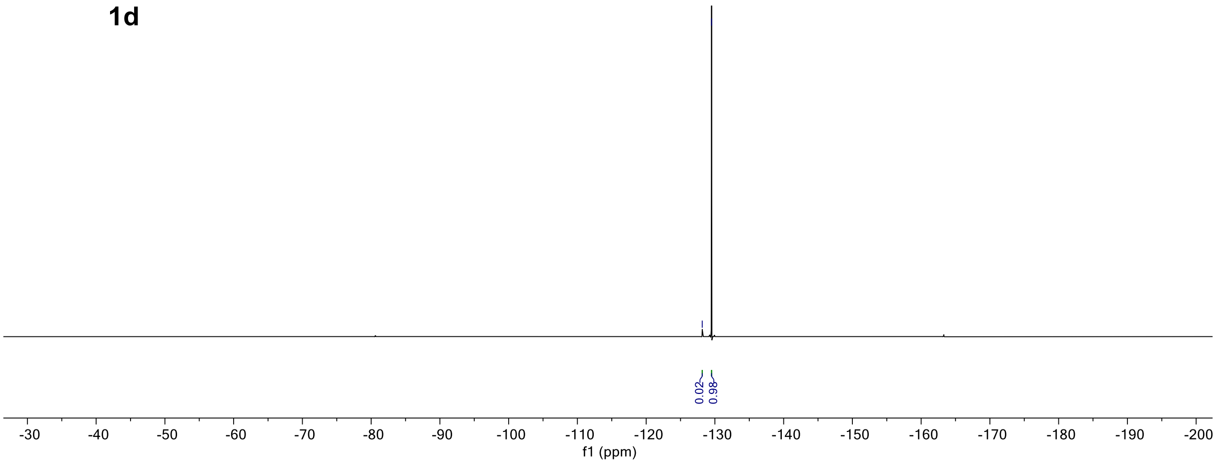

-128.15
-129.51
0.02
0.98
-30
-40
-50
-60
-70
-80
-90
-100
-110
-120
-130
-140
-150
-160
-170
-180
-190
-200
f1 (ppm)

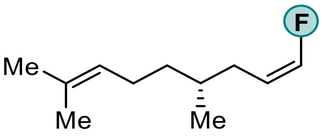
Me
Me
Me
F

1g

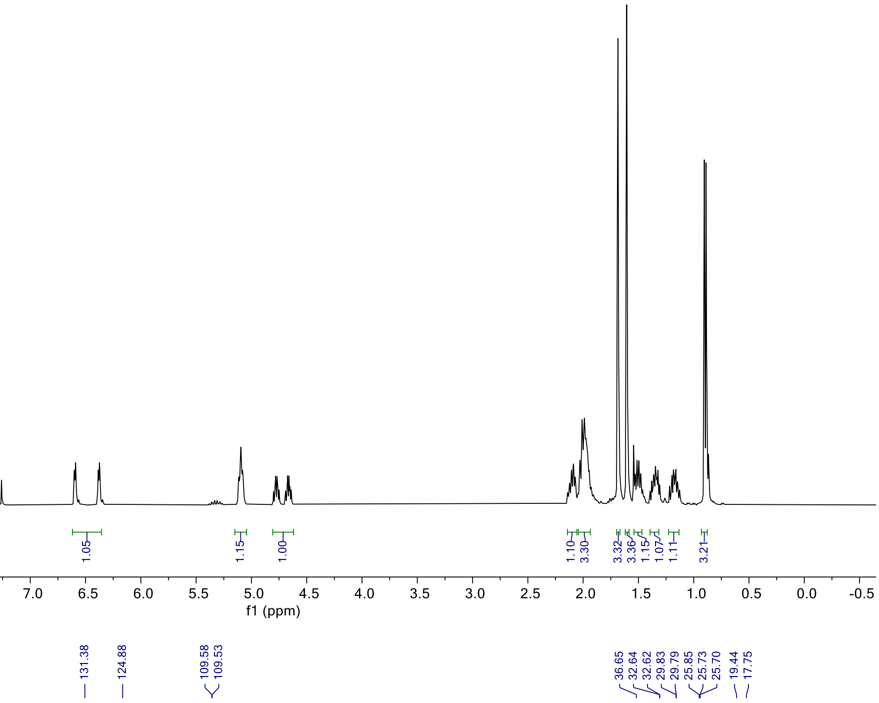
1.05
1.15
1.00
1.10
3.30
3.32
3.36
1.15
1.07
1.11
3.21
10.0 9.5 9.0 8.5 8.0 7.5 7.0 6.5 6.0 5.5 5.0 4.5 4.0 3.5 3.0 2.5 2.0 1.5 1.0 0.5 0.0 -0.5
f1 (ppm)

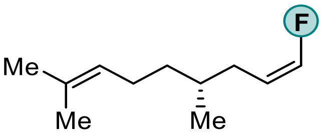
Me
Me
Me
F

1g

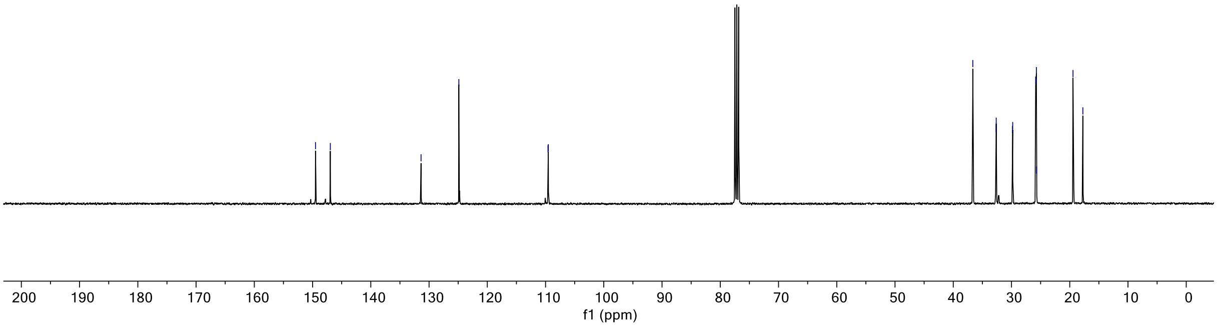
149.49
146.95
131.38
124.88
109.58
109.53
36.65
32.64
32.62
29.83
29.79
25.85
25.73
25.70
19.44
17.75
200 190 180 170 160 150 140 130 120 110 100 90 80 70 60 50 40 30 20 10 0
f1 (ppm)

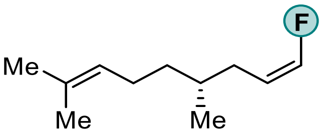
Me
Me
Me
F

1g

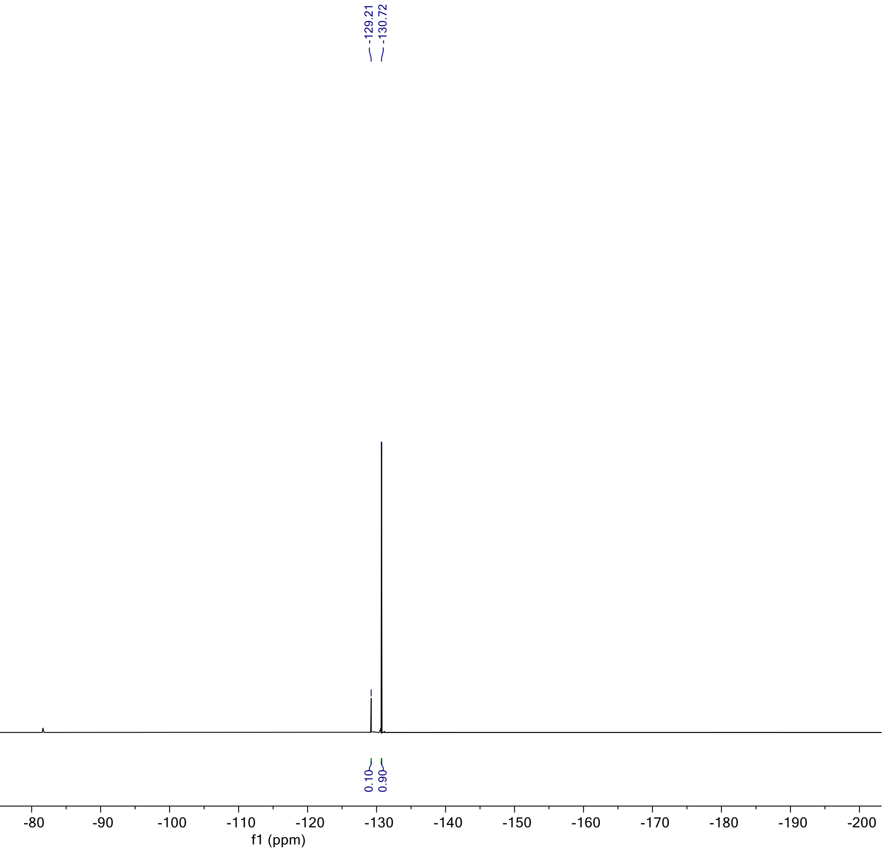
-129.21
-130.72
0.10
0.90
-30
-40
-50
-60
-70
-80
-90
-100
-110
-120
-130
-140
-150
-160
-170
-180
-190
-200
f1 (ppm)

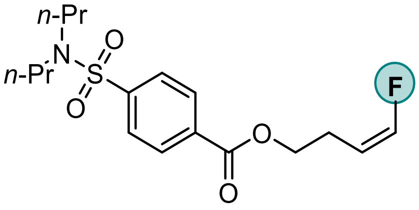
n-Pr
N
n-Pr
S
O
O
O
O
F

S-8

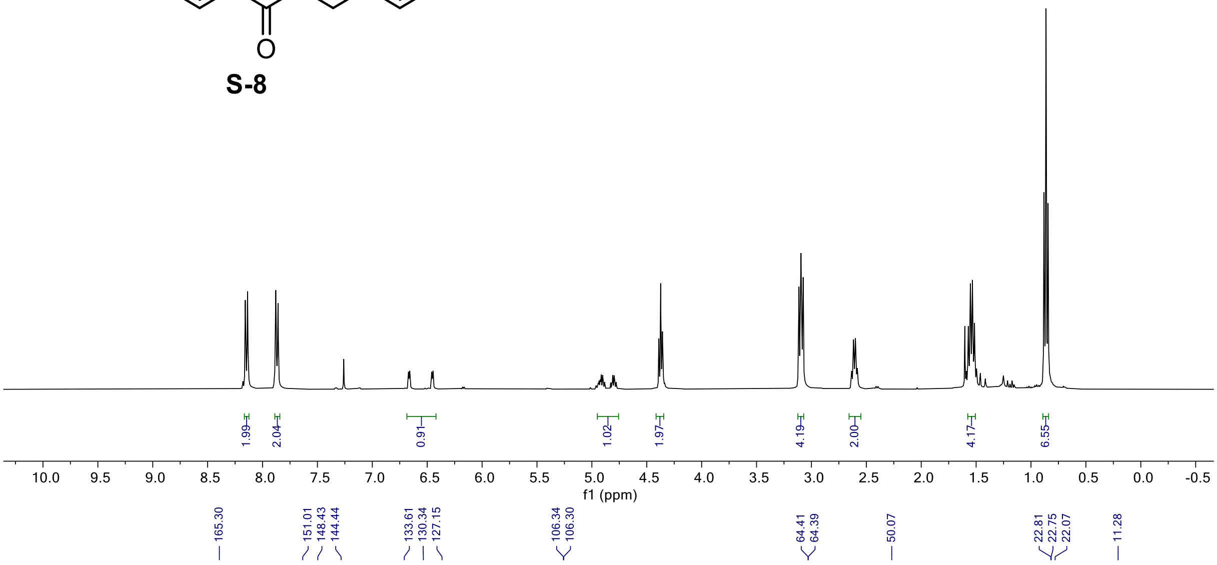
1.99
2.04
0.91
1.02
1.97
4.19
2.00
4.17
6.55
10.0 9.5 9.0 8.5 8.0 7.5 7.0 6.5 6.0 5.5 5.0 4.5 4.0 3.5 3.0 2.5 2.0 1.5 1.0 0.5 0.0 -0.5
f1 (ppm)
165.30
151.01
148.43
144.44
133.61
130.34
127.15
106.34
106.30
64.41
64.39
50.07
22.81
22.75
22.07
11.28

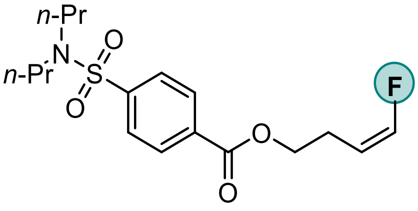
n-Pr
N
n-Pr
S
O
O
O
O
F

S-8

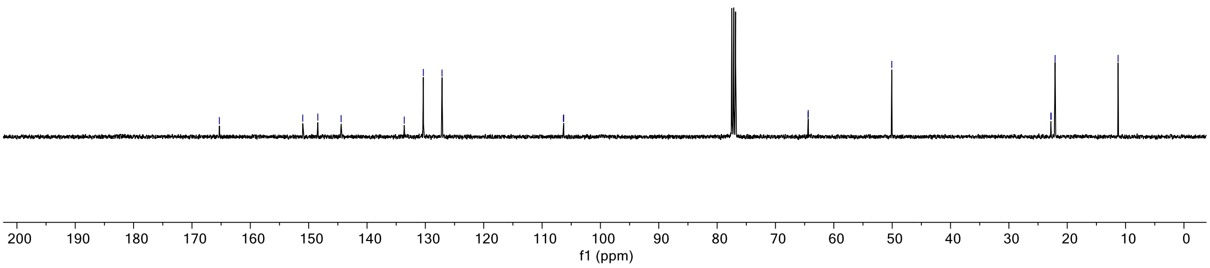
200 190 180 170 160 150 140 130 120 110 100 90 80 70 60 50 40 30 20 10 0
f1 (ppm)

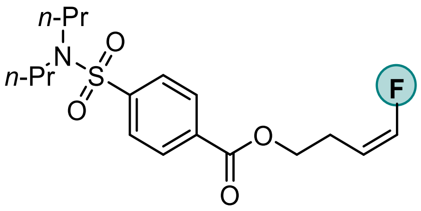
n-Pr
N
n-Pr
S
O
O
O
O
F

S-8

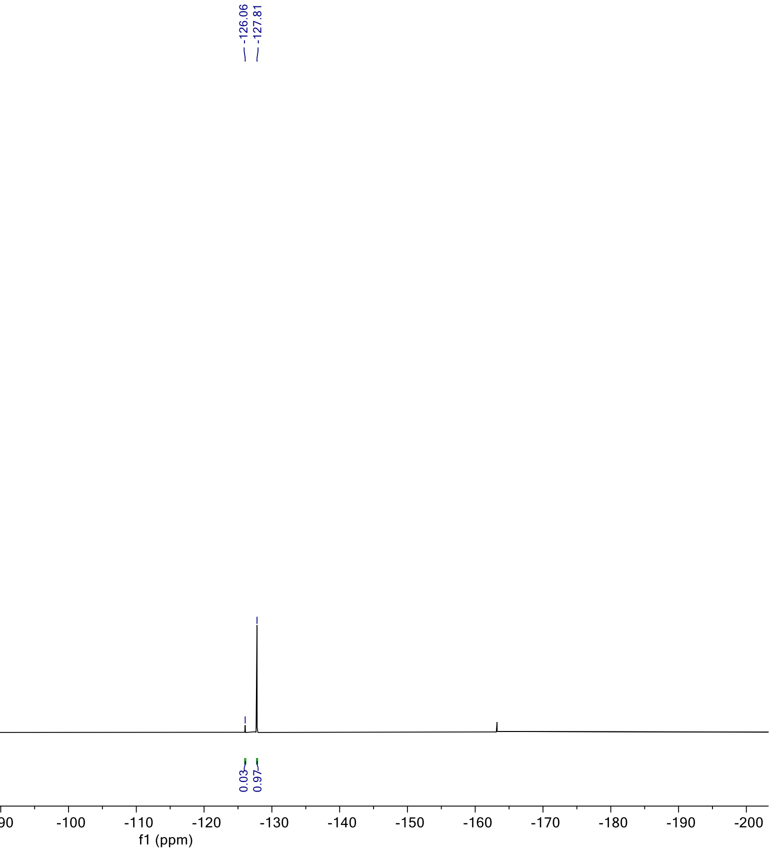
-126.06
-127.81
0.03
0.97
-30
-40
-50
-60
-70
-80
-90
-100
-110
-120
-130
-140
-150
-160
-170
-180
-190
-200
f1 (ppm)

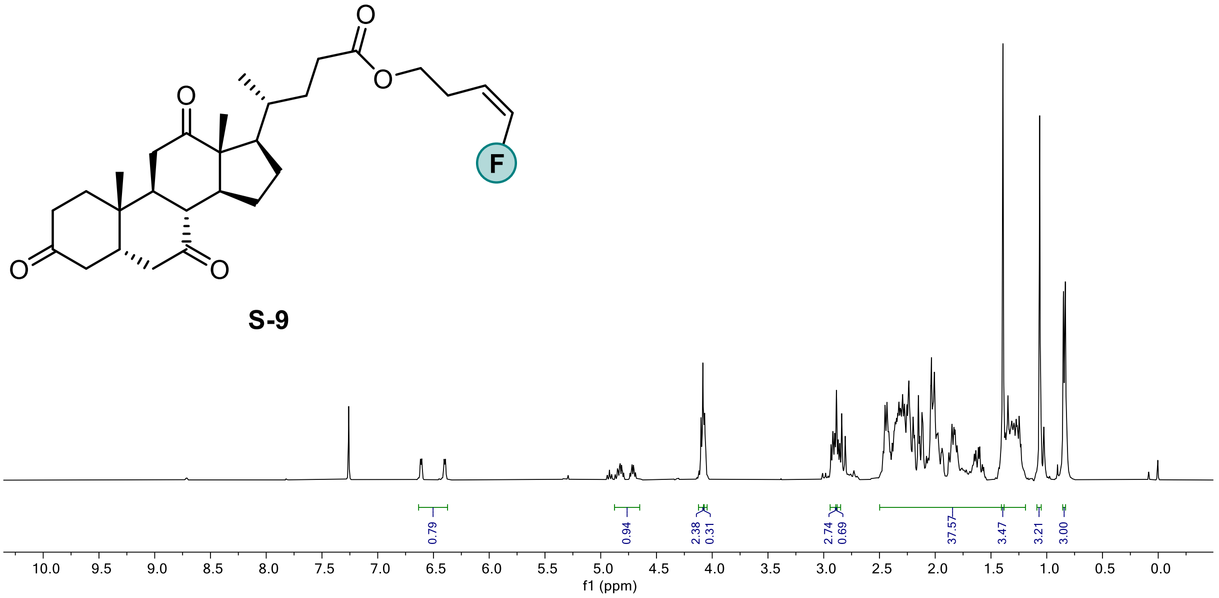
F
0.79
0.94
2.38
0.31
2.74
0.69
37.57
3.47
3.21
3.00
f1 (ppm)

S-9

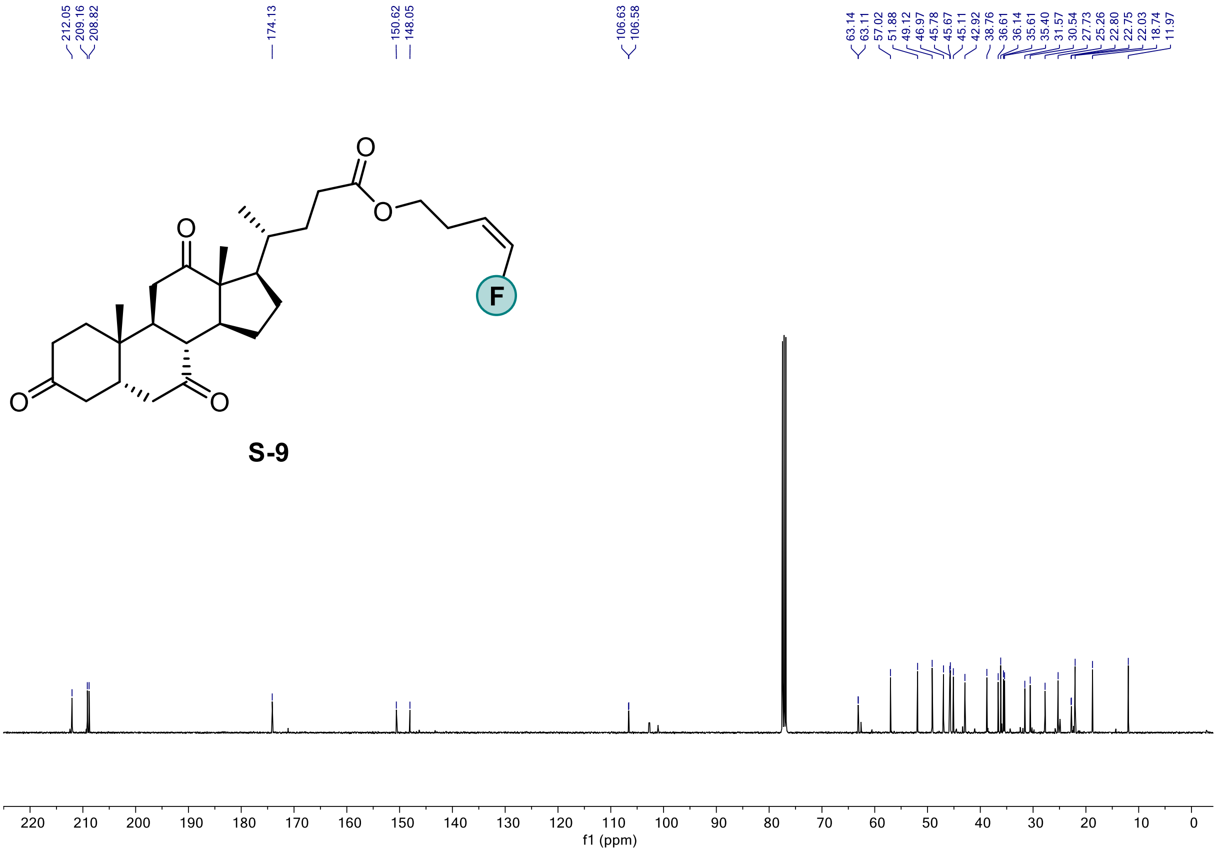
212.05
209.16
208.82
174.13
150.62
148.05
106.63
106.58
63.14
63.11
57.02
51.88
49.12
46.97
45.78
45.67
45.11
42.92
38.76
36.61
36.14
35.61
35.40
31.57
30.54
27.73
25.26
22.80
22.75
22.03
18.74
11.97
F
f1 (ppm)

S-9

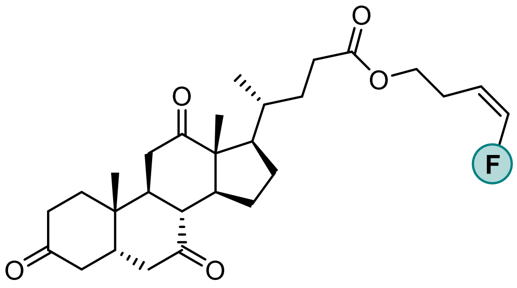

S-9

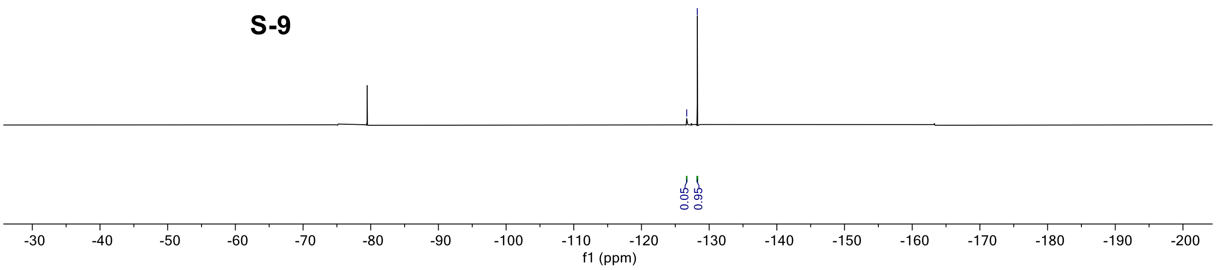

-126.68
-128.25
0.05
0.95
-30 -40 -50 -60 -70 -80 -90 -100 -110 -120 -130 -140 -150 -160 -170 -180 -190 -200
f1 (ppm)

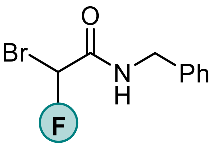

O
Br
N
H
Ph
F

6a

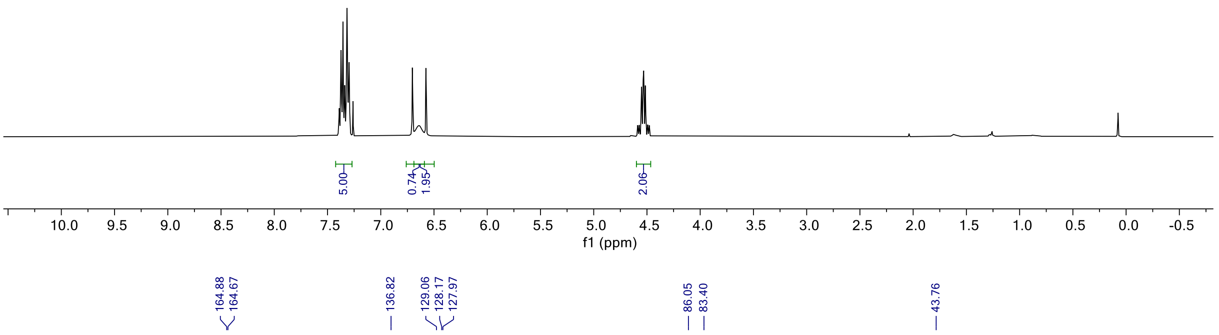

5.00
0.74
1.95
2.06
10.0 9.5 9.0 8.5 8.0 7.5 7.0 6.5 6.0 5.5 5.0 4.5 4.0 3.5 3.0 2.5 2.0 1.5 1.0 0.5 0.0 -0.5
f1 (ppm)
164.88
164.67
136.82
129.06
128.17
127.97
86.05
83.40
43.76

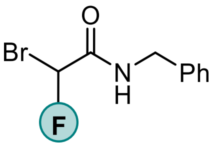

O
Br
N
H
Ph
F

6a

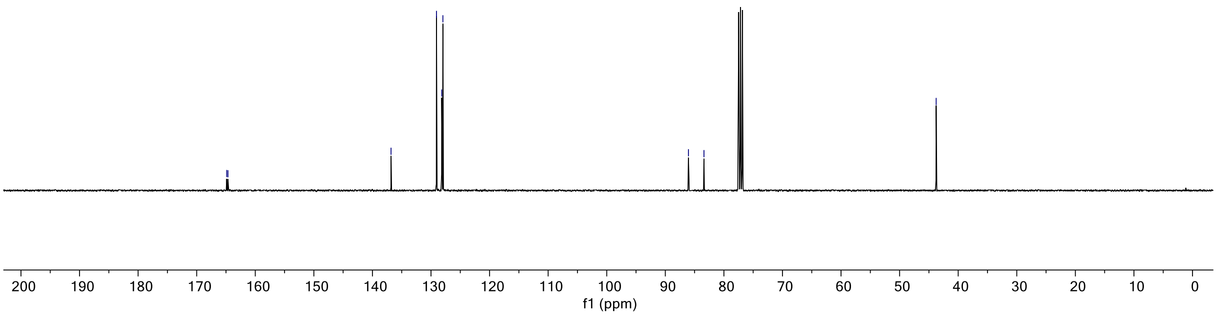

200 190 180 170 160 150 140 130 120 110 100 90 80 70 60 50 40 30 20 10 0
f1 (ppm)

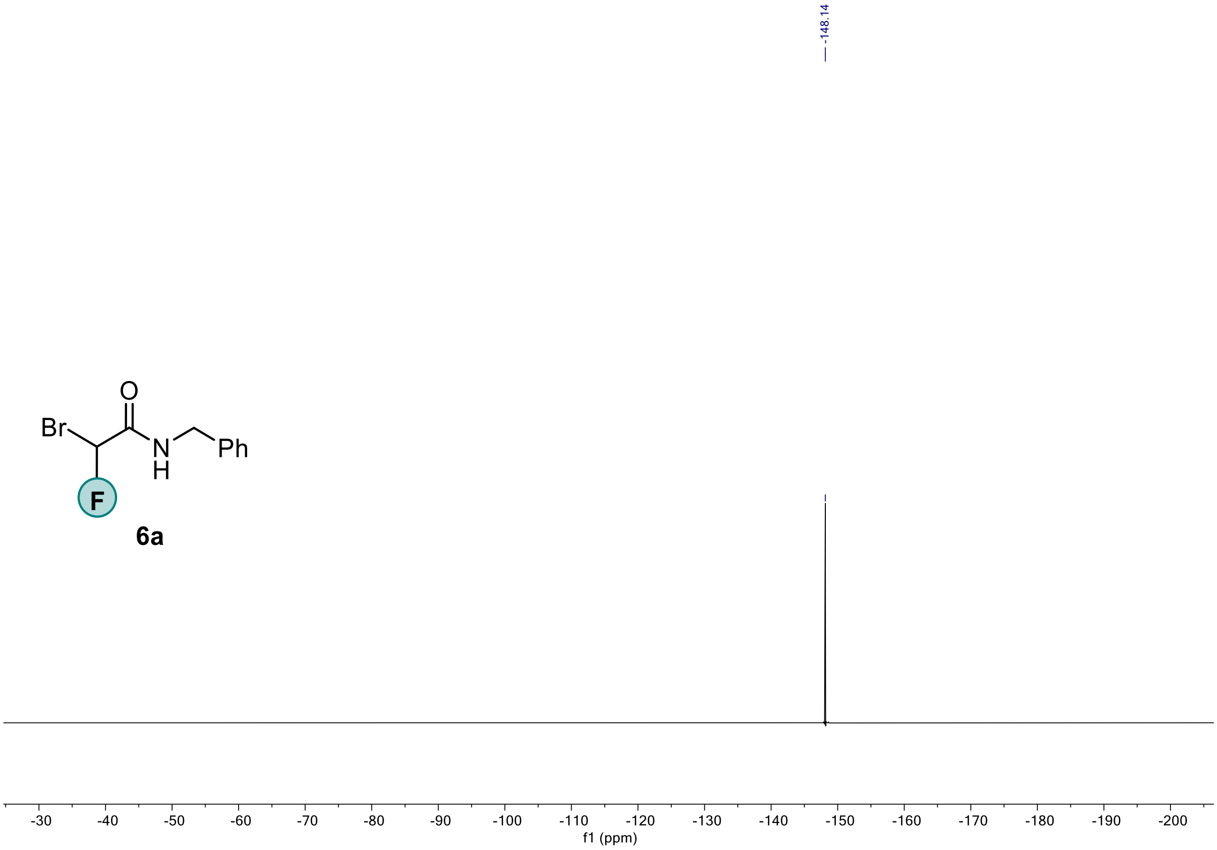
-148.14
Br
O
N
H
Ph
F
-30
-40
-50
-60
-70
-80
-90
-100
-110
-120
-130
-140
-150
-160
-170
-180
-190
-200
f1 (ppm)

6a

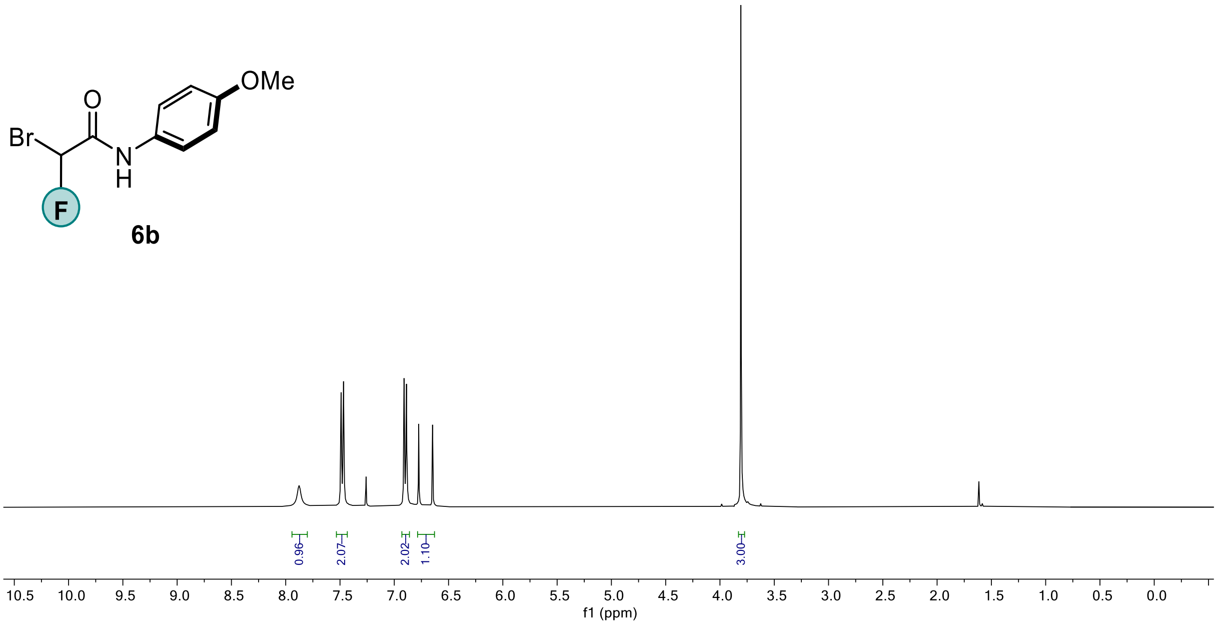

OMe
Br
O
N
H
F
6b
0.96
2.07
2.02
1.10
3.00
10.5 10.0 9.5 9.0 8.5 8.0 7.5 7.0 6.5 6.0 5.5 5.0 4.5 4.0 3.5 3.0 2.5 2.0 1.5 1.0 0.5 0.0
f1 (ppm)

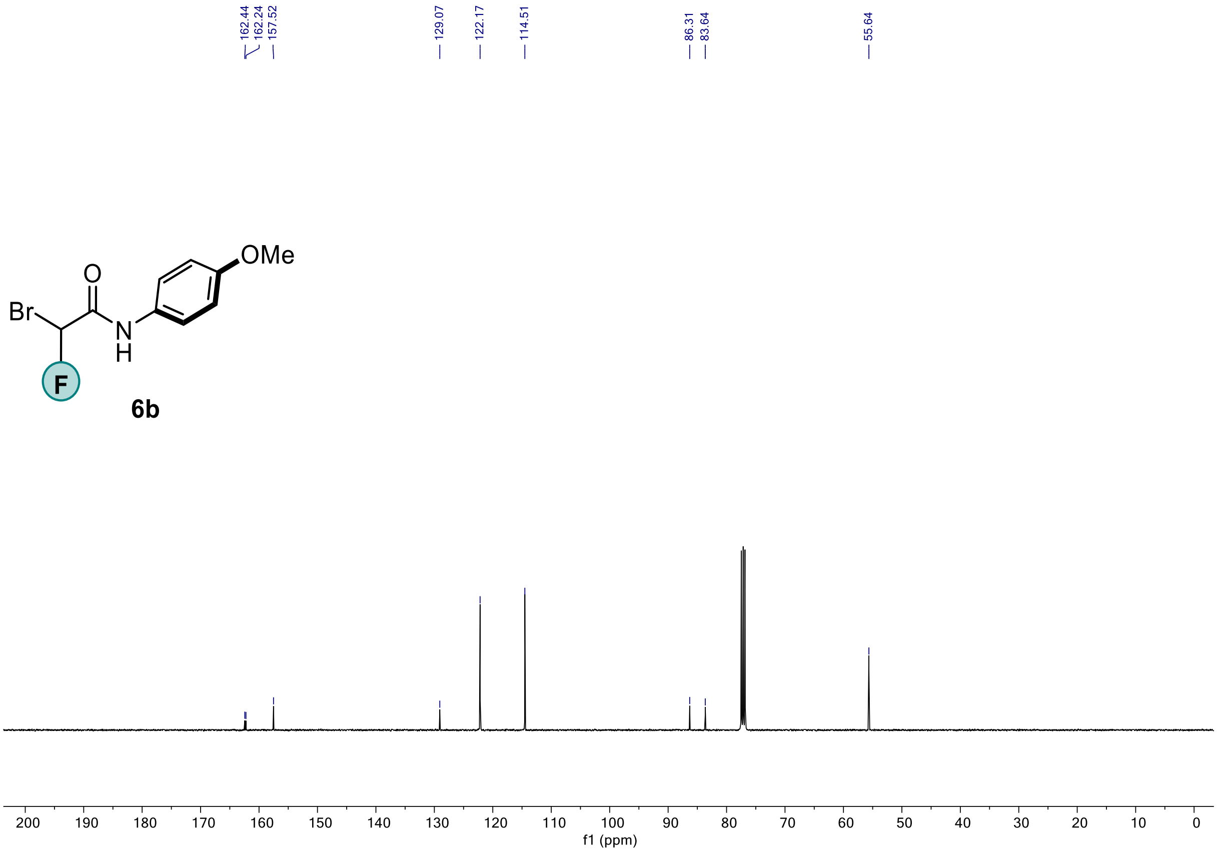

162.44
162.24
157.52
129.07
122.17
114.51
86.31
83.64
55.64
OMe
Br
O
N
H
F
6b
200 190 180 170 160 150 140 130 120 110 100 90 80 70 60 50 40 30 20 10 0
f1 (ppm)

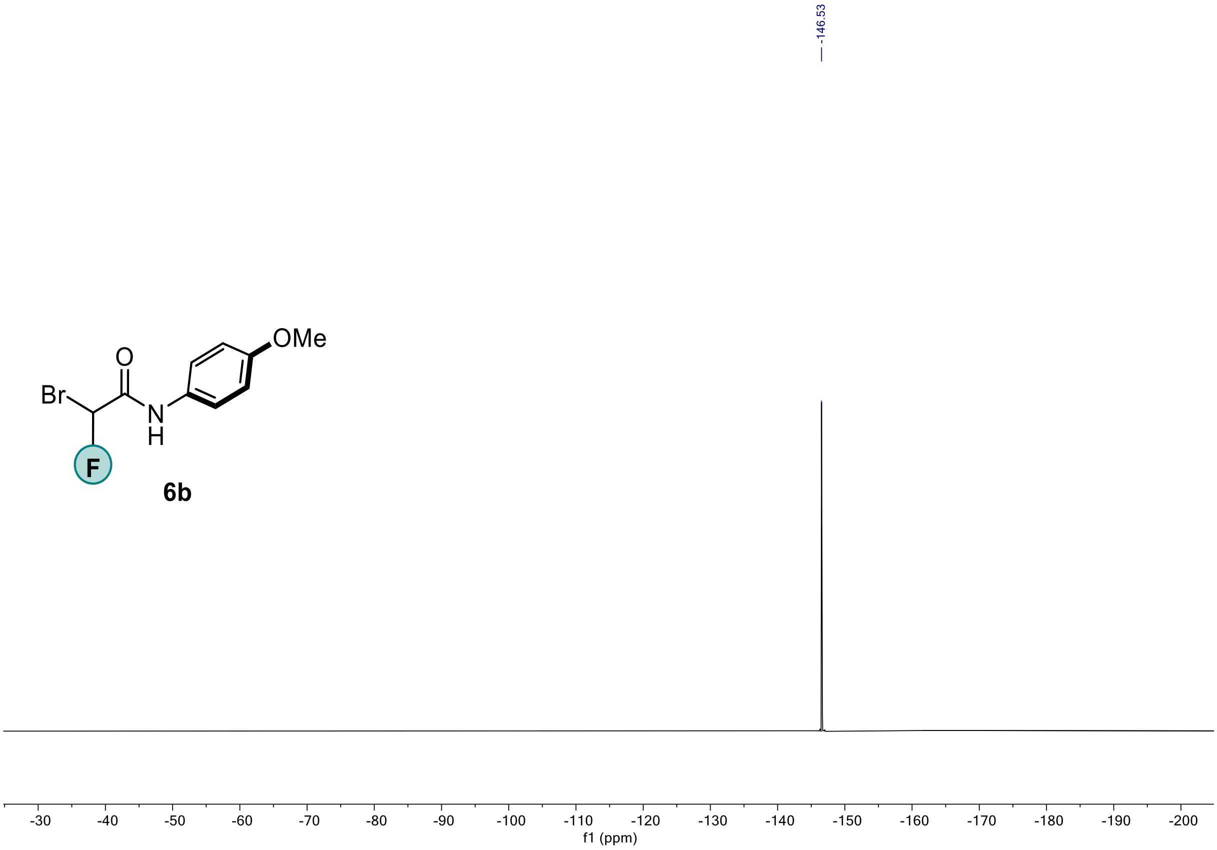
-146.53
OMe
O
Br
N
H
F
6b
-30 -40 -50 -60 -70 -80 -90 -100 -110 -120 -130 -140 -150 -160 -170 -180 -190 -200
f1 (ppm)

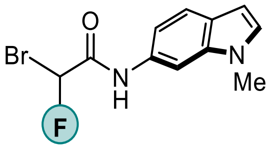

6c

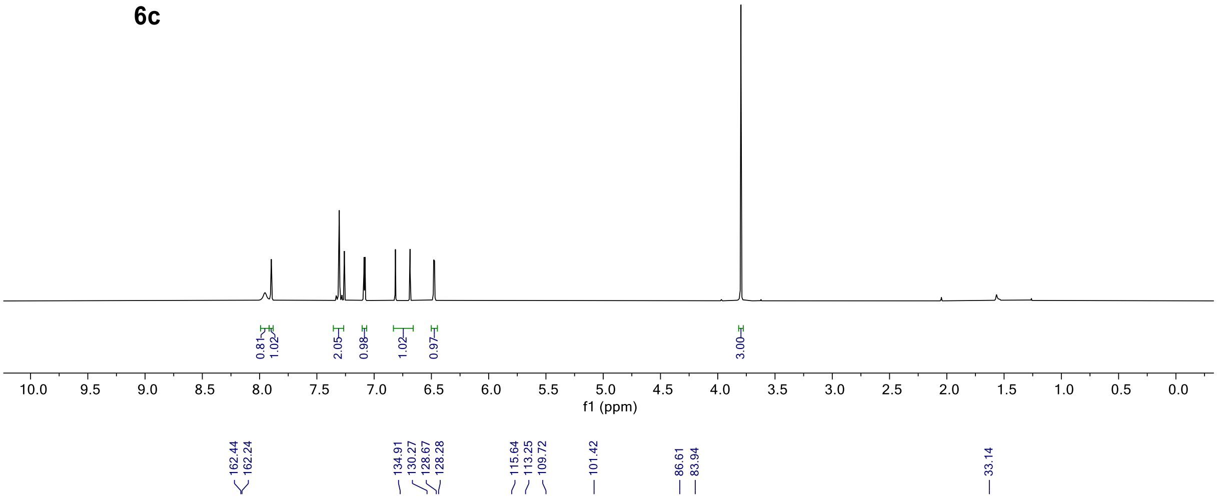

0.81
1.02
2.05
0.98
1.02
0.97
3.00
10.0 9.5 9.0 8.5 8.0 7.5 7.0 6.5 6.0 5.5 5.0 4.5 4.0 3.5 3.0 2.5 2.0 1.5 1.0 0.5 0.0
f1 (ppm)
162.44
162.24
134.91
130.27
128.67
128.28
115.64
113.25
109.72
101.42
86.61
83.94
33.14

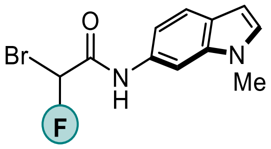

6c

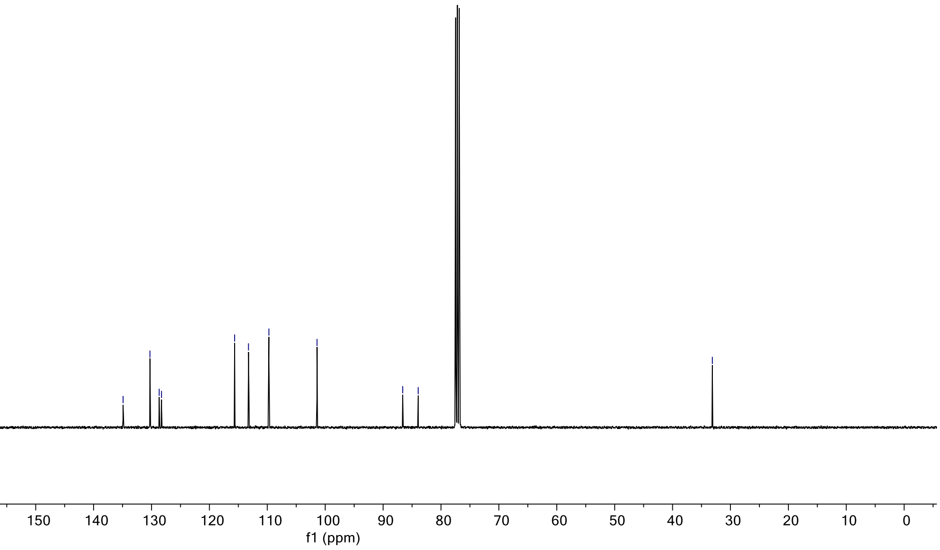

200 190 180 170 160 150 140 130 120 110 100 90 80 70 60 50 40 30 20 10 0
f1 (ppm)

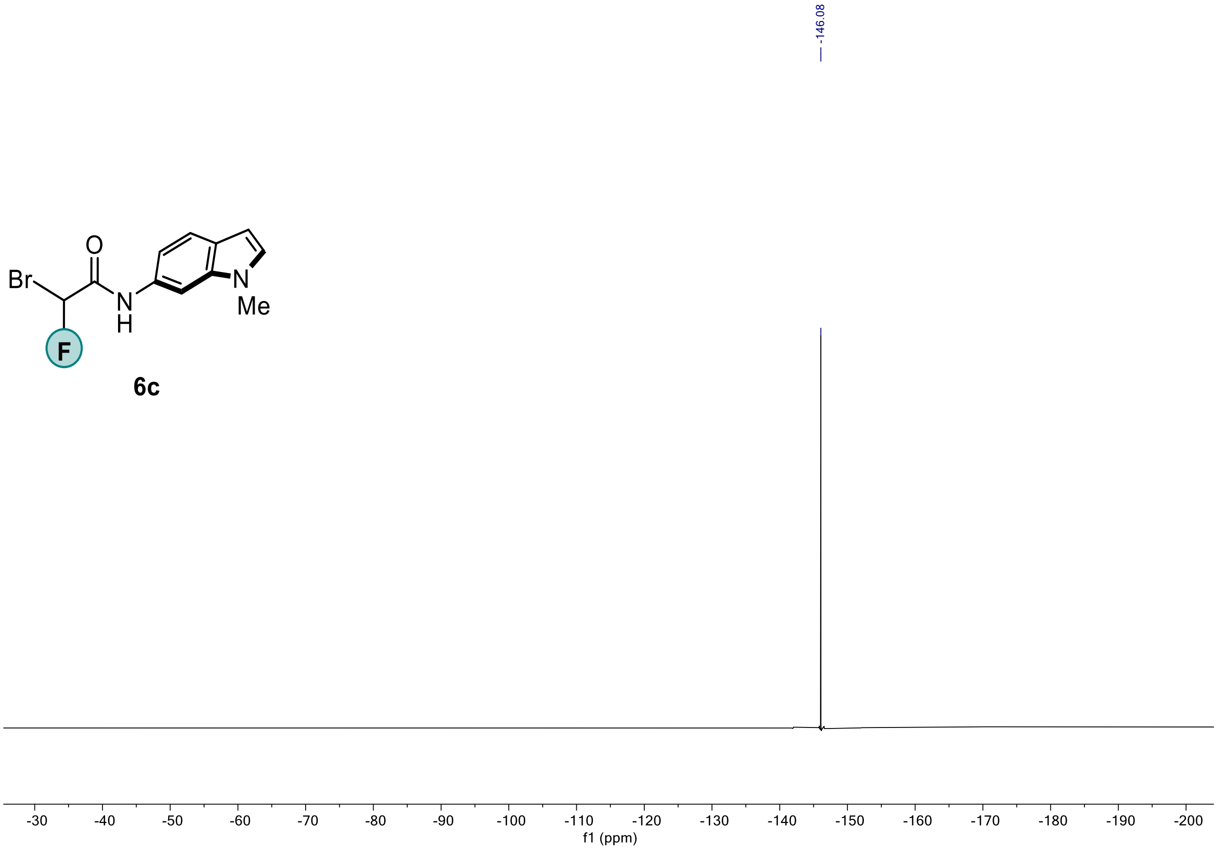

-146.08
-30
-40
-50
-60
-70
-80
-90
-100
-110
-120
-130
-140
-150
-160
-170
-180
-190
-200
f1 (ppm)

6c

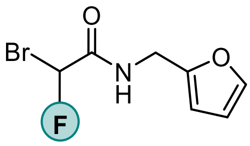
O
Br
N
H
O
F

6d

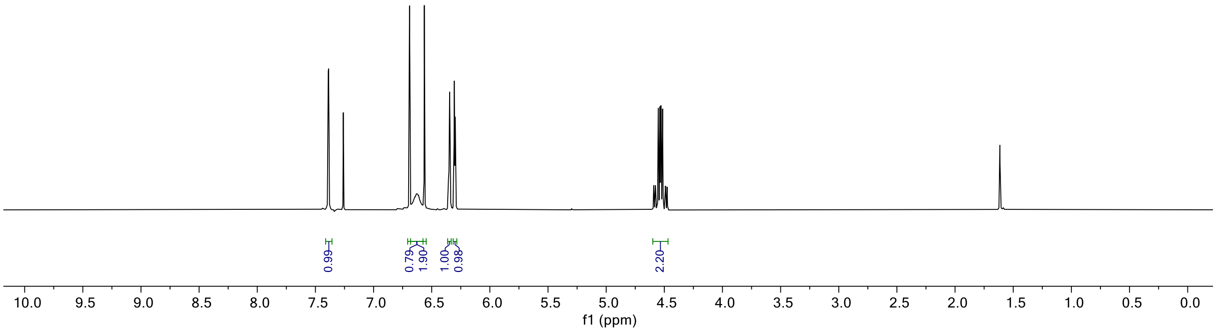
0.99
0.79
1.90
1.00
0.98
2.20
10.0
9.5
9.0
8.5
8.0
7.5
7.0
6.5
6.0
5.5
5.0
4.5
4.0
3.5
3.0
2.5
2.0
1.5
1.0
0.5
0.0
f1 (ppm)

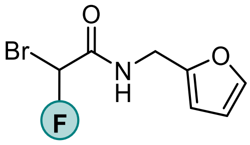
O
Br
N
H
O
F

6d

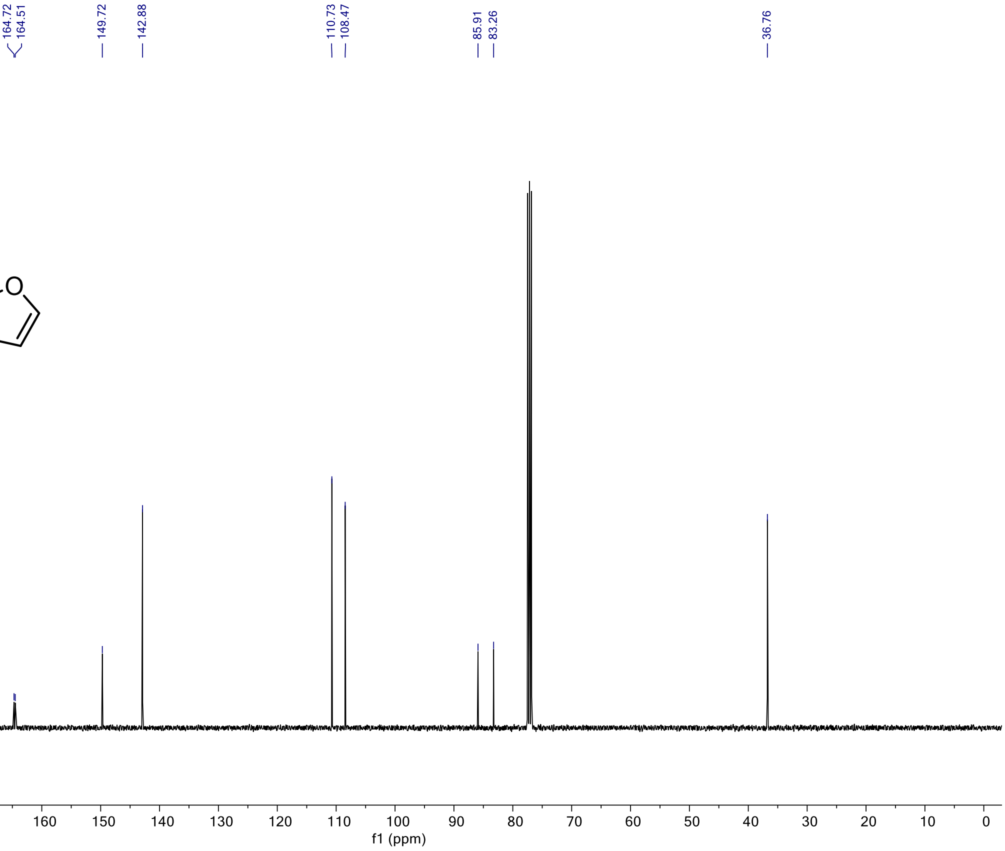
164.72
164.51
149.72
142.88
110.73
108.47
85.91
83.26
36.76
200
190
180
170
160
150
140
130
120
110
100
90
80
70
60
50
40
30
20
10
0
f1 (ppm)

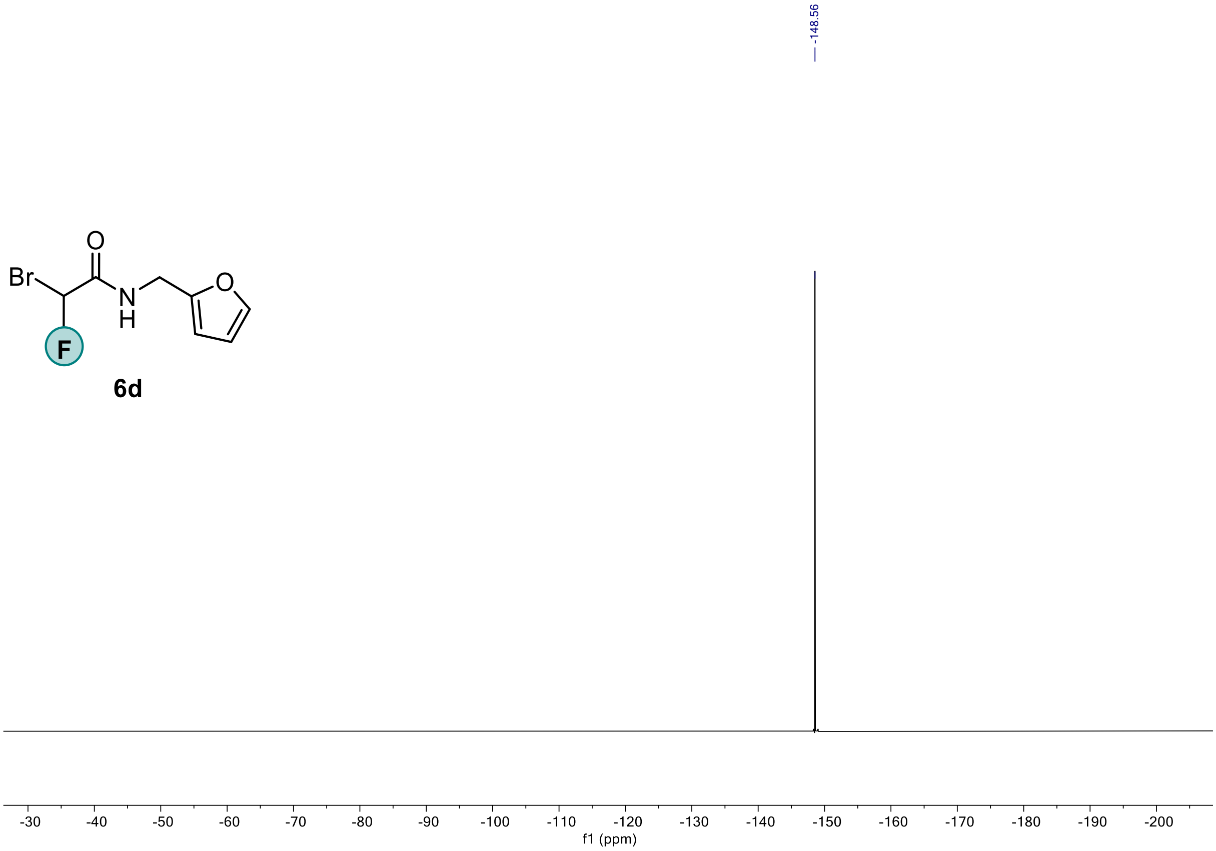
-148.56
Br
O
N
H
O
F
6d
-30 -40 -50 -60 -70 -80 -90 -100 -110 -120 -130 -140 -150 -160 -170 -180 -190 -200
f1 (ppm)

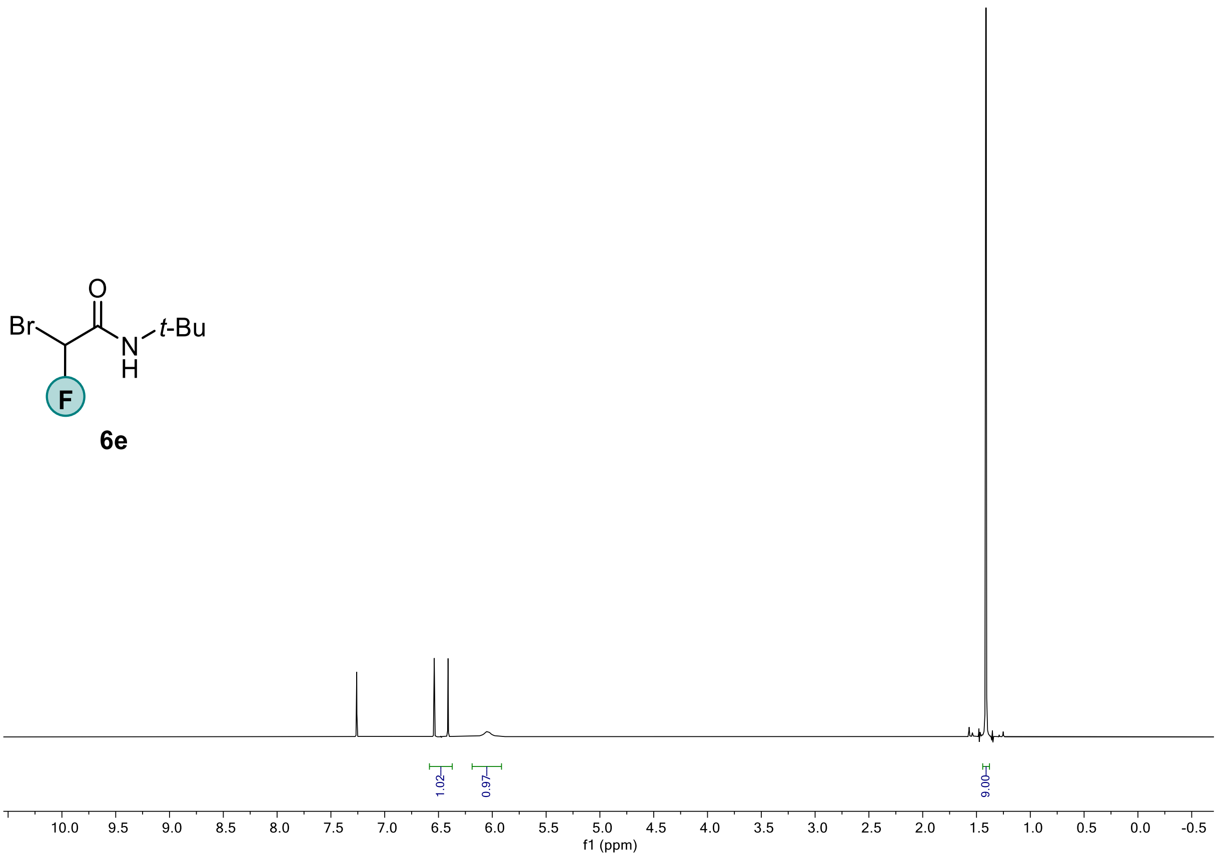
O
Br
N
t-Bu
H
F
1.02
0.97
9.00
10.0
9.5
9.0
8.5
8.0
7.5
7.0
6.5
6.0
5.5
5.0
4.5
4.0
3.5
3.0
2.5
2.0
1.5
1.0
0.5
0.0
-0.5
f1 (ppm)

6e

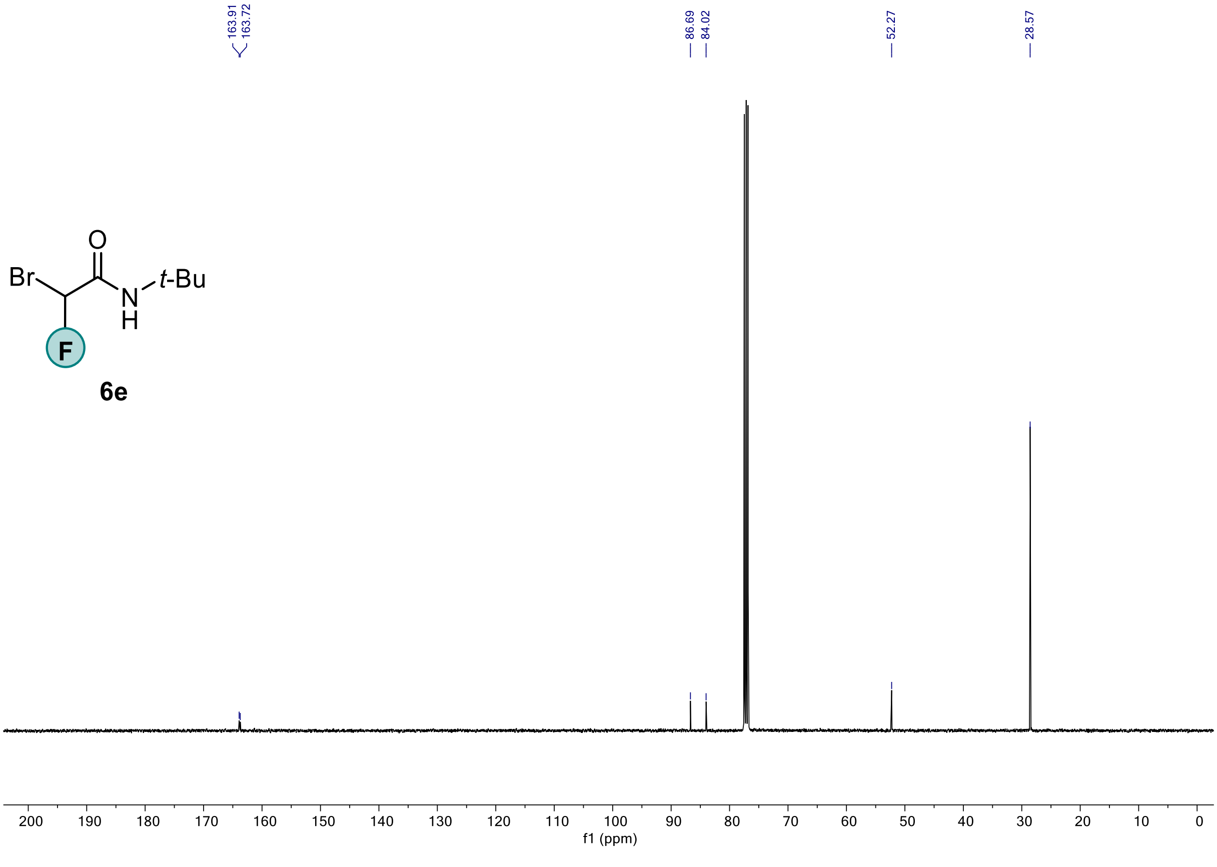
163.91
163.72
86.69
84.02
52.27
28.57
O
Br
N
t-Bu
H
F
200
190
180
170
160
150
140
130
120
110
100
90
80
70
60
50
40
30
20
10
0
f1 (ppm)

6e

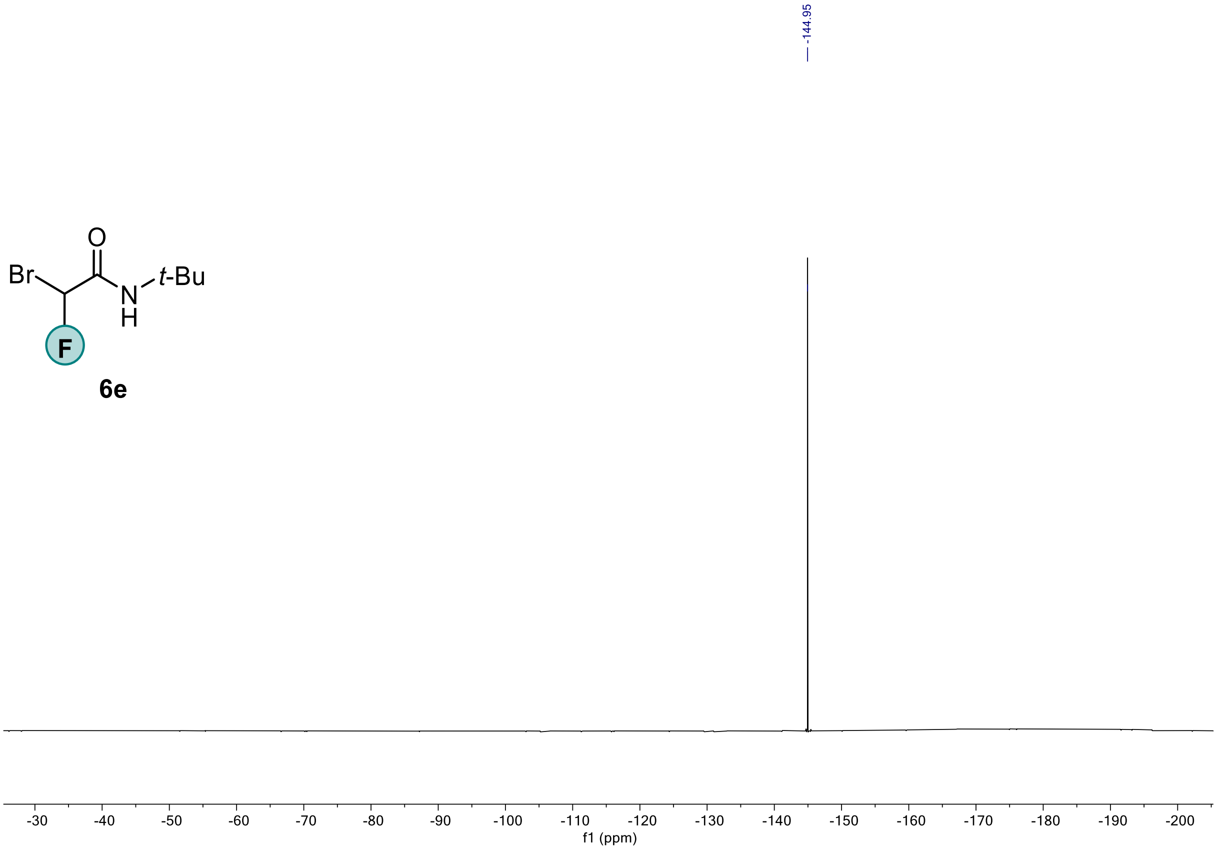

Br
O
N
H
t-Bu
F
6e
-144.95
-30
-40
-50
-60
-70
-80
-90
-100
-110
-120
-130
-140
-150
-160
-170
-180
-190
-200
f1 (ppm)

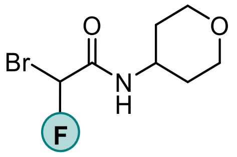
O
Br
N
H
O
F

6f

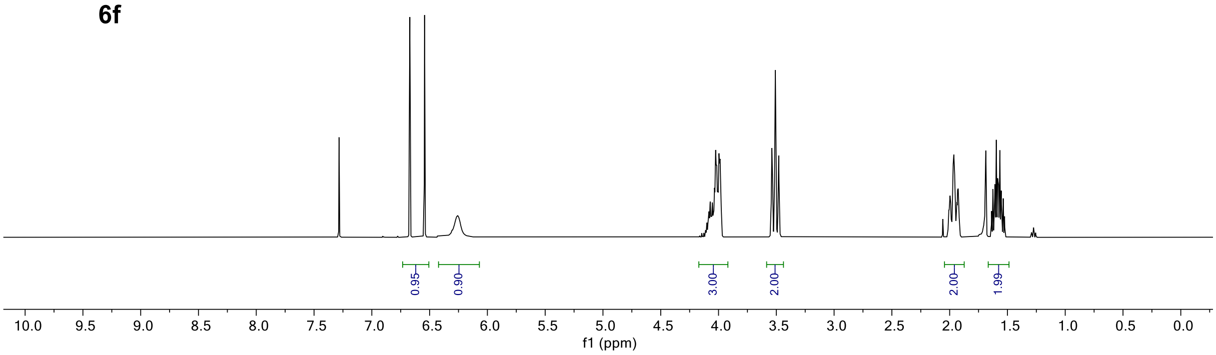
0.95
0.90
3.00
2.00
2.00
1.99
10.0 9.5 9.0 8.5 8.0 7.5 7.0 6.5 6.0 5.5 5.0 4.5 4.0 3.5 3.0 2.5 2.0 1.5 1.0 0.5 0.0
f1 (ppm)

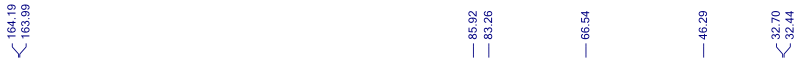
164.19
163.99
85.92
83.26
66.54
46.29
32.70
32.44

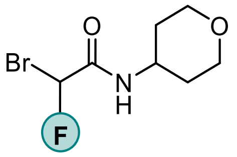
O
Br
N
H
O
F

6f

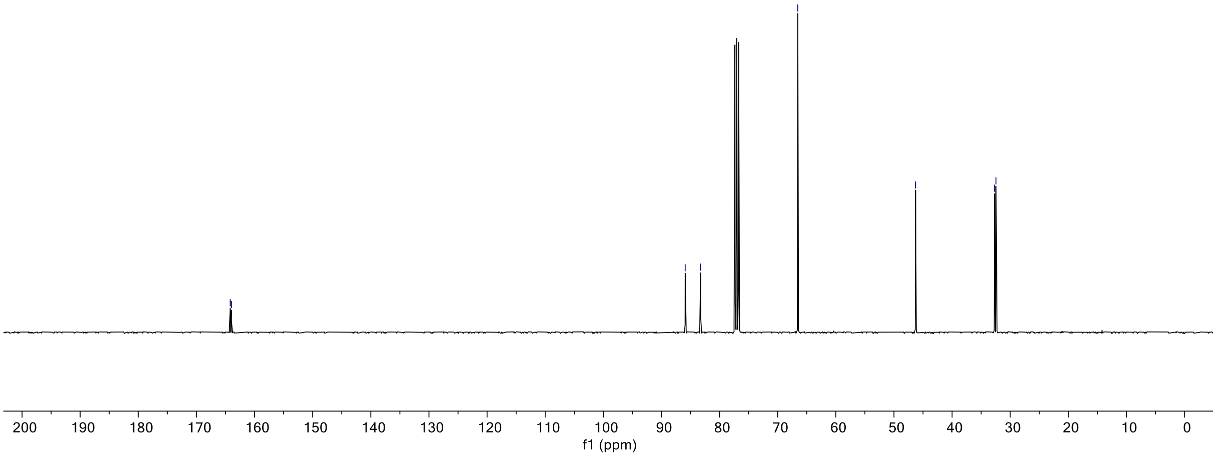
200 190 180 170 160 150 140 130 120 110 100 90 80 70 60 50 40 30 20 10 0
f1 (ppm)

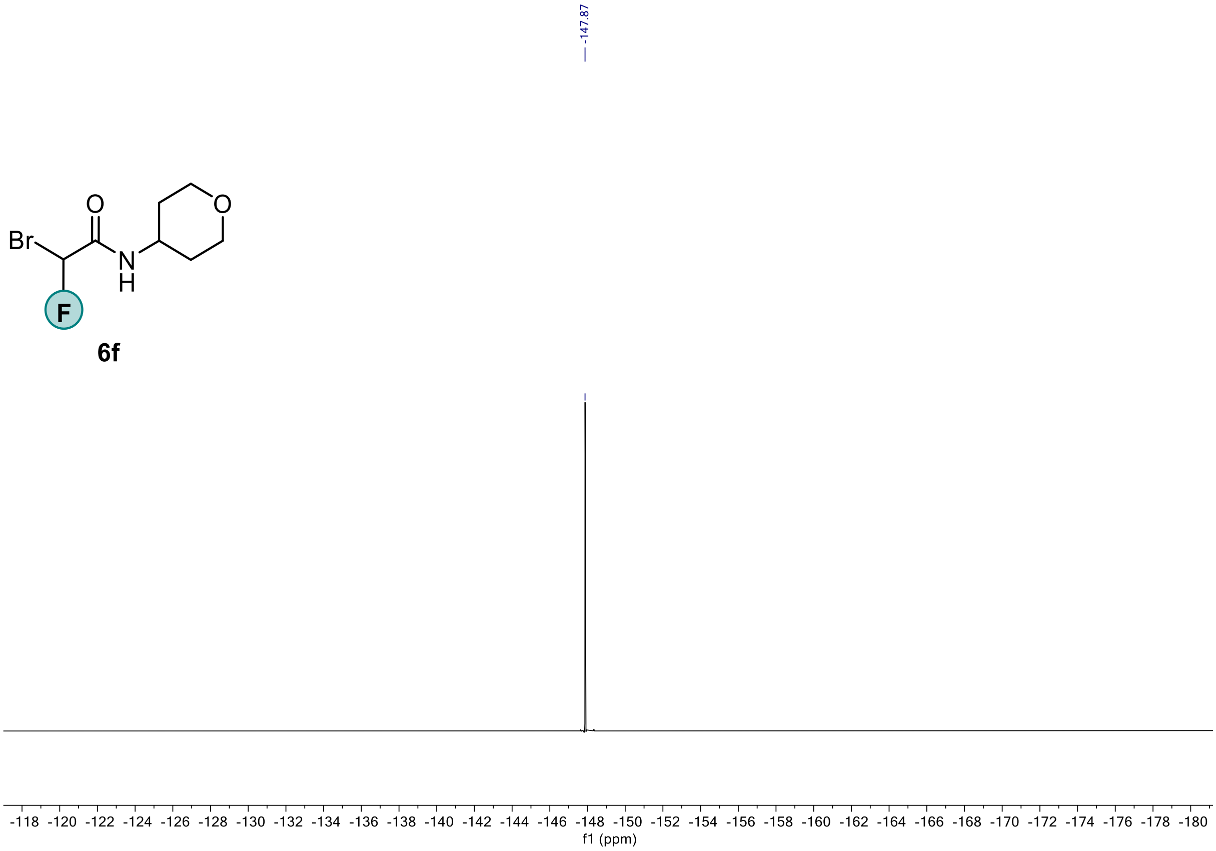
-147.87
Br
O
N
H
O
F
6f
-118 -120 -122 -124 -126 -128 -130 -132 -134 -136 -138 -140 -142 -144 -146 -148 -150 -152 -154 -156 -158 -160 -162 -164 -166 -168 -170 -172 -174 -176 -178 -180
f1 (ppm)

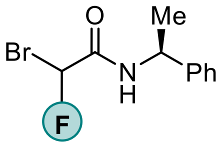
O
Me
Br
N
H
Ph
F

6g

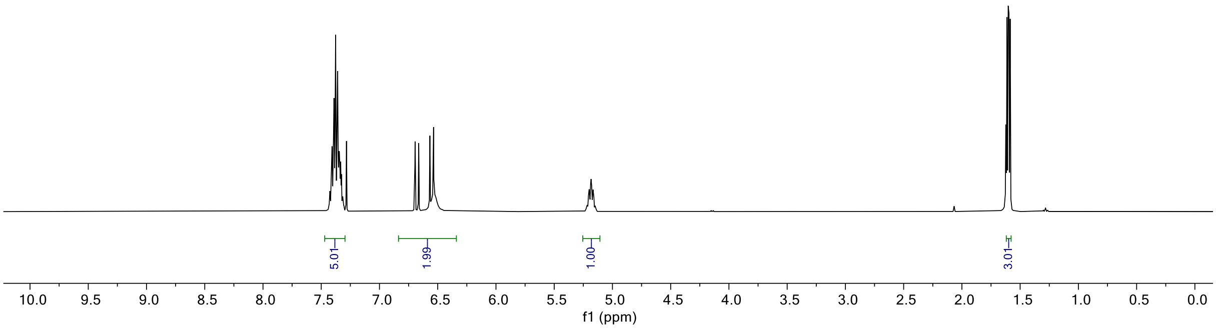
5.01
1.99
1.00
3.01
10.0
9.5
9.0
8.5
8.0
7.5
7.0
6.5
6.0
5.5
5.0
4.5
4.0
3.5
3.0
2.5
2.0
1.5
1.0
0.5
0.0
f1 (ppm)

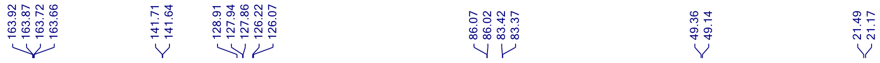
163.92
163.87
163.72
163.66
141.71
141.64
128.91
127.94
127.86
126.22
126.07
86.07
86.02
83.42
83.37
49.36
49.14
21.49
21.17

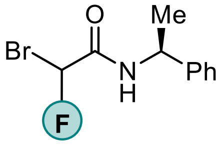
O
Me
Br
N
H
Ph
F

6g

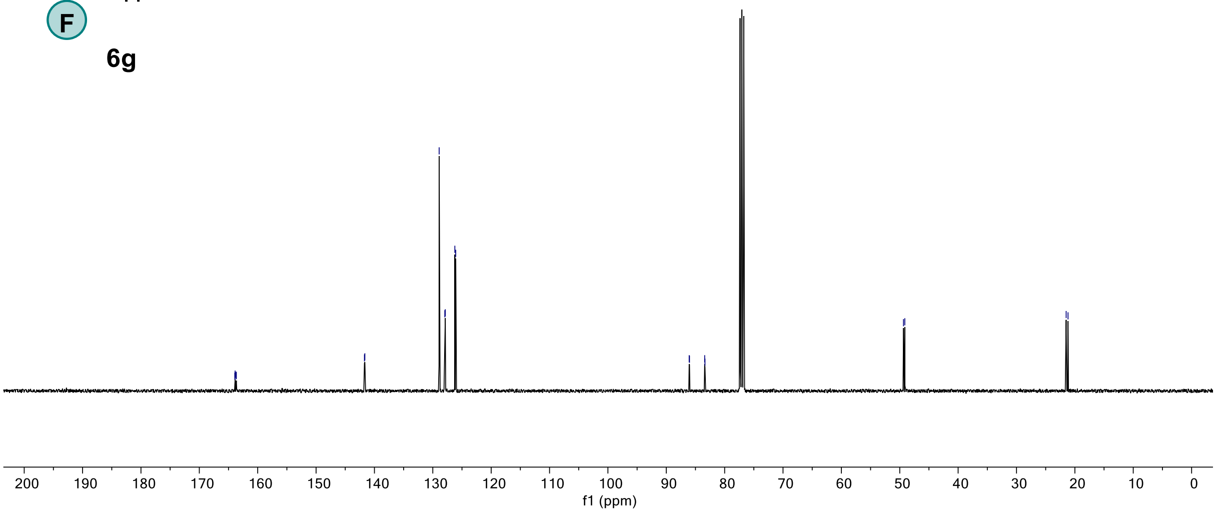
200
190
180
170
160
150
140
130
120
110
100
90
80
70
60
50
40
30
20
10
0
f1 (ppm)

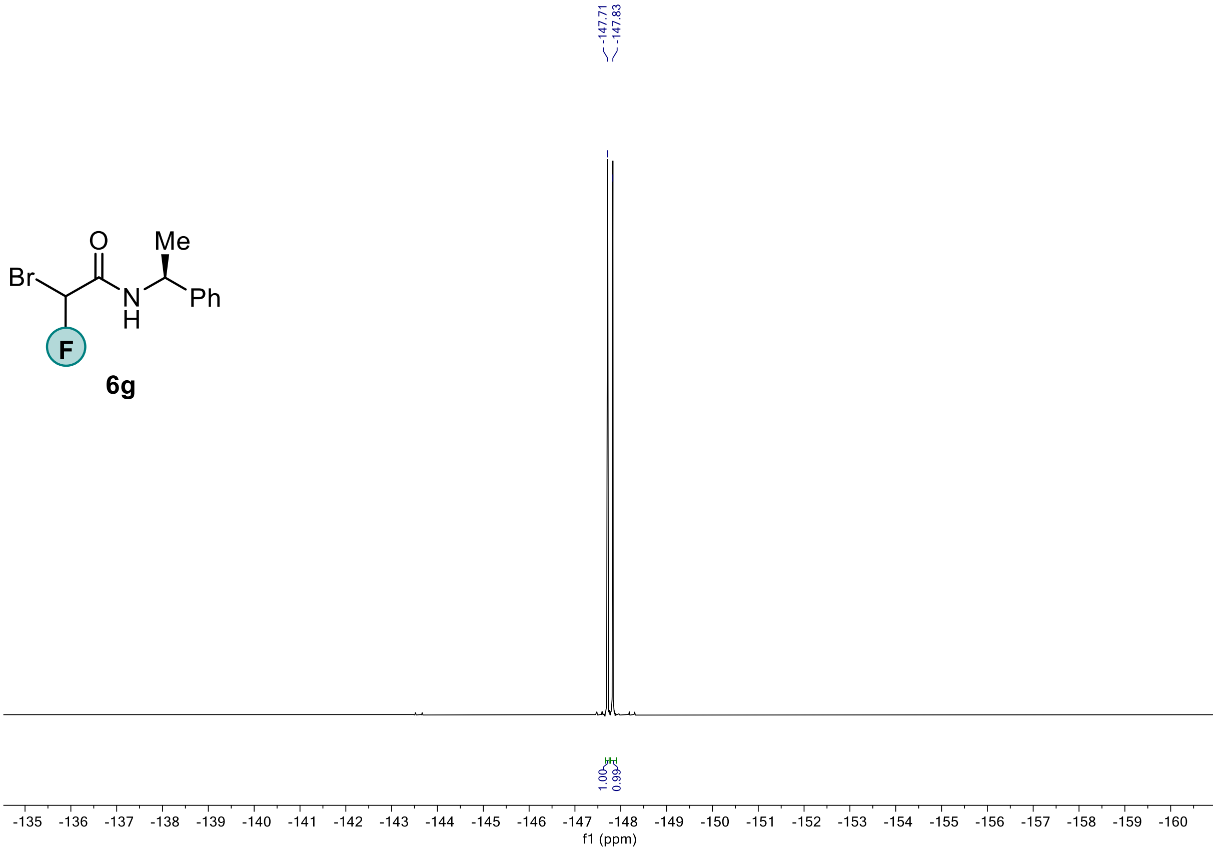
-147.71
-147.83
1.00
0.99
-135
-136
-137
-138
-139
-140
-141
-142
-143
-144
-145
-146
-147
-148
-149
-150
-151
-152
-153
-154
-155
-156
-157
-158
-159
-160
f1 (ppm)

6g

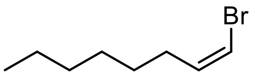
Br

S-11

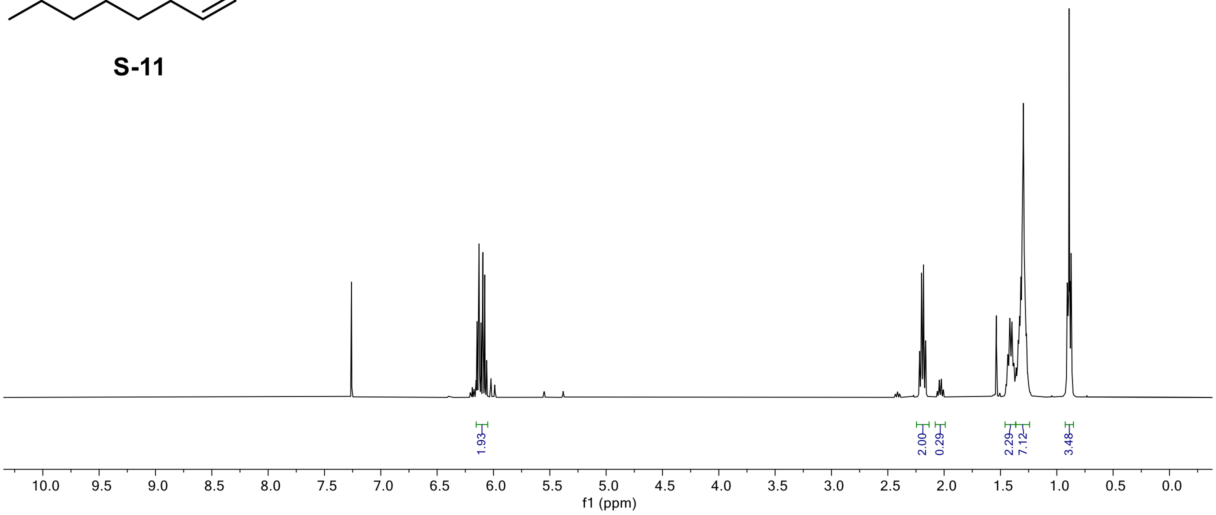
1.93
2.00
0.29
2.29
7.12
3.48
f1 (ppm)

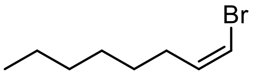
Br

S-11

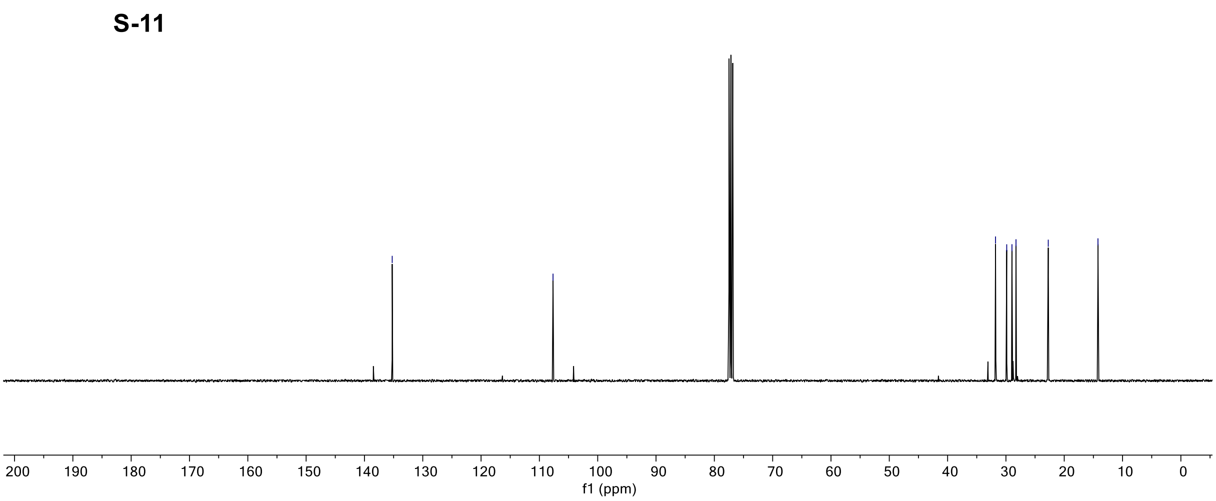
135.24
107.66
31.78
29.86
28.97
28.27
22.74
14.22
f1 (ppm)

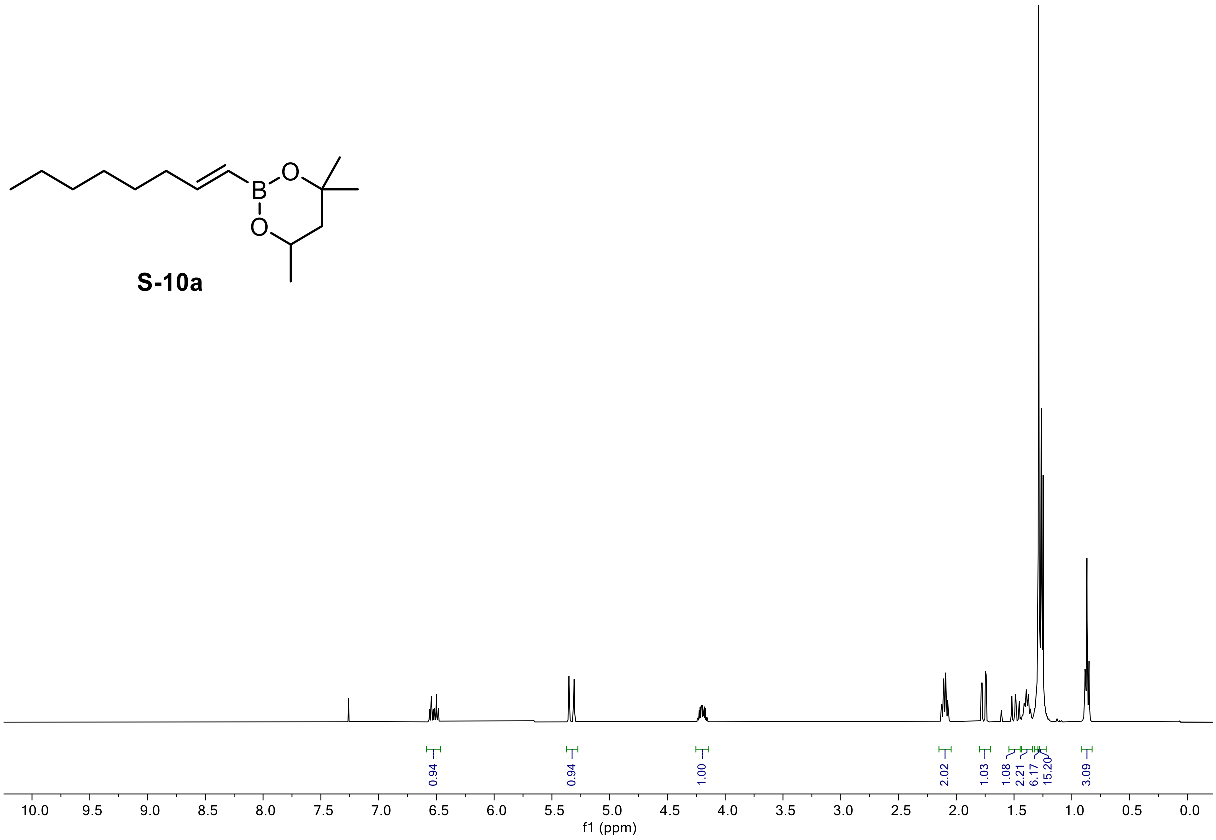
B
O
O
S-10a
0.94
0.94
1.00
2.02
1.03
1.08
2.21
6.17
15.20
3.09
10.0 9.5 9.0 8.5 8.0 7.5 7.0 6.5 6.0 5.5 5.0 4.5 4.0 3.5 3.0 2.5 2.0 1.5 1.0 0.5 0.0
f1 (ppm)

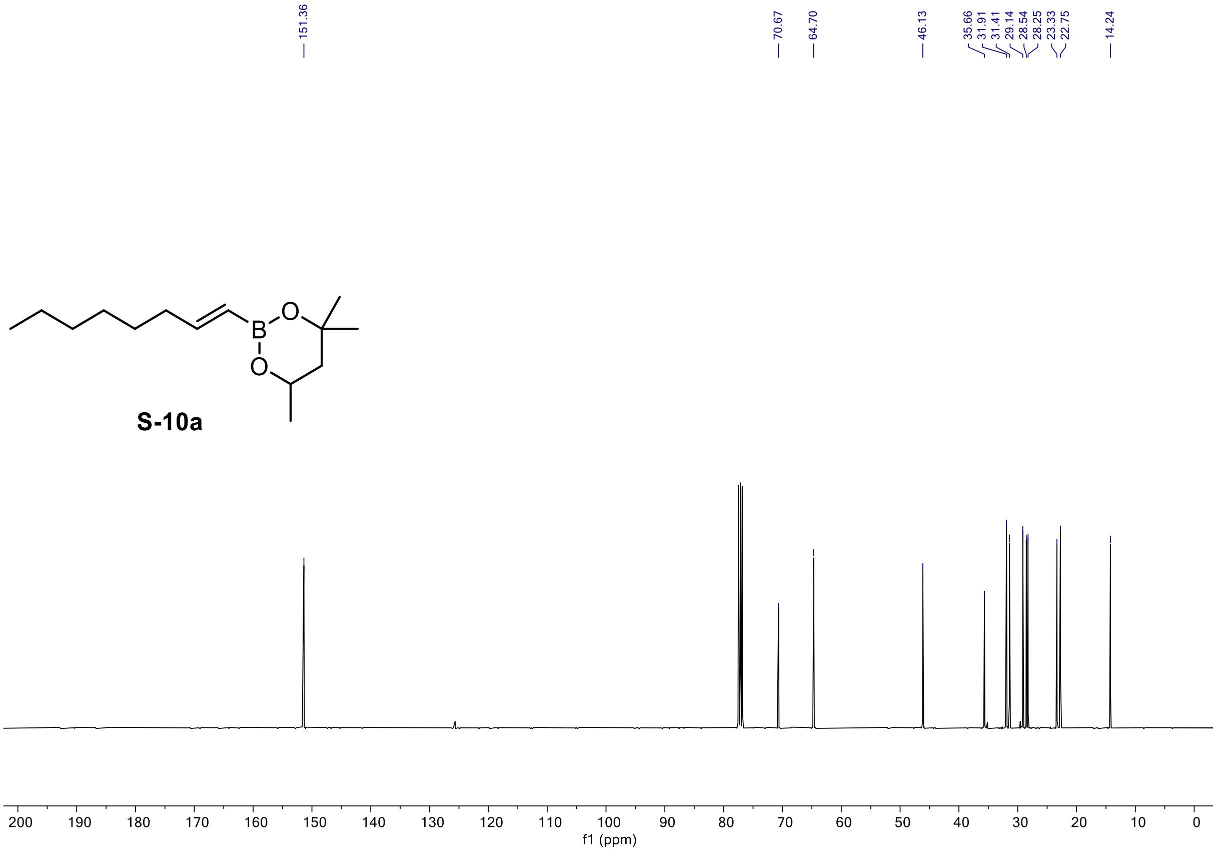
B
O
O
S-10a
151.36
70.67
64.70
46.13
35.66
31.91
31.41
29.14
28.54
28.25
23.33
22.75
14.24
200 190 180 170 160 150 140 130 120 110 100 90 80 70 60 50 40 30 20 10 0
f1 (ppm)

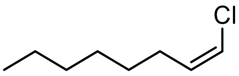
Cl

S-11

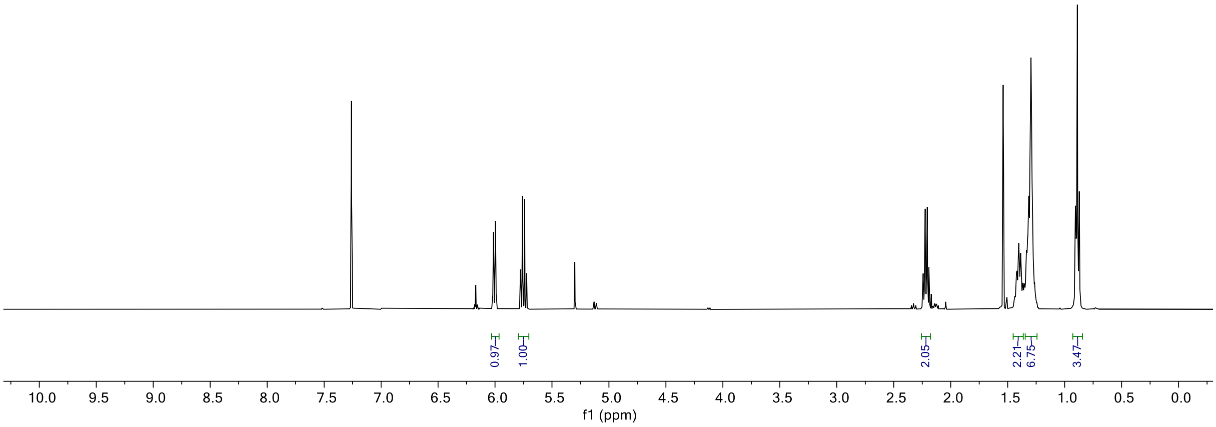
0.97
1.00
2.05
2.21
6.75
3.47
10.0 9.5 9.0 8.5 8.0 7.5 7.0 6.5 6.0 5.5 5.0 4.5 4.0 3.5 3.0 2.5 2.0 1.5 1.0 0.5 0.0
f1 (ppm)

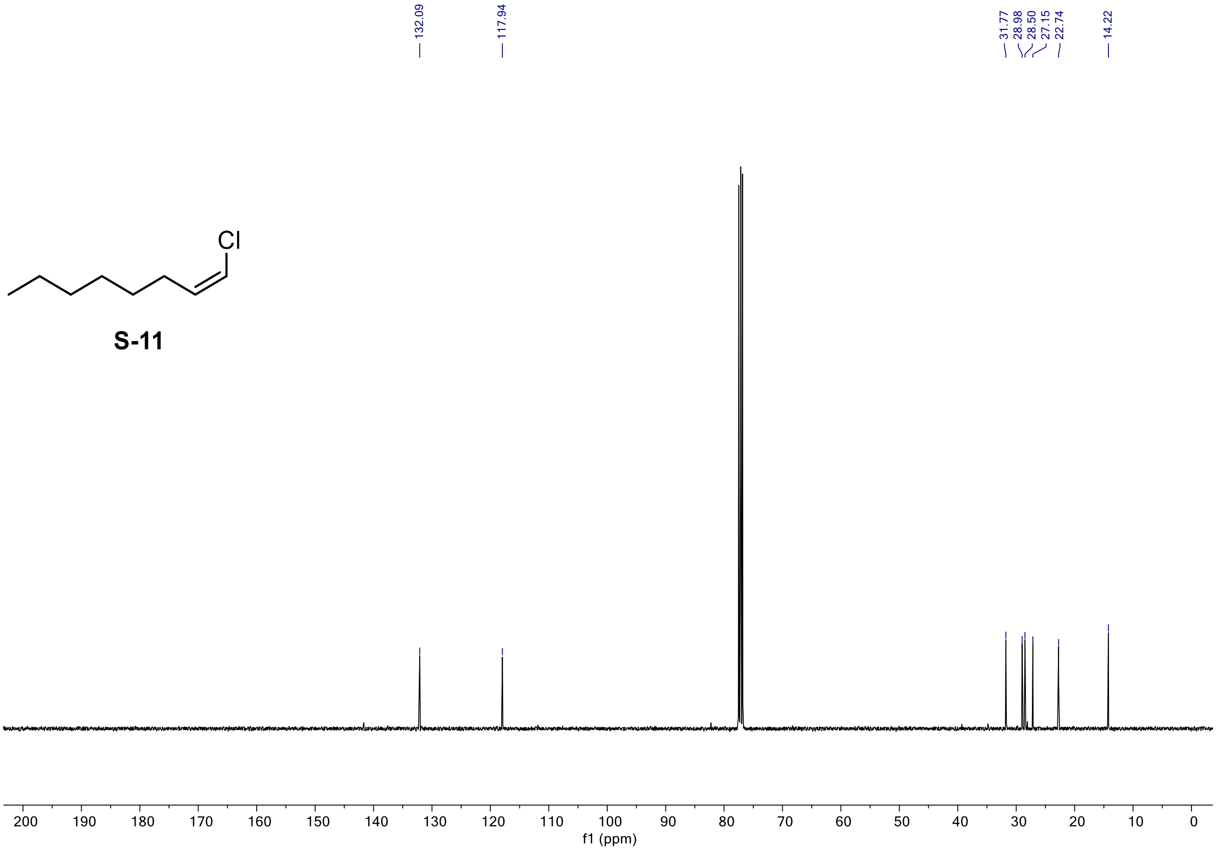
132.09
117.94
31.77
28.98
28.50
27.15
22.74
14.22
Cl
S-11
200 190 180 170 160 150 140 130 120 110 100 90 80 70 60 50 40 30 20 10 0
f1 (ppm)

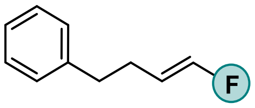

(*E*)-**1a**

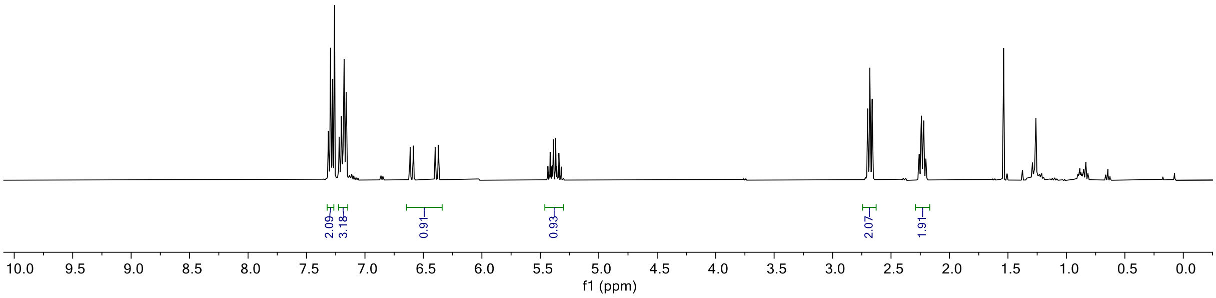

2.09
3.18
0.91
0.93
2.07
1.91
f1 (ppm)

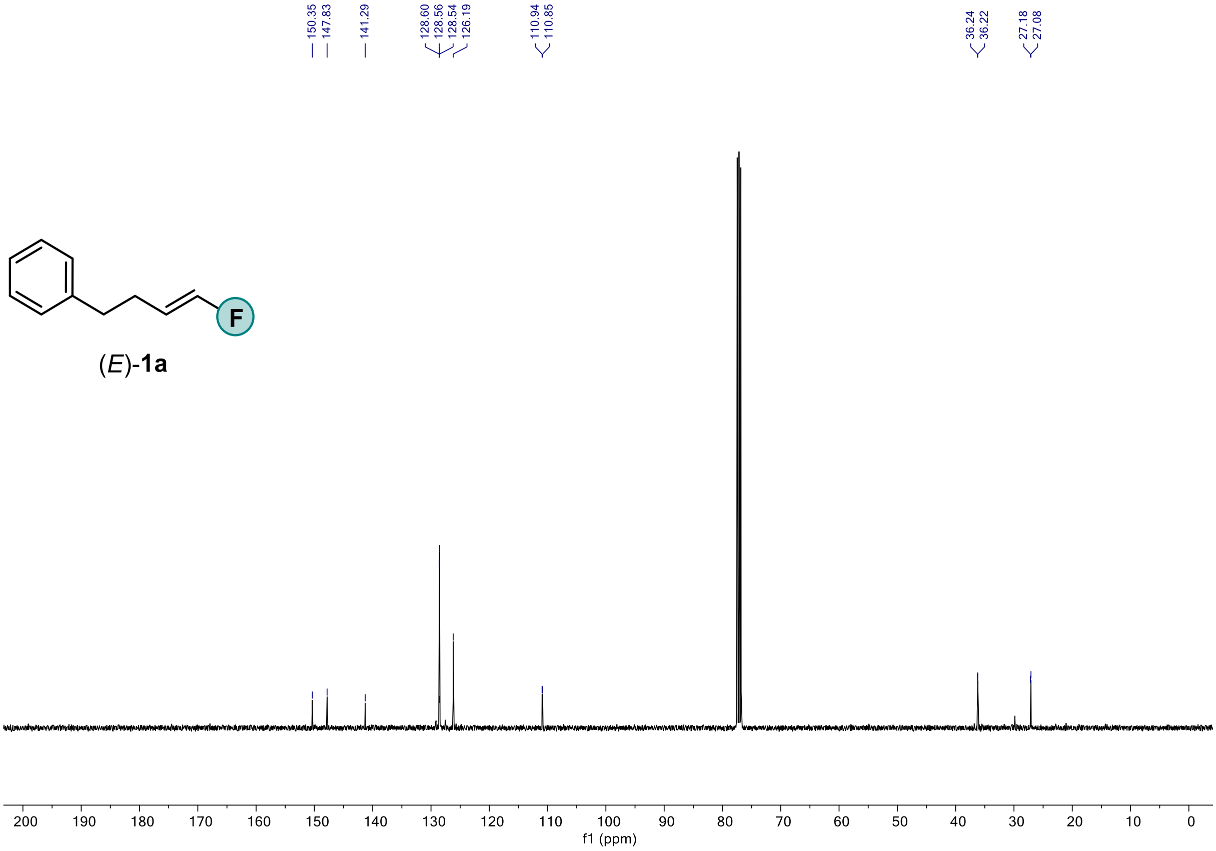

150.35
147.83
141.29
128.60
128.56
128.54
126.19
110.94
110.85
36.24
36.22
27.18
27.08
f1 (ppm)

(*E*)-**1a**

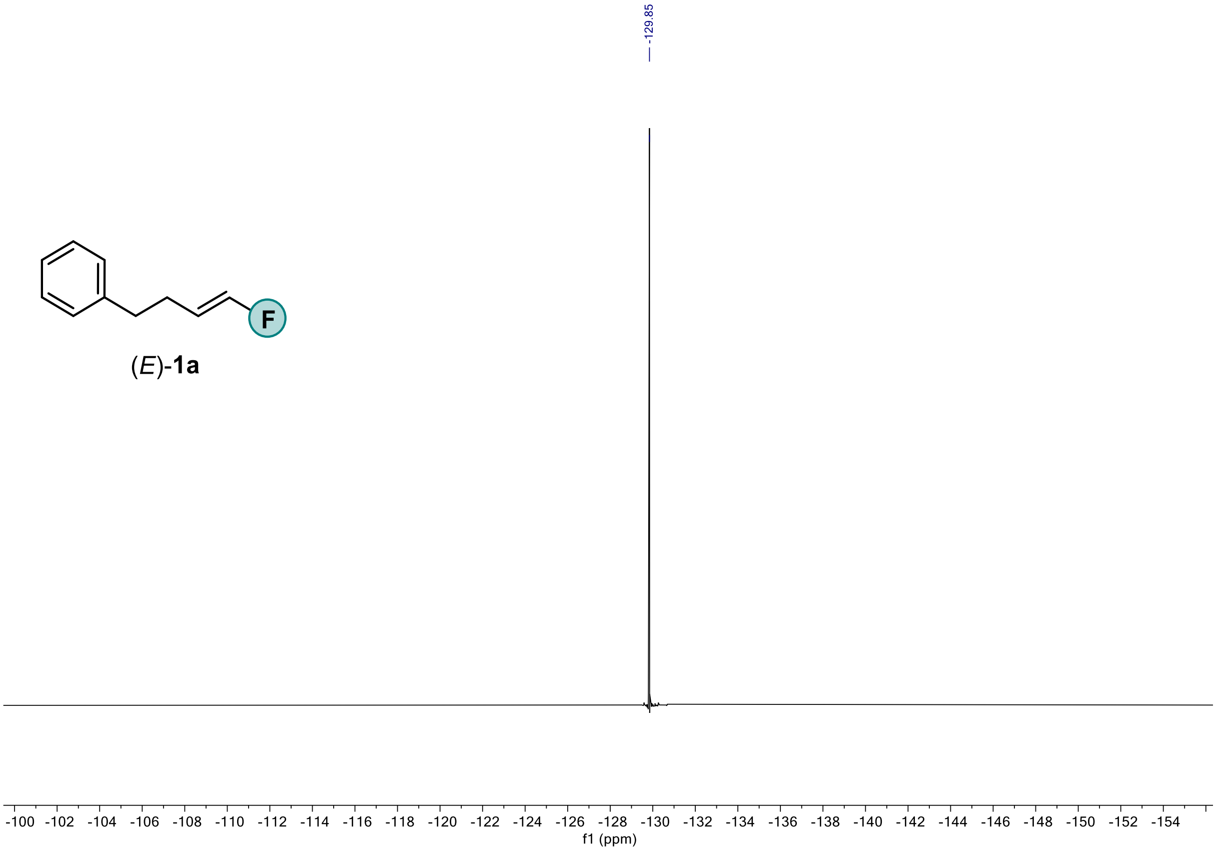
-129.85
(E)-1a
-100 -102 -104 -106 -108 -110 -112 -114 -116 -118 -120 -122 -124 -126 -128 -130 -132 -134 -136 -138 -140 -142 -144 -146 -148 -150 -152 -154
f1 (ppm)

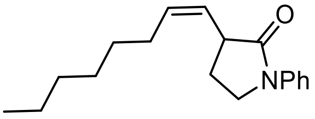
NPh
O

S-12

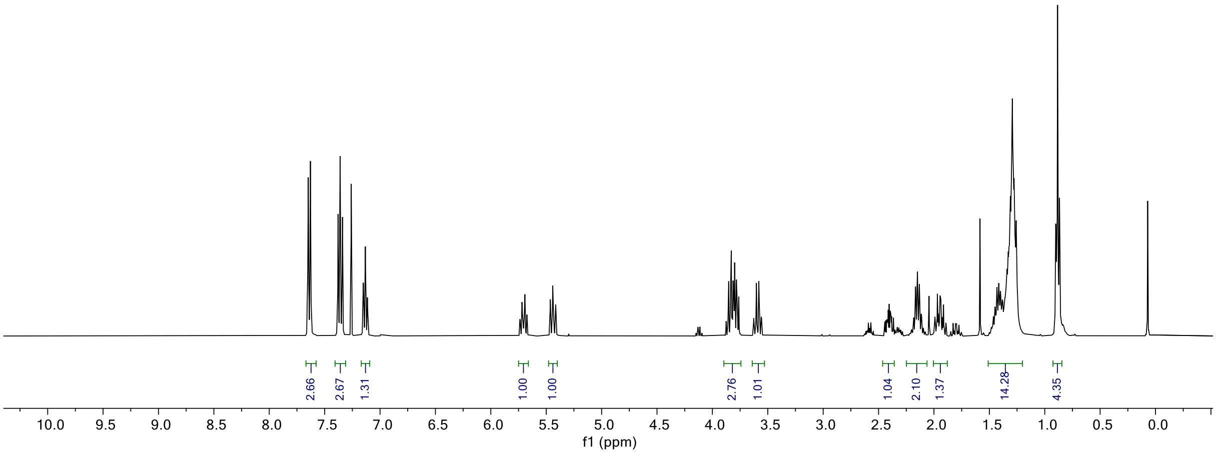
2.66
2.67
1.31
1.00
1.00
2.76
1.01
1.04
2.10
1.37
14.28
4.35
10.0 9.5 9.0 8.5 8.0 7.5 7.0 6.5 6.0 5.5 5.0 4.5 4.0 3.5 3.0 2.5 2.0 1.5 1.0 0.5 0.0
f1 (ppm)

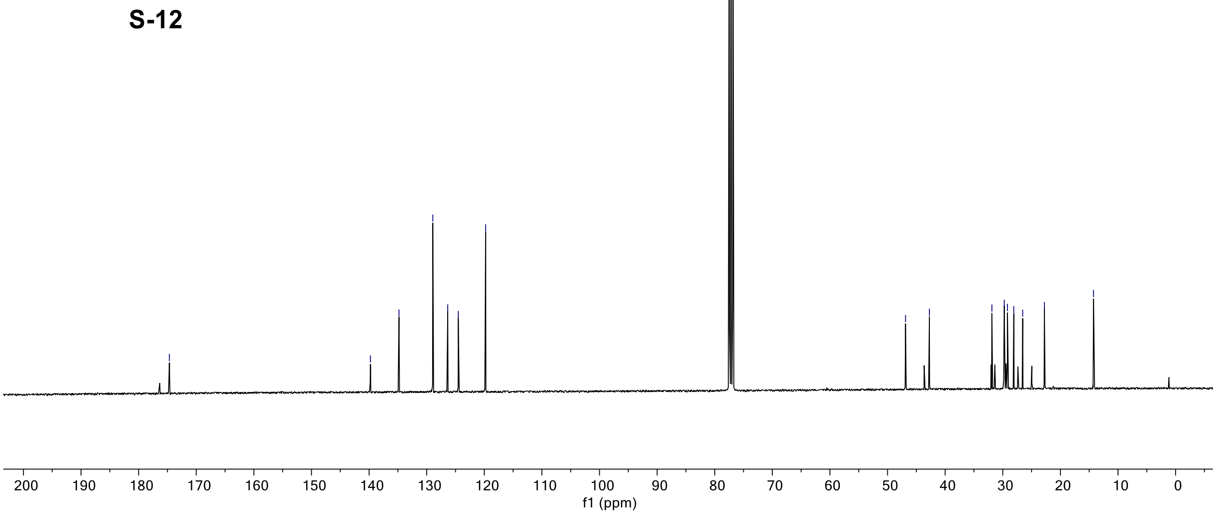
174.67
139.76
134.81
128.94
126.34
124.51
119.77
46.88
42.74
31.88
29.74
29.19
28.09
26.54
22.78
14.24
200 190 180 170 160 150 140 130 120 110 100 90 80 70 60 50 40 30 20 10 0
f1 (ppm)

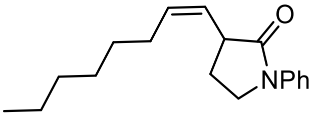
NPh
O

S-12

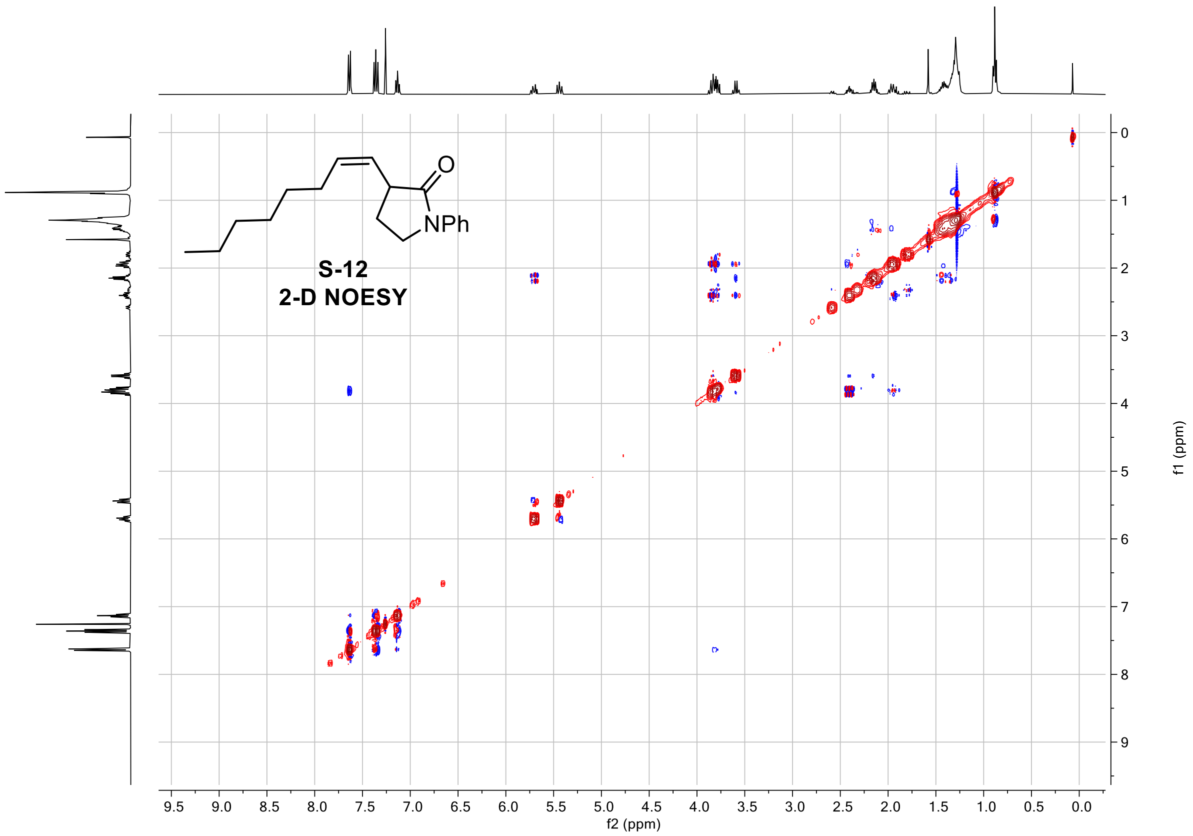

O
NPh
S-12
2-D NOESY
f1 (ppm)
f2 (ppm)

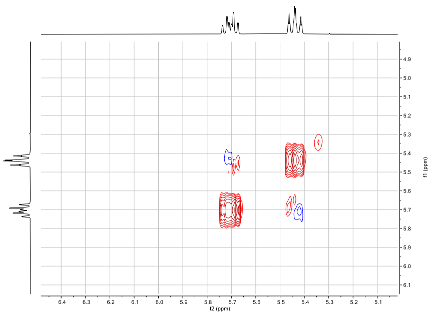

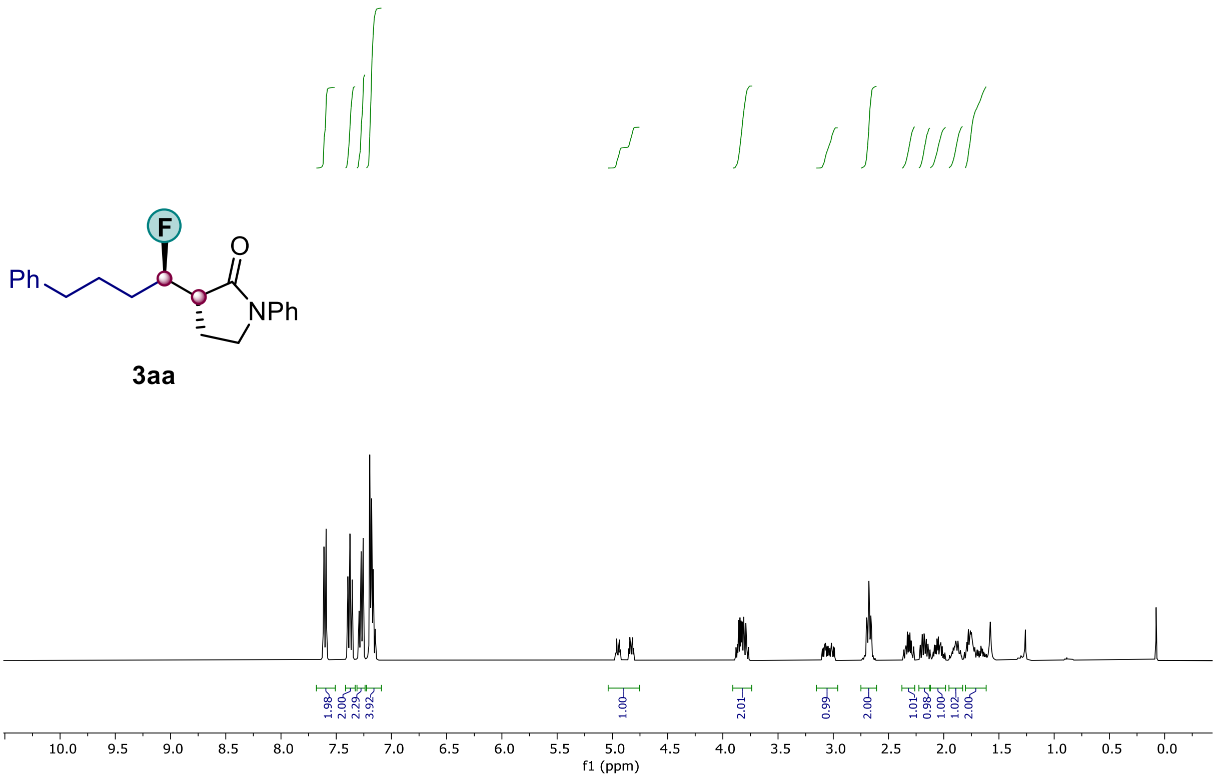

Ph
F
O
NPh
3aa
1.98
2.00
2.29
3.92
1.00
2.01
0.99
2.00
1.01
0.98
1.00
1.02
2.00
10.0
9.5
9.0
8.5
8.0
7.5
7.0
6.5
6.0
5.5
5.0
4.5
4.0
3.5
3.0
2.5
2.0
1.5
1.0
0.5
0.0
f1 (ppm)

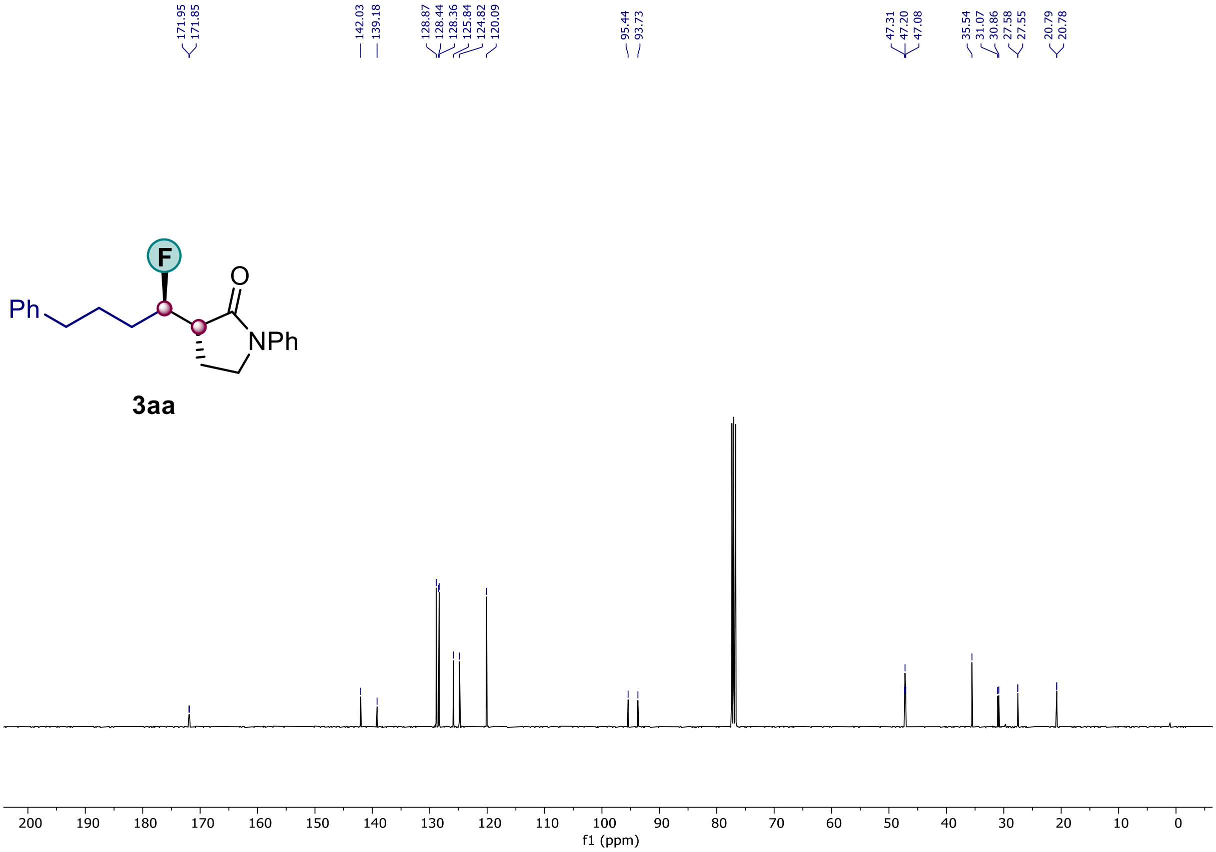

171.95
171.85
142.03
139.18
128.87
128.44
128.36
125.84
124.82
120.09
95.44
93.73
47.31
47.20
47.08
35.54
31.07
30.86
27.58
27.55
20.79
20.78
Ph
F
O
NPh
3aa
200
190
180
170
160
150
140
130
120
110
100
90
80
70
60
50
40
30
20
10
0
f1 (ppm)

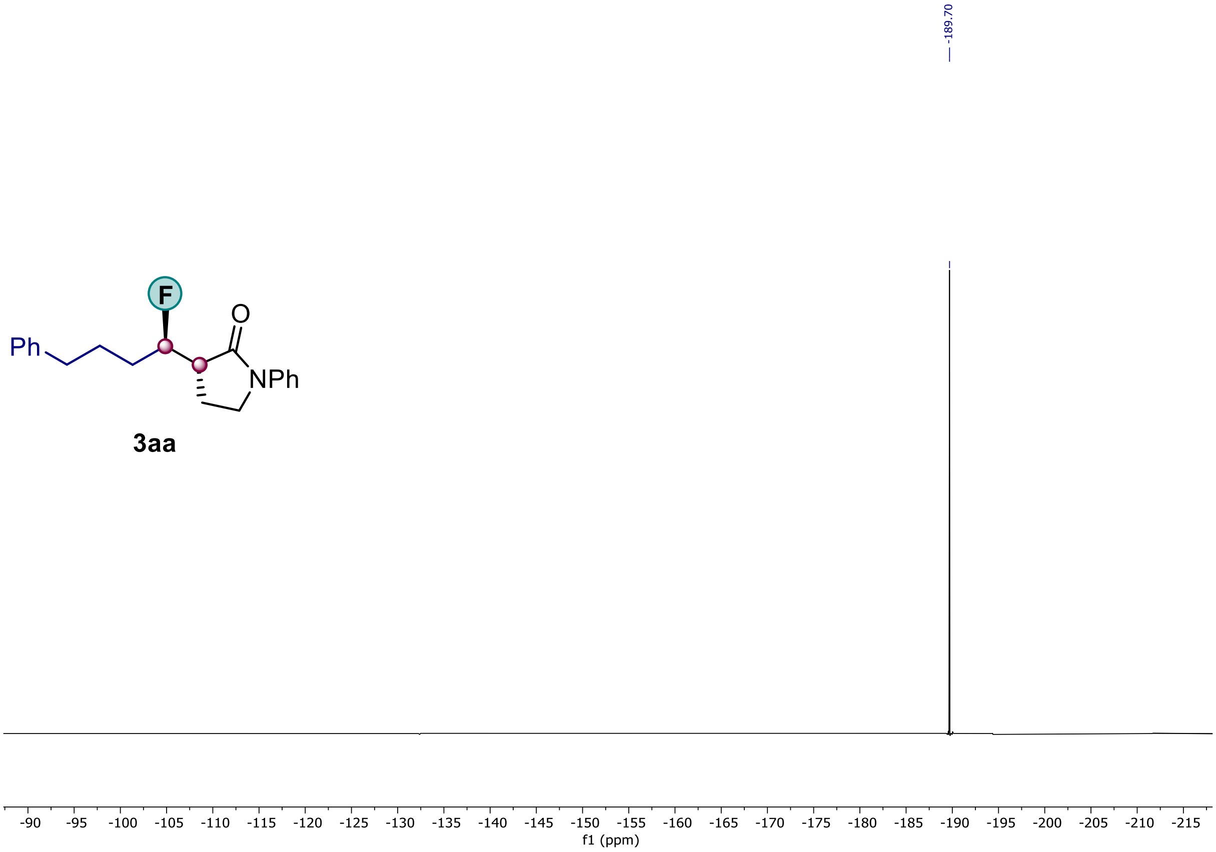
-189.70
-90 -95 -100 -105 -110 -115 -120 -125 -130 -135 -140 -145 -150 -155 -160 -165 -170 -175 -180 -185 -190 -195 -200 -205 -210 -215
3aa
f1 (ppm)

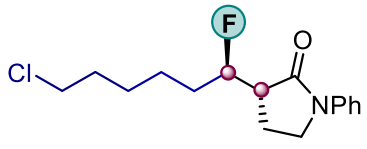

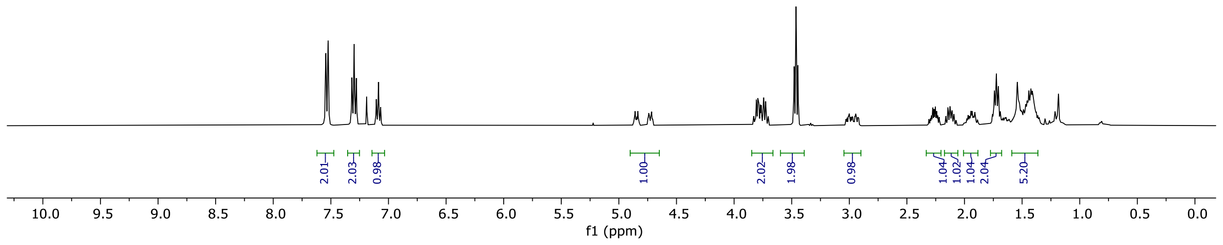

2.01
2.03
0.98
1.00
2.02
1.98
0.98
1.04
1.02
1.04
2.04
5.20
10.0 9.5 9.0 8.5 8.0 7.5 7.0 6.5 6.0 5.5 5.0 4.5 4.0 3.5 3.0 2.5 2.0 1.5 1.0 0.5 0.0
f1 (ppm)

3ba

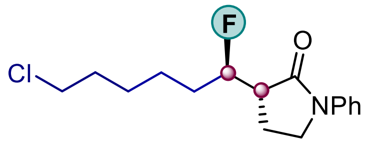

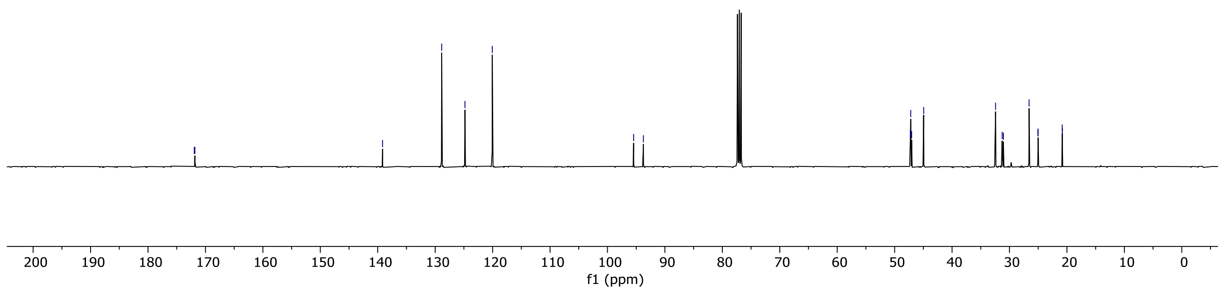

171.94
171.84
139.16
128.87
124.82
120.06
95.45
93.74
47.26
47.20
47.04
44.95
32.42
31.27
31.07
26.59
25.05
25.01
20.82
20.80
200 190 180 170 160 150 140 130 120 110 100 90 80 70 60 50 40 30 20 10 0
f1 (ppm)

3ba

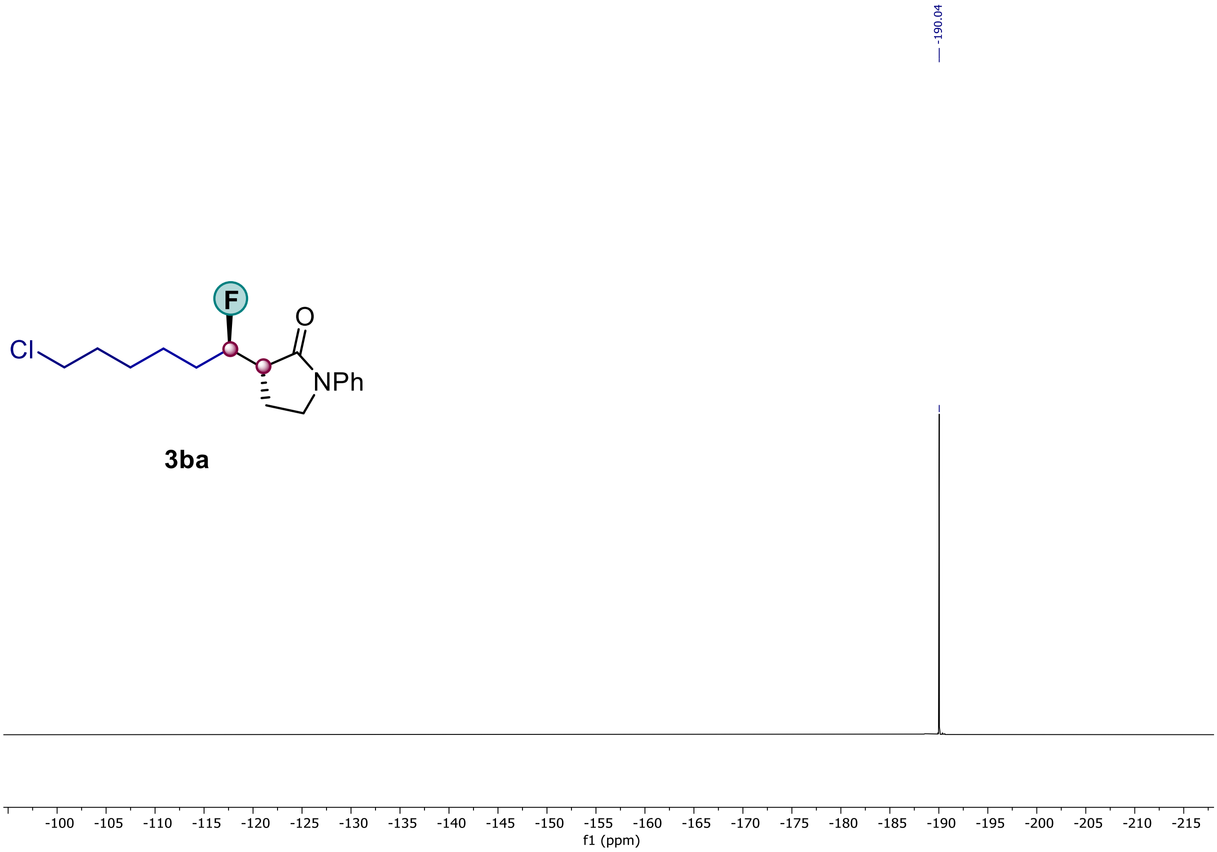
-190.04
Cl
F
O
NPh
3ba
-100
-105
-110
-115
-120
-125
-130
-135
-140
-145
-150
-155
-160
-165
-170
-175
-180
-185
-190
-195
-200
-205
-210
-215
f1 (ppm)

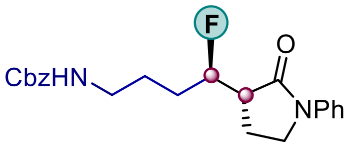
CbzHN
F
O
NPh

3ca

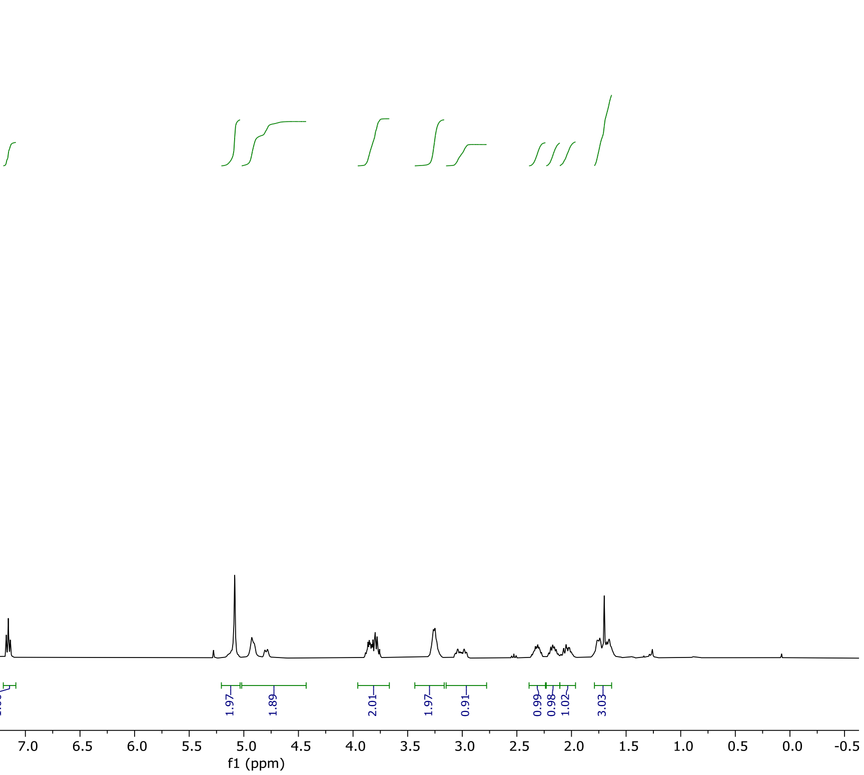
1.95
6.82
1.00
1.97
1.89
2.01
1.97
0.91
0.99
0.98
1.02
3.03
10.0 9.5 9.0 8.5 8.0 7.5 7.0 6.5 6.0 5.5 5.0 4.5 4.0 3.5 3.0 2.5 2.0 1.5 1.0 0.5 0.0 -0.5
f1 (ppm)

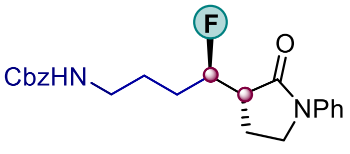
CbzHN
F
O
NPh

3ca

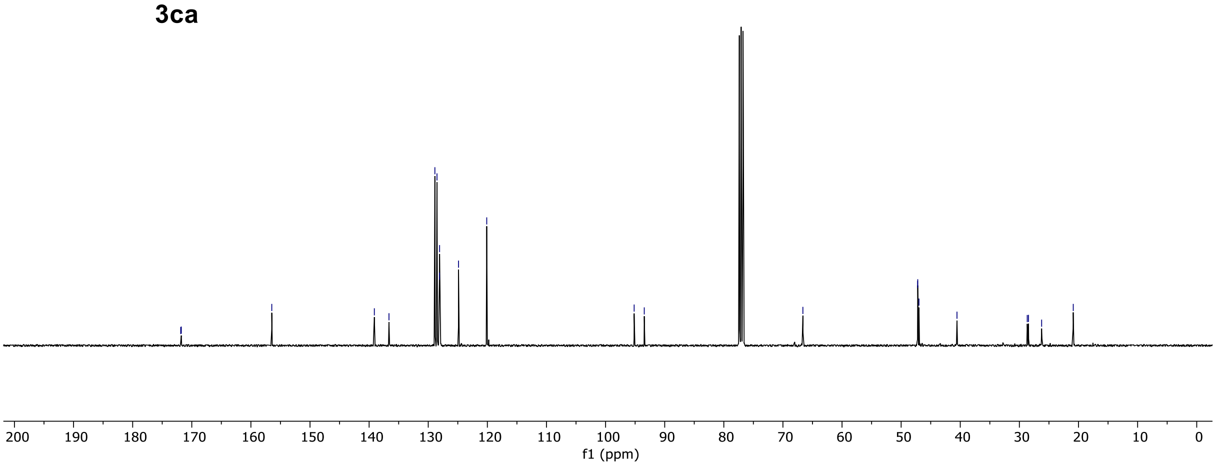
171.87
171.78
156.47
139.12
136.64
128.88
128.52
128.10
128.08
124.87
120.11
95.17
93.46
66.62
47.21
47.19
46.99
40.56
28.67
28.46
26.23
20.89
200 190 180 170 160 150 140 130 120 110 100 90 80 70 60 50 40 30 20 10 0
f1 (ppm)

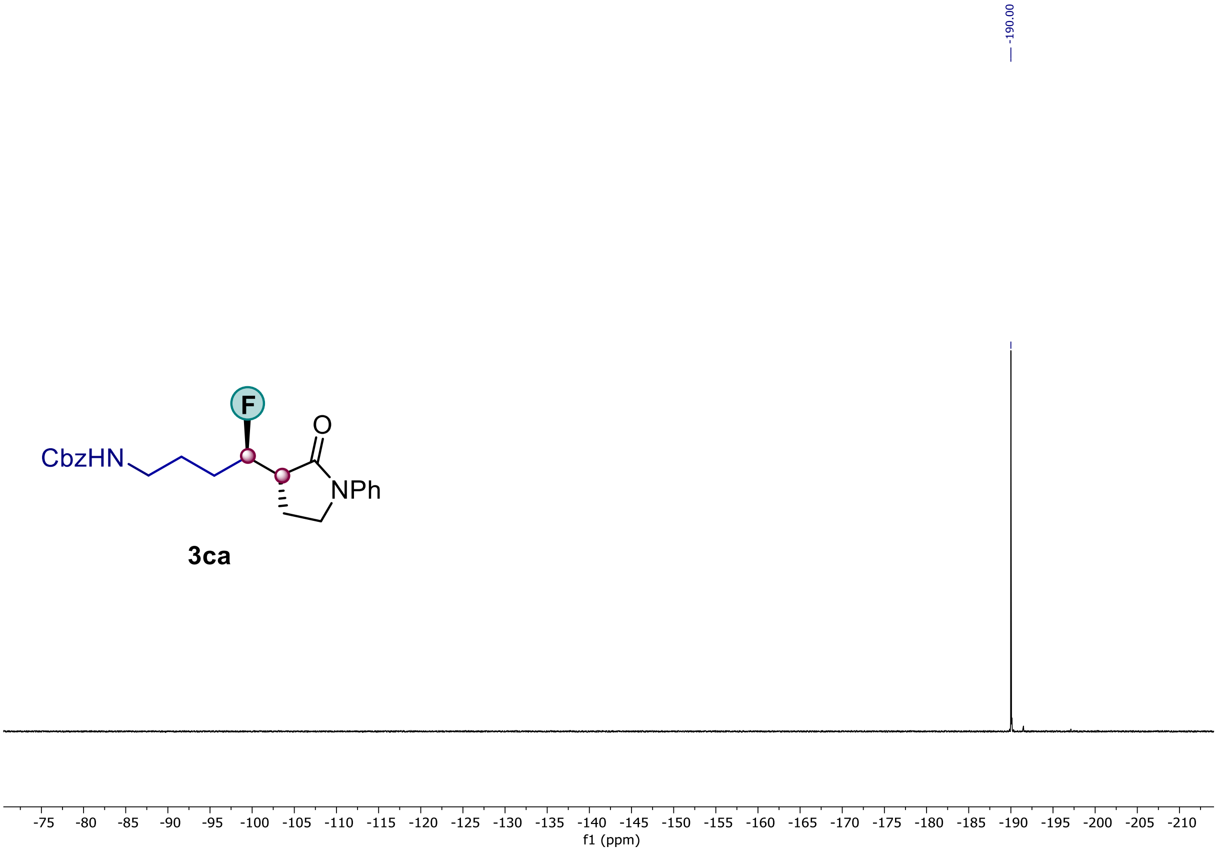
-190.00
F
O
CbzHN
NPh
3ca
-75 -80 -85 -90 -95 -100 -105 -110 -115 -120 -125 -130 -135 -140 -145 -150 -155 -160 -165 -170 -175 -180 -185 -190 -195 -200 -205 -210
f1 (ppm)

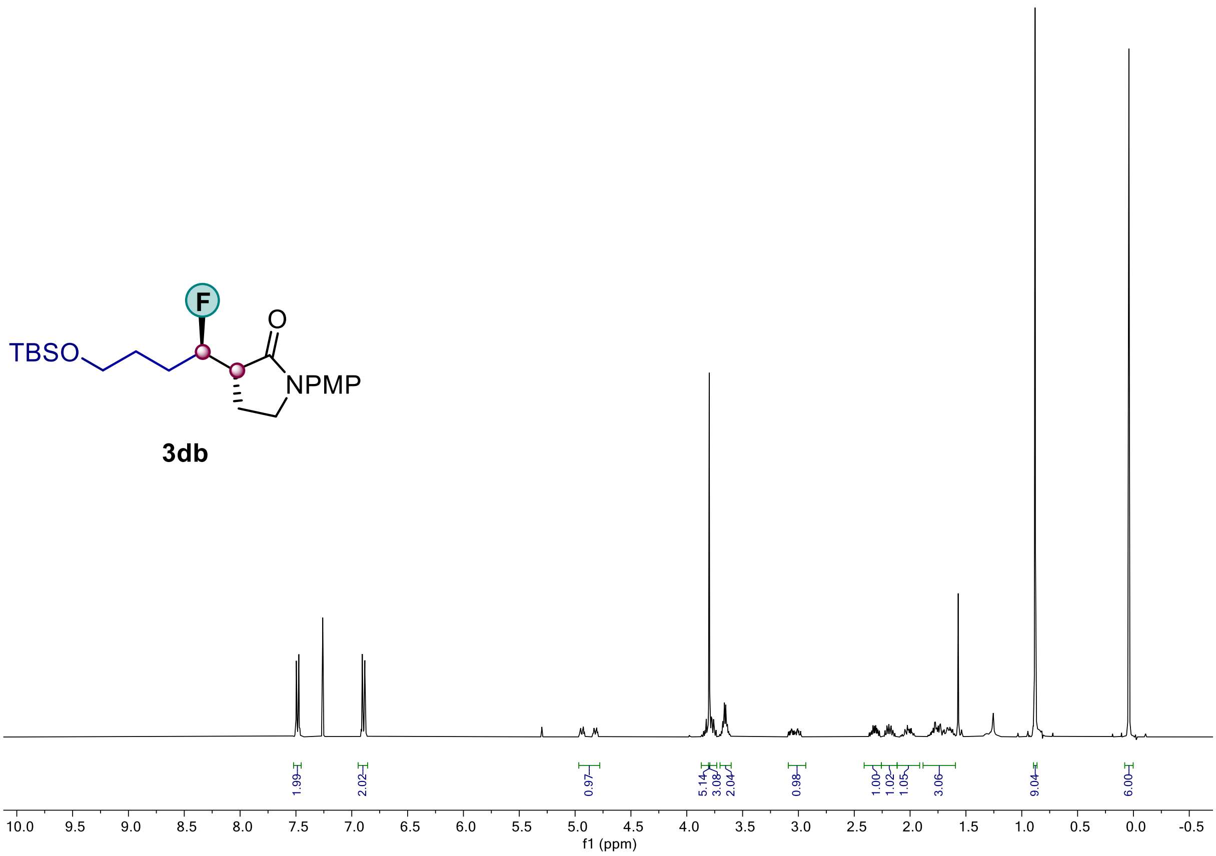

F
O
TBSO
NPMP
3db
1.99
2.02
0.97
5.14
3.08
2.04
0.98
1.00
1.02
1.05
3.06
9.04
6.00
10.0
9.5
9.0
8.5
8.0
7.5
7.0
6.5
6.0
5.5
5.0
4.5
4.0
3.5
3.0
2.5
2.0
1.5
1.0
0.5
0.0
-0.5
f1 (ppm)

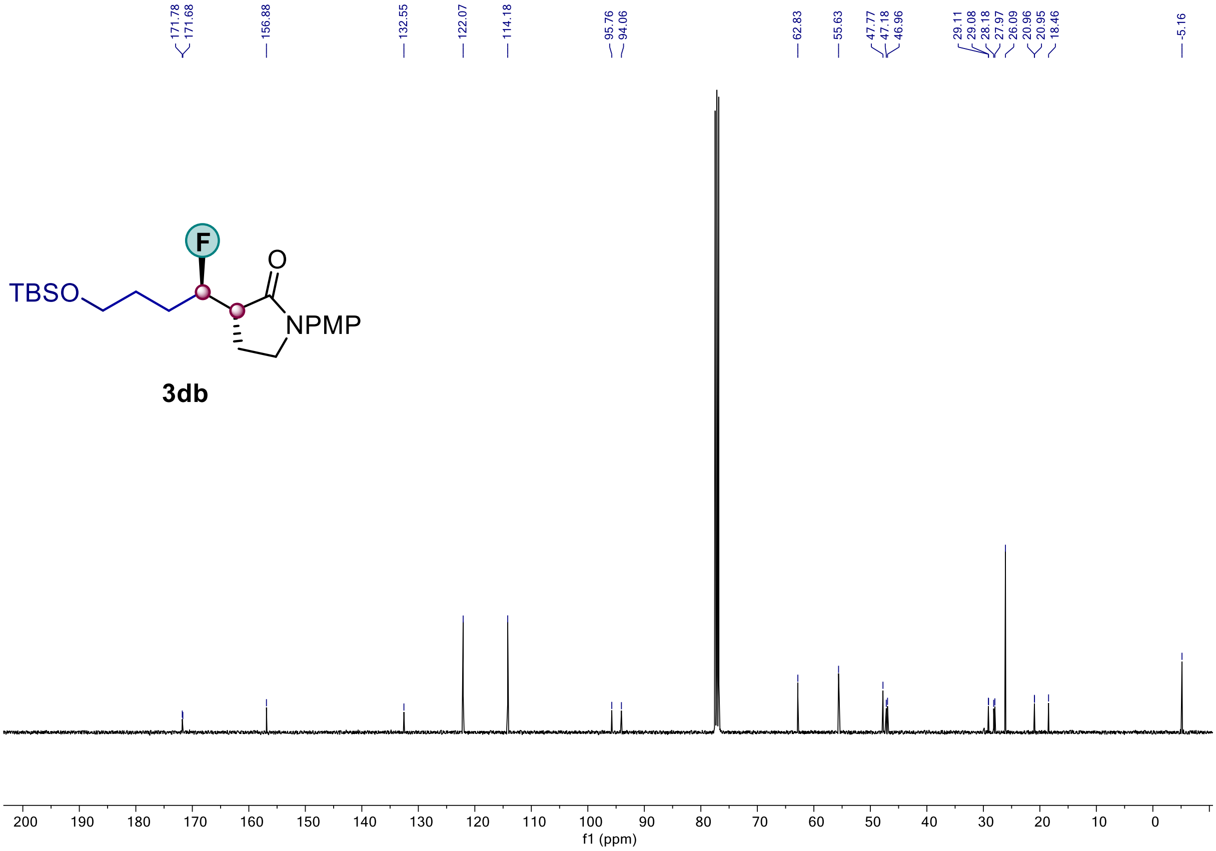

F
O
TBSO
NPMP
3db
171.78
171.68
156.88
132.55
122.07
114.18
95.76
94.06
62.83
55.63
47.77
47.18
46.96
29.11
29.08
28.18
27.97
26.09
20.96
20.95
18.46
-5.16
200
190
180
170
160
150
140
130
120
110
100
90
80
70
60
50
40
30
20
10
0
f1 (ppm)

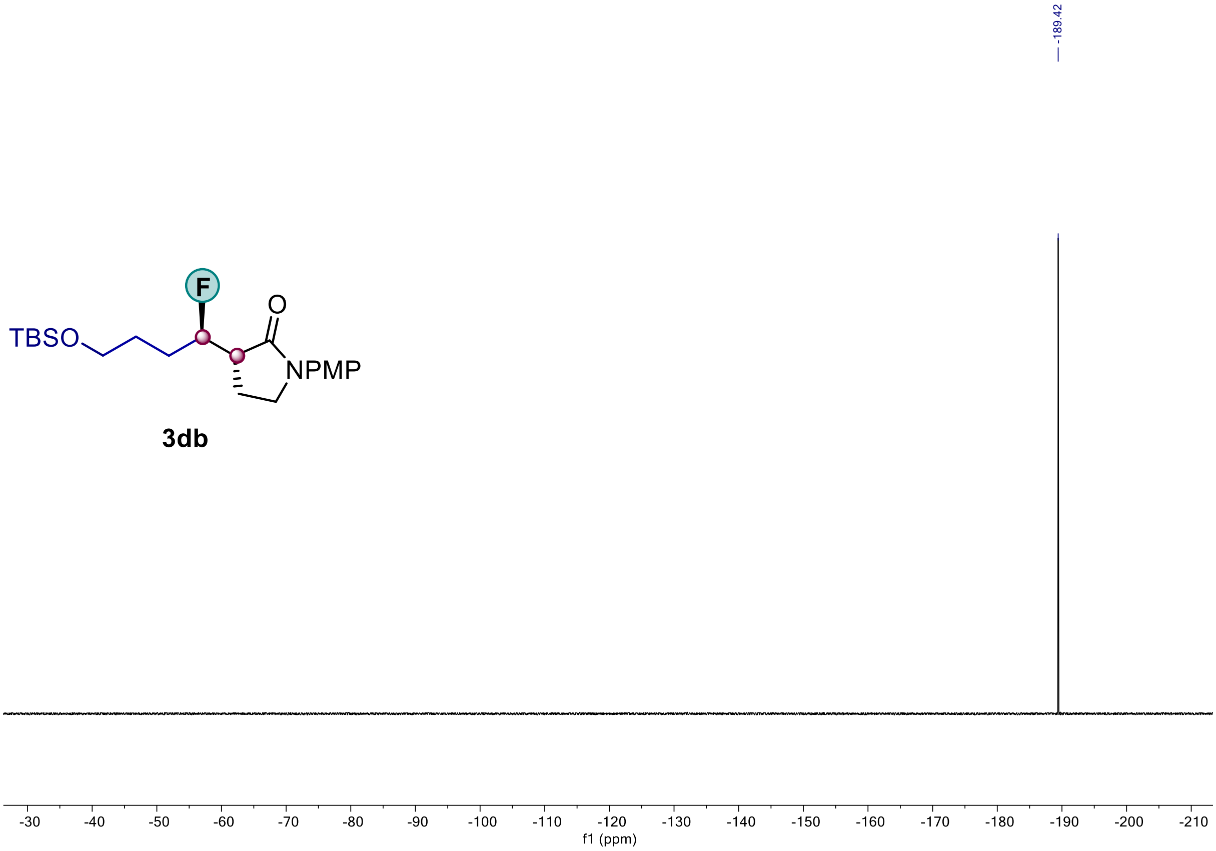

-189.42
TBSO
F
O
NPMP
-30
-40
-50
-60
-70
-80
-90
-100
-110
-120
-130
-140
-150
-160
-170
-180
-190
-200
-210
f1 (ppm)

3db

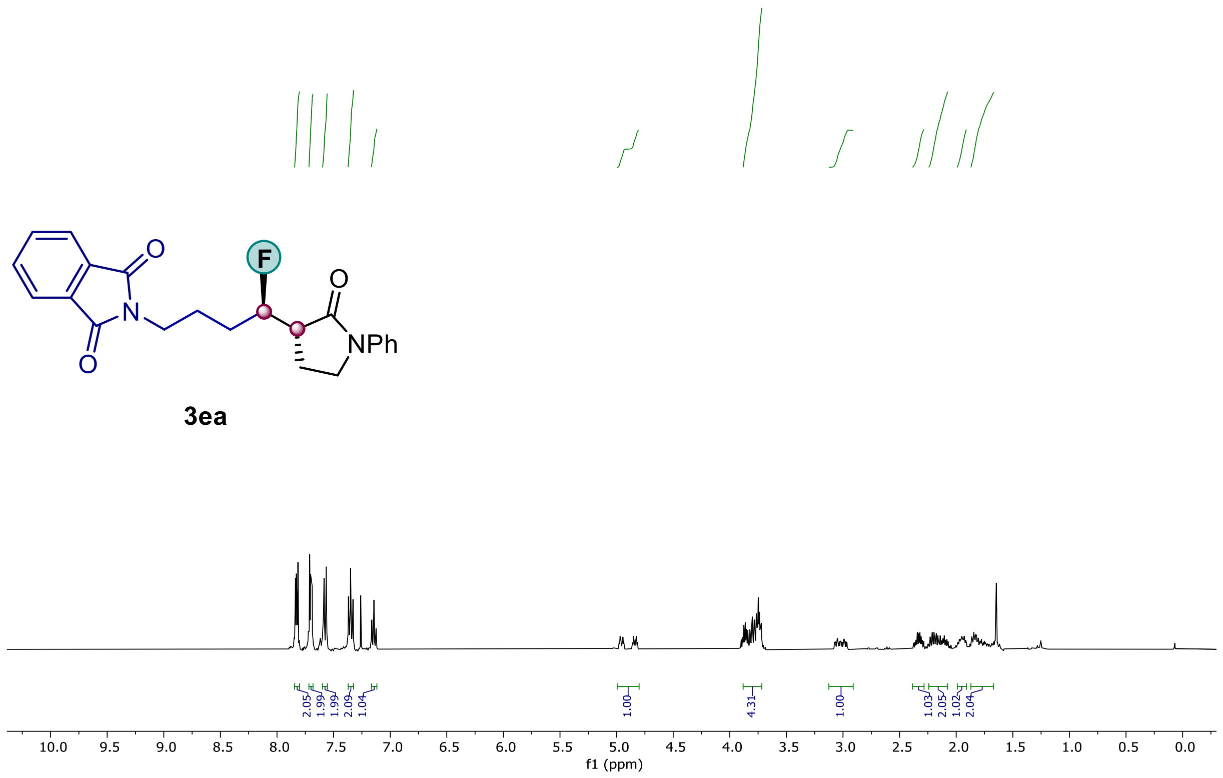
3ea
NPh
2.05
1.99
1.99
2.09
1.04
1.00
4.31
1.00
1.03
2.05
1.02
2.04
10.0 9.5 9.0 8.5 8.0 7.5 7.0 6.5 6.0 5.5 5.0 4.5 4.0 3.5 3.0 2.5 2.0 1.5 1.0 0.5 0.0
f1 (ppm)

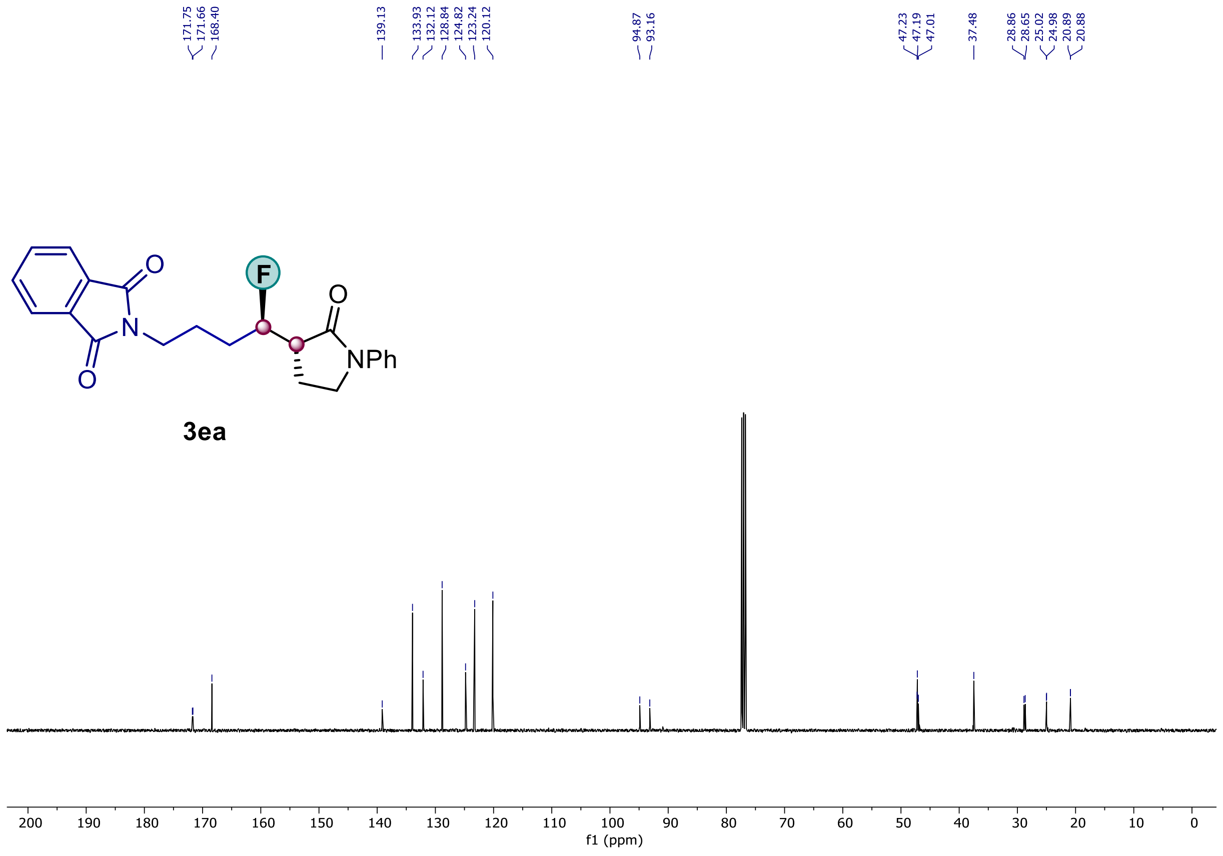
171.75
171.66
168.40
139.13
133.93
132.12
128.84
124.82
123.24
120.12
94.87
93.16
47.23
47.19
47.01
37.48
28.86
28.65
25.02
24.98
20.89
20.88
3ea
NPh
200 190 180 170 160 150 140 130 120 110 100 90 80 70 60 50 40 30 20 10 0
f1 (ppm)

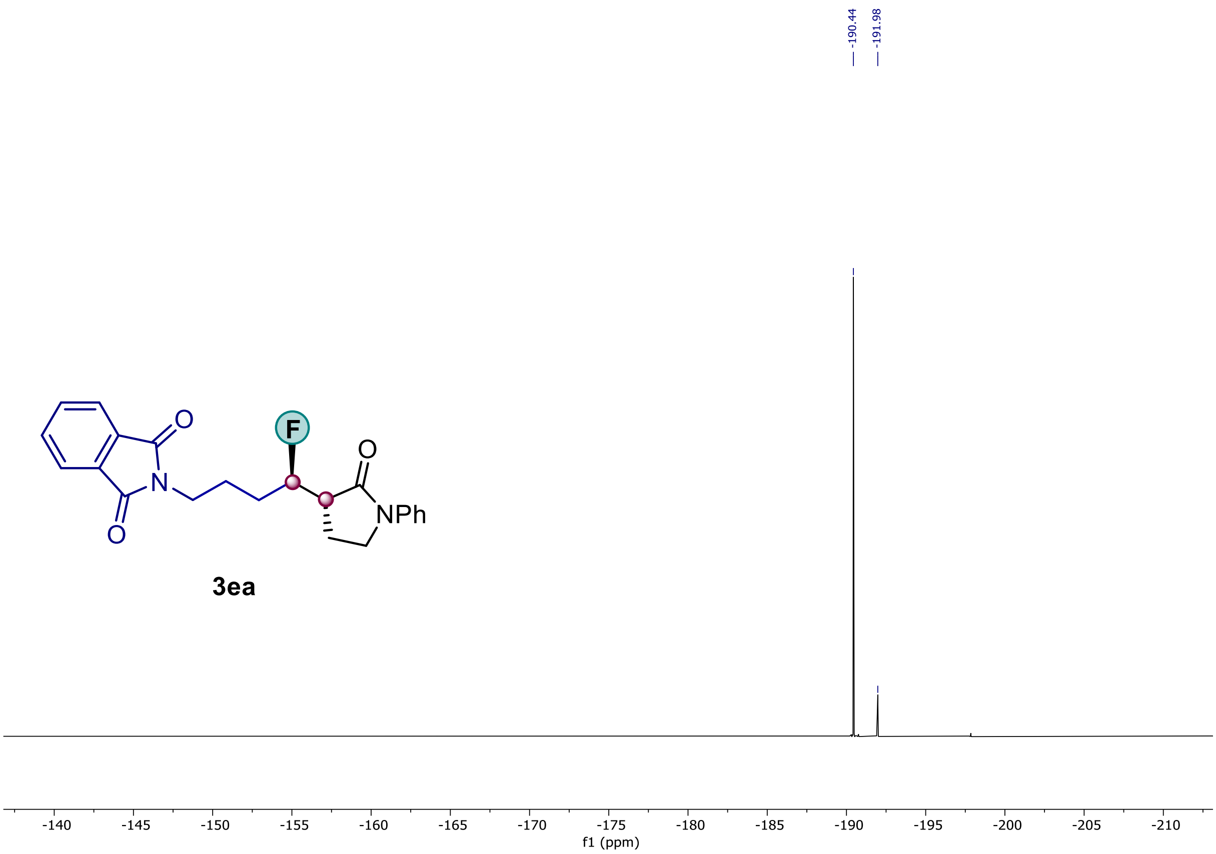

-190.44
-191.98
O
N
O
F
O
NPh
3ea
-140
-145
-150
-155
-160
-165
-170
-175
-180
-185
-190
-195
-200
-205
-210
f1 (ppm)

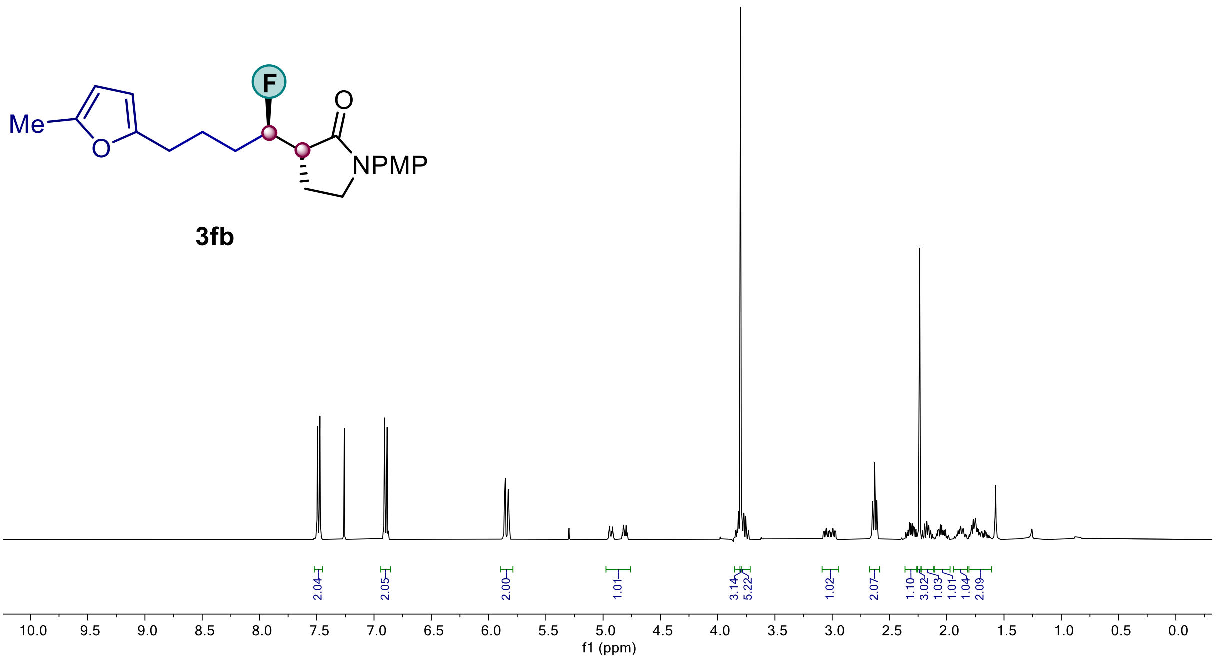
Me
O
F
O
NPMP
3fb
2.04
2.05
2.00
1.01
3.14
5.22
1.02
2.07
1.10
3.02
1.03
1.01
1.04
2.09
10.0 9.5 9.0 8.5 8.0 7.5 7.0 6.5 6.0 5.5 5.0 4.5 4.0 3.5 3.0 2.5 2.0 1.5 1.0 0.5 0.0
f1 (ppm)

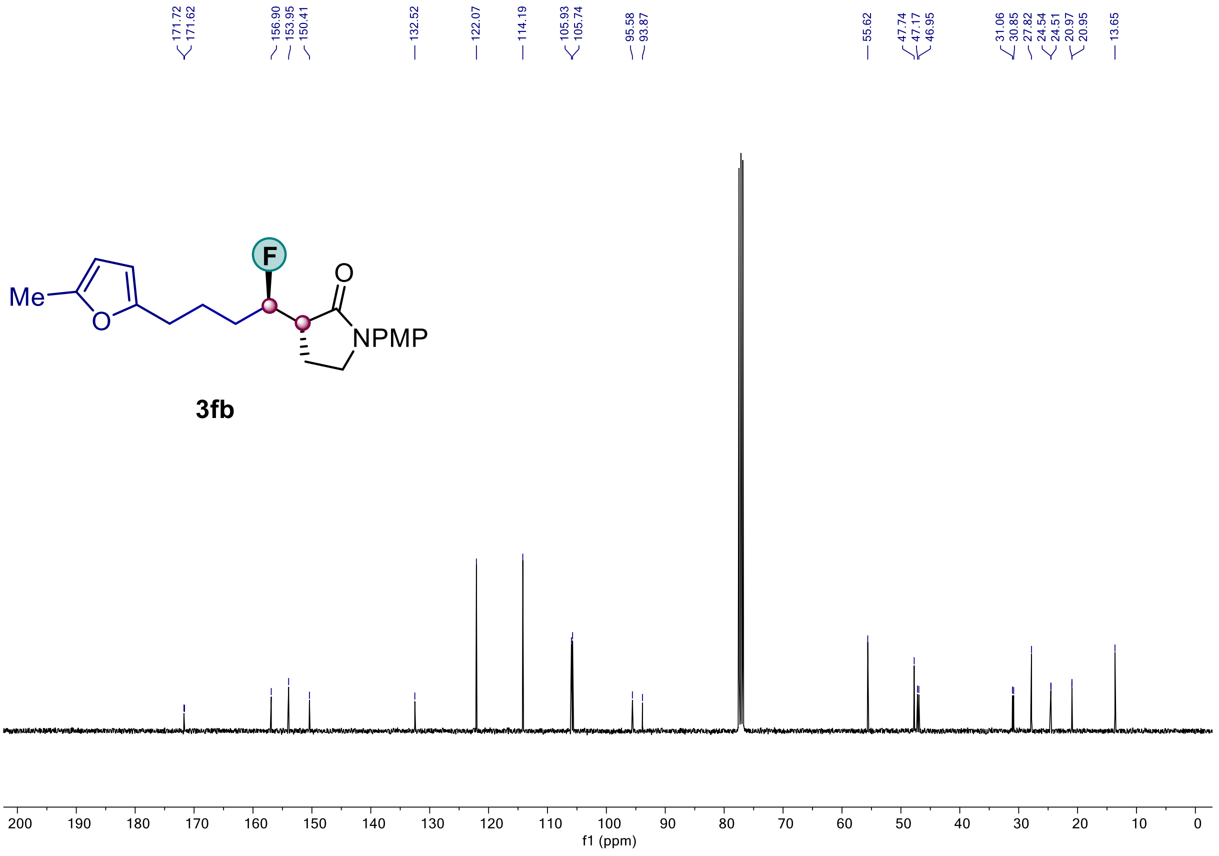
171.72
171.62
156.90
153.95
150.41
132.52
122.07
114.19
105.93
105.74
95.58
93.87
55.62
47.74
47.17
46.95
31.06
30.85
27.82
24.54
24.51
20.97
20.95
13.65
Me
O
F
O
NPMP
3fb
200 190 180 170 160 150 140 130 120 110 100 90 80 70 60 50 40 30 20 10 0
f1 (ppm)

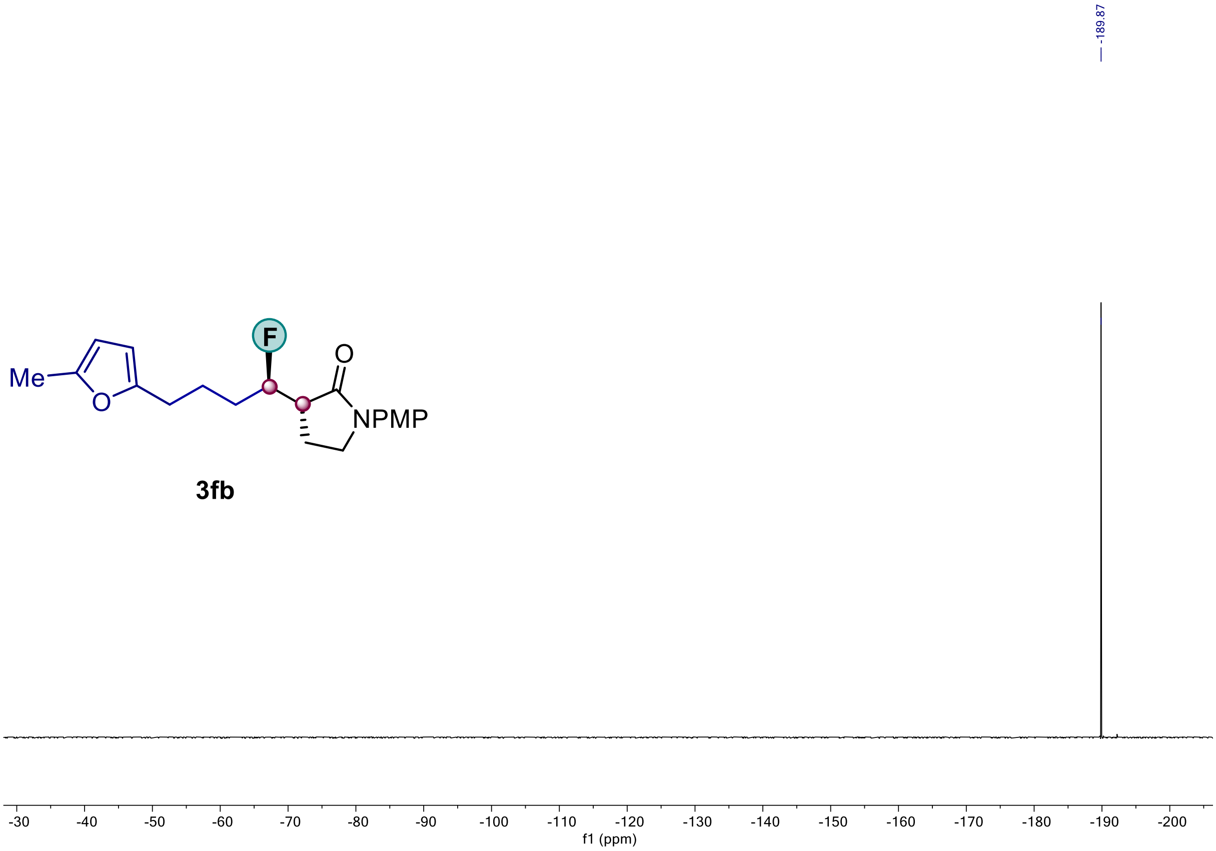
-189.87
Me
O
F
O
NPMP
-30
-40
-50
-60
-70
-80
-90
-100
-110
-120
-130
-140
-150
-160
-170
-180
-190
-200
f1 (ppm)

3fb

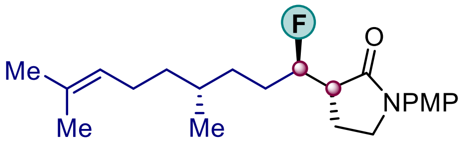

3gb

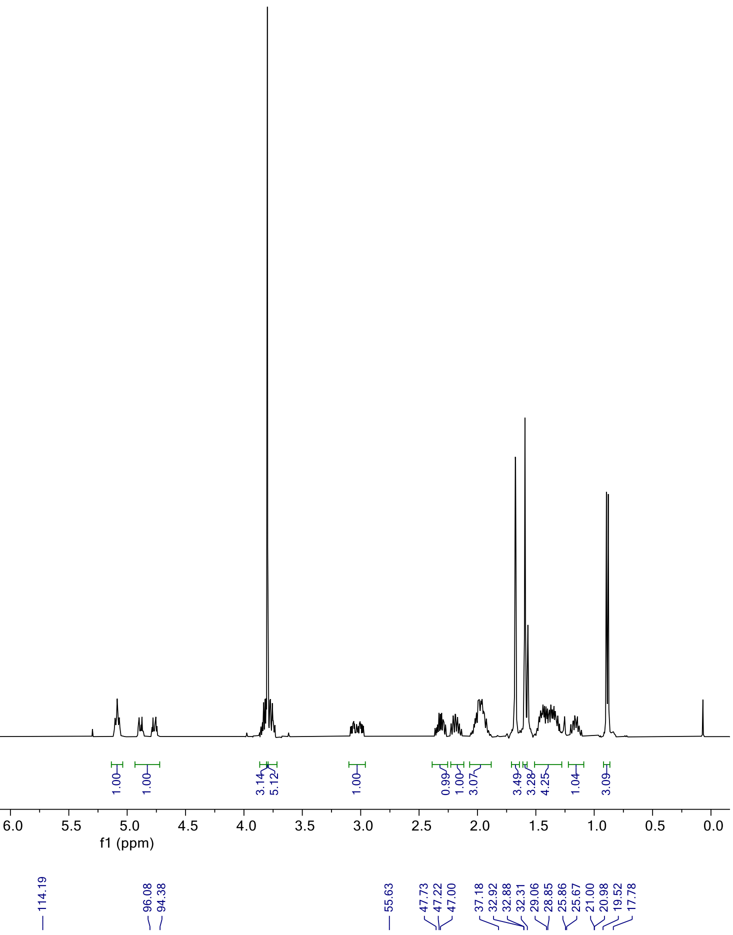

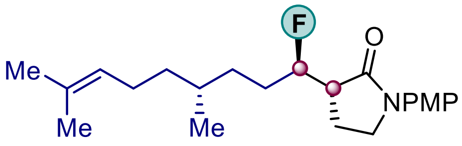

3gb

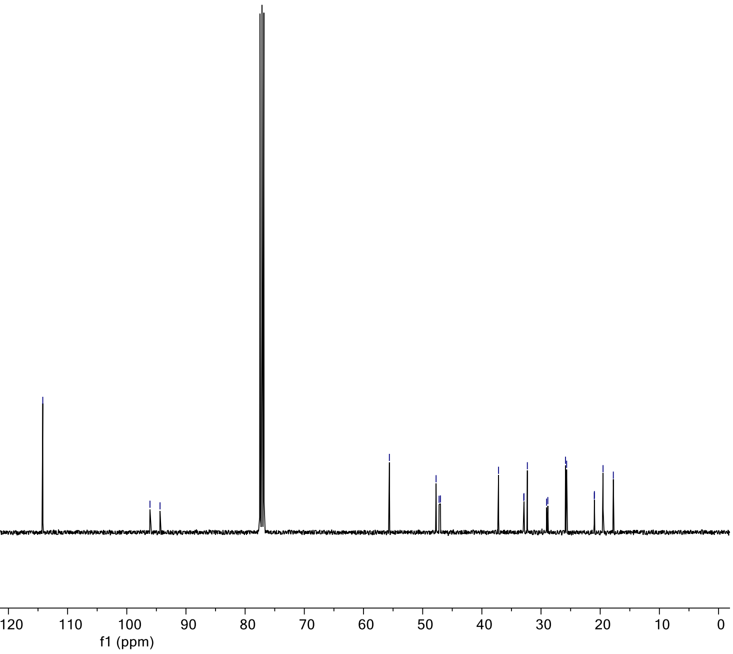

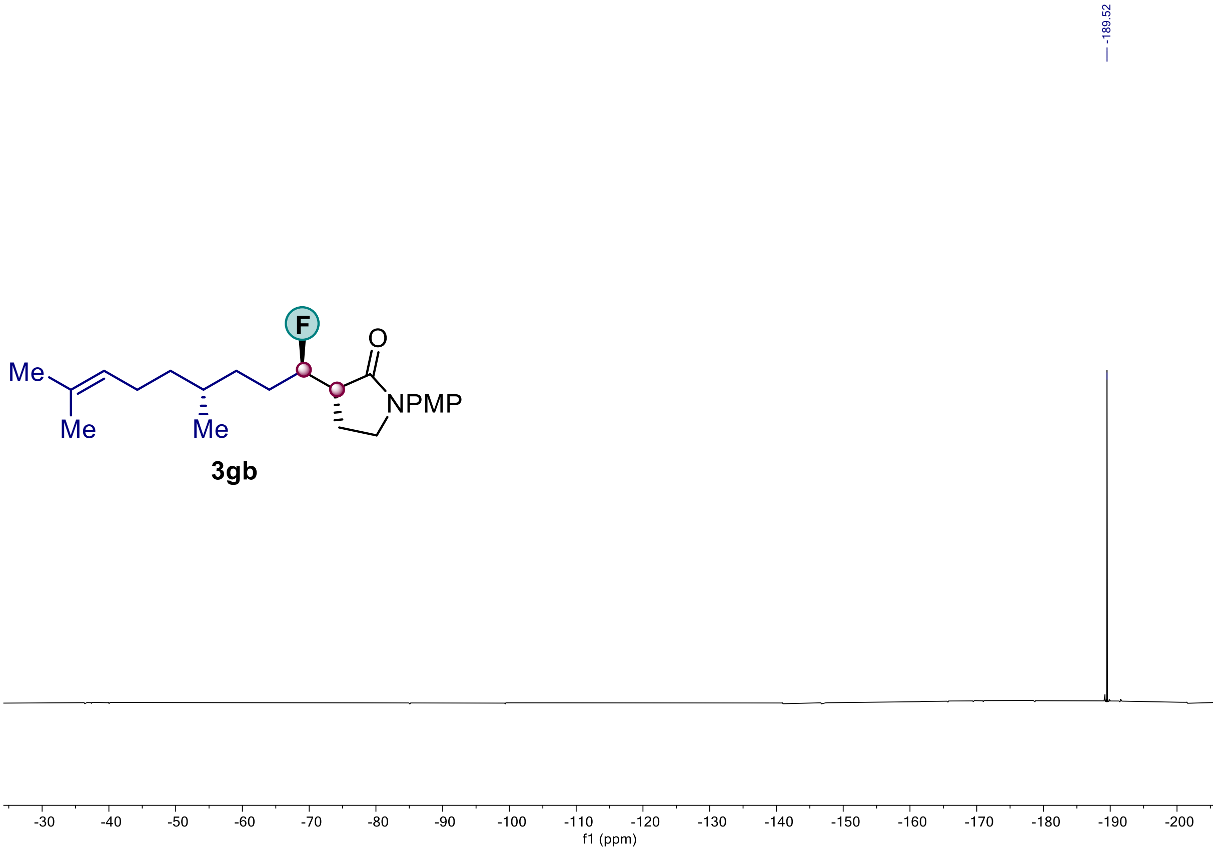
-189.52
Me
Me
Me
F
O
NPMP
3gb
-30
-40
-50
-60
-70
-80
-90
-100
-110
-120
-130
-140
-150
-160
-170
-180
-190
-200
f1 (ppm)

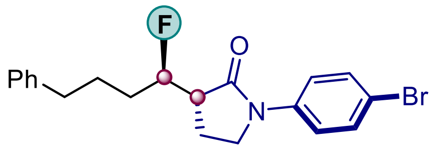
Ph
F
O
N
Br

3ac

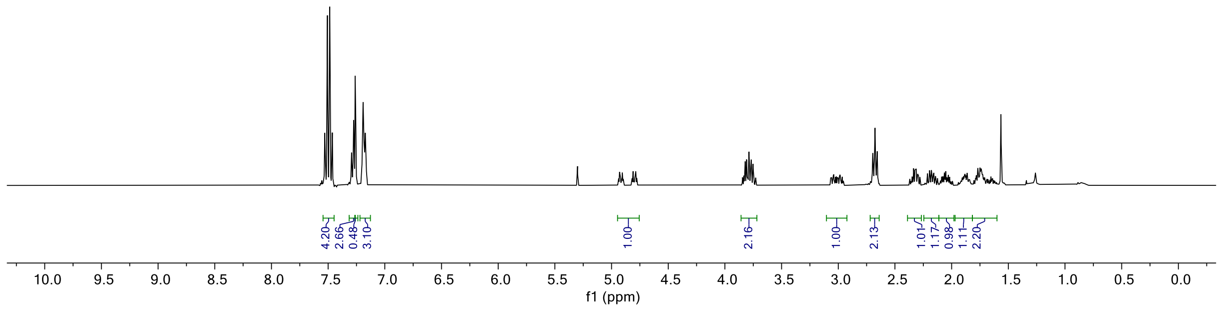
4.20
2.66
0.48
3.10
1.00
2.16
1.00
2.13
1.01
1.17
0.98
1.11
2.20
10.0 9.5 9.0 8.5 8.0 7.5 7.0 6.5 6.0 5.5 5.0 4.5 4.0 3.5 3.0 2.5 2.0 1.5 1.0 0.5 0.0
f1 (ppm)

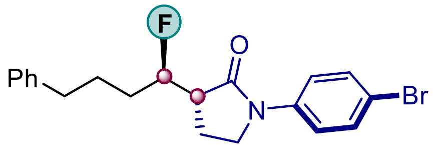
Ph
F
O
N
Br

3ac

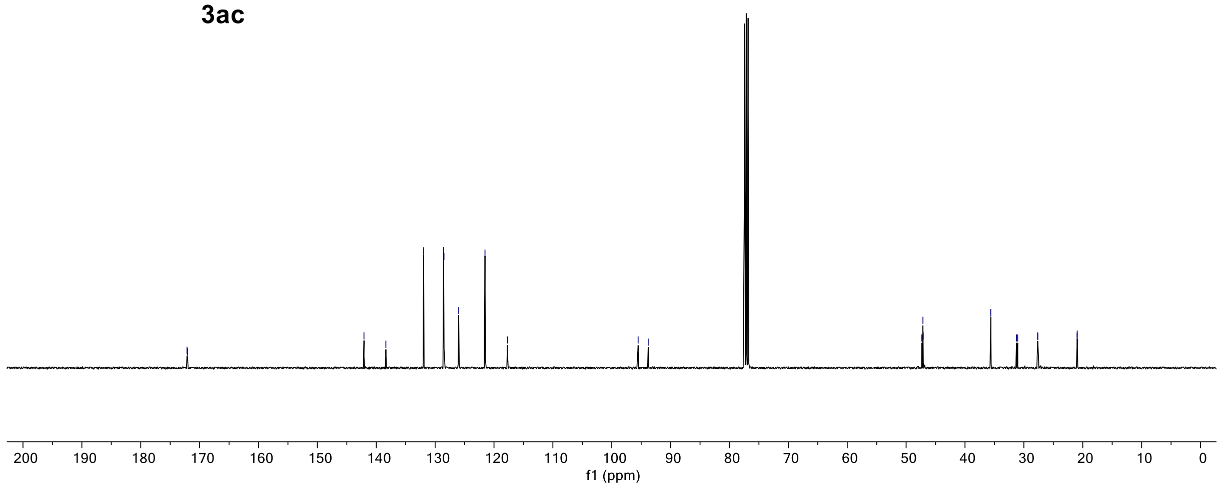
172.14
172.05
142.08
138.36
131.94
128.55
128.50
125.99
121.53
121.46
117.72
95.52
93.81
47.33
47.14
47.10
35.62
31.27
31.05
27.67
27.63
20.96
20.94
200 190 180 170 160 150 140 130 120 110 100 90 80 70 60 50 40 30 20 10 0
f1 (ppm)

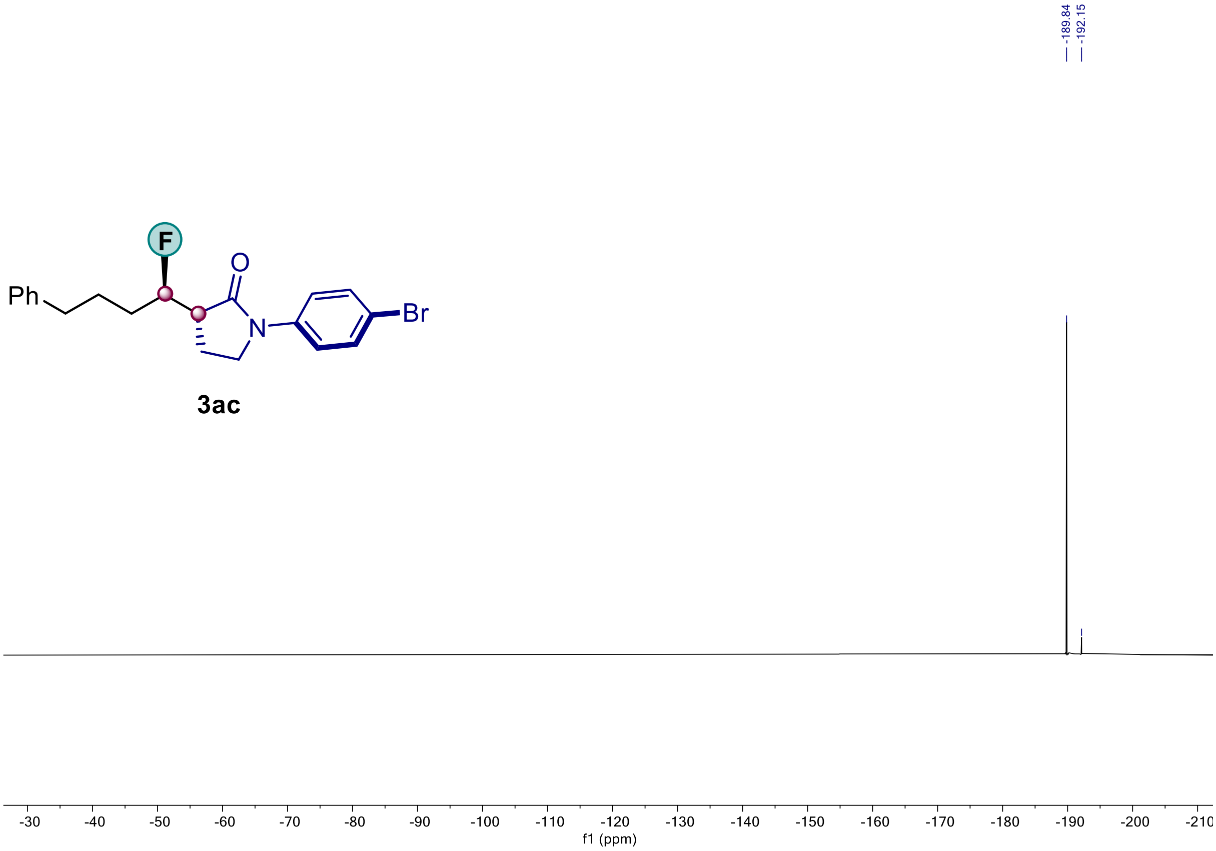

F
O
Ph
N
Br
-189.84
-192.15
-30
-40
-50
-60
-70
-80
-90
-100
-110
-120
-130
-140
-150
-160
-170
-180
-190
-200
-210
f1 (ppm)

3ac

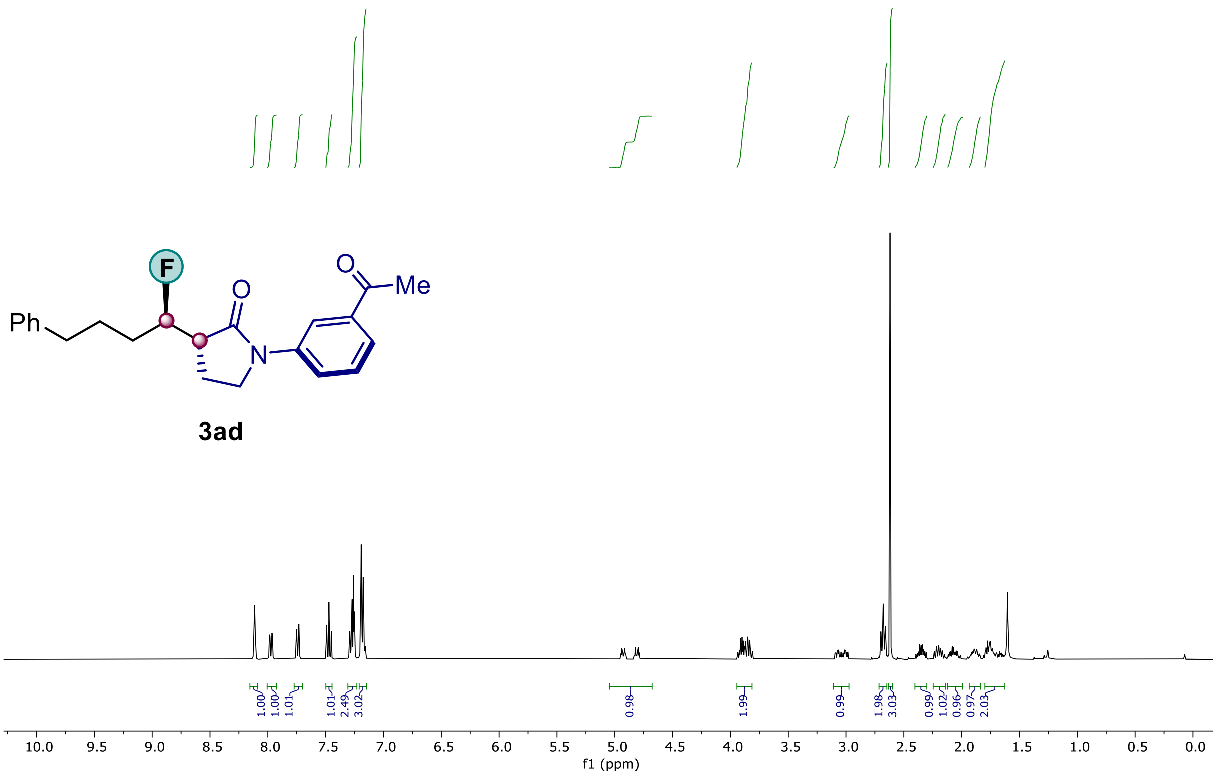

Ph
F
O
N
O
Me
3ad
1.00
1.00
1.01
1.01
2.49
3.02
0.98
1.99
0.99
1.98
3.03
0.99
1.02
0.96
0.97
2.03
10.0 9.5 9.0 8.5 8.0 7.5 7.0 6.5 6.0 5.5 5.0 4.5 4.0 3.5 3.0 2.5 2.0 1.5 1.0 0.5 0.0
f1 (ppm)

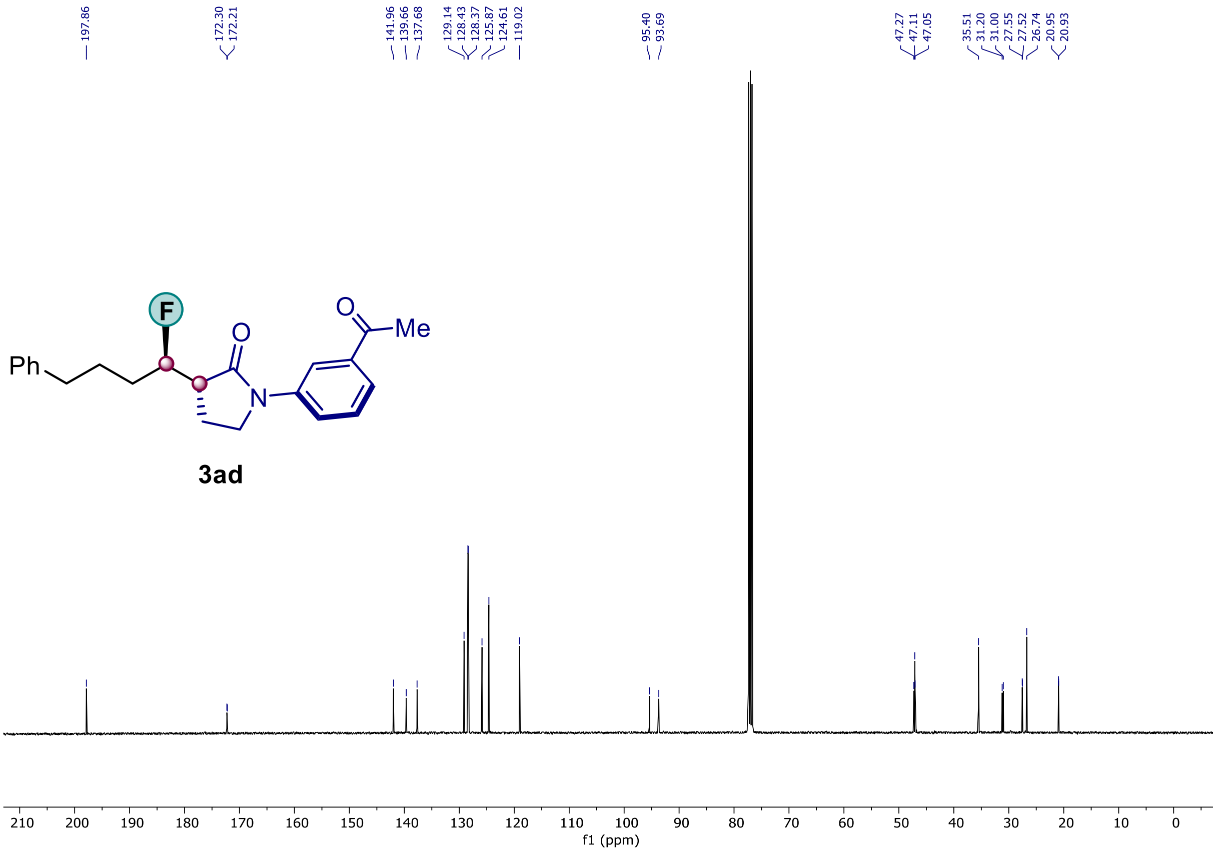

197.86
172.30
172.21
141.96
139.66
137.68
129.14
128.43
128.37
125.87
124.61
119.02
95.40
93.69
47.27
47.11
47.05
35.51
31.20
31.00
27.55
27.52
26.74
20.95
20.93
Ph
F
O
N
O
Me
3ad
210 200 190 180 170 160 150 140 130 120 110 100 90 80 70 60 50 40 30 20 10 0
f1 (ppm)

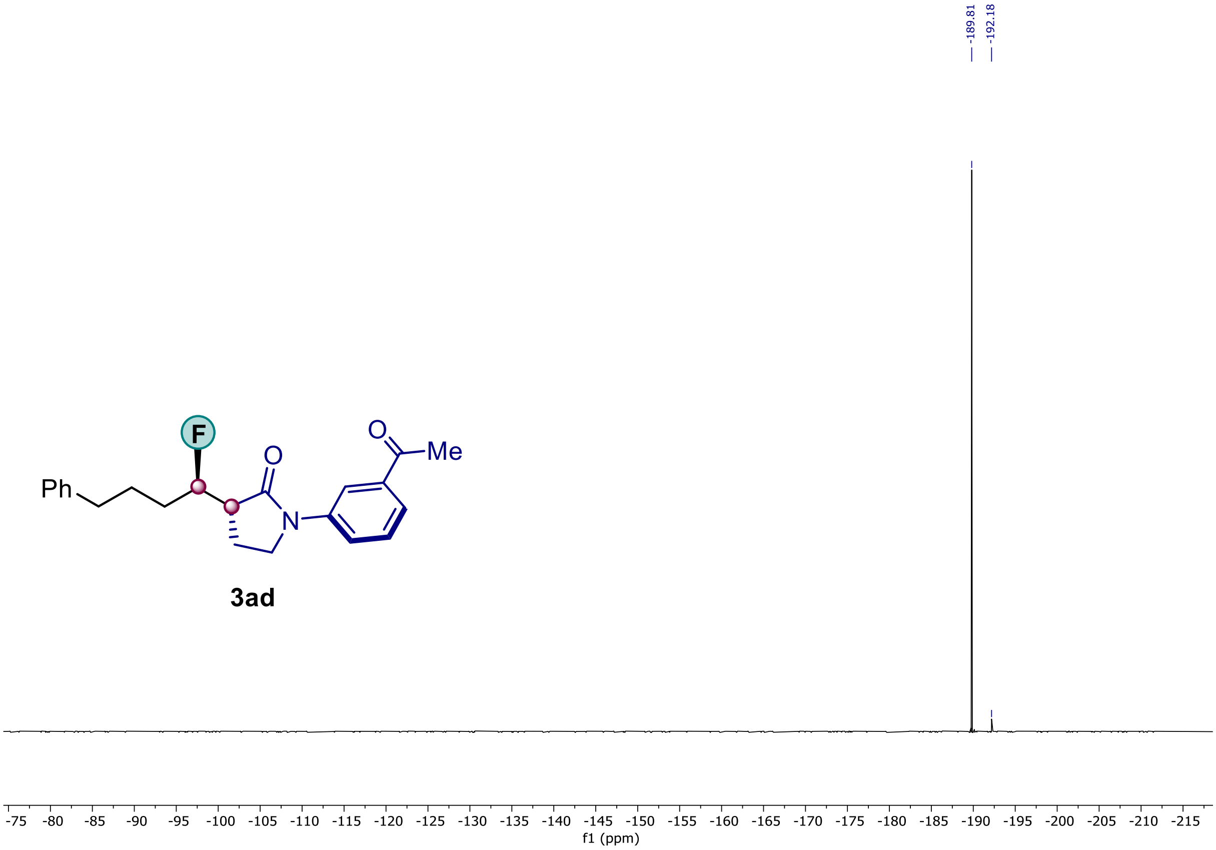

-189.81
-192.18
Ph
F
O
O
Me
N
3ad
-75 -80 -85 -90 -95 -100 -105 -110 -115 -120 -125 -130 -135 -140 -145 -150 -155 -160 -165 -170 -175 -180 -185 -190 -195 -200 -205 -210 -215
f1 (ppm)

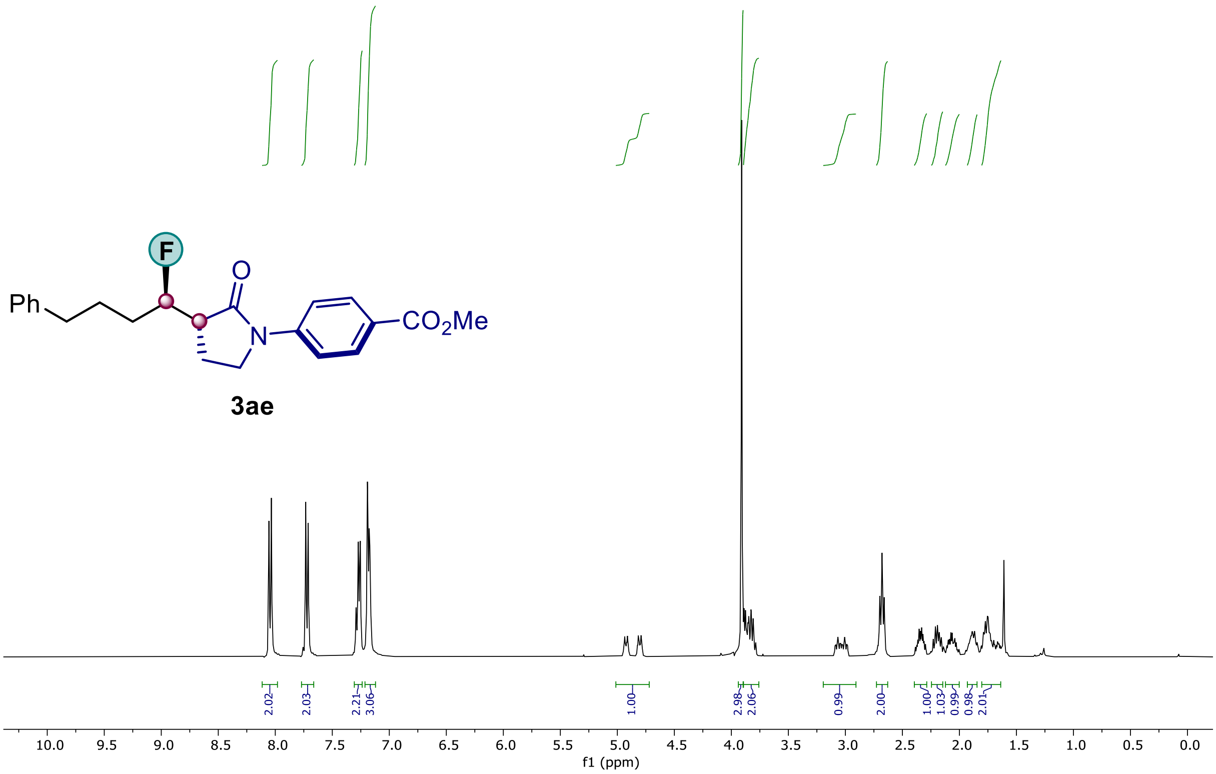
Ph
F
O
N
CO2Me
3ae
2.02
2.03
2.21
3.06
1.00
2.98
2.06
0.99
2.00
1.00
1.03
0.99
0.98
2.01
10.0 9.5 9.0 8.5 8.0 7.5 7.0 6.5 6.0 5.5 5.0 4.5 4.0 3.5 3.0 2.5 2.0 1.5 1.0 0.5 0.0
f1 (ppm)

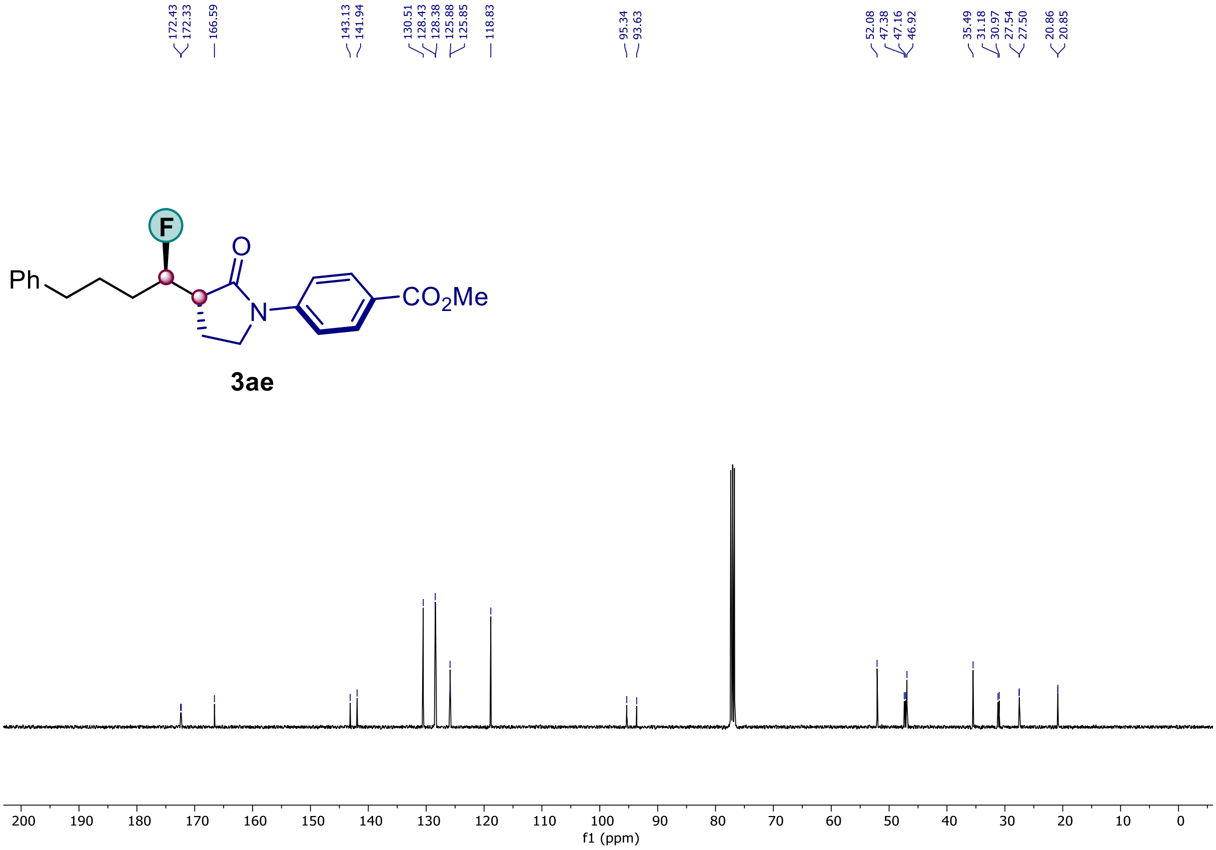
172.43
172.33
166.59
143.13
141.94
130.51
128.43
128.38
125.88
125.85
118.83
95.34
93.63
52.08
47.38
47.16
46.92
35.49
31.18
30.97
27.54
27.50
20.86
20.85
Ph
F
O
N
CO2Me
3ae
200 190 180 170 160 150 140 130 120 110 100 90 80 70 60 50 40 30 20 10 0
f1 (ppm)

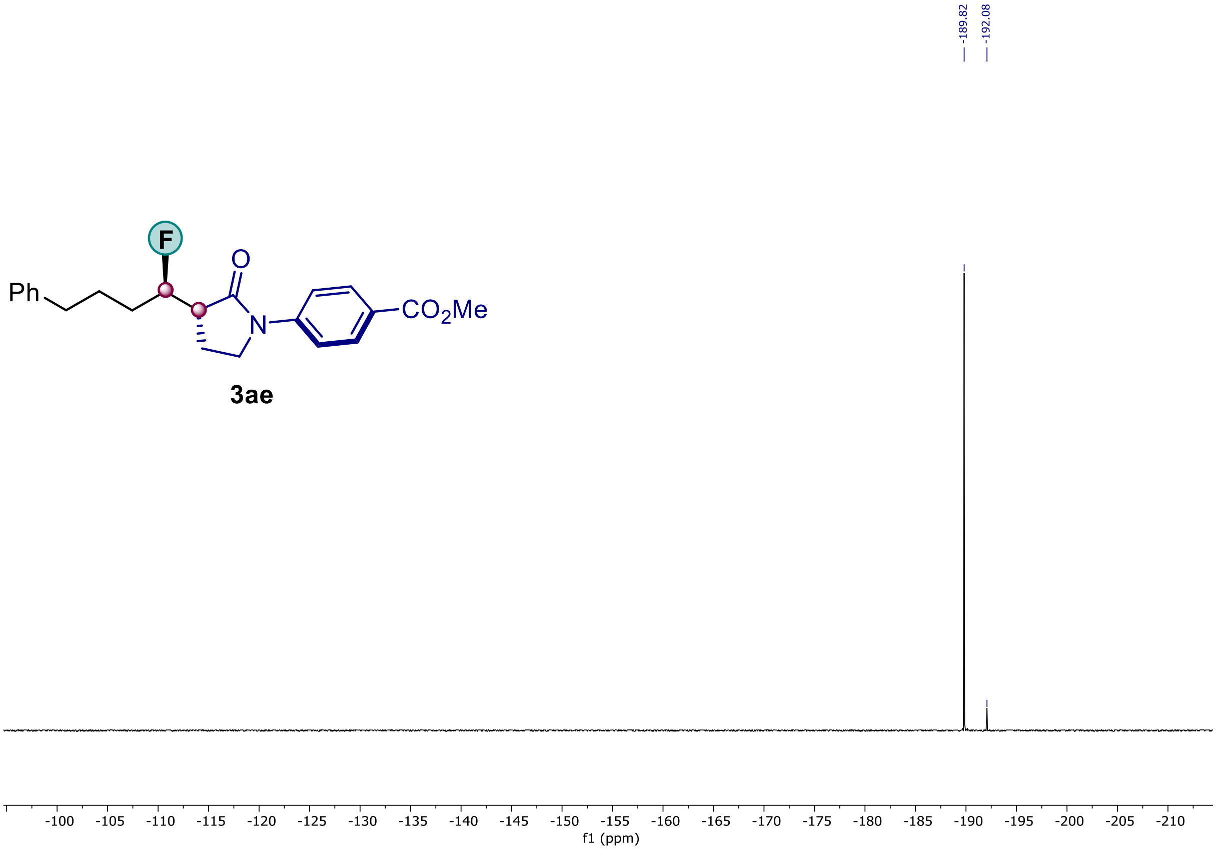
Ph
F
O
N
CO2Me
-189.82
-192.08
-100
-105
-110
-115
-120
-125
-130
-135
-140
-145
-150
-155
-160
-165
-170
-175
-180
-185
-190
-195
-200
-205
-210
f1 (ppm)

3ae

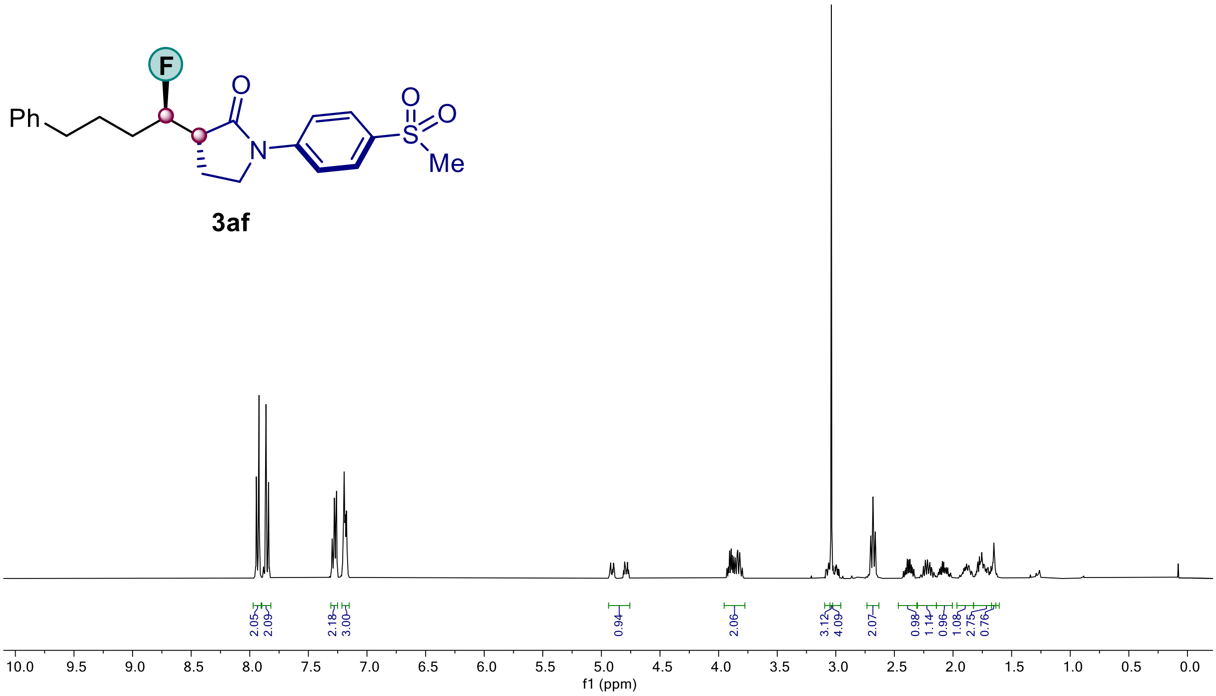
Ph
F
O
N
O
S
O
Me
3af
2.05
2.09
2.18
3.00
0.94
2.06
3.12
4.09
2.07
0.98
1.14
0.96
1.08
2.75
0.76
10.0 9.5 9.0 8.5 8.0 7.5 7.0 6.5 6.0 5.5 5.0 4.5 4.0 3.5 3.0 2.5 2.0 1.5 1.0 0.5 0.0
f1 (ppm)

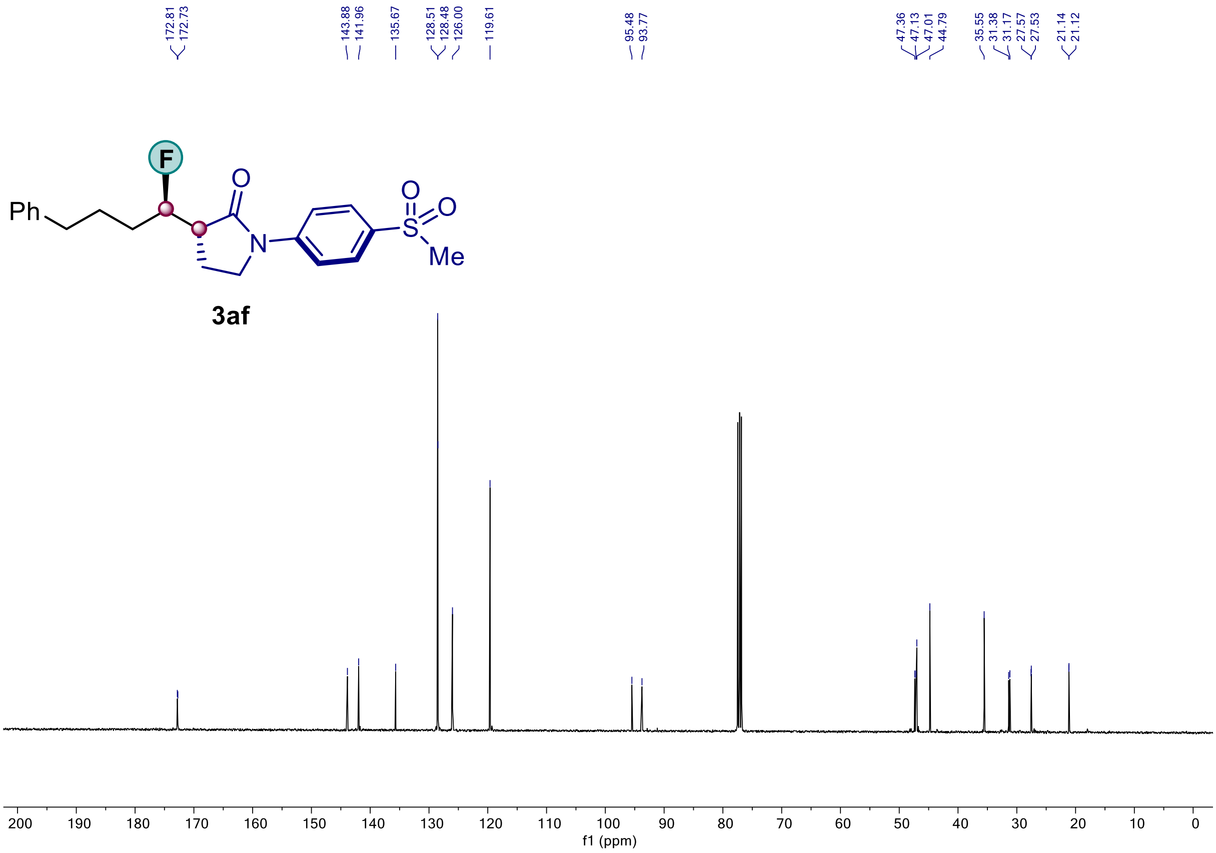
172.81
172.73
143.88
141.96
135.67
128.51
128.48
126.00
119.61
95.48
93.77
47.36
47.13
47.01
44.79
35.55
31.38
31.17
27.57
27.53
21.14
21.12
Ph
F
O
N
O
S
O
Me
3af
200 190 180 170 160 150 140 130 120 110 100 90 80 70 60 50 40 30 20 10 0
f1 (ppm)

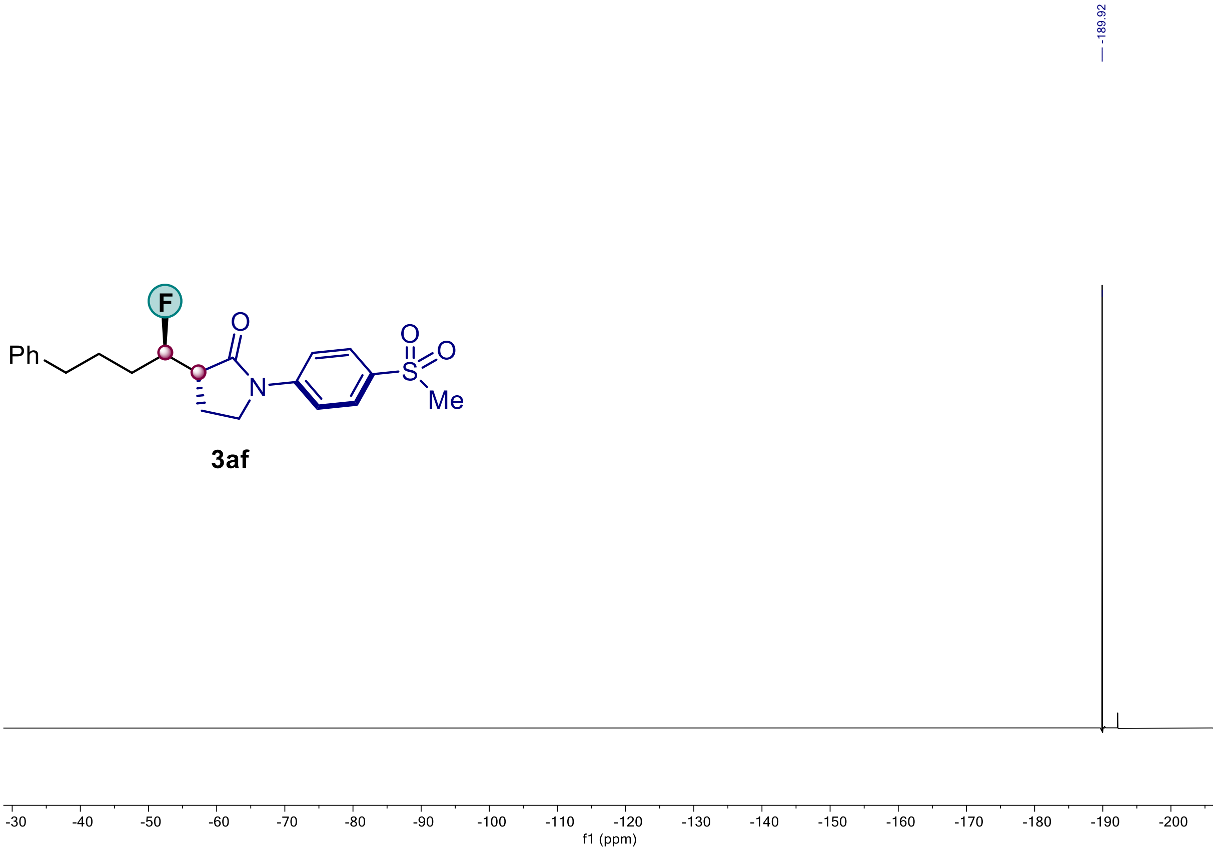
-189.92
Ph
F
O
N
O
S
O
Me
3af
-30
-40
-50
-60
-70
-80
-90
-100
-110
-120
-130
-140
-150
-160
-170
-180
-190
-200
f1 (ppm)

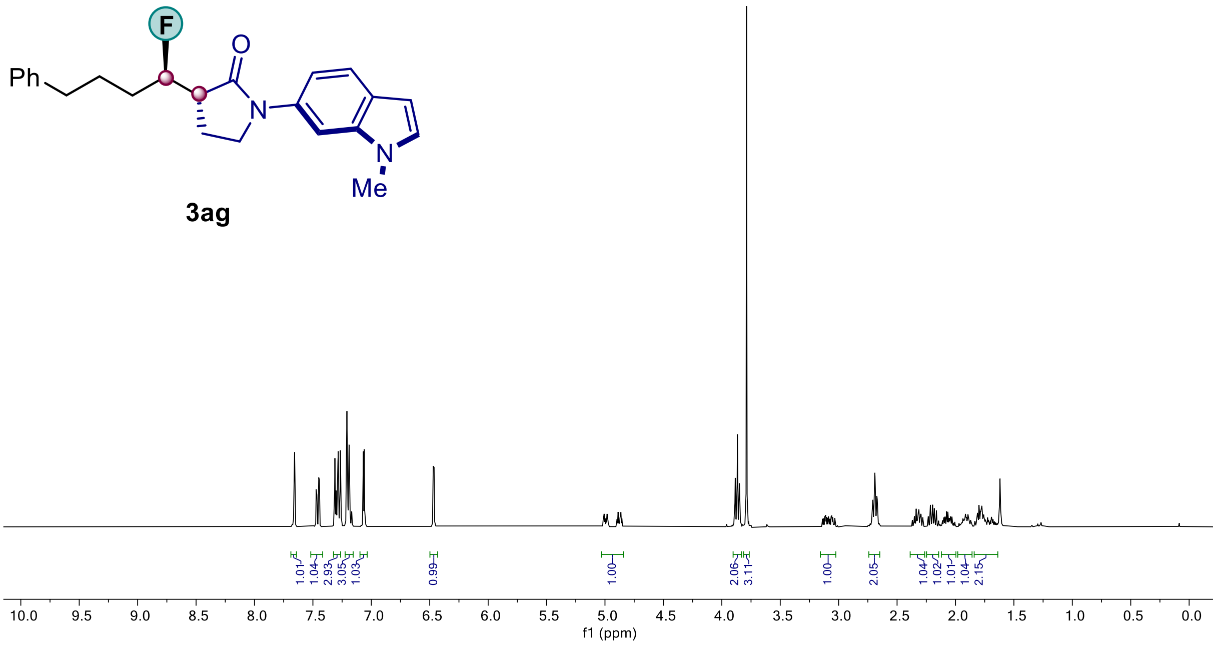
F
O
Ph
N
N
Me
3ag
1.01
1.04
2.93
3.05
1.03
0.99
1.00
2.06
3.11
1.00
2.05
1.04
1.02
1.01
1.04
2.15
10.0 9.5 9.0 8.5 8.0 7.5 7.0 6.5 6.0 5.5 5.0 4.5 4.0 3.5 3.0 2.5 2.0 1.5 1.0 0.5 0.0
f1 (ppm)

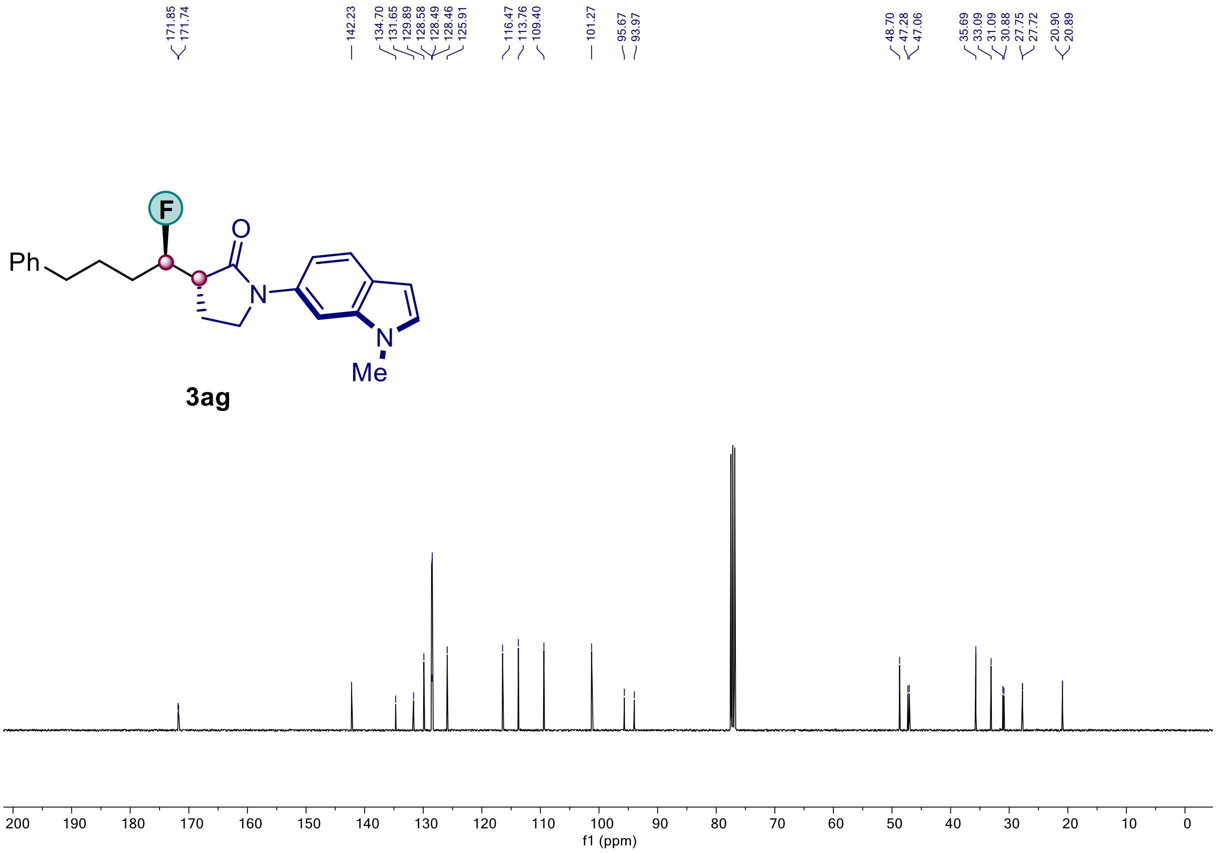
171.85
171.74
142.23
134.70
131.65
129.89
128.58
128.49
128.46
125.91
116.47
113.76
109.40
101.27
95.67
93.97
48.70
47.28
47.06
35.69
33.09
31.09
30.88
27.75
27.72
20.90
20.89
F
O
Ph
N
N
Me
3ag
200 190 180 170 160 150 140 130 120 110 100 90 80 70 60 50 40 30 20 10 0
f1 (ppm)

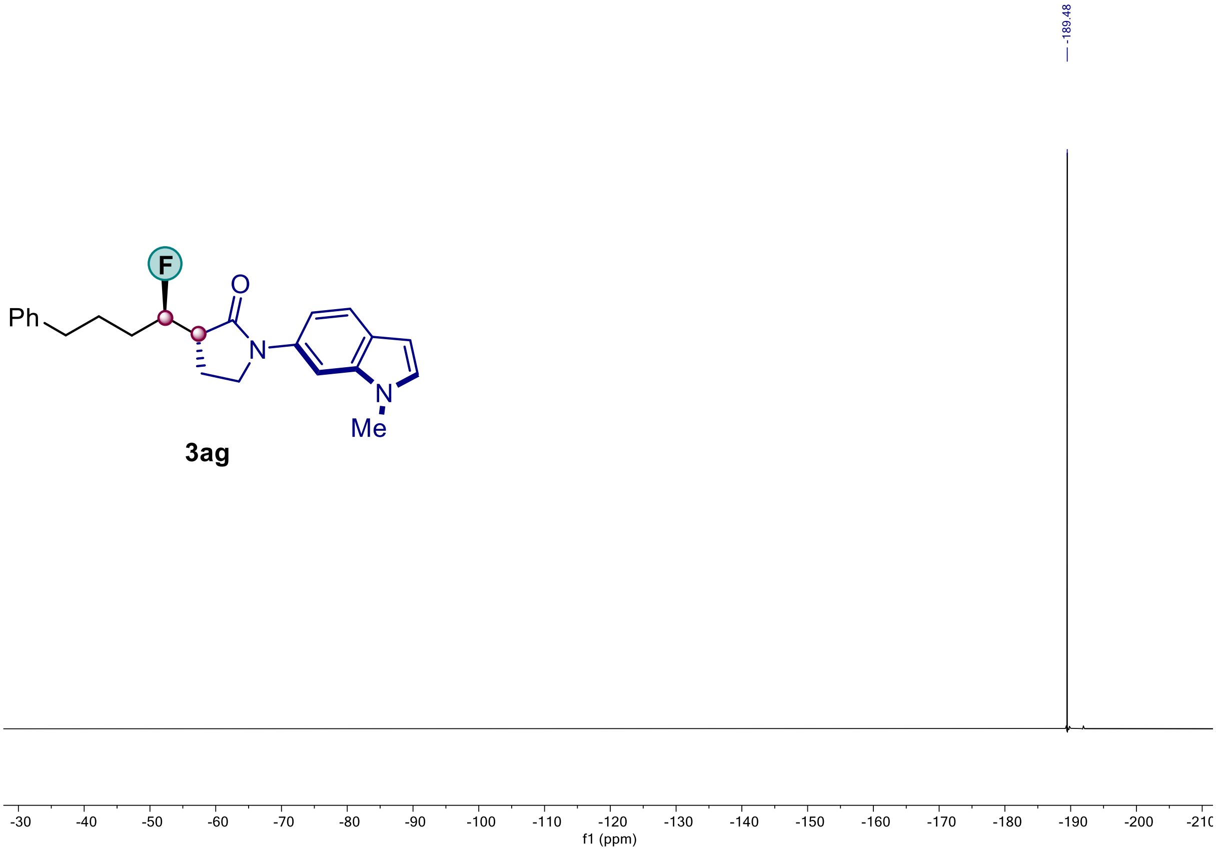
-189.48
Ph
F
O
N
N
Me
3ag
-30
-40
-50
-60
-70
-80
-90
-100
-110
-120
-130
-140
-150
-160
-170
-180
-190
-200
-210
f1 (ppm)

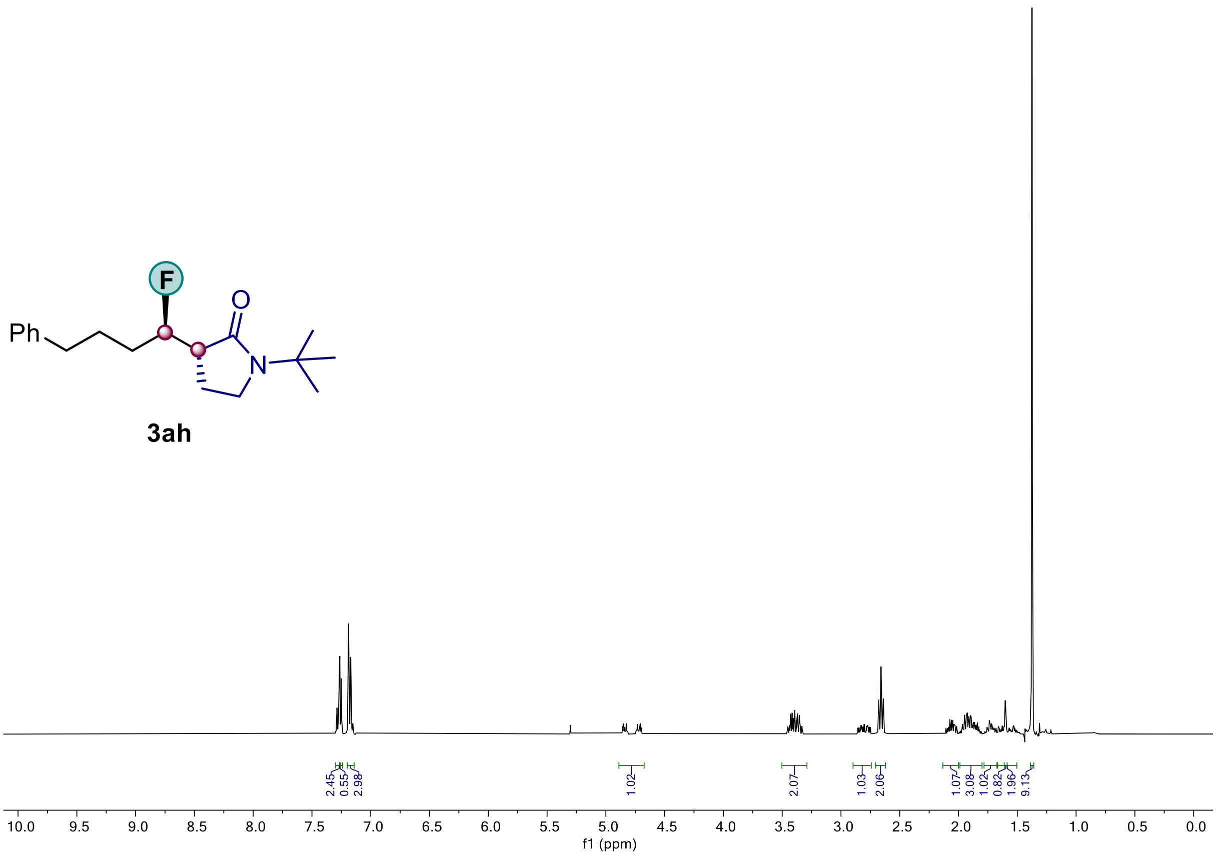

Ph
F
O
N
3ah
2.45
0.55
2.98
1.02
2.07
1.03
2.06
1.07
3.08
1.02
0.82
1.96
9.13
10.0 9.5 9.0 8.5 8.0 7.5 7.0 6.5 6.0 5.5 5.0 4.5 4.0 3.5 3.0 2.5 2.0 1.5 1.0 0.5 0.0
f1 (ppm)

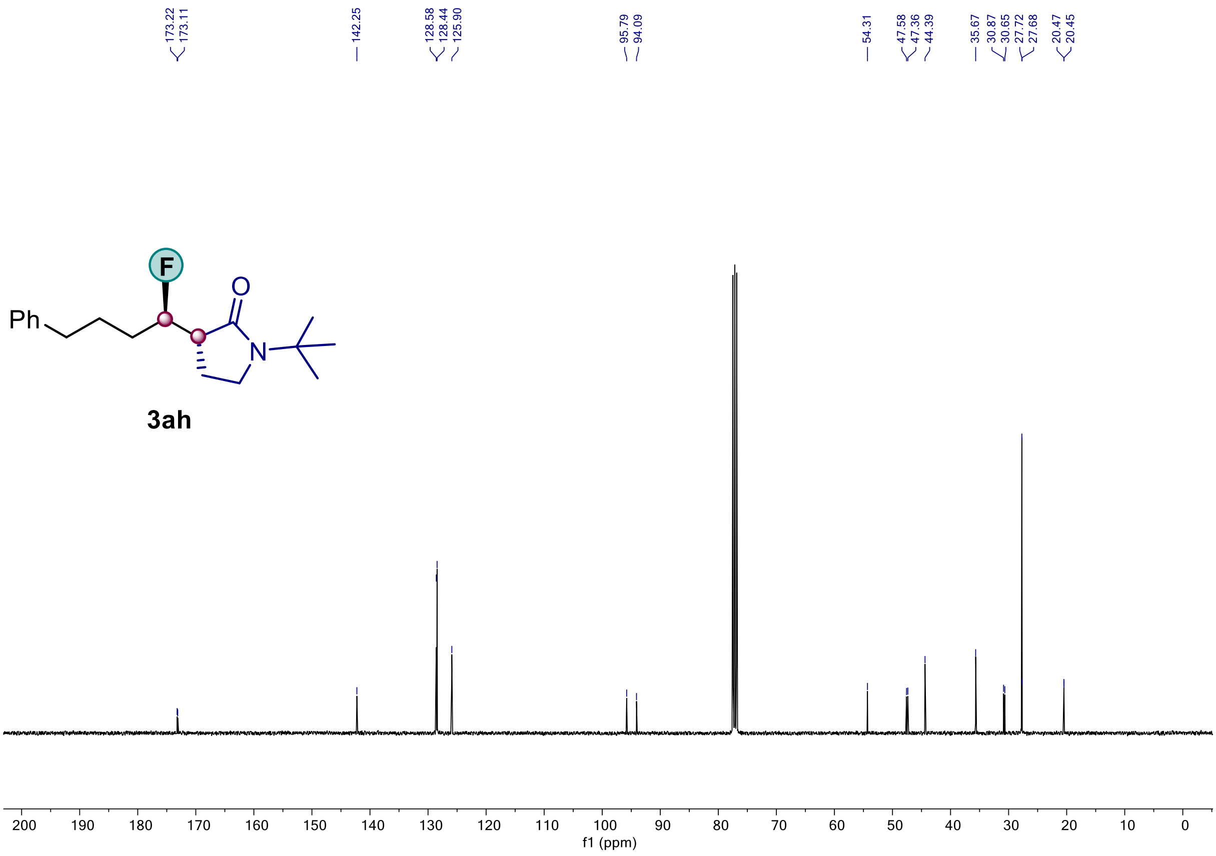

173.22
173.11
142.25
128.58
128.44
125.90
95.79
94.09
54.31
47.58
47.36
44.39
35.67
30.87
30.65
27.72
27.68
20.47
20.45
Ph
F
O
N
3ah
200 190 180 170 160 150 140 130 120 110 100 90 80 70 60 50 40 30 20 10 0
f1 (ppm)

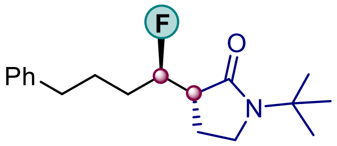

3ah

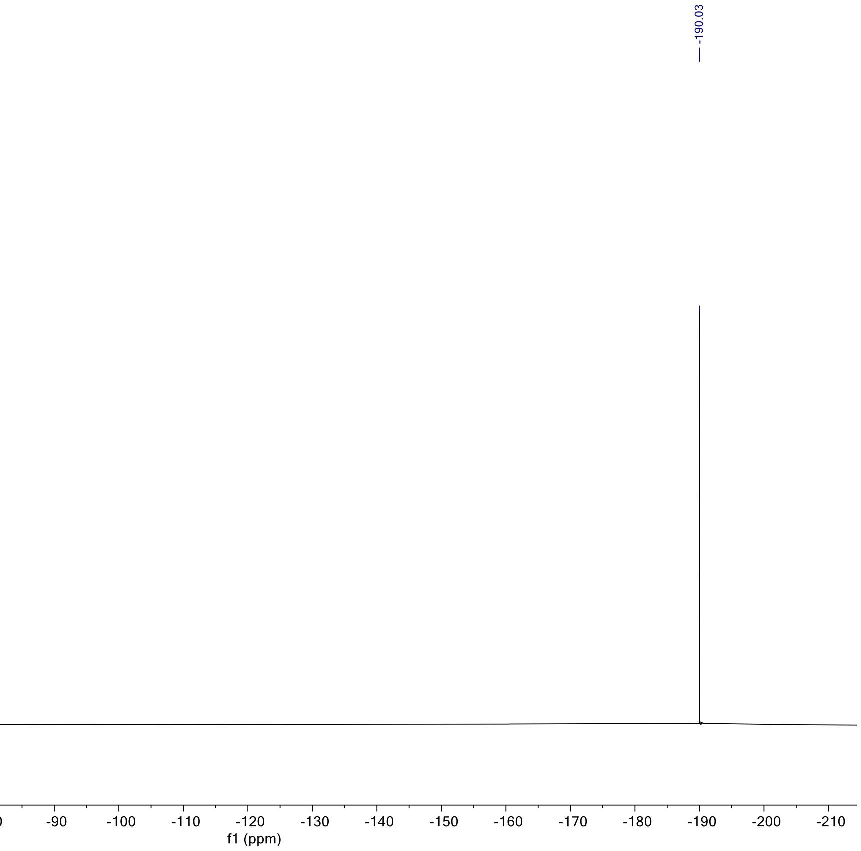
-190.03
-30
-40
-50
-60
-70
-80
-90
-100
-110
-120
-130
-140
-150
-160
-170
-180
-190
-200
-210
f1 (ppm)

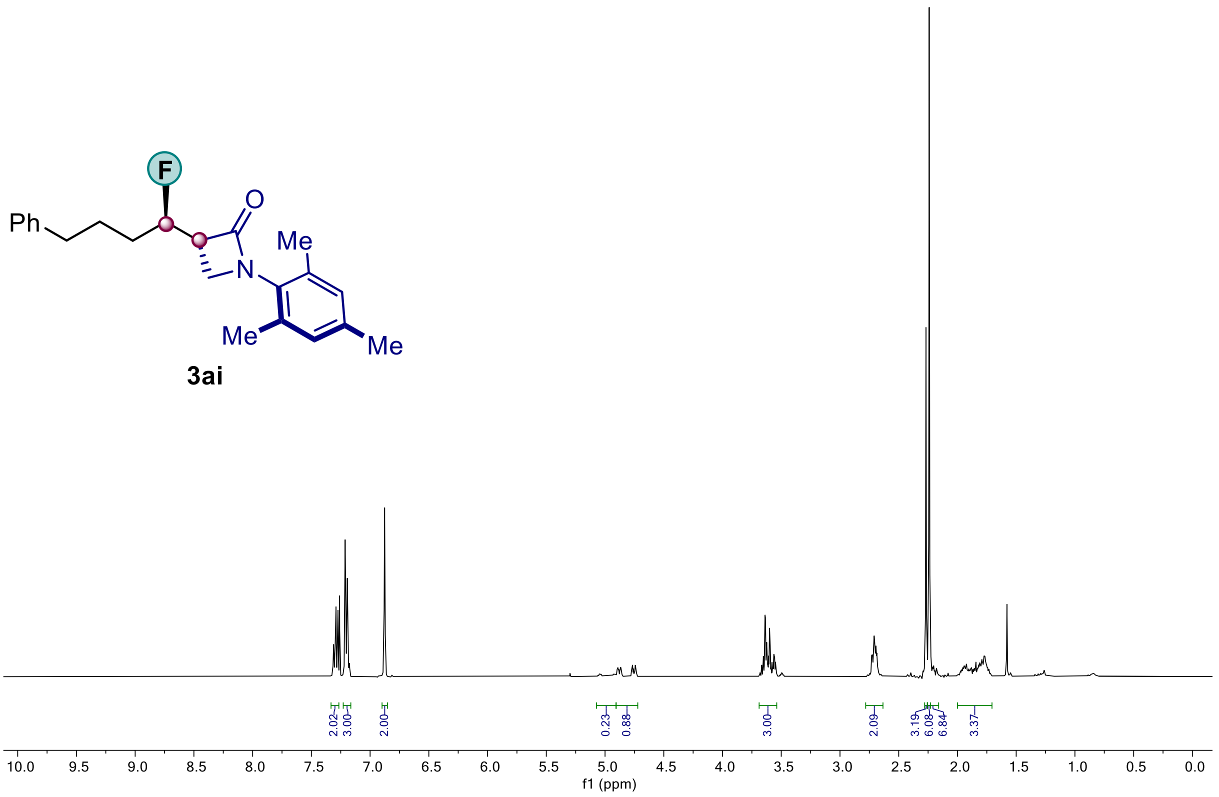
F
O
Ph
N
Me
Me
Me
3ai
2.02
3.00
2.00
0.23
0.88
3.00
2.09
3.19
6.08
6.84
3.37
10.0 9.5 9.0 8.5 8.0 7.5 7.0 6.5 6.0 5.5 5.0 4.5 4.0 3.5 3.0 2.5 2.0 1.5 1.0 0.5 0.0
f1 (ppm)

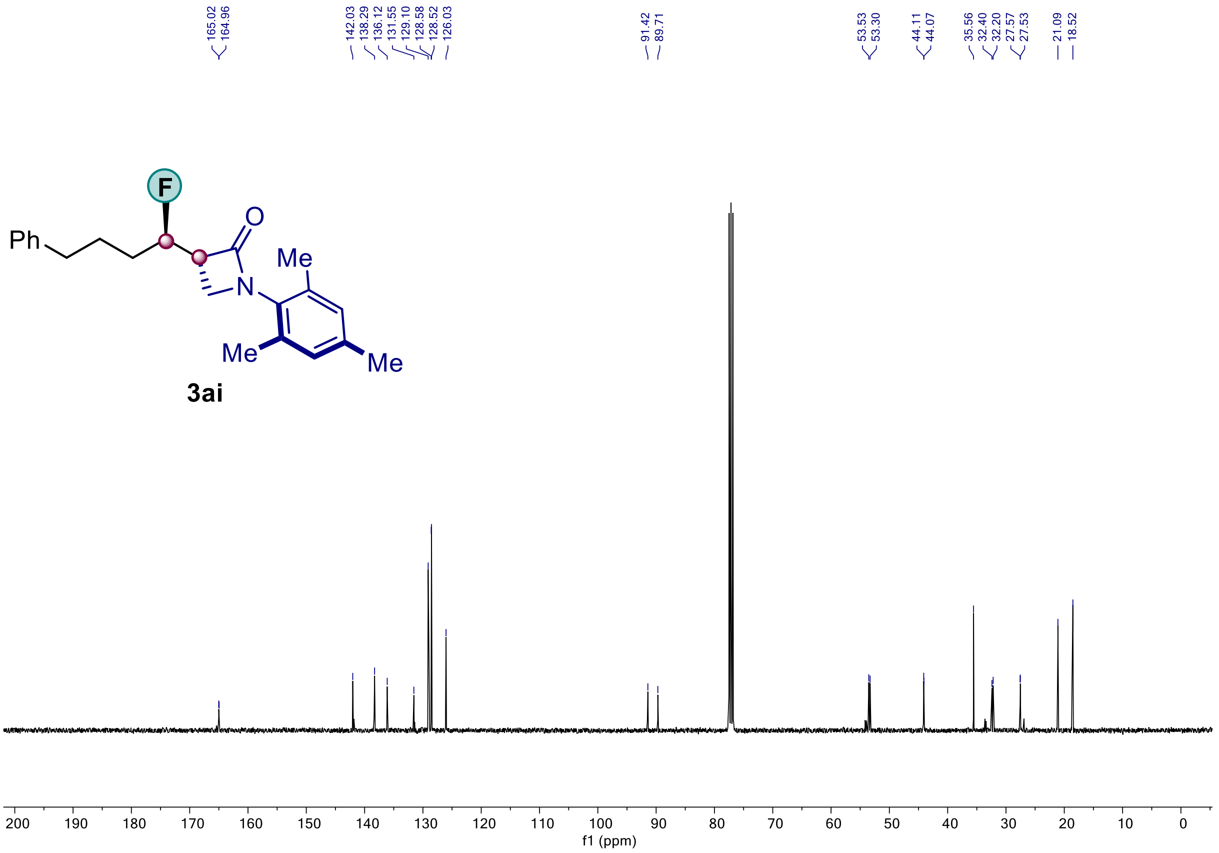
165.02
164.96
142.03
138.29
136.12
131.55
129.10
128.58
128.52
126.03
91.42
89.71
53.53
53.30
44.11
44.07
35.56
32.40
32.20
27.57
27.53
21.09
18.52
F
O
Ph
N
Me
Me
Me
3ai
200 190 180 170 160 150 140 130 120 110 100 90 80 70 60 50 40 30 20 10 0
f1 (ppm)

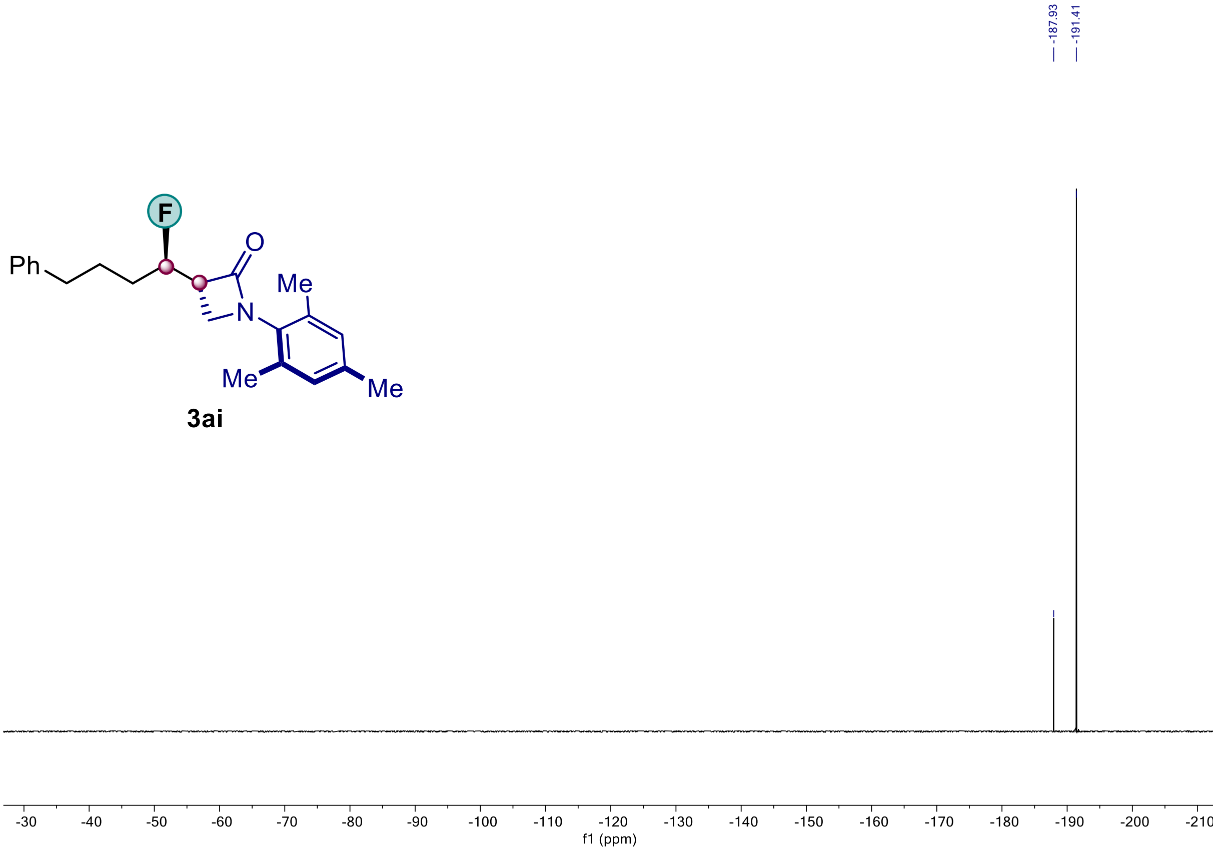
F
O
Ph
N
Me
Me
Me
3ai
-187.93
-191.41
-30 -40 -50 -60 -70 -80 -90 -100 -110 -120 -130 -140 -150 -160 -170 -180 -190 -200 -210
f1 (ppm)

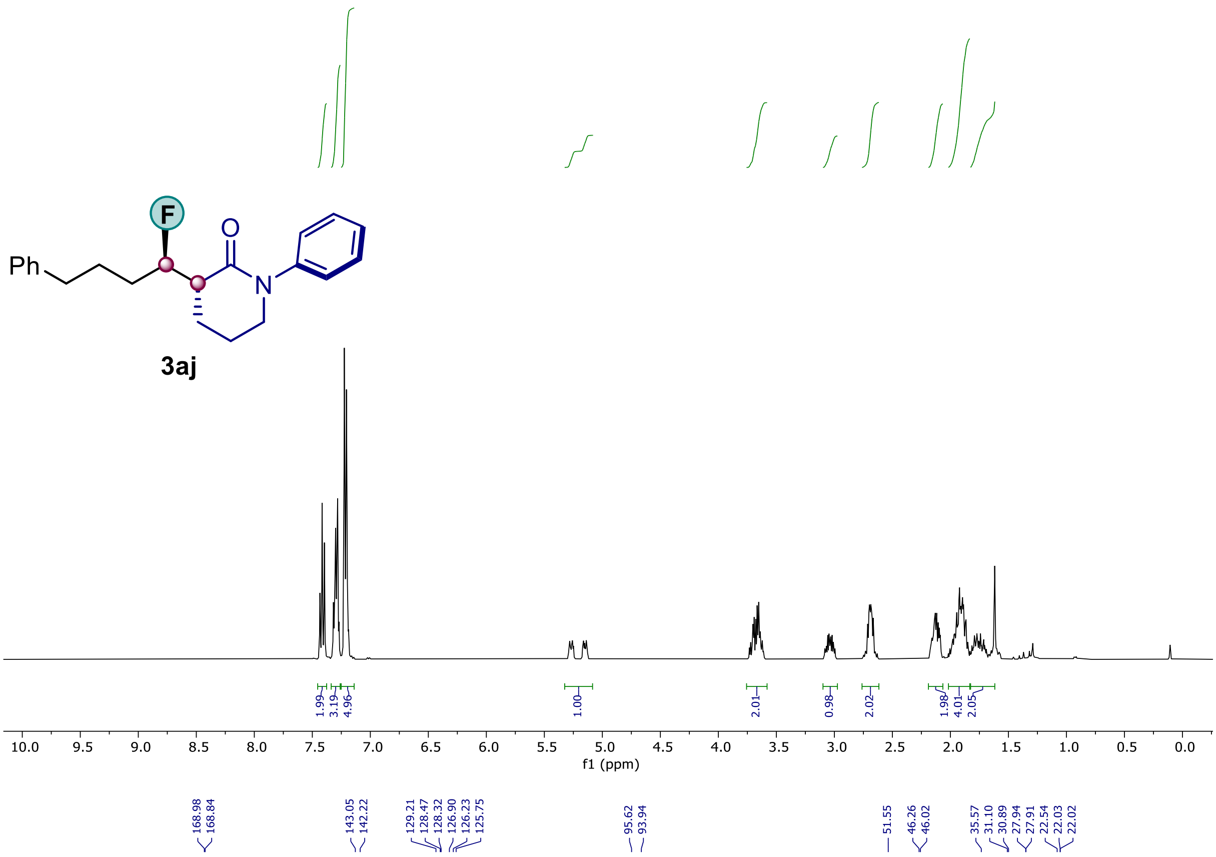
Ph
F
O
N
3aj
1.99
3.19
4.96
1.00
2.01
0.98
2.02
1.98
4.01
2.05
10.0 9.5 9.0 8.5 8.0 7.5 7.0 6.5 6.0 5.5 5.0 4.5 4.0 3.5 3.0 2.5 2.0 1.5 1.0 0.5 0.0
f1 (ppm)
168.98
168.84
143.05
142.22
129.21
128.47
128.32
126.90
126.23
125.75
95.62
93.94
51.55
46.26
46.02
35.57
31.10
30.89
27.94
27.91
22.54
22.03
22.02

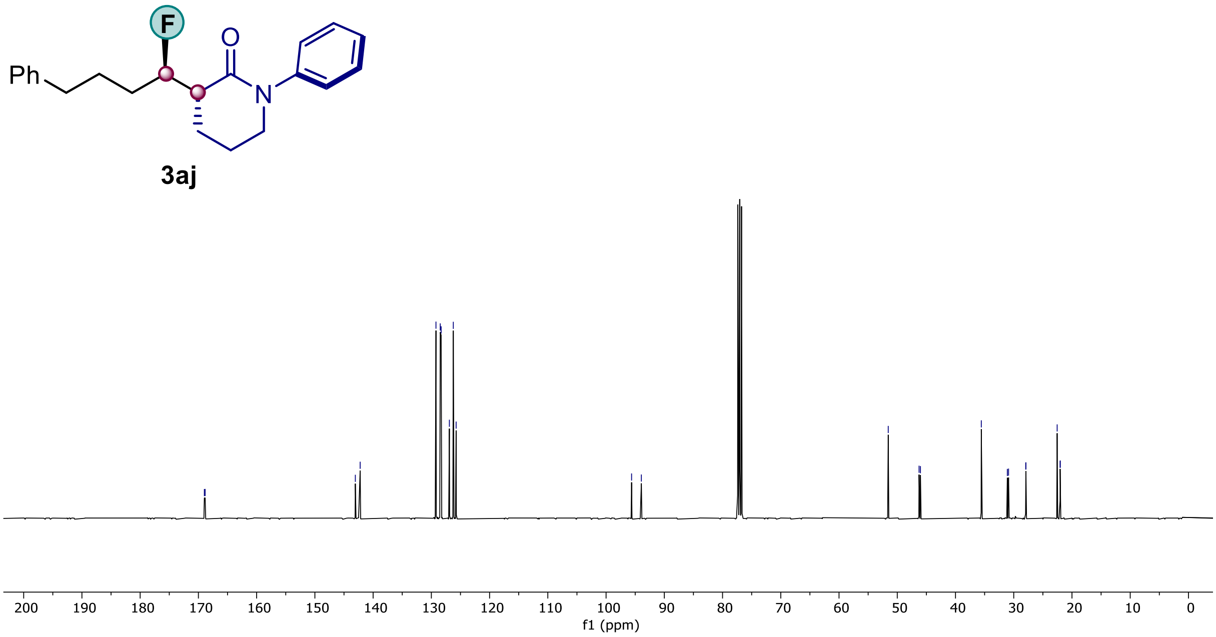
Ph
F
O
N
3aj
200 190 180 170 160 150 140 130 120 110 100 90 80 70 60 50 40 30 20 10 0
f1 (ppm)

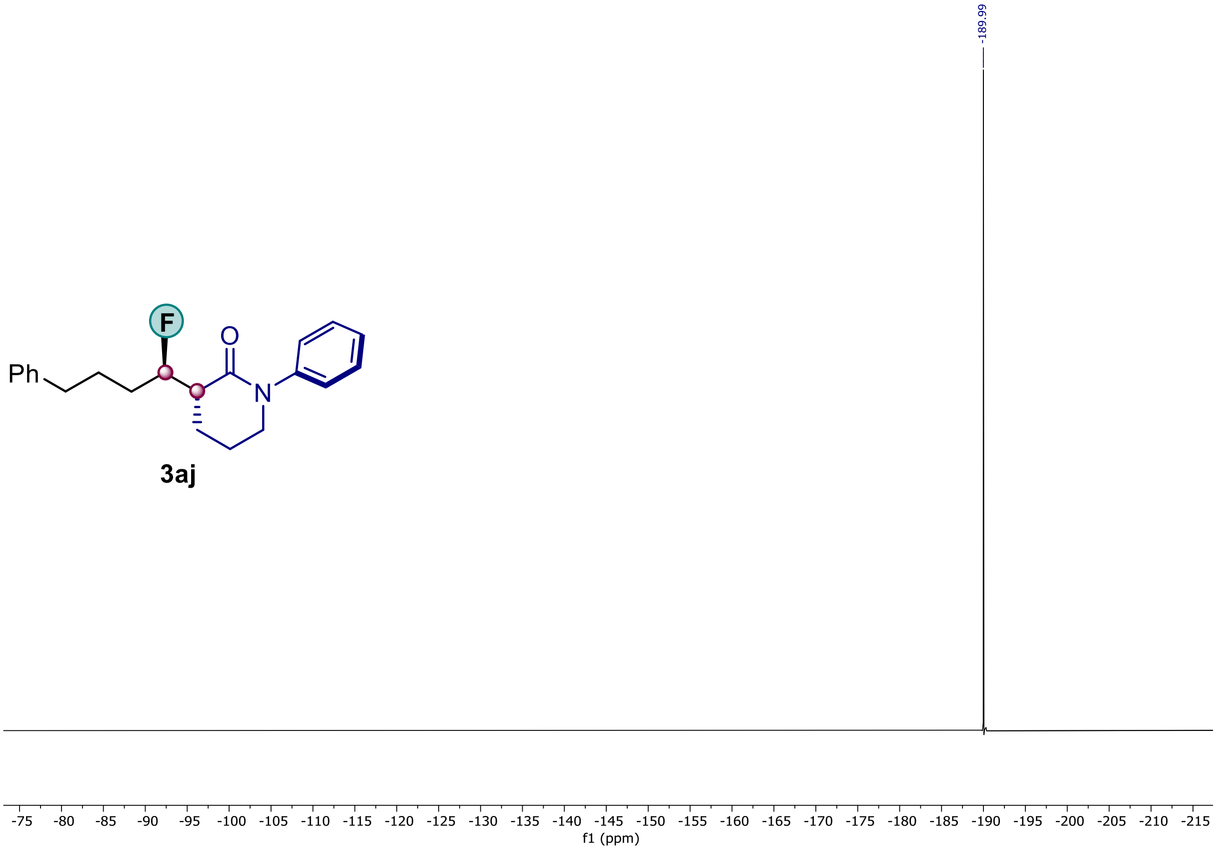
-189.99
Ph
F
O
N
3aj
-75 -80 -85 -90 -95 -100 -105 -110 -115 -120 -125 -130 -135 -140 -145 -150 -155 -160 -165 -170 -175 -180 -185 -190 -195 -200 -205 -210 -215
f1 (ppm)

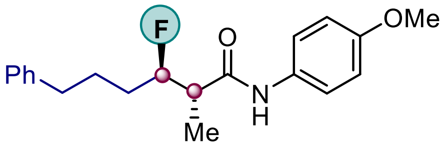

5aa

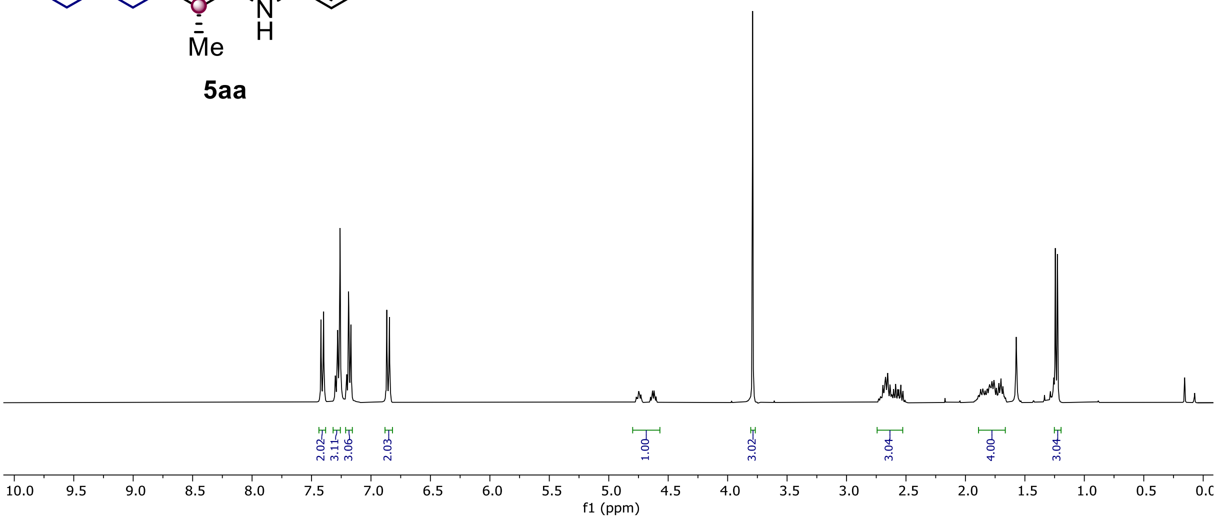

2.02
3.11
3.06
2.03
1.00
3.02
3.04
4.00
3.04
10.0 9.5 9.0 8.5 8.0 7.5 7.0 6.5 6.0 5.5 5.0 4.5 4.0 3.5 3.0 2.5 2.0 1.5 1.0 0.5 0.0
f1 (ppm)

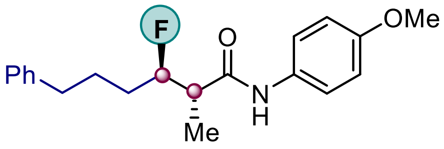

5aa

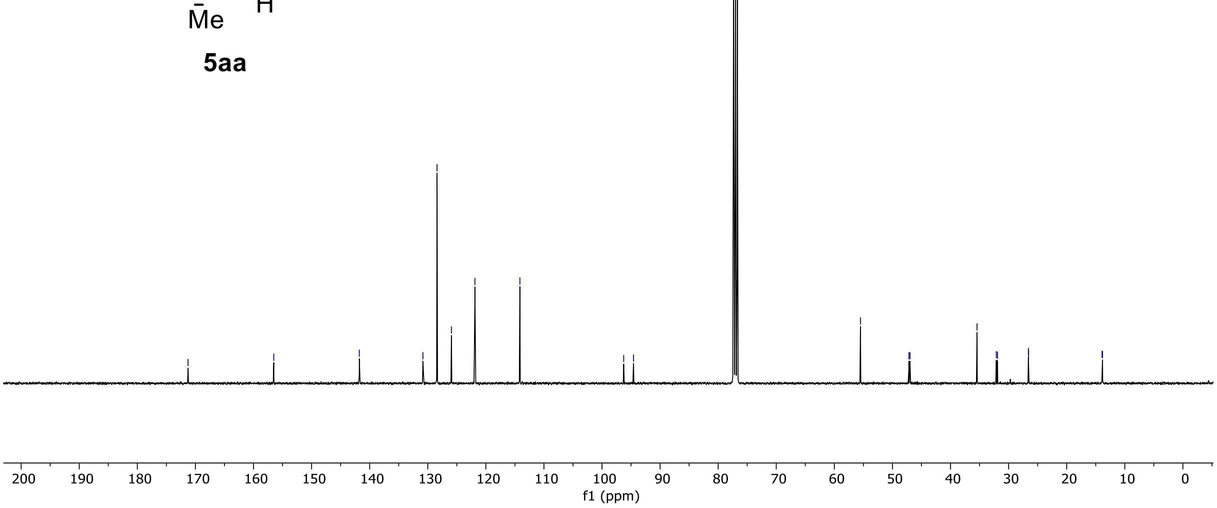

171.27
156.51
141.78
130.83
128.40
125.93
121.89
114.14
96.27
94.57
55.51
47.17
46.97
35.43
32.12
31.91
26.59
26.56
13.91
13.84
200 190 180 170 160 150 140 130 120 110 100 90 80 70 60 50 40 30 20 10 0
f1 (ppm)

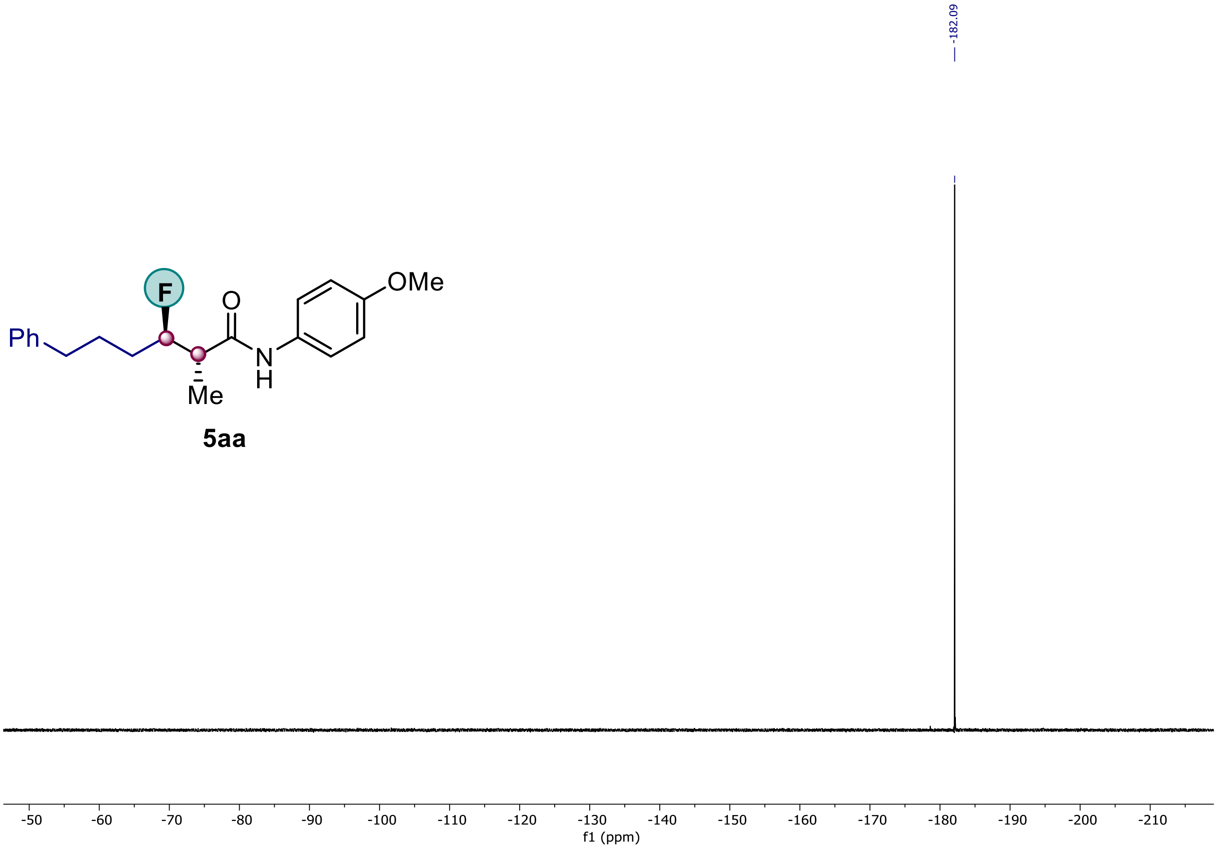
-182.09
Ph
F
O
OMe
N
H
Me
5aa
-50
-60
-70
-80
-90
-100
-110
-120
-130
-140
-150
-160
-170
-180
-190
-200
-210
f1 (ppm)

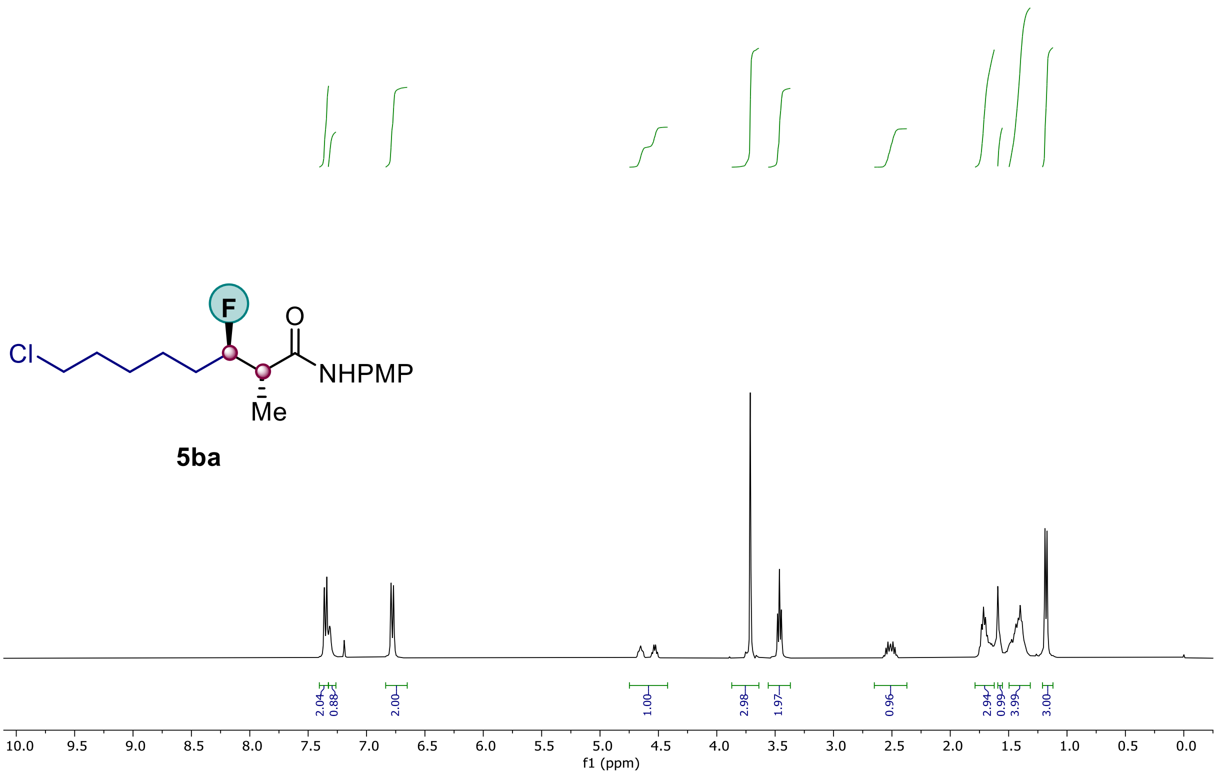
F
O
Cl
NHPMP
Me
5ba
2.04
0.88
2.00
1.00
2.98
1.97
0.96
2.94
0.99
3.99
3.00
10.0 9.5 9.0 8.5 8.0 7.5 7.0 6.5 6.0 5.5 5.0 4.5 4.0 3.5 3.0 2.5 2.0 1.5 1.0 0.5 0.0
f1 (ppm)

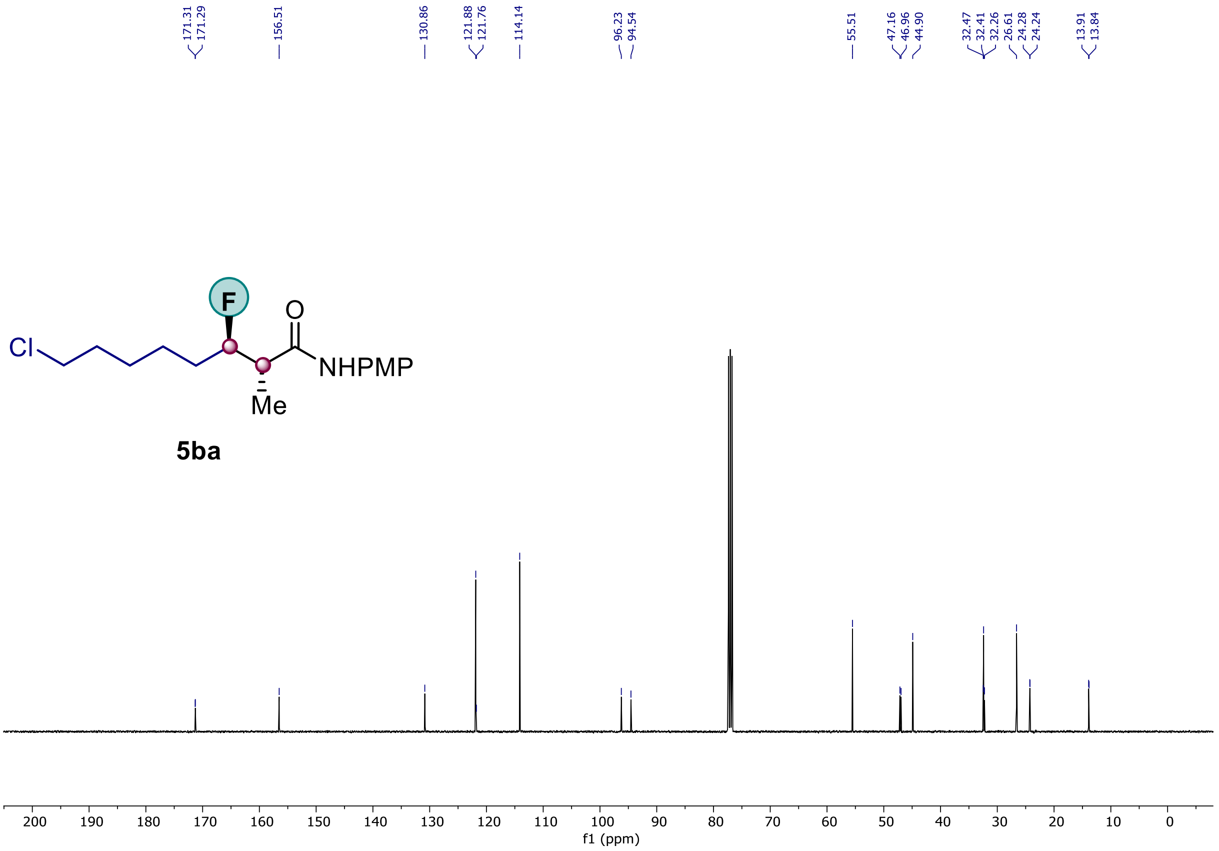
171.31
171.29
156.51
130.86
121.88
121.76
114.14
96.23
94.54
55.51
47.16
46.96
44.90
32.47
32.41
32.26
26.61
24.28
24.24
13.91
13.84
F
O
Cl
NHPMP
Me
5ba
200 190 180 170 160 150 140 130 120 110 100 90 80 70 60 50 40 30 20 10 0
f1 (ppm)

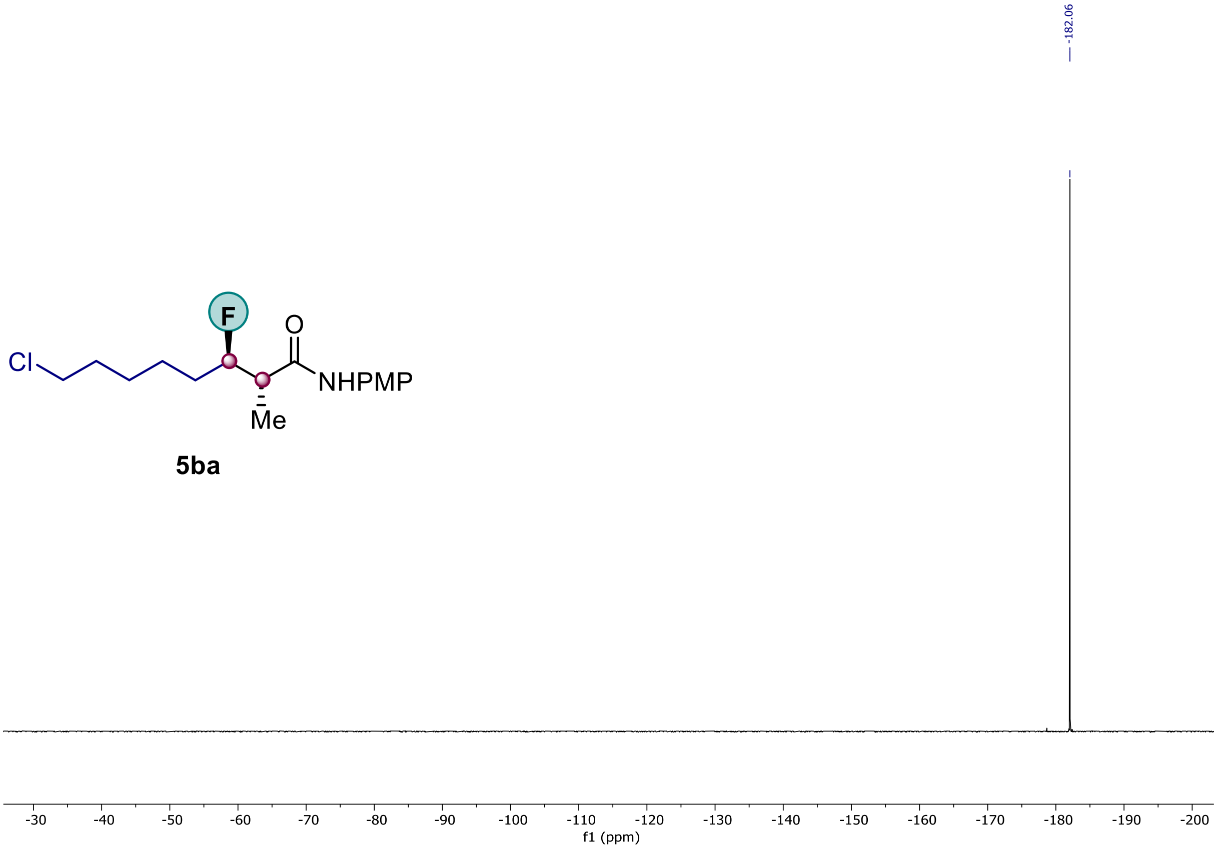
Cl
F
O
NHPMP
Me
-182.06
-30
-40
-50
-60
-70
-80
-90
-100
-110
-120
-130
-140
-150
-160
-170
-180
-190
-200
f1 (ppm)

5ba

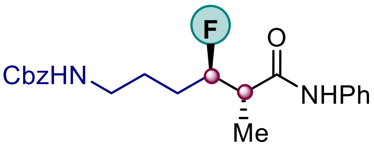
F
O
CbzHN
NHPh
Me

5cb

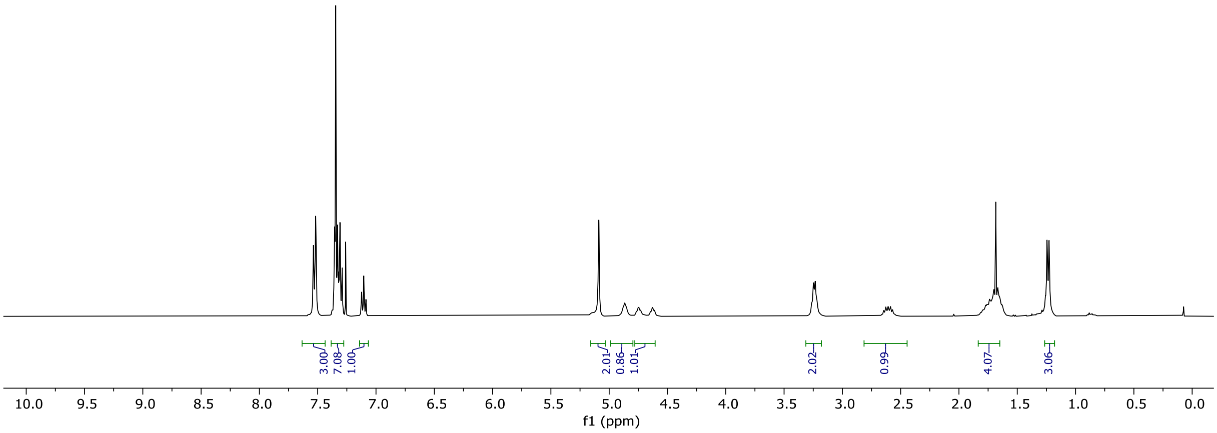
3.00
7.08
1.00
2.01
0.86
1.01
2.02
0.99
4.07
3.06
10.0 9.5 9.0 8.5 8.0 7.5 7.0 6.5 6.0 5.5 5.0 4.5 4.0 3.5 3.0 2.5 2.0 1.5 1.0 0.5 0.0
f1 (ppm)

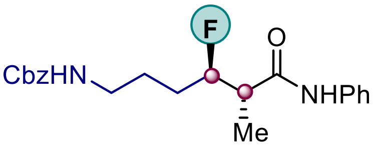
F
O
CbzHN
NHPh
Me

5cb

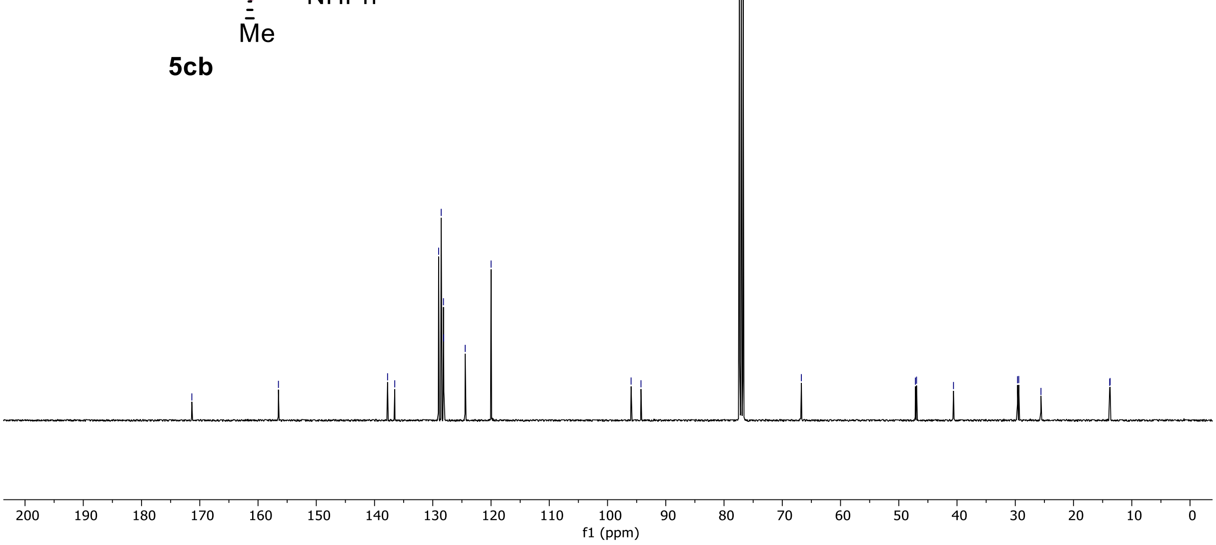
171.36
156.50
137.76
136.53
129.00
128.56
128.17
128.12
124.44
119.99
95.96
94.26
66.72
47.15
46.95
40.60
29.61
29.40
25.59
13.79
13.72
200 190 180 170 160 150 140 130 120 110 100 90 80 70 60 50 40 30 20 10 0
f1 (ppm)

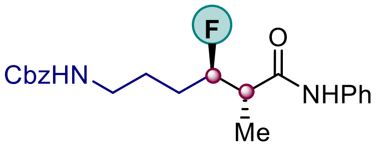
F
O
CbzHN
NHPh
Me

5cb

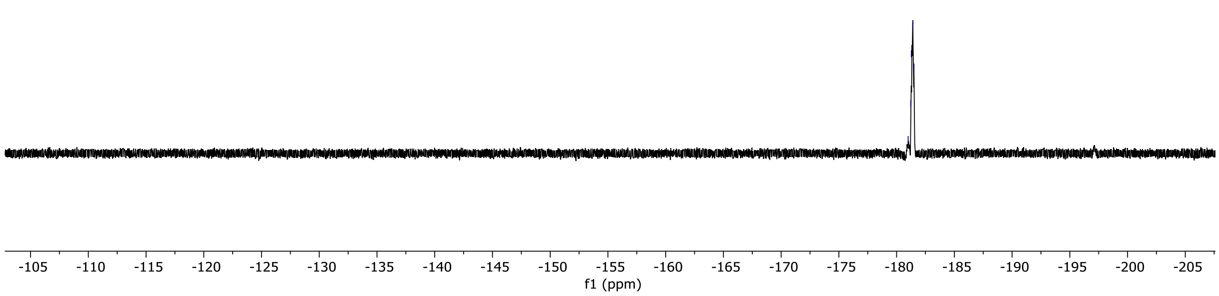
-181.00
-181.23
-181.28
-181.39
-181.50
-105 -110 -115 -120 -125 -130 -135 -140 -145 -150 -155 -160 -165 -170 -175 -180 -185 -190 -195 -200 -205
f1 (ppm)

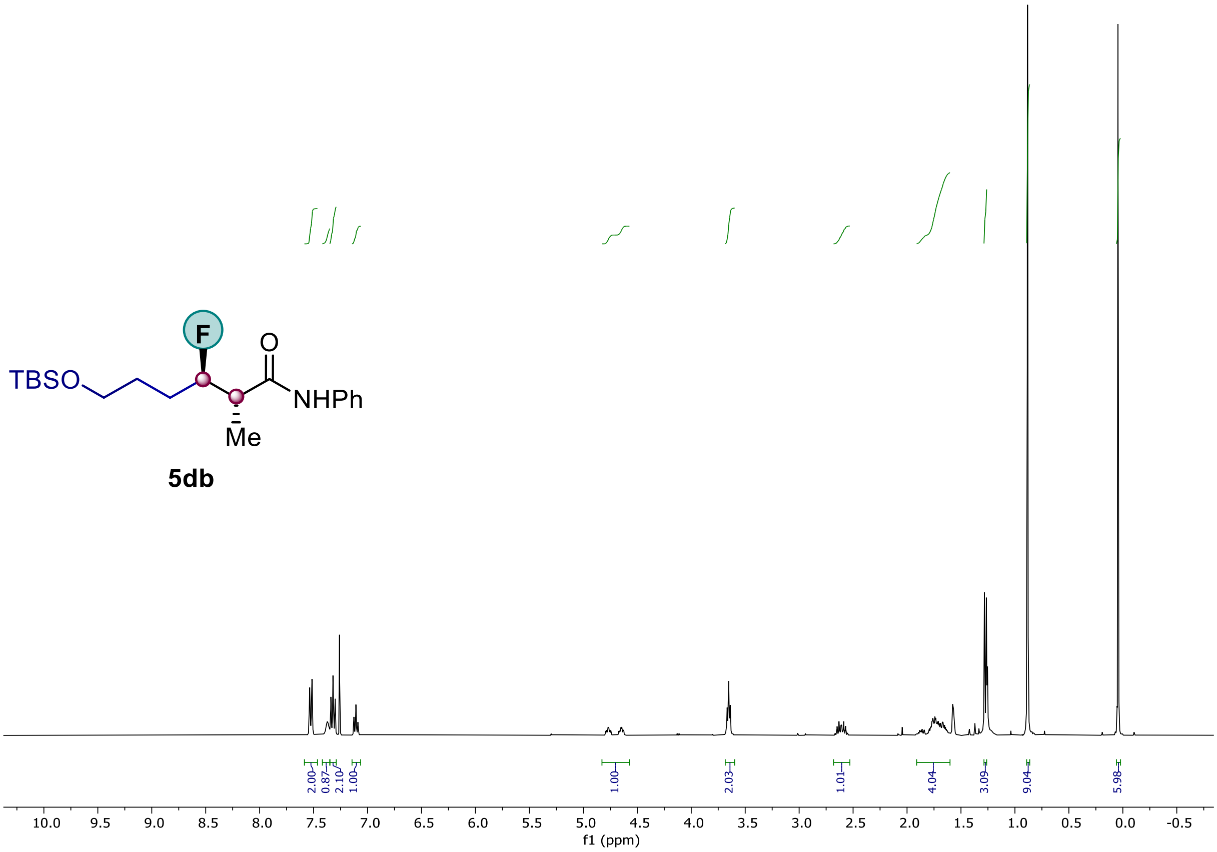
TBSO
F
O
NHPh
Me
5db
2.00
0.87
2.10
1.00
1.00
2.03
1.01
4.04
3.09
9.04
5.98
f1 (ppm)

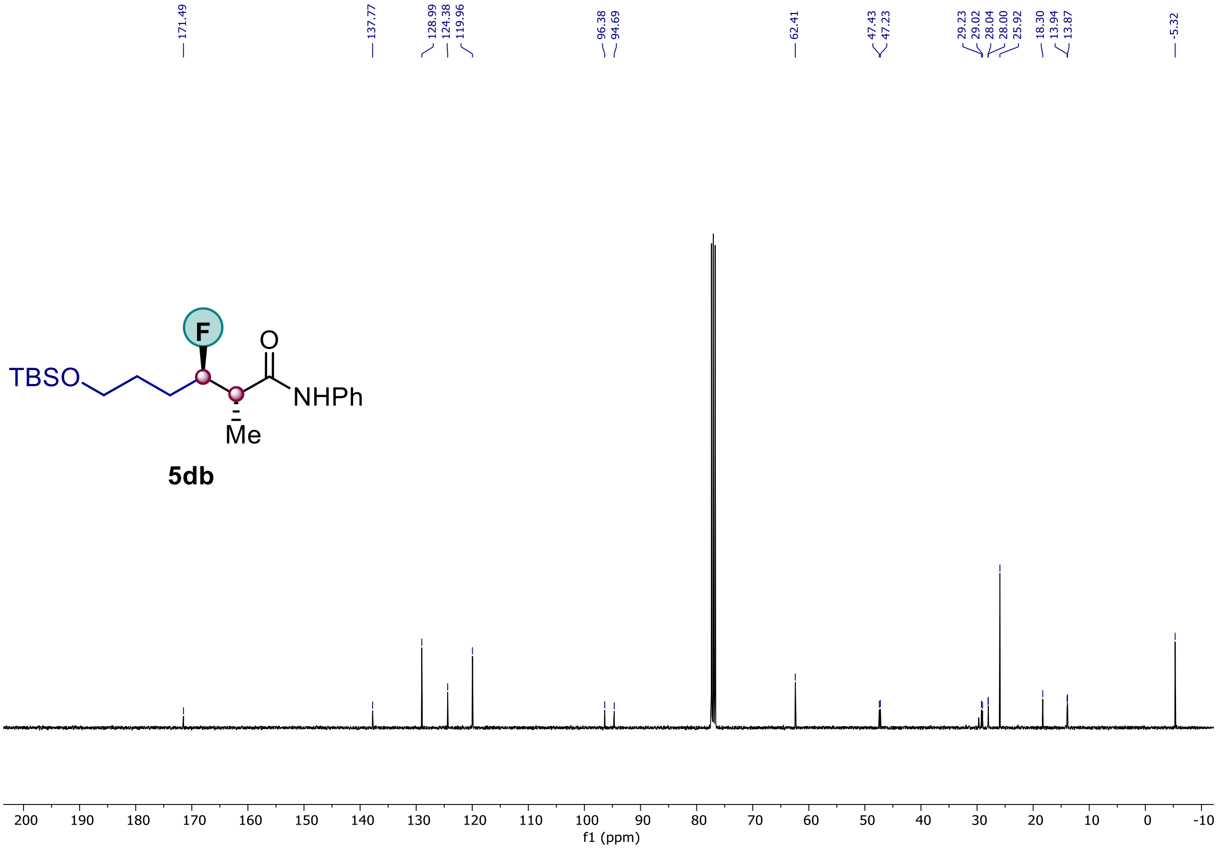
171.49
137.77
128.99
124.38
119.96
96.38
94.69
62.41
47.43
47.23
29.23
29.02
28.04
28.00
25.92
18.30
13.94
13.87
-5.32
TBSO
F
O
NHPh
Me
5db
f1 (ppm)

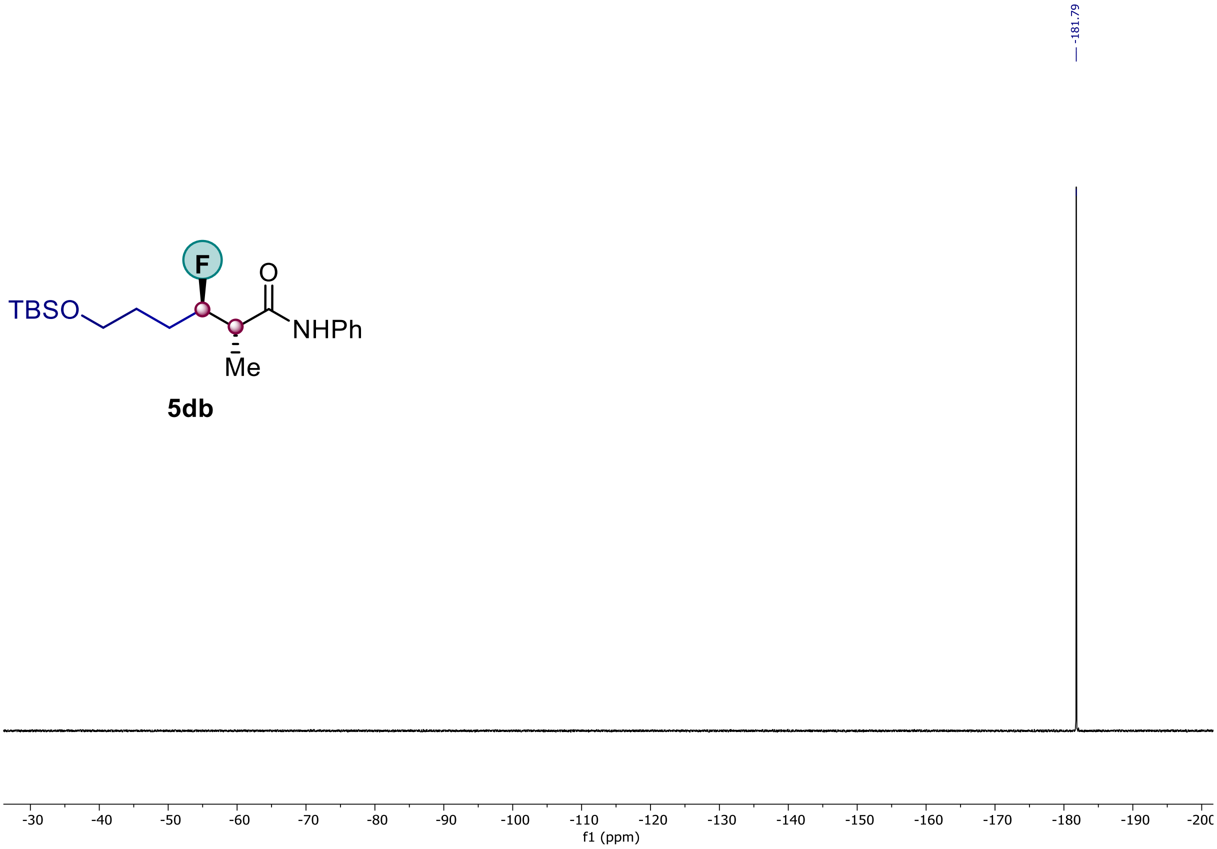
TBSO
F
O
NHPh
Me
-181.79
-30
-40
-50
-60
-70
-80
-90
-100
-110
-120
-130
-140
-150
-160
-170
-180
-190
-200
f1 (ppm)

5db

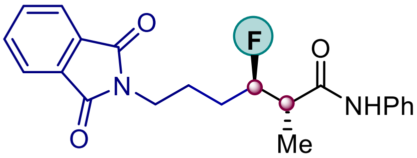

5eb

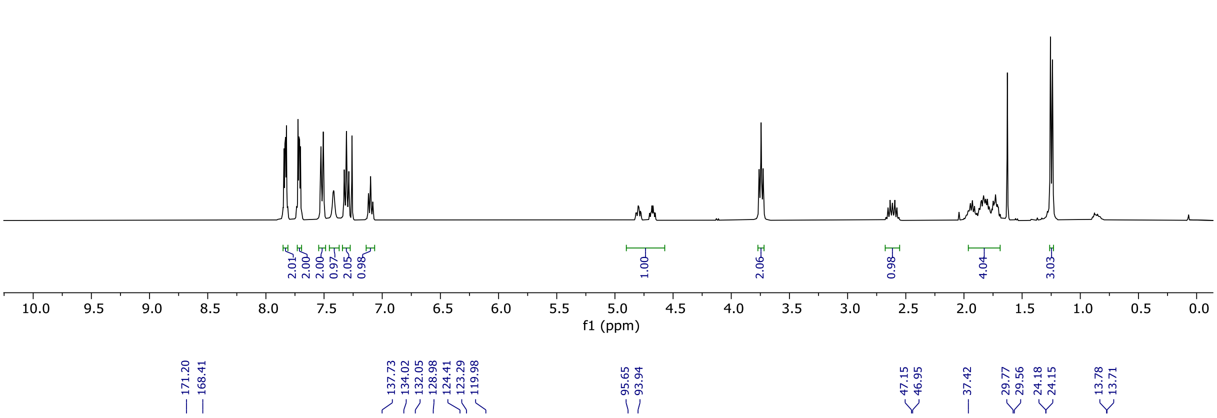

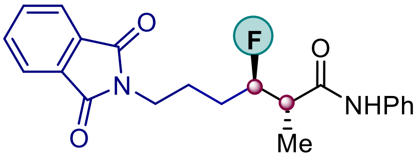

5eb

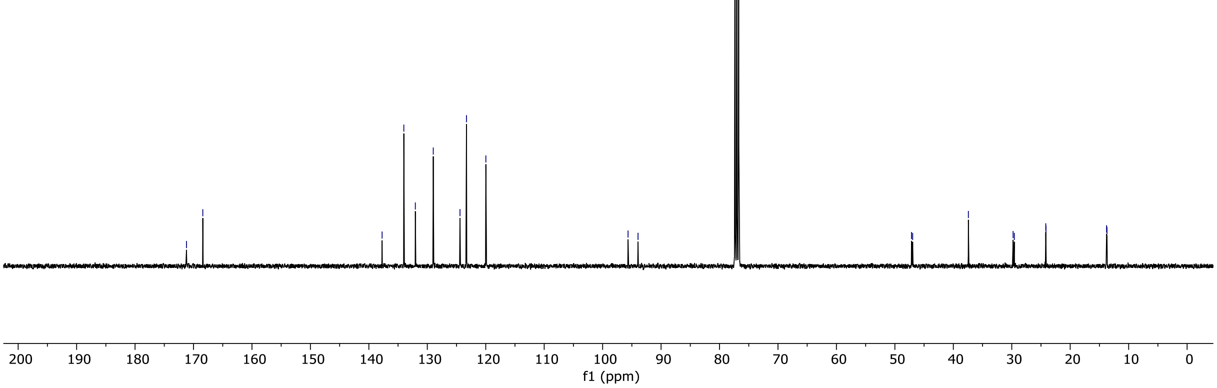

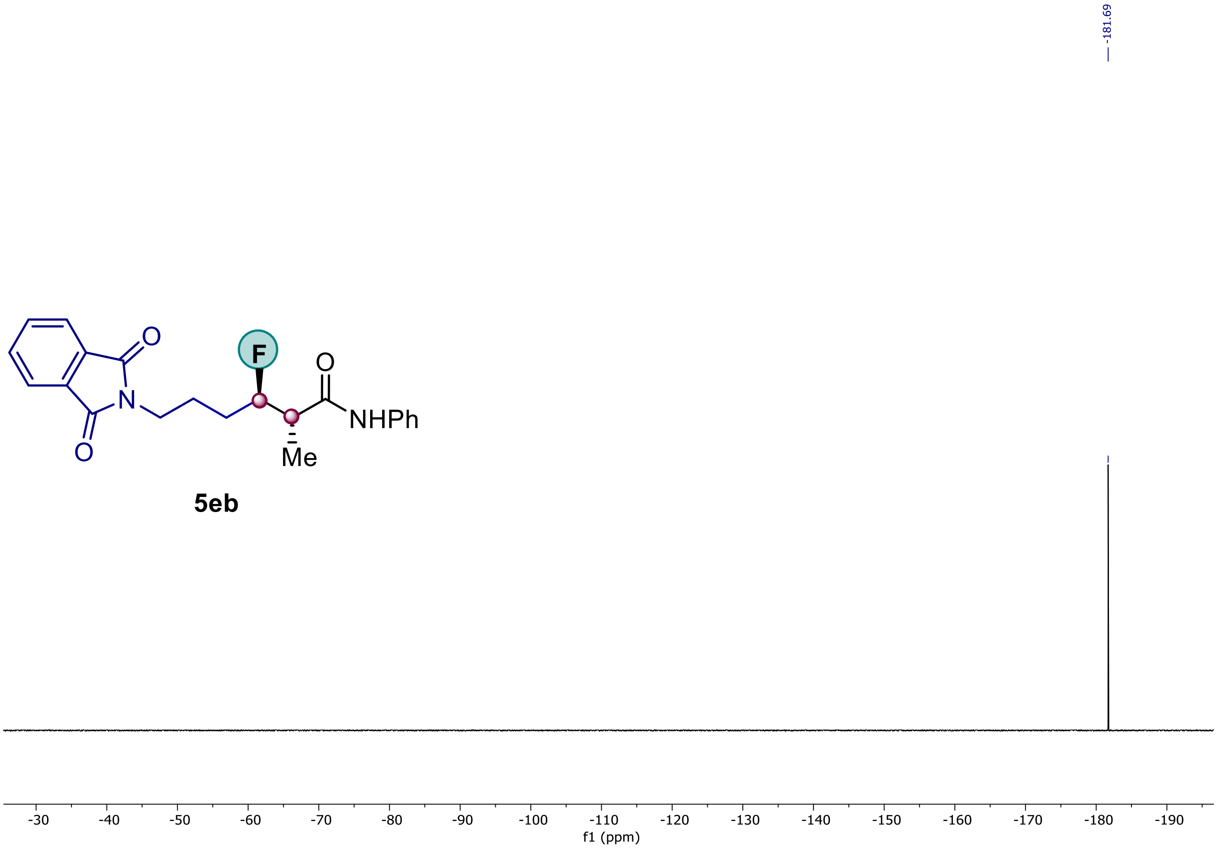

-181.69
-30
-40
-50
-60
-70
-80
-90
-100
-110
-120
-130
-140
-150
-160
-170
-180
-190
f1 (ppm)

5eb

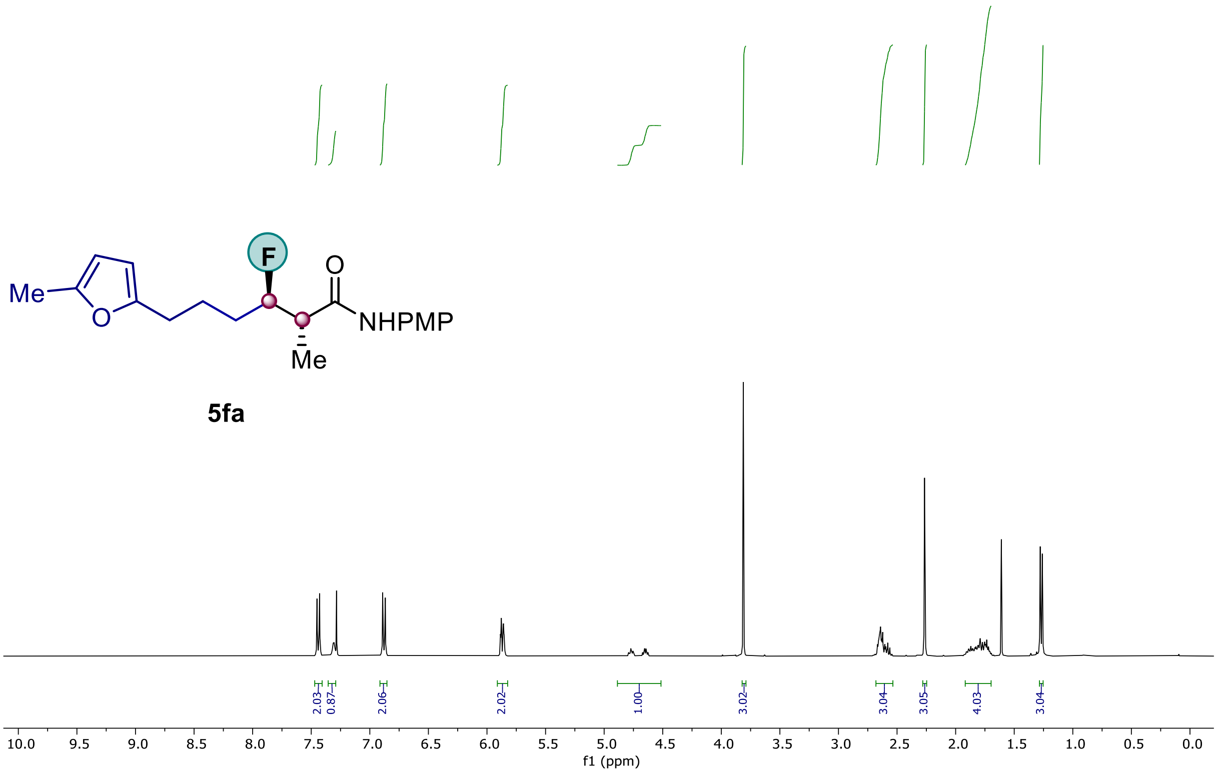

Me
O
F
O
NHPMP
Me
5fa
2.03
0.87
2.06
2.02
1.00
3.02
3.04
3.05
4.03
3.04
10.0 9.5 9.0 8.5 8.0 7.5 7.0 6.5 6.0 5.5 5.0 4.5 4.0 3.5 3.0 2.5 2.0 1.5 1.0 0.5 0.0
f1 (ppm)

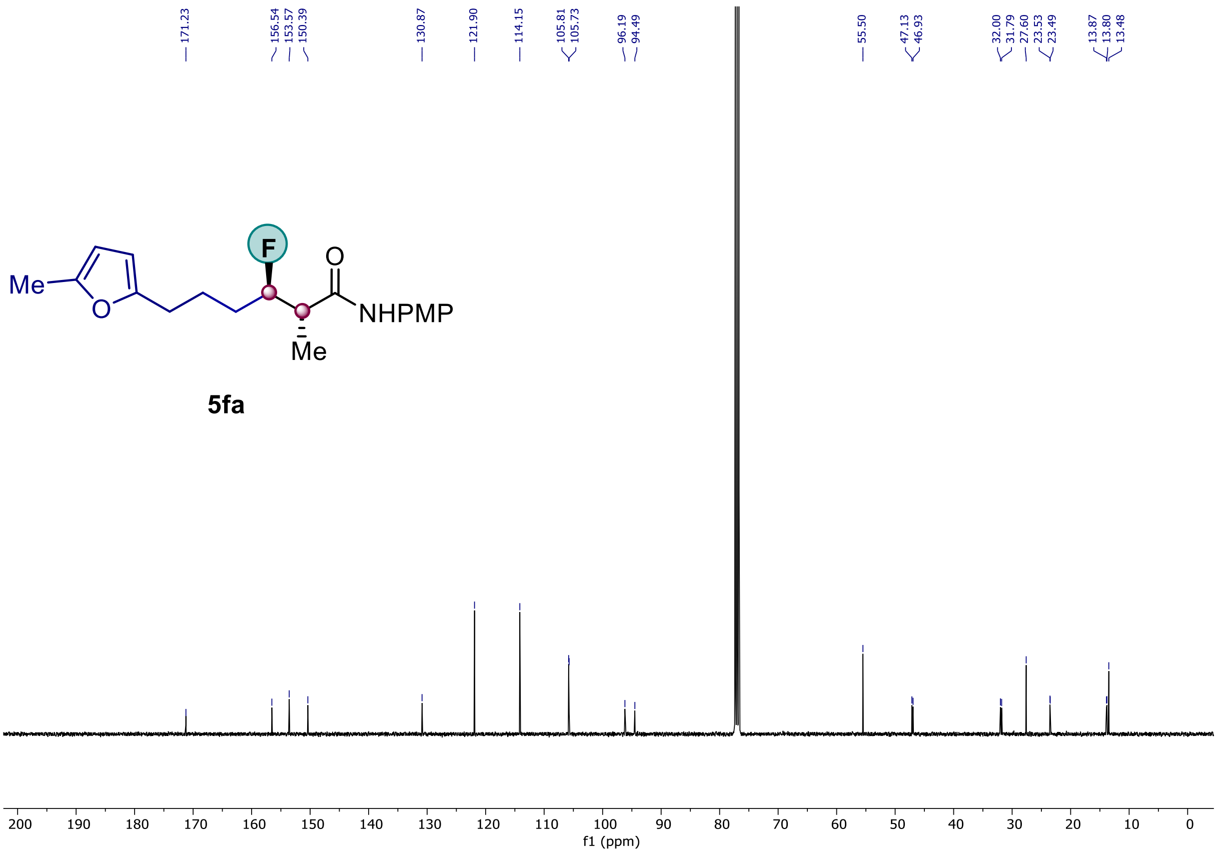

171.23
156.54
153.57
150.39
130.87
121.90
114.15
105.81
105.73
96.19
94.49
55.50
47.13
46.93
32.00
31.79
27.60
23.53
23.49
13.87
13.80
13.48
Me
O
F
O
NHPMP
Me
5fa
200 190 180 170 160 150 140 130 120 110 100 90 80 70 60 50 40 30 20 10 0
f1 (ppm)

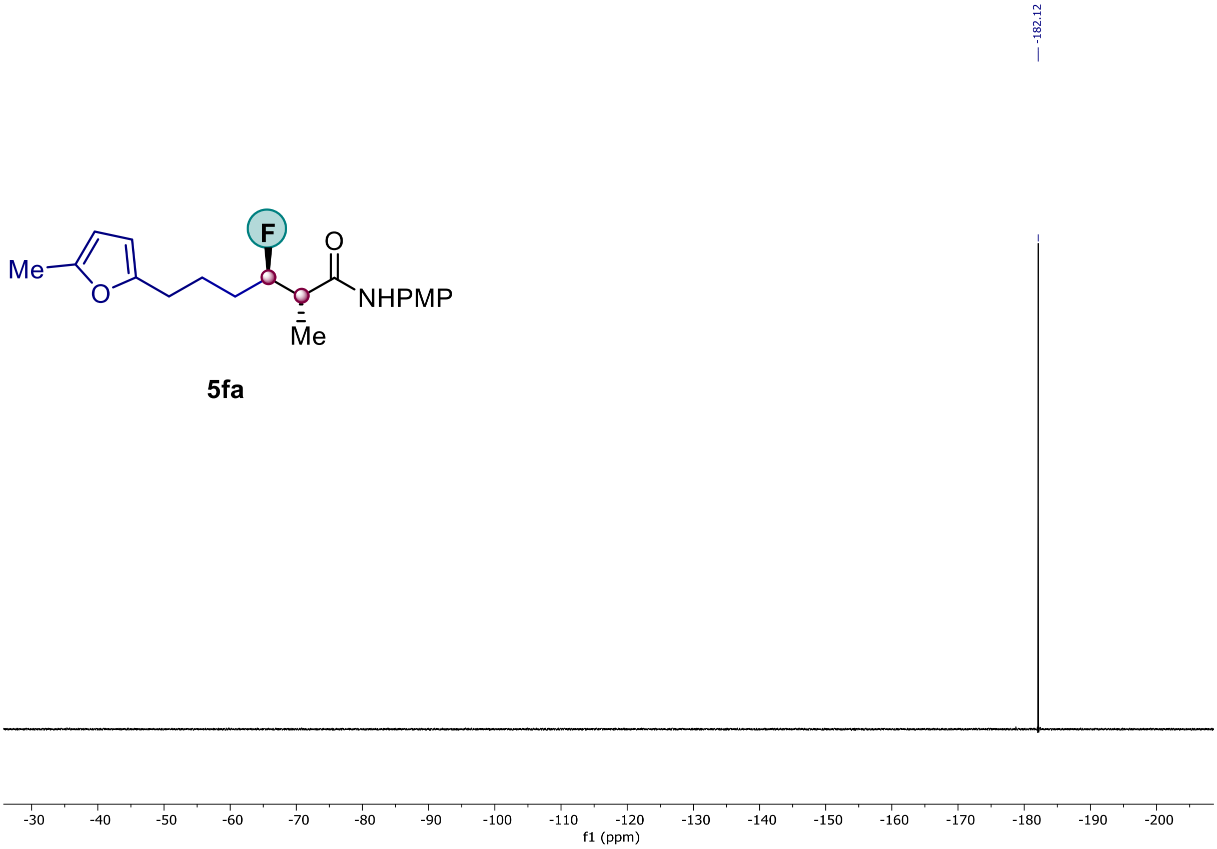
-182.12
Me
O
F
O
NHPMP
Me
5fa
-30
-40
-50
-60
-70
-80
-90
-100
-110
-120
-130
-140
-150
-160
-170
-180
-190
-200
f1 (ppm)

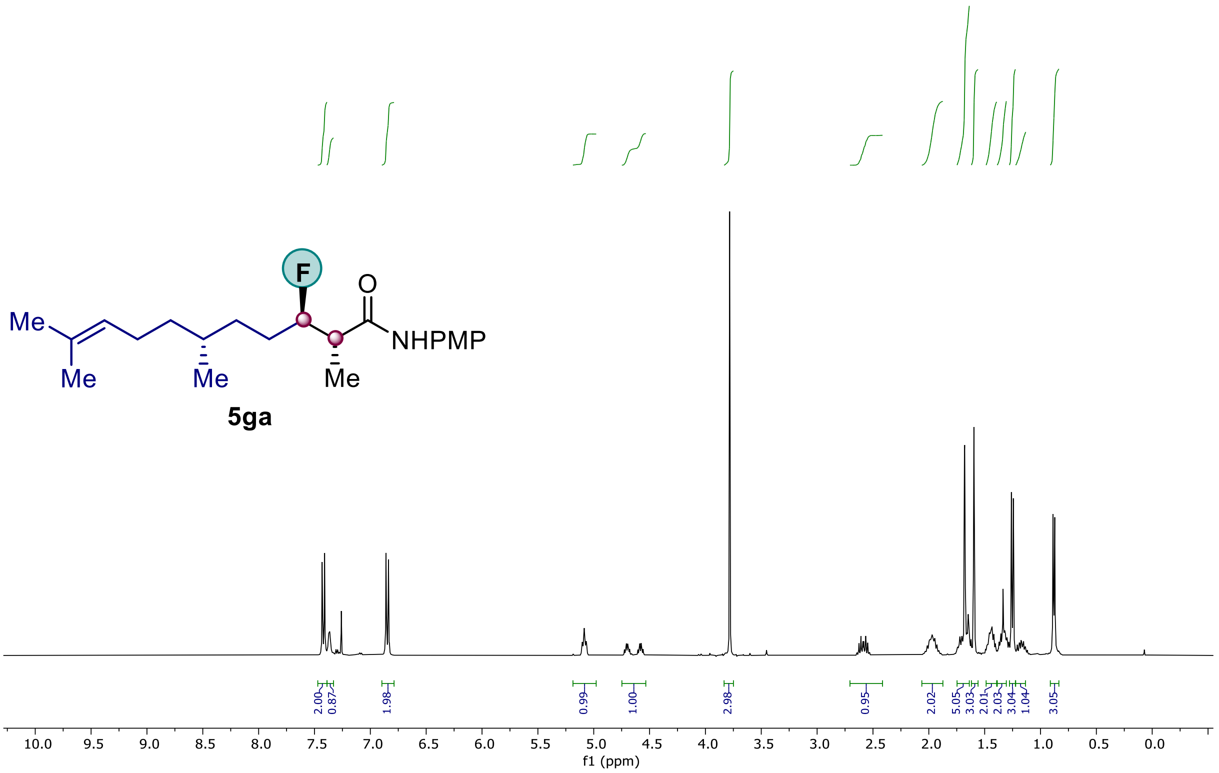

Me
Me
Me
Me
F
O
NHPMP
5ga
2.00
0.87
1.98
0.99
1.00
2.98
0.95
2.02
5.05
3.03
2.01
2.03
3.04
1.04
3.05
f1 (ppm)

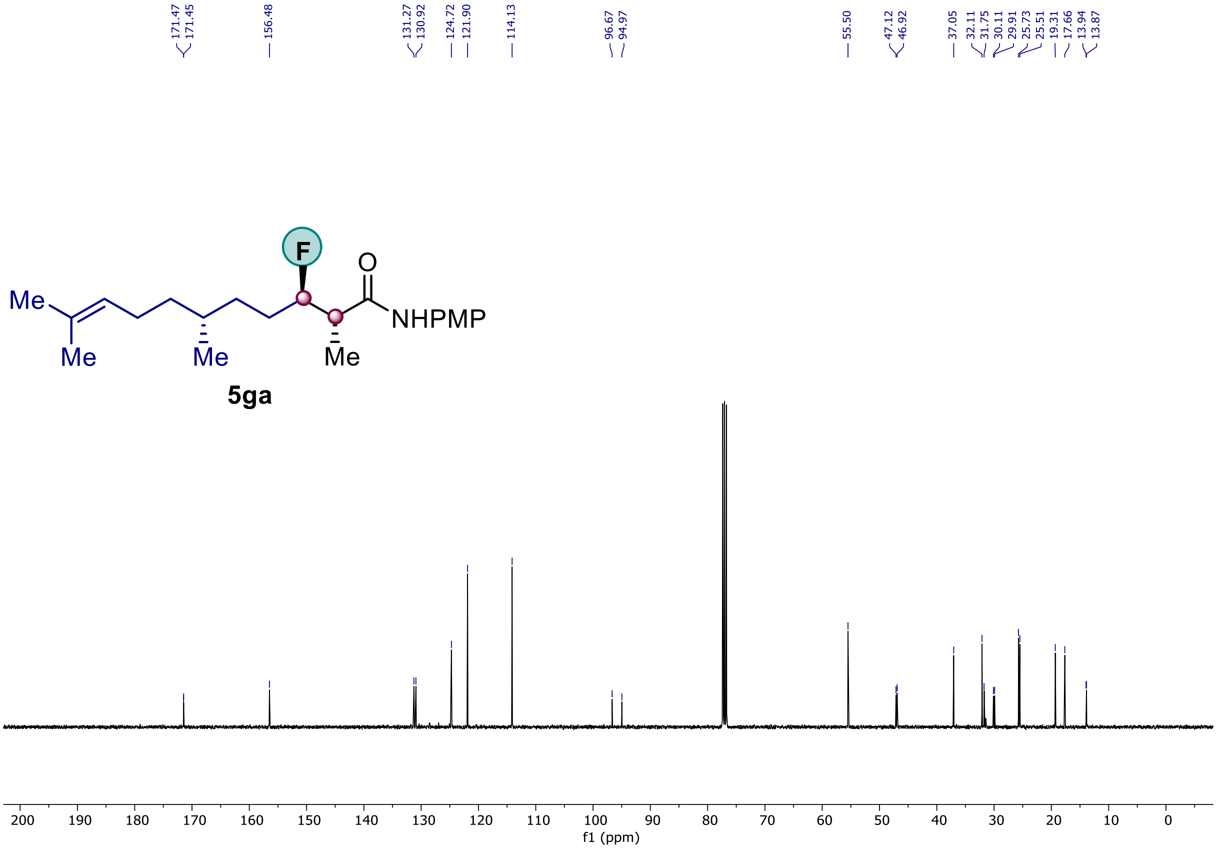

171.47
171.45
156.48
131.27
130.92
124.72
121.90
114.13
96.67
94.97
55.50
47.12
46.92
37.05
32.11
31.75
30.11
29.91
25.73
25.51
19.31
17.66
13.94
13.87
Me
Me
Me
Me
F
O
NHPMP
5ga
f1 (ppm)

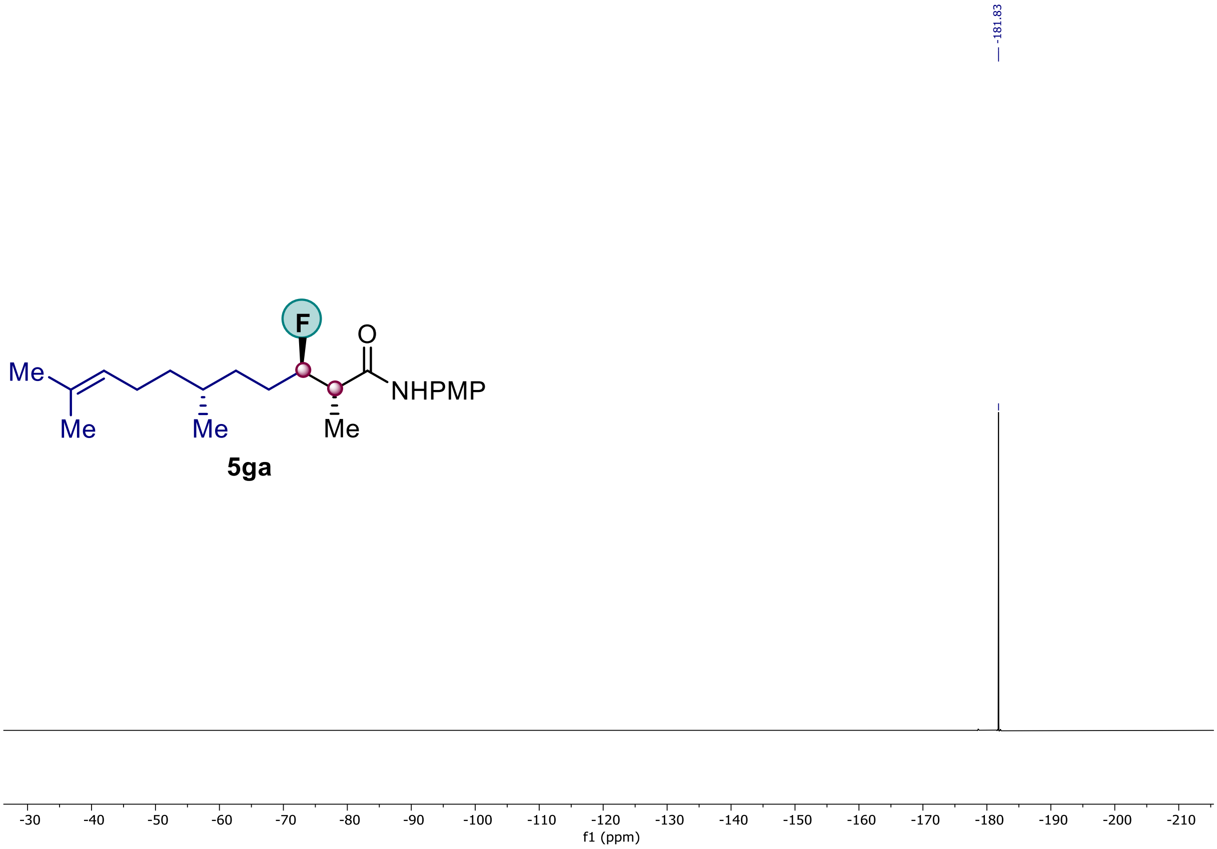
-181.83
Me
Me
Me
Me
F
O
NHPMP
-30
-40
-50
-60
-70
-80
-90
-100
-110
-120
-130
-140
-150
-160
-170
-180
-190
-200
-210
f1 (ppm)

5ga

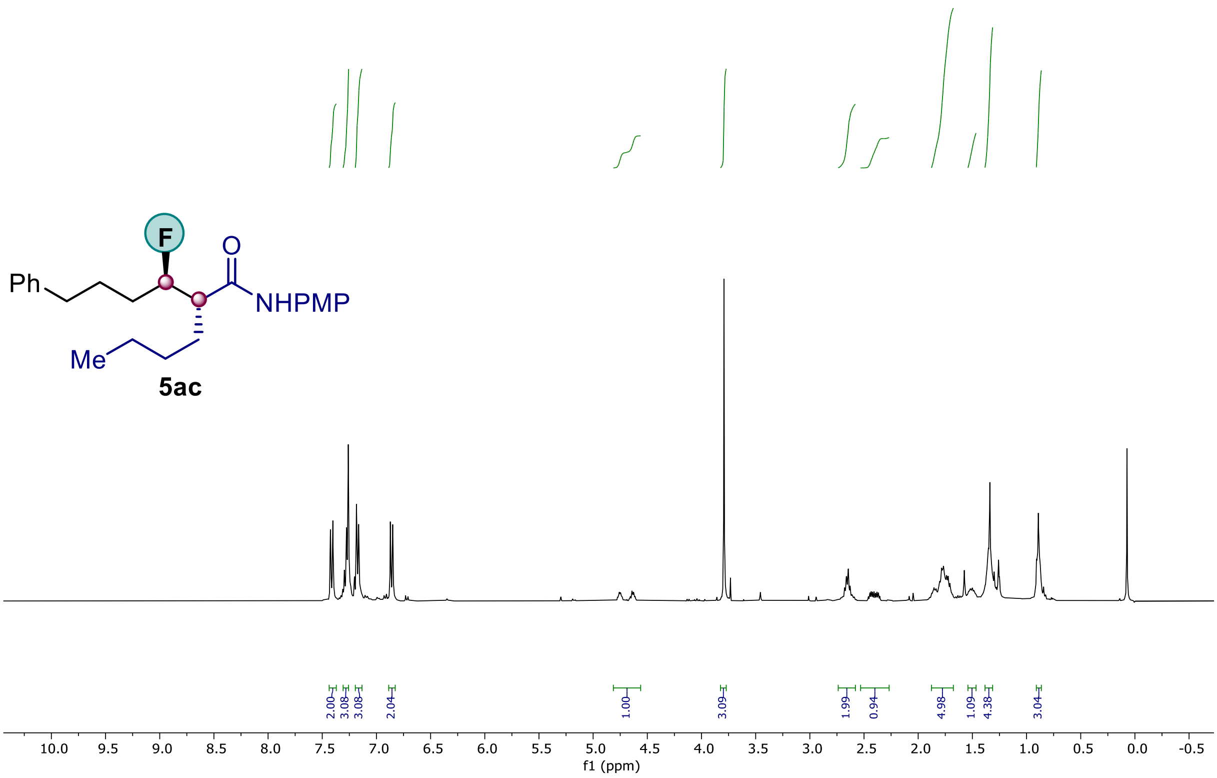
F
O
Ph
NHPMP
Me
5ac
2.00
3.08
3.08
2.04
1.00
3.09
1.99
0.94
4.98
1.09
4.38
3.04
10.0
9.5
9.0
8.5
8.0
7.5
7.0
6.5
6.0
5.5
5.0
4.5
4.0
3.5
3.0
2.5
2.0
1.5
1.0
0.5
0.0
-0.5
f1 (ppm)

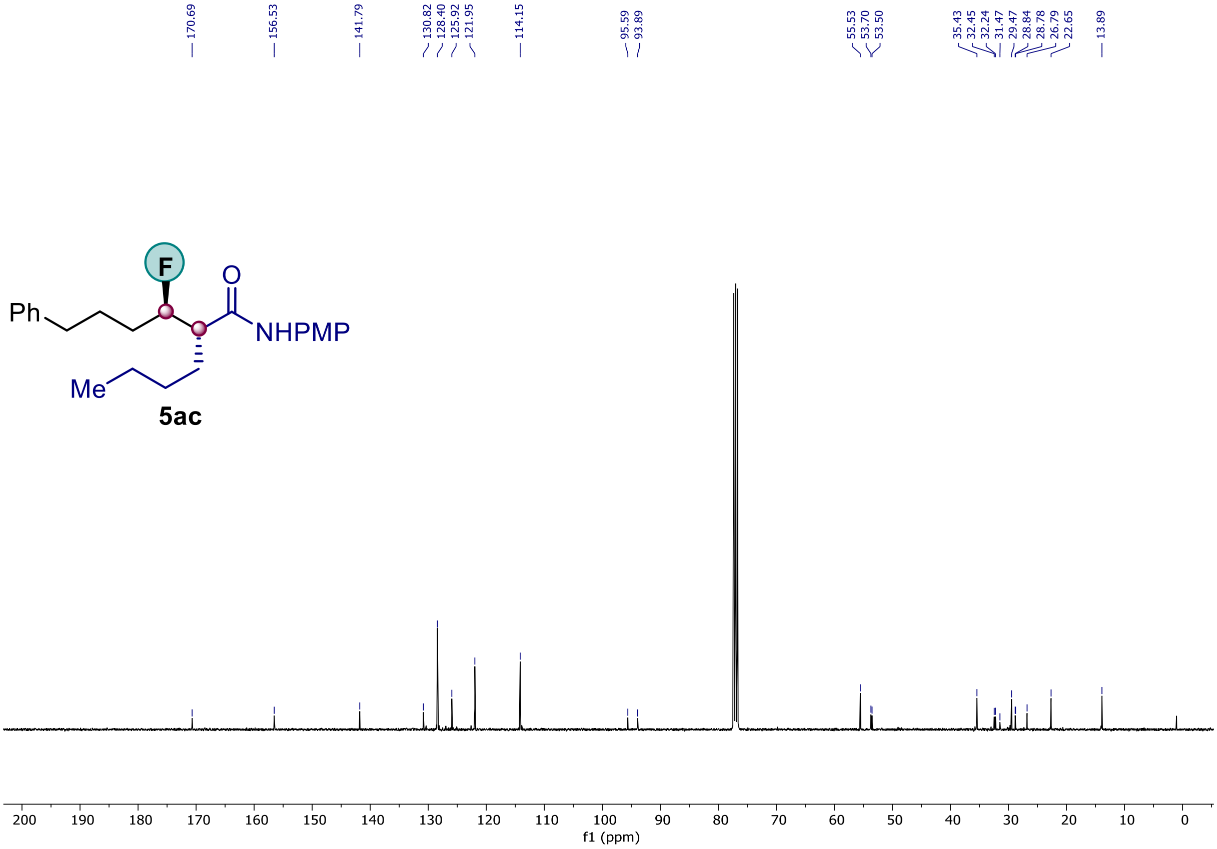
170.69
156.53
141.79
130.82
128.40
125.92
121.95
114.15
95.59
93.89
55.53
53.70
53.50
35.43
32.45
32.24
31.47
29.47
28.84
28.78
26.79
22.65
13.89
F
O
Ph
NHPMP
Me
5ac
200
190
180
170
160
150
140
130
120
110
100
90
80
70
60
50
40
30
20
10
0
f1 (ppm)

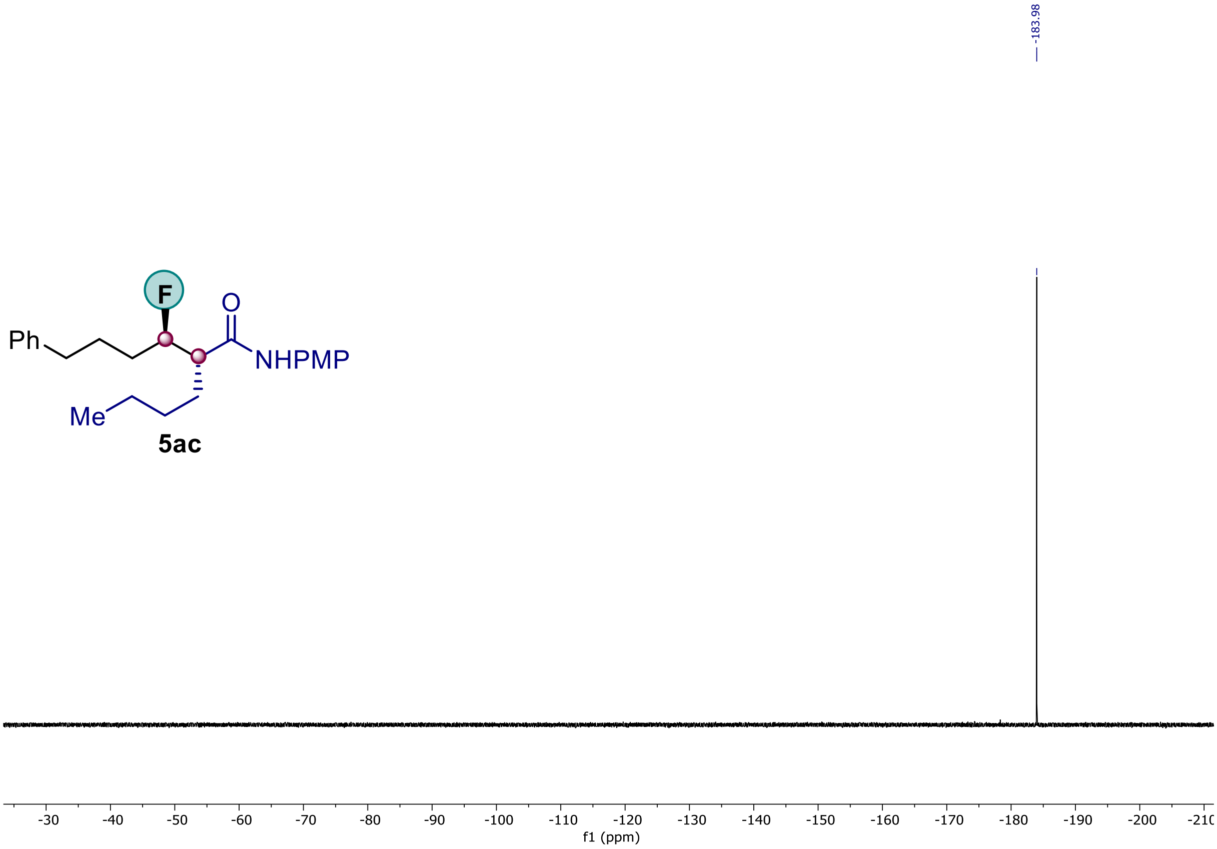

-183.98
F
O
Ph
NHPMP
Me
5ac
-30
-40
-50
-60
-70
-80
-90
-100
-110
-120
-130
-140
-150
-160
-170
-180
-190
-200
-210
f1 (ppm)

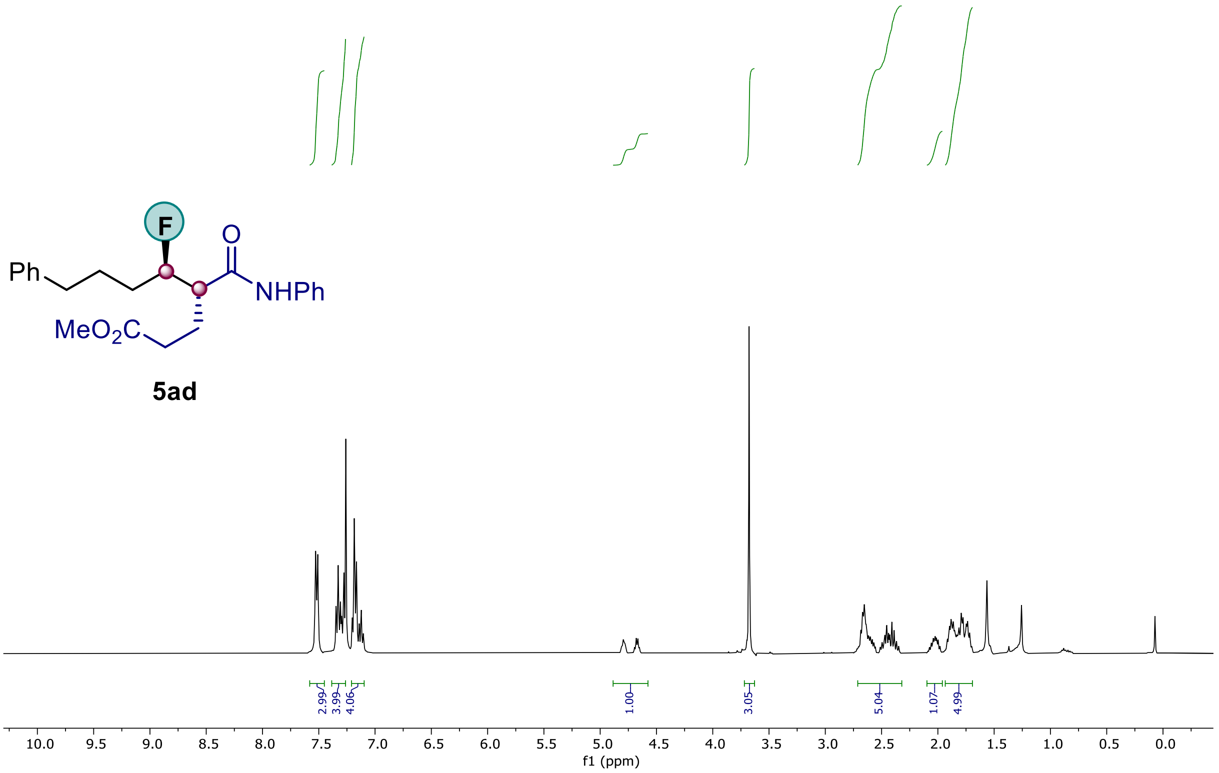
Ph
F
O
NHPh
MeO2C
5ad
2.99
3.99
4.06
1.00
3.05
5.04
1.07
4.99
10.0 9.5 9.0 8.5 8.0 7.5 7.0 6.5 6.0 5.5 5.0 4.5 4.0 3.5 3.0 2.5 2.0 1.5 1.0 0.5 0.0
f1 (ppm)

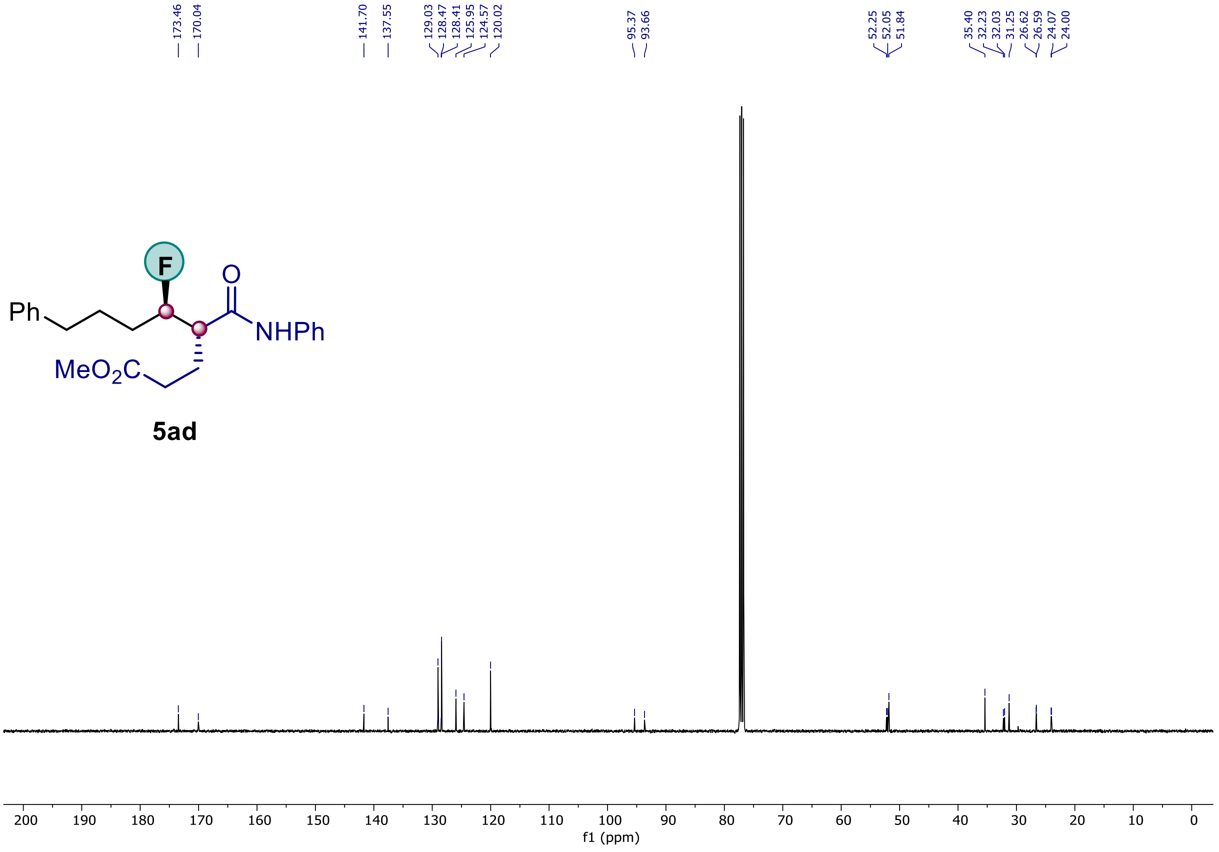
173.46
170.04
141.70
137.55
129.03
128.47
128.41
125.95
124.57
120.02
95.37
93.66
52.25
52.05
51.84
35.40
32.23
32.03
31.25
26.62
26.59
24.07
24.00
Ph
F
O
NHPh
MeO2C
5ad
200 190 180 170 160 150 140 130 120 110 100 90 80 70 60 50 40 30 20 10 0
f1 (ppm)

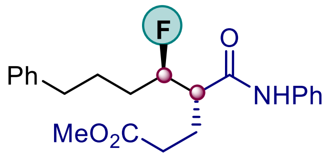
F
O
Ph
NHPh
MeO2C

5ad

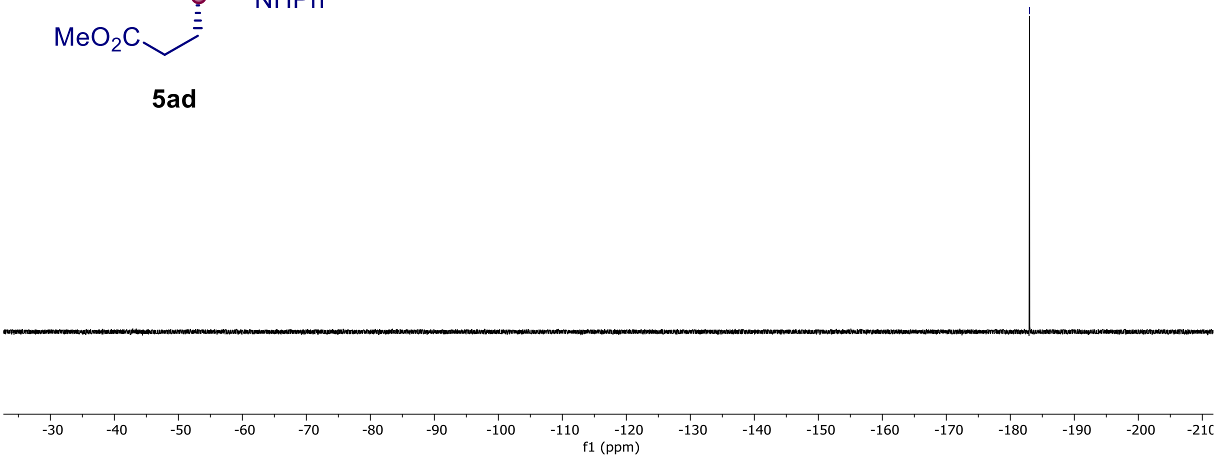
-183.00
-30
-40
-50
-60
-70
-80
-90
-100
-110
-120
-130
-140
-150
-160
-170
-180
-190
-200
-210
f1 (ppm)

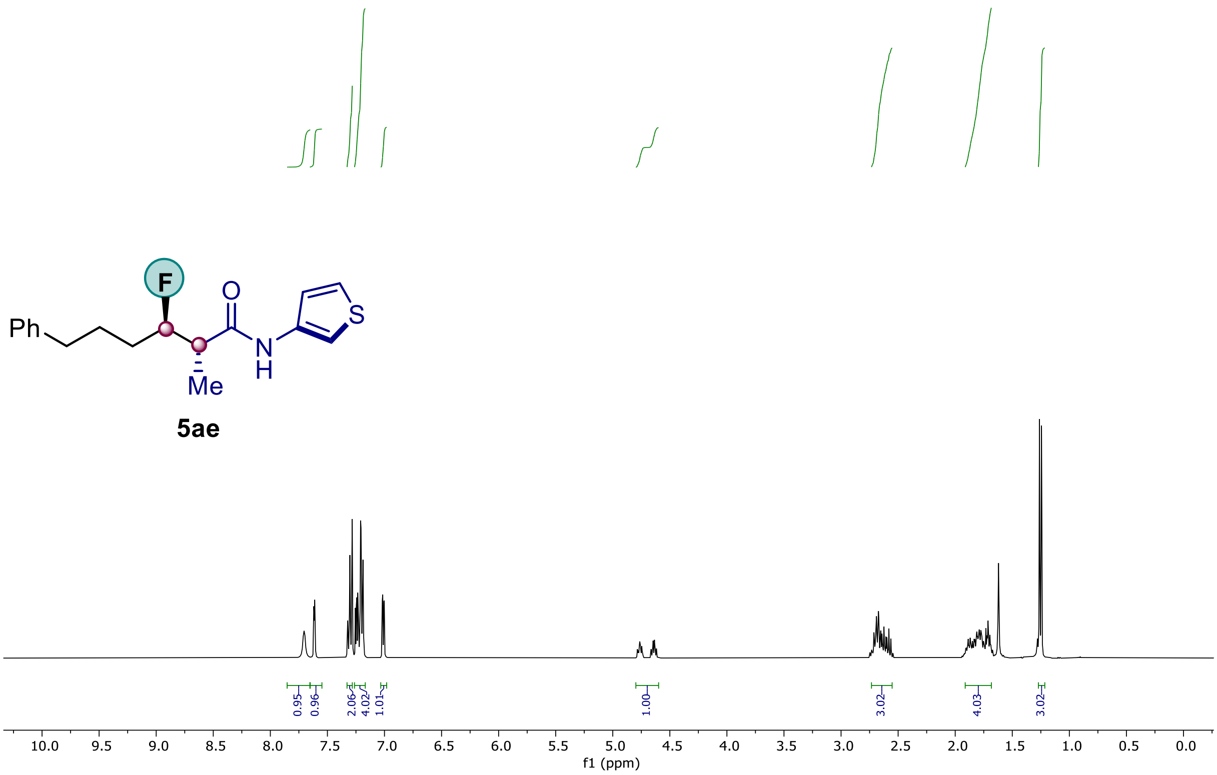
Ph
F
O
N
H
S
Me
5ae
0.95
0.96
2.06
4.02
1.01
1.00
3.02
4.03
3.02
10.0 9.5 9.0 8.5 8.0 7.5 7.0 6.5 6.0 5.5 5.0 4.5 4.0 3.5 3.0 2.5 2.0 1.5 1.0 0.5 0.0
f1 (ppm)

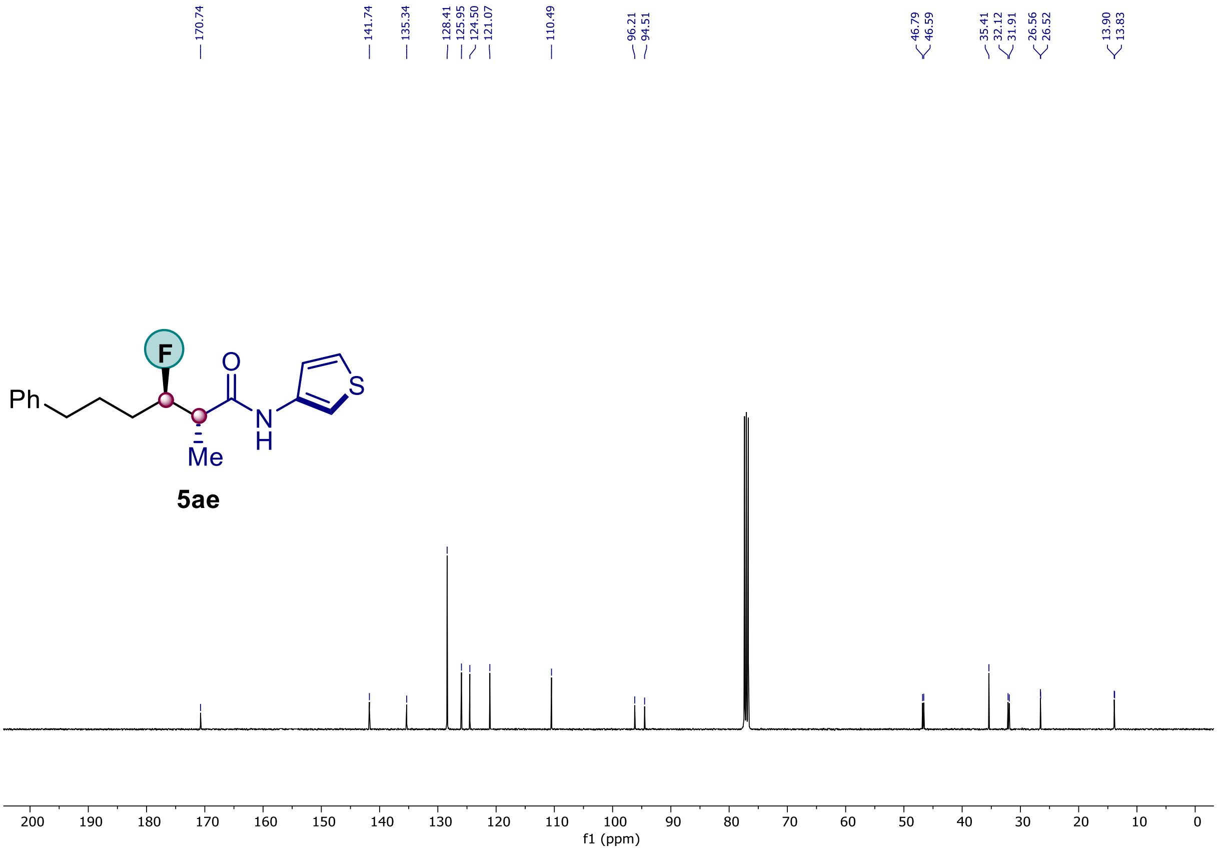
170.74
141.74
135.34
128.41
125.95
124.50
121.07
110.49
96.21
94.51
46.79
46.59
35.41
32.12
31.91
26.56
26.52
13.90
13.83
Ph
F
O
N
H
S
Me
5ae
200 190 180 170 160 150 140 130 120 110 100 90 80 70 60 50 40 30 20 10 0
f1 (ppm)

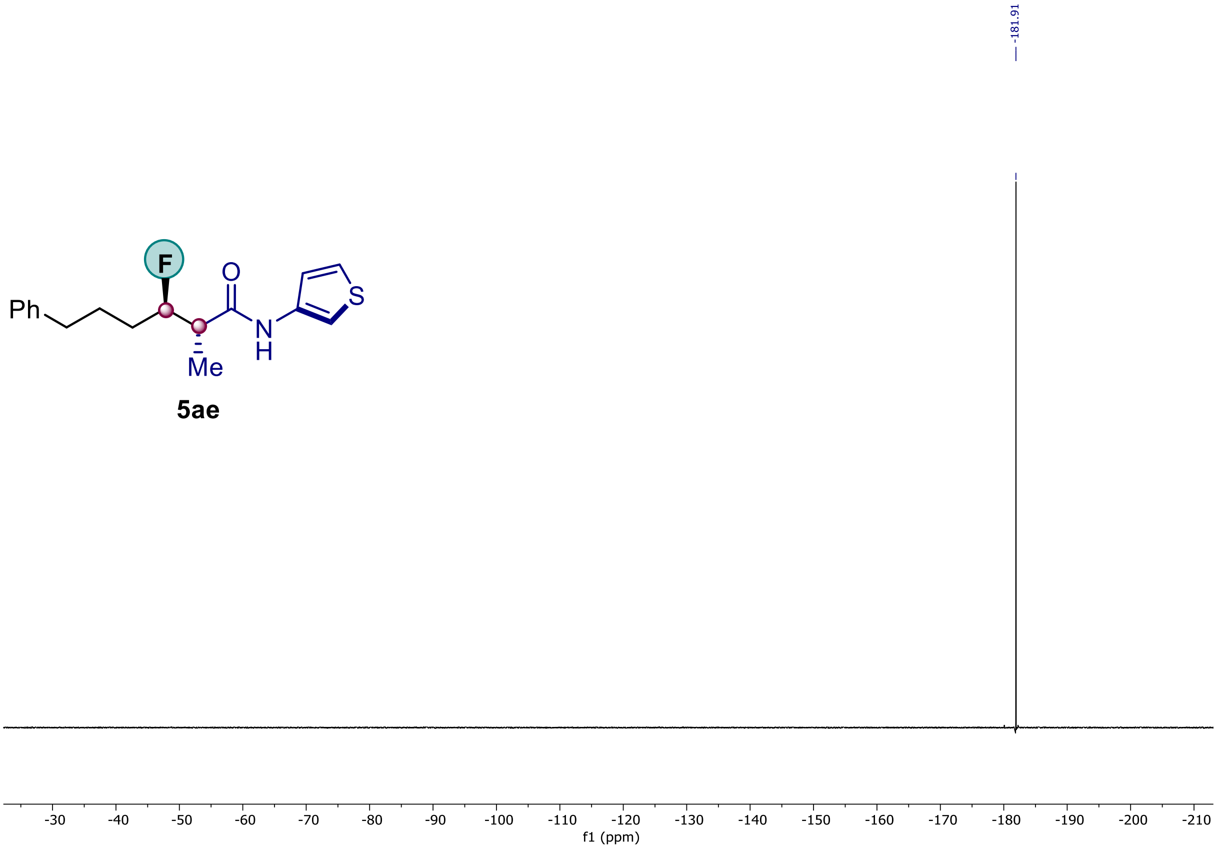
-181.91
F
O
Ph
N
H
S
Me
5ae
-30 -40 -50 -60 -70 -80 -90 -100 -110 -120 -130 -140 -150 -160 -170 -180 -190 -200 -210
f1 (ppm)

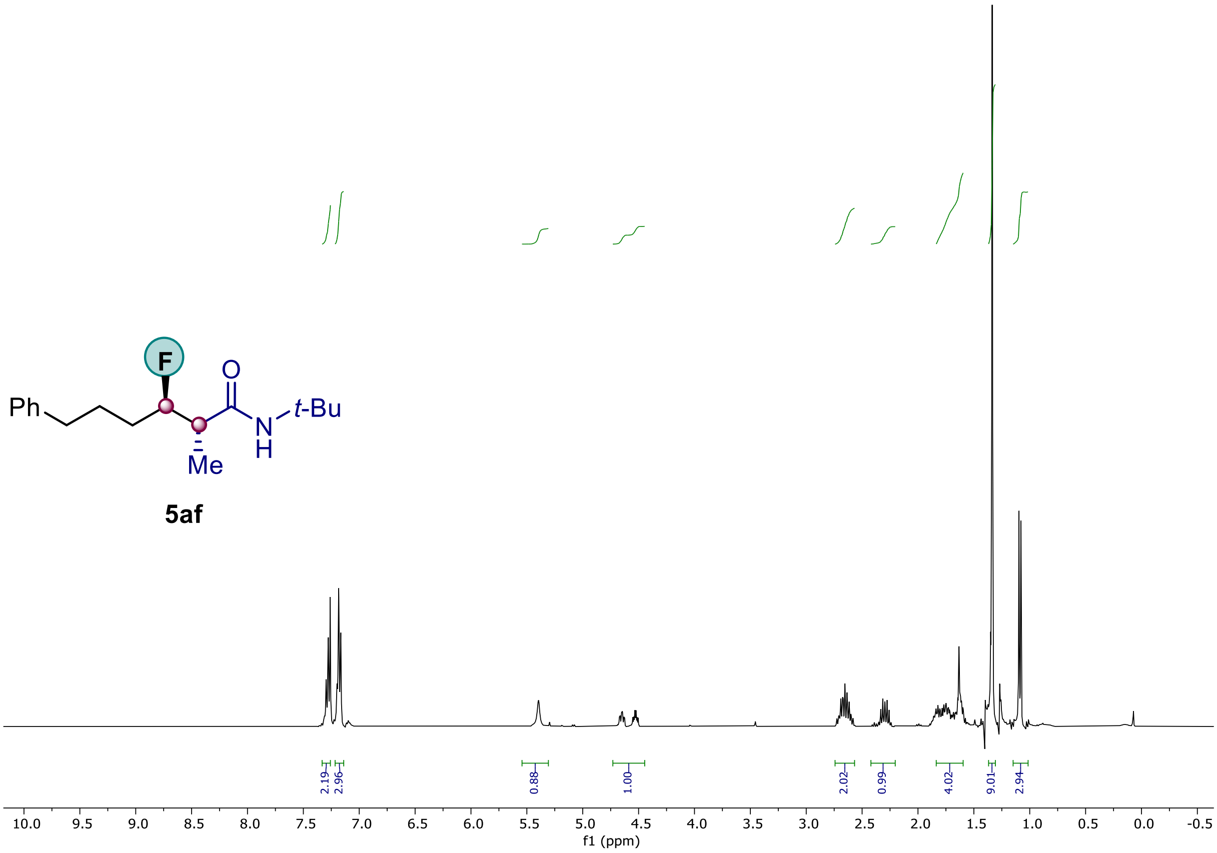
Ph
O
N
t-Bu
H
Me
F
2.19
2.96
0.88
1.00
2.02
0.99
4.02
9.01
2.94
f1 (ppm)

5af

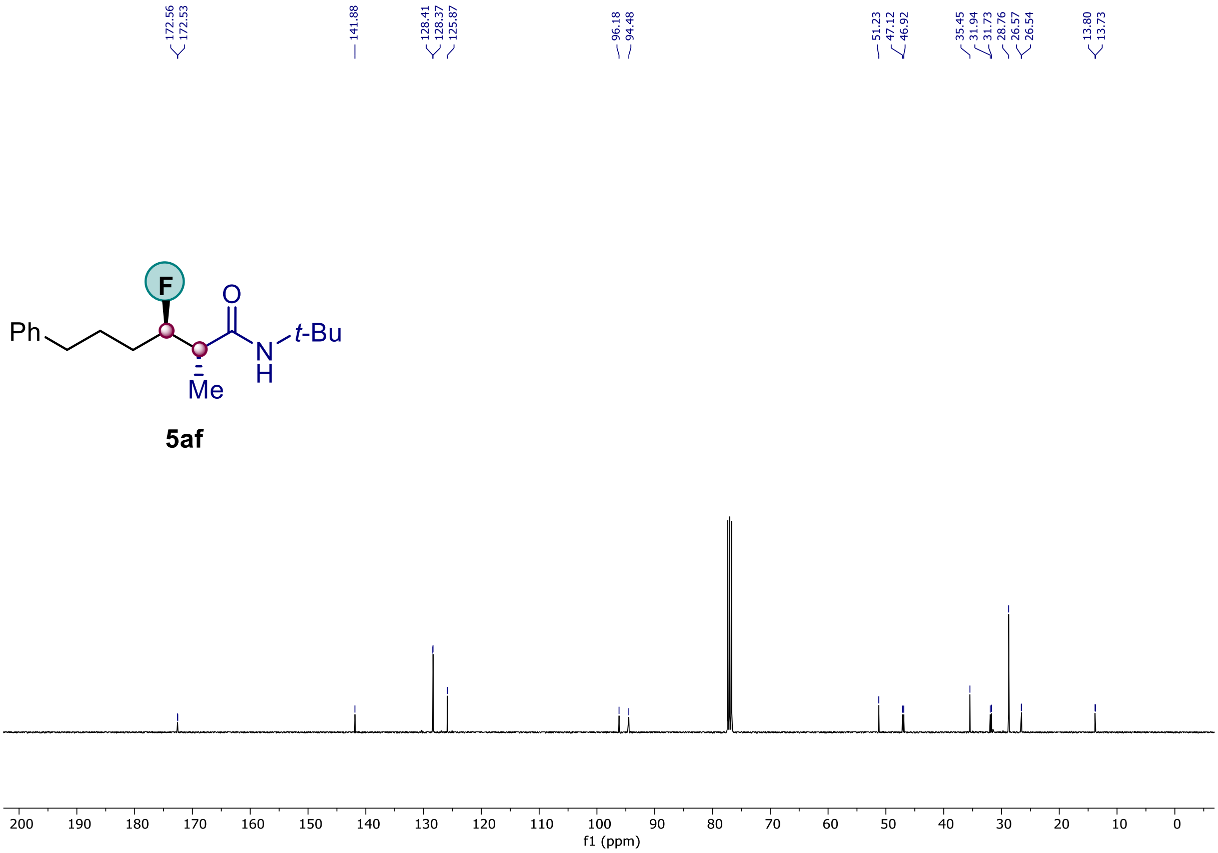
172.56
172.53
141.88
128.41
128.37
125.87
96.18
94.48
51.23
47.12
46.92
35.45
31.94
31.73
28.76
26.57
26.54
13.80
13.73
Ph
O
N
t-Bu
H
Me
F
f1 (ppm)

5af

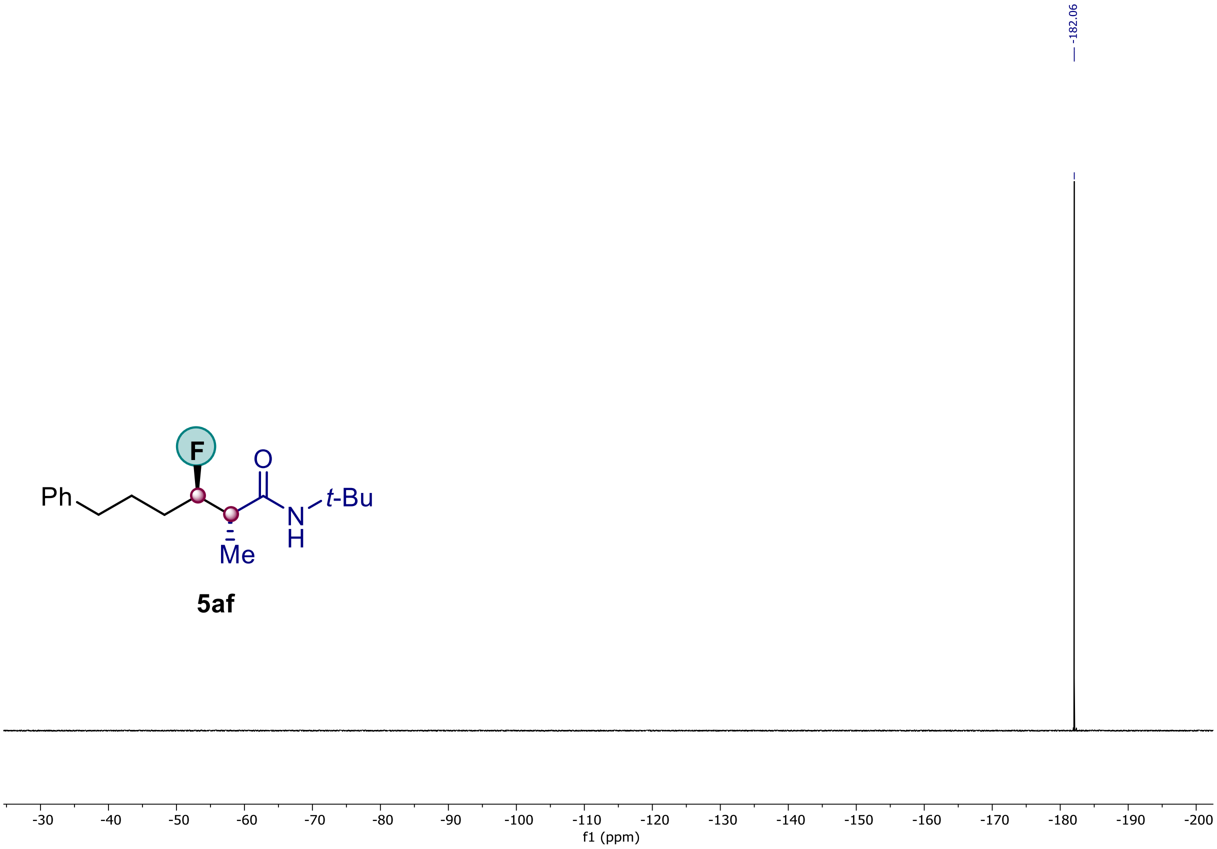
-182.06
Ph
F
O
N
t-Bu
H
Me
5af
-30
-40
-50
-60
-70
-80
-90
-100
-110
-120
-130
-140
-150
-160
-170
-180
-190
-200
f1 (ppm)

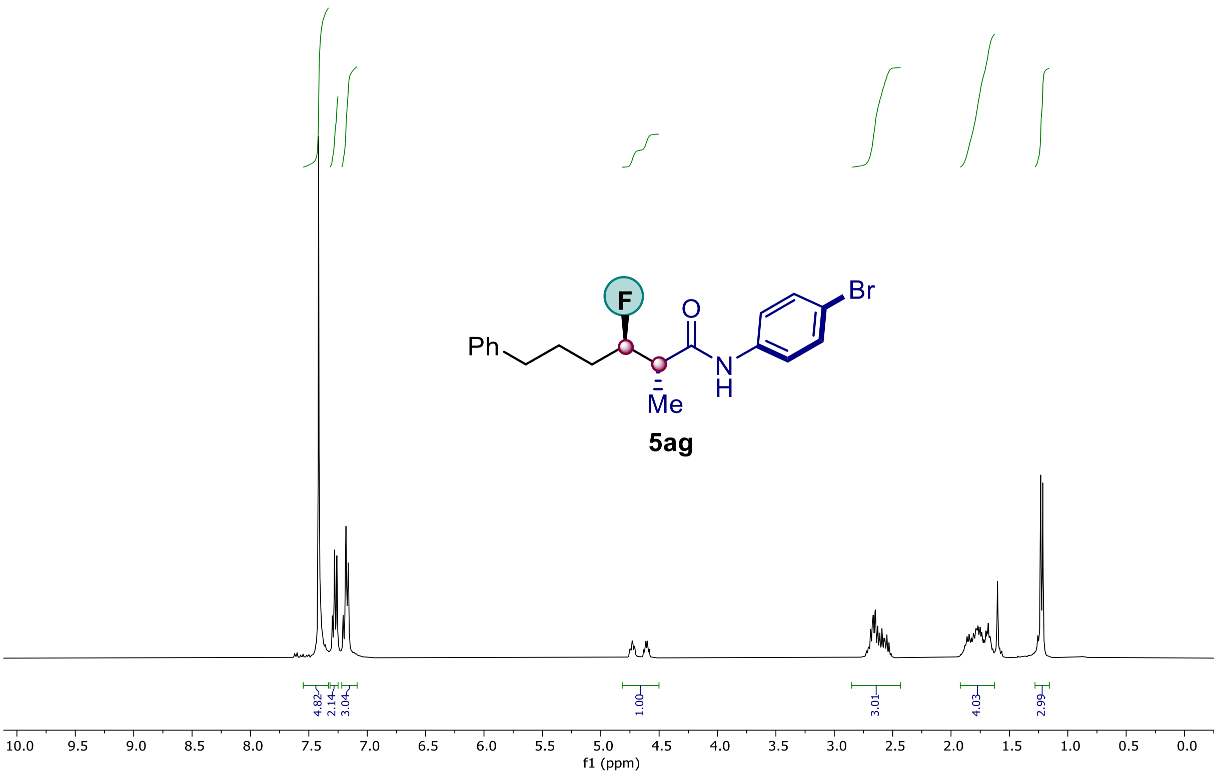

Ph
F
O
N
H
Br
Me
5ag
4.82
2.14
3.04
1.00
3.01
4.03
2.99
10.0 9.5 9.0 8.5 8.0 7.5 7.0 6.5 6.0 5.5 5.0 4.5 4.0 3.5 3.0 2.5 2.0 1.5 1.0 0.5 0.0
f1 (ppm)

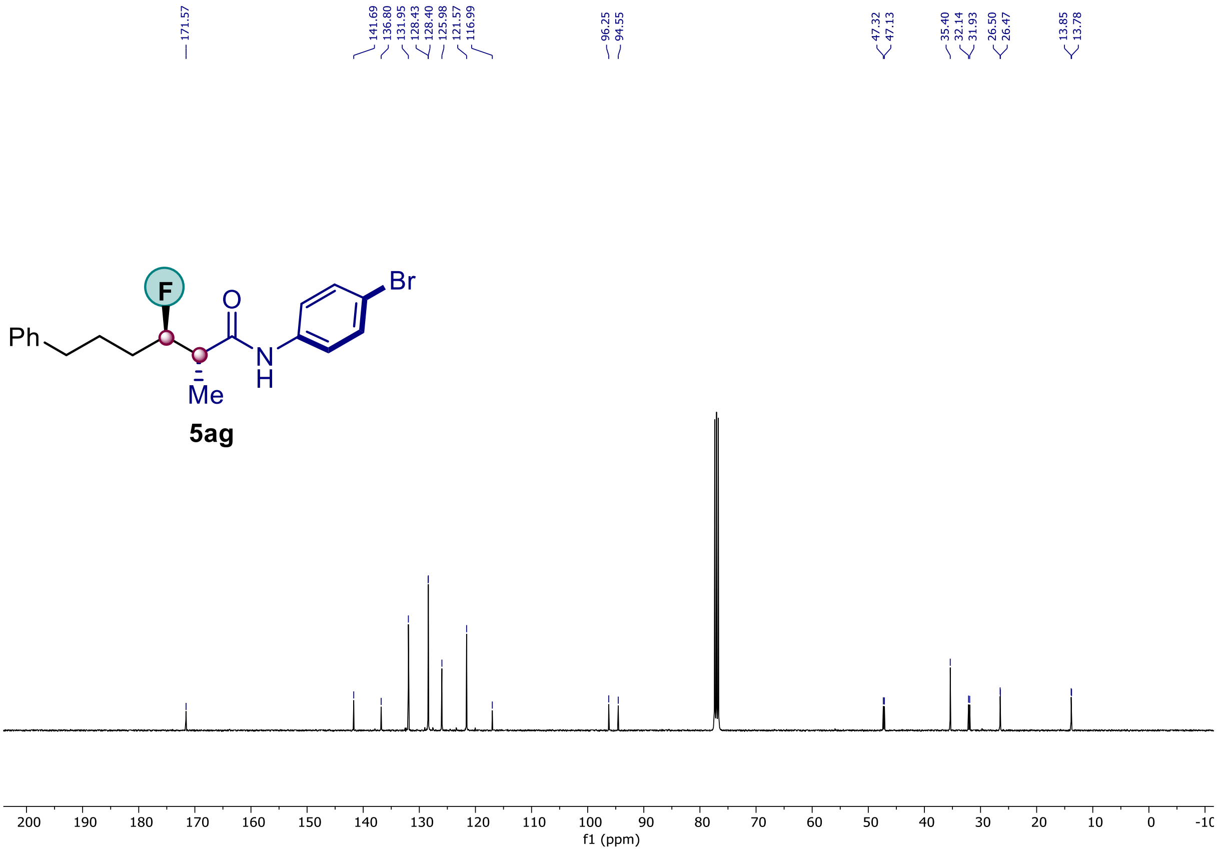

171.57
141.69
136.80
131.95
128.43
128.40
125.98
121.57
116.99
96.25
94.55
47.32
47.13
35.40
32.14
31.93
26.50
26.47
13.85
13.78
Ph
F
O
N
H
Br
Me
5ag
200 190 180 170 160 150 140 130 120 110 100 90 80 70 60 50 40 30 20 10 0
f1 (ppm)

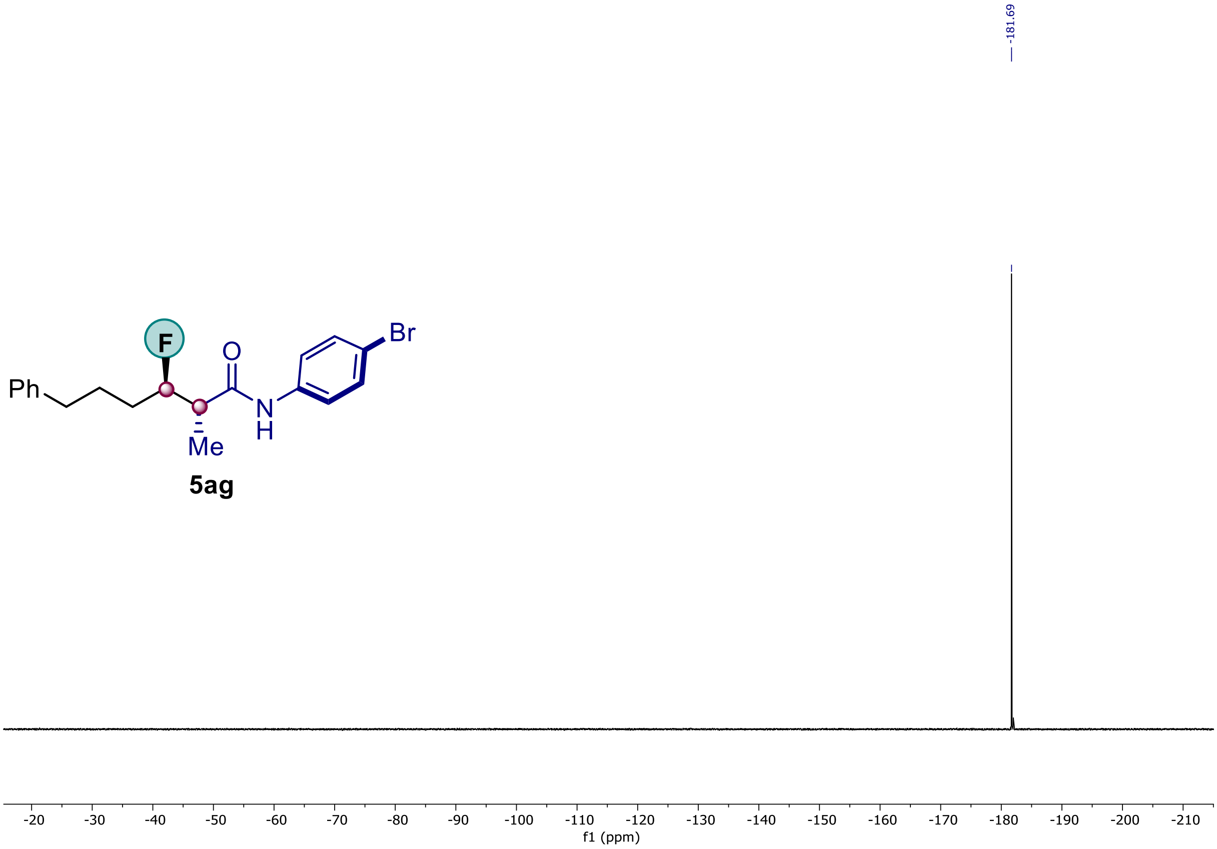
Ph
F
O
N
H
Br
Me

5ag

-181.69
-20
-30
-40
-50
-60
-70
-80
-90
-100
-110
-120
-130
-140
-150
-160
-170
-180
-190
-200
-210
f1 (ppm)

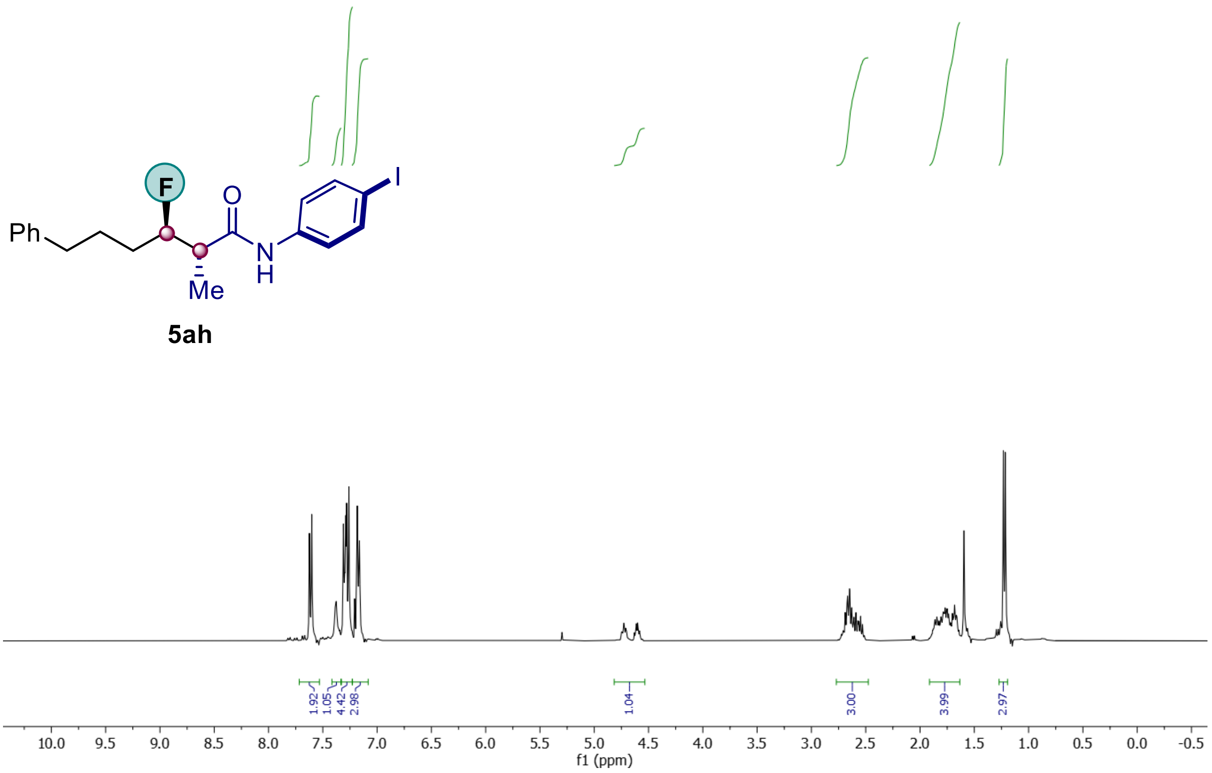
5ah
1.92
1.05
4.42
2.98
1.04
3.00
3.99
2.97
f1 (ppm)

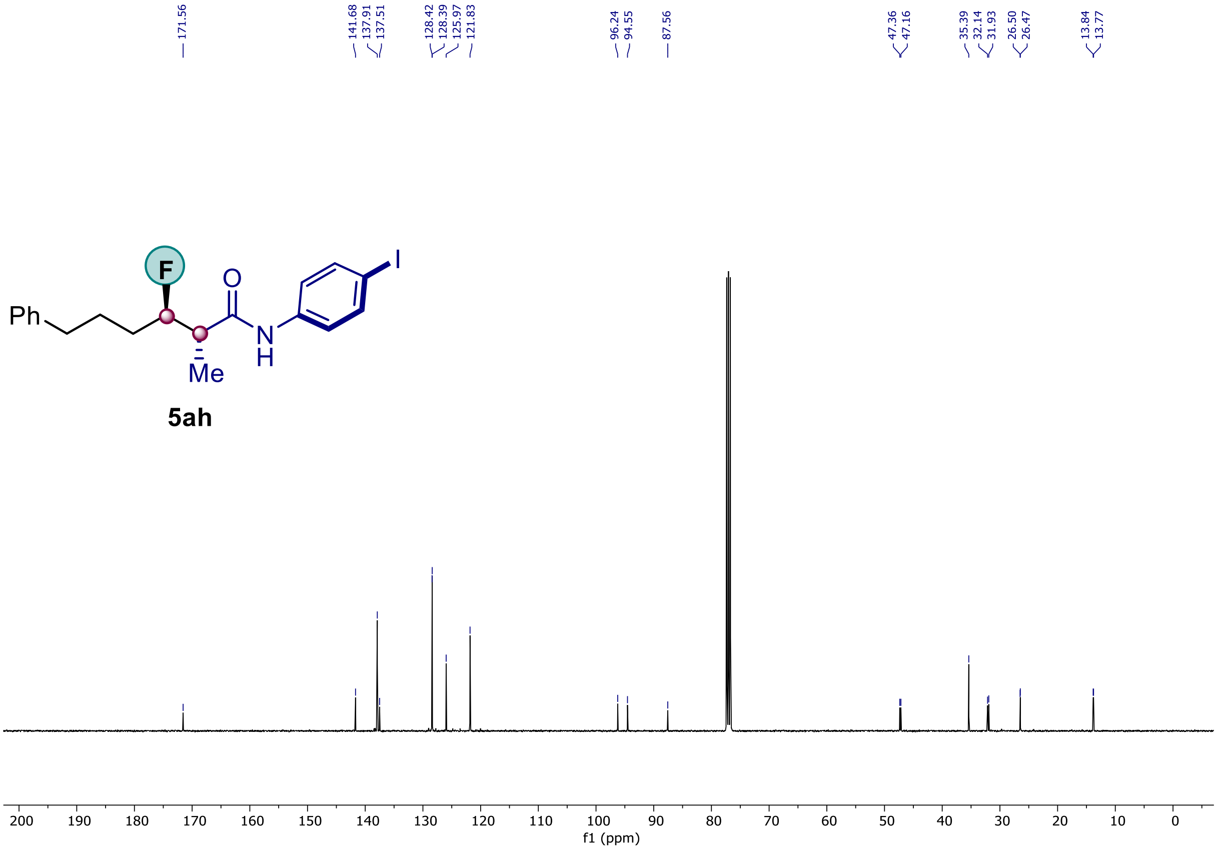
5ah
171.56
141.68
137.91
137.51
128.42
128.39
125.97
121.83
96.24
94.55
87.56
47.36
47.16
35.39
32.14
31.93
26.50
26.47
13.84
13.77
f1 (ppm)

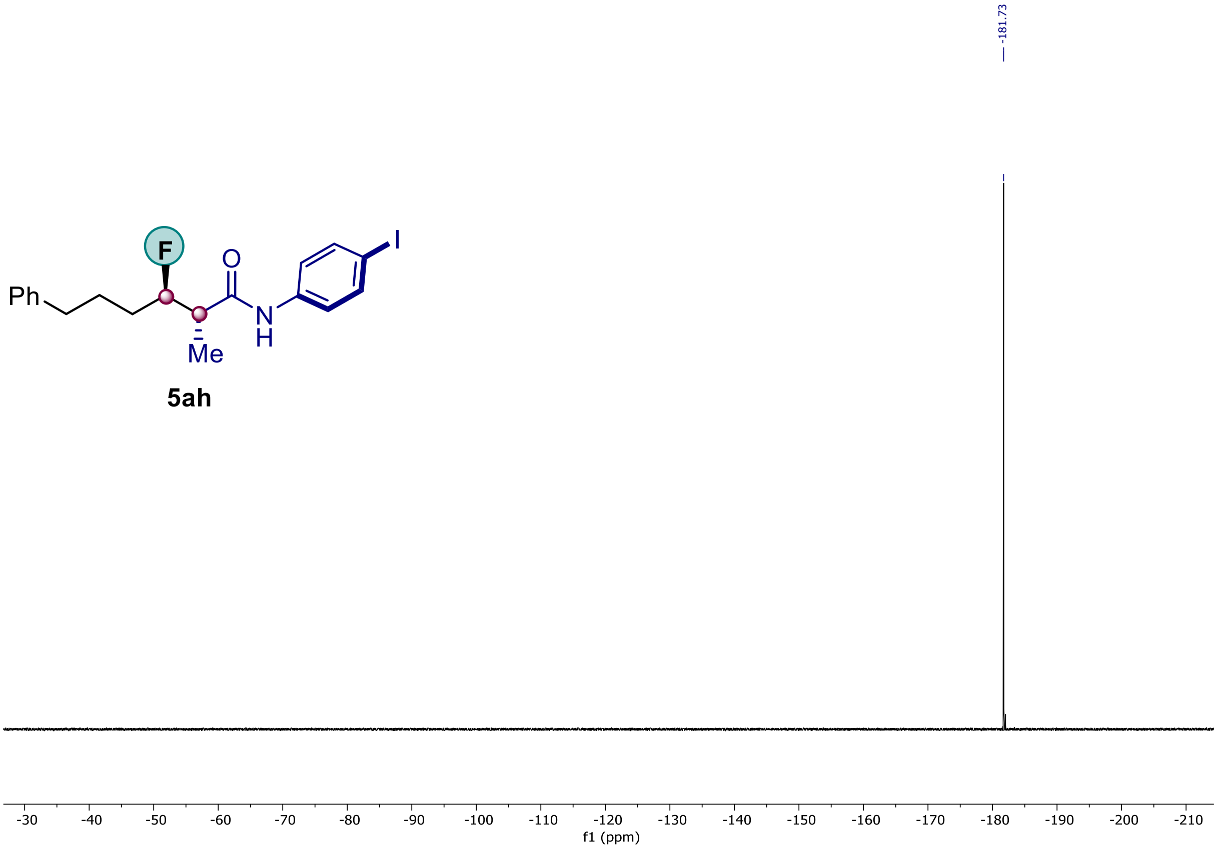
-181.73
F
O
I
Ph
N
H
Me
5ah
-30
-40
-50
-60
-70
-80
-90
-100
-110
-120
-130
-140
-150
-160
-170
-180
-190
-200
-210
f1 (ppm)

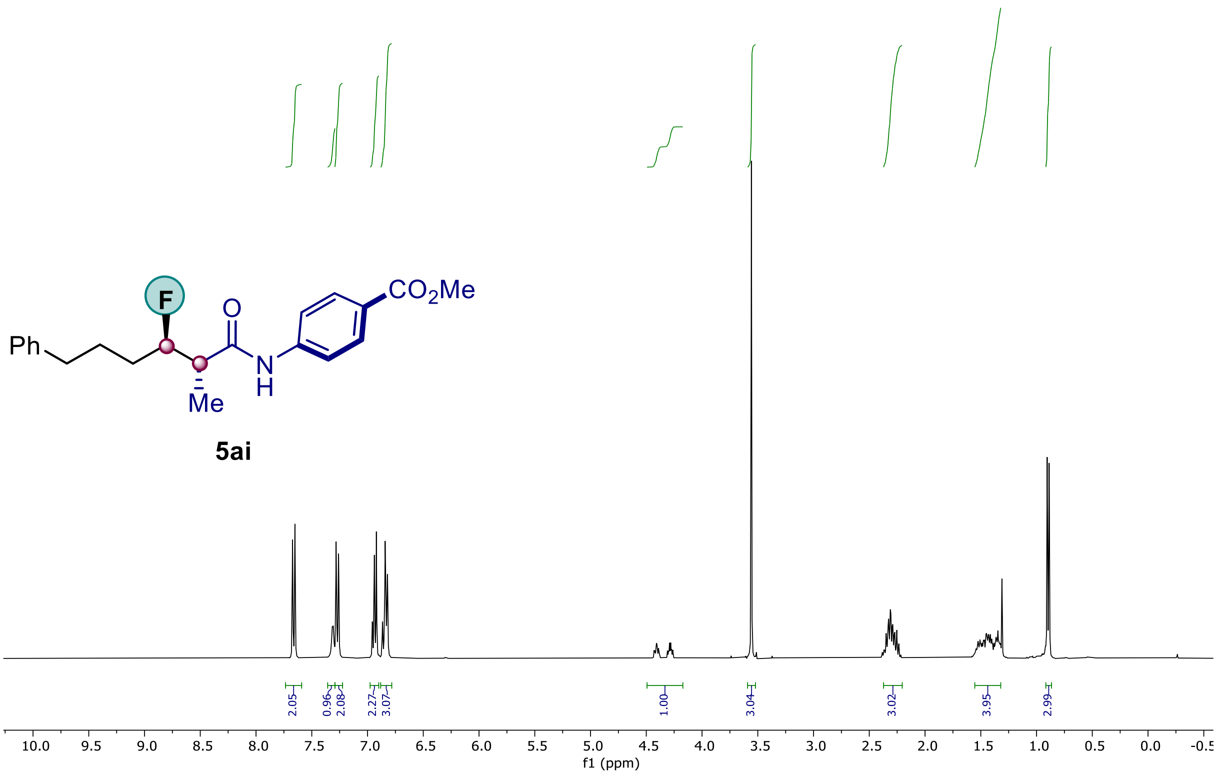
CO2Me
O
Ph
N
H
Me
F
5ai
2.05
0.96
2.08
2.27
3.07
1.00
3.04
3.02
3.95
2.99
f1 (ppm)

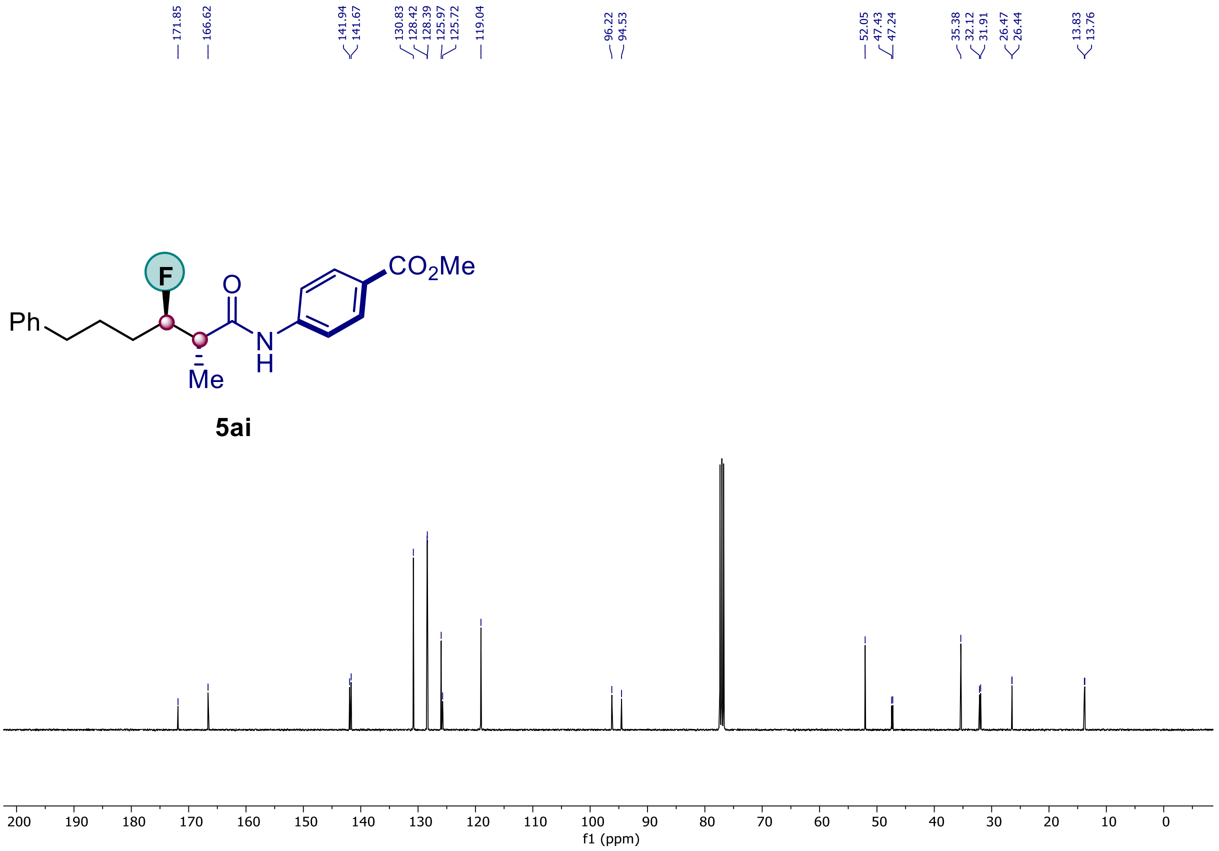
171.85
166.62
141.94
141.67
130.83
128.42
128.39
125.97
125.72
119.04
96.22
94.53
52.05
47.43
47.24
35.38
32.12
31.91
26.47
26.44
13.83
13.76
CO2Me
O
Ph
N
H
Me
F
5ai
f1 (ppm)

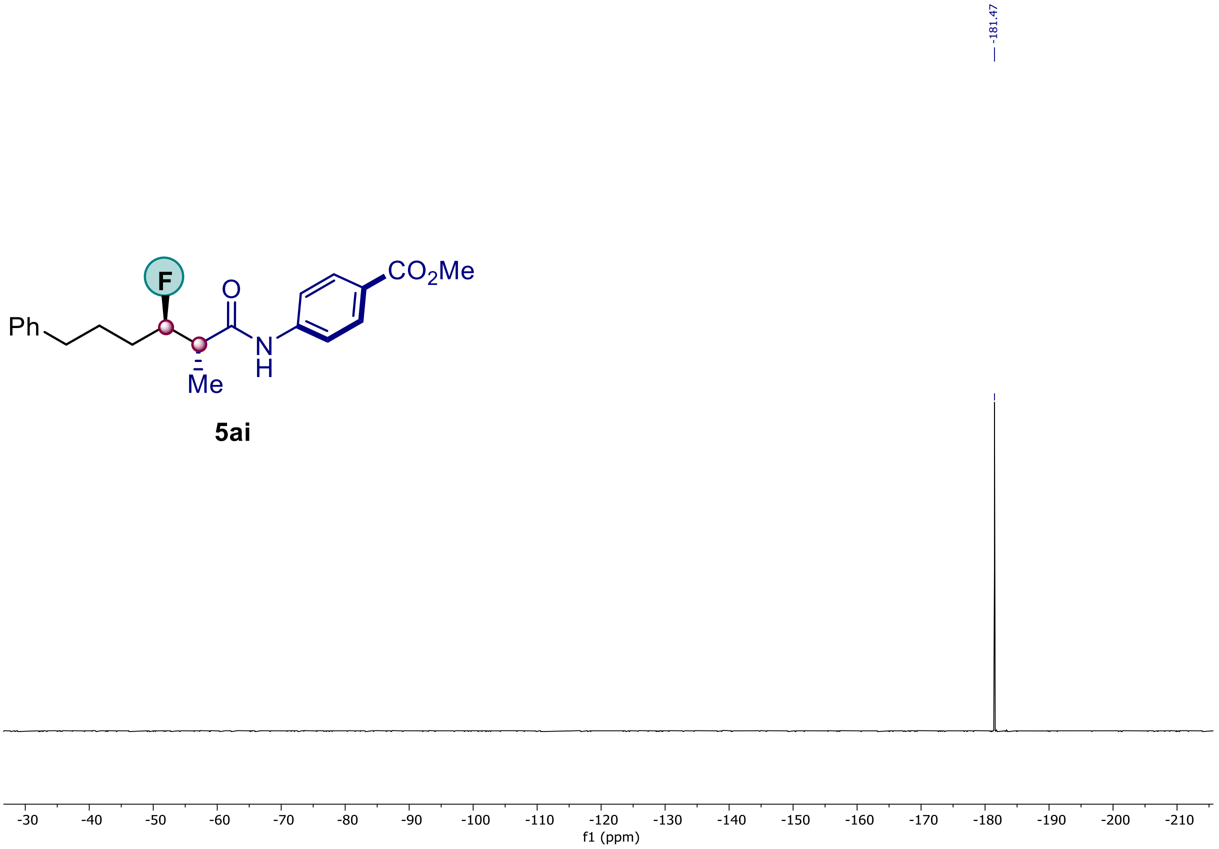

-181.47
Ph
F
O
N
H
CO2Me
Me
5ai
-30
-40
-50
-60
-70
-80
-90
-100
-110
-120
-130
-140
-150
-160
-170
-180
-190
-200
-210
f1 (ppm)

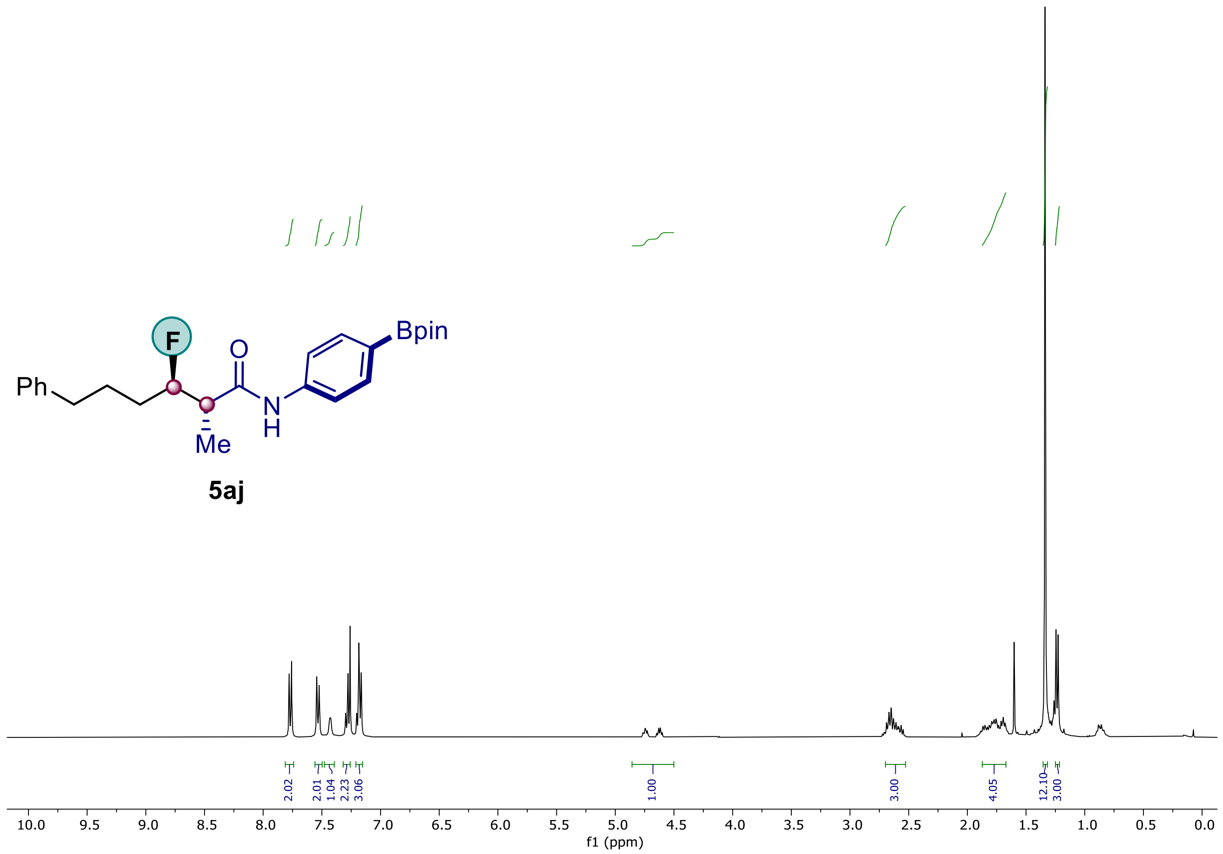

F
O
Bpin
Ph
N
H
Me
5aj
2.02
2.01
1.04
2.23
3.06
1.00
3.00
4.05
12.10
3.00
f1 (ppm)

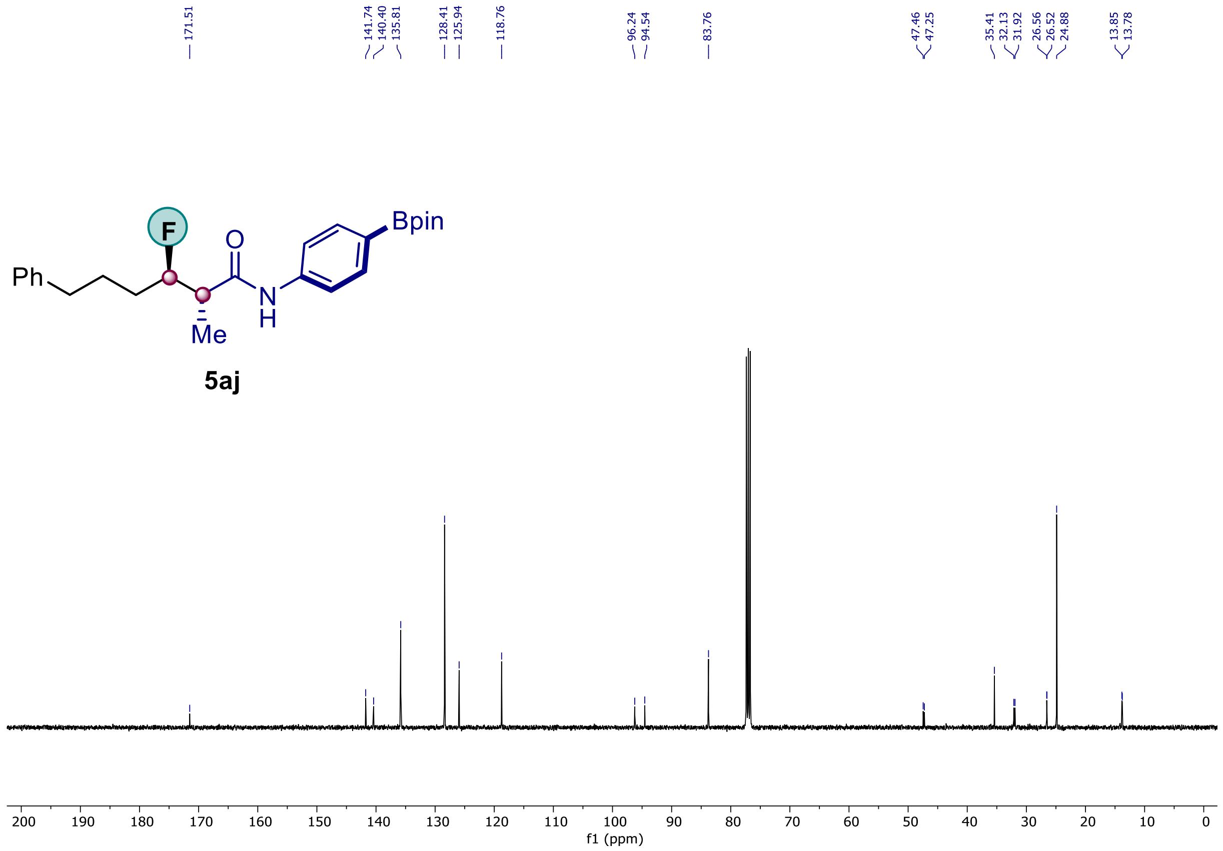

171.51
141.74
140.40
135.81
128.41
125.94
118.76
96.24
94.54
83.76
47.46
47.25
35.41
32.13
31.92
26.56
26.52
24.88
13.85
13.78
F
O
Bpin
Ph
N
H
Me
5aj
f1 (ppm)

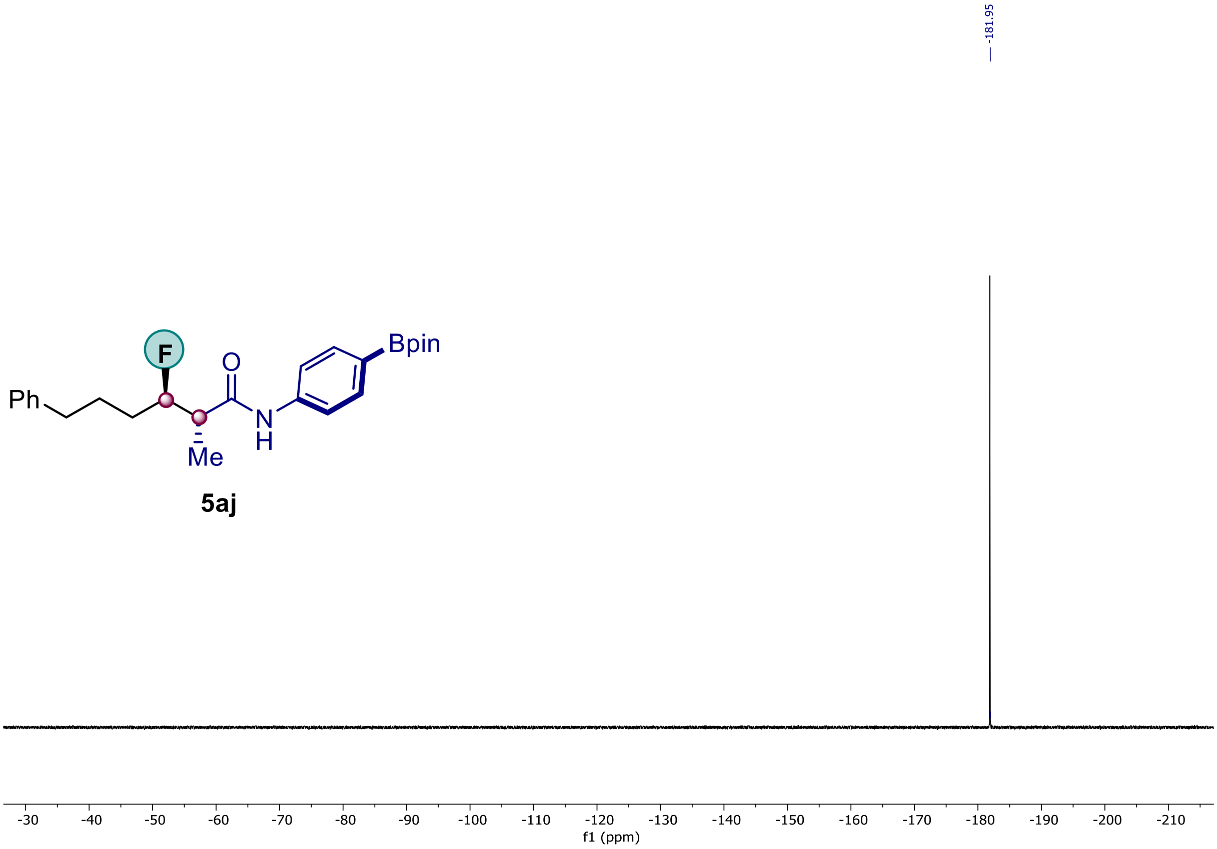

-181.95
Bpin
F
O
Ph
N
H
Me
5aj
-30 -40 -50 -60 -70 -80 -90 -100 -110 -120 -130 -140 -150 -160 -170 -180 -190 -200 -210
f1 (ppm)

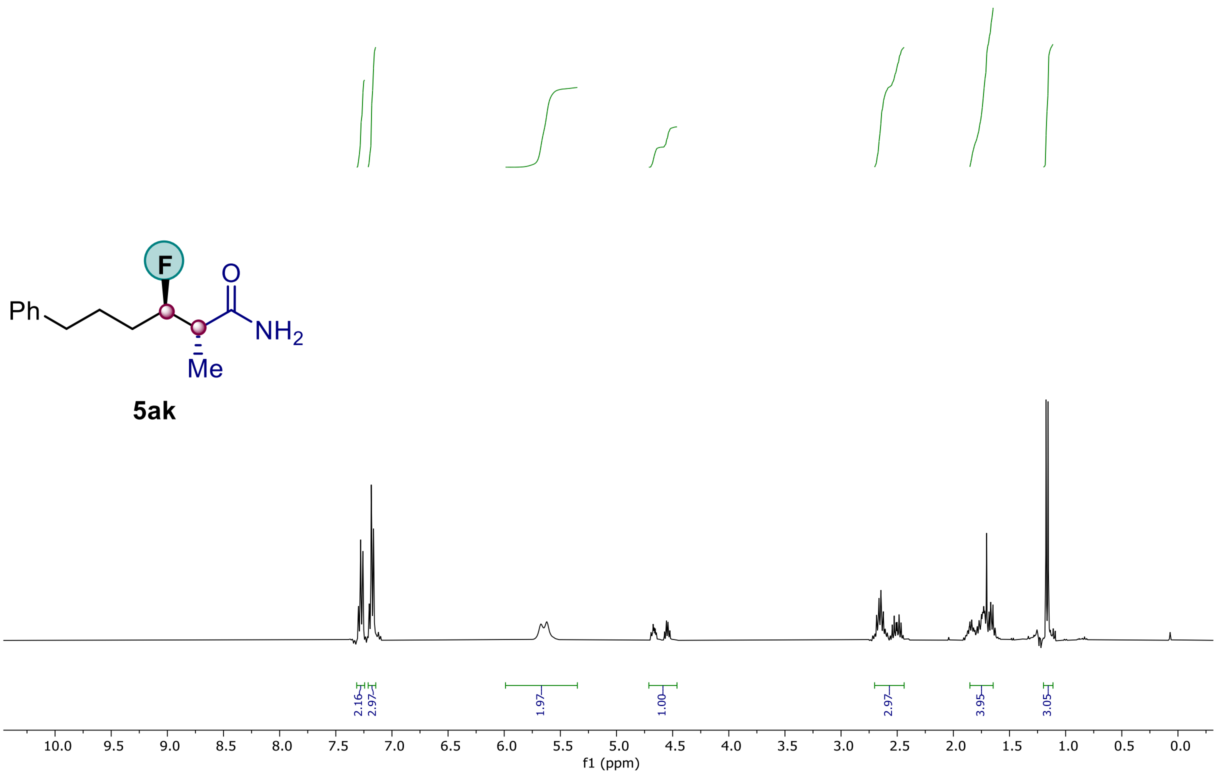

Ph
F
O
NH2
Me
5ak
2.16
2.97
1.97
1.00
2.97
3.95
3.05
10.0 9.5 9.0 8.5 8.0 7.5 7.0 6.5 6.0 5.5 5.0 4.5 4.0 3.5 3.0 2.5 2.0 1.5 1.0 0.5 0.0
f1 (ppm)

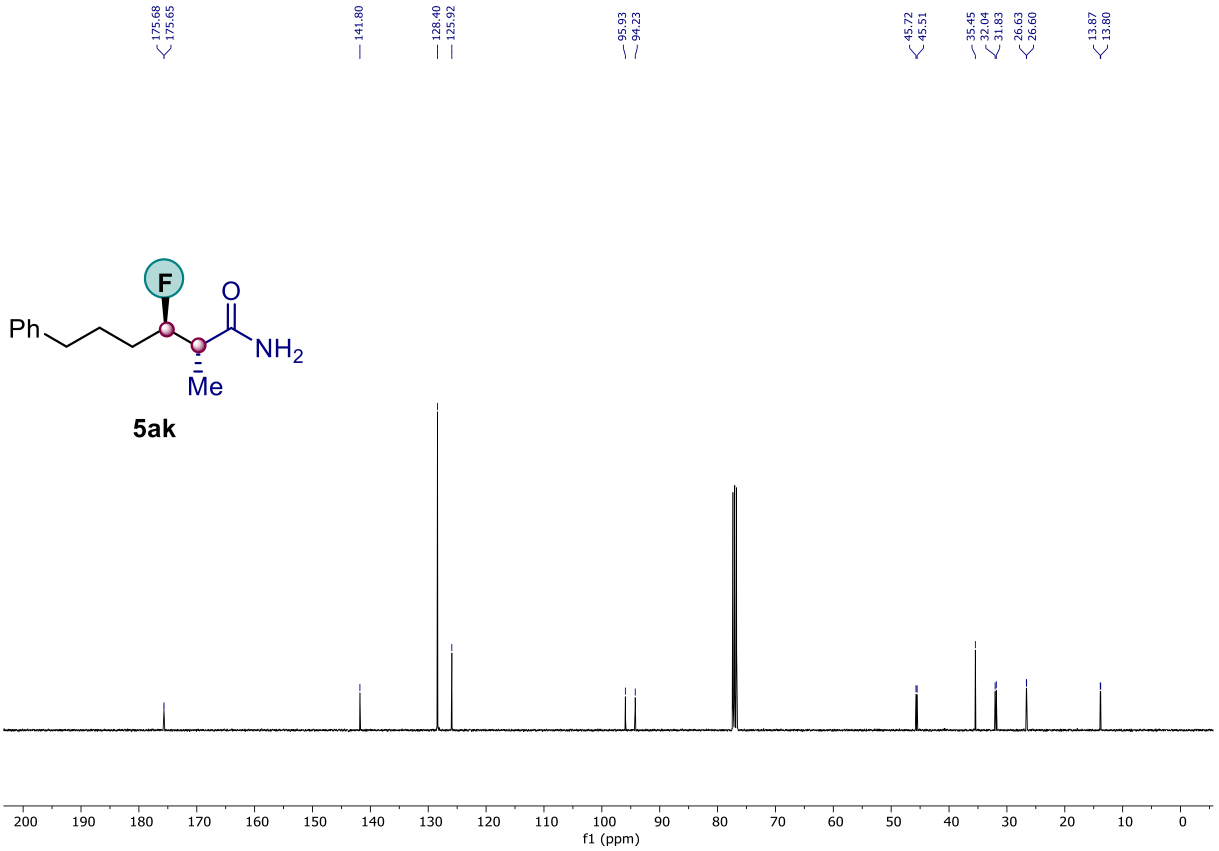

175.68
175.65
141.80
128.40
125.92
95.93
94.23
45.72
45.51
35.45
32.04
31.83
26.63
26.60
13.87
13.80
Ph
F
O
NH2
Me
5ak
200 190 180 170 160 150 140 130 120 110 100 90 80 70 60 50 40 30 20 10 0
f1 (ppm)

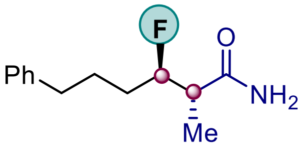
F
O
Ph
NH2
Me

5ak

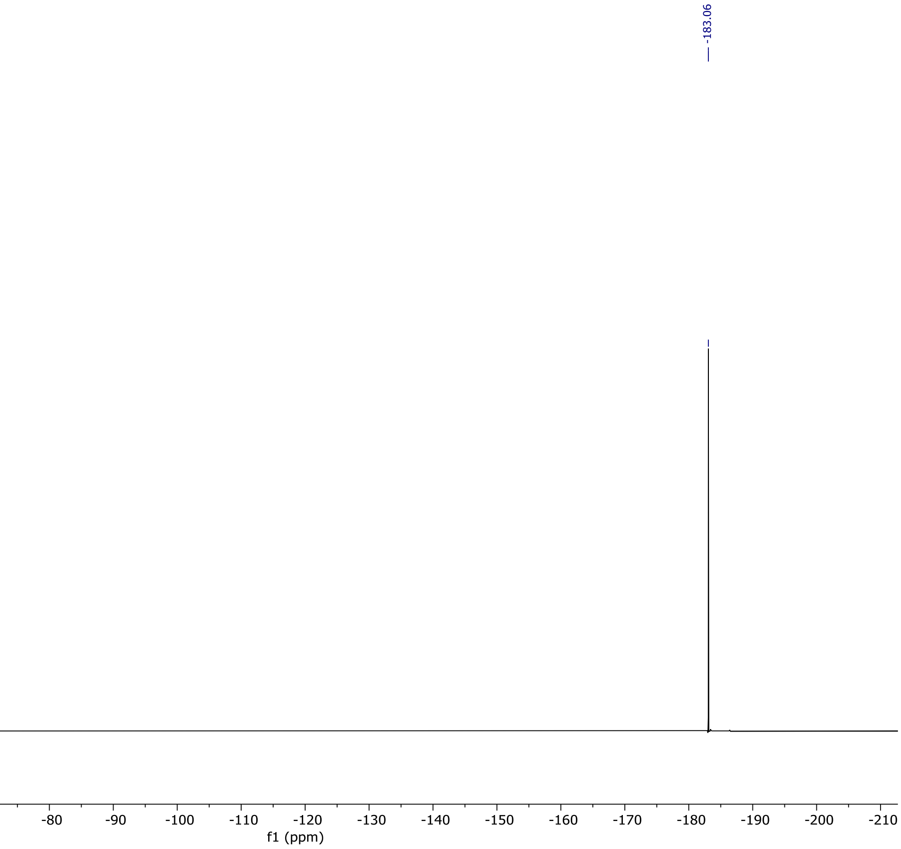
-183.06
-30
-40
-50
-60
-70
-80
-90
-100
-110
-120
-130
-140
-150
-160
-170
-180
-190
-200
-210
f1 (ppm)

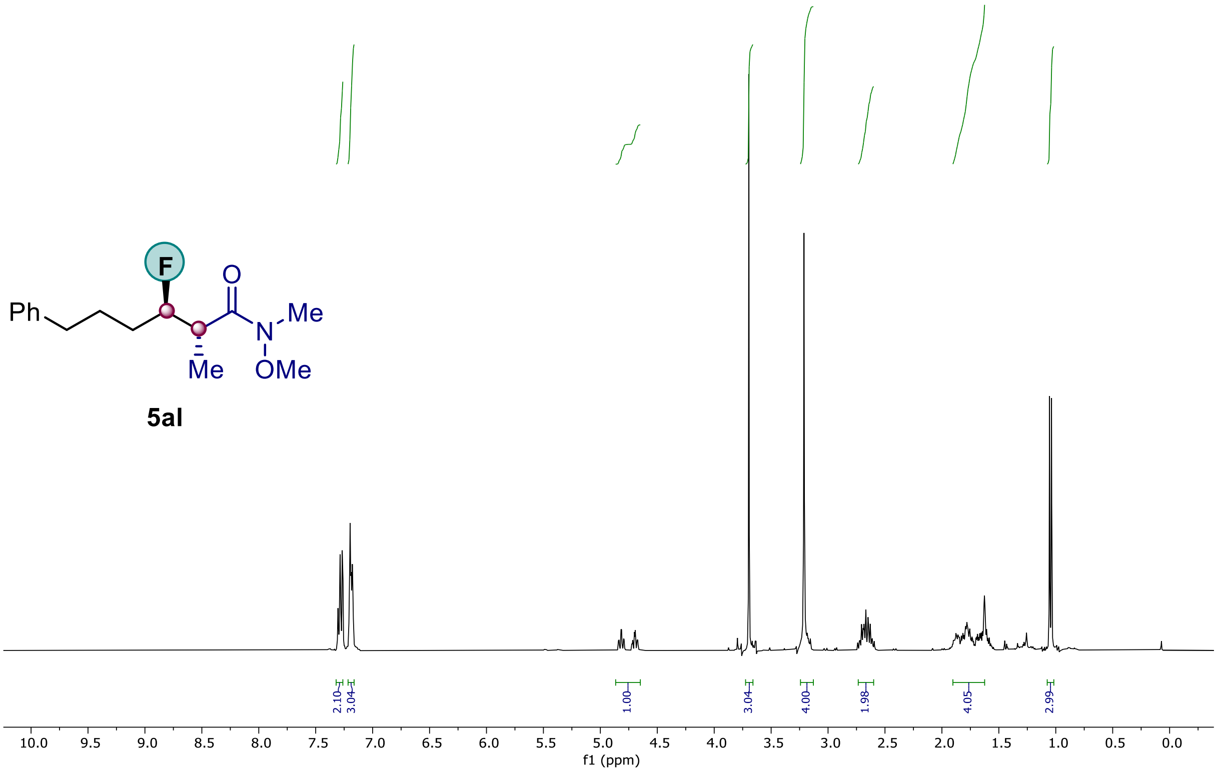

5al

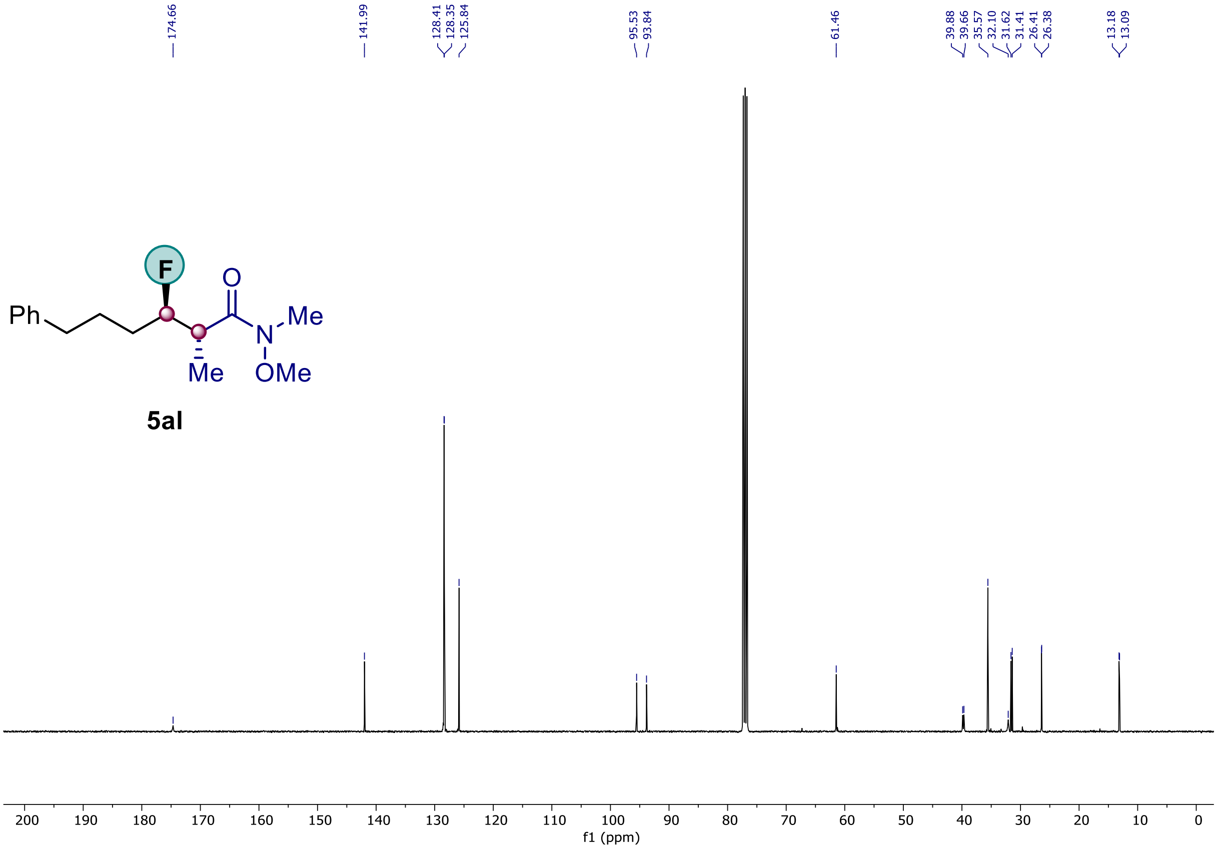

5al

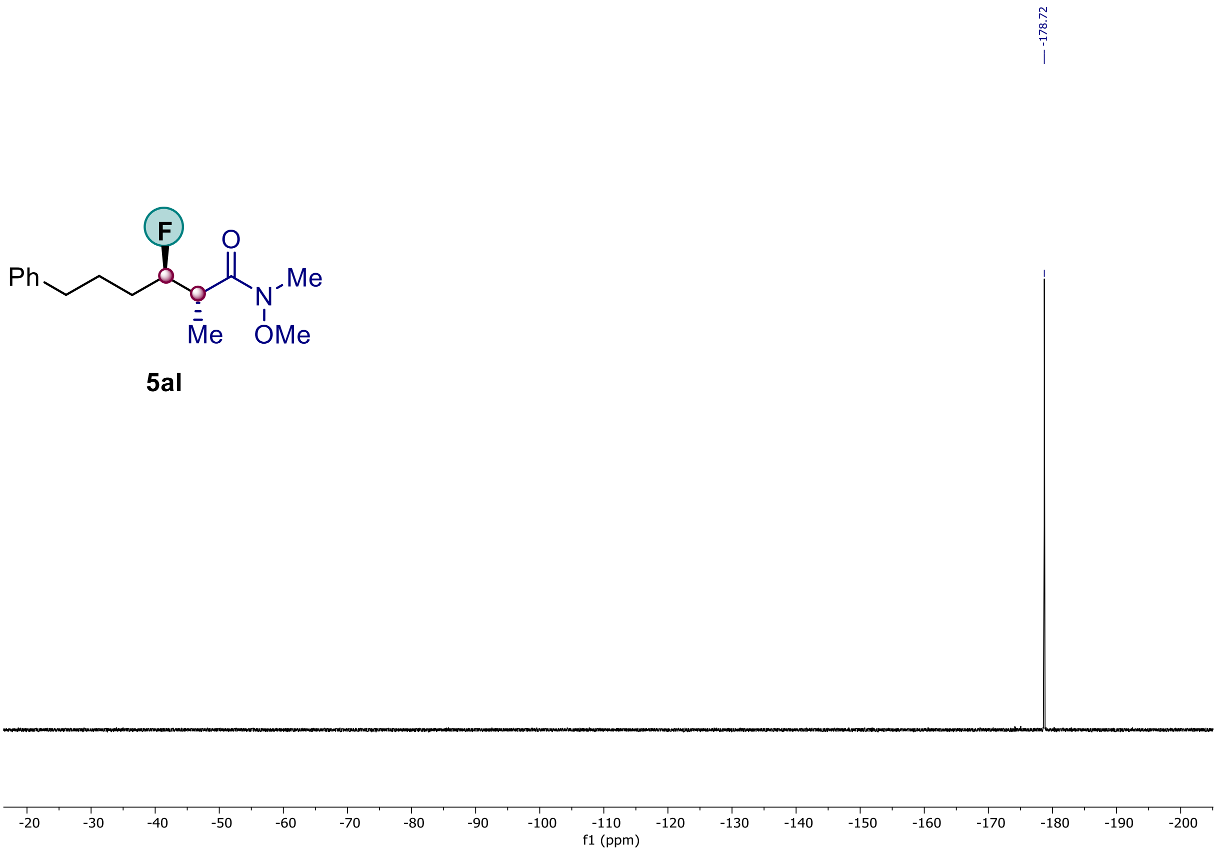
F
O
Ph
N
Me
Me
OMe
5al
-178.72
-20
-30
-40
-50
-60
-70
-80
-90
-100
-110
-120
-130
-140
-150
-160
-170
-180
-190
-200
f1 (ppm)

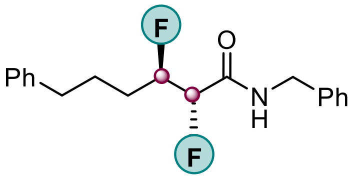
F
O
Ph
N
H
Ph
F

7aa

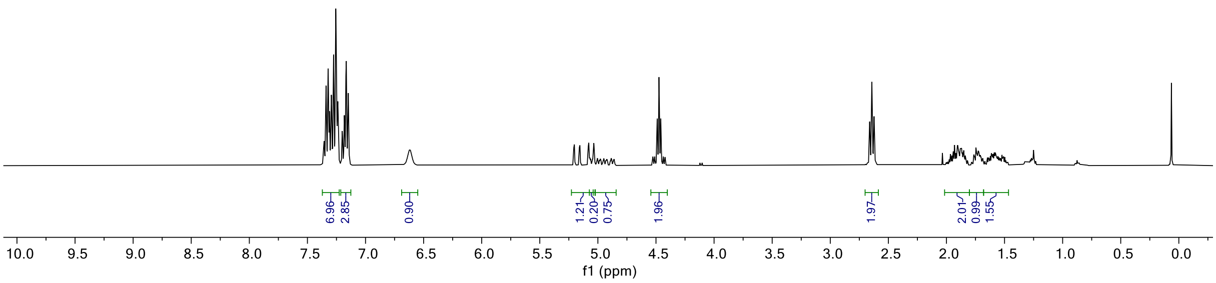
6.96
2.85
0.90
1.21
0.20
0.75
1.96
1.97
2.01
0.99
1.55
10.0 9.5 9.0 8.5 8.0 7.5 7.0 6.5 6.0 5.5 5.0 4.5 4.0 3.5 3.0 2.5 2.0 1.5 1.0 0.5 0.0
f1 (ppm)

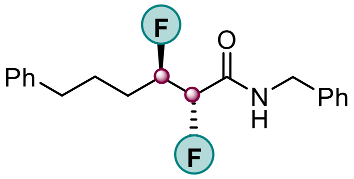
F
O
Ph
N
H
Ph
F

7aa

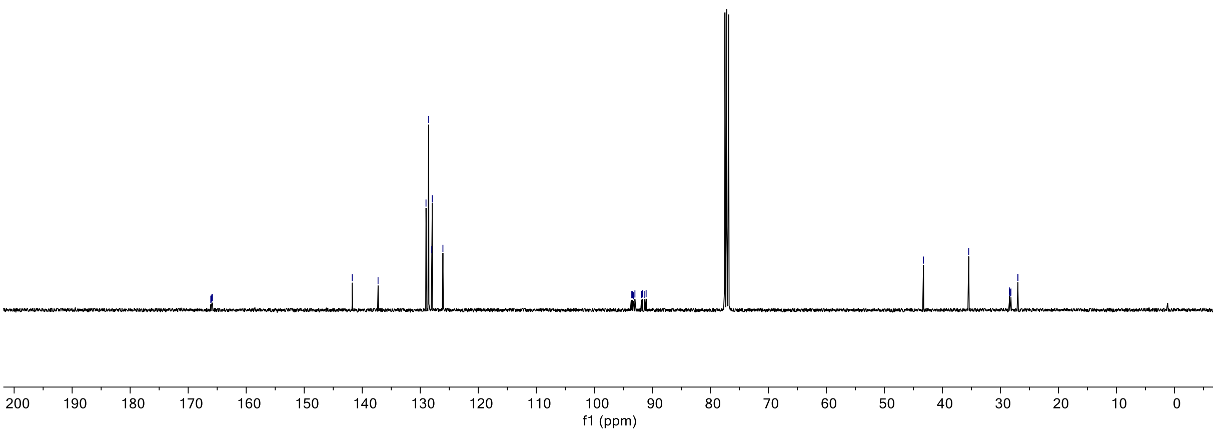
166.11
166.01
165.91
165.82
141.72
137.27
129.01
128.54
128.01
127.93
126.09
93.62
93.43
93.22
92.99
91.86
91.67
91.29
91.07
43.25
35.46
28.45
28.39
28.24
28.18
27.00
26.96
200 190 180 170 160 150 140 130 120 110 100 90 80 70 60 50 40 30 20 10 0
f1 (ppm)

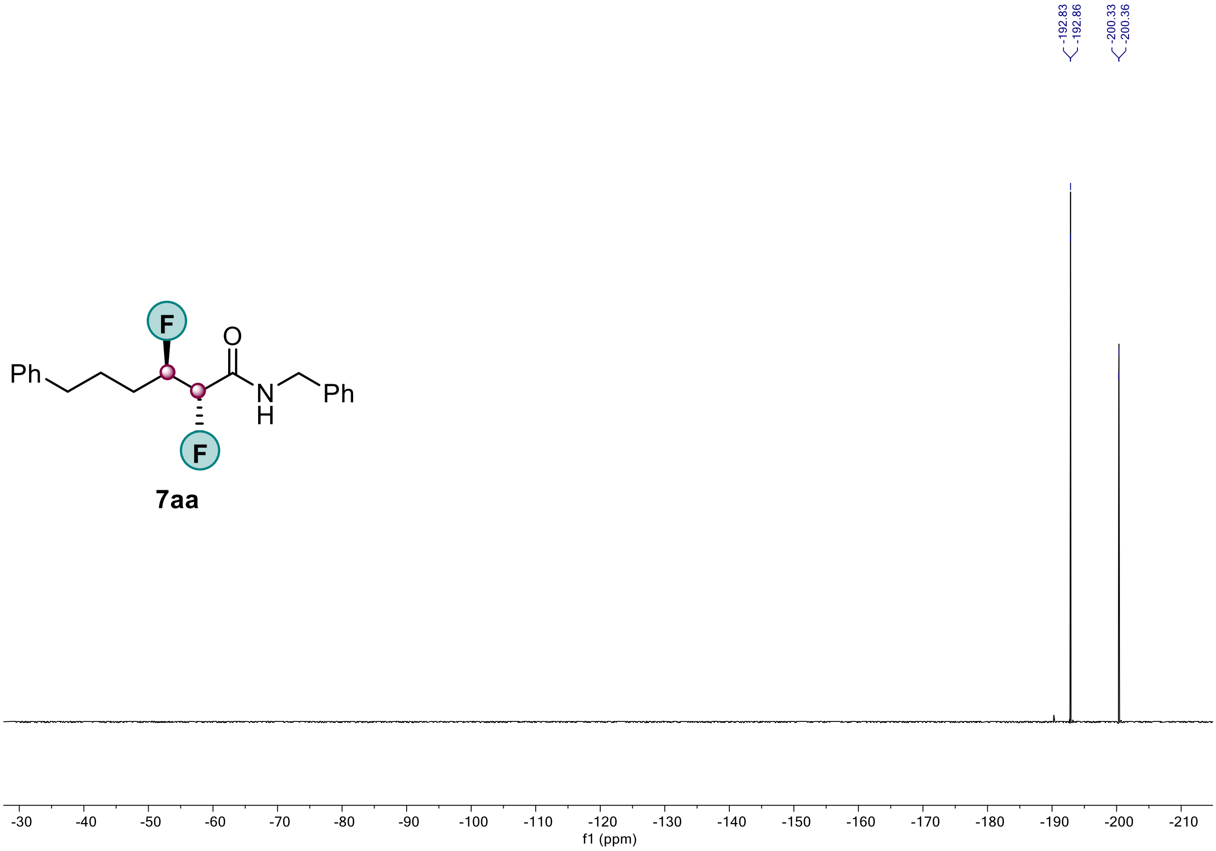
F
O
Ph
N
H
Ph
F
-192.83
-192.86
-200.33
-200.36
-30
-40
-50
-60
-70
-80
-90
-100
-110
-120
-130
-140
-150
-160
-170
-180
-190
-200
-210
f1 (ppm)

7aa

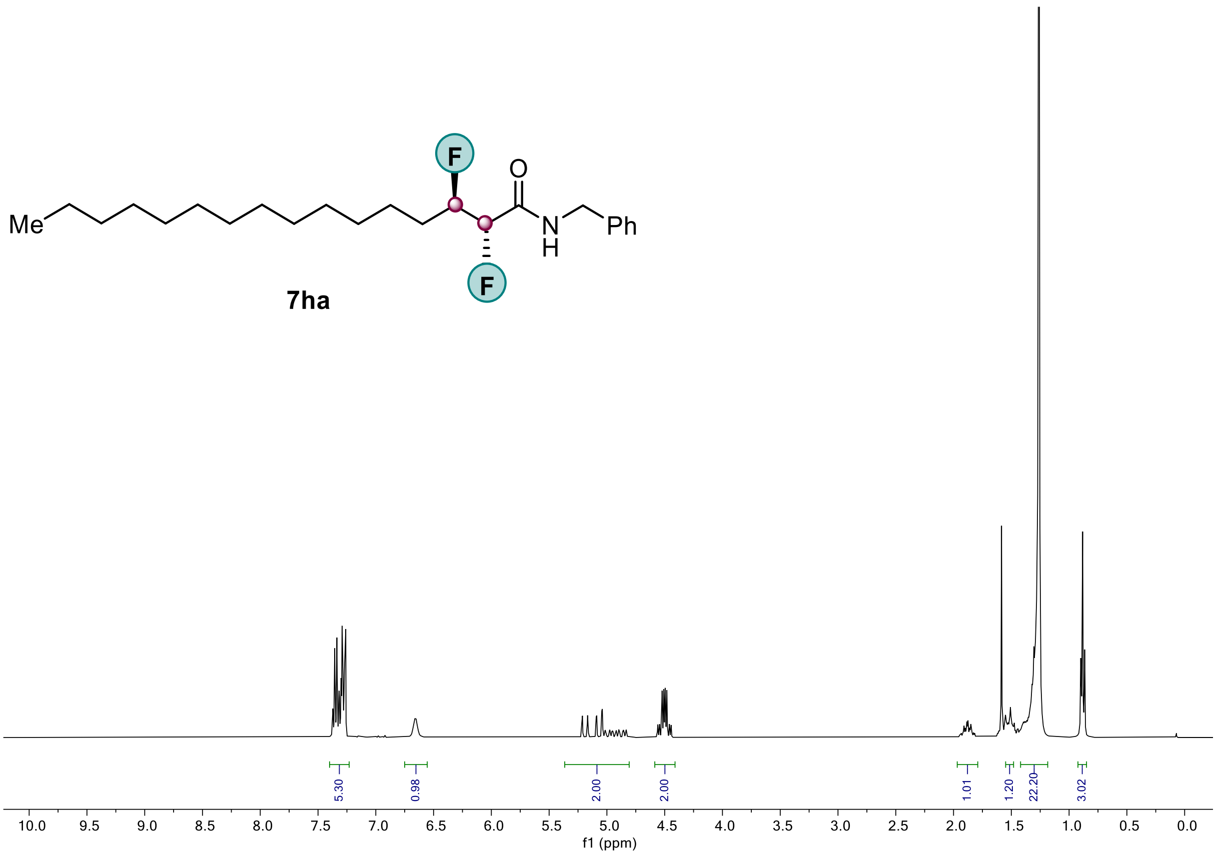
F
O
Me
N
H
Ph
F
7ha
5.30
0.98
2.00
2.00
1.01
1.20
22.20
3.02
10.0 9.5 9.0 8.5 8.0 7.5 7.0 6.5 6.0 5.5 5.0 4.5 4.0 3.5 3.0 2.5 2.0 1.5 1.0 0.5 0.0
f1 (ppm)

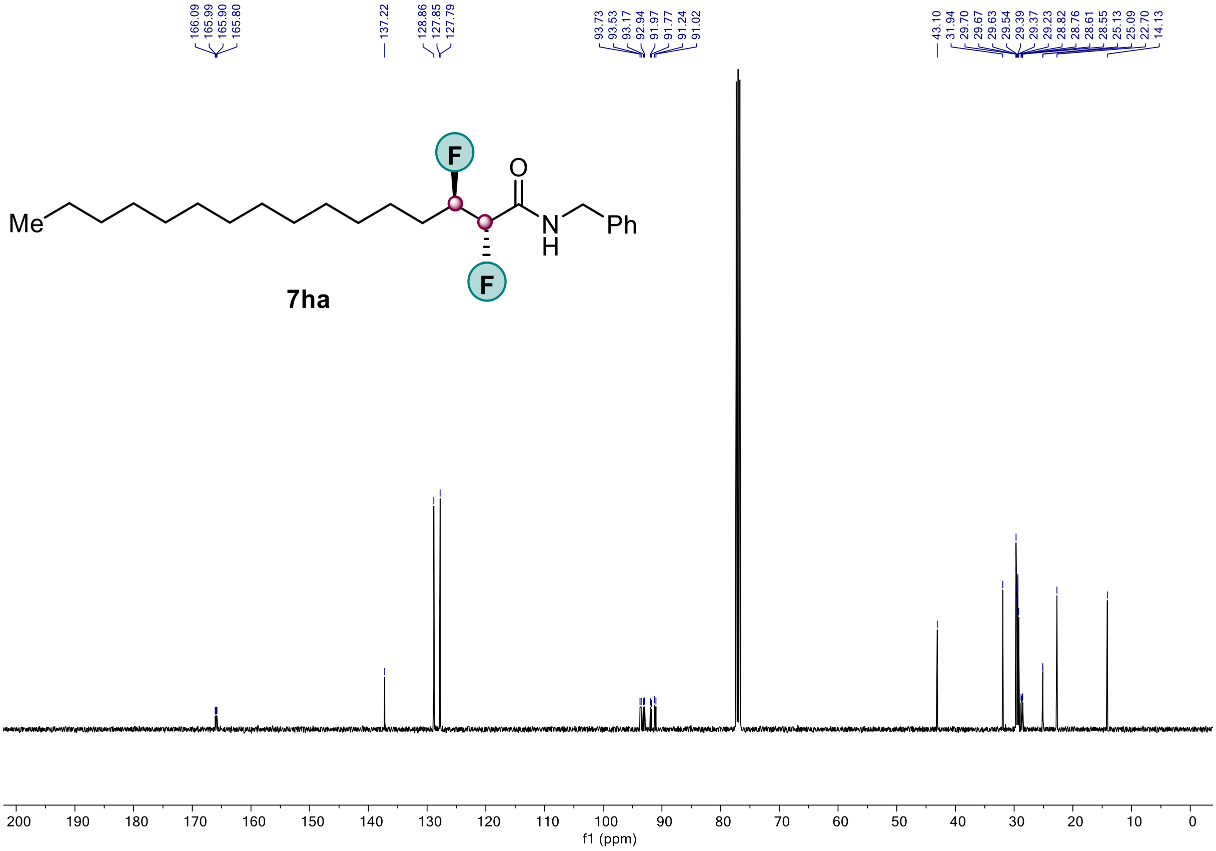
166.09
165.99
165.90
165.80
137.22
128.86
127.85
127.79
93.73
93.53
93.17
92.94
91.97
91.77
91.24
91.02
43.10
31.94
29.70
29.67
29.63
29.54
29.39
29.37
29.23
28.82
28.76
28.61
28.55
25.13
25.09
22.70
14.13
F
O
Me
N
H
Ph
F
7ha
200 190 180 170 160 150 140 130 120 110 100 90 80 70 60 50 40 30 20 10 0
f1 (ppm)

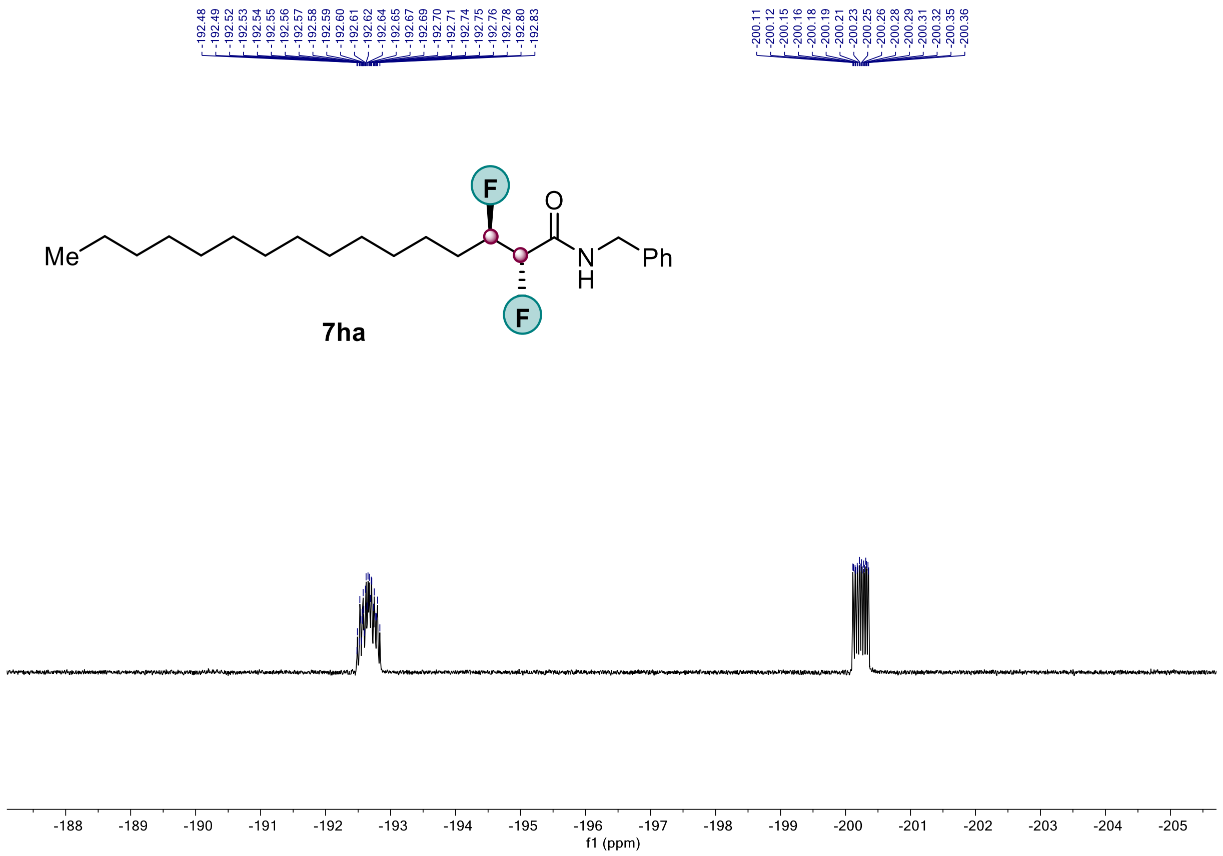
-192.48
-192.49
-192.52
-192.53
-192.54
-192.55
-192.56
-192.57
-192.58
-192.59
-192.60
-192.61
-192.62
-192.64
-192.65
-192.67
-192.69
-192.70
-192.71
-192.74
-192.75
-192.76
-192.78
-192.80
-192.83
-200.11
-200.12
-200.15
-200.16
-200.18
-200.19
-200.21
-200.23
-200.25
-200.26
-200.28
-200.29
-200.31
-200.32
-200.35
-200.36
F
O
Me
N
H
Ph
F
7ha
-188
-189
-190
-191
-192
-193
-194
-195
-196
-197
-198
-199
-200
-201
-202
-203
-204
-205
f1 (ppm)

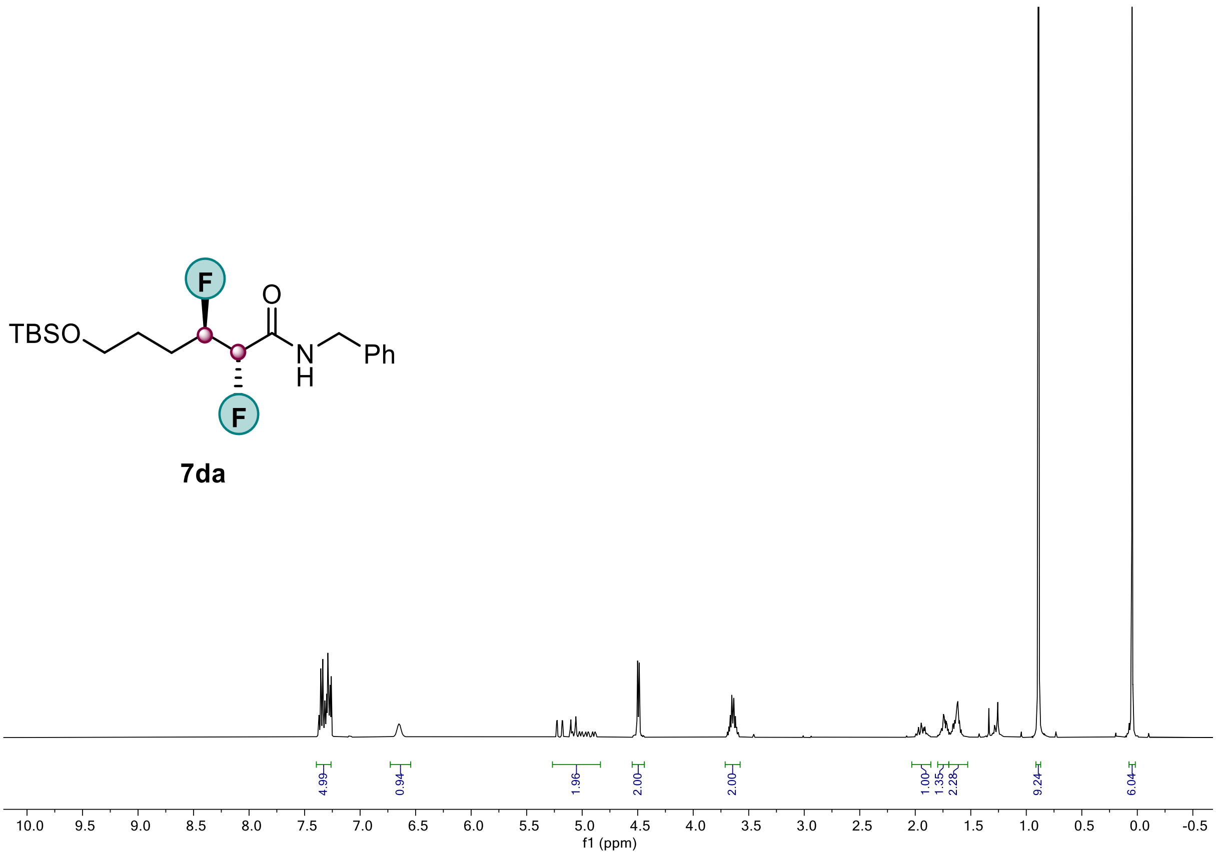

7da

4.99
0.94
1.96
2.00
2.00
1.00
1.35
2.28
9.24
6.04
f1 (ppm)

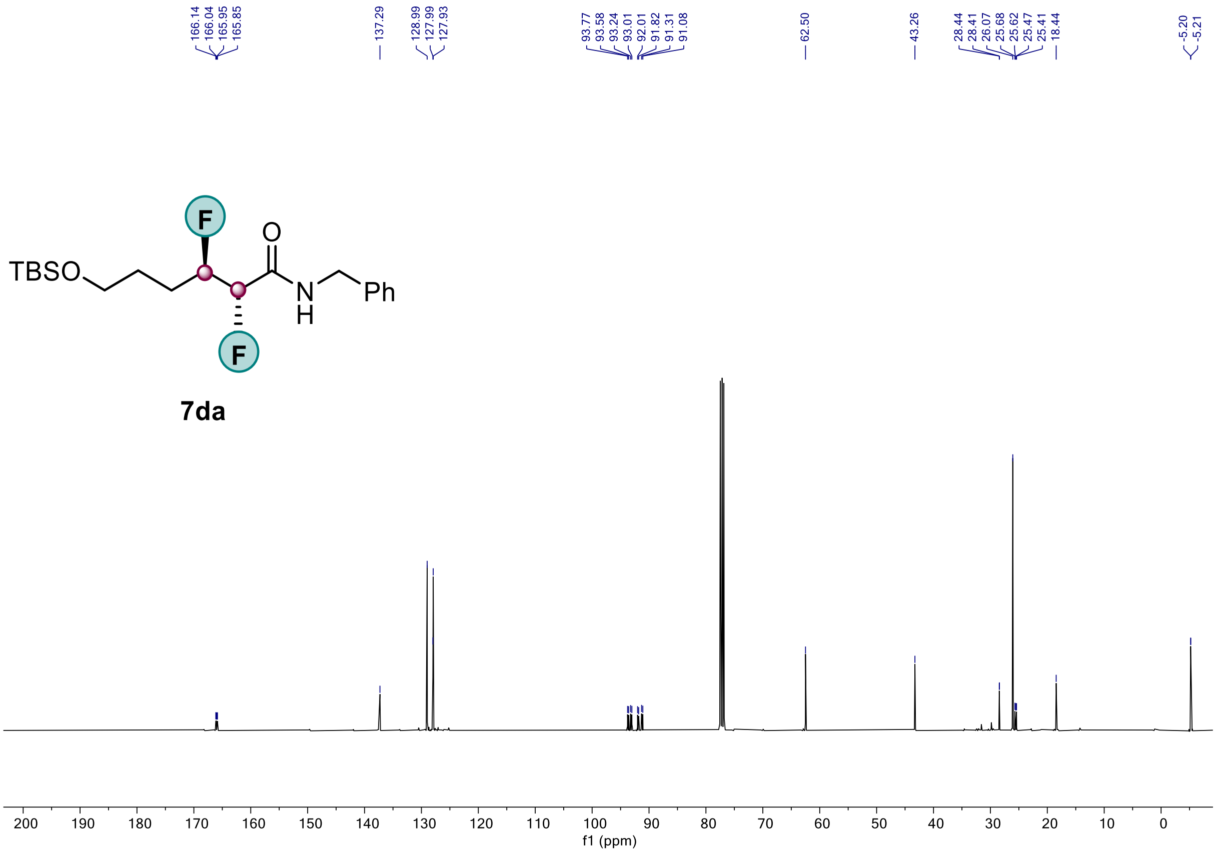

7da

166.14
166.04
165.95
165.85
137.29
128.99
127.99
127.93
93.77
93.58
93.24
93.01
92.01
91.82
91.31
91.08
62.50
43.26
28.44
28.41
26.07
25.68
25.62
25.47
25.41
18.44
-5.20
-5.21
f1 (ppm)

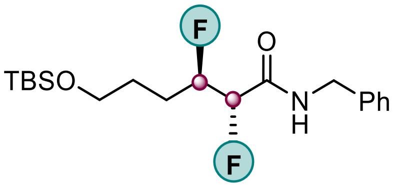

TBSO
F
O
N
H
Ph
F

7da

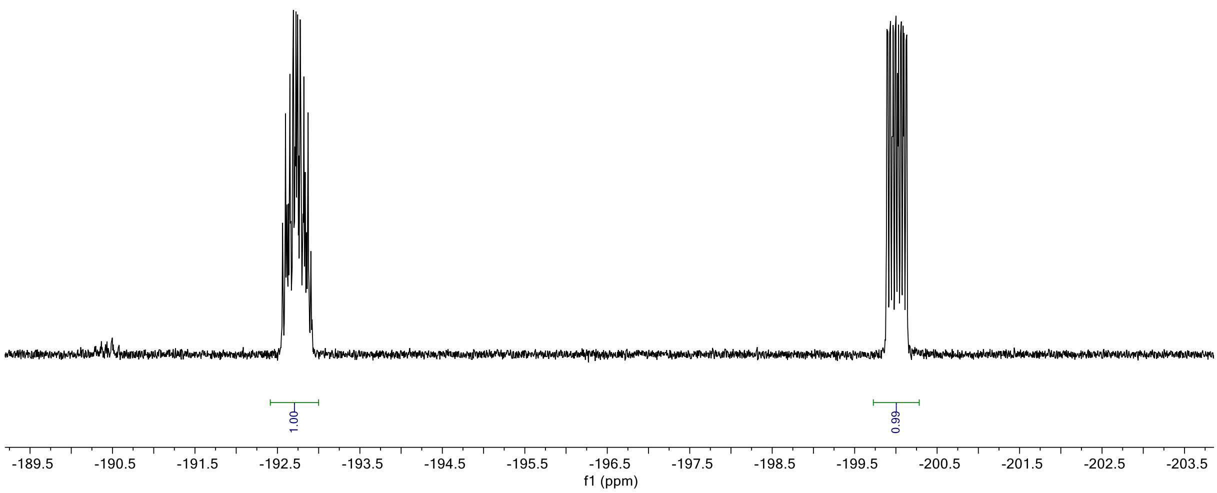

1.00
0.99
-189.5
-190.5
-191.5
-192.5
-193.5
-194.5
-195.5
-196.5
-197.5
-198.5
-199.5
-200.5
-201.5
-202.5
-203.5
f1 (ppm)

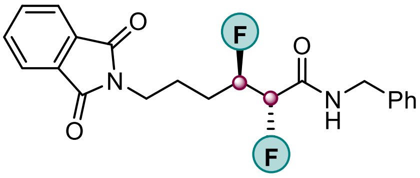
O
F
O
N
N
H
Ph
O
F

7ea

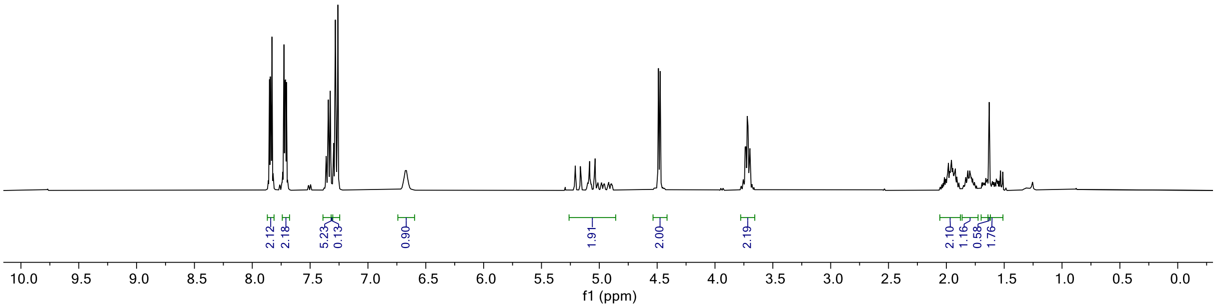
2.12
2.18
5.23
0.13
0.90
1.91
2.00
2.19
2.10
1.16
0.58
1.76
10.0 9.5 9.0 8.5 8.0 7.5 7.0 6.5 6.0 5.5 5.0 4.5 4.0 3.5 3.0 2.5 2.0 1.5 1.0 0.5 0.0
f1 (ppm)

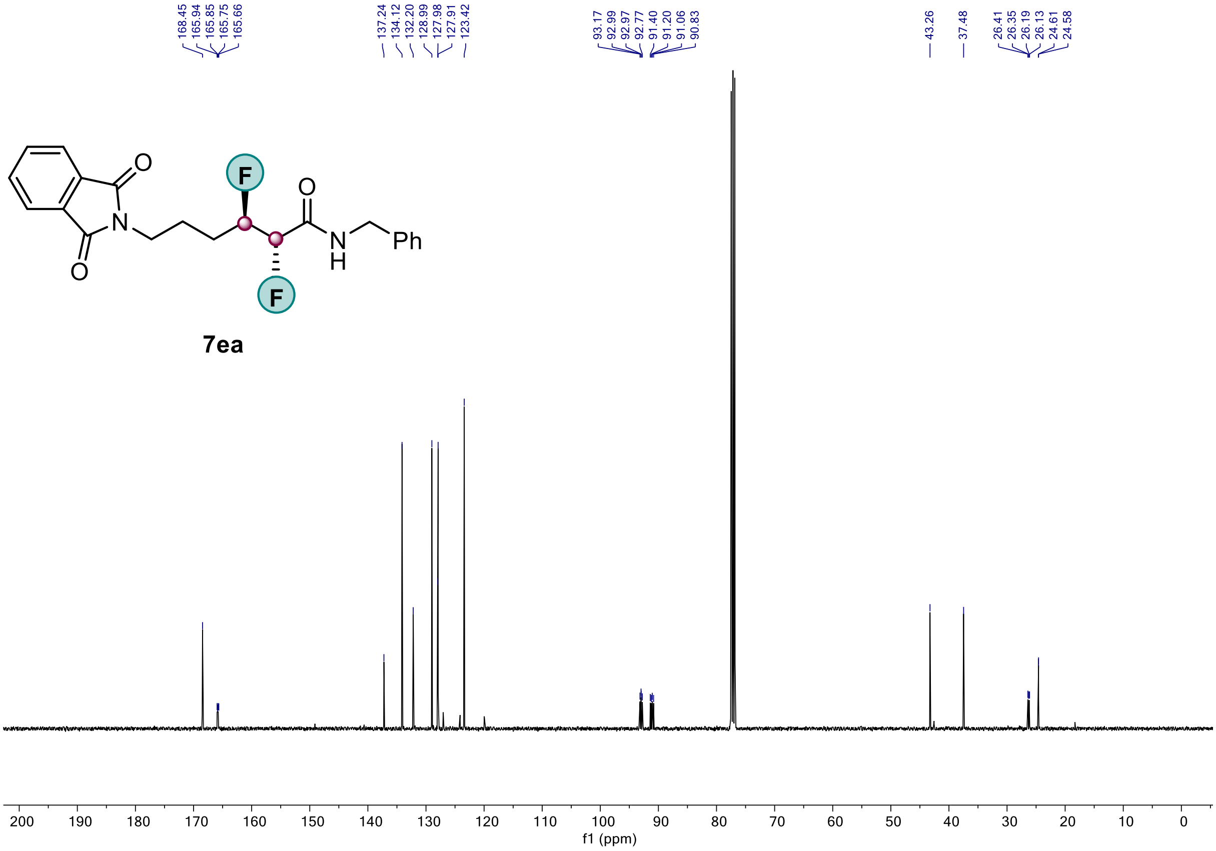
168.45
165.94
165.85
165.75
165.66
137.24
134.12
132.20
128.99
127.98
127.91
123.42
93.17
92.99
92.97
92.77
91.40
91.20
91.06
90.83
43.26
37.48
26.41
26.35
26.19
26.13
24.61
24.58
O
F
O
N
N
H
Ph
O
F
7ea
200 190 180 170 160 150 140 130 120 110 100 90 80 70 60 50 40 30 20 10 0
f1 (ppm)

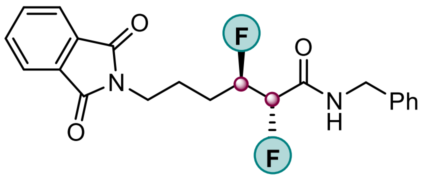
O
F
O
N
N
H
Ph
O
F

7ea

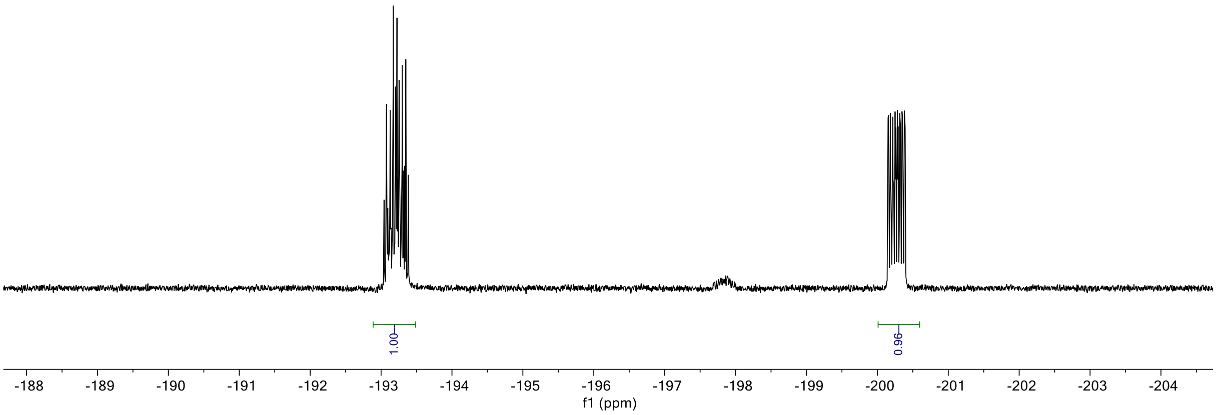
1.00
0.96
-188
-189
-190
-191
-192
-193
-194
-195
-196
-197
-198
-199
-200
-201
-202
-203
-204
f1 (ppm)

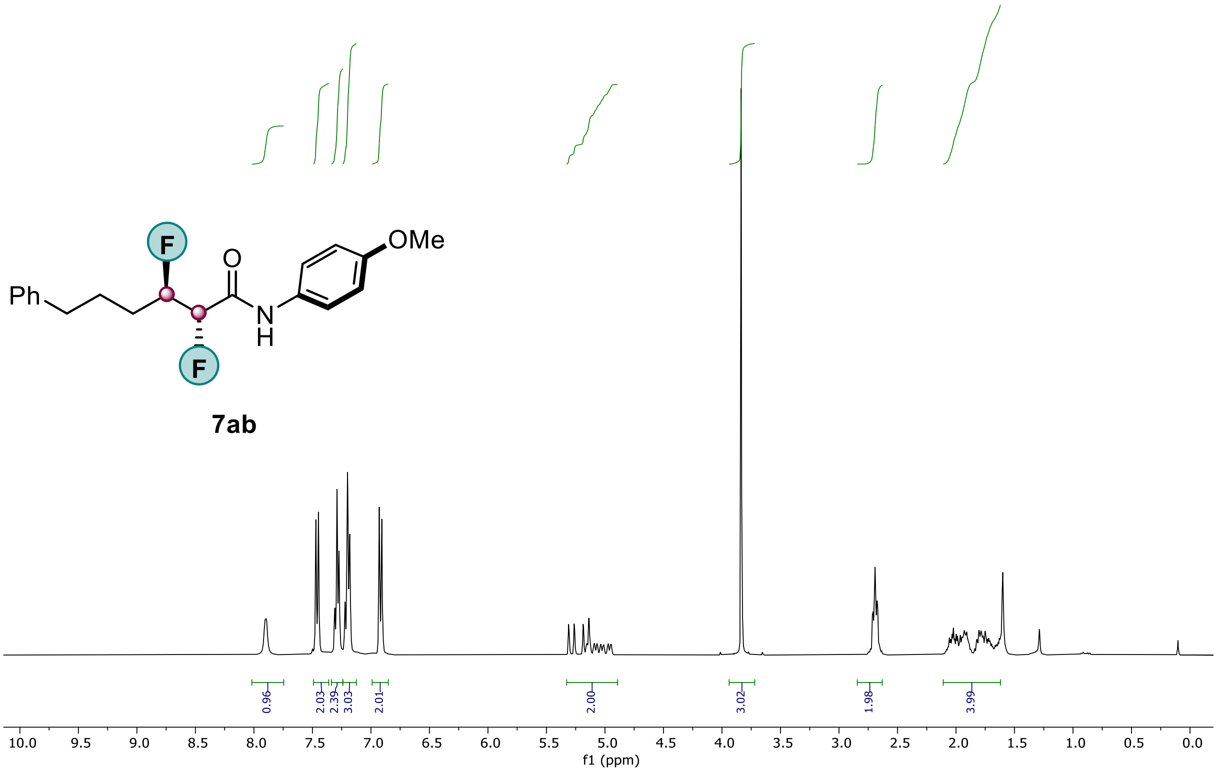
OMe
Ph
F
O
N
H
F
7ab
0.96
2.03
2.39
3.03
2.01
2.00
3.02
1.98
3.99
10.0 9.5 9.0 8.5 8.0 7.5 7.0 6.5 6.0 5.5 5.0 4.5 4.0 3.5 3.0 2.5 2.0 1.5 1.0 0.5 0.0
f1 (ppm)

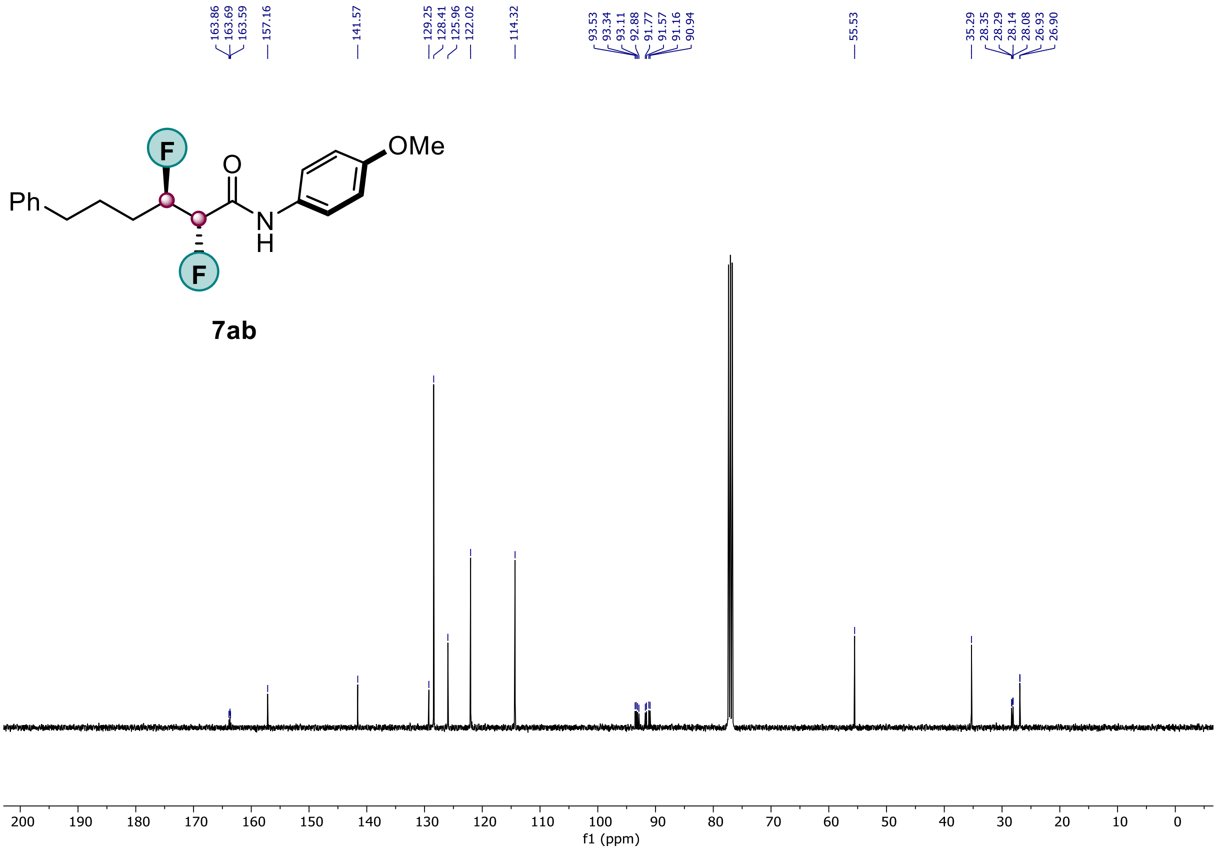
163.86
163.69
163.59
157.16
141.57
129.25
128.41
125.96
122.02
114.32
93.53
93.34
93.11
92.88
91.77
91.57
91.16
90.94
55.53
35.29
28.35
28.29
28.14
28.08
26.93
26.90
OMe
Ph
F
O
N
H
F
7ab
200 190 180 170 160 150 140 130 120 110 100 90 80 70 60 50 40 30 20 10 0
f1 (ppm)

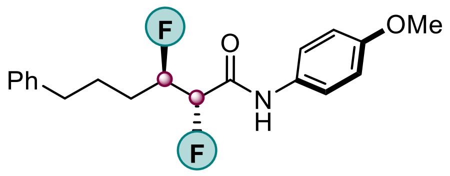
F
F
O
OMe
Ph
N
H

7ab

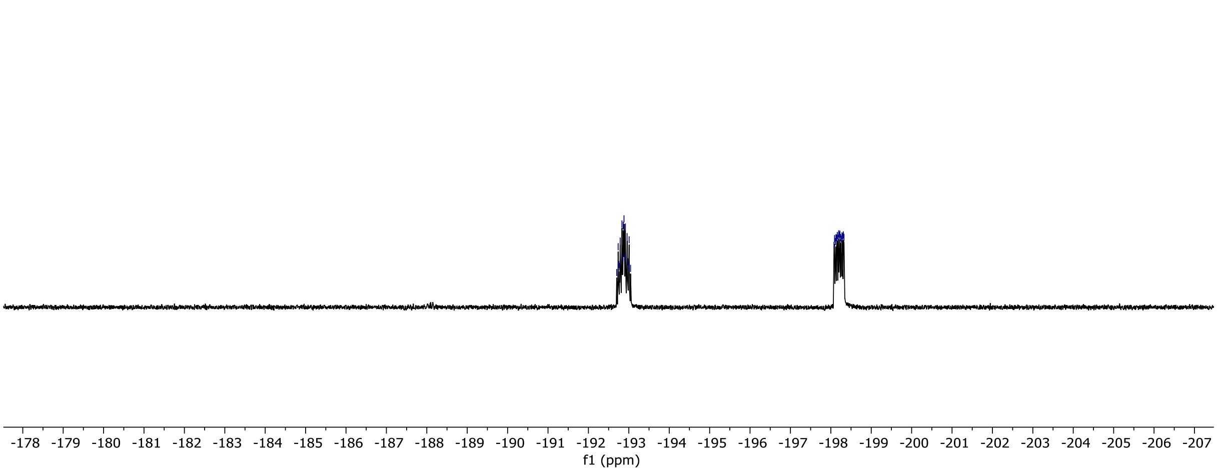
-192.70
-192.74
-192.75
-192.77
-192.79
-192.83
-192.85
-192.86
-192.88
-192.90
-192.92
-192.96
-192.97
-192.99
-193.01
-193.04
-198.08
-198.09
-198.11
-198.13
-198.14
-198.16
-198.18
-198.19
-198.21
-198.22
-198.24
-198.26
-198.28
-198.29
-198.31
-198.33
-178 -179 -180 -181 -182 -183 -184 -185 -186 -187 -188 -189 -190 -191 -192 -193 -194 -195 -196 -197 -198 -199 -200 -201 -202 -203 -204 -205 -206 -207
f1 (ppm)

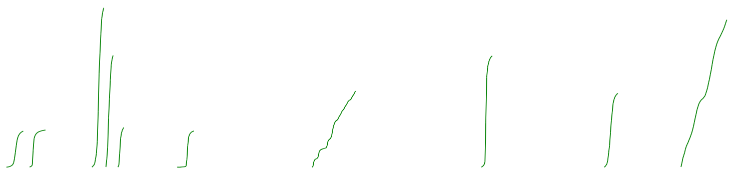

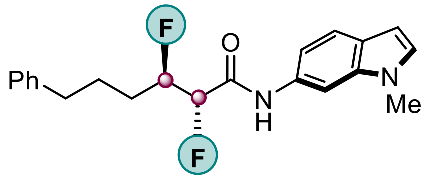

F
O
Ph
N
H
N
Me
F

7ac

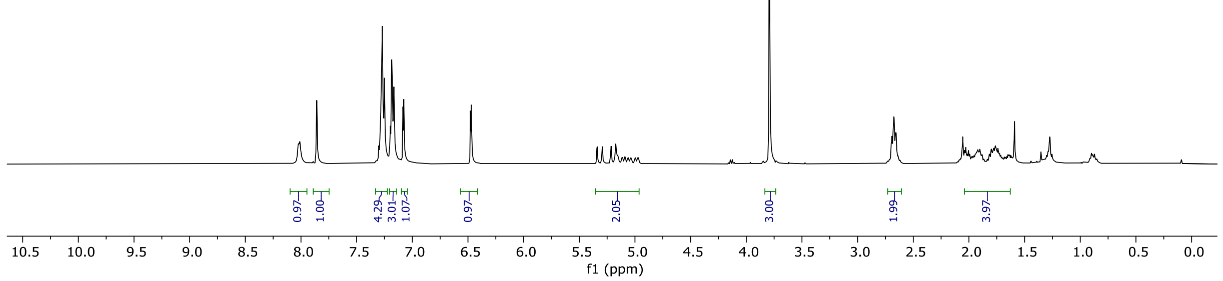

0.97
1.00
4.29
3.01
1.07
0.97
2.05
3.00
1.99
3.97
10.5 10.0 9.5 9.0 8.5 8.0 7.5 7.0 6.5 6.0 5.5 5.0 4.5 4.0 3.5 3.0 2.5 2.0 1.5 1.0 0.5 0.0
f1 (ppm)

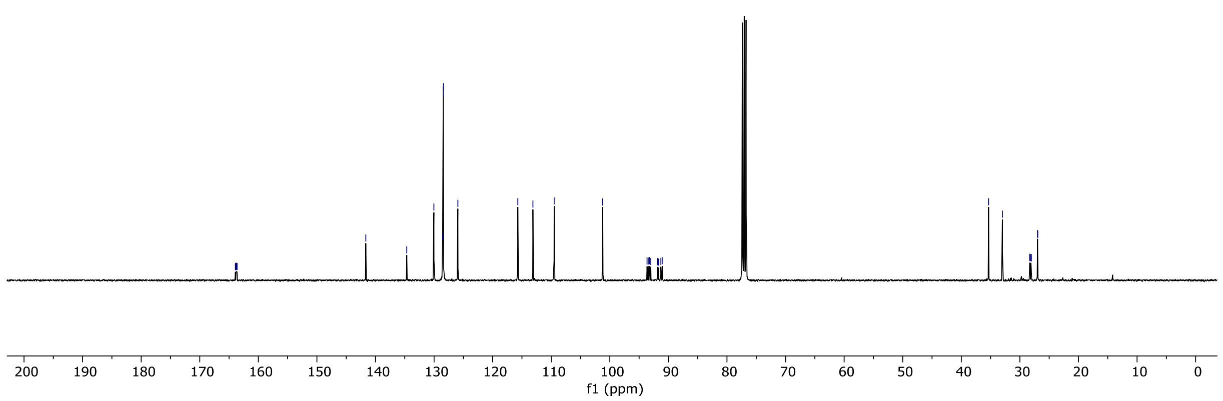

163.92
163.81
163.74
163.63
141.65
134.66
130.03
128.55
128.44
128.41
125.94
115.70
113.12
109.50
101.21
93.64
93.45
93.23
93.00
91.88
91.68
91.28
91.06
35.34
32.99
28.32
28.26
28.11
28.05
26.99
26.95
200 190 180 170 160 150 140 130 120 110 100 90 80 70 60 50 40 30 20 10 0
f1 (ppm)

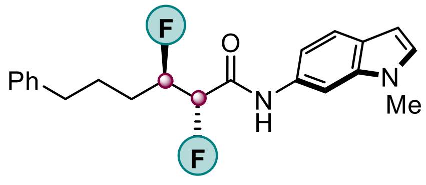

F
O
Ph
N
H
N
Me
F

7ac

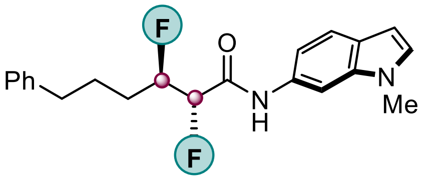

7ac

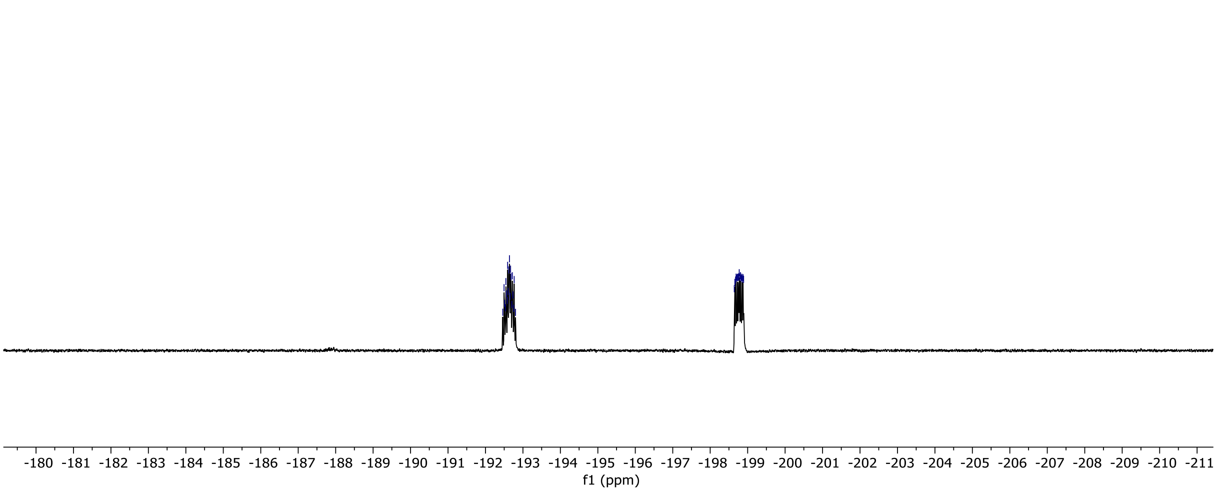

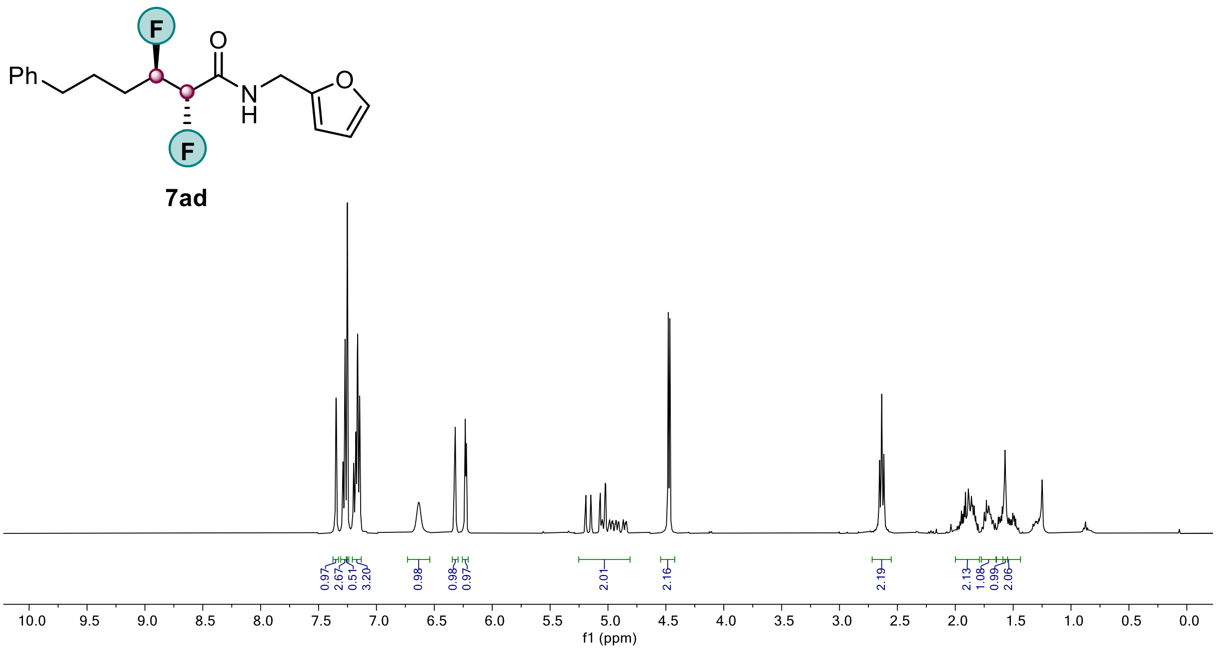
Ph
F
O
N
H
O
F
7ad
0.97
2.67
0.51
3.20
0.98
0.98
0.97
2.01
2.16
2.19
2.13
1.08
0.99
2.06
10.0 9.5 9.0 8.5 8.0 7.5 7.0 6.5 6.0 5.5 5.0 4.5 4.0 3.5 3.0 2.5 2.0 1.5 1.0 0.5 0.0
f1 (ppm)

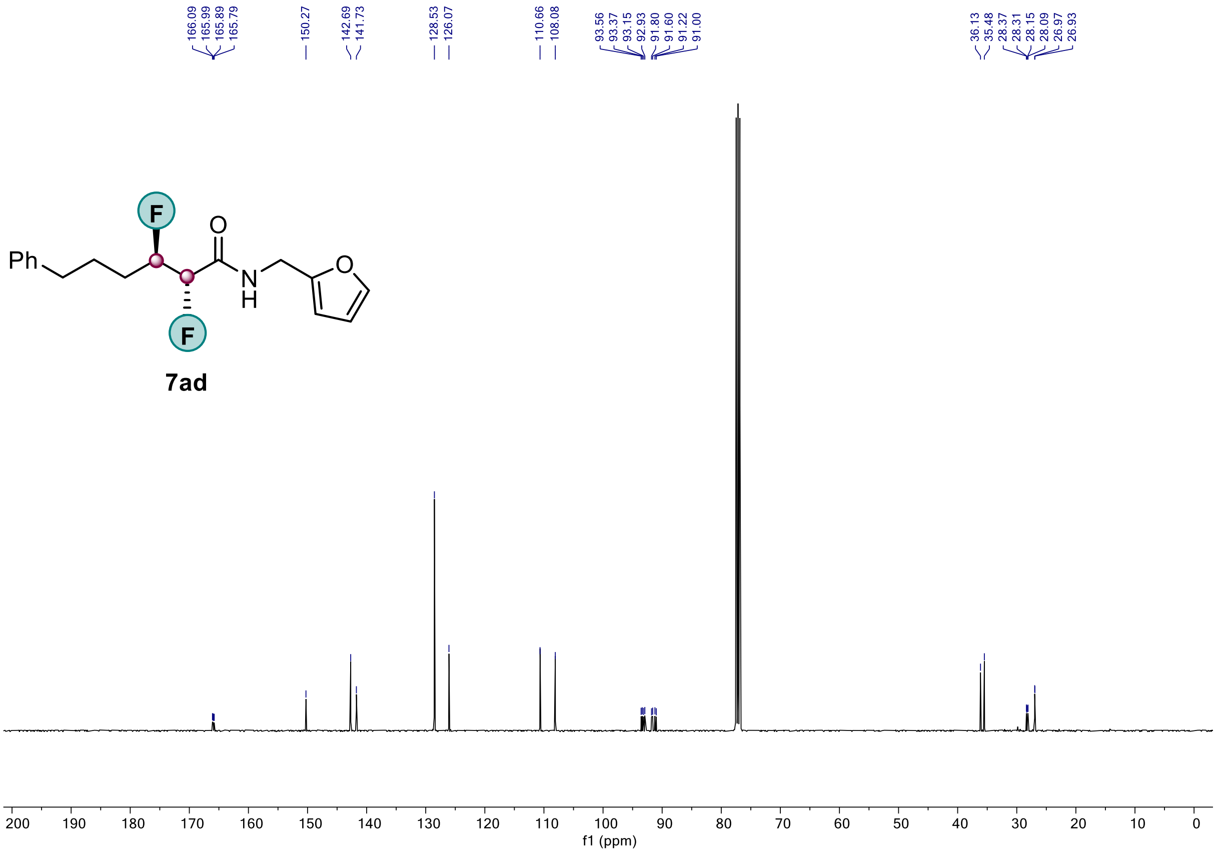
166.09
165.99
165.89
165.79
150.27
142.69
141.73
128.53
126.07
110.66
108.08
93.56
93.37
93.15
92.93
91.80
91.60
91.22
91.00
36.13
35.48
28.37
28.31
28.15
28.09
26.97
26.93
Ph
F
O
N
H
O
F
7ad
200 190 180 170 160 150 140 130 120 110 100 90 80 70 60 50 40 30 20 10 0
f1 (ppm)

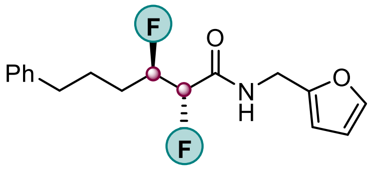
Ph
O
N
H
O
F
F

7ad

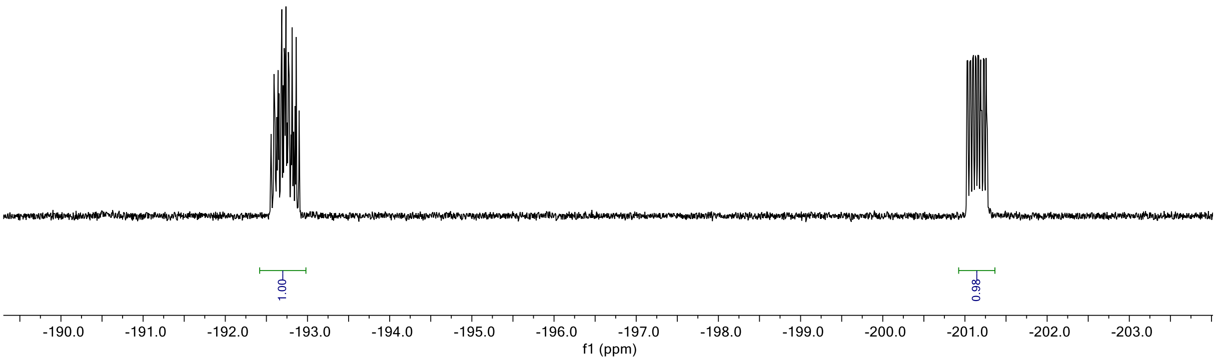
1.00
0.98
-190.0
-191.0
-192.0
-193.0
-194.0
-195.0
-196.0
-197.0
-198.0
-199.0
-200.0
-201.0
-202.0
-203.0
f1 (ppm)

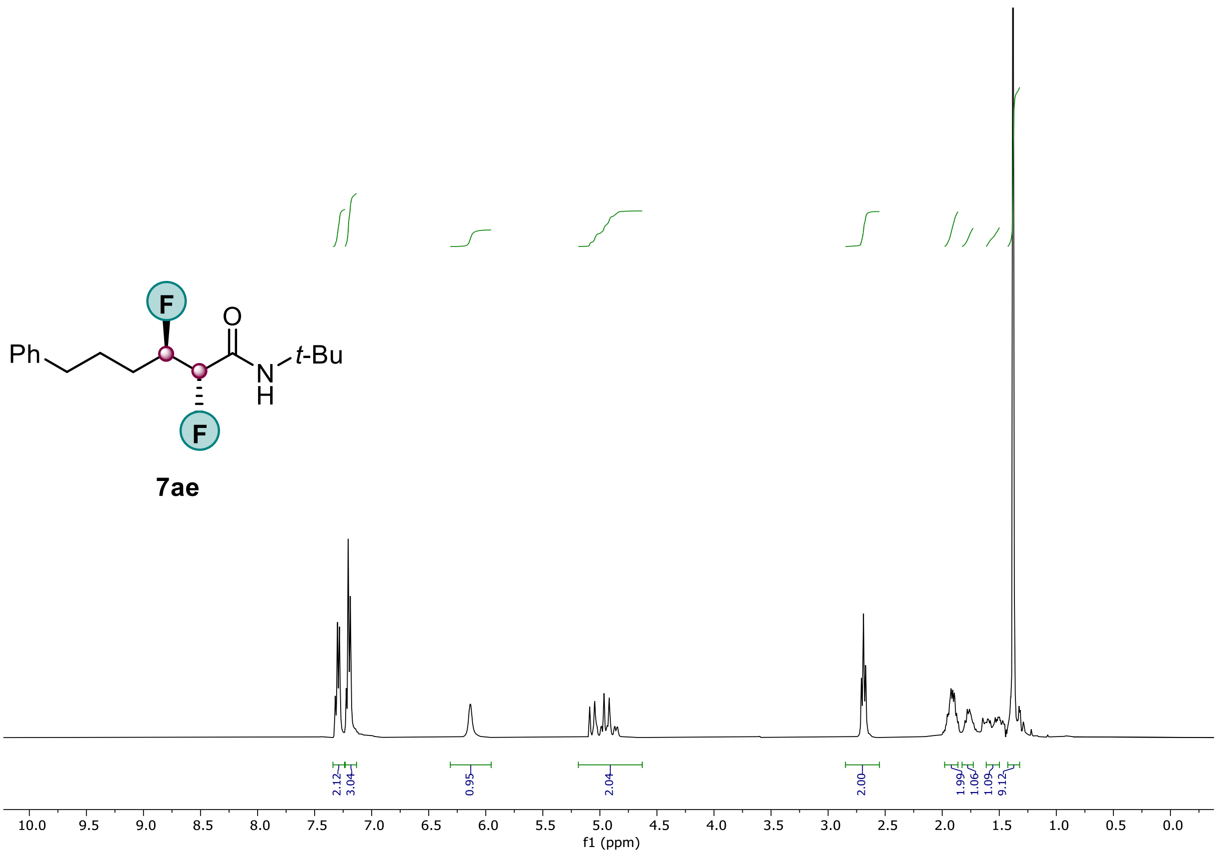
Ph
F
F
O
N
H
t-Bu
7ae
2.12
3.04
0.95
2.04
2.00
1.99
1.06
1.09
9.12
f1 (ppm)

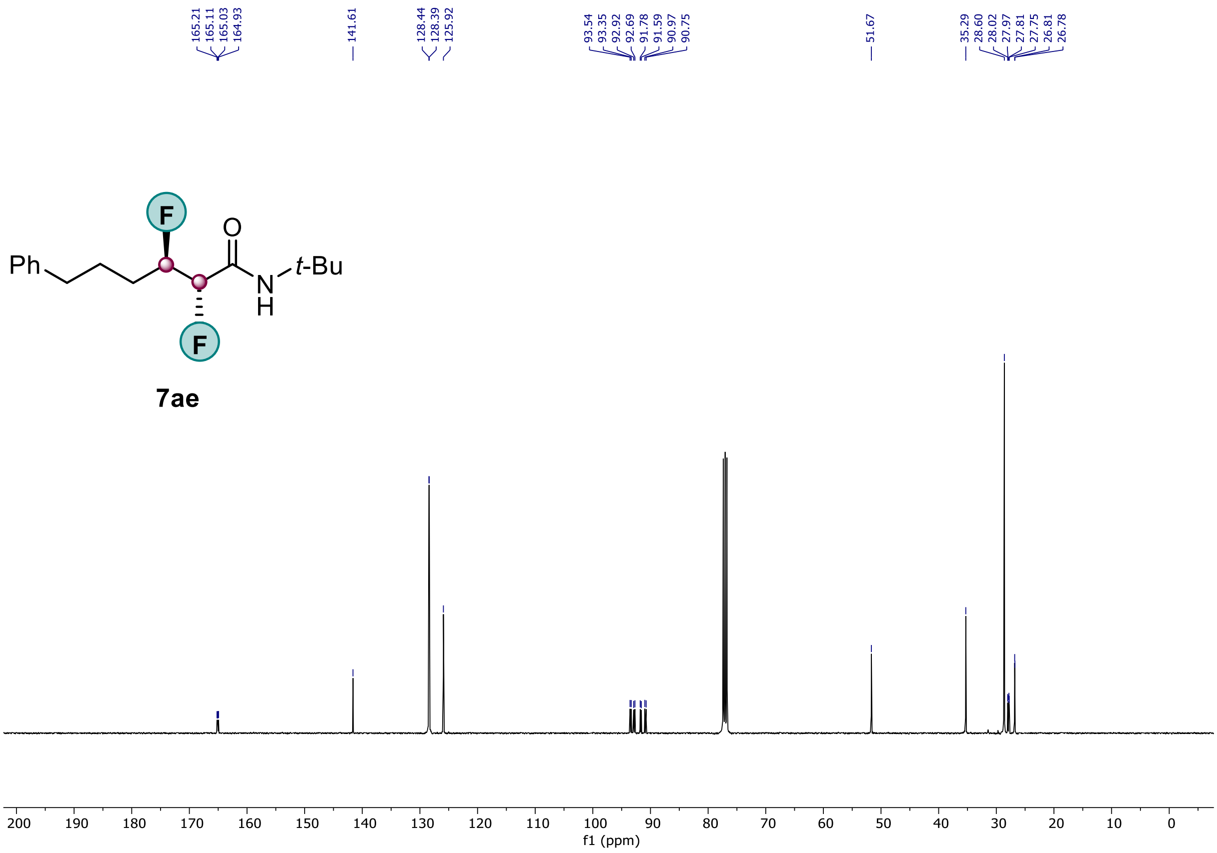
165.21
165.11
165.03
164.93
141.61
128.44
128.39
125.92
93.54
93.35
92.92
92.69
91.78
91.59
90.97
90.75
51.67
35.29
28.60
28.02
27.97
27.81
27.75
26.81
26.78
Ph
F
F
O
N
H
t-Bu
7ae
f1 (ppm)

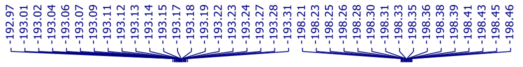
-192.97
-193.01
-193.02
-193.04
-193.06
-193.07
-193.09
-193.11
-193.12
-193.13
-193.14
-193.15
-193.17
-193.18
-193.19
-193.22
-193.23
-193.24
-193.27
-193.28
-193.31
-198.21
-198.23
-198.25
-198.26
-198.28
-198.30
-198.31
-198.33
-198.35
-198.36
-198.38
-198.39
-198.41
-198.43
-198.45
-198.46

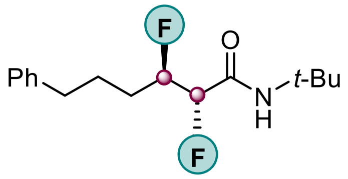
F
O
Ph
N
H
t-Bu
F

7ae

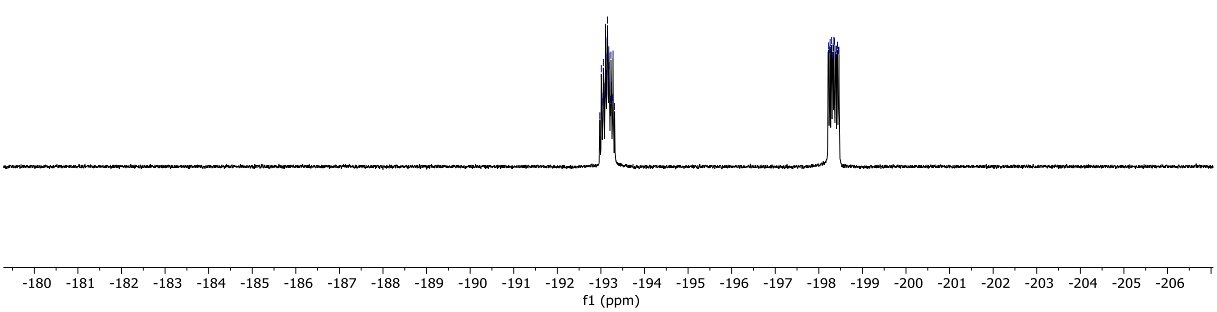
-180 -181 -182 -183 -184 -185 -186 -187 -188 -189 -190 -191 -192 -193 -194 -195 -196 -197 -198 -199 -200 -201 -202 -203 -204 -205 -206
f1 (ppm)

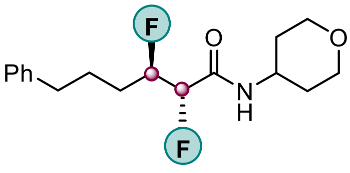

7af

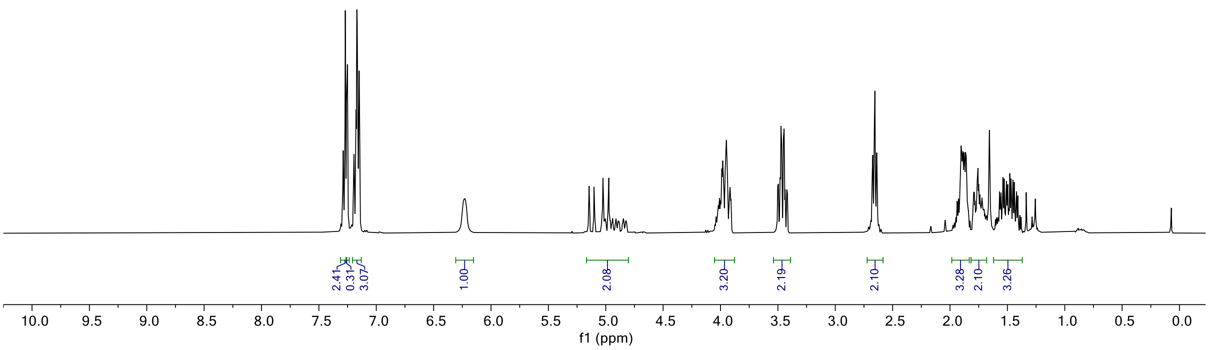

2.41
0.31
3.07
1.00
2.08
3.20
2.19
2.10
3.28
2.10
3.26
10.0 9.5 9.0 8.5 8.0 7.5 7.0 6.5 6.0 5.5 5.0 4.5 4.0 3.5 3.0 2.5 2.0 1.5 1.0 0.5 0.0
f1 (ppm)

165.51
165.41
165.32
165.23
141.63
128.57
128.54
128.51
126.06
93.52
93.33
93.06
92.83
91.76
91.56
91.13
90.90
66.73
66.69
45.68
35.37
32.98
32.82
28.26
28.20
28.04
27.98
26.88
26.85

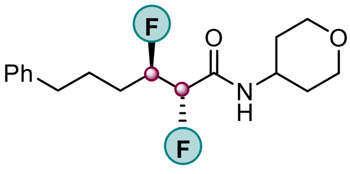

7af

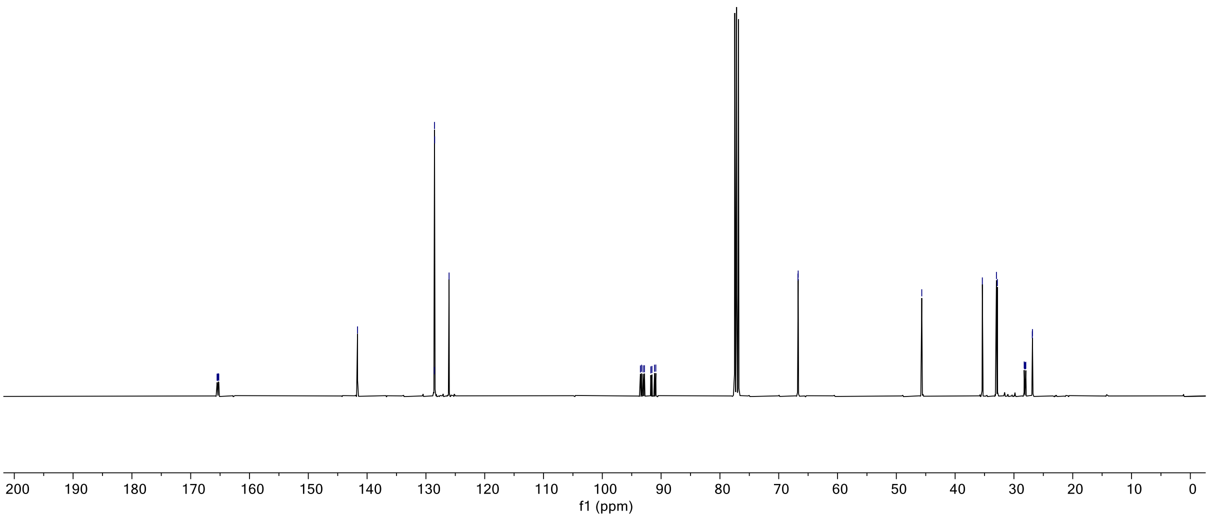

200 190 180 170 160 150 140 130 120 110 100 90 80 70 60 50 40 30 20 10 0
f1 (ppm)

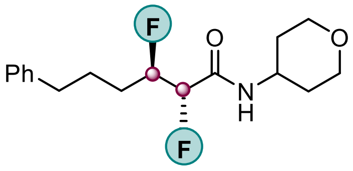

Ph
F
O
N
H
O
F

7af

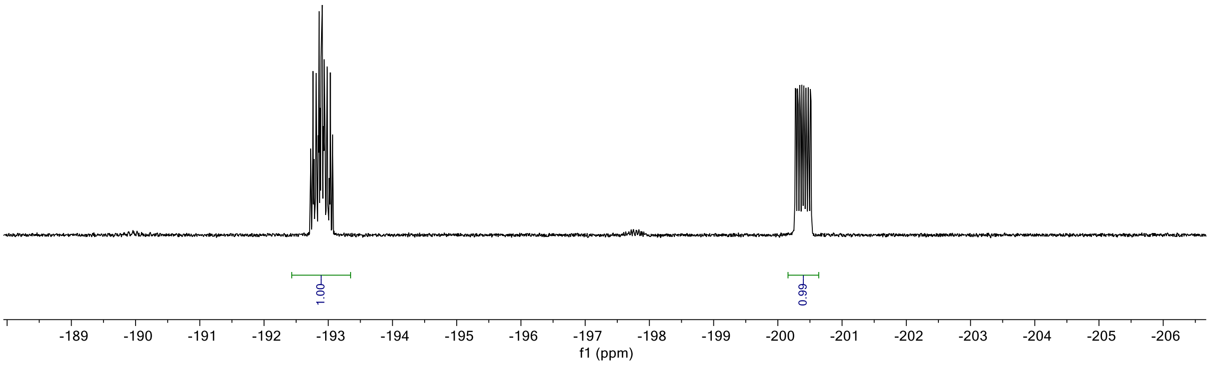

1.00
0.99
-189
-190
-191
-192
-193
-194
-195
-196
-197
-198
-199
-200
-201
-202
-203
-204
-205
-206
f1 (ppm)

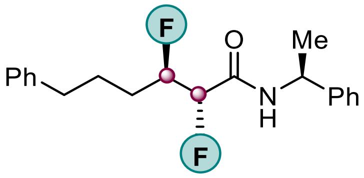

7ag

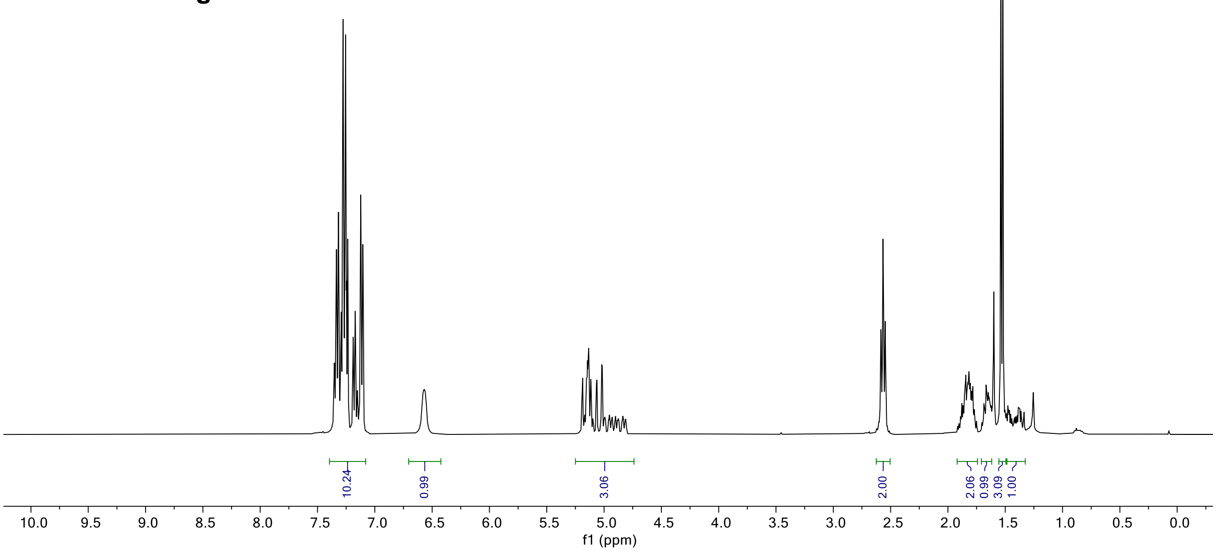

10.24
0.99
3.06
2.00
2.06
0.99
3.09
1.00
10.0 9.5 9.0 8.5 8.0 7.5 7.0 6.5 6.0 5.5 5.0 4.5 4.0 3.5 3.0 2.5 2.0 1.5 1.0 0.5 0.0
f1 (ppm)

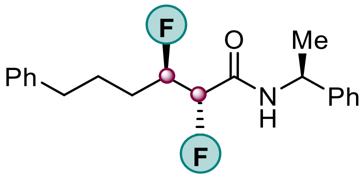

7ag

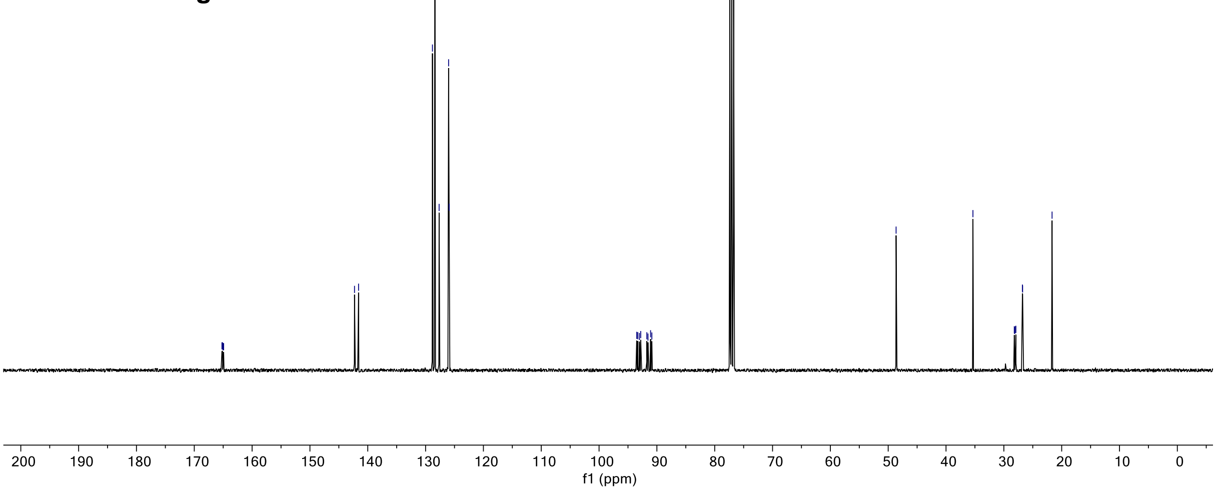

165.21
165.11
165.02
164.93
142.29
141.59
128.81
128.38
127.66
126.02
125.93
93.50
93.30
93.02
92.80
91.73
91.54
91.09
90.87
48.63
35.35
28.19
28.14
27.98
27.92
26.79
26.75
21.66
200 190 180 170 160 150 140 130 120 110 100 90 80 70 60 50 40 30 20 10 0
f1 (ppm)

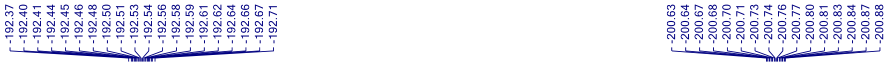
-192.37
-192.40
-192.41
-192.44
-192.45
-192.46
-192.48
-192.50
-192.51
-192.53
-192.54
-192.56
-192.58
-192.59
-192.61
-192.62
-192.64
-192.66
-192.67
-192.71
-200.63
-200.64
-200.67
-200.68
-200.70
-200.71
-200.73
-200.74
-200.76
-200.77
-200.80
-200.81
-200.83
-200.84
-200.87
-200.88

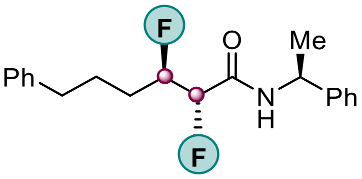
F
O
Me
Ph
N
H
Ph
F

7ag

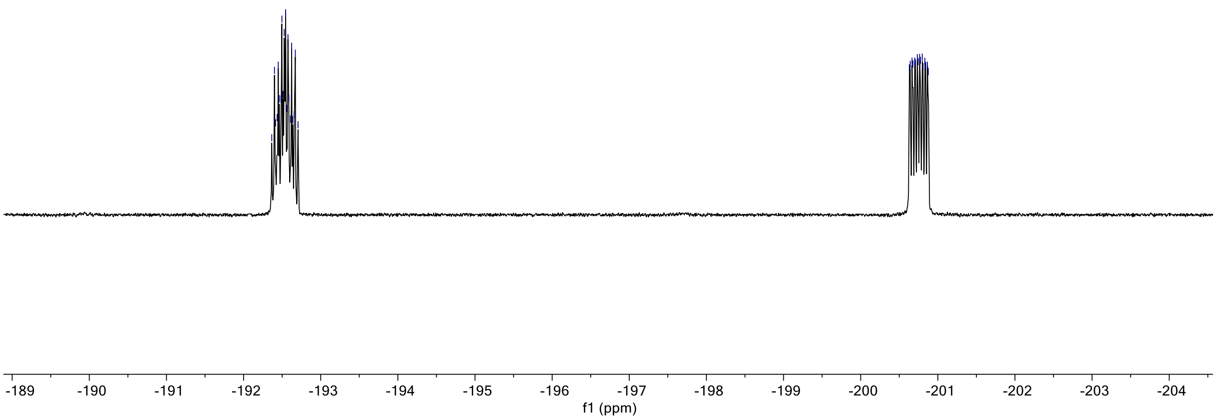
-189
-190
-191
-192
-193
-194
-195
-196
-197
-198
-199
-200
-201
-202
-203
-204
f1 (ppm)

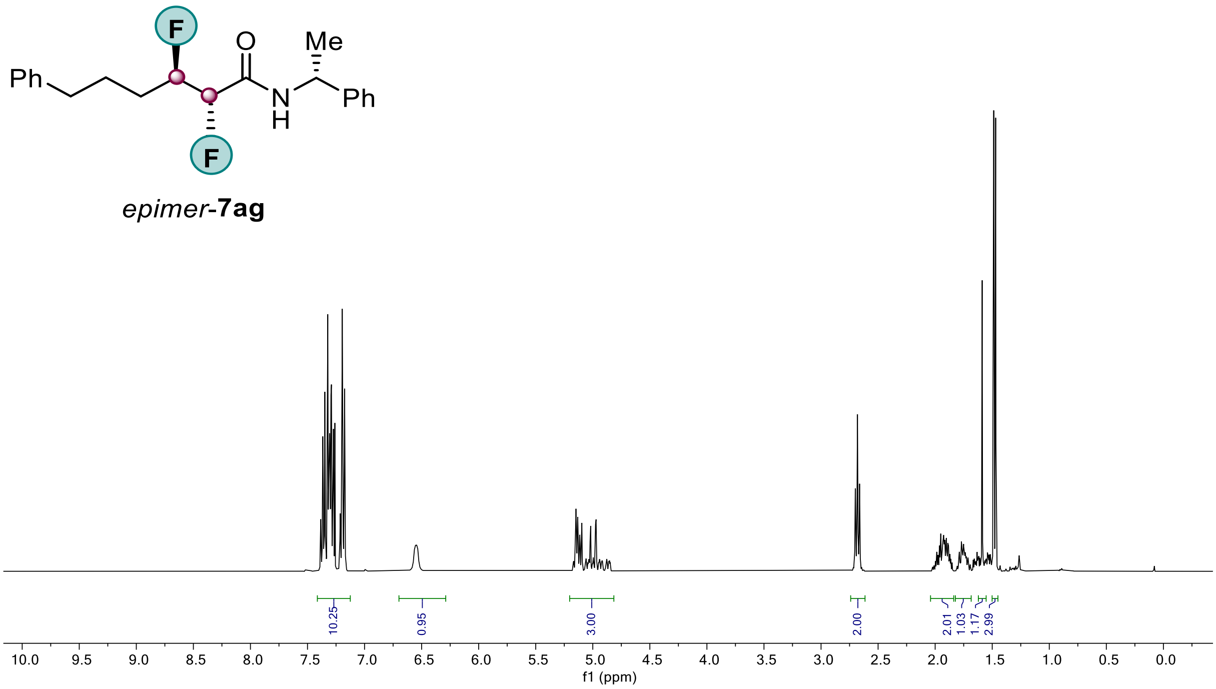
F
O
Me
Ph
N
H
Ph
F
epimer-7ag
10.25
0.95
3.00
2.00
2.01
1.03
1.17
2.99
f1 (ppm)

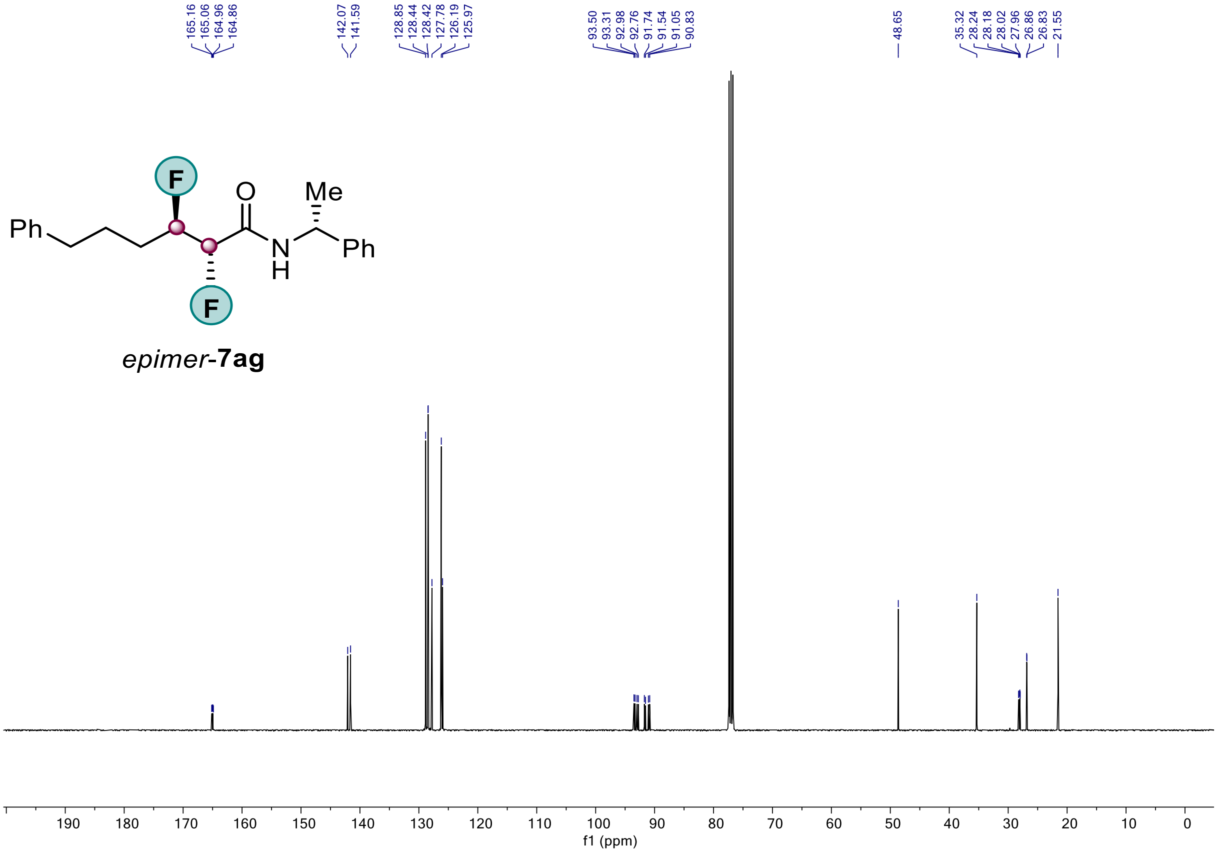
165.16
165.06
164.96
164.86
142.07
141.59
128.85
128.44
128.42
127.78
126.19
125.97
93.50
93.31
92.98
92.76
91.74
91.54
91.05
90.83
48.65
35.32
28.24
28.18
28.02
27.96
26.86
26.83
21.55
F
O
Me
Ph
N
H
Ph
F
epimer-7ag
f1 (ppm)

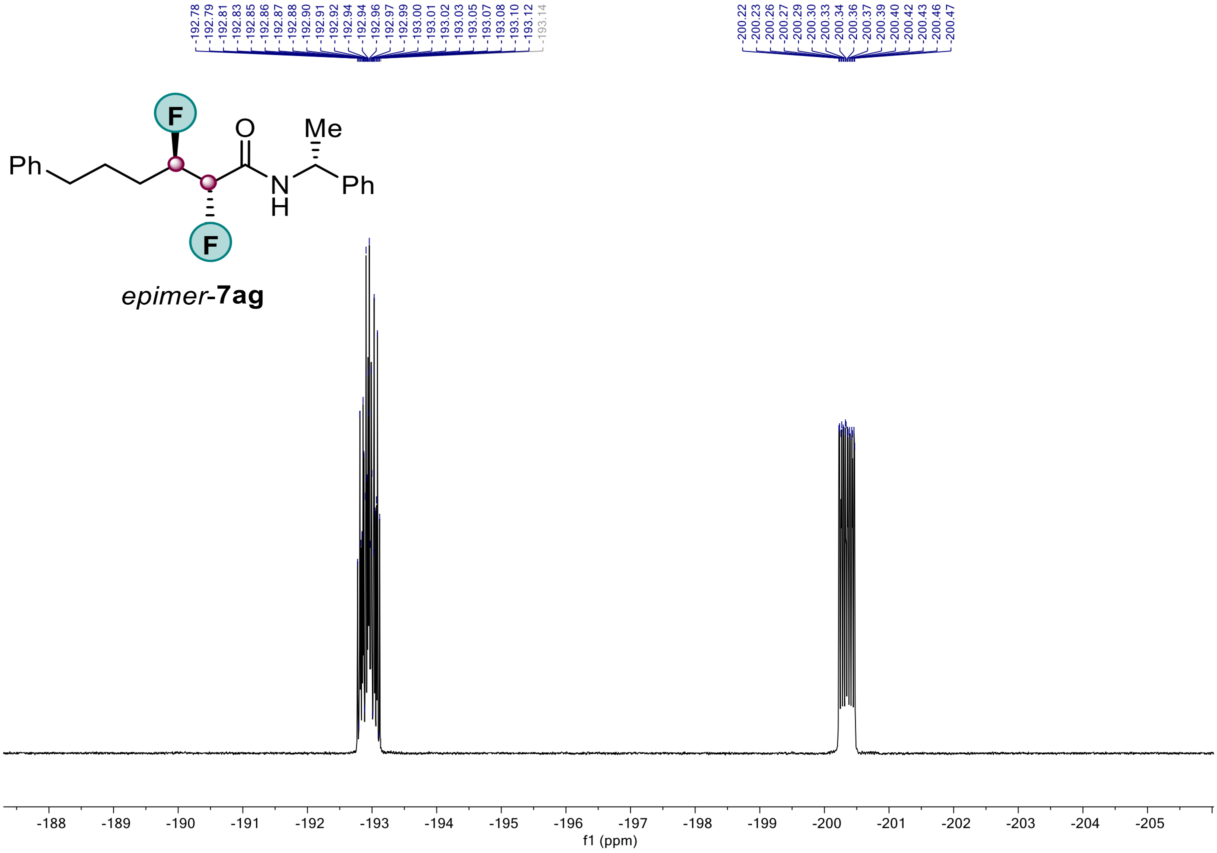
-192.78
-192.79
-192.81
-192.83
-192.85
-192.86
-192.87
-192.88
-192.90
-192.91
-192.92
-192.94
-192.94
-192.96
-192.97
-192.99
-193.00
-193.01
-193.02
-193.03
-193.05
-193.07
-193.08
-193.10
-193.12
-193.14
-200.22
-200.23
-200.26
-200.27
-200.29
-200.30
-200.33
-200.34
-200.36
-200.37
-200.39
-200.40
-200.42
-200.43
-200.46
-200.47
Ph
F
O
Me
N
H
Ph
F
epimer-7ag
-188
-189
-190
-191
-192
-193
-194
-195
-196
-197
-198
-199
-200
-201
-202
-203
-204
-205
f1 (ppm)

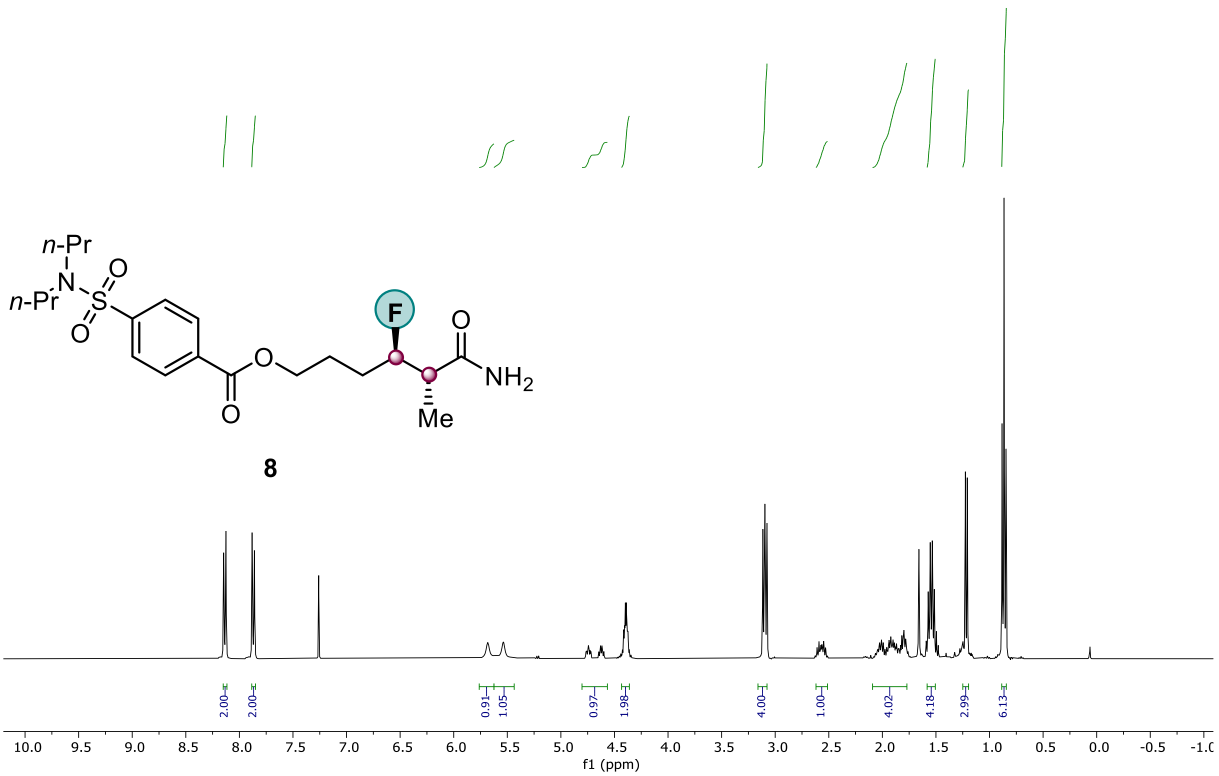
n-Pr
n-Pr
N
S
O
O
O
O
F
O
NH2
Me
8
2.00
2.00
0.91
1.05
0.97
1.98
4.00
1.00
4.02
4.18
2.99
6.13
f1 (ppm)

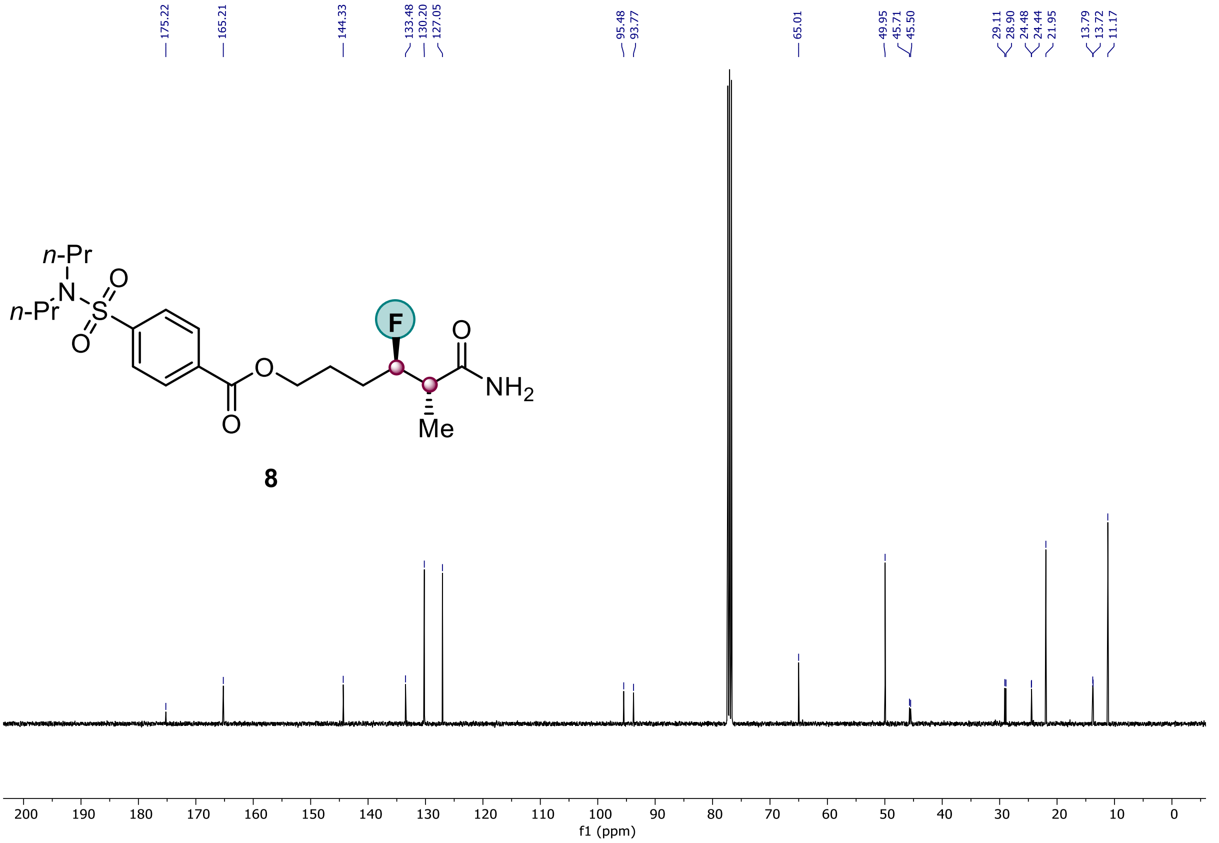
175.22
165.21
144.33
133.48
130.20
127.05
95.48
93.77
65.01
49.95
45.71
45.50
29.11
28.90
24.48
24.44
21.95
13.79
13.72
11.17
n-Pr
n-Pr
N
S
O
O
O
O
F
O
NH2
Me
8
f1 (ppm)

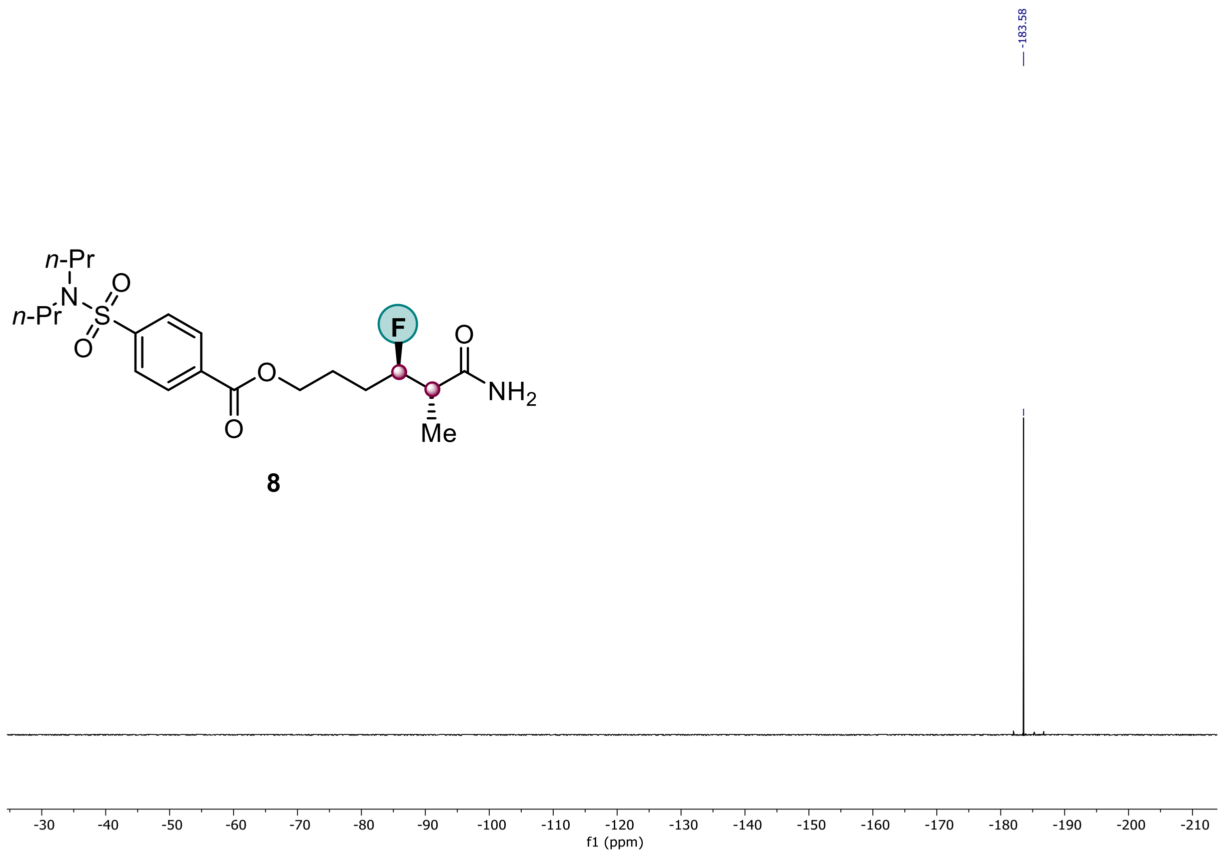
-183.58
n-Pr
n-Pr
N
S
O
O
O
O
F
O
NH2
Me
8
-30 -40 -50 -60 -70 -80 -90 -100 -110 -120 -130 -140 -150 -160 -170 -180 -190 -200 -210
f1 (ppm)

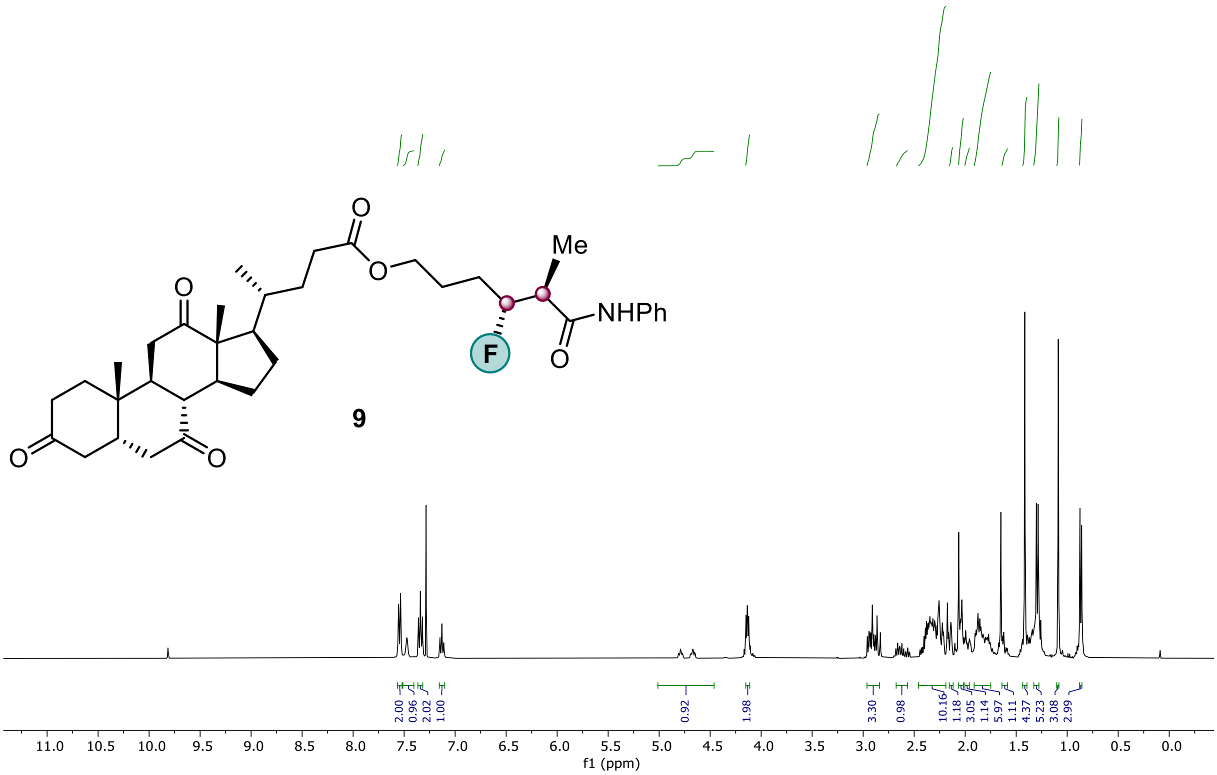
Me
NHPh
F
9
2.00
0.96
2.02
1.00
0.92
1.98
3.30
0.98
10.16
1.18
3.05
1.14
5.97
1.11
4.37
5.23
3.08
2.99
f1 (ppm)

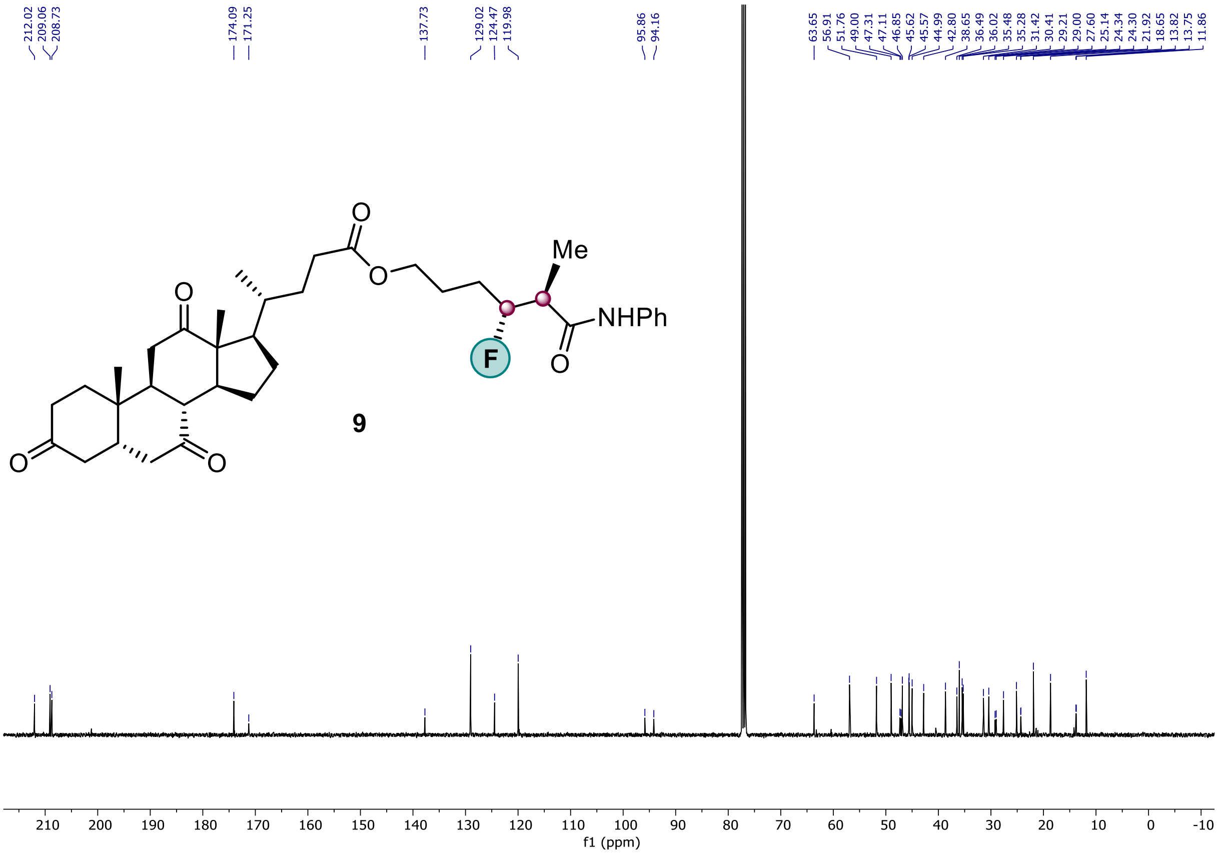
212.02
209.06
208.73
174.09
171.25
137.73
129.02
124.47
119.98
95.86
94.16
63.65
56.91
51.76
49.00
47.31
47.11
46.85
45.62
45.57
44.99
42.80
38.65
36.49
36.02
35.48
35.28
31.42
30.41
29.21
29.00
27.60
25.14
24.34
24.30
21.92
18.65
13.82
13.75
11.86
Me
NHPh
F
9
f1 (ppm)

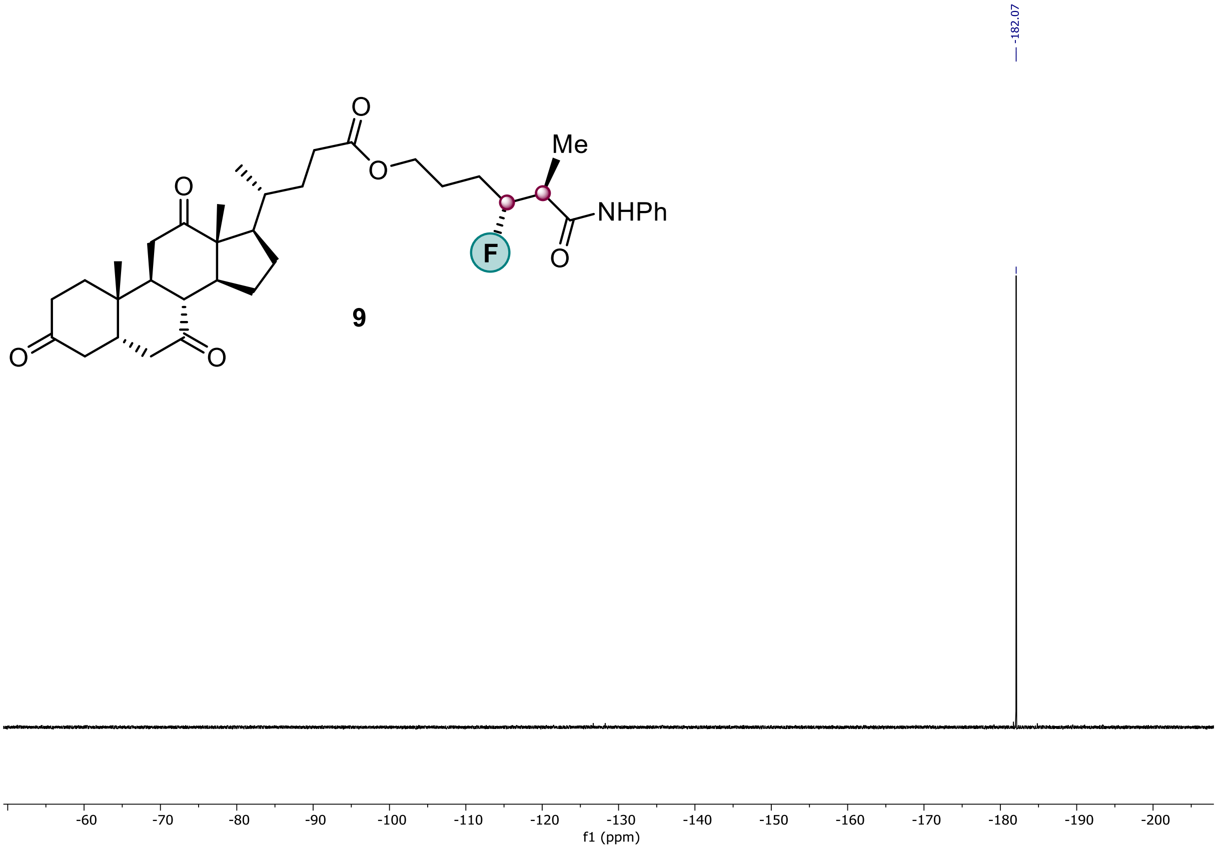
-182.07
Me
NHPh
F
9
-60
-70
-80
-90
-100
-110
-120
-130
-140
-150
-160
-170
-180
-190
-200
f1 (ppm)

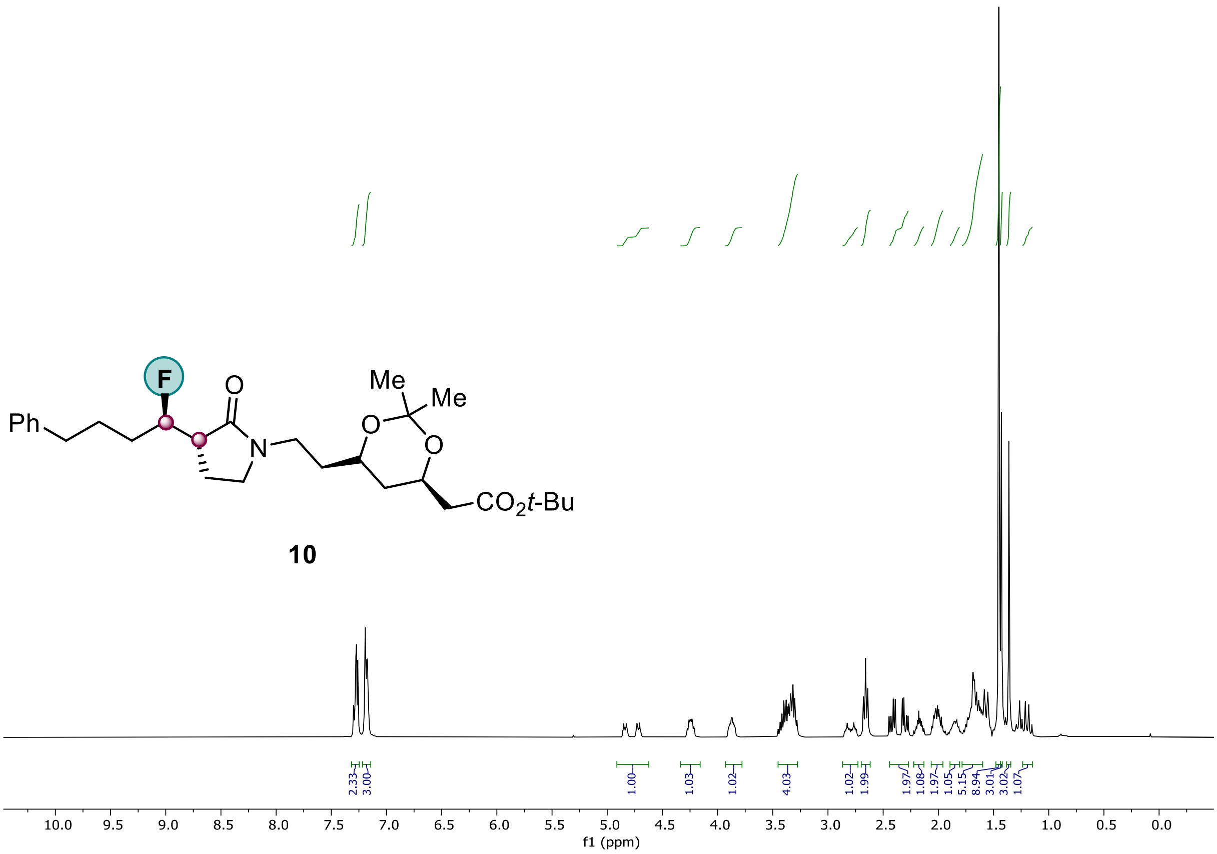
F
O
Me
Me
Ph
N
O
O
CO2t-Bu
10
2.33
3.00
1.00
1.03
1.02
4.03
1.02
1.99
1.97
1.08
1.97
1.05
5.15
8.94
3.01
3.02
1.07
10.0 9.5 9.0 8.5 8.0 7.5 7.0 6.5 6.0 5.5 5.0 4.5 4.0 3.5 3.0 2.5 2.0 1.5 1.0 0.5 0.0
f1 (ppm)

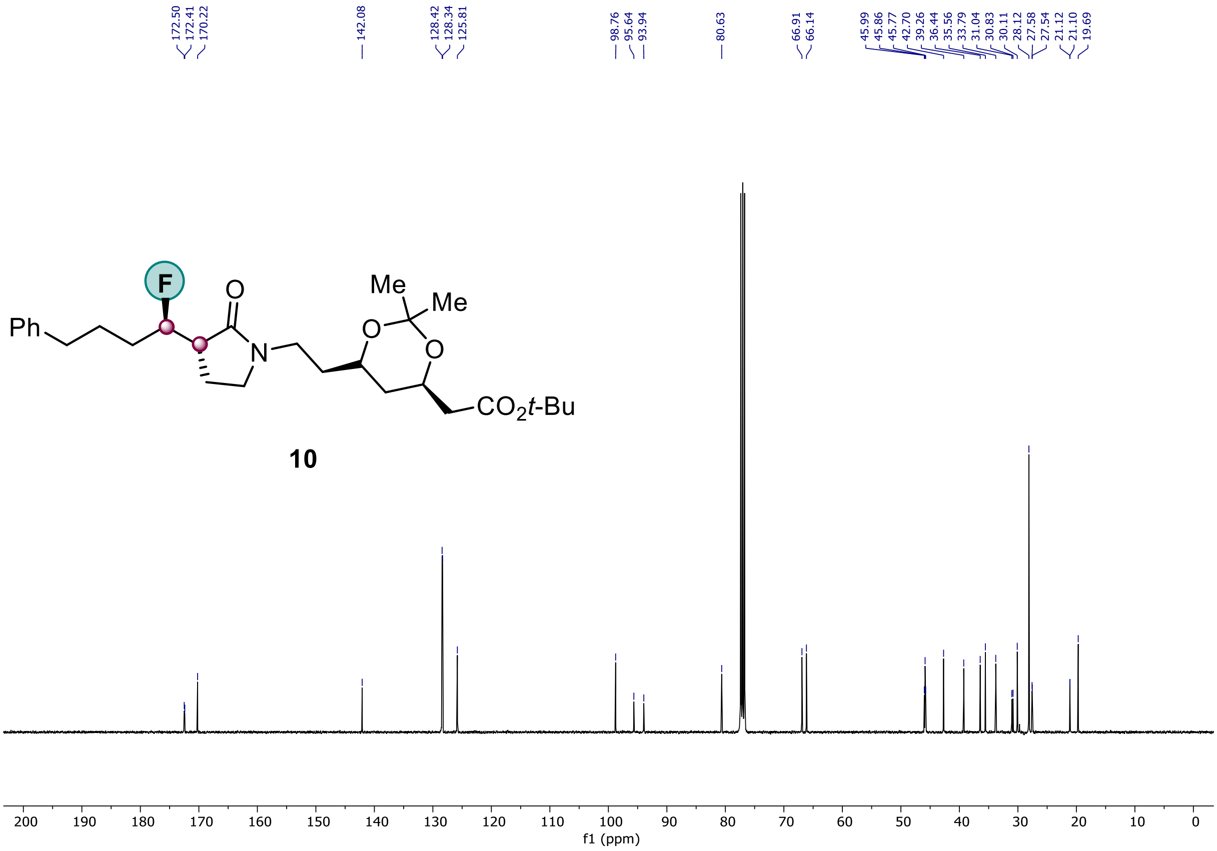
172.50
172.41
170.22
142.08
128.42
128.34
125.81
98.76
95.64
93.94
80.63
66.91
66.14
45.99
45.86
45.77
42.70
39.26
36.44
35.56
33.79
31.04
30.83
30.11
28.12
27.58
27.54
21.12
21.10
19.69
F
O
Me
Me
Ph
N
O
O
CO2t-Bu
10
200 190 180 170 160 150 140 130 120 110 100 90 80 70 60 50 40 30 20 10 0
f1 (ppm)

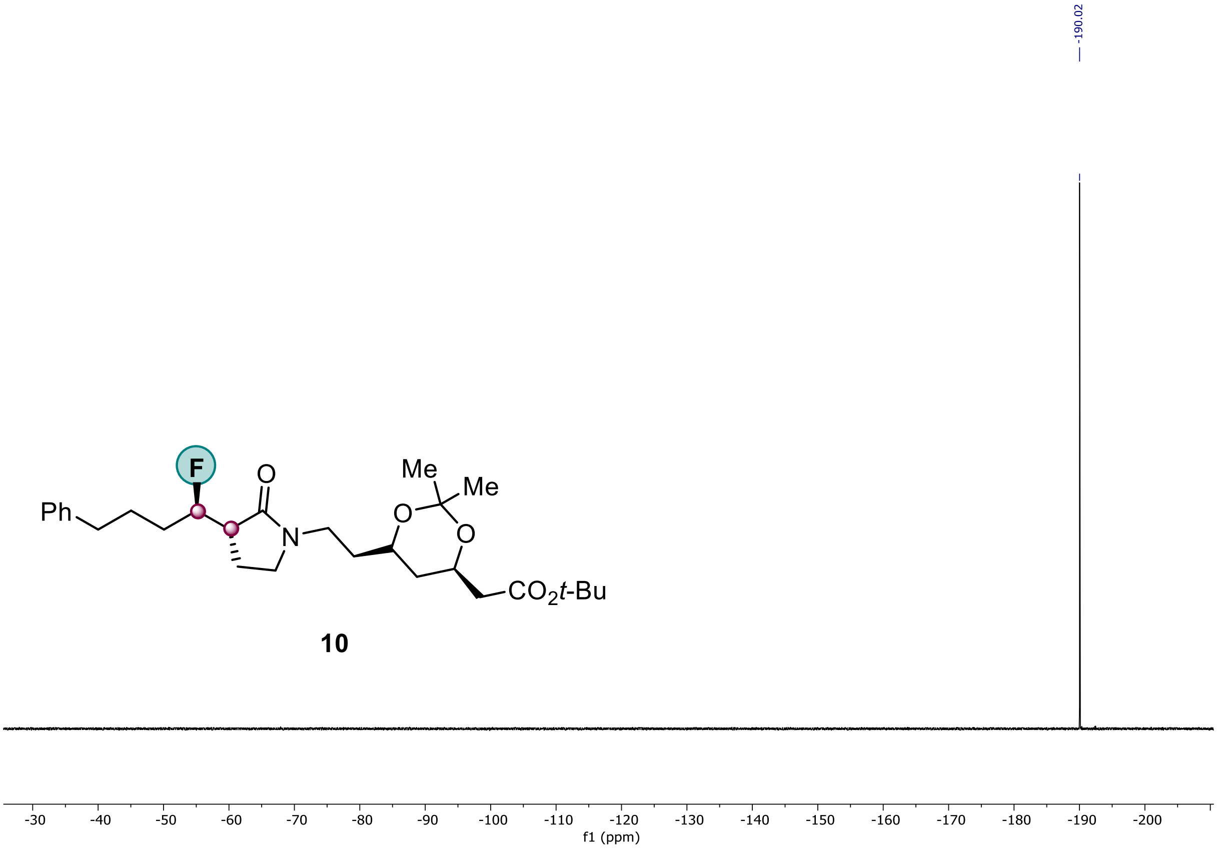

Ph
F
O
N
Me
Me
O
O
CO2t-Bu
10
-190.02
-30
-40
-50
-60
-70
-80
-90
-100
-110
-120
-130
-140
-150
-160
-170
-180
-190
-200
f1 (ppm)

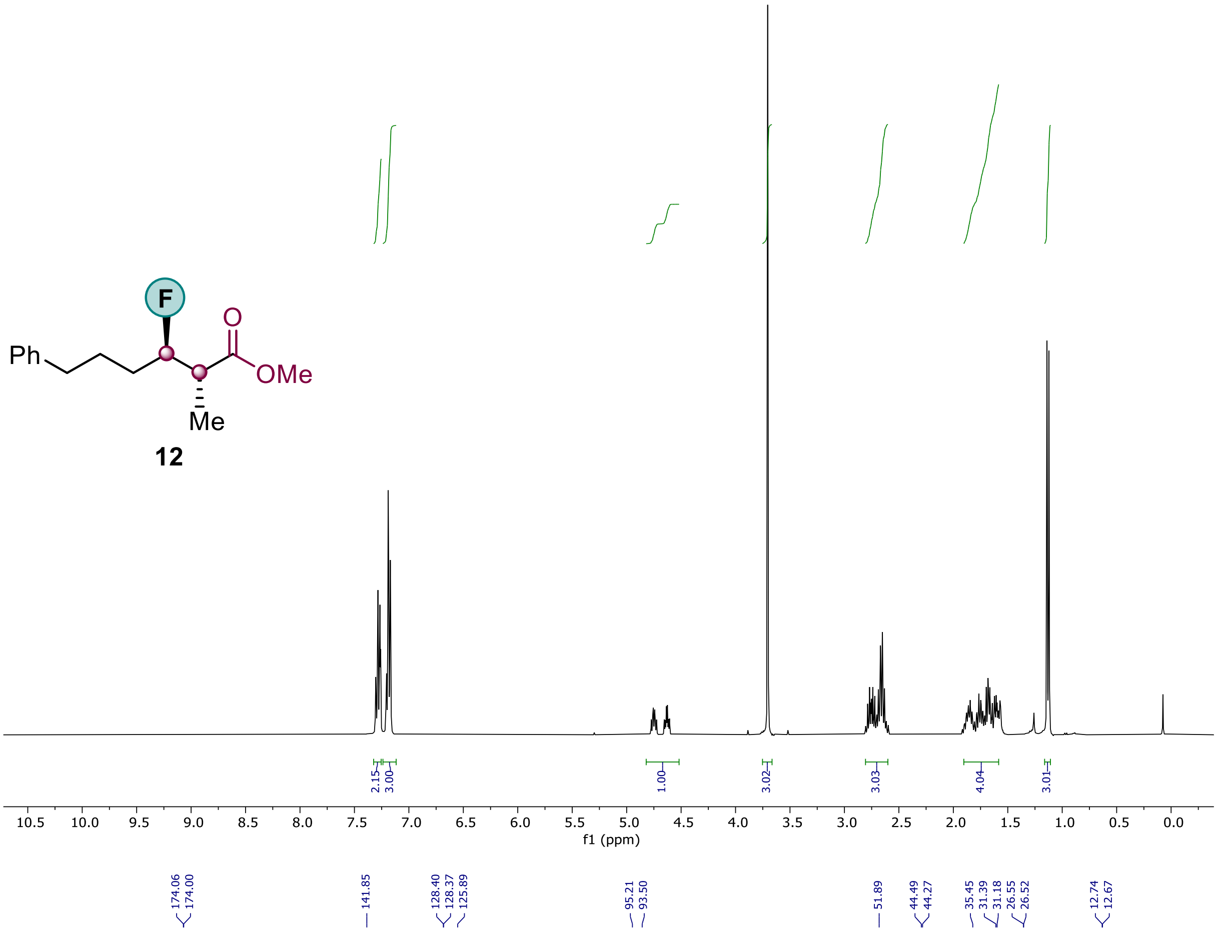
F
O
Ph
OMe
Me
12
2.15
3.00
1.00
3.02
3.03
4.04
3.01
10.5 10.0 9.5 9.0 8.5 8.0 7.5 7.0 6.5 6.0 5.5 5.0 4.5 4.0 3.5 3.0 2.5 2.0 1.5 1.0 0.5 0.0
f1 (ppm)
174.06
174.00
141.85
128.40
128.37
125.89
95.21
93.50
51.89
44.49
44.27
35.45
31.39
31.18
26.55
26.52
12.74
12.67

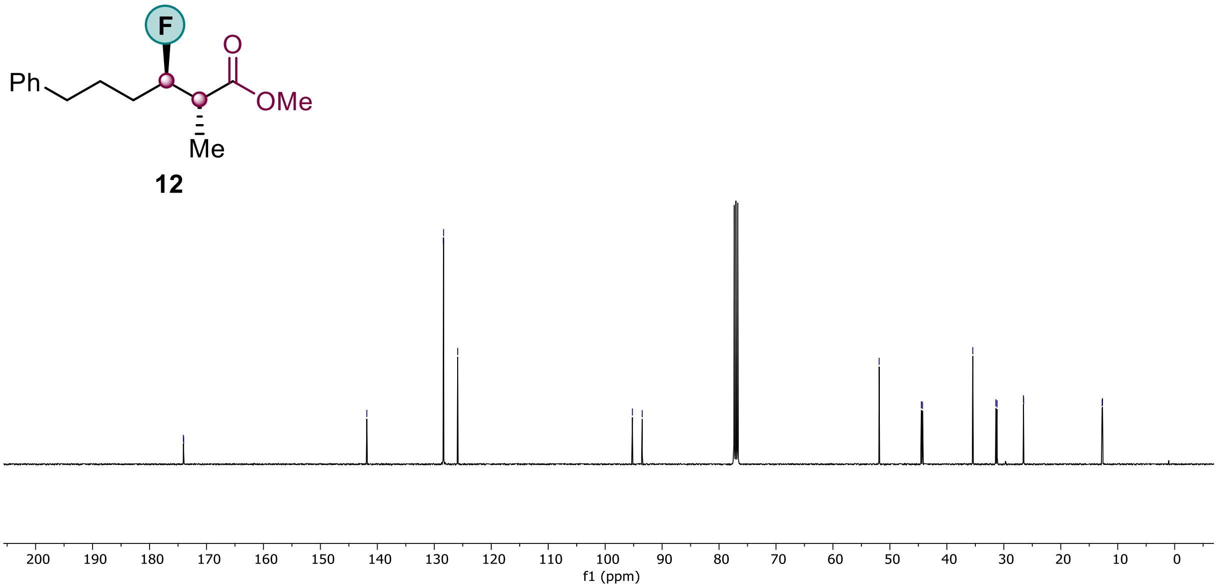
F
O
Ph
OMe
Me
12
200 190 180 170 160 150 140 130 120 110 100 90 80 70 60 50 40 30 20 10 0
f1 (ppm)

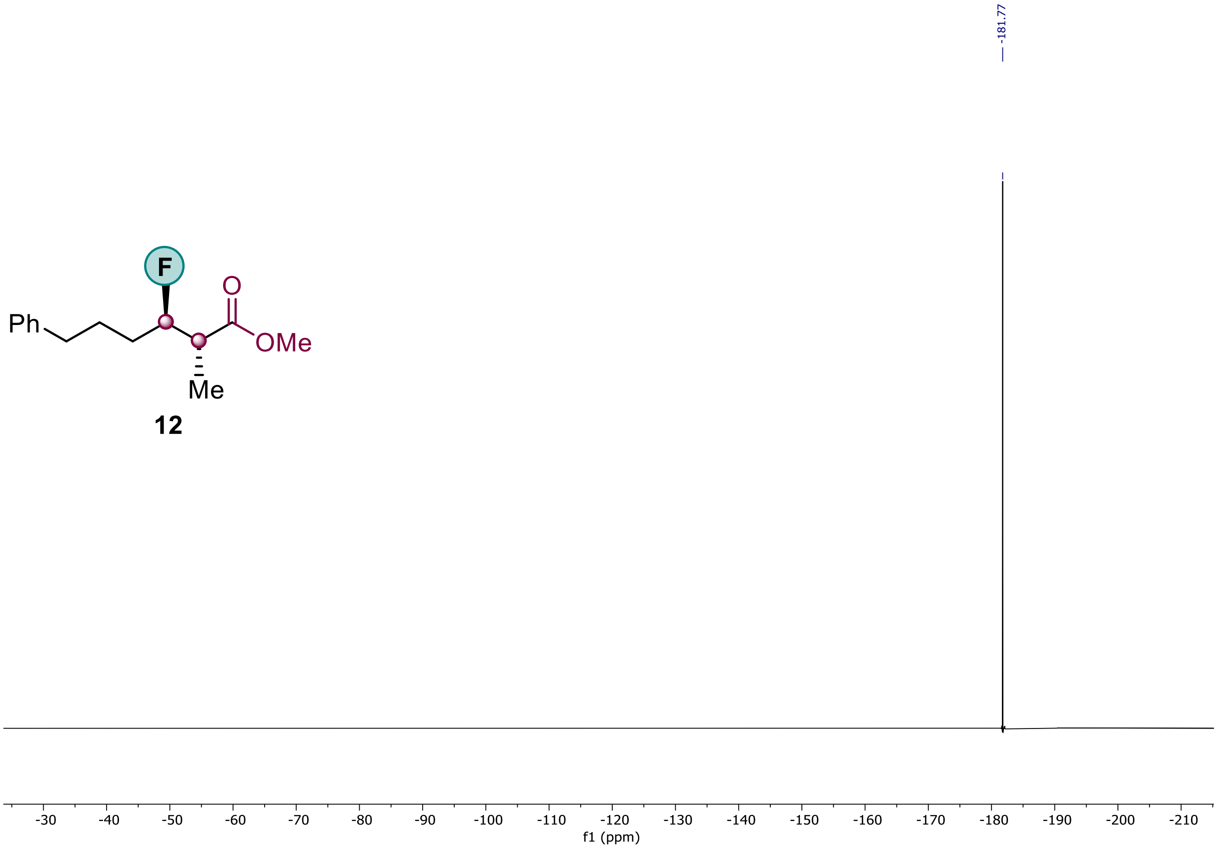
-181.77
-30
-40
-50
-60
-70
-80
-90
-100
-110
-120
-130
-140
-150
-160
-170
-180
-190
-200
-210
f1 (ppm)
Ph
F
O
OMe
Me
12

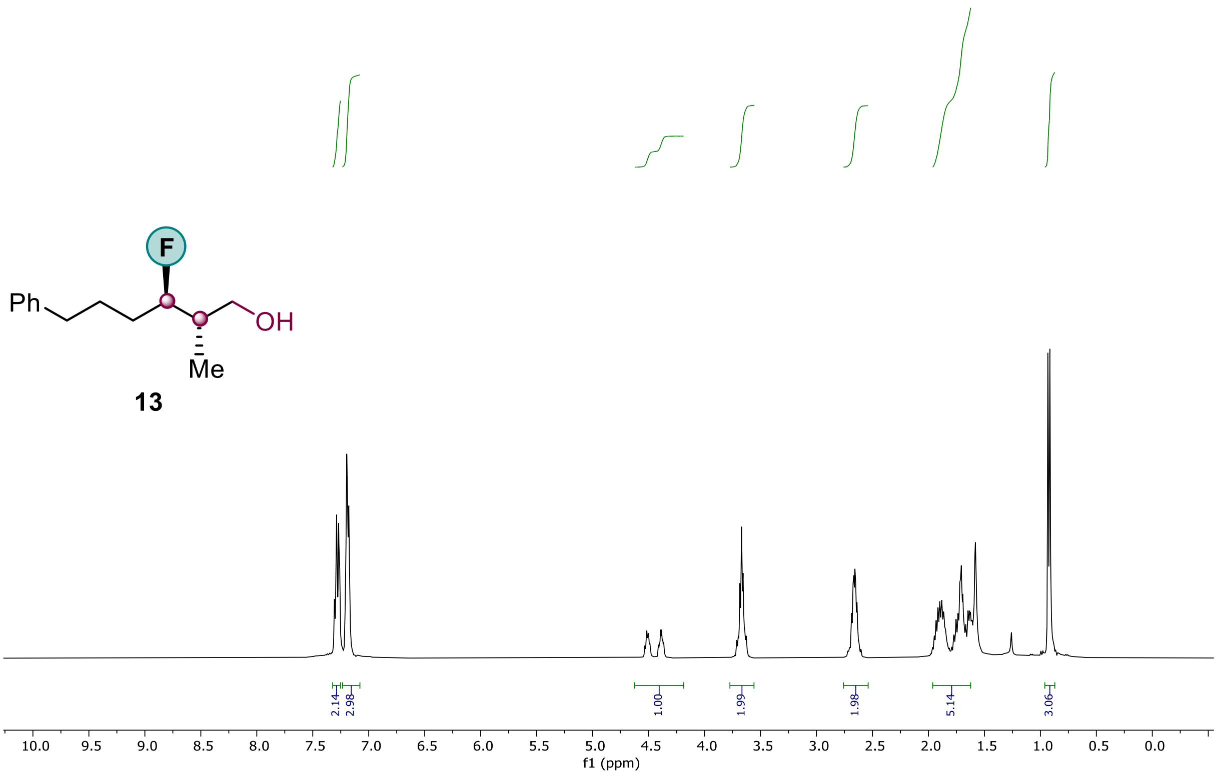
F
Ph
OH
Me
13
2.14
2.98
1.00
1.99
1.98
5.14
3.06
10.0 9.5 9.0 8.5 8.0 7.5 7.0 6.5 6.0 5.5 5.0 4.5 4.0 3.5 3.0 2.5 2.0 1.5 1.0 0.5 0.0
f1 (ppm)

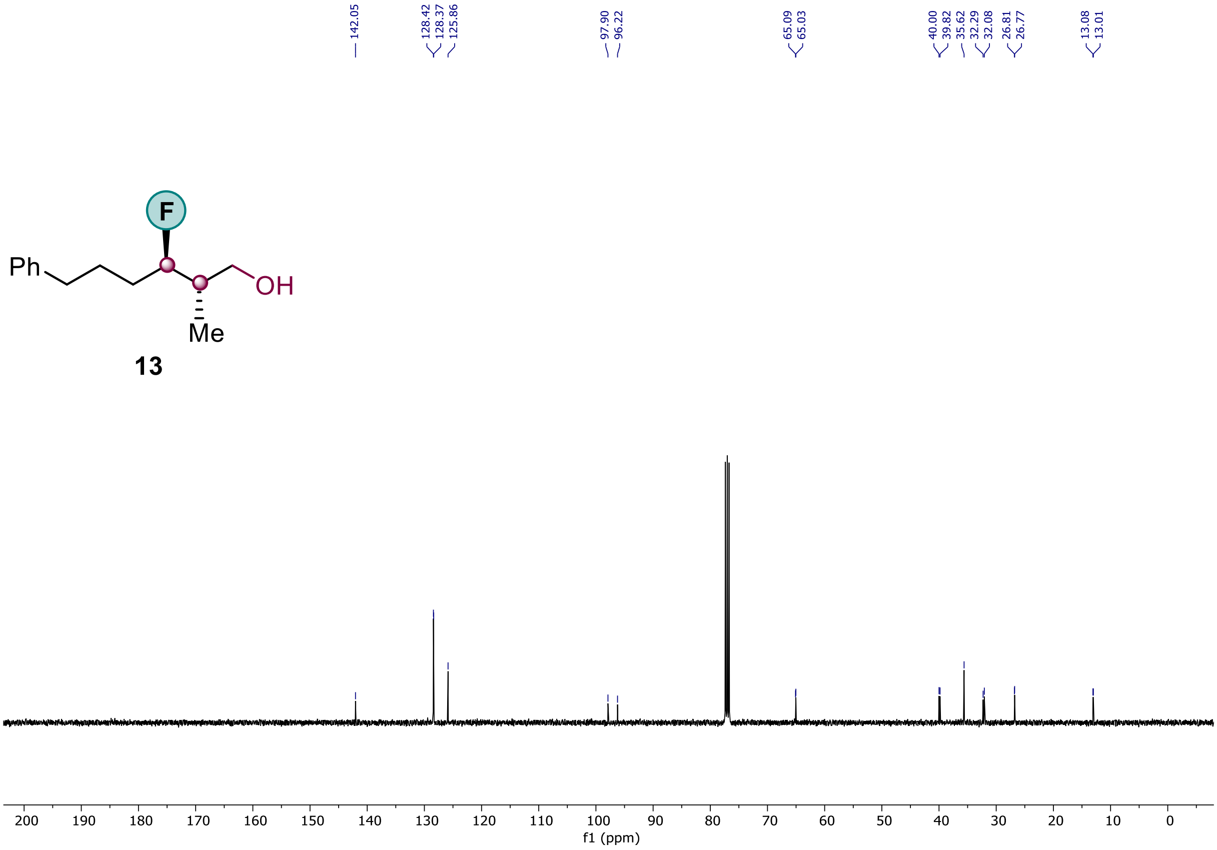
142.05
128.42
128.37
125.86
97.90
96.22
65.09
65.03
40.00
39.82
35.62
32.29
32.08
26.81
26.77
13.08
13.01
F
Ph
OH
Me
13
200 190 180 170 160 150 140 130 120 110 100 90 80 70 60 50 40 30 20 10 0
f1 (ppm)

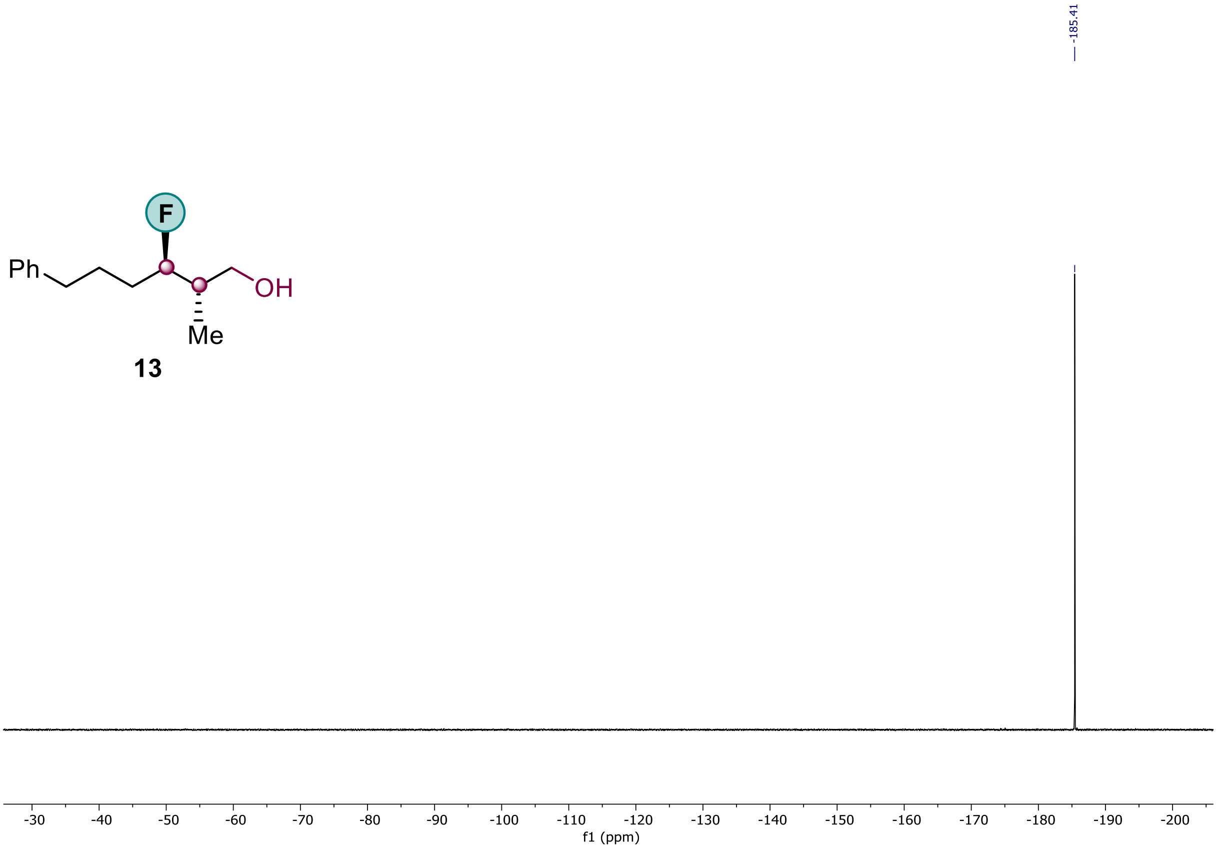

-185.41
Ph
F
OH
Me
13
-30
-40
-50
-60
-70
-80
-90
-100
-110
-120
-130
-140
-150
-160
-170
-180
-190
-200
f1 (ppm)

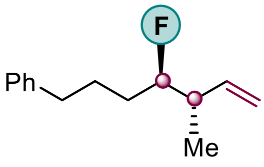
F
Ph
Me

11

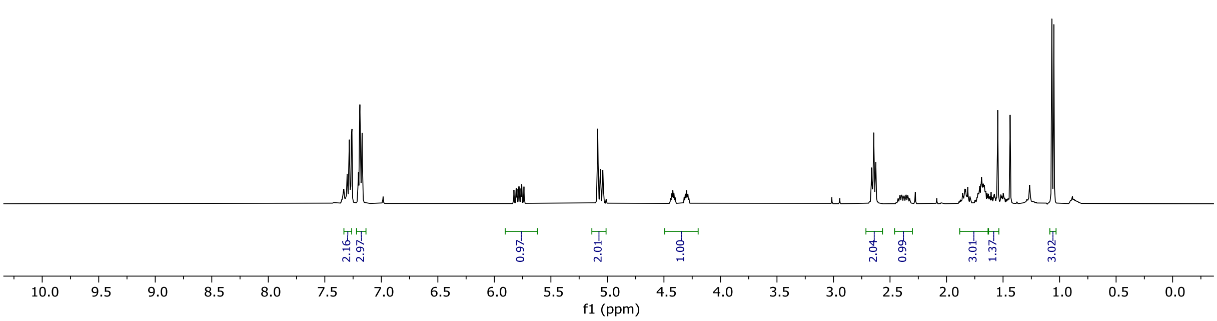
2.16
2.97
0.97
2.01
1.00
2.04
0.99
3.01
1.37
3.02
10.0 9.5 9.0 8.5 8.0 7.5 7.0 6.5 6.0 5.5 5.0 4.5 4.0 3.5 3.0 2.5 2.0 1.5 1.0 0.5 0.0
f1 (ppm)

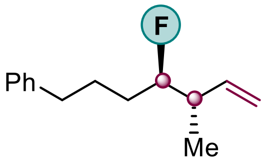
F
Ph
Me

11

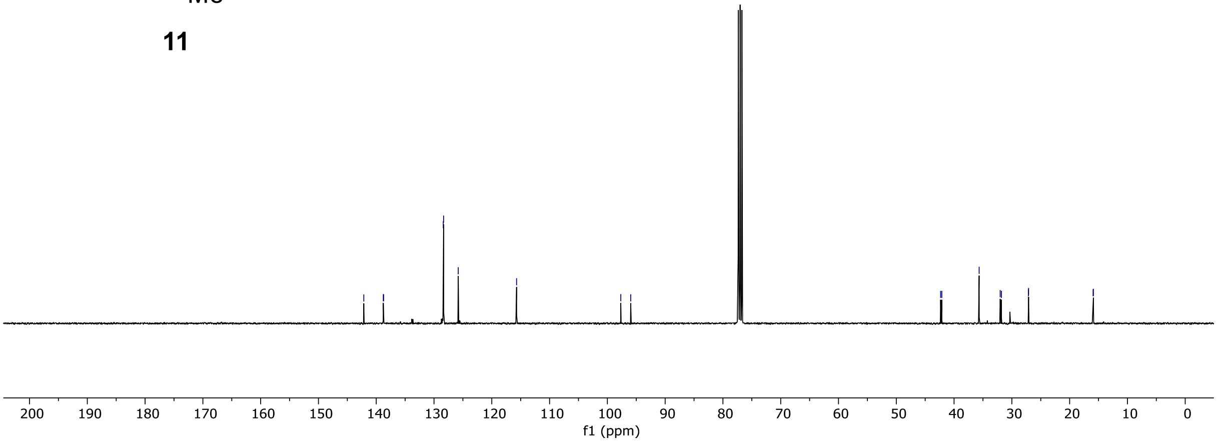
142.15
138.78
138.72
128.40
128.34
125.81
115.71
97.67
95.96
42.33
42.13
35.66
32.02
31.81
27.14
27.10
15.94
15.89
200 190 180 170 160 150 140 130 120 110 100 90 80 70 60 50 40 30 20 10 0
f1 (ppm)

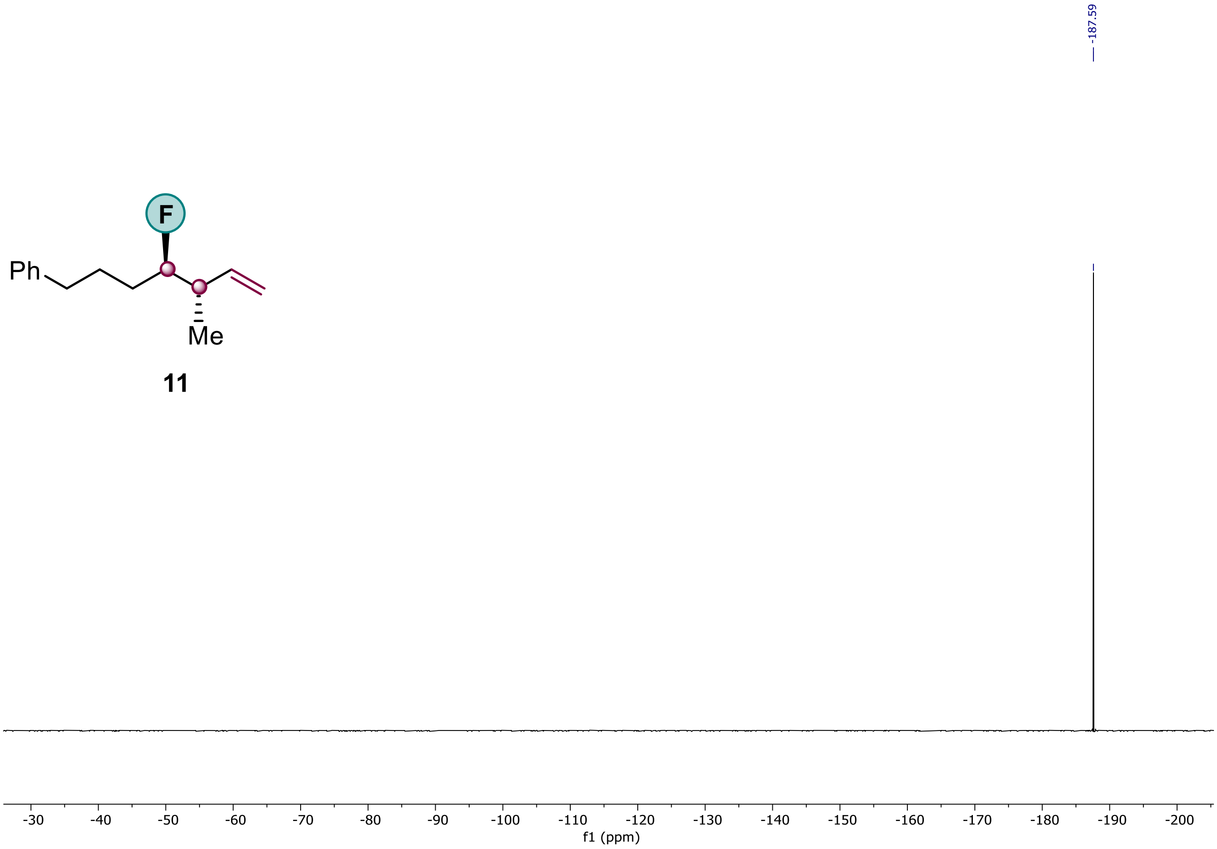
F
Ph
Me
-187.59
-30
-40
-50
-60
-70
-80
-90
-100
-110
-120
-130
-140
-150
-160
-170
-180
-190
-200
f1 (ppm)

11

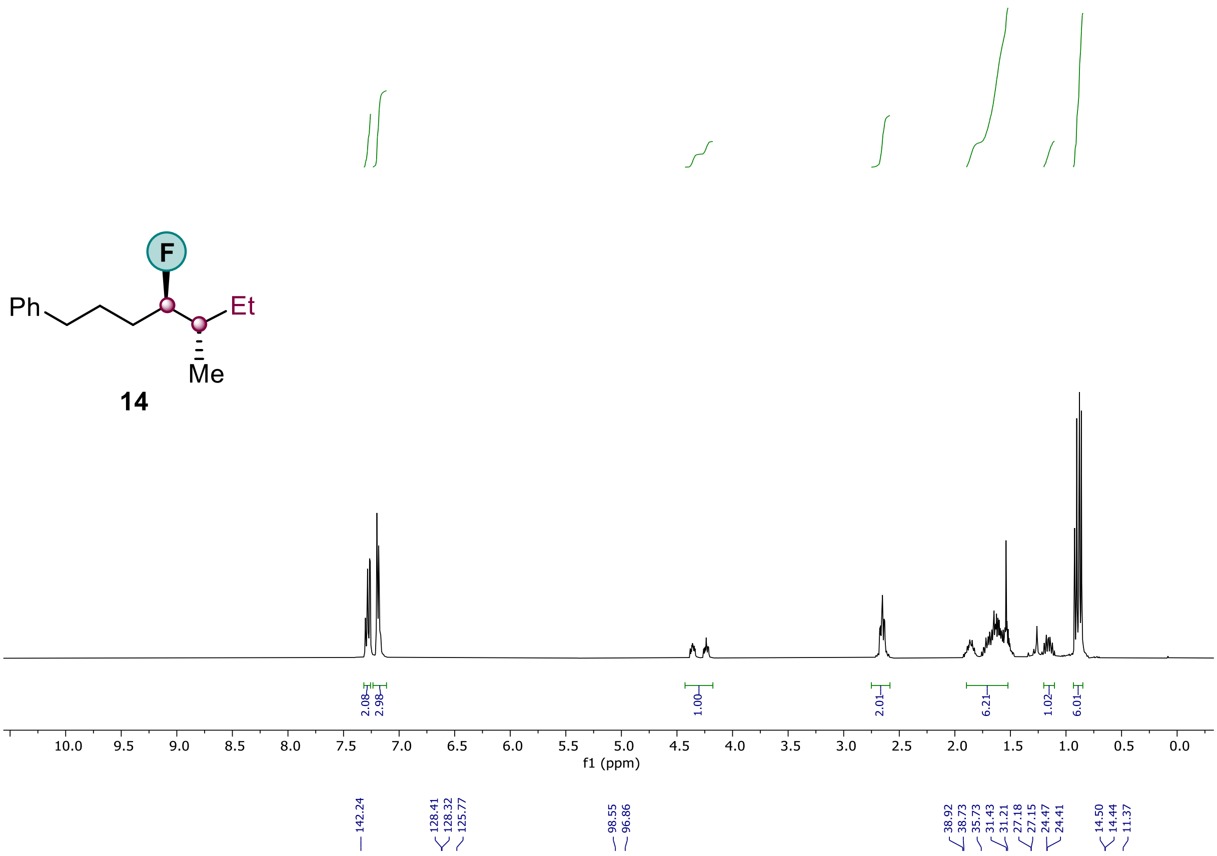
Ph
F
Et
Me
14
2.08
2.98
1.00
2.01
6.21
1.02
6.01
f1 (ppm)
142.24
128.41
128.32
125.77
98.55
96.86
38.92
38.73
35.73
31.43
31.21
27.18
27.15
24.47
24.41
14.50
14.44
11.37

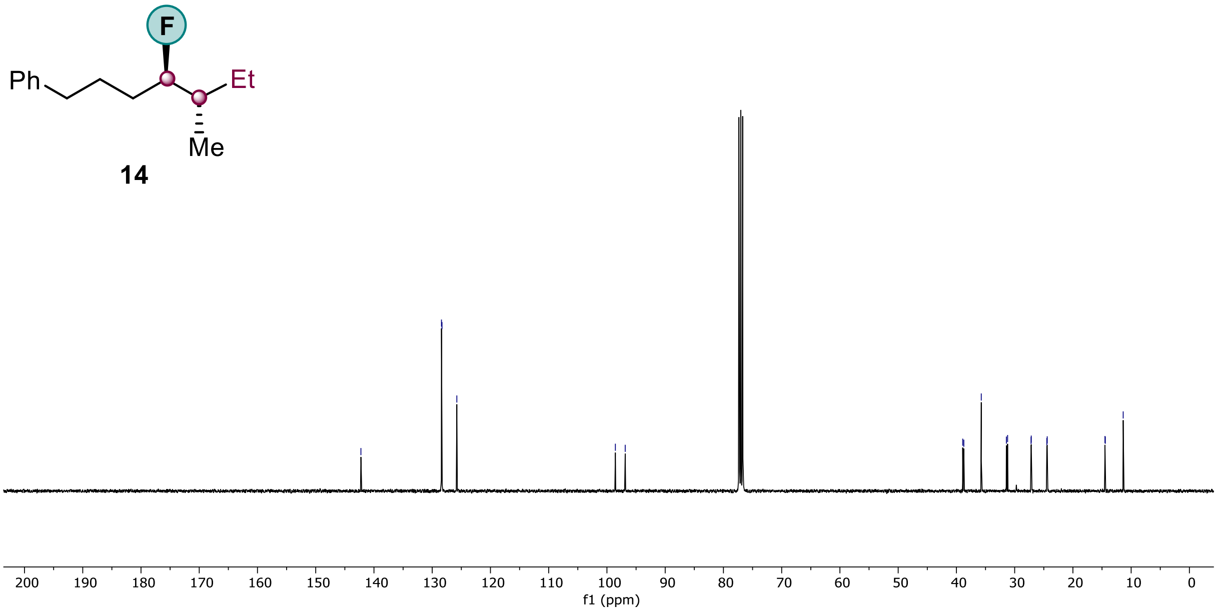
Ph
F
Et
Me
14
f1 (ppm)

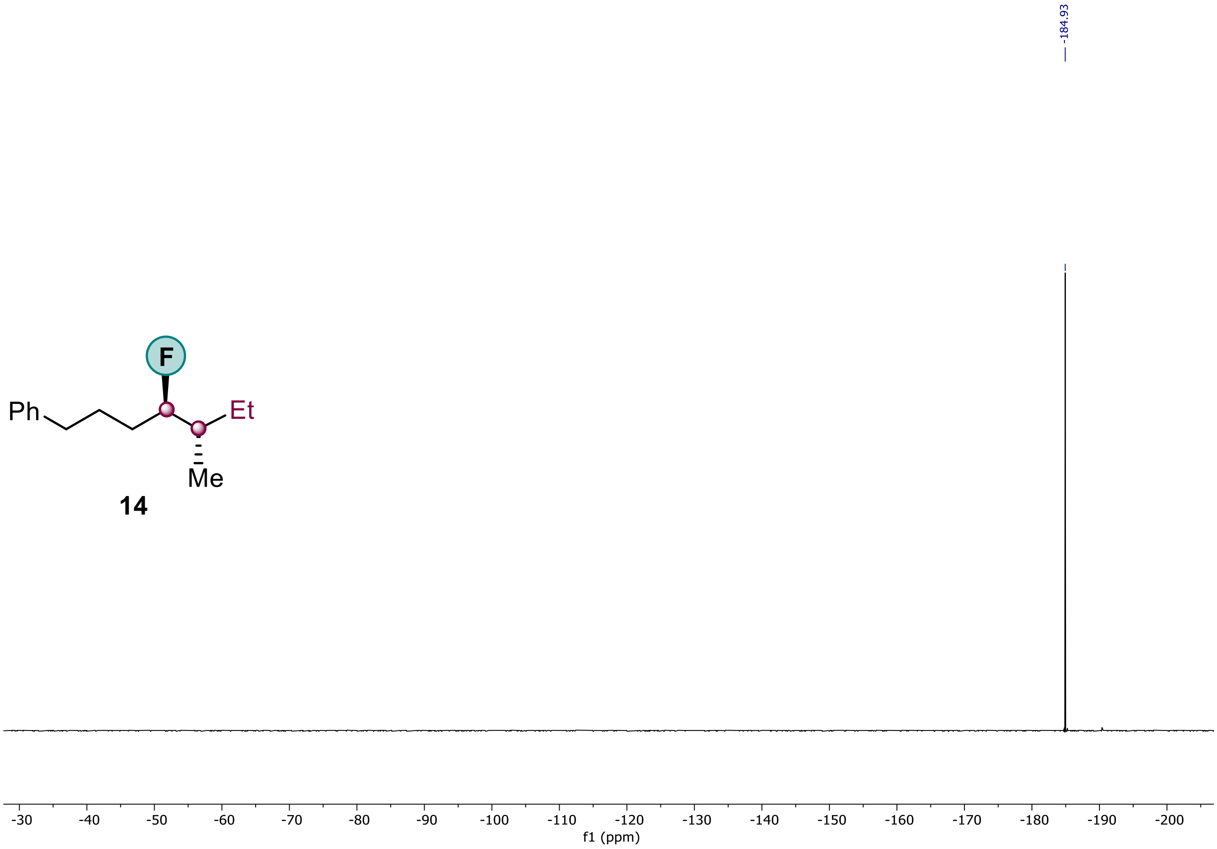

-184.93
Ph
F
Et
Me
14
-30 -40 -50 -60 -70 -80 -90 -100 -110 -120 -130 -140 -150 -160 -170 -180 -190 -200
f1 (ppm)

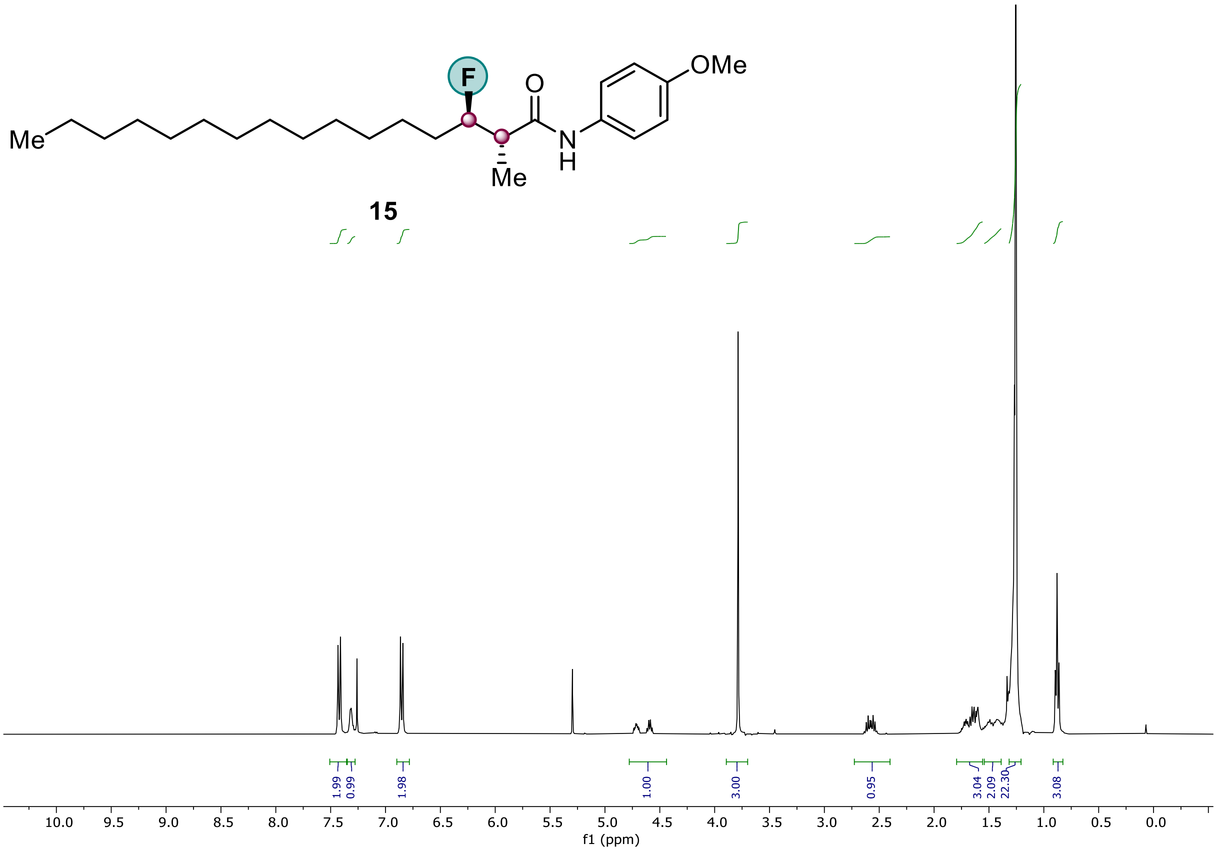

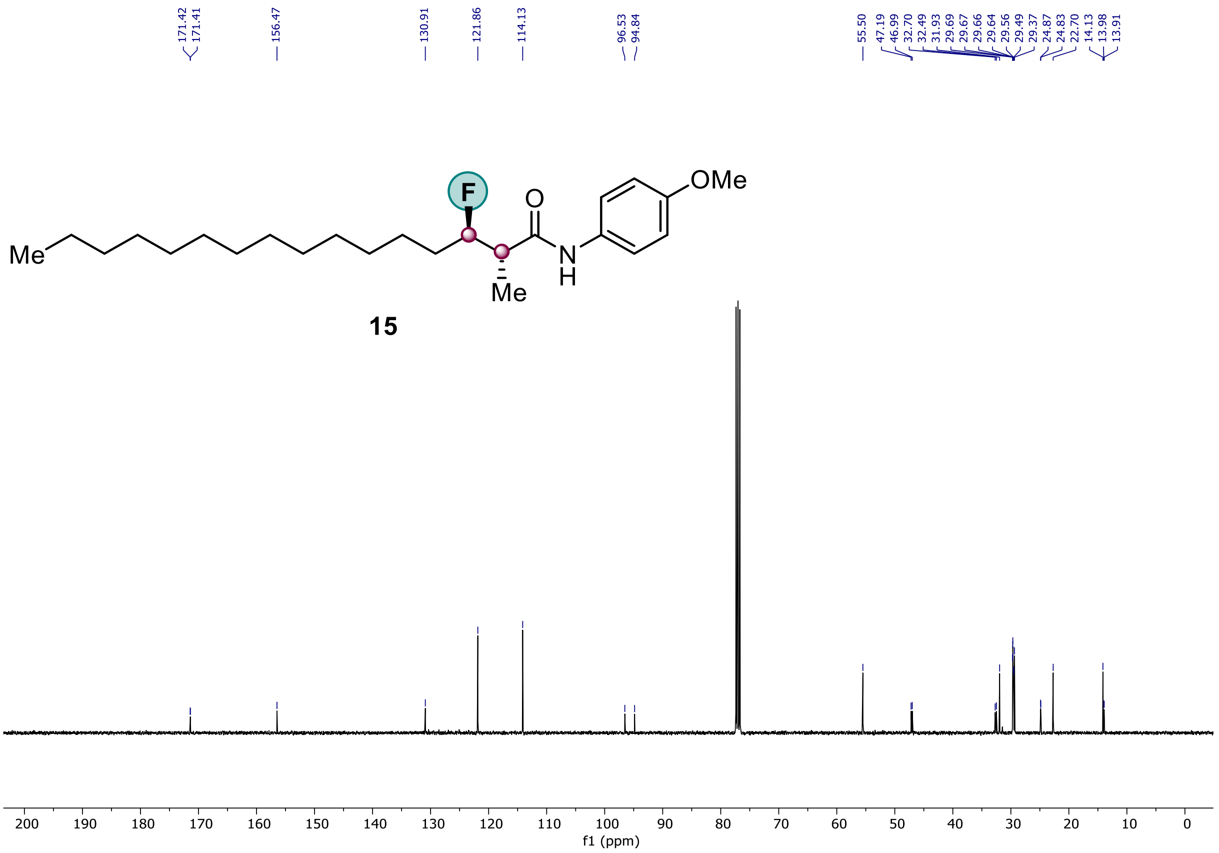

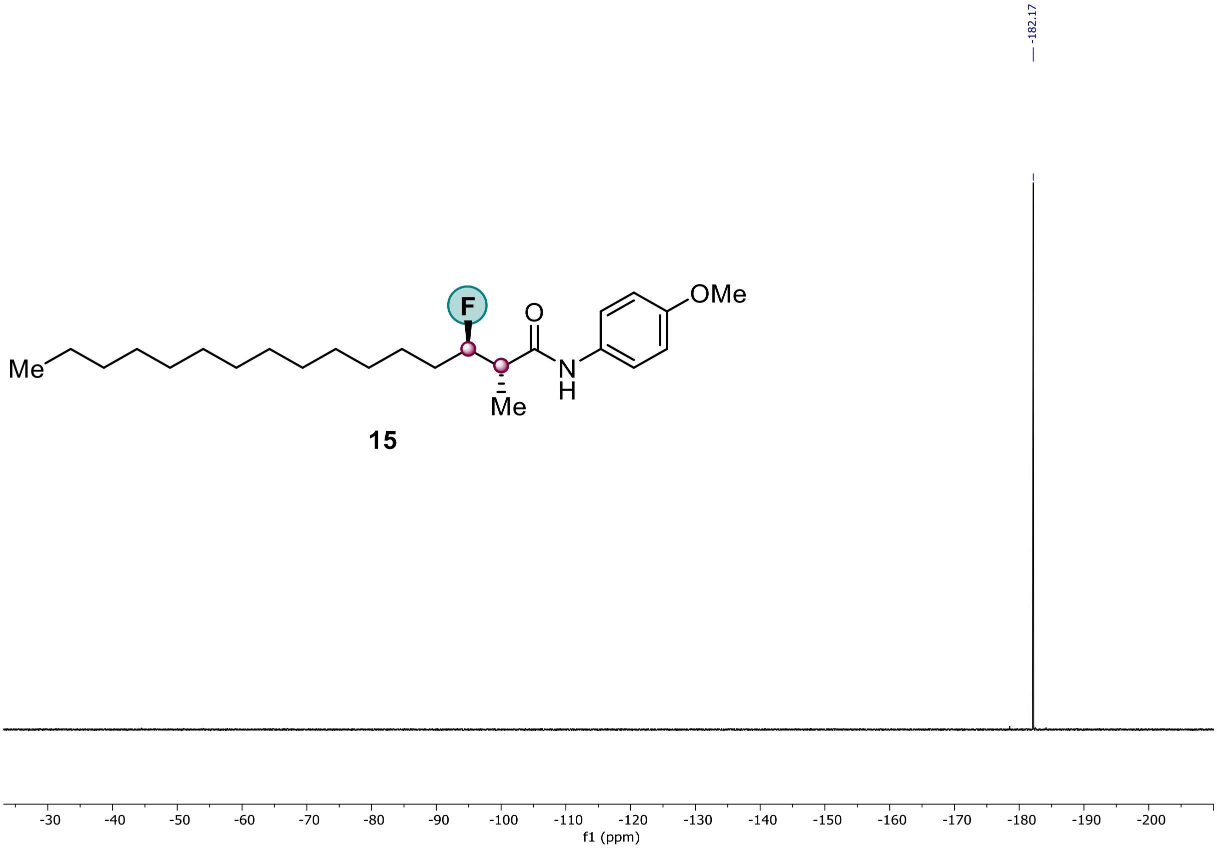
-182.17
F
O
OMe
Me
N
H
Me
15
-30
-40
-50
-60
-70
-80
-90
-100
-110
-120
-130
-140
-150
-160
-170
-180
-190
-200
f1 (ppm)

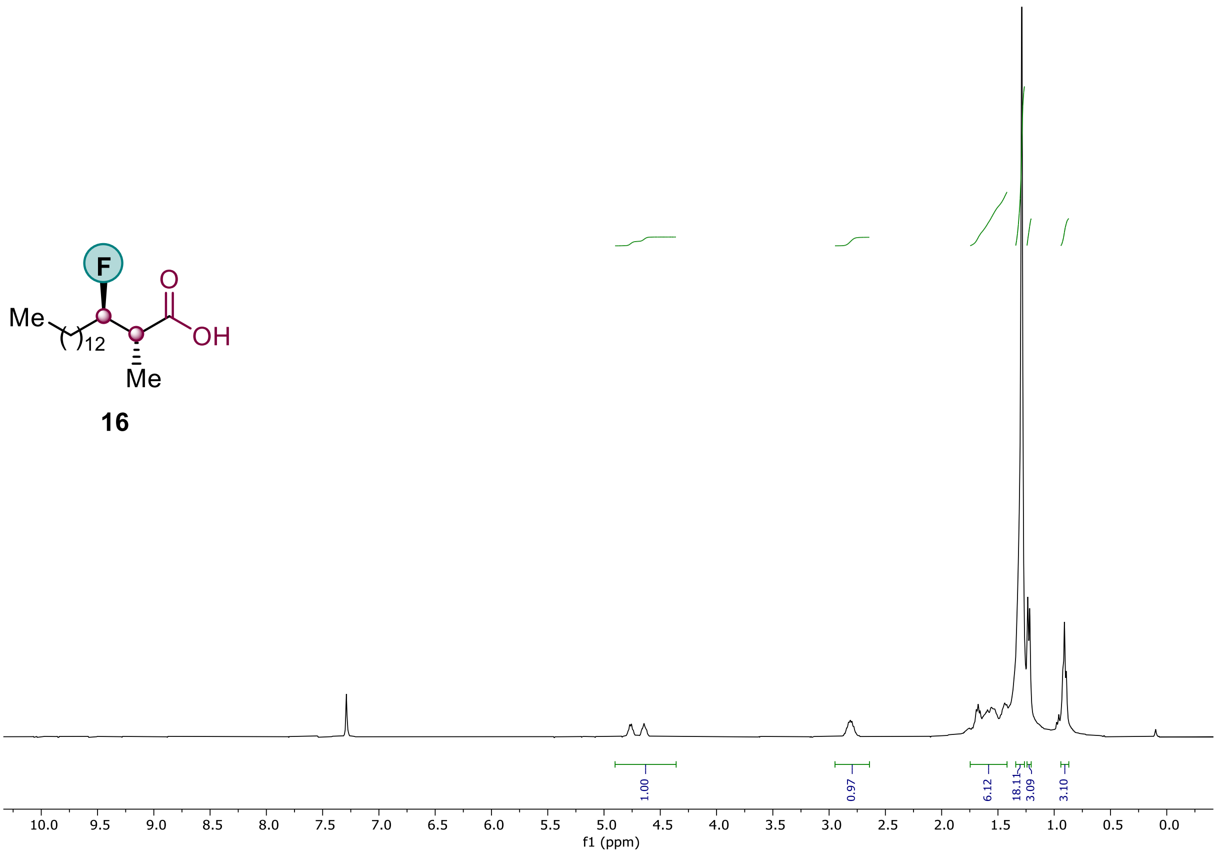

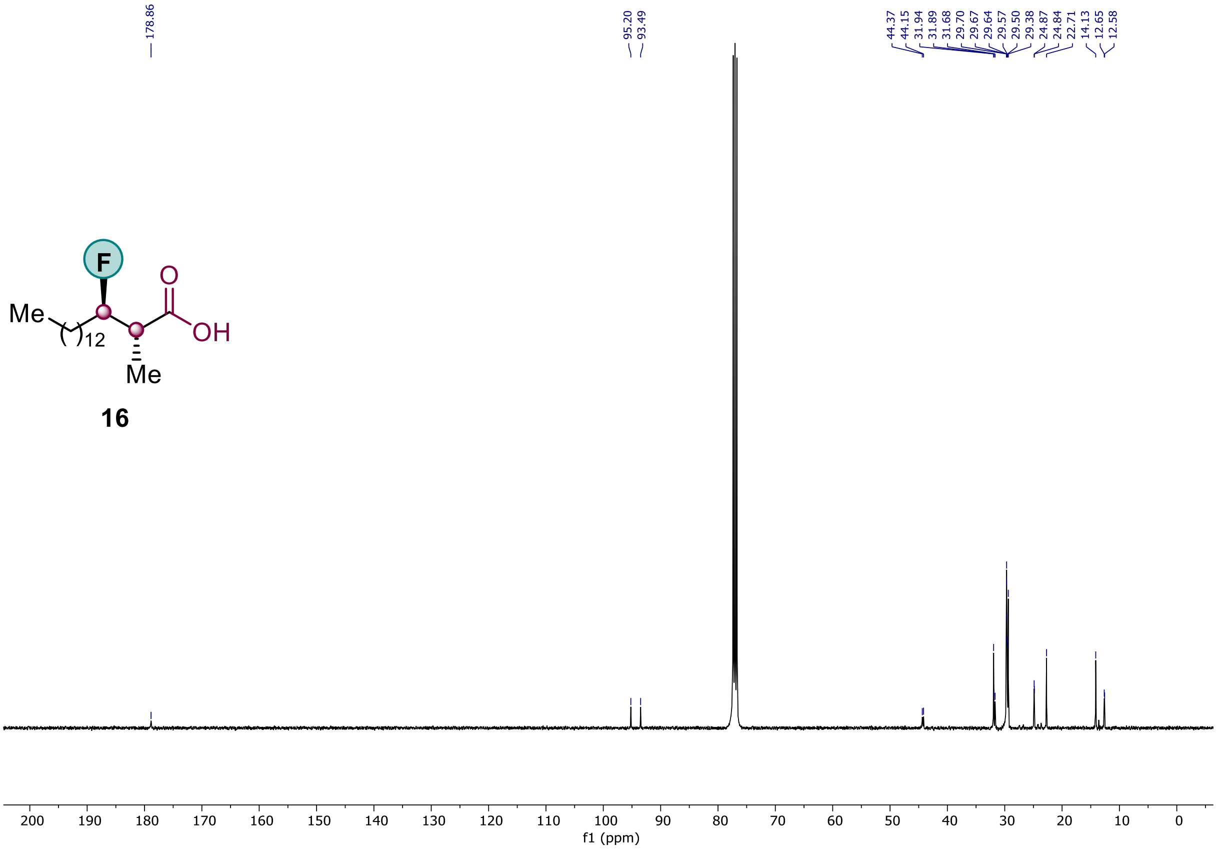

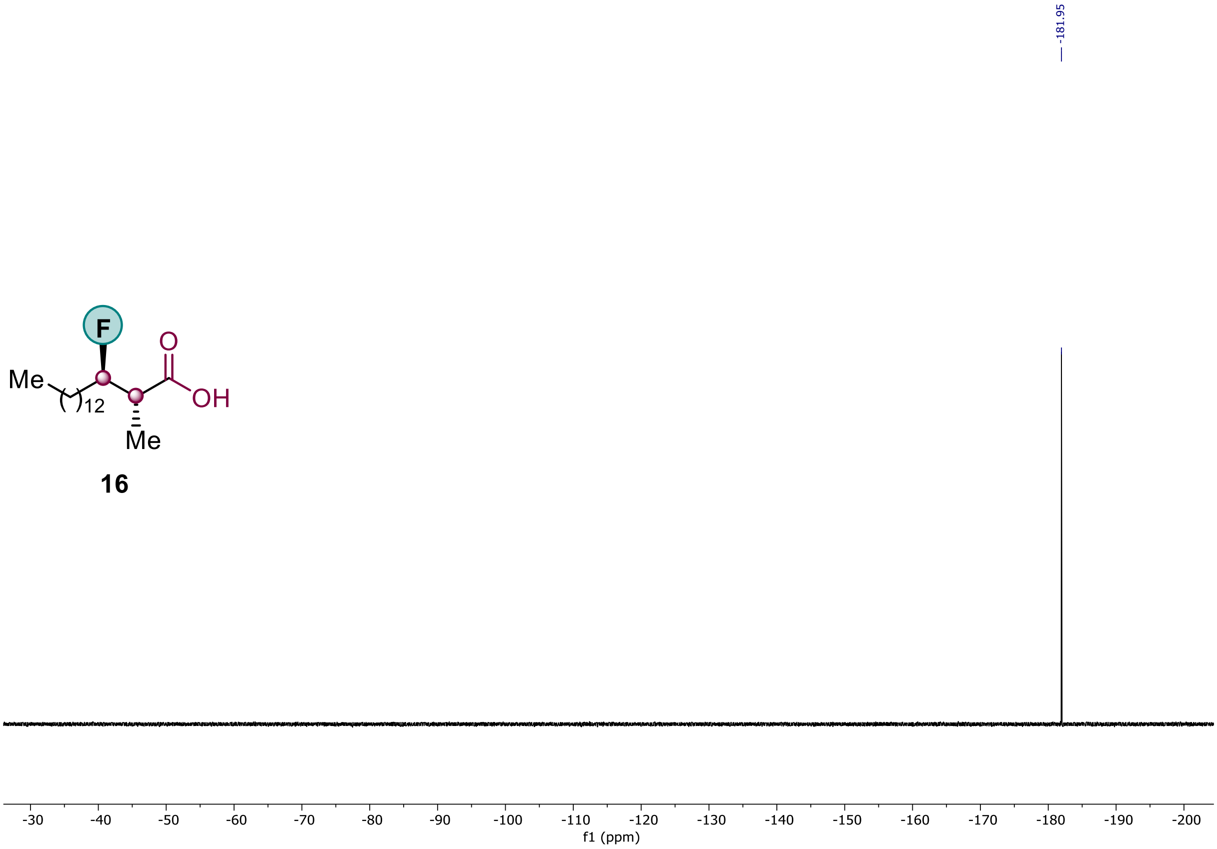
-181.95
F
O
Me
12
OH
Me
-30
-40
-50
-60
-70
-80
-90
-100
-110
-120
-130
-140
-150
-160
-170
-180
-190
-200
f1 (ppm)

16

References

1. Cox, D. G.; Gurusamy, N.; Burton, D. J. Surprising stereochemical control of Wittig olefination involving reaction of fluorine-containing phosphoranium salt and aldehydes. *J. Am. Chem. Soc.* **1985**, *107*, 2811–2812.

2. Li, Y.; Nie, W.; Chang, Z.; Wang, J.-W.; Lu, X.; Fu, Y. Cobalt-catalysed enantioselective C(sp^3)–C(sp^3) coupling. *Nat. Catal.* **2021**, *4*, 901–911.

3. Hu, J.; Han, X.; Yuan, Y.; Shi, Z. Stereoselective Synthesis of Z Fluoroalkenes through Copper-Catalyzed Hydrodefluorination of gem-Difluoroalkenes with Water. *Angew. Chem. Int. Ed.* **2017**, *56*, 13342–13346.

4. Bera, S.; Fan, C; Hu, X. Enantio- and diastereoselective construction of vicinal C(sp^3) centres via nickel-catalysed hydroalkylation of alkenes. *Nat. Catal.* **2022**, *5*, 1180–1187.

5. Chen, C; Fu, G. C. Copper-catalysed enantioconvergent alkylation of oxygen nucleophiles. *Nature* **2023**, *618*, 301–307.

6. Liang, F.; Chen, N.; Cheng, K.; Wang, Q. N-Heterocyclic Carbene and Manganese Synergistic Catalysis: A Three-Component Radical Acylmonofluoroalkylation of Alkenes. *Org. Lett.* **2023**, *25*, 8168–8172.

7. Fusini, G.; Barsanti, D.; Angelici, G.; Casotti, G.; Canale, A.; Benelli, G.; Lucchi, A; Carpita, A. Identification and Synthesis of New Sex-Specific Components of Olive Fruit Fly (*Bactrocera Oleae*) Female Rectal Gland, through Original Negishi Reactions on Supported Catalysts. *Tetrahedron* **2018**, *74*, 4381–4389.

8. Batsanov, A. S.; Howard, J. A. K.; Lightfoot, A. P.; Twiddle, S. J. R.; Whiting, A. Stereoselective Chloro-Deboronation Reactions Induced by Substituted Pyridine–Iodine Chloride Complexes. *Eur. J. Org. Chem.* **2005**, 1876–1883.

9. Hu, J.; Zhao, Y.; Shi, Z. Highly Tunable Multi-Borylation of Gem-Difluoroalkenes via Copper Catalysis. *Nat. Catal.* **2018**, *1*, 860–869.

10. Hayashi, S.; Nakai, T.; Ishikawa, N.; Burton, D. J.; Naae, D. G.; Kesling, H. S. Convenient procedures for conversion of carbonyl compounds to gem-difluoroolefins and their selective reductions to monfluoroolefins. *Chem. Lett.* **1979**, *8*, 983-986.
